# Comparative efficacy and safety of second-line treatments for advanced non-small cell lung cancer with wild-type or unknown status for epidermal growth factor receptor: a systematic review and network meta-analysis

**DOI:** 10.1186/s12916-017-0954-x

**Published:** 2017-10-30

**Authors:** Perrine Créquit, Anna Chaimani, Amélie Yavchitz, Nassima Attiche, Jacques Cadranel, Ludovic Trinquart, Philippe Ravaud

**Affiliations:** 1 Centre de Recherche Epidémiologie et Statistique Paris Sorbonne Cité, INSERM U1153, Paris, France; 2 Université Paris Descartes – Sorbonne Paris cité, Paris, France; 3 Centre d’Epidémiologie Clinique, Assistance Publique-Hôpitaux de Paris, Hôpital Hôtel-Dieu, Paris, France; 4 Service de Pneumologie, Assistance Publique-Hôpitaux de Paris, Hôpital Tenon, Paris, France; 5 Cochrane France, Paris, France; 6 Service d’Anesthésie-Réanimation, Hôpital Européen Georges Pompidou, Assistance Publique-Hôpitaux de Paris, Paris, France; 7 Sorbonne Universités, UPMC Univ., Paris 06, GRC-04, Théranoscan, Paris, France; 8 Boston University School of Public Health, Department of Biostatistics, Boston, MA USA; 9 Department of Epidemiology, Mailman School of Public Health, Columbia University, New York, NY USA; 10 Centre d’Epidémiologie Clinique, Hôpital Hôtel-Dieu, 1 place du Parvis Notre Dame, 75004 Paris, France

**Keywords:** Systematic review, Comparative effectiveness review, NSCLC, Immunotherapy, Treatments, Wild-type EGFR

## Abstract

**Background:**

Docetaxel, pemetrexed, erlotinib, and gefitinib are recommended as second-line treatment for advanced non-small cell lung cancer (NSCLC) with wild-type or unknown status for epidermal growth factor receptor (EGFR). However, the number of published randomized clinical trials (RCTs) on this topic is increasing. Our objective was to assess the comparative effectiveness and tolerability of all second-line treatments for advanced NSCLC with wild-type or unknown status for EGFR by a systematic review and network meta-analysis.

**Methods:**

MEDLINE, EMBASE, CENTRAL, ClinicalTrials.gov, and the US Food and Drug Administration website, as well as other sources, were searched for available reports up to June 6, 2017. Two reviewers independently selected published and unpublished reports of RCTs comparing any second-line treatments, extracted data and assessed the risk of bias of all included trials. We performed a Bayesian network meta-analysis. The primary outcomes were overall survival (OS) and progression-free survival (PFS). Secondary outcomes included objective response (ObR), the number of serious adverse events, and quality of life.

**Results:**

We included 102 RCTs involving 36,058 patients (62% male, median age 61 years, 81% with stage IV cancer, 80% smokers, and 92% with performance status 0–1). We revealed a differential reporting of outcomes between efficacy and safety outcomes. Half of the trials reported safety outcomes and less than 20% quality of life. For OS, nivolumab was more effective than docetaxel (hazard ratio (HR) 0.69, 95% credible interval (CrI) 0.56–0.83), pemetrexed (0.67, 0.52–0.83), erlotinib (0.68, 0.53–0.86), and gefitinib (0.66, 0.53–0.83). Pembrolizumab, atezolizumab, and pemetrexed plus erlotinib were also significantly more effective than docetaxel, pemetrexed, erlotinib, and gefitinib. For PFS, erlotinib plus cabozantinib was more effective than docetaxel (HR 0.39, 95% CrI 0.18–0.84), pemetrexed (0.38, 0.18–0.82), erlotinib (0.37, 0.18–0.78), and gefitinib (0.38, 0.18–0.82). Cabozantinib and pemetrexed plus erlotinib were also significantly more effective than the four recommended treatments. For ObR, no treatment was significantly more effective. The effectiveness of the four recommended treatments was similar and they were ranked among the 25 less-effective treatments. For safety, evidence is insufficient to draw certain conclusions.

**Conclusions:**

Nivolumab, pembrolizumab, atezolizumab, and pemetrexed plus erlotinib may be the most effective second-line treatments for NSCLC in terms of OS. The four recommended treatments seem to have relatively poor performance. However, the impact on life expectancy of immunotherapy versus other treatments should be further explored by future analyses, and more trials comparing the novel treatments are needed to reduce uncertainty in these results.

**Trial registration:**

Registration number: PROSPERO (CRD42015017592)

**Electronic supplementary material:**

The online version of this article (doi:10.1186/s12916-017-0954-x) contains supplementary material, which is available to authorized users.

## Background

Lung cancer remains the leading cause of cancer-related death worldwide, with a lower than 15% 5-year survival [1]. Further, it is the fifth leading cause of disability-adjusted life years in developed countries [2]. Non-small cell lung cancer (NSCLC) represents approximately 85% of lung cancer cases, with most patients in Western populations having wild-type or unknown status for epidermal growth factor receptor (EGFR) and do not present anaplastic lymphoma kinase gene rearrangement [3]. Most patients receive a diagnosis of advanced-stage disease and are candidates for palliative systemic therapy.

Patients with Eastern Cooperative Oncology Group performance status 0–2 with disease progression after first-line chemotherapy are given second-line treatments. In this setting, the American Society of Clinical Oncology (ASCO) clinical practice guidelines, updated in August 2015, recommend two cytotoxic drugs, docetaxel and pemetrexed (only for non-squamous cell carcinoma (NSCC) currently), and two EGFR-tyrosine kinase inhibitors, erlotinib and gefitinib [4]. However, several new treatments have been approved by the US Food and Drug Administration (FDA), including a combination of docetaxel and ramucirumab, nivolumab, pembrolizumab, and atezolizumab, and more than 40 treatments have been assessed in randomized clinical trials (RCTs) for second-line treatment of advanced NSCLC [5].

Conventional meta-analyses have only partially captured the available evidence for treatment of advanced NSCLC. Specifically, 29 systematic reviews were published between 2009 and 2015, but did not incorporate approximately 40% of the existing evidence in terms of alternative treatments and available trials [5]. Therefore, we need to consider the broader picture and simultaneously assess all treatments evaluated for this condition [6]. The aforementioned treatments approved recently have only been compared to docetaxel, and no trial has compared them with each other. Network meta-analysis (NMA) bypasses this limitation and can inform about these contrasts via indirect evidence, given that at least one trial has compared these treatments against other comparators. Two NMAs have been published, but they focused on small subsets of treatments (only four and six treatments, respectively) [7, 8]. Thus, we lack a comparative synthesis in this setting to assist clinical decision-making.

In this paper, we present an NMA comparing the relative efficacy and safety of all available treatments for second-line treatment of advanced NSCLC in patients with wild-type or unknown status for EGFR.

## Methods

### Systematic review

The present report has been prepared following the recommendations of the Cochrane Comparing Multiple Interventions Methods Group [9] and the PRISMA extension statement for systematic reviews incorporating NMAs [10]. The protocol of this NMA was also registered with PROSPERO (no. CRD42015017592).

#### Eligibility criteria

We considered RCTs assessing second-line treatments in patients with advanced NSCLC (stage IIIB unsuitable for radical radiotherapy or surgery and stage IV) with wild-type or unknown status for EGFR. Trials in which patients in the control arm received chemotherapy (e.g., docetaxel or pemetrexed) at the investigators’ discretion were included for the secondary analysis considering treatment categories. We also considered trials including both second- and third-line therapy, because there is no clinical reason to presume that these minority patients in third-line could not be randomized to any of the treatments. If a multi-arm trial compared one drug to two different dosages of another drug, we retained the usual treatment dosage or that corresponding to the 3-week scheme of administration. We excluded trials comparing different administration schemes of the same drug and those assessing combinations of different chemotherapy drugs.

#### Outcome measures

The primary outcomes were overall survival (OS) and progression-free survival (PFS). Secondary outcomes were objective response (ObR), defined as a complete response or a partial response according to the Response Evaluation Criteria in Solid Tumors (RECIST) [11], the number of serious adverse events (SAEs) as defined at ClinicalTrials.gov (see Additional file 1: Appendix 1 for a description), and quality of life (QoL) considering any scale used in eligible trials.

#### Data sources and searches

We searched for eligible RCTs up to June 6, 2017, in the Cochrane Central Register of Controlled Trials (CENTRAL), MEDLINE, and EMBASE with no restriction on language, status, or year of publication. To identify additional trials and unpublished data, we screened previous systematic reviews, reference lists of all selected trials, conference abstracts, both industry and non-industry trial registries and results databases, regulatory agency online databases, and health technology assessment agencies [12]. The full search strategy is available in Additional file 1: Appendix 2.

#### Selection of trial and data extraction

Two investigators (PC and AY) independently examined titles, abstracts, and full texts to assess the eligibility of each report. Disagreements were discussed with a third author (LT). The same authors extracted data independently by using a standardized form. With several reports pertaining to the same trial, we gave priority to the first available source among regulatory agency reports, results posted at ClinicalTrials.gov, published articles, reports from pharmaceutical companies, and conference abstracts. For conference abstracts and trials without published results, we contacted trial investigators to request the final report, if available, and the outcome data when missing from the available reports. The data extracted for each trial are available in Additional file 1: Appendix 3. When attempts to retrieve data from trialists failed, we obtained the necessary data from published Kaplan–Meier curves [13]. For each eligible trial, we extracted information related to the publication, trial and patient characteristics, description of the interventions, and outcome data (Additional file 1: Appendix 3). To evaluate the synthesis assumption for NMA, we extracted data on the effect modifiers of sex, histology, ethnicity, and smoking status.

#### Risk of bias assessment

Two investigators (PC and AY) assessed the risk of bias of the trials by using the risk of bias tool of The Cochrane Collaboration (Additional file 1: Appendix 4) [14]. Disagreements were discussed with a third author (LT). The considerations for defining the overall risk of bias for each trial are available in Additional file 1: Appendix 4.

### Network geometry

We produced network diagrams to show the amount of evidence available for each outcome. The size of the nodes is proportional to the total number of patients allocated to the corresponding treatment across all trials and the width of the lines is proportional to the total number of RCTs evaluating the corresponding treatment comparison. Our primary analyses considered treatments separately and in a secondary analysis, we grouped treatments into five categories, namely cytotoxic monochemotherapy, targeted treatment, immunotherapy, combination of a cytotoxic monochemotherapy and a targeted treatment, and combination of two targeted treatments (see Additional file 1: Appendix 5 for details on the classification). Trials in which patients in the control arm received chemotherapy (e.g., docetaxel or pemetrexed) at the investigators’ discretion contributed to the secondary analyses only.

### Data synthesis

We assessed the clinical and methodological heterogeneity of the eligible trials within and across the different comparisons in terms of trial and population characteristics. Results from the NMA were valid if the assumption of transitivity (i.e., one can infer the relative effect between two interventions via one or more intermediate comparators) is plausible [15–17]. To infer the plausibility of transitivity, we considered the similarity of the distribution of the potential effect modifiers across the different pairwise comparisons.

We used the hazard ratio (HR) as an effect measure for survival outcomes and the odds ratio (OR) for ObR and SAEs, estimating 95% credible intervals (95% CrIs). Trial- and arm-level data were used for survival and binary outcomes, respectively. For ObR, we assumed that participants with missing outcome data did not respond to the intervention. First, we conducted random-effects pairwise meta-analyses for every comparison involving at least two trials. Statistical heterogeneity was assessed by visual inspection of the forest plots and by considering the I^2^ statistic and the magnitude of the between-trial standard deviation τ. Then, we performed NMA by using a random-effects hierarchical model. We assumed that the different comparisons within each outcome shared the same heterogeneity parameter. For clarity, we present the relative effects between a subset of treatments (recommended and approved treatments or treatments with unexpected results) in league tables, with other relative effects reported in forest plots in the supplementary files. Additionally, for every treatment, we estimated the probability of being at each possible rank and used the SUrface under the Cumulative RAnking (SUCRA) curves to infer the relative ranking of the treatments [18]. We assessed statistical inconsistency (i.e., the agreement between direct and indirect evidence) by two approaches, namely (1) the loop-specific approach, which evaluates inconsistency in every closed loop of evidence [19, 20], and (2) the design-by-treatment interaction model, which assesses the presence of inconsistency in the entire network [19, 20].

We performed pre-specified subgroup analyses considering histologic subtypes (NSCC vs. squamous cell carcinoma (SCC)), ethnicity (Asian vs. non-Asian), and EGFR mutation status (wild-type vs. unknown status).

#### Reporting bias and credibility of the evidence

To assess the presence of small-study effects in the network for the primary outcomes we drew ‘comparison-adjusted’ funnel plots considering that small trials might give more favorable results for the sponsored drugs than larger trials and used network meta-regression models with the variance of the trials as a covariate [21, 22]. We defined a drug as being ‘sponsored’ if the drug was developed and marketed by the pharmaceutical company that sponsored the trial. We also evaluated the credibility of the evidence from network meta-analysis using the approach suggested by Salanti et al. [23], which extends the GRADE system into NMA. Details on the method and its implementations are available in Additional file 2: Appendix Figure S7.

#### Software

All analyses involved the use of R v3.0.2 [24] (metafor package) [25], WinBUGS v1.4.3 [26], and Stata v13 (network [27] and network graphs packages [28]). The WinBuGS codes can be found in Additional file 1: Appendix 12.

## Results

### Selection of trials

**Fig. 1:**
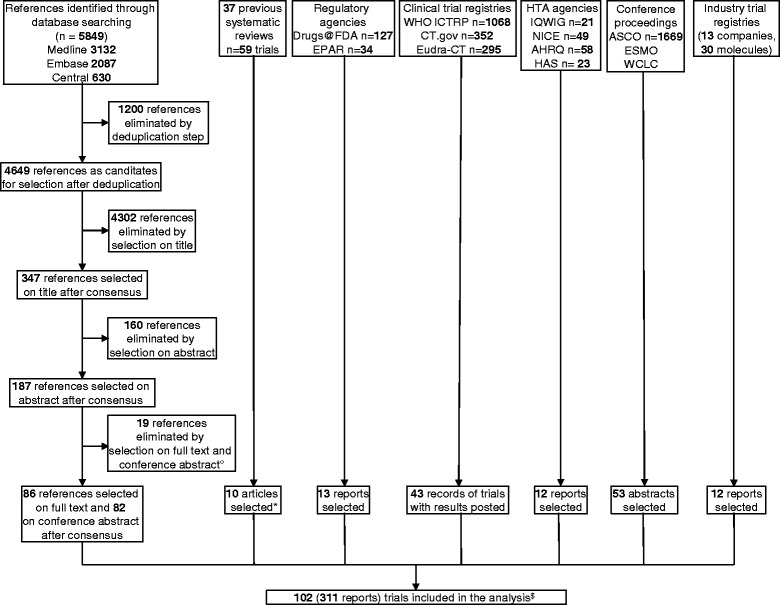
Flow diagram of the selection of trials for second-line treatments in advanced NSCLC with wild-type or unknown status for EGFR. ° Details in Appendix 6; * Additional articles not identified by searching bibliographic databases; ^$^ 5 trials (10 reports) with chemotherapy (i.e., docetaxel or pemetrexed) at the investigators’ discretion. The last search for randomized trials was conducted on June 6, 2017

We included 102 trials involving a total of 36,058 patients (Fig. 1, Additional file 1: Appendix 6), of which data for 87 trials were published and 15 (15%) unpublished (Additional file 1: Appendix 7). For 13 trials (13%), we obtained outcome data from available survival curves or the authors (Additional file 1: Appendix 3). Two eligible trials were excluded from the quantitative analyses since one was disconnected from the rest of the network (HANSHIN, 2015) and another involved suspected research misconduct (Zhang, 2015) [29]. In the latter trial, the reported HR and survival curves were contradictory and the authors did not answer our questions.

### Characteristics of eligible trials

The characteristics of the 102 individual trials are available in Additional file 1: Appendix 8 and their summary characteristics are provided in Table 1. Most trials were multi-center with industry funding. The majority of patients (29,864 patients, 83%) were randomized in phase III trials. Overall, 62% of patients were male, the mean age was 61 years, 81% had stage IV cancer, 80% were smokers, and 92% had a performance status score 0–1. In all, 26 trials (27%), including 4659 patients (13%), involved only Asians and 78 (76%) were of patients with both SCC or NSCC. The main results of these trials are provided in Additional file 1: Appendix 9.

**Table 1:** Trial and patient summary characteristics for the 102 eligible RCTs (36,058 patients) of second-line treatments for advanced NSCLC with wild-type or unknown status for EGFR

Trial characteristics	No. of trials (%)
Phase of trial	
II	48 (47)
III	53 (52)
Unclear	1 (1)
No. of centers	
Multi-center	88 (86)
Single-center	7 (7)
Unclear	7 (7)
Funding source	
Industry	69 (68)
Non-industry	13 (13)
Both	2 (2)
Not reported	18 (17)
No. of patients (median [Q1–Q3]; mean)	167 [105–528]; 354
Line therapy	
Second-line only	50 (49)
Second- and third-line	41 (40)
Proportion of patients with third-line therapy^a^	31%
Not specified	11 (11)
Population characteristics	
Geographic origin	
Most Western patients^b^	54 (53)
Asian patients only	26 (25)
Not specified	22 (22)
Proportion of Asian patients^a^	14%
Trials in specific histology subtype	
NSCC	20 (20)
SCC	4 (4)
Proportion of patients with SCC^a^	27%
Molecular characteristics at baseline	
Unknown status for EGFR	93 (91)
EGFR wild-type	6 (6)
KRAS mutation	3 (3)
Patient characteristics^a^	
Age, years	61
Male	62%
Stage IV cancer	81%
Patients PS 2	8%
Former or current smoker	80%
Patients receiving second-line treatment	85%

^a^Mean over trials

^b^ ≥ 60% of Caucasian patients

*EGFR* epidermal growth factor receptor, *NSCC* non-squamous cell carcinoma, *PS* Eastern Cooperative Oncology Group performance status, *SCC* squamous cell carcinoma

### Risk of bias

Only 47 trials (46%) described an adequate random sequence generation and 37 (36%) an adequate treatment allocation concealment. Patients and care providers were blinded in 29 trials (28%), and outcome assessors in 41 trials (40%). A detailed description of the risk of bias results is provided in Additional file 1: Appendix 4.

### Network geometry

**Fig. 2:**
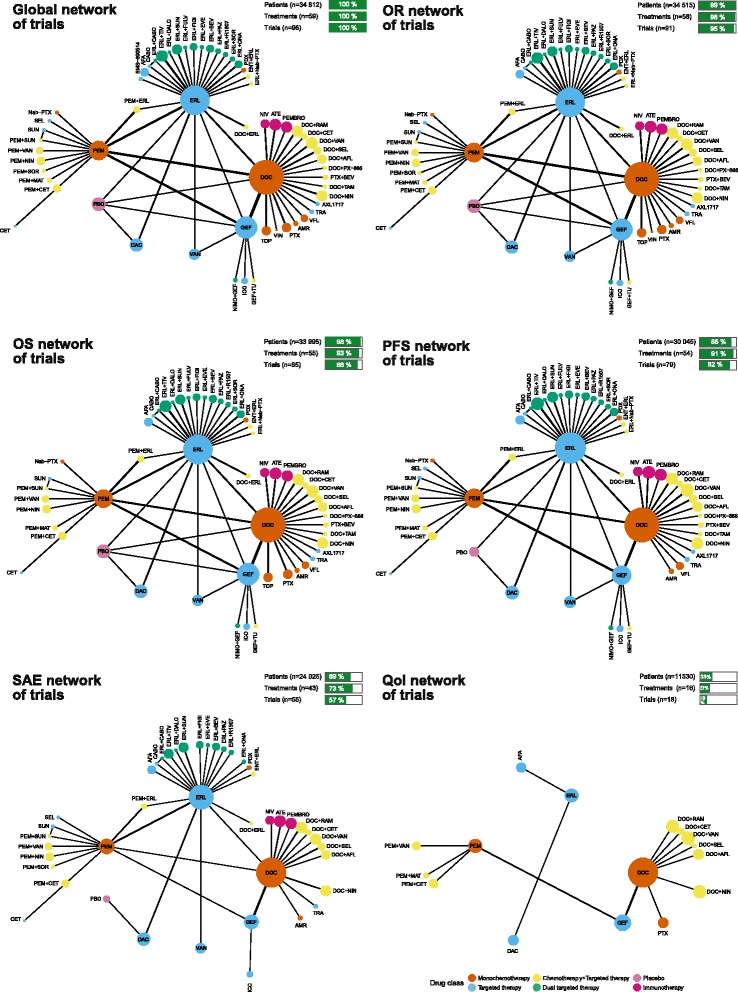
Network graphs of trials assessing second-line treatments in advanced NSCLC with wild-type or unknown status for EGFR for all eligible trials, ObR, OS, PFS, SAEs, and QoL. The five trials with chemotherapy (i.e., docetaxel or pemetrexed) at the investigators’ discretion and the HANSHIN trial were excluded from these networks. The thickness of the lines is proportional to the number of trials evaluating each comparison. The size of the nodes is proportional to the number of patients allocated to the corresponding treatment. *AFA* afatinib, *AFL* aflibercept, *AMR* amrubicin, *ATE* atezolizumab, *BEV* bevacizumab, *CABO* cabozantinib, *CET* cetuximab, *DAC* dacomitinib, *DALO* dalotuzumab, *DOC* docetaxel, *ENT* entinostat, *ERL* erlotinib, *EVE* everolimus, *FIGI* figitumumab, *FULV* fulvestrant, *GEF* gefitinib, *ICO* icotinib, *MAT* matuzumab, *Nab-PTX* nab-paclitaxel, *NIMO* nimotuzumab, *NIN* nintedanib, *NIV* nivolumab, *ONA* onartuzumab, *PAZ* pazopanib, *PBO* placebo, *PDX* pralatrexate, *PEM* pemetrexed, *PEMBRO* pembrolizumab, *PTX* paclitaxel, *RAM* ramucirumab, *SEL* selumetinib, *SOR* sorafenib, *SUN* sunitinib, *TAM* tamoxifen, *TIV* tivantinib, *TOP* topotecan, *TRA* trametinib, *TU* tegafur-uracil, *VAN* vandetanib, *VFL* vinflunine, *VIN* vinorelbine

Figure 2 shows six network diagrams representing the global network (i.e., all trials regardless of data availability) and the networks with data for ObR, OS, PFS, SAEs, and QoL. The four recommended treatments (docetaxel, pemetrexed, erlotinib, and gefitinib) were thoroughly compared against each other. However, novel treatments were compared against only one alternative intervention. ObR and OS were reported in almost all trials (91 trials (95%) and 85 trials (88%), respectively) and PFS in 79 trials (82%), whereas SAEs were reported in 55 trials (57%) and QoL in only 18 trials (19%). Moreover, more than six different scales for QoL were used in these trials, so we did not synthesize the data for QoL.

### Relative effects and ranking

We did not observe any important clinical differences in distribution of effect modifiers between trials comparing different sets of interventions; thus, we considered that the transitivity assumption was likely met (Table 1, see Additional file 1: Appendix 10 for patient characteristics).

#### Overall survival

Pairwise meta-analysis suggested a statistically significant OS benefit of nivolumab, atezolizumab, and docetaxel plus ramucirumab against docetaxel and pemetrexed plus erlotinib against pemetrexed. Additional file 1: Appendix 10 provides the results from pairwise meta-analyses for all available direct comparisons. For five comparisons, the heterogeneity standard deviation was greater than 0.10.

Table 2 presents all relative effects for the pre-specified treatments from the NMA. Relative effects of all other treatments against these treatments are presented in forest plots in Additional file 2: Appendix Figure S1. Eighteen treatments were significantly more effective than placebo in terms of OS. Nivolumab was more effective than docetaxel (HR 0.69, 95% CrI 0.56–0.83), pemetrexed (HR 0.67, 95% CrI 0.52–0.83), erlotinib (HR 0.68, 95% CrI 0.53–0.86), and gefitinib (HR 0.66, 95% CrI 0.53–0.83). Pembrolizumab, atezolizumab, and pemetrexed plus erlotinib were also significantly more effective than docetaxel, pemetrexed, erlotinib, and gefitinib. The τ value was close to 0 (τ = 0.04). Erlotinib plus cabozantinib, nivolumab, pembrolizumab, atezolizumab, and pemetrexed plus erlotinib represented the five most effective treatments in terms of OS (Additional file 1: Appendix Figure S2). The four recommended treatments were ranked in the 30th position (Additional file 1: Appendix Figure S3). Proper methodology to translate these results into absolute effects still remains to be developed. Nevertheless, we could consider as an illustrative – but not yet conclusive – example of the absolute effects the difference in restricted mean survival at 18 months of immunotherapy versus docetaxel (Additional file 1: Appendix 13). As the evidence presented in this systematic review does not allow for any definitive conclusions, the actual impact on life expectancy should be further explored by future analyses.

**Table 2:**
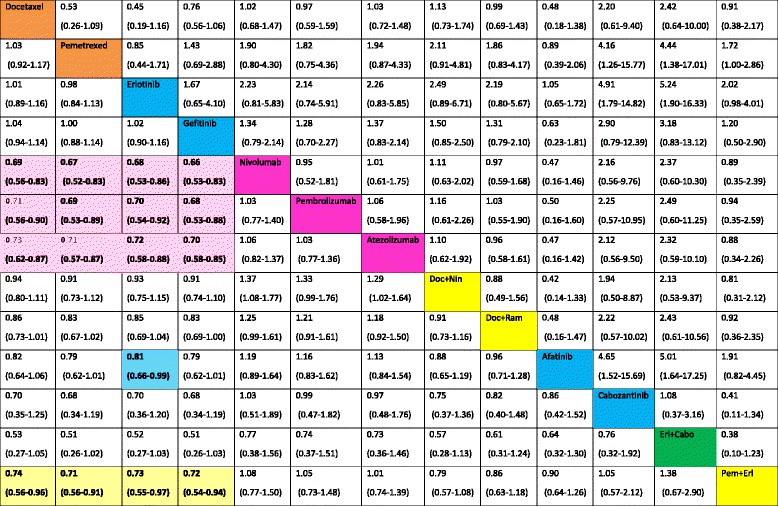
Hazard ratios (HRs) for overall survival (OS, lower triangle) and odds ratios for serious adverse events (SAEs, upper triangle) with their 95% credible intervals (95% CrIs) derived from network meta-analysis of 13 second-line treatments for NSCLC with wild-type or unknown status for EGFR

Treatment categories

*Pembro* pembrolizumab; *Ate* atezolizumab; *Doc* docetaxel; *Erl* erlotinib; *Nin* nintedanib; *Ram* ramucirumab; *Cabo* cabozantinib

The direction of the reported relative effects in each cell is defined as treatment on the right vs. treatment on the left. Values < 1 favor the intervention on the right. Values in parenthesis are 95% credible intervals (95% CrIs). Colored cells correspond to statistically significant relative effects for the respective treatment categories. For instance, nivolumab was more effective than docetaxel in terms of OS (HR 0.69, 95% CrI 0.56–0.83)

#### Progression-free survival

Pairwise meta-analyses suggested that, for PFS, treatment combinations often performed better when compared to a single treatment (Additional file 1: Appendix 10). For most comparisons, heterogeneity was 0. The largest heterogeneity was for gefitinib versus pemetrexed (τ = 0.51). According to the NMA results, erlotinib plus cabozantinib was more effective than docetaxel (HR 0.39, 95% CrI 0.18–0.84), pemetrexed (HR 0.38, 95% CrI 0.18–0.82), erlotinib (HR 0.37, 95% CrI 0.18–0.78), and gefitinib (HR 0.38, 95% CrI 0.18–0.82) (Tables 2 and 3, Additional file 2: Appendix Figure S1). Cabozantinib and pemetrexed plus erlotinib were also significantly more effective than docetaxel, pemetrexed, erlotinib, and gefitinib. The heterogeneity was larger as compared with OS (τ = 0.15). The four recommended treatments were ranked around the 40th position (Additional file 2: Appendix Figure S2). Additionally, combinations of dual-targeted therapies (erlotinib plus pazopanib) or chemotherapy plus targeted therapy (paclitaxel plus bevacizumab) appeared to be among the most effective treatments (Additional file 2: Appendix Figure S3).

**Table 3:**
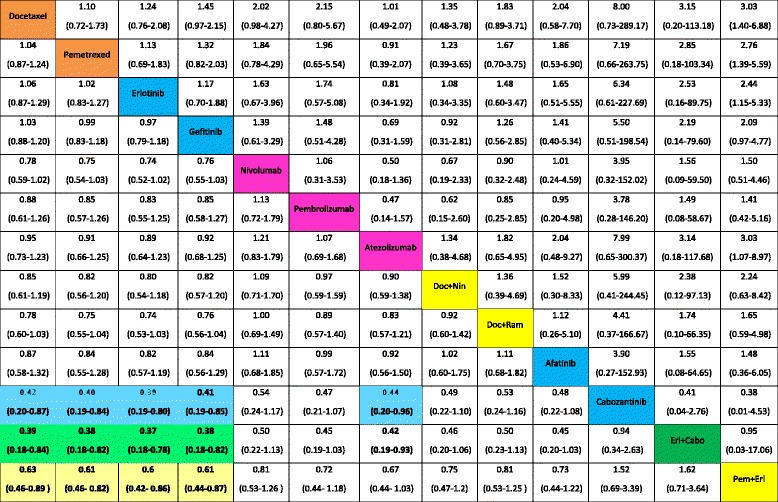
Hazard ratios (HRs) for progression-free survival (PFS, lower triangle) and odds ratios for objective response (ObR, upper triangle) with their 95% CrIs derived from network meta-analysis of 13 second-line treatments for NSCLC with wild-type or unknown status for EGFR

Treatment categories

*Pembro* pembrolizumab; *Ate* atezolizumab; *Doc* docetaxel; *Erl* erlotinib; *Nin* nintedanib; *Ram* ramucirumab; *Cabo* cabozantinib

The direction of the reported relative effects in each cell is defined as treatment on the right vs. treatment on the left. Values < 1 favor the intervention on the right. Values in parenthesis are 95% credible intervals (95% CrIs). Colored cells correspond to statistically significant relative effects for the respective treatment categories. For instance, cabozantinib was more effective than docetaxel in terms of PFS (HR 0.42 (95% CrI 0.20-0.87)

#### Secondary outcomes

Pairwise meta-analysis suggested a statistically significant benefit for ObR of nivolumab and some treatment combinations against a single treatment (Additional file 1: Appendix 10). For SAE, docetaxel plus selumetinib appeared more toxic than docetaxel (Additional file 1: Appendix 10). The heterogeneity was generally larger than that for the two primary outcomes for all comparisons. For ObR and SAEs, no pair of the previously mentioned treatments showed a statistically significant effect (Tables 2 and 3, Additional file 2: Appendix Figure S1). The τ value was moderate to high for ObR and moderate for SAEs [30]. Cabozantinib combined with erlotinib or alone and docetaxel plus selumetinib seemed to be among the most effective for ObR but also the most toxic (Additional file 2: Appendix Figures S2 and S3).

#### Inconsistency assessment

The design times treatment interaction model did not suggest statistical inconsistency for any outcome. Nevertheless, the *P* value was less than 10% for ObR, which indicates some inconsistency for this outcome. These results in general agreed with the loop-specific approach, finding no inconsistent loop SAEs, one loop for OS and PFS, and two loops for ObR (Additional file 2: Appendix Figure S4). A possible explanation for this inconsistency in PFS and ObR is the presence of one particular trial in these loops (the WJOG5108L trial for the loop docetaxel-erlotinib-gefitinib and the loop erlotinib-gefitinib-vandetanib, and the Lee, 2013 trial for docetaxel-erlotinib-pemetrexed), which differed greatly in patient characteristics (ethnicity, histology, and smoking status).

### Additional analyses

In subgroup analyses, we did not find any important differences in treatment effect estimates between trials of SCC and NSCC, only Asians, and of only wild-type patients (Additional file 2: Appendix Figure S5). For NSCC, immunotherapy seemed more efficacious for OS than erlotinib plus cabozantinib, docetaxel plus ramucirumab, and docetaxel plus nintedanib. For SCC, immunotherapy seemed better afatinib and docetaxel plus ramucirumab. According to the treatment categories analysis, immunotherapy and chemotherapy plus targeted therapy seemed the most effective treatments (Additional file 1: Appendix 11). Nevertheless, chemotherapy plus targeted therapy seemed more toxic.

### Reporting bias and credibility of the evidence

The funnel plot appeared symmetrical for OS but rather asymmetrical for PFS, which suggests that the sponsored treatments were favored more in small rather than larger trials (Additional file 2: Appendix Figure S6). However, the regression coefficient was not significant in the network meta-regression model that accounted for differences in trial size. This result might be explained by small-study effects not operating consistently across all comparisons, but the scarcity of the data did not allow us to explore this possibility. The GRADE evaluation suggests that the available evidence is of moderate credibility for the majority of the comparisons with respect to OS. For comparisons between immunotherapies and the combination pemetrexed plus erlotinib against the four recommended treatments, information came from low risk of bias trials with a contribution varying from 60% to 100%. On the contrary, for comparisons including either cabozantinib alone or in combination with erlotinib, information came mainly from moderate risk of bias trials. Less confidence can be placed on the results for PFS as most comparisons were rated at low or very low credibility.

## Discussion

Our review is, to date, the most comprehensive comparative effectiveness review for second-line treatments for advanced NSCLC with wild-type or unknown status for EGFR, involving 102 RCTs (36,058 patients). In terms of OS, immunotherapy (nivolumab, pembrolizumab, and atezolizumab) and the combination pemetrexed plus erlotinib seem to be more efficacious than the four recommended treatments, with no difference in effectiveness between the four recommended treatments. Other approved treatments did not have a statistically significant benefit as compared to the four recommended treatments. Our review highlighted that only half of the trials reported safety outcomes; thus, results for safety were very uncertain. Moreover, for OS, all relative effects were informed by trials at low and moderate risk of overall bias, and therefore the quality of evidence was moderate.

Our NMA has advantages over the two previous NMAs, which focused on a subset of the available treatments [7, 8]. Our exhaustive search strategy allowed us to identify (1) a large number of unpublished trials, which represented one-sixth of our data, and (2) several reports for the same trial, with a median of two reports by trial (range 1–17), which allowed us to compare results from each report and to give priority to the report corresponding to the best level of evidence. The unpublished trials were mainly phase III trials (9/15, 60%) and corresponded to negative trials assessing unsuccessfully licensed drugs or small trials (less than 100 patients); including these trials decreased the risk of publication bias and increased the power of treatment categories analysis.

Our NMA also provides the most up-to-date evidence synthesis results with last search date on June 6, 2017. Considering all the available evidence on any treatment for NSCLC that has appeared in the literature allowed us to (1) confirm the superiority of immunotherapies over all other treatments; (2) reveal highly efficacious treatment combinations (such as the combination pemetrexed plus erlotinib), which can be considered as equivalent alternatives to the new drugs although they are underrepresented in trials partly due to contradicting interests of the two pharmaceutical companies that market the two drugs; (3) investigate subgroups of patients considering histologic subtypes, such as the superiority of immunotherapies over the combination of docetaxel plus ramucirumab for NSCC, and ethnicity.

We also performed a detailed assessment of the credibility of the evidence to critically appraise our results. With respect to the five most efficacious treatments for OS, the level of evidence was higher (i.e., moderate) for nivolumab, pembrolizumab, atezolizumab, and pemetrexed plus erlotinib when compared to the four recommended treatments than for erlotinib plus cabozantinib, for which the level of evidence was low. For PFS, most comparisons were rated at low or very low credibility mainly due to lack of blinding and absence of an independent clinical endpoint adjudication committee to assess subjective outcomes, which led to very serious concerns for study limitation for many comparisons.

Moreover, considering all trials performed for a given condition facilitates the research planning analysis. Such analysis revealed a lack of head-to-head trials comparing novel treatments to each other in our network of trials. This apparent lack of direct evidence between novel treatments may help researchers plan subsequent trials (e.g., for choosing a control group). In their discussion, Neal et al. [31] wondered about the best comparator treatment for erlotinib plus cabozantinib in a phase III trial; in considering our results, nivolumab, atezolizumab, or pembrolizumab should be the best choice in considering treatment efficacy. Finally, our findings can be useful for policy-makers to prioritize future research and to facilitate the development of high-quality guidelines. Recently, the FDA modified the indication for erlotinib, restricting its use to patients with EGFR mutation [32]. Nevertheless, in our analysis, erlotinib as second-line therapy appeared to be as effective as docetaxel for OS.

Our study has several limitations. First, we did not distinguish between the different types of data; namely, we considered the 11 trials (11%) only identified through a conference abstract as the same level of evidence as published trials in the quantitative analysis. However, when available, results obtained from the different reports were very similar. Second, we could not formally assess the assumption of transitivity because, for most of treatment comparisons, there are very few trials included. However, we observed small differences in terms of tumor histology and ethnicity, which are unlikely to violate the transitivity assumption. These differences might explain the small inconsistencies found for ObR and PFS. Nevertheless, our subgroup analyses did not reveal any important differences in the relative effects when these populations were analyzed separately (Additional file 1: Appendix Figure S5). Third, for safety assessment, we focused on the number of SAEs because this allows a reproducible global assessment of severe toxicity. This outcome was actually the most frequently reported (52 of 102 trials, 51%), contrary to the number of grade 3–4 SAEs, which were only reported in 30 trials (29%). Reporting of specific adverse events is very heterogeneous across trials and did not allow the synthesis of evidence for each specific adverse event. Additionally, because of the lack of reporting of the number of SAEs across trials, we could not even infer on the effect of these treatments. Fourth, providing a broad panorama of all available evidence by considering trials performed over a 10-year period does not allow to take into account some parameters only described recently, such as some predictive biomarkers. Among the 102 selected trials, only nine were designed in a specific population in terms of biomarkers. Moreover, for NSCLC research, even in 2015, only one third of trials proposed an enrichment design and the majority of trials remained performed in an unselected population. Finally, we performed a NMA on the hazard ratios, which remain the most widely used measure of treatment effect in oncology, although this measure is probably not the most informative for patients. Alternatives to the hazard ratio have been proposed, such as the difference in restricted mean survival times [33, 34] or difference in survival rates at a pre-specified time-point [35]. However, these alternatives have never been applied, to date, to NMA as several issues remain to be solved (such as the choice of the time-point for defining the restricted mean survival or the survival rate).

## Conclusions

Our comparative effectiveness review of second-line treatments for advanced NSCLC with wild-type or unknown status for EGFR compared 61 treatments assessed in 102 trials (36,058 patients). Our NMA revealed that immunotherapy (nivolumab, pembrolizumab, and atezolizumab) and pemetrexed plus erlotinib might be more efficacious for OS than the four recommended treatments (docetaxel, pemetrexed, erlotinib, and gefitinib) and highlighted the relatively poor performance of these four treatments. The assessment of safety and patient reporting outcomes was uncertain because of a lack of reporting.

## Additional files

Additional file 1: Appendix 1. Definition used for objective response and serious adverse events. **Appendix 2.** Full search strategy. **Appendix 3.** Data extraction process. **Appendix 4.** Risk of bias assessment. **Appendix 5.** Classification of second-line treatments. **Appendix 6.** Reasons for excluding full texts and conference abstracts. **Appendix 7.** Identified reports for the eligible trials. **Appendix 8.** Characteristics of the 98 individual trials. **Appendix 9.** Results of individual trials. **Appendix 10.** Results of pairwise meta-analyses and patient characteristics across trials within each comparison. **Appendix 11.** Treatment categories analysis. **Appendix 12.** WinBUGS codes. **Appendix 13.** Difference of restricted mean survival times (RMST) at 18 months and the 1-year overall survival for trials comparing immunotherapy to docetaxel. (PDF 2899 kb)

Additional file 2: Figure S1. Forest plots for four outcomes. **Figure S2.** Cumulative ranking curves and SUCRA values for four outcomes. **Figure S3.** Rankograms for four outcomes. **Figure S4.** Evaluation of inconsistency. **Figure S5.** Subgroup analyses by histology and ethnicity: network graphs of trials for OS, forest plots, and SUCRA values for OS and PFS. **Figure S6.** Funnel plots of small-study effects. **Figure S7.** Overall assessment of bias. (PDF 1930 kb)
