# Supplementary material for: Comparative efficacy and safety of second-line treatments for advanced non-small cell lung cancer with wild-type or unknown status for epidermal growth factor receptor: a systematic review and network meta-analysis
Source: BMC Med. 2017 Oct 30;15:193. doi: 10.1186/s12916-017-0954-x (PMC5662096; doi:10.1186/s12916-017-0954-x)
Supplement: Supplementary file 1 — Definition used for objective response and serious adverse events. Appendix 2. Full search strategy. Appendix 3. Data extraction process. Appendix 4. Risk of bias assessment. Appendix 5. Classification of second-line treatments. Appendix 6. Reasons for excluding full texts and conference abstracts. Appendix 7. Identified reports for the eligible trials. Appendix 8. Characteristics of the 98 individual trials. Appendix 9. Results of individual trials. Appendix 10. Results of pairwise meta-analyses and patient characteristics across trials within each comparison. Appendix 11. Treatment categories analysis. Appendix 12. WinBUGS codes. Appendix 13. Difference of restricted mean survival times (RMST) at 18 months and the 1-year overall survival for trials comparing immunotherapy to docetaxel. (PDF 2899 kb) [file 12916_2017_954_MOESM1_ESM.pdf]

Supplementary appendix for

"Comparative efficacy and safety of second-line treatments for advanced non–small-cell lung cancer with wild-type or unknown status for epidermal growth factor receptor: a systematic review and network meta-analyses"

Appendix 1: Definition used for objective response and serious adverse events.......................... 2

Appendix 2: Full search strategy 3

Appendix 3: Data extraction process 8

Appendix 4: Risk of bias assessment... 10

Appendix 5: Classification of second-line treatments 19

Appendix 6: Reasons for excluding full texts and conference abstracts 20

Appendix 7: Identified reports for the eligible trials 22

Appendix 8: Characteristics of the 98 individual trials 43

Appendix 9: Results of individual trials.. 50

Appendix 10: Results of pairwise meta-analyses and patient characteristics across trials within each comparison 60

Appendix 11: Treatment categories analysis...63

Appendix 12: WinBUGS codes...67

Appendix 13: Difference of restricted mean survival times (RMST) at 18 months and the 1-year overall survival for trials comparing immunotherapy to docetaxel...............................70

Appendix 1: Definition used for objective response and serious adverse events

- *Objective response* (OR) corresponds to a complete response or a partial response, according to the Response Evaluation Criteria in Solid Tumors (RECIST) (Therasse. J Natl Cancer Inst 2000, 92(3):205-216). This evaluation may have been independently assessed (by a central radiology review) or investigator-assessed. It was assessed in the intent-to-treat population (i.e. the entire randomized population).
- toxicity evaluated by *serious adverse events* (SAE) as defined on clinicaltrials.gov (https://clinicaltrials.gov/ct2/help/glossary/serious-adverse-event):

  - "An adverse event that results in death, is life-threatening, requires inpatient hospitalization or extends a current hospital stay, results in an ongoing or significant incapacity or interferes substantially with normal life functions, or causes a congenital anomaly or birth defect. Medical events that do not result in death, are not life-threatening, or do not require hospitalization may be considered serious adverse events if they put the participant in danger or require medical or surgical intervention to prevent one of the results listed above "-.

  - In the safety assessment, we considered the number of serious adverse events compared to the number of patients evaluated for safety. This was not an intent-to-treat assessment.

  - When results were available in clinicaltrials.gov, we extracted the number of SAEs.

Appendix 2: Full search strategy

1. Searching in MEDLINE, CENTRAL and EMPBASE

All databases were searched using both controlled vocabulary (namely MeSH in MEDLINE and EMTREE in EMBASE) and a wide range of free-text terms (Higgins 2011)

Search code for MEDLINE (accessed via PubMed) and CENTRAL

Patients		
Lung cancer	#1	lung neoplasms[MeSH] OR carcinoma, non small cell lung[MeSH] OR lung carcinom*[tiab] OR lung neoplasm*[tiab] OR lung cancer[tiab] OR NSCLC[tiab] OR non small cell lung[tiab]
Advanced	#2	"Neoplasm Metastasis"[MeSH] OR advanced[tiab] OR stage IV[tiab] OR stage 4[tiab] OR stage four[tiab] OR metastatic[tiab] OR metastases[tiab]
Intervention		
Chemotherapy	#3	chemotherapy[tiab] OR chemotherapies[tiab] OR docetaxel[tiab] OR taxotere[tiab] OR "docetaxel"[Supplementary Concept] OR pemetrexed[tiab] OR alimta[tiab] OR "pemetrexed"[Supplementary Concept] OR gemcitabine[tiab] OR "gemcitabine"[Supplementary Concept] OR vinorelbine[tiab] OR "vinorelbine"[Supplementary Concept] OR paclitaxel[tiab] OR "Paclitaxel"[MeSH] OR "Vinblastine"[Supplementary Concept] OR vinblastine[tiab] OR "Ifosfamide"[MeSH] OR Ifosfamide[tiab] OR "irinotecan"[Supplementary Concept] OR irinotecan[tiab] OR "Mitomycin"[MeSH] OR mitomycin[tiab] OR vindesine[tiab] OR "Vindesine"[MeSH]
EGFR Targeted therapy	#4	"protein kinase inhibitors"[MeSH] OR "kinase inhibitor"[tiab] OR "kinase inhibitors"[tiab] OR gefitinib[tiab] OR iressa[tiab] OR "gefitinib"[Supplementary Concept] OR erlotinib[tiab] OR tarceva[tiab] OR "erlotinib"[Supplementary Concept] OR "icotinib"[Supplementary Concept] OR "icotinib"[tiab] OR afatinib[tiab] OR "BIBW 2992" [Supplementary Concept] OR "cetuximab"[Supplementary Concept] OR cetuximab[tiab]
VEGF targeted therapy	#5	bevacizumab[tiab] OR "bevacizumab"[Supplementary Concept] OR "vandetanib"[tiab] OR sunitinib[tiab] OR "sunitinib"[Supplementary Concept] OR sorafenib[tiab] OR "sorafenib"[Supplementary Concept] OR Ramucirumab[tiab] OR "ramucirumab"[Supplementary Concept] OR "nintedanib"[Supplementary Concept] OR nintedanib[tiab]
Second line	#6	second line[tiab] OR pretreat*[tiab] OR previously treated[tiab] OR refractory[tiab] OR recurrent[tiab]
Cochrane Highly Sensitive Search Strategy	#7	(randomized controlled trial[Publication Type] OR controlled clinical trial[Publication Type] OR randomized[tiab] OR placebo[tiab] OR drug therapy[sh] OR randomly[tiab] OR trial[tiab] OR groups[tiab]) NOT (animals[mh] NOT humans[mh])
Search algorithm	#8	#1 AND #2 AND (#3 OR #4 OR #5) AND #6 AND #7

Search code for EMBASE

Patients		
Lung cancer	#1	'lung tumor'/exp OR 'non small cell lung cancer'/exp OR (lung NEXT/1 carcinom*):ab,ti OR (lung NEXT/1 neoplasm*):ab,ti OR 'lung cancer':ab,ti OR nsclc:ab,ti OR 'non small cell lung':ab,ti
Advanced	#2	'metastasis'/exp OR advanced:ab,ti OR 'stage IV':ab,ti OR 'stage 4':ab,ti OR 'stage four':ab,ti OR metastatic:ab,ti OR metastases:ab,ti
Intervention		
Chemotherapy	#3	'chemotherapy':ab,ti OR 'chemotherapies':ab,ti OR docetaxel:ab,ti OR taxotere:ab,ti OR 'docetaxel'/exp OR pemetrexed:ab,ti OR alimta:ab,ti OR 'pemetrexed'/exp OR gemcitabine:ab,ti OR 'gemcitabine'/exp OR vinorelbine:ab,ti OR 'navelbine'/exp OR paclitaxel:ab,ti OR 'paclitaxel'/exp OR 'vinblastine'/exp OR vinblastine:ab,ti OR 'ifosfamide'/exp OR ifosfamide:ab,ti OR 'irinotecan'/exp OR irinotecan:ab,ti OR 'mitomycin'/exp OR mitomycin:ab,ti OR vindesine:ab,ti OR 'vindesine'/exp
EGFR Targeted therapy	#4	'protein kinase inhibitor'/exp OR 'kinase inhibitors':ab,ti OR 'kinase inhibitor':ab,ti OR gefitinib:ab,ti OR iressa:ab,ti OR 'gefitinib'/exp OR erlotinib:ab,ti OR tarceva:ab,ti OR 'erlotinib'/exp OR 'icotinib'/exp OR icotinib:ab,ti OR afatinib:ab,ti OR 'afatinib'/exp OR 'bibw 2992':ab,ti OR 'cetuximab'/exp OR cetuximab:ab,ti
VEGF targeted therapy	#5	bevacizumab:ab,ti OR 'bevacizumab'/exp OR vandetanib:ab,ti OR 'vandetanib'/exp OR sunitinib:ab,ti OR 'sunitinib'/exp OR sorafenib:ab,ti OR 'sorafenib'/exp OR ramucirumab:ab,ti OR 'ramucirumab'/exp OR 'nintedanib'/exp OR nintedanib:ab,ti
Second line	#6	'second line':ab,ti OR pretreat*:ab,ti OR 'previously treated':ab,ti OR 'refractory':ab,ti OR 'recurrent':ab,ti
Filter used by the UK Cochrane Centre	#7	'crossover procedure'/exp OR 'double-blind procedure'/exp OR 'randomized controlled trial'/exp OR 'single-blind procedure'/exp OR random* OR factorial* OR crossover* OR cross NEXT/1 over* OR placebo* OR doubl* NEAR/1 blind* OR singl* NEAR/1 blind* OR assign* OR allocat* OR volunteer*
Search algorithm	#8	#1 AND #2 AND (#3 OR #4 OR #5) AND #6 AND #7

2. Searching other resources

2.1. Previous systematic reviews in the Cochrane Database of Systematic Reviews, the Database of Abstracts of Reviews of Effectiveness and the PROSPERO international prospective register of systematic reviews for completed or published systematic reviews

2.2. Reference lists of all selected trials

2.3. Conference abstracts

- ASCO (American Society of Clinical Oncology) Meeting from 2009 to May 2017:

- 2009: http://meeting.ascopubs.org/content/vol27/15_suppl and http://meeting.ascopubs.org/content/vol27/18_suppl

- 2010: http://meeting.ascopubs.org/content/vol28/15_suppl and http://meeting.ascopubs.org/content/vol28/18_suppl
- 2011: http://meeting.ascopubs.org/content/vol29/15_suppl and http://meeting.ascopubs.org/content/vol29/18_suppl
- 2012 to 2016: http://meeting.ascopubs.org/ Section: Lung Cancer - Non-small Cell Metastatic

■ ESMO (European Society of Medical Oncology) Congress from 2009 to May 2017:

- European Journal of Cancer Supplements, Volume 7, issue 2, September 2009
- Annals of Oncology 21 (Supplement 8): viii122–viii161, 2010.
  Section non-small cell lung cancer, metastatic
- European Journal of Cancer, Volume 47 Supplement 1, September 2011
- Annals of Oncology 23 (Supplement 9): ix400–ix446, 2012.
  Section non-small cell lung cancer, metastatic
- European Journal of Cancer, Volume 49 Supplement 2, September 2013.
  Section Lung Cancer – Metastatic
- Annals of Oncology 25 (Supplement 4): iv426–iv470, 2014.
  Section non-small cell lung cancer, metastatic
- European Journal of Cancer, Volume 51, Supplement 3, Pages S1-S810 (September 2015).
  Section Lung Cancer – Metastatic Disease
- 2016: https://academic.oup.com/annonc/issue/27/suppl_6 and http://www.poster-submission.com/esmo2016/visitors/search/sresult

■ WCLC (World Conference on Lung Cancer) from 2009 to May 2017

- 2009: Journal of Thoracic Oncology, volume 4, number 9, supplement 1, September 2009.
  Section: NSCLC - Advanced Disease
- 2011: http://abstracts.webges.com/wclc2011/myitinerary Section:
  NSCLC - Advanced Stage
- 2013: http://abstracts.webges.com/wclc2013/myitinerary Keyword:
  Non-small cell lung cancer. Section: Medical oncology
- 2015: http://library.iaslc.org/virtual-library-search?product_id=1
  Keyword: Treatment of Advanced Diseases – NSCLC
- 2016: http://library.iaslc.org/virtual-library-search?product_id=6&author=&category=Advanced+NSCLC

2.4. Non-industry trial registries and results databases:

- WHO ICTRP (World Health Organization International Clinical Trials Registry Platform): http://apps.who.int/trialsearch/AdvSearch.aspx

Title: "second line" OR pretreated OR "previously treated" OR refractory OR recurrent Condition: "non small cell lung cancer" OR NSCLC

- ClinicalTrials.gov: https://clinicaltrials.gov/

Search terms: ("second line" OR pretreated OR "previously treated" OR refractory OR recurrent) AND (randomized OR randomly OR random)
Condition: "non small cell lung cancer" OR NSCLC

- EU Clinical Trials Register: https://www.clinicaltrialsregister.eu/ctr-search/search;jsessionid=ibt6KBrHrA42yLVJt9u22qA8QXoJ_EoYVTQqjK9ARwiqaDccKsdA!347291559

Search terms: ("non small cell lung cancer" OR NSCLC) AND ("second line" OR pretreated OR "previously treated" OR refractory OR recurrent)

2.5. Industry trial registries and results databases

Pharmaceutical company	Drugs	Link
AstraZeneca	Fulvestrant, Gefitinib, Selumetinib, Vandetanib	http://www.astrazenecaclinicaltrials.com/
Axelar	AXL1717	http://www.axelar.se/rd/posters-publications/
Bayer	Sorafenib	http://pharma.bayer.com/en/research-and-development/clinical-trials/trial-finder/index.php
Boehringer Ingelheim	Afatinib, Nintedanib	http://trials.boehringer-ingelheim.com/trial_results/clinical_trials_overview/clinical_trial_result.c=i.i=10.html
Bristol-Myers Squibb	Nivolumab	http://www.bms.com/research/pipeline/Pages/default.aspx
Celgene	Nab-Paclitaxel	http://www.celgene.com/research-development/clinical-trials/celgene-sponsored-trials/
Daiichi-Sankyo	Nimotuzumab, Tivantinib	http://www.daiichisankyo.com/rd/pipeline/development_pipeline/in dex.html
GlaxoSmithKline	Pazopanib, Topotecan, Trametinib, Vinorelbine	http://www.gsk-clinicalstudyregister.com/
Lilly	Gemcitabine, Pemetrexed	http://lillytrials.com/results/ctr_toc.pdf<br>http://lillytrials.com/results/alimta.pdf?bcsi_scan_628cd39dca2568d_2=0&bcsi_scan_filename=alimta.pdf<br>http://lillytrials.com/results/Gemzar.pdf?bcsi_scan_628cd39dca2568 d2=0&bcsi_scan_filename=Gemzar.pdf
Merck	Cetuximab, Dalotuzumab, Matuzumab, Pembrolizumab	http://biopharma.merckgroup.com/en/research_development/pipeli ne/pipeline.html
Novartis	Everolimus	http://www.novctrd.com/ctrdWebApp/clinicaltrialrepository/public/login.jsp
Roche	Atezolizumab, Bevacizumab, R1507, Tarceva, Onartuzumab	http://www.roche-trials.com/searchFullText.action?drug=2
Sanofi	Aflibercept, Docetaxel	http://en.sanofi.com/rd/clinical_trials/our_commitments/clinical_study_results.aspx

2.6. Regulatory agency online databases

- US Food and Drug Administration
http://www.accessdata.fda.gov/scripts/cder/drugsatfda/
Label, Review, Summary review for: Afatinib, Aflibercept, Atezolizumab, Bortezomib, Docetaxel, Everolimus, Fulvestrant, Gefitinib, Gemcitabine, Nab-Paclitaxel, Nintedanib, Nivolumab, Paclitaxel, Pazopanib, Pembrolizumab, Pemetrexed, Ramucirumab, Sorafenib, Tamoxifen, Topotecan, Vinorelbine.

- European Medicines Agency
http://www.ema.europa.eu/ema/index.jsp?curl=pages%2Fmedicines%2Flanding%2Fepar_search.jsp&mid=WC0b01ac058001d125&searchTab=&alreadyLoaded=true&isNewQuery=true&status=Authorised&status=Withdrawn&status=Suspended&status=Refused&keyword=Enter+keywords&searchType=name&taxonomyPath=Diseases.Cancer.Neoplasms+by+Site.Thoracic+Neoplasms.Respiratory+Tract+Neoplasms.Lung+Neoplasms.Carcinoma%2C+Bronchogenic&treeNumber=&currentCategory=Carcinoma%2C+Non-Small-Cell+Lung&searchGenericType=generics
European public assessment reports for: Afatinib, Bevacizumab, Docetaxel, Erlotinib, Gefitinib, Nintedanib, Nivolumab, Pembrolizumab, Pemetrexed.

2.7. Health technology assessment agencies

NICE (National Institute for Health and Care Excellence):
https://www.nice.org.uk/guidance/conditions-and-diseases/cancer/lung-cancer

AHRQ (Agency for Healthcare Research and Quality):
http://www.guideline.gov/browse/by-topic-detail.aspx?id=12697&ct=1

IQWIG (Institute for Quality and Efficiency in Health Care): https://www.iqwig.de/en/projects-results/publications/iqwig-reports.1071.html

HAS (Haute Autorité de Santé):
http://www.has-sante.fr/portail/jcms/c_39085/en/recherche?portlet=c_39085&opSearch=&lang=en&portal=c_2566858&FACET_TYPE=opinions%2Fgenerated.AVISMedicament&FACET_THEME=c_1151682

3. Pilot test for study selection

We pilot-tested the selection process on a sample of 100 records (for titles and abstracts) and 10 reports (for full-text articles) to ensure that the selection criteria were applied consistently by the two authors.

Appendix 3: Data extraction process

- Dates of publication of the full-text article(s) (online publication, if any) and conference abstract(s), date of results posting on non-industry and industry trial registries, and date of publication of reports by regulatory agencies or HTA agencies
- Trial characteristics: study phase (II or III), single-center or multicenter status, funding source (private, public, both or unclear), number of randomized patients in each arm
- Intervention characteristics: dosage, frequency and modality of administration
- Patient characteristics: age, gender, stage (IIIB versus IV), histology (non squamous versus squamous), ethnicity (Asian versus Caucasian), performance status (PS 0-1 versus PS 2), history of smoking (never versus former or current smoker), EGFR mutation status (wild-type versus unknown status), proportion of patients with second-line treatment
- Outcome data: hazard ratios (HRs) for PFS and OS and their 95% confidence intervals (CIs), number of patients with an objective response, number of patients with serious adverse events and means and standard deviations for quality of life.

To obtain missing information from the eligible studies we contacted trial investigators using the following procedure:

For conference abstracts, the first and the last author were contacted by email. For trials identified as "completed", the main investigator or the pharmaceutical company were contacted by email. Two weeks later, a reminder was sent and the inclusion procedures were closed one month later.
When primary outcomes (hazard ratio for OS and/or PFS and their associated 95% confidence intervals) could not be extracted from the original article, corresponding authors of manuscript were contacted by email. A reminder was sent two weeks later and the data collection process was closed one month later.

Using this procedure, we managed to obtain missing outcome data for the following studies:

**Trials**	**Authors contacted**	**Request**	**Answer**	**Outcome included**
**Unpublished trials**				
Bhatnagar, 2012	Bhatnagar, Kumbhaj	Trial results	Study results provided	ORR, QoL
ULTIMATE, 2016	Cortot, Besse	HR for OS	No	HR for OS°
**Published trials**				
Chen, 2011	Chen	HR for OS	No	HR for OS°
Dai, 2013	Dai	HR for PFS and OS	No	HR for PFS°
Esteban, 2003	Esteban	HR for PFS and OS[£]	No	HR for OS°
GFPC 05-06, 2011	Vergnenegre, Chouaid	HR for PFS	HR for PFS provided	HR for PFS
Jones, 2008	Jones	HR for PFS and OS[£]	No	HR for OS°
Kim, 2015	Kim YS	HR for PFS and OS	No	HR for PFS and OS°
Levy, 2014	Levy	HR for PFS and OS	Propose to contact laboratory	HR for PFS and OS°
Li, 2010	Li H	HR for PFS and OS	No	HR for OS°
Maitland, 2014	Maitland	HR for PFS and OS	Individual patient data provided	HR for PFS and OS
TALISMAN, 2015	Gridelli	HR for PFS and OS	No	HR for PFS and OS°
Tax 317, 2000	Shepherd	HR for PFS and OS[£]	No	HR for OS°

[£]trials reporting time to progression data instead of PFS
°HR recalculated by reconstructing individual patient data from published Kaplan-Meier

Appendix 4: Risk of bias assessment

For each risk of bias item, we rated the studies being at "low risk of bias", "high risk of bias" or "unclear risk of bias" using the risk of bias tool of The Cochrane Collaboration. We considered separately objective and subjective outcomes. Specifically, we assessed the eligible trials as follows:

Selection bias

- Sequence generation

Randomization was considered at "low risk", if the allocation sequence was generated from a table of random numbers, by minimization or by computer. It was considered "unclear", if it was stated that the trial was randomized but the method was not described.

- Allocation sequence concealment

Allocation concealment was considered at "low risk", if the report states that it was undertaken by means of sequentially pre-numbered, sealed opaque envelopes or by a centralized system. It was considered "unclear", if the method of allocation concealment was not described.

Performance bias

- Blinding of patients and care providers

Objective (overall mortality) and subjective outcomes (progression free survival, response and toxicity) was considered separately. Blinding of patients and care providers was considered at "low risk" if blinding was insured or if the outcome was unlikely to be affected by lack of blinding (OS) and at "high risk" for subjective outcomes if blinding was lacking.

Detection bias

- Blinding of outcome assessors

Objective and subjective outcomes was considered separately. Blinding of outcome assessors was considered at "low risk" if blinding was insured or for an objective outcome; it was considered at "low risk" if an independent Clinical Endpoint Adjudication Committee assessed subjective outcomes and at "high risk" otherwise.

Attrition bias

- Incomplete outcome data

We examined if there was imbalance across intervention groups in numbers or reasons for missing data and if the analysis was carried out based on intention to treat.

Reporting bias

- Selective outcome reporting

We evaluated if each outcome was measured, analyzed and reported. Outcomes specified in protocols (if available on the clinicaltrials.gov) and in materials and methods was compared to outcomes presented in the results section.

Overall bias

Up to 2 “unclear” and no “high risk”, the overall bias was considered “low”.
3 or more “unclear” and up to one “high risk”, the overall bias was considered “moderate”; and otherwise it was considered “high”.

Our final risk of bias assessments for all eligible trials are presented in the table below:

Assessment of objective outcome (mortality)

Trials	Sequence generation	Allocation concealment	Blinding of patients and care providers	Blinding of outcomes assessors	Incomplete outcome data	Selective outcome reporting	Overall bias
ARCHER 1009, 2014	low risk	low risk	low risk	low risk	low risk	low risk	low risk
ARQ 197-209, 2011	unclear	low risk	low risk	low risk	low risk	low risk	low risk
ATTENTION, 2015	unclear	unclear	low risk	low risk	low risk	low risk	low risk
Bergqvist, 2014	low risk	low risk	low risk	low risk	low risk	low risk	low risk
Besse, 2014	unclear	unclear	low risk	low risk	low risk	high risk	moderate risk
BeTa, 2011	low risk	low risk	low risk	low risk	low risk	low risk	low risk
Bhatnagar, 2012	unclear	unclear	NA	NA	unclear	unclear	high risk
Blumenschein, 2015	unclear	unclear	low risk	low risk	low risk	low risk	low risk
BR21, 2005	low risk	low risk	low risk	low risk	low risk	unclear	low risk
CALGB 30704, 2014	low risk	unclear	low risk	low risk	low risk	low risk	low risk
CheckMate017, 2015	low risk	low risk	low risk	low risk	low risk	low risk	low risk
CheckMate057, 2015	low risk	low risk	low risk	low risk	low risk	low risk	low risk
Chen, 2011	unclear	unclear	low risk	low risk	low risk	low risk	low risk
CTONG0806, 2014	low risk	unclear	low risk	low risk	low risk	low risk	low risk
Dai, 2013	unclear	unclear	NA	NA	unclear	unclear	high risk

DATE, 2015	low risk	unclear	low risk	low risk	low risk	low risk	low risk
DELTA, 2014	low risk	unclear	low risk	low risk	low risk	unclear	low risk
Dittrich, 2014	low risk	low risk	low risk	low risk	low risk	low risk	low risk
E1512, 2015	low risk	low risk	low risk	low risk	high risk	low risk	moderate risk
Esteban, 2003	unclear	unclear	low risk	low risk	low risk	unclear	moderate risk
Gerber, 2014	unclear	unclear	low risk	low risk	unclear	unclear	high risk
GFPC 05-06, 2011	unclear	unclear	low risk	low risk	high risk	unclear	moderate risk
Groen, 2013	low risk	low risk	low risk	low risk	low risk	low risk	low risk
Hainsworth, 2010	unclear	unclear	NA	NA	low risk	low risk	high risk
Han, 2011	unclear	unclear	NA	NA	unclear	unclear	high risk
HANSHIN, 2015	unclear	unclear	low risk	low risk	low risk	unclear	moderate risk
Herbst, 2007	unclear	unclear	low risk	low risk	low risk	unclear	moderate risk
Heymach, 2007	unclear	unclear	low risk	low risk	low risk	low risk	low risk
HORG, 2013	low risk	unclear	low risk	low risk	low risk	high risk	moderate risk
Hosomi, 2015	low risk	low risk	low risk	low risk	high risk	low risk	moderate risk
ICOGEN, 2013	low risk	low risk	low risk	low risk	low risk	low risk	low risk
INTEREST, 2008	low risk	low risk	low risk	low risk	low risk	low risk	low risk
ISEL, 2005	low risk	low risk	low risk	low risk	low risk	unclear	low risk
ISTANA, 2010	unclear	unclear	low risk	low risk	low risk	low risk	low risk
Janne, 2013	low risk	low risk	low risk	low risk	low risk	low risk	low risk
JMEI, 2004	unclear	unclear	low risk	low risk	low risk	unclear	moderate risk
Jones, 2008	low risk	unclear	low risk	low risk	low risk	low risk	low risk
Juan, 2014	unclear	unclear	low risk	low risk	low risk	low risk	low risk
Kapoor, 2015	unclear	unclear	low risk	low risk	unclear	unclear	high risk
Katakami, 2014	unclear	unclear	low risk	low risk	unclear	low risk	moderate risk
KCSG-LU08-01, 2012	low risk	unclear	low risk	low risk	low risk	low risk	low risk
Kelly, 2012	unclear	unclear	low risk	low risk	low risk	low risk	low risk
KEYNOTE-010,2015	low risk	low risk	low risk	low risk	low risk	low risk	low risk
Kim A, 2012	low risk	low risk	low risk	low risk	low risk	low risk	low risk
Kim B, 2012	low risk	low risk	low risk	low risk	low risk	low risk	low risk
Kim, 2015	unclear	unclear	low risk	low risk	unclear	high risk	moderate risk
Krzakowski, 2010	unclear	unclear	low risk	low risk	low risk	unclear	moderate risk

Kuo, 2013	unclear	unclear	NA	NA	unclear	unclear	high risk
Lee, 2013	low risk	low risk	low risk	low risk	low risk	low risk	low risk
Levy, 2014	unclear	unclear	low risk	low risk	low risk	low risk	low risk
Li, 2010	unclear	unclear	low risk	low risk	unclear	unclear	high risk
Li, 2012	unclear	unclear	NA	NA	high risk	unclear	high risk
Li, 2013	low risk	unclear	low risk	low risk	high risk	low risk	moderate risk
Li, 2014	low risk	unclear	low risk	low risk	low risk	low risk	low risk
Liu, 2015	unclear	unclear	low risk	low risk	low risk	unclear	moderate risk
LUME-COLUMBUS,2016	unclear	unclear	NA	NA	high risk	high risk	high risk
LUME-LUNG 1, 2014	low risk	low risk	low risk	low risk	low risk	low risk	low risk
LUME-LUNG 2, 2013	low risk	low risk	low risk	low risk	low risk	low risk	low risk
Lux-Lung 8, 2015	low risk	low risk	low risk	low risk	low risk	low risk	low risk
Maitland, 2014	unclear	unclear	low risk	low risk	high risk	low risk	moderate risk
Marangolo, 2000	unclear	unclear	NA	NA	unclear	unclear	high risk
MARQUEE, 2013	unclear	unclear	low risk	low risk	low risk	low risk	low risk
MARVEL, 2013	unclear	unclear	NA	NA	unclear	unclear	high risk
METLung, 2014	unclear	unclear	low risk	low risk	unclear	low risk	moderate risk
Moran, 2014	unclear	unclear	low risk	low risk	low risk	low risk	low risk
Natale, 2009	unclear	unclear	low risk	low risk	low risk	high risk	moderate risk
NCCTG N0626, 2011	unclear	unclear	NA	NA	high risk	unclear	high risk
NCIC CTG BR.26, 2014	low risk	low risk	low risk	low risk	low risk	low risk	low risk
NVALT-10, 2013	unclear	unclear	low risk	low risk	low risk	low risk	low risk
OAK,2016	low risk	low risk	low risk	low risk	low risk	low risk	low risk
Paz-Ares, 2008	unclear	unclear	low risk	low risk	low risk	unclear	moderate risk
POPLAR, 2016	low risk	low risk	low risk	low risk	low risk	low risk	low risk
PROSE, 2014	low risk	low risk	low risk	low risk	high risk	low risk	moderate risk
Ramalingam, 2011	unclear	unclear	low risk	low risk	low risk	low risk	low risk
Ramalingam, 2012	unclear	unclear	low risk	low risk	low risk	low risk	low risk
Ramlau, 2006	unclear	unclear	low risk	low risk	low risk	unclear	moderate risk
Ramlau, 2012	low risk	low risk	low risk	low risk	low risk	low risk	low risk
REVEL, 2014	low risk	low risk	low risk	low risk	low risk	low risk	low risk
Sbar, 2010	unclear	unclear	NA	NA	unclear	unclear	high risk

Scagliotti, 2012	low risk	low risk	low risk	low risk	low risk	low risk	low risk
Scagliotti, 2014	unclear	unclear	low risk	low risk	low risk	low risk	low risk
Schiller, 2010	low risk	unclear	low risk	low risk	low risk	low risk	low risk
SELECT 1,2017	low risk	low risk	low risk	low risk	low risk	low risk	low risk
SIGN, 2006	unclear	low risk	low risk	low risk	low risk	unclear	low risk
Spigel, 2011	low risk	unclear	low risk	low risk	low risk	low risk	low risk
Spigel, 2012	unclear	unclear	low risk	low risk	unclear	low risk	moderate risk
Spigel, 2013	low risk	low risk	low risk	low risk	low risk	low risk	low risk
Sun, 2013	unclear	unclear	low risk	low risk	low risk	low risk	low risk
TAILOR, 2013	low risk	low risk	low risk	low risk	low risk	low risk	low risk
TALISMAN, 2015	low risk	low risk	low risk	low risk	high risk	low risk	moderate risk
TAX 317, 2000	unclear	unclear	low risk	low risk	low risk	unclear	moderate risk
TITAN, 2012	low risk	low risk	low risk	low risk	low risk	low risk	low risk
TORI L-03, 2013	unclear	unclear	low risk	low risk	unclear	low risk	moderate risk
ULTIMATE,2016	unclear	unclear	low risk	low risk	unclear	unclear	high risk
V15-32, 2008	unclear	unclear	low risk	low risk	low risk	low risk	low risk
Wen, 2016	unclear	unclear	low risk	low risk	low risk	unclear	moderate risk
Witta, 2012	low risk	unclear	low risk	low risk	low risk	unclear	low risk
WJOG 5108L, 2016	unclear	low risk	low risk	low risk	low risk	unclear	low risk
ZEAL, 2011	low risk	unclear	low risk	low risk	low risk	low risk	low risk
ZEST, 2011	unclear	unclear	low risk	low risk	low risk	low risk	low risk
Zhang, 2015	unclear	unclear	low risk	low risk	low risk	unclear	moderate risk
ZODIAC, 2010	low risk	low risk	low risk	low risk	low risk	low risk	low risk

Assessment of subjective outcomes (progression free survival, objective response rate, serious adverse events)

Trials	Sequence generation	Allocation concealment	Blinding of patients and care providers	Blinding of outcomes assessors	Incomplete outcome data	Selective outcome reporting	Overall bias
ARCHER 1009, 2014	low risk	low risk	low risk	low risk	low risk	low risk	low risk
ARQ 197-209, 2011	unclear	low risk	low risk	low risk	low risk	low risk	low risk
ATTENTION, 2015	unclear	unclear	low risk	low risk	low risk	low risk	low risk
Bergqvist, 2014	low risk	low risk	high risk	low risk	low risk	low risk	moderate risk
Besse, 2014	unclear	unclear	high risk	high risk	low risk	high risk	high risk
BeTa, 2011	low risk	low risk	low risk	low risk	low risk	low risk	low risk
Bhatnagar, 2012	unclear	unclear	unclear	unclear	unclear	unclear	high risk
Blumenschein, 2015	unclear	unclear	high risk	high risk	low risk	low risk	high risk
BR21, 2005	low risk	low risk	low risk	unclear	low risk	unclear	low risk
CALGB 30704, 2014	low risk	unclear	high risk	high risk	low risk	low risk	high risk
CheckMate017, 2015	low risk	low risk	high risk	high risk	low risk	low risk	high risk
CheckMate057, 2015	low risk	low risk	high risk	high risk	low risk	low risk	high risk
Chen, 2011	unclear	unclear	high risk	high risk	low risk	low risk	high risk
CTONG0806, 2014	low risk	unclear	high risk	low risk	low risk	low risk	moderate risk
Dai, 2013	unclear	unclear	high risk	high risk	unclear	unclear	high risk
DATE, 2015	low risk	unclear	high risk	high risk	low risk	low risk	high risk
DELTA, 2014	low risk	unclear	high risk	high risk	low risk	unclear	high risk
Dittrich, 2014	low risk	low risk	high risk	high risk	low risk	low risk	high risk
E1512, 2015	low risk	low risk	high risk	high risk	high risk	low risk	high risk
Esteban, 2003	unclear	unclear	high risk	high risk	low risk	unclear	high risk
Gerber, 2014	unclear	unclear	high risk	low risk	unclear	unclear	high risk
GFPC 05-06, 2011	unclear	unclear	high risk	low risk	high risk	unclear	high risk
Groen, 2013	low risk	low risk	low risk	low risk	low risk	low risk	low risk
Hainsworth, 2010	unclear	unclear	high risk	high risk	low risk	low risk	high risk

Han, 2011	unclear	unclear	high risk	high risk	unclear	unclear	high risk
HANSHIN, 2015	unclear	unclear	high risk	low risk	low risk	unclear	moderate risk
Herbst, 2007	unclear	unclear	unclear	high risk	low risk	unclear	high risk
Heymach, 2007	unclear	unclear	low risk	low risk	low risk	low risk	low risk
HORG, 2013	low risk	unclear	high risk	low risk	low risk	high risk	high risk
Hosomi, 2015	low risk	low risk	low risk	low risk	high risk	low risk	moderate risk
ICOGEN, 2013	low risk	low risk	low risk	low risk	low risk	low risk	low risk
INTEREST, 2008	low risk	low risk	high risk	high risk	low risk	low risk	high risk
ISEL, 2005	low risk	low risk	low risk	low risk	low risk	unclear	low risk
ISTANA, 2010	unclear	unclear	high risk	high risk	low risk	low risk	high risk
Janne, 2013	low risk	low risk	low risk	low risk	low risk	low risk	low risk
JMEI, 2004	unclear	unclear	high risk	high risk	low risk	unclear	high risk
Jones, 2008	low risk	unclear	high risk	high risk	low risk	low risk	high risk
Juan, 2014	unclear	unclear	high risk	high risk	low risk	low risk	high risk
Kapoor, 2015	unclear	unclear	high risk	high risk	unclear	unclear	high risk
Katakami, 2014	unclear	unclear	high risk	unclear	unclear	low risk	high risk
KCSG-LU08-01, 2012	low risk	unclear	high risk	low risk	low risk	low risk	moderate risk
Kelly, 2012	unclear	unclear	high risk	high risk	low risk	low risk	high risk
KEYNOTE-010,2015	low risk	low risk	high risk	low risk	low risk	low risk	moderate risk
Kim A, 2012	low risk	low risk	high risk	low risk	low risk	low risk	moderate risk
Kim B, 2012	low risk	low risk	high risk	low risk	low risk	low risk	moderate risk
Kim, 2015	unclear	unclear	high risk	high risk	unclear	high risk	high risk
Krzakowski, 2010	unclear	unclear	high risk	high risk	low risk	unclear	high risk
Kuo, 2013	unclear	unclear	high risk	low risk	unclear	unclear	high risk
Lee, 2013	low risk	low risk	high risk	high risk	low risk	low risk	high risk
Levy, 2014	unclear	unclear	high risk	high risk	low risk	low risk	high risk
Li, 2010	unclear	unclear	high risk	high risk	unclear	unclear	high risk
Li, 2012	unclear	unclear	high risk	high risk	high risk	unclear	high risk
Li, 2013	low risk	unclear	high risk	high risk	high risk	low risk	high risk
Li, 2014	low risk	unclear	high risk	high risk	low risk	low risk	high risk
Liu, 2015	unclear	unclear	high risk	high risk	low risk	unclear	high risk
LUME-COLUMBUS,2016	unclear	unclear	low risk	low risk	high risk	high risk	high risk

LUME-LUNG 1, 2014	low risk	low risk	low risk	low risk	low risk	low risk	low risk
LUME-LUNG 2, 2013	low risk	low risk	low risk	low risk	low risk	low risk	low risk
Lux-Lung 8, 2015	low risk	low risk	high risk	low risk	low risk	low risk	moderate risk
Maitland, 2014	unclear	unclear	high risk	high risk	high risk	low risk	high risk
Marangolo, 2000	unclear	unclear	unclear	unclear	unclear	unclear	high risk
MARQUEE, 2013	unclear	unclear	low risk	low risk	low risk	low risk	low risk
MARVEL, 2013	unclear	unclear	high risk	high risk	unclear	unclear	high risk
METLung, 2014	unclear	unclear	low risk	low risk	unclear	low risk	moderate risk
Moran, 2014	unclear	unclear	high risk	high risk	low risk	low risk	high risk
Natale, 2009	unclear	unclear	unclear	unclear	low risk	high risk	high risk
NCCTG N0626, 2011	unclear	unclear	high risk	high risk	high risk	unclear	high risk
NCIC CTG BR.26, 2014	low risk	low risk	low risk	low risk	low risk	low risk	low risk
NVALT-10, 2013	unclear	unclear	high risk	high risk	low risk	low risk	high risk
OAK,2016	low risk	low risk	high risk	high risk	low risk	low risk	high risk
Paz-Ares, 2008	unclear	unclear	high risk	high risk	low risk	unclear	high risk
POPLAR, 2016	low risk	low risk	high risk	high risk	low risk	low risk	high risk
PROSE, 2014	low risk	low risk	high risk	high risk	high risk	low risk	high risk
Ramalingam, 2011	unclear	unclear	high risk	high risk	low risk	low risk	high risk
Ramalingam, 2012	unclear	unclear	high risk	high risk	low risk	low risk	high risk
Ramlau, 2006	unclear	unclear	high risk	high risk	low risk	unclear	high risk
Ramlau, 2012	low risk	low risk	low risk	low risk	low risk	low risk	low risk
REVEL, 2014	low risk	low risk	low risk	low risk	low risk	low risk	low risk
Sbar, 2010	unclear	unclear	low risk	low risk	unclear	unclear	high risk
Scagliotti, 2012	low risk	low risk	low risk	low risk	low risk	low risk	low risk
Scagliotti, 2014	unclear	unclear	high risk	high risk	low risk	low risk	high risk
Schiller, 2010	low risk	unclear	high risk	unclear	low risk	low risk	moderate risk
SELECT 1,2017	low risk	low risk	low risk	low risk	low risk	low risk	low risk
SIGN, 2006	unclear	low risk	high risk	high risk	low risk	unclear	high risk
Spigel, 2011	low risk	unclear	low risk	low risk	low risk	low risk	low risk
Spigel, 2012	unclear	unclear	low risk	low risk	unclear	low risk	moderate risk
Spigel, 2013	low risk	low risk	low risk	low risk	low risk	low risk	low risk
Sun, 2013	unclear	unclear	high risk	high risk	low risk	low risk	high risk

TAILOR, 2013	low risk	low risk	high risk	high risk	low risk	low risk	high risk
TALISMAN, 2015	low risk	low risk	high risk	high risk	high risk	low risk	high risk
TAX 317, 2000	unclear	unclear	high risk	high risk	low risk	unclear	high risk
TITAN, 2012	low risk	low risk	high risk	high risk	low risk	low risk	high risk
TORI L-03, 2013	unclear	unclear	high risk	high risk	unclear	low risk	high risk
ULTIMATE,2016	unclear	unclear	high risk	high risk	unclear	unclear	high risk
V15-32, 2008	unclear	unclear	high risk	low risk	low risk	low risk	moderate risk
Wen, 2016	unclear	unclear	high risk	high risk	low risk	unclear	high risk
Witta, 2012	low risk	unclear	low risk	low risk	low risk	unclear	low risk
WJOG 5108L, 2016	unclear	low risk	high risk	high risk	low risk	unclear	high risk
ZEAL, 2011	low risk	unclear	low risk	low risk	low risk	low risk	low risk
ZEST, 2011	unclear	unclear	low risk	low risk	low risk	low risk	low risk
Zhang, 2015	unclear	unclear	high risk	high risk	low risk	unclear	high risk
ZODIAC, 2010	low risk	low risk	low risk	low risk	low risk	low risk	low risk

Appendix 5: Classification of second-line treatments

- Cytotoxic monochemotherapy including Docetaxel, Pemetrexed. We did not consider trials assessing a combination of two cytotoxic drugs because:
  - In Di Maio meta-analysis of individual patient data which analyzed six trials (847 patients), doublet chemotherapy increased response rate and progression-free survival but was more toxic and did not improve overall survival compared to single-agent (1).
  - Mono-chemotherapy remains the standard treatment for second-line treatment by the ASCO and ESMO Clinical Practice Guidelines (2-3)
- Targeted therapy mainly targeting EGFR (TKI: e.g. Erlotinib, Gefitinib and monoclonal antibody: e.g. Cetuximab), VEGFR, c-met, IGF1R or multi-targeted tyrosine kinase inhibitors
- Immunotherapy: treatments targeting programmed death 1 (PD-1) and its ligands (PD-L1)
- Combination of a cytotoxic chemotherapy and a targeted therapy
- Combination of two targeted therapies
- Placebo or best supportive care

1. Di Maio M, l. Meta-analysis of single-agent chemotherapy compared with combination chemotherapy as second-line treatment of advanced non-small-cell lung cancer. J Clin Oncol 2009;27(11):1836–43.
2. Masters GA. Systemic Therapy for Stage IV Non-Small-Cell Lung Cancer: American Society of Clinical Oncology Clinical Practice Guideline Update. J Clin Oncol 2015;33(30):3488–515.
3. Novello S. Metastatic non-small-cell lung cancer: ESMO Clinical Practice Guidelines for diagnosis, treatment and follow-up. Ann Oncol 2016;27(suppl 5):v1–27.

Appendix 6: Reasons for excluding full texts and conference abstracts

Study	Reference	Reasons for exclusion
Chang 1993	Chang AY, Kim K, Glick J, Anderson T, Karp D, Johnson D. Phase II study of taxol, merbarone, and piroxantrone in stage IV non- small-cell lung cancer: The Eastern Cooperative Oncology Group Results. *Journal of the National Cancer Institute* 1993;85(5):388 - 94	First line
Kabbinavar 2010	Kabbinavar FF, Miller VA, Johnson BE, O'Connor PG, Soh C. Overall survival (OS) in ATLAS, a phase IIIb trial comparing bevacizumab (B) therapy with or without erlotinib (E) after completion of chemotherapy (chemo) with B for first-line treatment of locally advanced, recurrent, or metastatic non-small cell lung cancer (NSCLC). *Journal of Clinical Oncology* 2010;28(15)	Maintenance
Gian 2012	Gian V, Rubin MS, Shipley D, Burris HA, Kaplan J, Kosloff RA, et al. Sorafenib and continued erlotinib or sorafenib alone in patients with advanced non-small cell lung cancer progressing on erlotinib: A randomized phase II study of the Sarah Cannon Research Institute (SCRI). *Journal of Clinical Oncology* 2012; 30(15)	Trial assessing continuation of treatment beyond progression
Lee 2012	Lee JS, Hirsh V, Park K, Qin S, Blajman CR, Perng RP, et al. Vandetanib Versus placebo in patients with advanced non-small-cell lung cancer after prior therapy with an epidermal growth factor receptor tyrosine kinase inhibitor: a randomized, double-blind phase III trial (ZEPHYR). *J Clin Oncol* 2012;30(10):1114-21	Third line and more
Mok 2012	Mok TSK, Wu YL, Zhou C, Sun Y, Zhang L, Liao M, et al. Subset analyses of east asian patients enrolled in the mission phase III, randomized, double-blind, placebo-controlled trial of sorafenib monotherapy in patients with advanced relapsed/refractory nsclc of predominantly non-squamous histology who failed 2-3 chemotherapy regimens. *Journal of Thoracic Oncology* 2012;7(11):S458	Third line and more
Takeda 2012	Takeda M, Okamoto I, Yamanaka T, Nakagawa K, Nakanishi Y. Impact of treatment with bevacizumab beyond disease progression: a randomized phase II study of docetaxel with or without bevacizumab after platinum-based chemotherapy plus bevacizumab in patients with advanced nonsquamous non-small cell lung cancer (WJOG 5910L). *BMC Cancer* 2012;12	Trial assessing continuation of treatment beyond progression
Hirsh 2013	Hirsh V, Cadranel J, Cong J, Fairclough D, Finnern H, Lorence R, et al. Symptom and healthrelated quality of life benefit of afatinib (BIBW 2992) in advanced NSCLC patients previously treated with erlotinib or gefitinib: Results of a randomized phase III trial (luxlung 1). *Journal of Thoracic Oncology* 2011;6(6):S324 - S25<br>Hirsh V, Cadranel J, Cong XJ, Fairclough D, Finnern HW, Lorence RM, et al. Symptom and quality of life benefit of afatinib in advanced non-small-cell lung cancer patients previously treated with erlotinib or gefitinib: Results of a randomized phase iib/iii trial (lux-lung 1). *Journal of Thoracic Oncology* 2013;8(2):229 - 37	Third line and more
Schuler 2014	Schuler MH, Yang CH, Park K, Bennouna J, Chen YM, Chouaid C, et al. Continuation of afatinib beyond progression: Results of a randomized, open-label, phase III trial of afatanib plus paclitaxel (P) versus investigator's choice chemotherapy (CT) in patients (pts) with metastatic non-small cell lung cancer (NSCLC) progressed on erlotinib/gefitinib (E/G) and afatanib-LUX-Lung 5 (LL5). *Journal of Clinical Oncology* 2014;32(15)	Third line
Berghmans 2007	Berghmans T, Lafitte JJ, Lecomte J, Alexopoulos CG, Van Cutsem O, Giner V, et al. Second-line paclitaxel in non-small cell lung cancer initially treated with cisplatin: A study by the European Lung Cancer Working Party. *British Journal of Cancer* 2007;96(11):1644 - 49	Not randomized on second line

Cheng 2010	Cheng B, Lou GY, Wang Z, Qi YJ, Weng L, Zhang YP. Combination of short-term efficacy of two second-line regimins in patients with advanced non-small-cell lung cancer. *Chinese Pharmaceutical Journal* 2010;45(23):1856 - 58	Not randomized on second line
Hellerstedt 2012	Hellerstedt BA, Edelman G, Vogelzang NJ, Kluger HM, Yasenchak CA, Shen X, et al. Activity of cabozantinib (XL184) in metastatic NSCLC: Results from a phase II randomized discontinuation trial (RDT). *Journal of Clinical Oncology* 2012;30(15)	Randomized discontinuation trial
Fiala 2013	Fiala O, Pesek M, Finek J, Krejci J, Bortlicek Z, Benesova L, et al. Second-line treatment of advanced NSCLC: Comparison of efficacy of erlotinib and chemotherapy. *Neoplasma* 2013;60(2):129-34	Not randomized
Blumenschein 2012	Blumenschein GR, Ciuleanu T, Robert F, Groen HJM, Usari T, Ruiz-Garcia A, et al. Sunitinib plus erlotinib for the treatment of advanced/metastatic non-small-cell lung cancer: A lead-in study. *Journal of Thoracic Oncology* 2012;7(9):1406 - 16	Lead-in study (dose-limiting toxicities and pharmacokinetics)
Hernandez 2014	Hernandez M, Santiesteban ER, Ortiz RA, Neninger E, Acosta S, Flores Y, et al. RANIDO: A phase III clinical trial of racotumomab-alum or nimotuzumab versus docetaxel in advanced non-small cell lung cancer patients. *Journal of Clinical Oncology* 2014;32(15)	Trial protocol
Auliac 2014	Auliac JB, Chouaid C, Greiller L, Monnet I, Le Caer H, Falchero L, et al. Randomized open-label non-comparative multicenter phase II trial of sequential erlotinib and docetaxel versus docetaxel alone in patients with non-small-cell lung cancer after failure of first-line chemotherapy: GFPC 10.02 study. Lung Cancer. 2014; 85(3):415-9	Randomized non-comparative study
Lynch 2009	Lynch TJ, Fenton D, Hirsh V, Bodkin D, Middleman EL, Chiappori A, et al. A randomized phase 2 study of erlotinib alone and in combination with bortezomib in previously treated advanced non-small cell lung cancer. Journal of Thoracic Oncology. 2009; 4(8):1002–9.	Randomized non-comparative study
Kim 2012	Kim ST, Uhm JE, Lee J, Sun JM, Sohn I, Kim SW, et al. Randomized phase II study of gefitinib versus erlotinib in patients with advanced non-small cell lung cancer who failed previous chemotherapy. *Lung Cancer*. 2012;75(1):82 - 8.<br>Ahn J, Kim S, Ahn M, Lee J, Uhm J, Sun J, et al. Randomized phase II study of gefitinib versus erlotinib in patients with advanced non-small cell lung cancer who failed previous chemotherapy. *Journal of Clinical Oncology*. 2010;28(15): 7551.	Enriched population on EGFR mutation

Appendix 7: Identified reports for the eligible trials

Out of the 102 included studies, the results of 87 trials were published.
Among these 87 trials, 65 had results reported in other sources:

- 18 in conference abstracts
- 9 in non-industry trial registries and results databases or regulatory agency online databases
- 21 in both
- 4 in another published article
- 1 in industry trial registries and results databases
- 3 had results reported in 3 different sources
- 9 had results reported in at least 4 different sources

The remaining 15 trials had unpublished results:

- 9 conference abstracts
- 2 in non-industry trial registry and results database (CT.gov)
- 2 in non-industry trial registry and results database (CT.gov) and industry trial registry and results database
- 2 in conference abstracts and non-industry trial registry and results database (CT.gov)

List of all available references for each included trial (311 references in total):

ARCHER 1009 2014

- Ramalingam SS. ARCHER 1009: A Study of Dacomitinib (PF-00299804) Vs. Erlotinib in The Treatment of Advanced Non-Small Cell Lung Cancer (ARCHER 1009). clinicaltrialsgov. April 2017: NCT01360554
- Ramalingam SS, Janne PA, Mok T, O'Byrne K, Boyer MJ, Von Pawel J, et al. Dacomitinib versus erlotinib in patients with advanced-stage, previously treated non-small-cell lung cancer (ARCHER 1009): A randomised, double-blind, phase 3 trial. The Lancet Oncology. 2014;15(12):1369 - 78.
- Ramalingam SS, Janne PA, Mok T, O'Byrne K, Boyer MJ, Zhang H, et al. Randomized, double-blinded study of dacomitinib, an irreversible pan-human epidermal growth factor receptor (HER) inhibitor, versus erlotinib for second-line/third-line therapy of locally advanced/metastatic non-small cell lung cancer (ARCHER 1009). Journal of Clinical Oncology. 2014;32(15):8018.
- Mok T, O'Byrne K, Ramalingam S, Janne P, Boyer M, Ahn J, et al. Dacomitinib versus Erlotinib in second/third line NSCLC: outcome for asian patients from the ARCHER 1009 Global Phase 3 trial. Annals of Oncology. 2014;25(4): iv426–iv70.

ARQ 197-209 2011

- Sequist LV, Von Pawel J, Garmey EG, Akerley WL, Brugger W, Ferrari D, et al. Randomized phase II study of erlotinib plus tivantinib versus erlotinib plus placebo in previously treated non-small-cell lung cancer. Journal of Clinical Oncology. 2011;29(24):3307-15.
- Von Pawel J, Akerley W, Brugger W, Ferrari D, Garmey E, Gerbe D, et al. Final results from Arq 197- 209: A global randomized placebo controlled phase 2 clinical trial of erlotinib plus ARQ 197 versus erlotinib plus placebo in previously treated EGFR inhibitor-naive patients with advanced non-small cell lung cancer (NSCLC). Journal of Thoracic Oncology. 2010;5(12): S501.
- Schiller J, Akerley W, Brugger W, Ferrari D, Garmey E, Gerber D, et al. Results from ARQ 197-209: A global randomized placebo-controlled phase II clinical trial of erlotinib plus ARQ 197 versus erlotinib plus placebo in previously treated EGFR inhibitor-naive patients with locally advanced or metastatic non-small cell lung cancer (NSCLC). Journal of Clinical Oncology. 2010;28(18): LBA7502.
- Sequist L, Akerley W, Brugger W, Ferrari D, Garmey E, Gerber D, et al. Final results from ARQ 197- 209: a global randomized placebo-controlled Phase II clinical trial of Erlotinib plus ARQ 197 versus Erlotinib plus Placebo in previously treated EGFR-inhibitor naïve patients with advanced non-small cell lung cancer. Annals of Oncology. 2010;21(8): viii122–viii61.

ATTENTION 2015

- Yoshioka H, Azuma K, Yamamoto N, Takahashi T, Nishio M, Katakami N, et al. A randomized, double- blind, placebo-controlled, phase III trial of erlotinib with or without a c-Met inhibitor tivantinib (ARQ
- 197) in Asian patients with previously treated stage IIIB/IV nonsquamous nonsmall-cell lung cancer harboring wild-type epidermal growth factor receptor (ATTENTION study). Ann Oncol. 2015;26(10):2066-72.

Bergqvist 2014

- Bergqvist M, Holgersson G, Bondarenko I, Grechanaya E, Maximovich A, Andor G, et al. Phase II randomized study of the IGF-1R pathway modulator AXL1717 compared to docetaxel in patients with previously treated, locally advanced or metastatic non-small cell lung cancer. Acta oncologica. 2017;56(3):441-7.
- Bergqvist M, Bondarenko I, Thuresson M, Klockare M, Harmenberg J. Randomized, controlled, multicenter, multinational phase 2 study of docetaxel (DCT) or AXL1717 treatment in patients with squamous cell carcinoma (SCC) or adenocarcinoma (AC) of non-small cell lung cancer (NSCLC). Journal of Clinical Oncology. 2014;32(5s): abstr 8091.
- Axelar website: "Randomized, Controlled, Multicenter, Multinational Phase II Study of Docetaxel (DCT) or AXL1717 Treatment in Patients with Squamous Cell Carcinoma (SCC) or Adenocarcinoma (AC) of Non-Small Cell Lung Cancer (NSCLC)". http://www.axelar.se/rd/posters-publications/

Besse 2014

- Besse B, Leighl N, Bennouna J, Papadimitrakopoulou VA, Blais N, Traynor AM, et al. Phase II study of everolimus-erlotinib in previously treated patients with advanced non-small-cell lung cancer. Annals of Oncology. 2014;25(2):409 - 15.
- Bennouna J, Besse B, Leighl NB, Blais N, Traynor AM, Papadimitrakopoulou V, et al. Everolimus plus erlotinib versus erlotinib alone in previously treated patients with advanced non-small-cell lung cancer (NSCLC). Annals of Oncology. 2010;21: viii140.

- Leighl NB, Soria J, Bennouna J, Blais N, Traynor AM, Papadimitrakopoulou V, et al. Phase II study of everolimus plus erlotinib in previously treated patients with advanced non-small cell lung cancer (NSCLC). Journal of Clinical Oncology. 2010;28(15): 7524.
- Novartis report 2014: https://www.novctrd.com/CtrdWeb/displaypdf.nov?trialresultid=5703

BeTa 2011

- Herbst R. A Phase III, Multicenter, Placebo-Controlled, Double-Blind, Randomized Clinical Trial to Evaluate the Efficacy of Bevacizumab in combination with Tarceva (Erlotinib) compared with Tarceva Alone for Treatment of Advanced Non-Small Cell Lung Cancer (NSCLC) After Failure of Standard First- Line Chemotherapy. Clinicaltrialsgov. November 2009: NCT00130728.
- Herbst RS, Ansari R, Bustin F, Flynn P, Hart L, Otterson GA, et al. Efficacy of bevacizumab plus erlotinib versus erlotinib alone in advanced non-small-cell lung cancer after failure of standard first-line chemotherapy (BeTa): A double-blind, placebo-controlled, phase 3 trial. The Lancet. 2011;377(9780):1846 - 54.

Bhatnagar 2012

- Bhatnagar AR, Singh DP, Sharma R, Kumbhaj P. Docetaxel versus geftinib in patients with locally advanced or metastatic NSCLC pretreated with platinum-based chemotherapy. Journal of Thoracic Oncology. 2012;7(9): S159.

Blumenschein 2015

- Blumenschein G. A Phase II, Open-label, Multicenter, Randomized Study to Assess the Efficacy and Safety of GSK1120212 Compared with Docetaxel in 2nd Line Subjects with Targeted Mutations (KRAS, NRAS, BRAF, MEK1) in Locally Advanced or Metastatic Non-small Cell Lung Cancer (NSCLC Stage IV). Clinicaltrialsgov. June 2013: NCT01362296.
- Blumenschein G, Smit EF, Planchard D, Kim DW, Cadranel J, De Pas T, et al. A randomized phase 2 study of the MEK1/MEK2 inhibitor trametinib (GSK1120212) compared with docetaxel in KRAS-mutant advanced non-small cell lung cancer (NSCLC). Annals of Oncology. 2015. pii: mdv072.
- Blumenschein GR, Smit EF, Planchard D, Kim DW, Cadranel J, De Pas T, et al. MEK114653: A randomized, multicenter, phase II study to assess efficacy and safety of trametinib (T) compared with docetaxel (D) in KRAS-mutant advanced non-small cell lung cancer (NSCLC). Journal of Clinical Oncology. 2013;31(15) : 8029.
- GSK report 2015: http://www.gsk-clinicalstudyregister.com/files2/373c6bed-07dd-4af1-a2bb-80019d5c705f

BR21 2005

- EMA 2005 Report: http://www.ema.europa.eu/docs/en_GB/document_library/EPAR_-
- _Scientific_Discussion/human/000618/WC500033991.pdf
- Shepherd FA, Rodrigues Pereira J, Ciuleanu T, Tan EH, Hirsh V, Thongprasert S, et al. Erlotinib in previously treated non-small-cell lung cancer. N Engl J Med. 2005;353(2):123-32.
- Zhu CQ, da Cunha Santos G, Ding K, Sakurada A, Cutz JC, Liu N, et al. Role of KRAS and EGFR as biomarkers of response to erlotinib in National Cancer Institute of Canada Clinical Trials Group Study BR.21. Journal of Clinical Oncology. 2008;26(26):4268-75.
- NICE report 16/12/2015: https://www.nice.org.uk/guidance/ta374

CALGB 30704 2014

- Heist R. A Randomized Phase II Study to Assess the Efficacy of Pemetrexed or Sunitinib (NSC # 736511) or Pemetrexed Plus Sunitinib in the Second-Line Treatment of Advanced Non-Small Cell Lung Cancer. Clinicaltrialsgov. February 2015: NCT00698815.
- Heist RS, Wang X, Hodgson L, Otterson GA, Stinchcombe TE, Gandhi L, et al. CALGB 30704 (Alliance): A randomized phase II study to assess the efficacy of pemetrexed or sunitinib or pemetrexed plus sunitinib in the second-line treatment of advanced non-small-cell lung cancer. Journal of Thoracic Oncology. 2014;9(2): 214 - 21.
- Heist RS, Wang XF, Hodgson L, Otterson GA, Stinchcombe T, Vokes EE, et al. CALGB 30704: A randomized phase II study to assess the efficacy of pemetrexed or sunitinib or pemetrexed plus sunitinib in the second-line treatment of advanced non-small cell lung cancer (NSCLC). Journal of Clinical Oncology. 2012; 30(15).

CheckMate 017 2015

- FDA 11/2015 report: http://www.accessdata.fda.gov/drugsatfda_docs/label/2015/125554s012lbl.pdf

- EPAR 14/01/2016 report: http://www.ema.europa.eu/docs/en_GB/document_library/EPAR_-_Product_Information/human/003840/WC500190648.pdf
- Brahmer J. Study of BMS-936558 (Nivolumab) Compared to Docetaxel in Previously Treated Advanced or Metastatic Squamous Cell Non-Small Cell Lung Cancer (NSCLC) (CheckMate 017). Clinicaltrialsgov. February 2016; NCT01642004.
- Brahmer J, Reckamp KL, Baas P, Crinò L, Eberhardt WEE, Poddubskaya E, et al. Nivolumab versus Docetaxel in Advanced Squamous-Cell Non-Small-Cell Lung Cancer. N Engl J Med. 2015; 373(2):123– 35.
- Borghaei H, Lynch TJ, Rizvi NA, Chow LQM, Reilly R, Crino L, et al. A phase III comparative study of nivolumab (anti-PD-1; BMS-936558; ONO-4538) versus docetaxel in patients with previously treated advanced or metastatic squamous cell non-small cell lung cancer (NSCLC). Journal of Clinical Oncology. 2013;31(15).
- Spigel DR, Reckamp KL, Rizvi NA, Poddubskaya E, West HJ, Eberhardt WEE, et al. A phase III study (CheckMate 017) of nivolumab (NIVO; anti-programmed death-1 [PD-1]) vs docetaxel (DOC) in previously treated advanced or metastatic squamous (SQ) cell non-small cell lung cancer (NSCLC). Journal of Clinical Oncology. 2015;33(15):8009.
- Reck M, Coon C, Taylor F, DeRosa M, Penrod J, Dastani H, et al. Evaluation of overall health status in patients with advanced squamous non-small cell lung cancer treated with nivolumab or docetaxel in CheckMate 017. European Journal of Cancer. September 2015;51(Supplement 3): S599.
- Gralla RJ, Reck M, Taylor F, Orsini L, Penrod JR, Coon C, et al. Evaluation of disease-related symptoms in patients with advanced squamous non-small cell lung cancer treated with nivolumab or docetaxel. Journal of Thoracic Oncology. 2015;10(9): S233-S4.
- Borghaei H, Brahmer JR, Horn L, Ready N, Steins M, Felip E, et al. Nivolumab (nivo) vs docetaxel (doc) in patients (pts) with advanced NSCLC: CheckMate 017/057 2-y update and exploratory cytokine profile analyses. Journal of Clinical Oncology. 2016;34(Supplement): abstract 9025.
- Reckamp K, Brahmer JR, Spigel DR, Rizvi NA, Poddubskaya E, West H, et al. Phase 3, randomized trial (checkmate 017) of nivolumab (NIVO) vs docetaxel in advanced squamous (SQ) cell non-small cell lung cancer (NSCLC). Journal of Thoracic Oncology 2015;10(9): S174-S5.
- Venkatachalam M, Stenehjem DD, Pietri G, Penrod JR, Korytowsky B. Healthcare Resource Utilization in Patients with Advanced NSCLC in CheckMate 017 and 057 Based on Treatment-Related Adverse Events. Annals of Oncology. 2016;27(Supplement 6):1220P.
- Barlesi F, Steins M, Horn L, Ready N, Felip E, Borghaei H, et al. Long-term outcomes with nivolumab (Nivo) vs docetaxel (Doc) in patients (Pts) with advanced (Adv) NSCLC: CheckMate 017 and CheckMate 057 2-y update. Annals of Oncology. 2016;27(Supplement 6): vi416–vi54.
- Reck M, Coon C, Taylor F, Derosa M, Penrod JR, Dastani H, et al. Evaluation of overall health status in patients with advanced squamous non-small cell lung cancer treated with nivolumab or docetaxel in CheckMate 017. Annals of Oncology. 2015;26(Supplement 9): ix125-ix47 (abstract 460P).
- BMS 01/2016: http://packageinserts.bms.com/pi/pi_opdivo.pdf

CheckMate 057 2015

- FDA 11/2015 report: http://www.accessdata.fda.gov/drugsatfda_docs/label/2015/125554s012lbl.pdf
- EMA report 2017: http://www.ema.europa.eu/docs/en_GB/document_library/EPAR_-_Product_Information/human/003985/WC500189765.pdf
- Borghaei H. Study of BMS-936558 (Nivolumab) Compared to Docetaxel in Previously Treated Metastatic Non-squamous NSCLC (CheckMate057). Clinicaltrialsgov. January 2016: NCT01673867.
- Borghaei H, Paz-Ares L, Horn L, Spigel DR, Steins M, Ready NE, et al. Nivolumab versus Docetaxel in Advanced Nonsquamous Non-Small-Cell Lung Cancer. N Engl J Med. 2015 Oct 22;373(17):1627-39.
- Gettinger SN, Brahmer JR, Rizvi NA, Ready N, Chow LQM, Antonia SJ, et al. A phase III comparative study of nivolumab (anti-PD-1; BMS-963558; ONO-4538) versus docetaxel in patients (pts) with previously treated advanced/metastatic nonsquamous non-small-cell lung cancer (NSCLC). Journal of Clinical Oncology. 2013;31(15).
- Paz-Ares L, Horn L, Borghaei H, Spigel D, Steins M, Ready N, et al. Phase III, randomized trial (CheckMate 057) of nivolumab (NIVO) versus docetaxel (DOC) in advanced non-squamous cell (non- SQ) non-small cell lung cancer (NSCLC). Journal of Clinical Oncology. 2015;33(suppl; abstr LBA109).
- Horn L, Brahmer J, Reck M, Borghaei H, Spigel D, Steins M, et al. Phase 3, randomized trial (CheckMate

- 057) of nivolumab (NIVO) vs docetaxel (DOC) in advanced non-squamous (non-SQ) non-small cell lung cancer(NSCLC): Subgroup analyses and patient reported outcomes (PROs). European Journal of Cancer. 2015;51(Supplement 3): S1-S810.
- Li A, Fayette J, Reck M, Spigel D, Barlesi F, Ready N, et al. Phase 3, randomized trial (CheckMate 057) of nivolumab (NIVO) vs. Docetaxel (DOC) in advanced non-squamous (non- SQ) non-small cell lung cancer (NSCLC)-subgroup analyses and patient reported outcomes (PROs). Oncology Research and Treatment. 2016;39: 27.
- Li A, Fayette J, Reck M, Vokes EE, Barlesi F, Felip E, et al. Phase 3, randomized trial (CheckMate 057) of nivolumab vs docetaxel in advanced non-squamous (non-SQ) non-small cell lung cancer (NSCLC): Subgroup analyses and patient reported outcomes (PROs). Annals of Oncology. 2015;26: ix125.
- Gralla RJ, Spigel DR, Bennett B, Taylor F, Penrod JR, DeRosa M, et al. Lung Cancer Symptom Scale (LCSS) as a marker of treatment (tx) benefit with nivolumab (nivo) vs docetaxel (doc) in patients (pts) with advanced (adv) non-squamous (NSQ) NSCLC from CheckMate 057. Journal of Clinical Oncology. 2016;34(Supplement): abstract 9031.
- Reck M, Brahmer J, Bennett B, Taylor F, Penrod JR, DeRosa M, et al. Overall Health Status in Patients With Advanced Non-Squamous NSCLC Treated With Nivolumab or Docetaxel in CheckMate 057. Annals of Oncology. 2016;27(Supplement 6): abstract 1217PD.
- IQWIG report 2016 : https://www.iqwig.de/download/A16-25_Nivolumab_Extract-of-dossier-assessment_V1-0.pdf
- BMS 01/2016: http://packageinserts.bms.com/pi/pi_opdivo.pdf

Chen 2011

- Chen Y-M, Fan W-C, Tsai C-M, Liu S-H, Shih J-F, Chou T-Y, et al. A phase II randomized trial of gefitinib alone or with tegafur/uracil treatment in patients with pulmonary adenocarcinoma who had failed previous chemotherapy. Lung Cancer. 2011;6(6):1110-6.

CTONG0806 2014

- Zhou Q, Cheng Y, Yang JJ, Zhao MF, Zhang L, Zhang XC, et al. Pemetrexed versus gefitinib as a second- line treatment in advanced nonsquamous nonsmall-cell lung cancer patients harboring wild-type EGFR (CTONG0806): a multicenter randomized trial. Annals of Oncology. 2014;25(12):2385-91.
- Yang J, Cheng Y, Zhao M, Zhou Q, Yan HH, Zhang L, et al. A phase II trial comparing pemetrexed with gefitinib as the second-line treatment of nonsquamous NSCLC patients with wild-type EGFR (CTONG0806). Journal of Clinical Oncology. 2013;31(15).

Dai 2013

- Dai H, Xu L, Xia C, Chen W. A randomized clinical study of gefitinib and pemetrexed as second line therapy for advanced non-squamous non-small cell lung cancer. Chinese Journal of Lung Cancer. 2013;16(8):405 - 10.

DATE 2015

- Kim HR, Jang JS, Sun JM, Ahn MJ, Kim DW, Jung I, et al. A randomized, phase II study of gefitinib alone versus nimotuzumab plus gefitinib after platinum-based chemotherapy in advanced non-small cell lung cancer (KCSG LU12-01). Oncotarget. 2016;28, 8(9):15943-51.
- Kim H, Jang J, Sun J, Ahn M, Kim D, Jung I, et al. A randomized, phase II study of nimotuzumab plus gefitinib vs gefitinib in advanced non-small cell lung cancer after platinum- based chemotherapy. Journal of Thoracic Oncology. 2015;10(9): Supplement 2 (S663-S4).

DELTA 2014

- Kawaguchi T, Ando M, Asami K, Okano Y, Fukuda M, Nakagawa H, et al. Randomized phase III trial of erlotinib versus docetaxel as second- or third-line therapy in patients with advanced non-small-cell lung cancer: Docetaxel and erlotinib lung cancer trial (DELTA). Journal of Clinical Oncology. 2014;32(18):1902 - 8.
- Okano Y, Ando M, Asami K, Fukuda M, Nakagawa H, Ibata H, et al. Randomized phase III trial of erlotinib (E) versus docetaxel (D) as second- or third-line therapy in patients with advanced non-small cell lung cancer (NSCLC) who have wild-type or mutant epidermal growth factor receptor (EGFR): Docetaxel and Erlotinib Lung Cancer Trial (DELTA). Journal of Clinical Oncology. 2013;31(15): 8006.
- Ibata H, Ando M, Asami K, Okano Y, Fukuda M, Nakagawa H, et al. Updated overall survival (OS) results of randomized phase III trial of erlotinib (E) versus (v) docetaxel (D) as second- or third-line therapy in patients

with advanced non-small cell lung cancer (NSCLC): Docetaxel and Erlotinib Lung Cancer Trial (DELTA). Journal of Clinical Oncology. 2014;32(15): e19003.
Dittrich 2014
- Dittrich C. A Phase 2 Study of Pemetrexed Versus Pemetrexed Plus Erlotinib in Second-Line Treatment in Patients With Nonsquamous NSCLC. Clinicaltrialsgov. June 2011: NCT00447057.
- Dittrich C, Papai-Szekely Z, Vinolas N, Sederholm C, Hartmann JT, Behringer D, et al. A randomised phase II study of pemetrexed versus pemetrexed + erlotinib as second-line treatment for locally advanced or metastatic non-squamous non-small cell lung cancer. European journal of cancer. 2014;50(9):1571-80.
- Von Pawel J, Papai-Szekely Z, Vinolas N, Sederholm C, Klima M, Desaiah D, et al. A randomized phase 2 study of pemetrexed vs. pemetrexed+erlotinib in second-line treatment for locally advanced or metastatic, non-squamous NSCLC. Journal of Clinical Oncology. 2011;29(15):7526.
E1512 2015
- Neal JW. Erlotinib Hydrochloride and Cabozantinib-s-Malate Alone or in Combination as Second or Third Line Therapy in Treating Patients with Stage IV Non-Small Cell Lung Cancer. Clinicaltrialsgov. November 2016; NCT01708954.
- Neal JW, Dahlberg SE, Wakelee HA, Aisner SC, Bowden M, Huang Y, et al. Erlotinib, cabozantinib, or erlotinib plus cabozantinib as second-line or third-line treatment of patients with EGFR wild-type advanced non-small-cell lung cancer (ECOG-ACRIN 1512): a randomised, controlled, open-label, multicentre, phase 2 trial. Lancet Oncol. 2016;17(12):1661-71.
- Neal J, Dahlberg S, Wakelee H, Aisner S, Bowden M, Carbone D, et al. Cabozantinib (C), erlotinib (E) or the combination (E+C) as second- or third-line therapy in patients with EGFR wild-type (wt) non- small cell lung cancer (NSCLC): A randomized phase 2 trial of the ECOG-ACRIN Cancer Research Group (E1512). Journal of Clinical Oncology. 2015;33(15):8003.
- Neal J, Dahlberg S, Wakelee H, Aisner S, Bowden M, Carbone D, et al. A Randomized Phase 2 Trial of Cabozantinib, Erlotinib or the Combination as 2nd or 3rd Line Therapy in EGFR Wild-Type NSCLC: ECOG-ACRIN E1512. Journal of Thoracic Oncology. 2015;10(9): Supplement 2.
Esteban 2003
- Esteban E, González de Sande L, Fernández Y, Corral N, Fra J, Muñiz I, et al. Prospective randomised phase II study of docetaxel versus paclitaxel administered weekly in patients with non-small-cell lung cancer previously treated with platinum-based chemotherapy. Annals of Oncology. 2003;14(11):1640- 7.
- Blay P, Esteban E, Fra J, Muniz I, Palacio I, Fernandez JL, et al. Randomized phase II study of weekly docetaxel (D) versus paclitaxel (P) in second line advanced non-small cell lung cancer (NSCLC): a Grupo Oncologico del Norte (GON) study. Proceedings of the American Society of Clinical Oncology. 2002;21 (Pt 2):214b, Abstract 2675.
Gerber 2014
- Gerber D, Socinski M, Neal J, Wakelee H, Shirai K, Sequist L, et al. Randomized Phase 2 Study of Tivantinib (ARQ 197) Plus Erlotinib Versus Single-Agent Chemotherapy in Previously Treated KRAS Mutant Advanced Non-Small Cell Lung Cancer (NSCLC) Metastatic Non-Small Cell Lung Cancer. International Journal of Radiation Oncology Biology Physics. 2014;90(5): Supplement 1.
GFPC 05-06 2011
- Vergnenegre A, Corre R, Berard H, Paillotin D, Dujon C, Robinet G, et al. Cost-effectiveness of second- line chemotherapy for non-small cell lung cancer: an economic, randomized, prospective, multicenter phase III trial comparing docetaxel and pemetrexed: the GFPC 05-06 study. Journal of Thoracic Oncology. 2011 Jan; 6(1):161-8.
Groen 2013
- Groen H. Randomized, Double-Blind, Phase 2 Study of Erlotinib with or Without SU011248 In The Treatment Of Metastatic Non-Small Cell Lung Cancer. Clinicaltrialsgov. January 2011: NCT00265317.
- Groen HJM, Socinski MA, Grossi F, Juhasz E, Gridelli C, Baas P, et al. A randomized, double-blind, phase II study of erlotinib with or without sunitinib for the second-line treatment of metastatic non-small- cell lung cancer (NSCLC). Annals of Oncology. 2013;24(9):2382 - 9.
- Groen HJM, Socinski M, Grossi F, Juhasz E, Gridelli C, Baas P, et al. Randomized phase II study of sunitinib (SU) plus erlotinib (E) vs. placebo (P) plus E for the treatment of metastatic non-small cell lung cancer (NSCLC). Annals of Oncology. 2010;21: viii139.

Hainsworth 2010
- Hainsworth JD, Cebotaru CL, Kanarev V, Ciuleanu TE, Damyanov D, Stella P, et al. A phase II, open- label, randomized study to assess the efficacy and safety of AZD6244 (ARRY-142886) versus pemetrexed in patients with non-small cell lung cancer who have failed one or two prior chemotherapeutic regimens. Journal of Thoracic Oncology. 2010; 5(10):1630 - 6.

Han 2011
- Han YH, Han QS, Liu SD. Comparison of pemetrexed and docetaxel as single chemotherapy in advanced non-small cell lung cancer. Chinese Journal of Cancer Prevention and Treatment. 2011;18(1):57 - 9.

HANSHIN Oncology Group 0110 2015
- Nishinoa K, Imamuraa F, Kumagaia T, Katakamib N, Hatab A, Okudab C, et al. A randomized phase II study of bevacizumab in combination with docetaxel or S-1 in patients with non-squamous non-small- cell lung cancer previously treated with platinum based chemotherapy (HANSHIN Oncology Group 0110). Lung Cancer. 2015; 89(2):146-53.

Herbst, 2007*
- Herbst RS, O'Neill VJ, Fehrenbacher L, Belani CP, Bonomi PD, Hart L, et al. Phase II study of efficacy and safety of bevacizumab in combination with chemotherapy or erlotinib compared with chemotherapy alone for treatment of recurrent or refractory non-small-cell lung cancer. Journal of Clinical Oncology. 2007; 25(30):4743-50.

Heymach 2007
- Heymach JV, Johnson BE, Prager D, Csada E, Roubec J, Pesek M, et al. Randomized, placebo-controlled phase II study of vandetanib plus docetaxel in previously treated non-small-cell lung cancer. Journal of Clinical Oncology. 2007;25(27):4270 - 7.
- Herbst R, Johnson B, Rowbottom J, et al. ZD6474 plus docetaxel in patients with previously treated NSCLC: results of a randomized, placebo-controlled phase II trial. Lung Cancer 2005; 49 (suppl 2): S35.

HORG 2013
- Karampeazis A, Voutsina A, Souglakos J, Kentepozidis N, Giassas S, Christofillakis C, et al. Pemetrexed versus erlotinib in pretreated patients with advanced non-small cell lung cancer: A Hellenic Oncology Research Group (HORG) randomized phase 3 study. Cancer. 2013;119(15):2754 - 64.
- Vamvakas L, Agelaki S, Kentepozidis NK, Karampeazis A, Pallis AG, Christophyllakis C, et al. Pemetrexed (MTA) compared with erlotinib (ERL) in pretreated patients with advanced non-small cell lung cancer (NSCLC): Results of a randomized phase III Hellenic Oncology Research Group trial. Journal of Clinical Oncology. 2010;28(15).

Hosomi 2015
- Hosomi Y. A Study of Docetaxel and Ramucirumab Versus Docetaxel and Placebo in the Treatment of Stage IV Non-Small Cell Lung Cancer. Clinicaltrialsgov. December 2015: NCT01703091.
- Yoh K, Hosomi Y, Kasahara K, Yamada K, Takahashi T, Yamamoto N, et al. A randomized, double-blind, phase II study of ramucirumab plus docetaxel vs placebo plus docetaxel in Japanese patients with stage IV non-small cell lung cancer after disease progression on platinum-based therapy. Lung cancer. 2016;99: 186-93.
- Hosomi Y, Yoh K, Kasahara K, Yamada K, Takahashi T, Tanaka K, et al. Docetaxel + ramucirumab (DR) versus docetaxel + placebo (D) as second-line treatment for advanced non-small cell lung cancer(NSCLC): A randomized, phase II, double-blind, multicenter trial in Japan. Journal of Clinical Oncology. 2015;33(15):8054.

ICOGEN 2013
- Shi Y. A Randomized, Double-blind, Multicenter Phase III Trial to Evaluate the Safety and Efficacy of Icotinib and Gefitinib in Advanced NSCLC Patients Previously Treated with Chemotherapy. Clinicaltrialsgov. February 2012: NCT01040780.
- Shi Y, Zhang L, Liu X, Zhou C, Zhang S, Wang D, et al. Icotinib versus gefitinib in previously treated advanced non-small-cell lung cancer (ICOGEN): A randomised, double-blind phase 3 non-inferiority trial. The Lancet Oncology. 2013;14(10):953 - 61.
- Sun Y, Shi Y, Zhang L, Liu X, Zhou C, Wang D. A randomized, doubleblind phase III study of icotinib versus gefitinib in patients with advanced non-small cell lung cancer (NSCLC) previously treated with chemotherapy (icogen). Journal of Thoracic Oncology. 2011;6(6): S317-S8.

- Sun Y, Shi Y, Zhang L, Liu X, Zhou C, Zhang L, et al. Final overall survival and updated biomarker analysis results from the randomized phase III ICOGEN trial. Journal of Clinical Oncology. 2012;30(15):7559.
- Yan S, Yuankai S, Li Z, Xiaoqing L, Zhou C, Li Z, et al. Final overall survival and updated biomarker analysis results from the randomized Phase III ICOGEN Trial. Annals of Oncology. 2012;23(9): ix416.

INTEREST 2008

- Kim ES, Hirsh V, Mok T, Socinski MA, Gervais R, Wu YL, et al. Gefitinib versus docetaxel in previously treated non-small-cell lung cancer (INTEREST): a randomised phase III trial. The Lancet. 2008;372(9652):1809 - 18.
- Douillard JY, Shepherd FA, Hirsh V, Mok T, Socinski MA, Gervais R, et al. Molecular predictors of outcome with gefitinib and docetaxel in previously treated non-small-cell lung cancer: Data from the randomized phase III INTEREST trial. Journal of Clinical Oncology. 2010;28(5):744 - 52.

ISEL 2005

- Thatcher N, Chang A, Parikh P, Rodrigues Pereira J, Ciuleanu T, Pawel J, et al. Gefitinib plus best supportive care in previously treated patients with refractory advanced non-small-cell lung cancer: results from a randomised, placebo-controlled, multicentre study (Iressa Survival Evaluation in Lung Cancer). The Lancet. 2005. p. 1527-37.
- Chang A, Parikh P, Thongprasert S, Tan EH, Perng RP, Ganzon D, et al. Gefitinib (IRESSA) in patients of Asian origin with refractory advanced non-small cell lung cancer: Subset analysis from the ISEL study. Journal of Thoracic Oncology. 2006;1(8):847 - 55.

ISTANA 2010

- Lee DH, Park K, Kim JH, Lee JS, Shin SW, Kang JH, et al. Randomized phase III trial of gefitinib versus docetaxel in non-small cell lung cancer patients who have previously received platinum-based chemotherapy. Clinical Cancer Research. 2010;16(4):1307 - 14.

Janne 2013

- Janne PA, Shaw AT, Pereira JR, Jeannin G, Vansteenkiste J, Barrios C, et al. Selumetinib plus docetaxel for KRAS-mutant advanced non-small-cell lung cancer: A randomised, multicentre, placebo-controlled, phase 2 study. The Lancet Oncology. 2013;14(1):38 - 47.
- Janne P, Shaw A, Pereira J, Jeannin G, Vansteenkiste J, Barrios C, et al. Efficacy and patient-reported outcomes with Selumetinib (AZD6244, ARRY-142866; SEL) + Docetaxel (DOC) in KRAS-mutant advanced non-small cell lung cancer: a randomized Phase II trial. Annals of Oncology. 2012;23(9):ix403- 1233PD.
- Janne P, Tsang Shaw A, Rodrigues Pereira J, Jeannin G, Vansteenkiste J, Barrios C, et al. Phase II double-blind, randomized study of selumetinib (SEL) plus docetaxel (DOC) versus DOC plus placebo as second-line treatment for advanced KRAS mutant non-small cell lung cancer (NSCLC). Journal of Clinical Oncology. 2012;30(15):7503.

JMEI 2004

- EMA 2004 report: http://www.ema.europa.eu/docs/en_GB/document_library/EPAR_-_Scientific_Discussion/human/000564/WC500025606.pdf
- Hanna N, Shepherd FA, Fossella FV, Pereira JR, Demarinis F, Von Pawel J, et al. Randomized phase III trial of pemetrexed versus docetaxel in patients with non-small-cell lung cancer previously treated with chemotherapy. Journal of Clinical Oncology. 2004;22(9):1589 - 97.
- NICE report 22/08/2007: https://www.nice.org.uk/guidance/ta124
- HAS report 2016: https://www.has-sante.fr/portail/upload/docs/application/pdf/2016-06/alimta_cbnpc_pic_reev_avis2modifie_ct14873.pdf
- Lilly report 12/11/2004: CT Registry ID# 488; Clinical Study Summary: Study H3E-MC-JMEI http://lillytrials.com/results/alimta.pdf

Jones 2008

- Jones S, Thompson D, Barton J, Patton J, Shipley D, Greco FA, et al. A randomized phase II trial of oral topotecan versus docetaxel in the second-line treatment of non-small-cell lung cancer. Clinical lung cancer. 2008;9(3):154-9.

Juan 2014

- Juan Ó, Aparisi F, Sánchez-Hernández A, Muñoz-Langa J, Esquerdo G, García-Sánchez J, et al. Intercalated Dosing Schedule of Erlotinib and Docetaxel as a Therapeutic Strategy to Avoid Antagonism and Optimize Its Benefits in Advanced Non-Small-Cell Lung Cancer. A Randomized Phase II Clinical Trial. Clinical Lung Cancer. 2014 Nov 23. pii: S1525-7304.

- Aparisi F, Garcia Sanchez J, Sanchez-Hernandez A, Giner V, Munoz-Langa J, Esquerdo G, et al. Multicenter, open, randomized, phase II study to investigate the sequential administration of docetaxel and intermittent erlotinib versus erlotinib as a second-line therapy for advanced non-small cell lung cancer (NSCLC). Journal of Clinical Oncology. 2011;29(15).
- Aparisi F, Sanchez A, Giner V, Munoz J, Esquerdo G, Garde J, et al. A Multi-center, Open, Randomized, Phase II Study to Investigate the Sequential Administration of Docetaxel and Intermittent Erlotinib Versus Erlotinib as a Second-line Therapy for Advanced Non-Small Cell Lung Cancer (NSCLC). European Journal of Cancer. 2011;47(S1): S630.
- Vidal O, Sánchez J, Sánchez A, Giner V, Munoz Langa J, Esquerdo G, et al. Clinical benefits of sequential administration of docetaxel and intermittent erlotinib as a second-line therapy for advanced non-small cell lung cancer (NSCLC), a phase II randomized study. Journal of Thoracic Oncology. 2011;6(6): P3.030.

Kapoor 2015

- Kapoor A, Kumar N, Narayan S, Nirban R, Maharia S, Beniwal S, et al. A prospective randomized open label phase III study of gefitinib versus docetaxel as second or third line therapy in patients with advanced non-small cell lung cancer in Asian indians. Annals of Oncology. 2015;26(Supplement 1): i29–i44.

Katakami 2014

- Katakami N, Yoshioka H, Okamoto H, Iwamoto Y, Seto T, Takahashi T, et al. Amrubicin (AMR) versus Docetaxel (DTX) as second- or third-line treatment for non-small cell lung cancer (NSCLC): a randomized phase III trial. Annals of oncology. 2014;25((Supplement 4)): iv 426-iv70.

KCSG-LU08-01 2012

- Sun JM, Lee KH, Kim SW, Lee DH, Min YJ, Yun HJ, et al. Gefitinib versus pemetrexed as second-line treatment in patients with non-small cell lung cancer previously treated with platinum-based chemotherapy (KCSG-LU08-01): An open-label, phase 3 trial. Cancer. 2012;118(24):6234 - 42.
- Ahn M, Sun J, Ahn JS, Kim S, Lee KH, Min YJ, et al. Randomized phase III trial of gefitinib or pemetrexed as second-line treatment in patients with non-small cell lung cancer previously treated with platinum- based chemotherapy (KCSG-LU08-01). Journal of Clinical Oncology. 2011;29(15).
- Ahn M, Sun J, Lee K, Ahn J, Kim S, Min Y, et al. Randomized Phase III trial of Gefitinib or Pemetrexed as second-line treatment in patients with non-small cell lung cancer previously treated with platinum- based chemotherapy (KCSG-LU08-01). Journal of Thoracic Oncology. 2011;6(6): S317.

Kelly 2012

- Kelly K. A Randomized, Phase 2b, Multi-center Study of Pralatrexate Versus Erlotinib in Patients with Stage IIIB/IV Non-Small Cell Lung Cancer After Failure of at Least 1 Prior Platinum-based Treatment. Clinicaltrialsgov. December 2010: NCT00606502.
- Kelly K, Azzoli CG, Zatloukal P, Albert I, Jiang PY, Bodkin D, et al. Randomized phase 2b study of pralatrexate versus erlotinib in patients with stage IIIB/IV non-small-cell lung cancer (NSCLC) after failure of prior platinum-based therapy. Journal of Thoracic Oncology. 2012 Jun;7(6):1041-8.
- Kelly K, Azzoli CG, Patel JD, Weems G, Zatloukal P. Randomized phase 2B study of pralatrexate vs erlotinib in patients with stage IIIB/IV non-small cell lung cancer (NSCLC) after failure of prior platinum- based therapy. Annals of Oncology. 2010;21: viii8.

KEYNOTE-010 2016

- FDA report 2017: https://www.accessdata.fda.gov/drugsatfda_docs/label/2017/125514s017s018lbl.pdf
- EMA report 2017: http://www.ema.europa.eu/docs/en_GB/document_library/EPAR_-_Product_Information/human/003820/WC500190990.pdf
- Herbst RS. Study of Two Doses of Pembrolizumab (MK-3475) Versus Docetaxel in Previously Treated Participants With Non-Small Cell Lung Cancer (MK-3475-010/KEYNOTE-010). Clinicaltrialsgov. May 2017:NCT01905657.
- Herbst RS, Baas P, Kim D-W, Felip E, Pérez-Gracia JL, Han J-Y, et al. Pembrolizumab versus docetaxel for previously treated, PD-L1-positive, advanced non-small-cell lung cancer (KEYNOTE-010): a randomised controlled trial. Lancet. 2016; 387(10027):1540-50.
- NICE report 2017: https://www.nice.org.uk/guidance/ta428/resources/pembrolizumab-for-treating-pdl1positive-nonsmallcell-lung-cancer-after-chemotherapy-pdf-82604670410437
- IQWIG report 2016: https://www.iqwig.de/download/A16-55_Pembrolizumab_Extract-of-dossier-assessment-V1-0.pdf

- Herbst R, Gurpide A, Surmont V, Kim D, Waqar S, Herder J, et al. A phase II/III randomized trial of two doses of MK-3475 versus docetaxel in previously treated subjects with non-small cell lung cancer. Journal of Clinical Oncology. 2014;32(15 Supplement): TPS8124.
- Baas P, Garon EB, Herbst RS, Felip E, Perez-Gracia JL, Han J-Y, et al. Relationship between level of PD-L1 expression and outcomes in the KEYNOTE-010 study of pembrolizumab vs docetaxel for previously treated, PD-Ll-Positive NSCLC. Journal of Clinical Oncology. 2016;34 (Supplement): abstract 9015.
- Garon EB, Herbst RS, Kim DW, Felip E, Perez-Gracia JL, Han JY, et al. Pembrolizumab vs docetaxel for previously treated advanced NSCLC with a PD-L1 tumor proportion score (TPS) 1%-49%: Results from KEYNOTE-010. Journal of Clinical Oncology. 2016;34(Supplement): abstract 9024.
- Herbst R, Garon E, Kim DW, Cho BC, Gadgeel S, Léna H, et al. Keynote-010: Durable clinical benefit in patients with previously treated, PD-l1-expressing NSCLC who completed pembrolizumab. Journal of Thoracic Oncology. 2017;12(1): S254-S5.
- Herbst RS, Baas P, Kim DW, Felip E, Pérez-Gracia JL, Han JY, et al. Pembrolizumab versus Docetaxel for Previously Treated, PD-L1–Expressing NSCLC: Updated Outcomes of KEYNOTE-010. Annals of Oncology. 2016;27(Supplement 6):vi416-54.
- Barlesi F, Garon EB, Kim DW, Felip E, Han JY, Kim JH, et al. Assessment of Health-Related Quality of Life in KEYNOTE-010: A Phase 2/3 Study of Pembrolizumab Versus Docetaxel in Patients with Previously Treated Advanced NSCLC. Annals of Oncology. 2016;27(Supplument 6):vi416-vi54.

Kim A 2012

- Kim E. Randomized Phase III Study of Docetaxel or Pemetrexed with or Without Cetuximab in Patients with Recurrent or Progressive Non-Small Cell Lung Cancer After Platinum-Based Therapy. Clinicaltrialsgov. June 2012: NCT00095199.
- Kim ES, Neubauer M, Cohn A, Schwartzberg L, Garbo L, Caton J, et al. Docetaxel or pemetrexed with or without cetuximab in recurrent or progressive non-small-cell lung cancer after platinum-based therapy: A phase 3, open-label, randomised trial. The Lancet Oncology. 2013;14(13):1326 - 36.

Kim B 2012

- Kim E. Randomized Phase III Study of Docetaxel or Pemetrexed with or Without Cetuximab in Patients with Recurrent or Progressive Non-Small Cell Lung Cancer After Platinum-Based Therapy. Clinicaltrialsgov. June 2012: NCT00095199.

Kim 2015

- Kim Y, Cho E, Woo H, Hong J, Ahn H, Park I, et al. Randomized Phase II Study of Pemetrexed Versus Gefitinib in Previously Treated Patients with Advanced Non-Small Cell Lung Cancer. Cancer Res Treat 2015. doi: 10.4143/crt.2014.307.
- Kim YS, Cho EK, Sym SJ, Hong J, Park I, Ahn HK, et al. Randomized phase II study of pemetrexed versus gefitinib in previously treated patients with advanced non-small cell lung cancer. Journal of Clinical Oncology. 2014;32(15): e19039.
- Hong J, Kyung SY, Lee SP, Park JW, Jung SH, Sym SJ, et al. Randomized phase II study of pemetrexed versus gefitinib for patients with previously treated non-small cell lung cancer. Journal of Thoracic Oncology. 2010;5(12): S401.
- Cho E, Kyung S, Sym S, Kim Y, Lee S, Park J, et al. Randomized phase II trial of pemetrexed versus gefitinib in previously treated non-small cell lung cancer: preliminary results. Journal of Thoracic Oncology. 2009;4(9): S672.

Krzakowski 2010

- Krzakowski M, Ramlau R, Jassem J, Szczesna A, Zatloukal P, Von Pawel J, et al. Phase III trial comparing vinflunine with docetaxel in second-line advanced non-small-cell lung cancer previously treated with platinum-containing chemotherapy. Journal of Clinical Oncology. 2010;28(13):2167-73.

Kuo 2013

- Kuo H. Study to Assess Safety/Tolerability/Efficacy of Gefitinib Versus Docetaxel in Locally Advanced or Metastatic Non-Small Cell Lung Cancer (NSCLC). Clinicaltrialsgov. August 2013: NCT00536107.
- Pharmaceutical report: http://www.astrazenecaclinicaltrials.com/Submission/View?id=2086

Lee 2013

- Lee DH. A Randomized Phase 2 Study Comparing Erlotinib-Pemetrexed, Pemetrexed Alone, and Erlotinib Alone, as Second-Line Treatment for Non-Smoker Patients with Locally Advanced or Metastatic

Nonsquamous Non-Small Cell Lung Cancer. Clinicaltrialsgov. January 2013: NCT00550173.
- Lee DH, Lee JS, Kim SW, Rodrigues-Pereira J, Han B, Song XQ, et al. Three-arm randomised controlled phase 2 study comparing pemetrexed and erlotinib to either pemetrexed or erlotinib alone as second- line treatment for never-smokers with non-squamous non-small cell lung cancer. Eur J Cancer. 2013;49(15):3111-21.
- Lee D, Lee J, Kim S, Rodrigues Pereira J, Han B, Song X, et al. A randomized Phase 2 study of Erlotinib plus Pemetrexed versus Erlotinib or Pemetrexed alone as second-line treatment for never-smoker patients with non-squamous advanced non-small cell lung cancer (NSCLC). Annals of Oncology. 2012;23(9): ix400–ix46.
- Lee D, Lee J, Wang J, Hsia T, Wang X, Kim J, et al. Pemetrexed-Erlotinib, Pemetrexed Alone, or Erlotinib Alone as Second-Line Treatment for East Asian and Non-East Asian Never-Smokers with Locally Advanced or Metastatic Nonsquamous Non-Small Cell Lung Cancer: Exploratory Subgroup Analysis of a Phase II Trial. Cancer Research and Treatment 2015; 47(4):616-29.

Levy 2014
- Levy B, Spira A, Becker D, Evans T, Schnadig I, Camidge DR, et al. A randomized, phase 2 trial of docetaxel with or without PX-866, an irreversible oral phosphatidylinositol 3-kinase inhibitor, in patients with relapsed or metastatic non-small-cell lung cancer. Journal of Thoracic Oncology. 2014;9(7):1031 - 5.

Li 2010
- Li H, Wang X, Hua F. Second-line treatment with gefitinib or docetaxel for advanced non-small cell lung cancer. Chinese Journal of Clinical Oncology. 2010;37(1):16 - 8.

Li 2012
- Li R, Sun L, Wang J, Qian J, Wang Z, Jiao X. Pemetrexed versus docetaxel in second line non-small-cell lung cancer: Results and subsets analyses of a multi-center, randomized, exploratory trial in Chinese patients. Pulmonary Pharmacology and Therapeutics. 2012;25(5):364 - 70.

Li 2013
- Li T, Piperdi B, Walsh WV, Kim M, Beckett LA, Gucalp R, et al. Randomized Phase 2 Trial of Pharmacodynamic Separation of Pemetrexed and Intercalated Erlotinib Versus Pemetrexed Alone for Advanced Nonsquamous, Non-small-cell Lung Cancer. Clinical lung cancer. 2017;18(1):60-7.
- Li T, Piperdi B, Walsh W, Kim M, Gucalp R, Haigentz M, et al. Randomized phase II study of pharmacodynamic separation (PDS) of pemetrexed (Pem) and erlotinib (Erl) versus pem alone in patients (pts) with advanced non-small cell lung cancer (NSCLC). Journal of Clinical oncology. 2013;31(5):8097.
- Li T, Piperdi B, Walsh W, Kim M, Beckett L, Wen H, et al. Pharmacodynamic separation (PDS) of pemetrexed (Pem) and erlotinib (Erl) in patients (pts) with advanced, EGFR wild-type (wt) Non-Small Cell Lung Cancer (NSCLC): A randomized phase II trial. Journal of Clinical Oncology 2015;33(15):8044.

Li 2014
- Wang S. A Randomized Phase II Trial of Erlotinib Versus Pemetrexed as Second-Line Therapy in Treating Patients With Advanced EGFR Wild-Type and EGFR FISH-Positive Lung Adenocarcinoma. Clinicaltrialsgov. September 2014: NCT01565538.
- Li N, Ou W, Yang H, Liu QW, Zhang SL, Wang BX, et al. A randomized phase 2 trial of erlotinib versus pemetrexed as second-line therapy in the treatment of patients with advanced EGFR wild-type and EGFR FISH-positive lung adenocarcinoma. Cancer. 2014;120(9):1379 - 86.
- Wang SY, Li N, Ou W, Zhang L, Zhang SL. A phase II trial of erlotinib versus pemetrexed as second-line therapy in treating patients with advanced EGFR wild-type and EGFR fish-positive lung adenocarcinoma. Journal of Thoracic Oncology. 2013;8: S575.

Liu, 2015
- Liu Z, Wei Z, Hu Y, Gao F, Hao L, Fang P, et al. A phase II open-label clinical study of comparing nab- paclitaxel with pemetrexed as second-line chemotherapy for patients with stage IIIB/IV non-small-cell lung cancer. Med Oncol. 2015; 32(8):216.

LUME-COLUMBUS, 2017
- Boehringer I. LUME-Columbus: Nintedanib Plus Docetaxel in Advanced Non-Small Cell Lung Cancer with Translational Research. Clinicaltrialsgov. February 2017: NCT02231164.
- Boehringer Ingelheim report 2017: https://trials.boehringer-ingelheim.com/public/trial_results_documents/1199/1199.128_Statement_DR.pdf

LUME-Lung 1 2014
- EMA 2015 report: http://www.ema.europa.eu/docs/en_GB/document_library/EPAR_-
- _Product_Information/human/002569/WC500179970.pdf
- Reck M. Multicentre, Randomised, Double-blind, Phase III Trial to Investigate the Efficacy and Safety of Oral BIBF 1120 Plus Standard Docetaxel Therapy Compared to Placebo Plus Standard Docetaxel Therapy in Patients with Stage IIIB/IV or Recurrent Non-Small Cell Lung Cancer After Failure of First Line Chemotherapy. Clinicaltrialsgov. November 2014: NCT00805194.
- Reck M, Kaiser R, Mellemgaard A, Douillard JY, Orlov S, Krzakowski M, et al. Docetaxel plus nintedanib versus docetaxel plus placebo in patients with previously treated non-small-cell lung cancer (LUME- Lung 1): A phase 3, double-blind, randomised controlled trial. The Lancet Oncology. 2014;15(2):143 - 55.
- Novello S, Kaiser R, Mellemgaard A, Douillard JY, Orlov S, Krzakowski M, et al. Analysis of patient- reported outcomes from the LUME-Lung 1 trial: a randomised, double-blind, placebo-controlled, Phase III study of second-line nintedanib in patients with advanced non-small cell lung cancer. Eur J Cancer. 2015 Feb;51(3):317-26.
- NICE report 22/07/2015: https://www.nice.org.uk/guidance/ta347
- IQWIG report 30/03/2015: https://www.iqwig.de/download/A15-01_Nintedanib_Extract-of-dossier-assessment.pdf
- HAS report 2015: https://www.has-sante.fr/portail/upload/docs/evamed/CT-14048_VARGATEF_PIC_INS_Avis3_CT14048.pdf
- Reck M, Mellemgaard A, Douillard JY, Orlov S, Krzakowski M, Von Pawel J, et al. Nintedanib (BIBF 1120) + docetaxel as second-line therapy in patients with stage IIIB/IV or recurrent NSCLC: Results of the phase III, randomised, double-blind LUME-Lung 1 trial. Lung Cancer. 2014;83: S12.
- Reck M, Novello S, Mellemgaard A, Orlov S, Kaiser R, Barrueco J, et al. Impact of tumor burden on the overall survival analysis of the lume-lung 1 study: A randomized, double-blind phase 3 trial of nintedanib (BIBF 1120) + docetaxel in NSCLC patients progressing after first-line chemotherapy. Journal of Thoracic Oncology. 2013;8: S196.
- Mellemgaard A, Douillard JY, Novello S, Kaiser R, Orlov S, Krzakowski M, et al. Patient-reported outcomes from the lume-lung 1 trial: A randomised, double-blind, placebo-controlled phase III study in second-line advanced NSCLC patients. Journal of Thoracic Oncology. 2014;9(9): S152 - S3.
- Reck M, Kaiser R, Mellemgaard A, Douillard J, Orlov S, Krzakowski M, et al. Nintedanib (BIBF 1120) plus docetaxel in NSCLC patients progressing after first-line chemotherapy: LUME Lung 1, a randomized, double-blind phase III trial. Journal of Clinical Oncology. 2013;31(18): LBA8011.
- Mellemgaard A, Kaiser R, Douillard JY, Orlov SV, Krzakowski MJ, Von Pawel J, et al. Analysis of overall survival in adenocarcinoma NSCLC patients receiving 2nd line combination treatment with nintedanib (BIBF 1120) + docetaxel in the LUME-Lung 1 trial: A randomized, double-blind, placebo-controlled phase 3 study. European Journal of Cancer. 2013;49: S798.
- Reck M, Buchner H, Gottfried M, Novello S, Mellemgaard A, Heigener D, et al. Tumour growth over time in patients with advanced non-small cell lung cancer treated with nintedanib plus docetaxel or placebo plus docetaxel: Analysis of data from the LUME-Lung 1 study. European Journal of Cancer. 2015;51: Supplement 3 (3102).
- Wu Y, Cheng Y, Kim B, Lu S, Gaschler-Markefski B, Kaiser R, et al. Efficacy of nintedanib/docetaxel in East Asian patients with lung adenocarcinoma (ADE): analysis from the LUME-Lung 1 study. Annals of Oncology. 2015;26: Supplement 9 (ix 133).
- Bondarenko I, Reck M, Krzakowski M, Bennouna J, Kaiser R, Novello S, et al. Efficacy of nintedanib/docetaxel after bevacizumab, pemetrexed or taxanes therapy. Journal of Thoracic Oncology. 2015;10(9):S323-S4.
- Heigener D, Gottfried M, Bennouna J, Bondarenko I, Douillard JY, Krzakowski M, et al. Efficacy and safety of nintedanib/docetaxel in patients with lung adenocarcinoma: further analyses from the LUME-Lung 1 study. Annals of Oncology. 2016;27(Supplement 6):abstract 1276P.
- Boehringer Ingelheim report 2016: https://trials.boehringer-ingelheim.com/public/trial_results_documents/1199/1199.14_119914c296153FUpdf.pdf

LUME-LUNG 2 2013
- Hanna N. A Randomized Double-blind Multicenter Phase III Trial of Nintedanib Plus Pemetrexed vs.

Pemetrexed/ Placebo in Advanced or Recurrent Non-Small Cell Lung Cancer Patients After Failure of First Line Therapy. Clinicaltrialsgov. November 2014: NCT00806819.

- Hanna NH, Kaiser R, Sullivan RN, Aren OR, Ahn MJ, Tiangco B, et al. Nintedanib plus pemetrexed versus placebo plus pemetrexed in patients with relapsed or refractory, advanced non-small cell lung cancer (LUME-Lung 2): A randomized, double-blind, phase III trial. Lung cancer. 2016;102:65-73.
- Hanna NH, Kaiser R, Sullivan RN, Aren OR, Ahn MJ, Tiangco B, et al. Lume-lung 2: A multicenter, randomized, double-blind, phase III study of nintedanib plus pemetrexed versus placebo plus pemetrexed in patients with advanced nonsquamous non-small cell lung cancer (NSCLC) after failure of first-line chemotherapy. Journal of Clinical Oncology. 2013;31(15).
- Pharmaceutical Report: http://trials.boehringer-ingelheim.com/content/dam/internet/opu/clinicaltrial/com_EN/results/1199/1199.14_U12-2160-01-PE-DS-DR.pdf?bcsi_scan_628cd39dca2568d2=0&bcsi_scan_filename=1199.14_U12-2160-01-PE-DS-DR.pdf

Lux-Lung 8 2015

- FDA report 2016: https://www.accessdata.fda.gov/drugsatfda_docs/label/2016/201292s009lbl.pdf
- Soria J. LUX-Lung 8: A Phase III Trial of Afatinib (BIBW 2992) Versus Erlotinib for the Treatment of Squamous Cell Lung Cancer After at Least One Prior Platinum Based Chemotherapy. Clinicaltrialsgov October 2014: NCT01523587.
- Soria JC, Felip E, Cobo M, Lu S, Syrigos K, Lee KH, et al. Afatinib versus erlotinib as second-line treatment of patients with advanced squamous cell carcinoma of the lung (LUX-Lung 8): an open-label randomised controlled phase 3 trial. Lancet Oncol. 2015 Aug;16(8):897-907.
- NICE report 2017: https://www.nice.org.uk/guidance/ta444/resources/afatinib-for-treating-advanced-squamous-nonsmallcell-lung-cancer-after-platinumbased-chemotherapy-terminated-appraisal-pdf-82604784624325
- IQWIG report 2016: https://www.iqwig.de/download/A16-22_Afatinib_Extract-of-dossier-assessment.pdf
- Goss G, Lu S, Felip E, Ardizzoni A, Georgoulias V, Gadgeel S, et al. LUX-lung 8: A randomized, open- label, phase III trial of afatinib vs. erlotinib in patients with advanced squamous cell carcinoma of the lung as second-line therapy following first-line platinum-based chemotherapy. Annals of Oncology 2012;23(SUPPL. 9): ix174.
- Soria J, Felip E, Cobo M, Lu S, Syrigos K, Lee K, et al. Afatinib (A) vs erlotinib (E) as second-line therapy of patients (pts) with advanced squamous cell carcinoma (SCC) of the lung following platinum-based chemotherapy: Overall survival (OS) analysis from the global phase III trial LUX-Lung 8 (LL8). Journal of Clinical Oncology. 2015;33(15):8002.
- Gadgeel S, Dols M, Felip E, Soria J, Lee K, Lu S, et al. Afatinib (A) vs erlotinib (E) as second-line treatment of patients (pts) with advanced squamous cell carcinoma (SCC) of the lung following first- line platinum-based chemotherapy: Patient-reported outcome (PRO) data from the LUX-Lung 8 Phase III global trial. Journal of Clinical Oncology. 2015;33(15):8100.
- Hirsh V, Gadgeel S, Soria J, Felip E, Cobo M, Lu S, et al. LUX-lung 8: A global phase III trial of Afatinib (A) vs Erlotinib (E) as second-line treatment in Patients (Pts) with advanced Squamous Cell Carcinoma (SCC) of the lung following first-line platinum-based chemotherapy. Chest 2015;148(4): (meeting abstract).
- Lu S, Wang B, Goss GD, Felip E, Georgoulias V, Soria J-C, et al. Afatinib (A) vs erlotinib (E) as second-line treatment of patients (PTS) with advanced squamous cell carcinoma (SCC) of the lung following first-line platinum-based chemotherapy: Lux-lung 8 (LL8), a phase III global trial. Annals of Oncology. 2015;26: i34-i.
- Goss G, Felip E, Cobo M, Lu S, Syrigos K, Li K, et al. Phase III trial of afatinib vs erlotinib in patients with squamous cell carcinoma (SCC) of the lung (LUX-Lung 8): EGFR molecular aberrations and survival outcomes. European Journal of Cancer. 2015;51: Supplement 3 (3084).
- Popat S, Felip E, Cobo M, Fulop A, Dayen C, Trigo J, et al. Second-line afatinib vs erlotinib in patients with advanced squamous cell carcinoma (SCC) of the lung: patient-reported outcome (PRO) data from the global LUX-Lung 8 (LL8) Phase III trial. European Journal of Cancer. 2015;51: Supplement 3 (3085).
- Felip E, Soria JC, Cobo M, Lu S, Syrigos K, Lee KH, et al. Second-Line Afatinib versus Erlotinib for Patients

with Squamous Cell Carcinoma of the Lung (LUX-Lung 8): Analysis of Tumour and Serum Biomarkers. Journal of Thoracic Oncology. 2017;12(1):P3.02b-003.
- Goss G, Cobo M, Lu S, Syrigos K, Morabito A, Albert I, et al. Second-Line Afatinib for Advanced Squamous Cell Carcinoma of the Lung: Analysis of Afatinib Long-Term Responders in the Phase III LUX-Lung 8 Trial. Journal of Thoracic Oncology. 2017;12(1): OA23.03.
- Goss GD, Lee KH, Felip E, Cobo M, Syrigos KN, Göker E, et al. Evaluation of VeriStrat, a serum proteomic test, in the randomized, open-label, phase 3 LUX-Lung 8 (LL8) trial of afatinib (A) versus erlotinib (E) for the second-line treatment of advanced squamous cell carcinoma (SCC) of the lung. Journal of Clinical Oncology. 2016;34(e20510).
- Goss G, Lee KH, Felip E, Cobo M, Syrigos K, Goker E, et al. Evaluation of VeriStrat®, a serum proteomic test, in the randomised, open-label, Phase III LUX-Lung 8 trial of afatinib versus erlotinib for the second-line treatment of advanced squamous cell carcinoma of the lung. Annals of Oncology. 2016;27(Supplement 6): vi416-vi54.
- Boehringer-ingelheim report 2014: http://trials.boehringer-ingelheim.com/content/dam/internet/opu/clinicaltrial/com_EN/results/1200/1200.125_c02191506-02_PE_DR.pdf

Maitland 2014
- Maitland M. A Randomized Phase II Trial Comparing Cetuximab with Concurrent Pemetrexed/Cetuximab Therapy for Non-Small Cell Lung Cancer Refractory to Primary Treatment. Clinicaltrialsgov. December 2013: NCT00203931.
- Maitland ML, Levine MR, Lacouture ME, Wroblewski KE, Chung CH, Gordon IO, et al. Evaluation of a novel rash scale and a serum proteomic predictor in a randomized phase II trial of sequential or concurrent cetuximab and pemetrexed in previously treated non-small cell lung cancer. BMC Cancer. 2014;14: 5.

Marangolo 2000
- Marangolo, Cognetti, Di C, Selvaggi, Scagliotti, G V. Preliminary results of a phase II randomized trial of docetaxel vs vinorelbine as second-line treatment in previously cisplatin-treated advanced non- small cell lung cancer (NSCLC). Lung cancer (Amsterdam, Netherlands) 2000;29(9 Suppl 1): S 75

MARQUEE 2013
- Scagliotti G, von Pawel J, Novello S, Ramlau R, Favaretto A, Barlesi F, et al. Phase III Multinational, Randomized, Double-Blind, Placebo-Controlled Study of Tivantinib (ARQ 197) Plus Erlotinib Versus Erlotinib Alone in Previously Treated Patients with Locally Advanced or Metastatic Nonsquamous Non-Small-Cell Lung Cancer. Journal of Clinical Oncology. 2015; 33(24):2667–74.
- Novello S, Scagliotti G, Ramlau R, Favaretto A, Barlesi F, Akerley W, et al. Efficacy analysis for molecular subgroups in marquee: A randomized, doubleblind, placebo-controlled, phase 3 trial of tivantinib (ARQ 197) plus erlotinib versus placebo plus erlotinib in previously treated patients with locally advanced or metastatic, nonsquamous, non-small cell lung cancer (NSCLC). Journal of Thoracic Oncology. 2013;8: S901-S902.
- Scagliotti G, Novello S, Ramlau R, Favaretto A, Barlesi F, Akerley W, et al. MARQUEE: A randomized, double-blind, placebo-controlled, phase 3 trial of tivantinib (ARQ 197) plus erlotinib versus placebo plus erlotinib in previously treated patients with locally advanced or metastatic, non-squamous, non- small-cell lung cancer (NSCLC). European Journal of Cancer. 2013;49: S798 - S9.
- Scagliotti G, Akerley W, Von Pawel J, Roder J, Shuster D, Schwartz B, et al. Retrospective evaluation, of the randomized Phase 3 MARQUEE trial of tivantinib (T) + erlotinib (E) versus placebo (P) + erlotinib
- (E) using VeriStrat in patients with previously treated nonsquamous NSCLC. Cancer Research 2015;75(15): supplement 1.

MARVEL 2013
- Adjei A. Pemetrexed Disodium or Erlotinib Hydrochloride as Second-Line Therapy in Treating Patients with Advanced Non-Small Cell Lung Cancer. Clinicaltrialsgov. November 2013: NCT00738881.

METLung 2014
- Spigel D, Edelman M, O'Byrne K, Paz-Ares L, Shames D, Yu W, et al. Onartuzumab plus erlotinib versus erlotinib in previously treated stage IIIb or IV NSCLC: Results from the pivotal phase III randomized, multicenter, placebo-controlled METLung (OAM4971g) global trial. Journal of Clinical Oncology. 2014;32(15):8000.

Moran 2014

- Moran T. A Study Evaluating Dalotuzumab (MK-0646) in Combination with Erlotinib for Participants with Non-Small Cell Lung Cancer (MK-0646-007). Clinicaltrialsgov. March 2017: NCT00654420.
- Moran T, Felip E, Keedy V, Borghaei H, Shepherd FA, Insa A, et al. Activity of dalotuzumab, a selective anti-IGF1R antibody, in combination with erlotinib in unselected patients with Non-small-cell lung cancer: a phase I/II randomized trial. Exp Hematol Oncol. 2014;3(1):26.
- Rosell R, Moran T, Felip E, Torres JMS, Borghaei H, Guan S, et al. An open label, randomized ph II study evaluating dalotuzumab combined with erlotinib in patients with non-small cell lung cancer following failure of prior chemotherapy. Journal of Thoracic Oncology. 2011;6(6): S358 - S9.

Natale 2009

- Natale RB, Bodkin D, Govindan R, Sleckman BG, Rizvi NA, Cap. Vandetanib versus gefitinib in patients with advanced non-small-cell lung cancer: results from a two-part, double-blind, randomized phase II study. Journal of Clinical Oncology. 2009;27(15):2523-9.
- Natale R, Bodkin D, Govindan R, et al. A comparison of the antitumour efficacy of ZD6474 and gefitinib (Iressa™) in patients with NSCLC: results of a randomized, double-blind phase II study. Lung Cancer 2005; 49(suppl 2): S37.

NCCTG N0626 2011

- Adjei AA. Pemetrexed Disodium with or Without Sorafenib as Second-Line Therapy in Treating Patients with Stage IIIB or Stage IV Non-Small Cell Lung Cancer. Clinicaltrialsgov. February 2017: NCT00454194.
- Molina JR, Dy GK, Foster NR, Allen Ziegler KL, Adjei A, Rowland KM, et al. A randomized phase II study of pemetrexed (PEM) with or without sorafenib (S) as second-line therapy in advanced non-small cell lung cancer (NSCLC) of nonsquamous histology: NCCTG N0626 study. Journal of Clinical Oncology. 2011;29(15).

NCIC CTG BR.26 2014

- Ellis P. A Double Blind Placebo Controlled Randomized Trial of PF-804 in Patients With Incurable Stage IIIB/IV Non-Small Cell Lung Cancer After Failure of Standard Therapy for Advanced or Metastatic Disease. Clinicaltrialsgov. September 2014: NCT01000025.
- Ellis PM, Shepherd FA, Millward M, Perrone F, Seymour L, Liu G, et al. Dacomitinib compared with placebo in pretreated patients with advanced or metastatic non-small-cell lung cancer (NCIC CTG BR.26): A double-blind, randomised, phase 3 trial. The Lancet Oncology. 2014;15(12):1379 - 88.
- Ellis PM, Liu G, Millward M, Perrone F, Shepherd FA, Sun S, et al. NCIC CTG BR.26: A phase III randomized, double blind, placebo controlled trial of dacomitinib versus placebo in patients with advanced/metastatic non-small cell lung cancer (NSCLC) who received prior chemotherapy and an EGFR TKI. Journal of Clinical Oncology. 2014;32(15).

NVALT-10, 2013*

- Aerts JG, Codrington H, Lankheet NA, Burgers S, Biesma B, Dingemans AM, et al. A randomized phase II study comparing erlotinib versus erlotinib with alternating chemotherapy in relapsed non- small-cell lung cancer patients: the NVALT-10 study. Ann Oncol. 2013 Nov; 24(11):2860-5.

OAK, 2016

- FDA report 2016: https://www.accessdata.fda.gov/drugsatfda_docs/label/2016/761041lbl.pdf
- Rittmeyer A, Barlesi F, Waterkamp D, Park K, Ciardiello F, von Pawel J, et al. Atezolizumab versus docetaxel in patients with previously treated non-small-cell lung cancer (OAK): a phase 3, open-label, multicentre randomised controlled trial. Lancet. 2017;389(10066):255-65.
- Gadgeel S, Ciardiello F, Rittmeyer A, Barlesi F, Cortinovis D, Barrios C, et al. OAK, a randomized ph III study of atezolizumab vs docetaxel in patients with advanced NSCLC: Results from subgroup analyses. Journal of Thoracic Oncology. 2017;12(1):S9-S10.

Paz-Ares 2008

- Paz-Ares L, Ross H, O'Brien M, Riviere A, Gatzemeier U, Pawel J, et al. Phase III trial comparing paclitaxel poliglumex vs docetaxel in the second-line treatment of non-small-cell lung cancer. British Journal of Cancer. 2008. p. 1608-13.

POPLAR, 2016

- Fehrenbacher L. A Randomized Phase 2 Study of Atezolizumab (an Engineered Anti-PDL1 Antibody) Compared With Docetaxel in Participants With Locally Advanced or Metastatic Non-Small Cell Lung Cancer Who Have Failed Platinum Therapy - "POPLAR". Clinicaltrialsgov. April 2017: NCT01903993.

- Fehrenbacher L, Spira A, Ballinger M, Kowanetz M, Vansteenkiste J, Mazieres J, et al. Atezolizumab versus docetaxel for patients with previously treated non-small-cell lung cancer (POPLAR): a multicentre, open-label, phase 2 randomised controlled trial. Lancet. 2016; 387(10030):1837-46.
- Spira A, Park K, Mazières J, Vansteenkiste J, Rittmeyer A, Ballinger M, et al. Efficacy, safety and predictive biomarker results from a randomized phase II study comparing MPDL3280A vs docetaxel in 2L/3L NSCLC (POPLAR). Journal of Clinical Oncology 2015;33(15).
- Smith DA, Vansteenkiste JF, Fehrenbacher L, Park K, Mazieres J, Rittmeyer A, et al. Updated survival and biomarker analyses of a randomized phase II study of atezolizumab vs docetaxel in 2L/3L NSCLC (POPLAR). Journal of Clinical Oncology. 2016;34(Supplement): abstract 9028.
- Mazieres J, Fehrenbacher L, Rittmeyer A, Spira AI, Park K, Smith DA, et al. Non-classical response measured by immune-modified RECIST and post-progression treatment effects of atezolizumab in 2L/3L NSCLC: results from the randomized phase II study POPLAR. Journal of Clinical Oncology. 2016;34(Supplement): abstract 9032.

PROSE, 2013*

- Gregorc V, Novello S, Lazzari C, Barni S, Aieta M, Mencoboni M, et al. Predictive value of a proteomic signature in patients with non-small-cell lung cancer treated with second-line erlotinib or chemotherapy (PROSE): a biomarker-stratified, randomised phase 3 trial. Lancet Oncol 2014;15(7):713-21.
- Sorlini C, Barni S, Petrelli F, Novello S, De Marinis F, De Pas TM, et al. PROSE: Randomized proteomic stratified phase III study of second line erlotinib versus chemotherapy in patients with inoperable non-small cell lung cancer (NSCLC). Journal of Clinical Oncology 2011;29(15).
- Lazzari C, Novello S, Barni S, Aieta M, De Marinis F, De Pas T, et al. Randomized proteomic stratified phase III study of second-line erlotinib (E) versus chemotherapy (CT) in patients with inoperable non- small cell lung cancer (PROSE). Journal of Clinical Oncology 2013;31(18).
- Gregorc V. Tissue biomarker analysis in PROSE, a randomized proteomic stratified phase III study of second line erlotinib (E) versus chemotherapy (CT) in patients with inoperable non-small cell lung cancer (NSCLC). European Journal of Cancer 2013;49(2).

Ramalingam 2011

- Ramalingam SS, Spigel DR, Chen D, Steins MB, Engelman JA, Schneider CP, et al. Randomized phase II study of erlotinib in combination with placebo or R1507, a monoclonal antibody to insulin-like growth factor-1 receptor, for advanced-stage non-small-cell lung cancer. Journal of Clinical Oncology. 2011;29(34):4574-80.
- Ramalingam S, Spigel D, Steins M, Engelman J, Schneider C, Novello S, et al. Randomized, double- blind, phase II study of erlotinib in combination with placebo or R1507, a monoclonal antibody to insulin-like growth factor receptor-1 (IGF-1R), for advanced-stage non-small cell lung cancer (NSCLC). Journal of Clinical Oncology. 2011;29(15):7527.

Ramalingam 2012

- Ramalingam S. A Randomized Trial Of PF-00299804 Taken Orally Versus Erlotinib Taken Orally For Treatment Of Advanced Non-Small Cell Lung Cancer That Has Progressed After One Or Two Prior Chemotherapy Regimen. Clinicaltrialsgov. July 2015: NCT00769067.
- Ramalingam SS, Blackhall F, Krzakowski M, Barrios CH, Park K, Bover I, et al. Randomized phase II study of dacomitinib (PF-00299804), an irreversible pan-human epidermal growth factor receptor inhibitor, versus erlotinib in patients with advanced non-small-cell lung cancer. Journal of Clinical Oncology. 2012;30(27):3337-44.
- Boyer MJ, Blackhall FH, Park K, Barrios CH, Krzakowski MJ, Taylor I, et al. Efficacy and safety of PF299804 versus erlotinib (E): A global, randomized phase II trial in patients (pts) with advanced non- small cell lung cancer (NSCLC) after failure of chemotherapy (CT). Journal of Clinical Oncology. 2010;28(18).
- Ramalingam S, Boyer M, Park K, Barrios C, Krzakowski M, Taylor I, et al. Randomized Phase 2 study of PF299804, an irreversible human epidermal growth factor receptor (EGFR) inhibitor, versus Erlotinib in patients with advanced non-small cell lung cancer (NSCLC) after chemotherapy failure: quantitative and qualitative benefits. Annals of Oncology. 2010;21(18): viii122–viii61.
- Boyer M, Blackhall F, Barrios C, Frank R, Heo D, Park K, et al. Overall Survival (OS) Results of a Randomized Phase 2 Trial of PF299804 versus Erlotinib in Patients with Advanced Non-Small Cell Lung Cancer (NSCLC) After Failure of Chemotherapy. Journal of Thoracic Oncology. 2011;6(6): O10.07.

Ramlau 2006

- Ramlau R, Gervais R, Krzakowski M, Von Pawel J, Kaukel E, Abratt RP, et al. Phase III study comparing oral topotecan to intravenous docetaxel in patients with pretreated advanced non-small-cell lung cancer. Journal of Clinical Oncology. 2006;24(18):2800 - 7.
- GSK report 09/07/2009 : http://www.gsk-clinicalstudyregister.com/files2/20023.pdf

Ramlau 2012

- Ramlau R. A Multinational, Randomized, Double-Blind Study Comparing Aflibercept Versus Placebo in Patients Treated with Second-Line Docetaxel After Failure of One Platinum Based Therapy for Locally Advanced or Metastatic Non-Small-Cell Lung Cancer. Clinicaltrialsgov. August 2012: NCT 00532155.
- Ramlau R, Gorbunova V, Ciuleanu TE, Novello S, Ozguroglu M, Goksel T, et al. Aflibercept and docetaxel versus docetaxel alone after platinum failure in patients with advanced or metastatic non- small-cell lung cancer: A randomized, controlled phase III trial. Journal of Clinical Oncology. 2012;30(29):3640 - 7.

REVEL 2014

- FDA report 04/2015: http://www.accessdata.fda.gov/drugsatfda_docs/label/2015/125477s011lbl.pdf
- Garon E. A Randomized, Double-Blind, Phase 3 Study of Docetaxel and Ramucirumab Versus Docetaxel and Placebo in the Treatment of Stage IV Non-Small Cell Lung Cancer Following Disease Progression After One Prior Platinum-Based Therapy. Clinicaltrialsgov. December 2014: NCT01168973.
- Garon EB, Ciuleanu TE, Arrieta O, Prabhash K, Syrigos KN, Goksel T, et al. Ramucirumab plus docetaxel versus placebo plus docetaxel for second-line treatment of stage IV non-small-cell lung cancer after disease progression on platinum-based therapy (REVEL): A multicentre, double-blind, randomised phase 3 trial. The Lancet. 2014;384(9944):665 - 73.
- NICE report 2016 : https://www.nice.org.uk/guidance/ta403/resources/ramucirumab-for-previously-treated-locally-advanced-or-metastatic-nonsmallcell-lung-cancer-pdf-82604541080005
- Reck M, Paz-Ares LG, Bidoli P, Cappuzzo F, Dakhil SR, Moro-Sibilot D, et al. Exploratory subgroup analysis of patients (Pts) refractory to first-line (1L) chemotherapy from REVEL, a randomized phase III study of docetaxel (DOC) with ramucirumab (RAM) or placebo (PBO) for second-line (2L) treatment of stage IV non-small-cell lung cancer (NSCLC). Journal of Clinical Oncology. 2016;34(Supplement): abstract 9079.
- Schuette W, Reck M, Kimmich M, Schumann C, Paz-Ares L, Garon E, et al. Exploratory analysis of efficacy by histology and frontline therapies in a nonsquamous non-small cell lung cancer (NSCLC) subgroup in REVEL: A randomized phase III study of ramucirumab (RAM) plus docetaxel (DOC) vs DOC plus placebo (PBO) for second-line treatment of stage IV NSCLC. Oncology Research and Treatment. 2016;39:90.
- Kang JH, Park K, Kim J-H, Cho EK, Shih JY, Zimmermann AH, et al. Subgroup analysis of east asian patients in the phase III revel trial. Journal of Thoracic Oncology. 2015;10(9):S323.
- Perol M, Ciuleanu TE, Arrieta O, Prabhash K, Syrigos KN, Goksel T, et al. REVEL: A randomized, double-blind, phase III study of docetaxel (DOC) and ramucirumab (RAM; IMC-1121B) versus DOC and placebo (PL) in the second-line treatment of stage IV non-small cell lung cancer (NSCLC) following disease progression after one prior platinum-based therapy. Journal of Clinical Oncology. 2014;32(15).
- Garon E, Ciuleanu T, Arrieta O, Prabhash K, Syrigos K, Göksel T, et al. Quality of life results from the Phase 3 REVEL study of Ramucirumab + Docetaxel versus Placebo + Docetaxel in advanced/metastatic NSCLC patients with progression after platinum based chemotherapy. Annals of Oncology. 2014;25(4): iv426–iv70.
- Paz-Ares L, Perol M, Ciuleanu T, Kowalyszyn R, Reck M, Lewanski C, et al. Exploratory analysis of safety by histology and efficacy in a nonsquamous NSCLC subgroup in REVEL: A randomized phase III study of ramucirumab (RAM) plus docetaxel (DOC) vs DOC for second-line treatment of stage IV non-small-cell lung cancer (NSCLC). Journal of Clinical Oncology. 2015; 33(15):8055.
- Reck M, Smit E, Garon E, Cappuzzo F, Bidoli P, Cohen R, et al. Exposure-response relationship for ramucirumab (RAM) from the randomized, double-blind, phase 3 REVEL trial (docetaxel [DOC] plus placebo [PL] vs DOC plus RAM) in second-line treatment of metastatic non-small cell lung cancer (NSCLC). Oncology Research and Treatment 2016;39: Supplement 1
- Smit E, Perol M, Reck M, Cappuzzo F, Bidoli P, Cohen R, et al. Exposure-response relationship for ramucirumab (RAM) from the randomized, double-blind, phase III REVEL trial (docetaxel [DOC] vs DOC plus RAM) in second-line treatment of metastatic non-small cell lung cancer (NSCLC). Journal of Clinical Oncology. 2015;33(15): Supplement 1.

- Bidoli P, Cappuzzo F, Favaretto A, Alabiso O, Tiseo M, Chella A, et al. Update of REVEL: A randomized, double-blind, phase III study of docetaxel (DOC) and ramucirumab (RAM; IMC-1121B) versus DOC and placebo (PL) in the second-line (2L) treatment of stage IV non-small cell lung cancer (NSCLC) including subgroup analysis of histology. Annals of Oncology. 2015;26: Supplement 6
- Garon E, Scagliotti G, Gautschi O, Reck M, Thomas M, Docampo L, et al. Exploratory analysis of frontline therapies in REVEL: A randomized phase III study of ramucirumab (RAM) plus docetaxel (DOC) versus DOC for the treatment of stage IV non-small-cell lung cancer (NSCLC) after disease progression on platinum-based therapy. European Journal of Cancer. 2015;51: Supplement 3 (3073). Sbar 2010
- Sbar E, Besse B, Felip E, Shaw A, Ahn M, Salvati M, et al. A double-blind, randomized, parallel, two- arm phase II trial of BMS-690514 versus erlotinib in previous chemotherapy-treated non-small cell lung cancer (NSCLC) patients: A safety review. Journal of Thoracic Oncology. 2010;5(12): S538 - S9.

Scagliotti 2012

- Scagliotti G. A Multicenter, Randomized, Double-Blind, Controlled Phase 3, Efficacy and Safety Study of Sunitinib (SU011248) In Patients with Advanced/Metastatic Non-Small Cell Lung Cancer Treated with Erlotinib. Clinicaltrialsgov. July 2011: NCT00457392.
- EUDRA 2014 report: https://www.clinicaltrialsregister.eu/ctr-search/trial/2007-001915-52/results
- Scagliotti GV, Krzakowski M, Szczesna A, Strausz J, Makhson A, Reck M, et al. Sunitinib plus erlotinib versus placebo plus erlotinib in patients with previously treated advanced non-small-cell lung cancer: A phase III trial. Journal of Clinical Oncology. 2012;30(17):2070 - 8.
- Thongprasert S, Tung Y, Kim JH, Chang GC, Park K, Su WC, et al. Sunitinib plus erlotinib for the treatment of advanced NSCLC: Subset analysis of east asian patients participating in a phase III trial. Journal of Thoracic Oncology. 2011;6(6): S551 - S2.
- Scagliotti GV, Krzakowski M, Szczesna A, Strausz J, Makhson A, Reck M, et al. Sunitinib (SU) in combination with erlotinib (E) for the treatment of advanced/metastatic nonsmall cell lung cancer (NSCLC): A phase III study. Annals of Oncology. 2010;21: viii3 - viii4.

Scagliotti 2014

- Scagliotti G. Randomized, Open Label, Phase 3 Trial of Erlotinib Alone Or In Combination With CP- 751,871 In Patients With Advanced Non6Small Cell Lung Cancer Of Non Adenocarcinoma Histology. Clinicaltrialsgov. April 2013: NCT00673049.
- Scagliotti GV, Bondarenko I, Blackhall F, Barlesi F, Hsia TC, Jassem J, et al. Randomized, phase III trial of figitumumab in combination with erlotinib versus erlotinib alone in patients with nonadenocarcinoma nonsmall-cell lung cancer. Annals of Oncology. 2014.

Schiller 2010

- Schiller JH, Von Pawel J, Schutt P, Ansari RH, Thomas M, Saleh M, et al. Pemetrexed with or without matuzumab as second-line treatment for patients with stage IIIB/IV non-small cell lung cancer. Journal of Thoracic Oncology. 2010;5(12):1977 - 85.

SELECT 1, 2017

- Janne PA, van den Heuvel MM, Barlesi F, Cobo M, Mazieres J, Crino L, et al. Selumetinib Plus Docetaxel Compared With Docetaxel Alone and Progression-Free Survival in Patients With KRAS-Mutant Advanced Non-Small Cell Lung Cancer: The SELECT-1 Randomized Clinical Trial. Jama. 2017;317(18):1844-53.
- Jänne PA, Van Den Heuvel MM, Barlesi F, Cobo M, Mazieres J, Crinò L, et al. Impact of PD-L1 status on clinical response in SELECT-1: Selumetinib + docetaxel in KRASm advanced NSCLC. Journal of Thoracic Oncology. 2017;12(1): S952-S3.
- Janne PA, van den Heuvel MM, Barlesi F, Cobo M, Mazieres J, Crino L, et al. Selumetinib in combination with docetaxel as second-line treatment for patients with KRAS-mutant advanced NSCLC: results from the phase III SELECT-1 trial. Annals of Oncology. 2016;27(Supplement 6): vi552-vi87.

SIGN 2006

- Cufer T, Vrdoljak E, Gaafar R, Erensoy I, Pemberton K. Phase II, open-label, randomized study (SIGN) of single-agent gefitinib (IRESSA) or docetaxel as second-line therapy in patients with advanced (stage IIIb or IV) non-small-cell lung cancer. Anti-Cancer Drugs. 2006;17(4):401 - 9.

Spigel 2011

- Spigel D. A Randomized Double-Blind Placebo-Controlled Phase II Trial of Sorafenib and Erlotinib or Erlotinib Alone in Previously Treated Advanced Non-Small Cell Lung Cancer. Clinicaltrialsgov. August 2012:

NCT00600015.
- Spigel DR, Burris HA, 3rd, Greco FA, Shipley DL, Friedman EK, Waterhouse DM, et al. Randomized, double-blind, placebo-controlled, phase II trial of sorafenib and erlotinib or erlotinib alone in previously treated advanced non-small-cell lung cancer. Journal of Clinical Oncology. 2011;29(18):2582-9.
- Waterhouse D, Stults D, Daniel D, Griner P, Greco F, Burris H, et al. KRAS subset analysis from randomized phase II trials of erlotinib versus erlotinib plus sorafenib or pazopanib in refractory non- small cell lung cancer (NSCLC). Journal of Clinical Oncology. 2013;31(15):8091.

Spigel 2012
- Spigel DR. Randomized, Double-Blind Trial of Erlotinib/Pazopanib or Erlotinib/Placebo in Patients with Previously Treated Advanced Non-Small-Cell Lung Cancer. Clinicaltrialsgov. October 2015: NCT01027598.
- Spigel D, Burris HA, Greco FA, Shih KC, Lipman AJ, Flora DB, et al. A randomized phase II study of pazopanib or placebo in combination with erlotinib in patients with advanced non-small-cell lung cancer. Journal of Thoracic Oncology. 2012;7(9): S208.

Spigel 2013
- Spigel D. A Randomized, Phase II, Multicenter, Double-Blind, Placebo-Controlled Study Evaluating the Safety and Activity of MetMAb, a Monovalent Antagonist Antibody to the Receptor Met, Administered to Patients with Advanced Non-Small Cell Lung Cancer, in Combination with Tarceva (Erlotinib). Clinicaltrialsgov. September 2011: NCT00854308.
- Spigel DR, Ervin TJ, Ramlau RA, Daniel DB, Goldschmidt Jr JH, Blumenschein Jr GR, et al. Randomized phase II trial of Onartuzumab in combination with erlotinib in patients with advanced non-small-cell lung cancer. Journal of clinical oncology. 2013;31(32):4105 - 14.

Sun 2013
- Sun Y. Phase 3 Study of Pemetrexed versus Docetaxel in Patients with Locally Advanced or Metastatic Non-Small Cell Lung Cancer Who Have Had Prior Chemotherapy. Clinicaltrialsgov. December 2009: NCT00391274.
- Sun Y, Wu YL, Zhou CC, Zhang L, Liu XY, Yu SY, et al. Second-line pemetrexed versus docetaxel in Chinese patients with locally advanced or metastatic non-small cell lung cancer: A randomized, open- label study. Lung Cancer. 2013;79(2):143 - 50.

TAILOR 2013
- Garassino MC, Martelli O, Broggini M, Farina G, Veronese S, Rulli E, et al. Erlotinib versus docetaxel as second-line treatment of patients with advanced non-small-cell lung cancer and wild-type EGFR tumours (TAILOR): A randomised controlled trial. The Lancet Oncology. 2013;14(10):981.
- Rulli E, Marabese M, Torri V, Farina G, Veronese S, Bettini A, et al. Value of KRAS as prognostic or predictive marker in NSCLC: results from the TAILOR trial. Ann Oncol. 2015 Oct;26(10):2079-84.

TALISMAN 2015
- Gridelli C. A Study of Erlotinib [Tarceva] as Monotherapy or Intermittent Dosing With Docetaxel in Patients With Advanced or Metastatic Non-Small Cell Lung Cancer (TALISMAN). Clinicaltrialsgov. October 2015: NCT01204697.
- Gridelli C, Chella A, Valmadre G, Allegrini G, Brighenti M, Bidoli P, et al. Second-line Erlotinib or Intermittent Erlotinib plus Docetaxel in Male Ex-smokers with Squamous NSCLC: The TALISMAN Randomized Trial. Anticancer research. 2016;36: 6535-40.

TAX 317 2000
- FDA report 2015: http://www.accessdata.fda.gov/drugsatfda_docs/label/2015/020449s075lbl.pdf
- Shepherd FA, Dancey J, Ramlau R, Mattson K, Gralla R, O'Rourke M, et al. Prospective randomized trial of docetaxel versus best supportive care in patients with non-small-cell lung cancer previously treated with platinum- based chemotherapy. Journal of Clinical Oncology. 2000;18(10):2095 - 103.
- Dancey J, Shepherd FA, Gralla RJ, Kim YS. Quality of life assessment of second-line docetaxel versus best supportive care in patients with non-small-cell lung cancer previously treated with platinum- based chemotherapy: Results of a prospective, randomized phase III trial. Lung Cancer. 2004;43(2):183-94.

TITAN, 2012*
- Ciuleanu T. A Study of Tarceva (Erlotinib) and Standard of Care Chemotherapy in Patients with Advanced, Recurrent, or Metastatic Non-Small Cell Lung Cancer(NSCLC). Clinicaltrialsgov. December 2014: NCT00556322.

- Ciuleanu T, Stelmakh L, Cicenas S, Miliauskas S, Grigorescu AC, Hillenbach C, et al. Efficacy and safety of erlotinib versus chemotherapy in second-line treatment of patients with advanced, non-small-cell lung cancer with poor prognosis (TITAN): A randomised multicentre, open-label, phase 3 study. The Lancet Oncology 2012;13(3):300-08.
- Ciuleanu T, Stelmakh L, Cicens S, Gonzlez EE. Efficacy and safety of erlotinib verus chemotherapy in second-line advanced non-small-cell lung cancer (NSCLC) with poor prognosis: The phase III titan study. Lung Cancer 2011;71: S44.

TORI L-03 2013

- Garon EB, Dubinett SM, Kabbinavar FF, Reckamp KL, Marquez-Garban DC, Goodglick L, et al. Randomized, multicenter phase II study of erlotinib (E) or E plus fulvestrant (F) in previously treated advanced non-small cell lung cancer (NSCLC). Journal of Clinical Oncology. 2011;29(15).
- Garon EB, Siegfried JM, Dubinett SM, Elashoff RM, Park DJ, Parikh RJ, et al. Result of TORI L-03, a randomized, multicenter phase II clinical trial of erlotinib (E) or E + fulvestrant (F) in previously treated advanced non-small cell lung cancer (NSCLC). Cancer Research. 2013;73(8).

ULTIMATE IFCT-1103, 2016

- Cortot AB, Audigier-Valette C, Molinier O, Le Moulec S, Barlesi F, Zalcman G, et al. Weekly paclitaxel plus bevacizumab versus docetaxel as second or third-line treatment in advanced non-squamous non-small cell lung cancer (NSCLC): Results from the phase III study IFCT-1103 ULTIMATE. Journal of Clinical Oncology. 2016;34(Supplement): abstract 9005.
- Cortot AB, Audigier Valette C, Molinier O, Le Moulec S, Barlesi F, Zalcman G, et al. Prolonged OS of Patients Exposed to Weekly Paclitaxel and Bevacizumab: Impact of the Cross-Over in the IFCT-1103 ULTIMATE Study. Journal of Thoracic Oncology 2017;12(1): OA11.01.

V15-32 2008

- Maruyama R, Nishiwaki Y, Tamura T, Yamamoto N, Tsuboi M, Nakagawa K, et al. Phase III study, V- 15-32, of gefitinib versus docetaxel in previously treated Japanese patients with non-small-cell lung cancer. Journal of Clinical Oncology. 2008;26(26):4244 - 52.
- Sekine I, Ichinose Y, Nishiwaki Y, Yamamoto N, Tsuboi M, Nakagawa K, et al. Quality of life and disease-related symptoms in previously treated Japanese patients with non-small-cell lung cancer: Results of a randomized phase III study (V-15-32) of gefitinib versus docetaxel. Annals of Oncology. 2009;20(9):1483 - 8.

Wen 2016

- Wen S, Fu X, Li G, He L, Zhao C, Hu X, et al. Efficacy of tamoxifen in combination with docetaxel in patients with advanced non-small-cell lung cancer pretreated with platinum-based chemotherapy. Anticancer Drugs. 2016 2016/06//;27(5):447-56.

Witta 2012

- Witta SE, Jotte RM, Konduri K, Neubauer MA, Spira AI, Ruxer RL, et al. Randomized phase II trial of erlotinib with and without entinostat in patients with advanced non-small-cell lung cancer who progressed on prior chemotherapy. Journal of Clinical Oncology. 2012;30(18):2248-55.

WJOG5108L 2014

- Urata Y, Katakami N, Morita S, Kaji R, Yoshioka H, Seto T, et al. Randomized Phase III Study Comparing Gefitinib with Erlotinib in Patients with Previously Treated Advanced Lung Adenocarcinoma: WJOG 5108L. Journal of Clinical Oncology. 2016; 34(27):3248-57.
- Katakami N, Morita S, Yoshioka H, Seto T, Urata Y, Satouchi M, et al. Randomized phase III study comparing gefitinib (G) with erlotinib (E) in patients (pts) with previously treated advanced lung adenocarcinoma (LA): WJOG 5108L. Journal of Clinical Oncology. 2014;32(15).
- Nishiyama A, Katakami N, Morita S, Seto T, Iwamoto Y, Hirashima T, et al. Randomized Phase III study comparing Gefitinib with Erlotinib in patients with previously treated advanced lung adenocarcinoma: WJOG5108L. Annals of Oncology. 2014;25(4): iv426–iv70.

ZEAL 2011

- De Boer R. A Phase III, Randomized, Double-blinded, Parallel Group, Multi-centre Study to Assess the Efficacy and Safety of ZD6474 (ZACTIMA™) in Combination with Pemetrexed (Alimta®) Versus Pemetrexed Alone in Patients with Locally-Advanced or Metastatic NSCLC. Clinicaltrialsgov. April 2011: NCT00418886.

- De Boer RH, Arrieta O, Yang CH, Gottfried M, Chan V, Raats J, et al. Vandetanib plus pemetrexed for the second-line treatment of advanced non - small-cell lung cancer: A randomized, double-blind phase III trial. Journal of Clinical Oncology. 2011;29(8):1067 - 74.
- De Boer R, Arrieta O, Gottfried M, Blackhall FH, Raats J, Yang CH, et al. Vandetanib plus pemetrexed versus pemetrexed as second-line therapy in patients with advanced non-small cell lung cancer (NSCLC): A randomized, double-blind phase III trial (ZEAL). Journal of Clinical Oncology. 2009;27(15):8010.

ZEST 2011

- Natale R. A Phase III, International, Randomised, Double Blind, Parallel-Group Study to Assess the Efficacy of Zactima™ Versus Tarceva® in Patients with Locally Advanced or Metastatic Non-Small Cell Lung Cancer After Failure of at Least One Prior Chemotherapy. Clinicaltrialsgov. April 2011: NCT00364351.
- Natale RB, Thongprasert S, Greco FA, Thomas M, Tsai CM, Sunpaweravong P, et al. Phase III trial of vandetanib compared with erlotinib in patients with previously treated advanced non-small-cell lung cancer. Journal of Clinical Oncology. 2011;29(8): 1059-66.
- Natale RB, Thongprasert S, Greco FA, Thomas M, Tsai CM, Sunpaweravong P, et al. Vandetanib versus erlotinib in patients with advanced non-small cell lung cancer (NSCLC) after failure of at least one prior cytotoxic chemotherapy: A randomized, double-blind phase III trial (ZEST). Journal of Clinical Oncology. 2009;27(15):8009.

Zhang 2015

- Zhang Y, Gao C, Qu W, Gao Y, Zhu S, Zhang S, et al. A Randomized Phase II Study of Erlotinib Plus Nab-Paclitaxel Versus Erlotinib Alone as Second-Line Therapy for Chinese Patients with Advanced EGFR Wild-Type Non-Small-Cell Lung Cancer. Cancer Investigation. 2015; Early Online:1– 5:1532-4192 online.

ZODIAC 2010

- Herbst R. A Phase III, Randomized, Double-Blinded, Multi-Center, Study to Assess the Efficacy of Docetaxel (TAXOTERE™) in Combination with ZD6474 (ZACTIMA™) Versus Docetaxel (TAXOTERE™) With Placebo in Subjects with Locally Advanced or Metastatic NSCLC. Clinicaltrialsgov. April 2011: NCT00312377.
- Herbst RS, Sun Y, Eberhardt WEE, Germonpre P, Saijo N, Zhou C, et al. Vandetanib plus docetaxel versus docetaxel as second-line treatment for patients with advanced non-small-cell lung cancer (ZODIAC): A double-blind, randomised, phase 3 trial. The Lancet Oncology. 2010;11(7):619 - 26.
- Herbst RS, Sun Y, Korfee S, Germonpre P, Saijo N, Zhou C, et al. Vandetanib plus docetaxel versus docetaxel as second-line treatment for patients with advanced non-small cell lung cancer (NSCLC): A randomized, double-blind phase III trial (ZODIAC). Journal of Clinical Oncology. 2009;27(18): CRA8003.
- Heymach J, Lockwood S, Herbst R, Johnson B, Ryan A. EGFR biomarkers predict benefit from vandetanib in combination with docetaxel in a randomized phase III study of second-line treatment of patients with advanced non-small cell lung cancer. Annals of Oncology. 2014;25: 1941–8.
- Johnson B, Ryan A, J. H, Stephens C, Kennedy S, Langmuir P, et al. Tumor biomarker analyses from the phase III ZODIAC study of docetaxel (D) plus or minus vandetanib (VAN) in second-line advanced NSCLC. Journal of Clinical Oncology. 2010;28(15):7516.

*Trials in which patients in the control arm received chemotherapy (e.g., docetaxel or pemetrexed) at the investigators' discretion were included for the secondary analysis considering treatments categories

Appendix 8: Characteristics of the 102 individual trials

Trials	Treatment	Sample size	Age	Male	Asian	Caucasian	Stage IV	Squamous	PS2	Smokers	Second line	EGFR-WT or unknown[¤]
ARCHER 1009, 2014	Erlotinib	439	62	277	88	331	403	128	46	357	281	395
ARCHER 1009, 2014	Dacomitinib	439	64	288	91	335	400	115	42	360	256	392
ARQ 197-209, 2011	Erlotinib	83	62	49			72	24	0	65	51	72
ARQ 197-209, 2011	Erlotinib+Tivantinib	84	64	51			76	31	1	67	50	78
ATTENTION, 2015	Erlotinib+Tivantinib	154	63	109	154	0	118	0	0	113	92	154
ATTENTION, 2015	Erlotinib	153	63	102	153	0	115	0	0	114	90	153
Bergqvist, 2014	Docetaxel	41	58	31			40	20	1			41
Bergqvist, 2014	AXL1717	58	57	40			52	29	3			58
Besse, 2014	Erlotinib+Everolimus	66	60	36	7	54	64	10	0	61	51	66
Besse, 2014	Erlotinib	67	60	33	2	57	63	10	0	64	41	67
BeTa, 2011	Erlotinib	317	65	170	18	257		17	20	284	317	299
BeTa, 2011	Erlotinib+Bevacizumab	319	65	171	23	264		11	23	285	319	307
Bhatnagar, 2012	Docetaxel	15	57	11	15	0	11	8	2	12	15	15
Bhatnagar, 2012	Gefitinib	15	58	12	15	0	12	9	3	12	15	15
Blumenschein, 2015	Docetaxel	45	61	23	10	35		1	1	38	45	KRAS
Blumenschein, 2015	Trametinib	89	61	46	11	74		0	0	83	89	KRAS
BR21, 2005	Placebo	243	59	160	28	188		78	56	201	121	175
BR21, 2005	Erlotinib	488	62	315	63	379		144	126	384	243	371
CALGB 30704, 2014	Pemetrexed+Sunitinib	41	63	22	1	32	38	6	0		39	41
CALGB 30704, 2014	Pemetrexed	42	63	22	0	36	37	4	0		38	42
CALGB 30704, 2014	Sunitinib	47	63	25	1	43	39	7	0		43	47
CheckMate017, 2015	Nivolumab	135	62	111	4	122	105	135	0	125	135	135
CheckMate017, 2015	Docetaxel	137	64	97	2	130	112	137	0	130	137	137
CheckMate057, 2015	Docetaxel	290	62	168	8	266		0	0	230	259	252
CheckMate057, 2015	Nivolumab	292	61	151	9	267		0	0	234	256	256
Chen, 2011	Tegafur/uracil+Gefitinib	57	65	37	57	0		0	11	25	25	37
Chen, 2011	Gefitinib	58	64	33	58	0		0	24	28	31	42

CTONG0806, 2014	Pemetrexed	80	56	47	80	0	66	4	0	36	80	80
CTONG0806, 2014	Gefitinib	81	58	54	81	0	77	2	0	48	81	81
Dai, 2013	Gefitinib	23	62	15	23	0	19	0	6	13	23	23
Dai, 2013	Pemetrexed	23	61	14	23	0	18	0	5	12	23	23
DATE, 2015	Gefitinib+Nimotuzumab	78	63	54				27	3		78	66
DATE, 2015	Gefitinib	77	63	48				27	0		77	62
DELTA, 2014	Erlotinib	150	68	108	150	0	120	29	6	111	121	150
DELTA, 2014	Docetaxel	151	67	107	151	0	122	32	6	114	130	151
Dittrich, 2014	Pemetrexed+Erlotinib	79	64	46	1	75	64	0	9	69	79	79
Dittrich, 2014	Pemetrexed	83	60	49	1	82	70	0	11	69	83	83
E1512, 2015	Cabozantinib	40	66	14	0	33	24	0	4	34	23	38
E1512, 2015	Erlotinib	42	66	18	2	32	29	0	5	37	23	38
E1512, 2015	Erlotinib+Cabozantinib	43	64	18	0	31	25	0	4	37	21	35
Esteban, 2003	Docetaxel	35	55	29			35	13	12		27	35
Esteban, 2003	Paclitaxel	36	62	30			36	10	13		25	36
Gerber, 2014*	Erlotinib+Tivantinib	51	64	17							22	51
Gerber, 2014*	Chemotherapy	45	64	15							20	45
GFPC 05-06, 2011	Docetaxel	75	59	64			59	23	5		78	75
GFPC 05-06, 2011	Pemetrexed	75	58	62			62	18	4		75	75
Groen, 2013	Erlotinib+Sunitinib	65	59	39	1	63	63	15	0	58	39	61
Groen, 2013	Erlotinib	67	61	45	2	64	67	19	1	58	46	66
Hainsworth, 2010	Selumetinib	40	62	26	0	39		25		34	31	40
Hainsworth, 2010	Pemetrexed	44	64	27	1	42		24		32	35	44
Han, 2011	Docetaxel	40	50	22	40	0		16	4		40	40
Han, 2011	Pemetrexed	44	50	26	44	0		18	6		44	44
HANSHIN, 2015	Docetaxel+Bevacizumab	45	64	27	45	0	36	0	0	34	45	30
HANSHIN, 2015	S1+Bevacizumab	45	64	27	45	0	37	0	0	31	45	27
Herbst, 2007*	Erlotinib+Bevacizumab	39	68	17	3	29		0	0	33	39	38
Herbst, 2007*	Chemotherapy	41	65	25	1	31		0	1	35	41	41
Herbst, 2007*	Chemotherapy+Bevacizumab	40	64	23	2	34		0	0	36	40	40
Heymach, 2007	Docetaxel+Vandetanib 100	42	61	21	0	42	28	12	0	35	42	42
Heymach, 2007	Docetaxel	41	58	27	0	41	28	11	0	37	41	41

Heymach, 2007	Docetaxel+Vandetanib 300	44	60	25	0	44	35	14	0	40	44	44
HORG, 2013	Pemetrexed	178	66	138	0	178	147	36	31	154	101	178
HORG, 2013	Erlotinib	179	65	135	0	179	154	39	18	150	89	179
Hosomi, 2015	Docetaxel+Ramucirumab	79	66	59	76	0		9	0	65	76	76
Hosomi, 2015	Docetaxel	81	65	62	81	0		9	0	67	81	81
ICOGEN, 2013	Gefitinib	199	56	114	199	0	162	36	21	97	107	160
ICOGEN, 2013	Icotinib	200	56	118	200	0	162	34	26	99	126	171
INTEREST, 2008	Gefitinib	733	61	466	154	550		185	86	585	619	711
INTEREST, 2008	Docetaxel	733	60	488	169	540		176	84	583	610	711
ISEL, 2005	Placebo	563	61	378	107	431	450	187	145	438	274	563
ISEL, 2005	Gefitinib	1129	62	761	235	843	896	399	332	879	549	1129
ISTANA, 2010	Docetaxel	79	58	45	79	0	65	11	5	43	79	79
ISTANA, 2010	Gefitinib	82	57	55	82	0	71	17	6	52	82	82
Janne, 2013	Docetaxel	43	59	20	0	40	42	6	0	38	43	KRAS
Janne, 2013	Docetaxel+Selumetinib	44	60	21	0	41	39	3	0	39	44	KRAS
JMEI, 2004	Pemetrexed	283	59	194	64	207	212	78	30		283	283
JMEI, 2004	Docetaxel	288	57	217	72	206	215	93	34		288	288
Jones, 2008	Topotecan	39	63	23					1		39	39
Jones, 2008	Docetaxel	41	62	23					6		41	41
Juan, 2014	Docetaxel+Erlotinib	34	58	31	0	34	30	16	5	32	33	33
Juan, 2014	Erlotinib	36	64	29	0	36	29	13	6	34	35	34
Kapoor, 2015	Gefitinib	54	56	43	54	0	40		17			20
Kapoor, 2015	Docetaxel	53	56	42	53	0	39		17			17
Katakami, 2014	Amrubicin	101	64	66	98	0	81	17	0	75	78	80
Katakami, 2014	Docetaxel	101	64	69	99	0	74	18	0	75	80	82
KCSG-LU08-01, 2012	Pemetrexed	70	64	10	70	0	61	0	6	0	70	46
KCSG-LU08-01, 2012	Gefitinib	71	58	10	71	0	62	0	6	0	71	49
Kelly, 2012	Pralatrexate	100	63	69		74	87	29	0	100		100
Kelly, 2012	Erlotinib	101	62	68		78	84	47	0	101		101
KEYNOTE-010,2015	Pembrolizumab 2	344	63	212	73	246		76	3	281	243	316
KEYNOTE-010,2015	Pembrolizumab 10	346	63	213	72	250		80	1	286	235	320
KEYNOTE-010,2015	Docetaxel	343	62	209	72	251		66	2	276	235	317

Kim A, 2012	Pemetrexed+Cetuximab	301	63	173	3	268		76	12		301	301
Kim A, 2012	Pemetrexed	304	64	188	6	265		71	14		304	304
Kim B, 2012	Docetaxel	166	64	93	2	141		51	11		166	166
Kim B, 2012	Docetaxel+Cetuximab	167	64	92	5	146		41	2		167	167
Kim, 2015	Pemetrexed	47	64	33	47	0	44	10	15	33	32	46
Kim, 2015	Gefitinib	48	67	35	48	0	46	9	17	33	30	47
Krzakowski, 2010	Vinflunine	274	62	205			247	100			274	274
Krzakowski, 2010	Docetaxel	277	60	208			248	95			277	277
Kuo, 2013	Docetaxel	6	54	3	6	0		0	1		6	6
Kuo, 2013	Gefitinib	8	67	4	8	0		0	0		8	8
Lee, 2013	Pemetrexed+Erlotinib	78	56	20	58	20	72	0	7	0	78	78
Lee, 2013	Pemetrexed	80	56	35	68	12	68	0	4	0	80	80
Lee, 2013	Erlotinib	82	54	28	76	6	68	0	6	0	82	82
Levy, 2014	Docetaxel	47	60	24		34	45	12	0			47
Levy, 2014	Docetaxel+PX-866	48	65	33		39	45	8	0			48
Li, 2010	Docetaxel	48	48	29	48	0	19	27			48	48
Li, 2010	Gefitinib	50	51	30	50	0	21	22			50	50
Li, 2012	Docetaxel	128	56	74	102	0	61	25			102	128
Li, 2012	Pemetrexed	132	58	67	106	0	70	19			106	132
Li, 2013	Pemetrexed	27	62	14	0	18		0	3	22	27	27
Li, 2013	Pemetrexed+Erlotinib	52	64	23	2	36		0	5	35	52	45
Li, 2014	Erlotinib	61	54	40	61	0	40	0	4	46	61	61
Li, 2014	Pemetrexed	62	55	39	62	0	38	0	3	45	62	62
Liu, 2015	Pemetrexed	56	51	34	56	0		32	11	55	56	56
Liu, 2015	Nab-Paclitaxel	55	52	37	55	0		27	15	55	55	55
LUME-COLUMBUS,2016	Docetaxel+Nintedanib	6	63	4								6
LUME-COLUMBUS,2016	Docetaxel	6	60	5								6
LUME-LUNG 1, 2014	Docetaxel+Nintedanib	655	60	476	116	533	588	276	1	490	655	655
LUME-LUNG 1, 2014	Docetaxel	659	60	479	123	530	605	279	0	498	659	659
LUME-LUNG 2, 2013	Pemetrexed+Nintedanib	353	60	195	103	225	307	3	0	244	353	336
LUME-LUNG 2, 2013	Pemetrexed	360	59	208	105	230	328	2	0	238	360	349
Lux-Lung 8, 2015	Erlotinib	397	64	331	86	311	345	382	1	379	397	397

Lux-Lung 8, 2015	Afatinib	398	65	335	86	312	349	381	3	372	398	398
Maitland, 2014	Cetuximab	20	62	14	0	13		6		18		20
Maitland, 2014	Pemetrexed+Cetuximab	23	55	13	1	17		8		21		23
Marangolo, 2000	Docetaxel	14	64					4			14	14
Marangolo, 2000	Vinorelbine	11	64					6			11	11
MARQUEE, 2013	Erlotinib	522	61	309	5	446	501	0	1	424	348	469
MARQUEE, 2013	Erlotinib+Tivantinib	526	62	310	8	430	499	0	1	425	346	470
MARVEL, 2013	Erlotinib	11	69	5							11	11
MARVEL, 2013	Pemetrexed	12	55	6							12	12
METLung, 2014	Erlotinib	249	63	140	37	180		31	3		157	220
METLung, 2014	Erlotinib+Onartuzumab	250	62	140	35	182		40	5		161	222
Moran, 2014	Erlotinib+Dalotuzumab	37	62	27	0	37	33	11	2	33	27	37
Moran, 2014	Erlotinib	38	59	28	2	36	29	6	1	27	20	38
Natale, 2009	Vandetanib	83	63	48			69	16	0	71		83
Natale, 2009	Gefitinib	85	61	52			63	22	0	77		85
NCCTG N0626, 2011	Pemetrexed+Sorafenib	47	62	27			39	0	0			47
NCCTG N0626, 2011	Pemetrexed	51	62	23			45	0	0			51
NCIC CTG BR26, 2014	Placebo	240	66	120	70	144	212	44	58	155	64	172
NCIC CTG BR26, 2014	Dacomitinib	480	64	244	141	288	443	63	119	306	133	366
NVALT-10, 2013*	Erlotinib	115	64	75			86	40	9	108	115	115
NVALT-10, 2013*	Erlotinib+Chemotherapy	116	63	73			94	34	9	107	116	116
OAK,2016	Atezolizumab	613	63	261	85	302		112	0	529	320	383
OAK,2016	Docetaxel	612	64	259	95	296		110	0	540	320	382
Paz-Ares, 2008	Docetaxel	422	63	302	5	381	343		62		422	422
Paz-Ares, 2008	Paclitaxel	427	62	308	5	397	342		57		427	427
POPLAR, 2016	Atezolizumab	144	62	93				49	2	117	93	133
POPLAR, 2016	Docetaxel	143	62	76				48	1	114	96	135
PROSE, 2014*	Chemotherapy	142	64	91			110	16	8	125	142	123
PROSE, 2014*	Erlotinib	143	66	99			121	31	8	122	143	126
Ramalingam, 2011	Erlotinib	57	62	37	0	55	46	12		48	43	54
Ramalingam, 2011	Erlotinib+R1507 3W	57	62	38	0	56	50	16		52	39	56
Ramalingam, 2011	Erlotinib+R1507 W	58	63	39	0	55	50	15		50	44	56

Ramalingam, 2012	Dacomitinib	94	60	55	23	68		32	19	76	51	75
Ramalingam, 2012	Erlotinib	94	62	56	24	67		33	3	75	63	83
Ramlau, 2006	Topotecan	414	59	311	31	368	308	155	58		414	414
Ramlau, 2006	Docetaxel	415	59	310	39	359	298	181	65		415	415
Ramlau, 2012	Docetaxel+Aflibercept	456	60	305	34	411	414	0	21		456	456
Ramlau, 2012	Docetaxel	457	60	300	40	405	410	0	23		457	457
REVEL, 2014	Docetaxel	625	61	415	86	503		171	0	484	625	607
REVEL, 2014	Docetaxel+Ramucirumab	628	62	419	74	526		157	0	519	628	613
Sbar, 2010	Erlotinib	70		50				17		61		70
Sbar, 2010	BMS-690514	71		50				18		61		71
Scagliotti, 2012	Erlotinib+Sunitinib	480	61	297	52	412	438	135	1	384	340	480
Scagliotti, 2012	Erlotinib	480	61	284	51	413	448	135	0	390	340	480
Scagliotti, 2014	Erlotinib	290	62	225	23	238	235	263	54	264		290
Scagliotti, 2014	Erlotinib+Figitumumab	293	62	228	21	249	230	263	56	277		293
Schiller, 2010	Pemetrexed+Matuzumab 1600	49	63	27	0	47	41	17	0		49	49
Schiller, 2010	Pemetrexed	50	61	33	0	47	44	18	0		50	50
Schiller, 2010	Pemetrexed+Matuzumab 800	51	62	35	0	47	42	11	0		51	51
SELECT 1,2017	Docetaxel	256	61	145		243	246	14	0	235	256	KRAS
SELECT 1,2017	Docetaxel+Selumetinib	254	62	158		241	239	14	0	238	254	KRAS
SIGN, 2006	Gefitinib	68	63	47	0	61	41		25	50	68	68
SIGN, 2006	Docetaxel	73	60	51	0	61	41		21	55	73	73
Spigel, 2011	Erlotinib	56	65	26	0	47		17	10	48	28	53
Spigel, 2011	Erlotinib+Sorafenib	112	65	62	0	97		33	13	93	73	110
Spigel, 2012	Erlotinib	67	67	39							43	67
Spigel, 2012	Erlotinib+Pazopanib	134	66	71							64	134
Spigel, 2013	Erlotinib	68	63	42	1	61		20	2	60	46	62
Spigel, 2013	Erlotinib+Onartuzumab	69	64	40	2	61		20	4	59	46	62
Sun, 2013	Docetaxel	104	56	61	104	0	84	25	11	55	104	104
Sun, 2013	Pemetrexed	107	57	73	107	0	81	27	12	57	107	107
TAILOR, 2013	Docetaxel	110	67	73	1	109	103	23	7	80	102	110
TAILOR, 2013	Erlotinib	112	66	77	1	108	103	31	9	93	100	109
TALISMAN, 2015	Docetaxel+Erlotinib	37	66	36				37	0	37	37	37

TALISMAN, 2015	Erlotinib	36	68	36				36	1	36	36	36
TAX 317, 2000	Placebo	49	61	32			40		12		37	49
TAX 317, 2000	Docetaxel	55	61	35			40		14		44	55
TITAN, 2012*	Chemotherapy	221	59	160	26	190	170	77	46	177	221	211
TITAN, 2012*	Erlotinib	203	59	161	28	172	162	77	39	173	203	195
TORI L-03, 2013	Erlotinib+Fulvestrant	50										50
TORI L-03, 2013	Erlotinib	50										50
ULTIMATE, 2016	Paclitaxel+Bevacizumab	111	60	78				11	8	102	76	111
ULTIMATE, 2016	Docetaxel	55	60	42				4	4	46	39	55
V15-32, 2008	Docetaxel	244		151	244	0	150	41	10	157	201	244
V15-32, 2008	Gefitinib	245		151	245	0	159	37	11	174	212	245
Wen, 2016	Docetaxel+Tamoxifen	60	58	44	60	0	27	32	0	35	60	60
Wen, 2016	Docetaxel	60	59	47	60	0	30	31	0	37	60	60
Witta, 2012	Entinostat+Erlotinib	67	66	39	1	55		18	8	56	66	64
Witta, 2012	Erlotinib	65	67	43	2	55		21	9	54	64	62
WJOG 5108L, 2016	Gefitinib	279	68	127	279	0	193	0	17	138	199	76
WJOG 5108L, 2016	Erlotinib	280	67	128	280	0	194	0	21	139	193	82
ZEAL, 2011	Pemetrexed+Vandetanib	256	59	159	28	197	219	54	18	199	217	256
ZEAL, 2011	Pemetrexed	278	60	171	34	218	232	60	13	224	240	278
ZEST, 2011	Erlotinib	617	61	393	207	388	519	143	77	472	391	617
ZEST, 2011	Vandetanib	623	60	381	202	401	517	129	65	493	404	623
Zhang, 2015	Erlotinib	43	55	24	43	0		6	2	31	43	43
Zhang, 2015	Nab-Paclitaxel+Erlotinib	45	55	23	45	0		7	3	35	45	45
ZODIAC, 2010	Docetaxel+Vandetanib	694	59	497	259	410	598	184	6	536	694	662
ZODIAC, 2010	Docetaxel	697	58	473	252	417	590	160	2	524	697	679

* Trials in which patients in the control arm received a chemotherapy (e.g., docetaxel or pemetrexed) at the investigators' discretion; ¤ during the trial, some patients have been detected for EGFR mutation so we provided the number of patients excluding patients identified EGFR positive

Appendix 9: Results of individual trials

Overall survival (OS) and progression-free survival (PFS)

Trials	Treatment (arm 1)	Treatment (arm 2)	OS, HR (95% CI)	PFS, HR (95% CI)
ARCHER 1009, 2014	Dacomitinib	Erlotinib	1.03 (0.89-1.19)	0.93 (0.80-1.10)
ARQ 197-209, 2011	Erlotinib+Tivantinib	Erlotinib	0.87 (0.59-1.27)	0.74 (0.51-1.06)
ATTENTION, 2015	Erlotinib+Tivantinib	Erlotinib	0.89 (0.67-1.18)	0.72 (0.54-0.95)
Bergqvist, 2014	AXL1717	Docetaxel	1.10 (0.80-1.50)	1.30 (0.83-2.02)
Besse, 2014	Erlotinib+Everolimus	Erlotinib	1.28 (0.69-2.37)	0.77 (0.51-1.17)
BeTa, 2011	Erlotinib+Bevacizumab	Erlotinib	0.97 (0.8-1.18)	0.62 (0.52-0.75)
Bhatnagar, 2012	Docetaxel	Gefitinib	-	-
Blumenschein, 2015	Trametinib	Docetaxel	0.97 (0.52-1.83)	1.23 (0.81-1.87)
BR21, 2005	Erlotinib	Placebo	0.73 (0.60-0.87)	0.61 (0.51-0.73)
CALGB 30704, 2014	Sunitinib	Pemetrexed	1.40 (0.90-2.30)	1.40 (0.90-2.20)
CALGB 30704, 2014	Pemetrexed+Sunitinib	Pemetrexed	2.00 (1.20-3.20)	1.30 (0.90-2.10)
CheckMate017, 2015	Nivolumab	Docetaxel	0.59 (0.44-0.79)	0.62 (0.47-0.81)
CheckMate057, 2015	Nivolumab	Docetaxel	0.73 (0.60-0.89)	0.92 (0.77-1.11)
Chen, 2011	Tegafur/uracil+Gefitinib	Gefitinib	0.82 (0.48-1.40)	0.65 (0.43-0.97)
CTONG0806, 2014	Pemetrexed	Gefitinib	0.72 (0.49-1.04)	0.53 (0.38-0.75)
Dai, 2013	Gefitinib	Pemetrexed	-	0.77 (0.42-1.43)
DATE, 2015	Gefitinib+Nimotuzumab	Gefitinib	0.86 (0.57-1.30)	1.03 (0.71-1.41)
DELTA, 2014	Erlotinib	Docetaxel	0.91 (0.68-1.22)	1.22 (0.97-1.55)
Dittrich, 2014	Pemetrexed+Erlotinib	Pemetrexed	0.68 (0.47-0.98)	0.63 (0.44-0.90)
E1512, 2015	Cabozantinib	Erlotinib	0.68 (0.33-1.03)	0.39 (0.17-0.61)
E1512, 2015	Erlotinib+Cabozantinib	Erlotinib	0.51 (0.22-0.80)	0.37 (0.15-0.58)
Esteban, 2003	Paclitaxel	Docetaxel	1.84 (1.12-3.02)	-
Gerber, 2014	Erlotinib+Tivantinib	Chemotherapy	1.07 (0.66-1.74)	1.19 (0.71-1.97)
GFPC 05-06, 2011	Pemetrexed	Docetaxel	0.85 (0.61-1.20)	0.93 (0.67-1.29)
Groen, 2013	Erlotinib+Sunitinib	Erlotinib	1.07 (0.71-1.61)	0.90 (0.46-1.76)
Hainsworth, 2010	Selumetinib	Pemetrexed	-	1.08 (0.62-1.86)
Han, 2011	Pemetrexed	Docetaxel	-	-

HANSHIN, 2015	Docetaxel+Bevacizumab	S1+Bevacizumab	1.25 (0.73-2.14)	0.84 (0.53-1.32)
Herbst, 2007	Chemotherapy+Bevacizumab	Chemotherapy	0.71 (0.41-1.21)	0.66 (0.38-1.16)
Herbst, 2007	Erlotinib+Bevacizumab	Chemotherapy	0.78 (0.46-1.31)	0.72 (0.42-1.23)
Heymach, 2007	Docetaxel+Vandetanib	Docetaxel	0.91 (0.55-1.52)	0.64 (0.38-1.05)
HORG, 2013	Pemetrexed	Erlotinib	0.99 (0.78-1.29)	-
Hosomi, 2015	Docetaxel+Ramucirumab	Docetaxel	0.86 (0.56-1.32)	0.83 (0.59-1.16)
ICOGEN, 2013	Icotinib	Gefitinib	1.02 (0.82-1.27)	0.84 (0.67-1.05)
INTEREST, 2008	Gefitinib	Docetaxel	1.02 (0.91-1.14)	1.04 (0.93-1.16)
ISEL, 2005	Gefitinib	Placebo	0.89 (0.77-1.02)	-
ISTANA, 2010	Gefitinib	Docetaxel	0.87 (0.61-1.24)	0.73 (0.50-1.06)
Janne, 2013	Docetaxel+Selumetinib	Docetaxel	0.81 (0.48-1.36)	0.58 (0.36-0.94)
JMEI, 2004	Pemetrexed	Docetaxel	0.99 (0.82-1.20)	0.97 (0.82-1.16)
Jones, 2008	Topotecan	Docetaxel	0.79 (0.50-1.25)	-
Juan, 2014	Docetaxel+Erlotinib	Erlotinib	0.70 (0.41-1.19)	0.65 (0.39-1.06)
Kapoor, 2015	Gefitinib	Docetaxel	0.89 (0.49-1.29)	1.12 (0.77-1.45)
Katakami, 2014	Amrubicin	Docetaxel	1.02 (0.72-1.43)	0.96 (0.69-1.34)
KCSG-LU08-01, 2012	Gefitinib	Pemetrexed	0.80 (0.50-1.30)	0.54 (0.37-0.79)
Kelly, 2012	Pralatrexate	Erlotinib	0.83 (0.61-1.13)	0.91 (0.63-1.32)
KEYNOTE-010,2015	Pembrolizumab	Docetaxel	0.71 (0.58-0.88)	0.88 (0.73-1.04)
Kim A, 2012	Pemetrexed+Cetuximab	Pemetrexed	1.01 (0.86-1.20)	1.03 (0.87-1.21)
Kim B, 2012	Docetaxel+Cetuximab	Docetaxel	1.13 (0.90-1.41)	0.91 (0.73-1.13)
Kim, 2015	Pemetrexed	Gefitinib	0.98 (0.64-1.50)	0.96 (0.64-1.44)
Krzakowski, 2010	Vinflunine	Docetaxel	0.97 (0.81-1.18)	1.00 (0.84-1.12)
Kuo, 2013	Docetaxel	Gefitinib	-	-
Lee, 2013	Pemetrexed+Erlotinib	Erlotinib	1.08 (0.69-1.67)	0.57 (0.40-0.81)
Lee, 2013	Pemetrexed+Erlotinib	Pemetrexed	0.75 (0.49-1.13)	0.58 (0.39-0.85)
Lee, 2013	Erlotinib	Pemetrexed	0.69 (0.45-1.06)	1.01 (0.71-1.43)
Levy, 2014	Docetaxel+PX-866	Docetaxel	1.49 (0.90-2.48)	1.08 (0.71-1.63)
Li, 2010	Gefitinib	Docetaxel	0.97 (0.63-1.49)	-
Li, 2012	Pemetrexed	Docetaxel	-	-
Li, 2013	Pemetrexed+Erlotinib	Pemetrexed	0.71 (0.42-1.11)	0.63 (0.40-1.00)
Li, 2014	Erlotinib	Pemetrexed	1.01 (0.66-1.54)	0.92 (0.62-1.37)
Liu, 2015	Pemetrexed	Nab-Paclitaxel	1.23 (0.78-1.59)	1.12 (0.69-1.31)

LUME-COLUMBUS,2016	Docetaxel+Nintedanib	Docetaxel	-	-
LUME-LUNG 1, 2014	Docetaxel+Nintedanib	Docetaxel	0.94 (0.83-1.05)	0.85 (0.75-0.96)
LUME-LUNG 2, 2013	Pemetrexed+Nintedanib	Pemetrexed	1.01 (0.85-1.21)	0.84 (0.70-1.00)
Lux-Lung 8, 2015	Afatinib	Erlotinib	0.81 (0.69-0.95)	0.82 (0.68-0.99)
Maitland, 2014	Pemetrexed+Cetuximab	Cetuximab	0.63 (0.32-1.26)	0.59 (0.32-1.10)
Marangolo, 2000	Docetaxel	Vinorelbine	-	-
MARQUEE, 2013	Erlotinib+Tivantinib	Erlotinib	0.98 (0.84-1.14)	0.74 (0.64-0.85)
MARVEL, 2013	Erlotinib	Pemetrexed	-	-
METLung, 2014	Erlotinib+Onartuzumab	Erlotinib	1.27 (0.98-1.65)	0.99 (0.81-1.20)
Moran, 2014	Erlotinib+Dalotuzumab	Erlotinib	1.80 (0.87-3.72)	0.86 (0.47-1.57)
Natale, 2009	Vandetanib	Gefitinib	1.19 (0.84-1.68)	0.69 (0.50-0.96)
NCCTG N0626, 2011	Pemetrexed+Sorafenib	Pemetrexed	-	-
NCIC CTG BR.26, 2014	Dacomitinib	Placebo	1.00 (0.83-1.21)	0.66 (0.55-0.79)
NVALT-10, 2013	Erlotinib+Chemotherapy	Erlotinib	0.67 (0.49-0.91)	0.76 (0.58-1.02)
OAK,2016	Atezolizumab	Docetaxel	0.74 (0.63-0.87)	0.95 (0.82-1.10)
Paz-Ares, 2008	Paclitaxel	Docetaxel	1.09 (0.94-1.27)	-
POPLAR, 2016	Atezolizumab	Docetaxel	0.69 (0.52-0.92)	0.94 (0.72-1.23)
PROSE, 2014	Erlotinib	Chemotherapy	1.14 (0.88-1.49)	1.27 (0.99-1.62)
Ramalingam, 2011	Erlotinib+R1507	Erlotinib	0.72 (0.50-1.04)	0.93 (0.64-1.36)
Ramalingam, 2012	Dacomitinib	Erlotinib	0.82 (0.59-1.15)	0.66 (0.47-0.91)
Ramlau, 2006	Topotecan	Docetaxel	1.16 (1.00-1.35)	-
Ramlau, 2012	Docetaxel+Aflibercept	Docetaxel	1.01 (0.87-1.17)	0.82 (0.72-0.94)
REVEL, 2014	Docetaxel+Ramucirumab	Docetaxel	0.86 (0.75-0.98)	0.76 (0.68-0.86)
Sbar, 2010	Erlotinib	BMS-690514	-	-
Scagliotti, 2012	Erlotinib+Sunitinib	Erlotinib	0.94 (0.82-1.08)	0.81 (0.70-0.94)
Scagliotti, 2014	Erlotinib+Figitumumab	Erlotinib	1.09 (0.91-1.31)	1.08 (0.90-1.29)
Schiller, 2010	Pemetrexed+Matuzumab	Pemetrexed	1.66 (0.97-2.86)	1.46 (0.90-2.38)
SELECT 1,2017	Docetaxel+Selumetinib	Docetaxel	1.05 (0.85-1.30)	0.93 (0.77-1.12)
SIGN, 2006	Gefitinib	Docetaxel	0.97 (0.61-1.52)	0.94 (0.64-1.39)
Spigel, 2011	Erlotinib+Sorafenib	Erlotinib	0.89 (0.59-1.34)	0.86 (0.60-1.22)
Spigel, 2012	Erlotinib+Pazopanib	Erlotinib	1.10 (0.77-1.55)	0.59 (0.43-0.83)
Spigel, 2013	Erlotinib+Onartuzumab	Erlotinib	0.80 (0.50-1.28)	1.09 (0.73-1.62)
Sun, 2013	Pemetrexed	Docetaxel	1.02 (0.74-1.40)	1.05 (0.75-1.46)

TAILOR, 2013	Docetaxel	Erlotinib	0.78 (0.51-1.05)	0.72 (0.55-0.94)
TALISMAN, 2015	Docetaxel+Erlotinib	Erlotinib	0.87 (0.51-1.48)	0.96 (0.58-1.58)
TAX 317, 2000	Docetaxel	Placebo	0.56 (0.35-0.88)	-
TITAN, 2012	Erlotinib	Chemotherapy	0.96 (0.78-1.19)	1.19 (0.97-1.46)
TORI L-03, 2013	Erlotinib+Fulvestrant	Erlotinib	0.96 (0.60-1.55)	0.85 (0.55-1.33)
ULTIMATE, 2016	Paclitaxel+Bevacizumab	Docetaxel	1.25 (0.88-1.78)	0.62 (0.44-0.87)
V15-32, 2008	Gefitinib	Docetaxel	1.12 (0.89-1.40)	0.90 (0.72-1.12)
Wen, 2016	Docetaxel+Tamoxifen	Docetaxel	0.72 (0.48-1.06)	0.67 (0.45-0.96)
Witta, 2012	Entinostat+Erlotinib	Erlotinib	0.85 (0.59-1.23)	0.99 (0.68-1.44)
WJOG 5108L, 2016	Gefitinib	Erlotinib	1.03 (0.83-1.29)	1.07 (0.89-1.28)
ZEAL, 2011	Pemetrexed+Vandetanib	Pemetrexed	0.86 (0.68-1.09)	0.86 (0.71-1.04)
ZEST, 2011	Vandetanib	Erlotinib	1.01 (0.89-1.15)	0.98 (0.87-1.10)
Zhang, 2015	Nab-Paclitaxel+Erlotinib	Erlotinib	0.68 (0.25-0.81)	0.24 (0.14-0.74)
ZODIAC, 2010	Docetaxel+Vandetanib	Docetaxel	0.91 (0.79-1.04)	0.79 (0.71-0.88)

Objective response rate (ORR)

Trials	Treatment (arm 1)	ORR	Treatment (arm 2)	ORR	Treatment (arm 3)	ORR
ARCHER 1009, 2014	Dacomitinib	12%	Erlotinib	8%		
ARQ 197-209, 2011	Erlotinib+Tivantinib	8%	Erlotinib	6%		
ATTENTION, 2015	Erlotinib+Tivantinib	8%	Erlotinib	7%		
Bergqvist, 2014	AXL1717	0%	Docetaxel	12%		
Besse, 2014	Erlotinib+Everolimus	12%	Erlotinib	10%		
BeTa, 2011	Erlotinib+Bevacizumab	12%	Erlotinib	6%		
Bhatnagar, 2012	Docetaxel	27%	Gefitinib	33%		
Blumenschein, 2015	Docetaxel	11%	Trametinib	11%		
BR21, 2005	Erlotinib	8%	Placebo	1%		
CALGB 30704, 2014	Pemetrexed+Sunitinib	22%	Sunitinib	17%	Pemetrexed	14%
CheckMate017, 2015	Nivolumab	20%	Docetaxel	9%		
CheckMate057, 2015	Nivolumab	19%	Docetaxel	12%		
Chen, 2011	Tegafur/uracil+Gefitinib	37%	Gefitinib	34%		
CTONG0806, 2014	Pemetrexed	14%	Gefitinib	12%		
Dai, 2013	Gefitinib	17%	Pemetrexed	13%		
DATE, 2015	Gefitinib+Nimotuzumab	17%	Gefitinib	22%		
DELTA, 2014	Erlotinib	17%	Docetaxel	17%		
Dittrich, 2014	Pemetrexed+Erlotinib	16%	Pemetrexed	11%		
E1512, 2015	Cabozantinib	10%	Erlotinib+Cabozantinib	5%	Erlotinib	2%
Esteban, 2003	Paclitaxel	14%	Docetaxel	3%		
Gerber, 2014	Erlotinib+Tivantinib	0%	Chemotherapy	9%		
GFPC 05-06, 2011	Pemetrexed	12%	Docetaxel	11%		
Groen, 2013	Erlotinib+Sunitinib	5%	Erlotinib	3%		
Hainsworth, 2010	Selumetinib	5%	Pemetrexed	5%		
Han, 2011	Pemetrexed	14%	Docetaxel	10%		
HANSHIN, 2015	Docetaxel+Bevacizumab	20%	S1+Bevacizumab	2%		
Herbst, 2007	Erlotinib+Bevacizumab	18%	Chemotherapy+Bevacizumab	13%	Chemotherapy	12%

Heymach, 2007	Docetaxel+Vandetanib	26%	Docetaxel	12%		
HORG, 2013	Pemetrexed	11%	Erlotinib	8%		
Hosomi, 2015	Docetaxel+Ramucirumab	28%	Docetaxel	19%		
ICOGEN, 2013	Icotinib	28%	Gefitinib	27%		
INTEREST, 2008	Gefitinib	8%	Docetaxel	7%		
ISEL, 2005	Gefitinib	7%	Placebo	1%		
ISTANA, 2010	Gefitinib	28%	Docetaxel	8%		
Janne, 2013	Docetaxel+Selumetinib	36%	Docetaxel	0%		
JMEI, 2004	Pemetrexed	8%	Docetaxel	8%		
Jones, 2008	Topotecan	8%	Docetaxel	7%		
Juan, 2014	Docetaxel+Erlotinib	3%	Erlotinib	8%		
Kapoor, 2015	Gefitinib	-	Docetaxel	-		
Katakami, 2014	Amrubicin	14%	Docetaxel	19%		
KCSG-LU08-01, 2012	Gefitinib	44%	Pemetrexed	27%		
Kelly, 2012	Pralatrexate	2%	Erlotinib	7%		
KEYNOTE-010,2015	Pembrolizumab	18%	Docetaxel	9%		
Kim A, 2012	Pemetrexed+Cetuximab	7%	Pemetrexed	4%		
Kim B, 2012	Docetaxel+Cetuximab	8%	Docetaxel	7%		
Kim, 2015	Pemetrexed	13%	Gefitinib	8%		
Krzakowski, 2010	Vinflunine	4%	Docetaxel	5%		
Kuo, 2013	Docetaxel	-	Gefitinib	-		
Lee, 2013	Pemetrexed+Erlotinib	44%	Erlotinib	29%	Pemetrexed	10%
Levy, 2014	Docetaxel+PX-866	6%	Docetaxel	0%		
Li, 2010	Gefitinib	22%	Docetaxel	19%		
Li, 2012	Pemetrexed	8%	Docetaxel	4%		
Li, 2013	Pemetrexed+Erlotinib	27%	Pemetrexed	11%		
Li, 2014	Erlotinib	20%	Pemetrexed	8%		
Liu, 2015	Nab-Paclitaxel	15%	Pemetrexed	11%		
LUME-COLUMBUS,2016	Docetaxel+Nintedanib	-	Docetaxel	-		
LUME-LUNG 1, 2014	Docetaxel+Nintedanib	4%	Docetaxel	3%		

LUME-LUNG 2, 2013	Pemetrexed+Nintedanib	9%	Pemetrexed	8%
Lux-Lung 8, 2015	Afatinib	4%	Erlotinib	3%
Maitland, 2014	Pemetrexed+Cetuximab	13%	Cetuximab	0%
Marangolo, 2000	Docetaxel	7%	Vinorelbine	0%
MARQUEE, 2013	Erlotinib+Tivantinib	10%	Erlotinib	7%
MARVEL, 2013	Erlotinib	-	Pemetrexed	-
METLung, 2014	Erlotinib+Onartuzumab	6%	Erlotinib	9%
Moran, 2014	Erlotinib+Dalotuzumab	3%	Erlotinib	8%
Natale, 2009	Vandetanib	8%	Gefitinib	1%
NCCTG N0626, 2011	Pemetrexed+Sorafenib	13%	Pemetrexed	10%
NCIC CTG BR.26, 2014	Dacomitinib	7%	Placebo	1%
NVALT-10, 2013	Erlotinib+Chemotherapy	13%	Erlotinib	7%
OAK,2016	Atezolizumab	9%	Docetaxel	9%
Paz-Ares, 2008	Docetaxel	12%	Paclitaxel	8%
POPLAR, 2016	Atezolizumab	15%	Docetaxel	15%
PROSE, 2014	Chemotherapy	9%	Erlotinib	7%
Ramalingam, 2011	Erlotinib+R1507	7%	Erlotinib	9%
Ramalingam, 2012	Dacomitinib	17%	Erlotinib	5%
Ramlau, 2006	Topotecan	5%	Docetaxel	5%
Ramlau, 2012	Docetaxel+Aflibercept	21%	Docetaxel	8%
REVEL, 2014	Docetaxel+Ramucirumab	23%	Docetaxel	14%
Sbar, 2010	Erlotinib	-	BMS-690514	-
Scagliotti, 2012	Erlotinib+Sunitinib	11%	Erlotinib	7%
Scagliotti, 2014	Erlotinib+Figitumumab	5%	Erlotinib	4%
Schiller, 2010	Pemetrexed+Matuzumab	2%	Pemetrexed	4%
SELECT 1,2017	Docetaxel+Selumetinib	20%	Docetaxel	14%
SIGN, 2006	Gefitinib	13%	Docetaxel	14%
Spigel, 2011	Erlotinib+Sorafenib	8%	Erlotinib	11%
Spigel, 2012	Erlotinib+Pazopanib	9%	Erlotinib	4%
Spigel, 2013	Erlotinib+Onartuzumab	6%	Erlotinib	4%

Sun, 2013	Pemetrexed	9%	Docetaxel	4%
TAILOR, 2013	Docetaxel	14%	Erlotinib	3%
TALISMAN, 2015	Docetaxel+Erlotinib	8%	Erlotinib	3%
TAX 317, 2000	Docetaxel	5%	Placebo	0%
TITAN, 2012	Erlotinib	8%	Chemotherapy	6%
TORI L-03, 2013	Erlotinib+Fulvestrant	24%	Erlotinib	14%
ULTIMATE, 2016	Paclitaxel+Bevacizumab	23%	Docetaxel	5%
V15-32, 2008	Gefitinib	18%	Docetaxel	10%
Wen, 2016	Docetaxel+Tamoxifen	37%	Docetaxel	15%
Witta, 2012	Entinostat+Erlotinib	3%	Erlotinib	9%
WJOG 5108L, 2016	Gefitinib	40%	Erlotinib	36%
ZEAL, 2011	Pemetrexed+Vandetanib	19%	Pemetrexed	8%
ZEST, 2011	Vandetanib	12%	Erlotinib	12%
Zhang, 2015	Nab-Paclitaxel+Erlotinib	33%	Erlotinib	19%
ZODIAC, 2010	Docetaxel+Vandetanib	17%	Docetaxel	10%

Serious adverse events (SAE)

Trials	Treatment (arm 1)	SAE (%)	Treatment (arm 2)	SAE (%)	Treatment (arm 3)	SAE (%)
ARCHER 1009, 2014	Dacomitinib	41%	Erlotinib	39%		
Besse, 2014	Erlotinib+Everolimus	41%	Erlotinib	18%		
BeTa, 2011	Erlotinib+Bevacizumab	42%	Erlotinib	36%		
Bhatnagar, 2012	Docetaxel	13%	Gefitinib	0%		
Blumenschein, 2015	Trametinib	37%	Docetaxel	21%		
CALGB 30704, 2014	Pemetrexed+Sunitinib	51%	Sunitinib	40%	Pemetrexed	31%
CheckMate017, 2015	Nivolumab	47%	Docetaxel	54%		
CheckMate057, 2015	Nivolumab	47%	Docetaxel	41%		
Dittrich, 2014	Pemetrexed+Erlotinib	70%	Pemetrexed	52%		
E1512, 2015	Erlotinib+Cabozantinib	72%	Cabozantinib	70%	Erlotinib	33%
Gerber, 2014	Erlotinib+Tivantinib	4%	Chemotherapy	11%		
Groen, 2013	Erlotinib+Sunitinib	45%	Erlotinib	44%		
Hainsworth, 2010	Selumetinib	3%	Pemetrexed	7%		
Herbst, 2007	Chemotherapy+Bevacizumab	41%	Chemotherapy	55%	Erlotinib+Bevacizumab	33%
Hosomi, 2015	Docetaxel+Ramucirumab	32%	Docetaxel	32%		
ICOGEN, 2013	Icotinib	7%	Gefitinib	8%		
INTEREST, 2008	Gefitinib	22%	Docetaxel	29%		
ISTANA, 2010	Docetaxel	25%	Gefitinib	16%		
Janne, 2013	Docetaxel+Selumetinib	84%	Docetaxel	67%		
Katakami, 2014	Amrubicin	15%	Docetaxel	22%		
KCSG-LU08-01, 2012	Pemetrexed	15%	Gefitinib	19%		
Kelly, 2012	Pralatrexate	32%	Erlotinib	32%		
KEYNOTE-010,2015	Pembrolizumab	34%	Docetaxel	35%		
Kim A, 2012	Pemetrexed+Cetuximab	41%	Pemetrexed	30%		
Kim B, 2012	Docetaxel+Cetuximab	53%	Docetaxel	40%		
Kuo, 2013	Docetaxel	17%	Gefitinib	25%		
Lee, 2013	Pemetrexed+Erlotinib	33%	Pemetrexed	29%	Erlotinib	22%
Li, 2014	Erlotinib	7%	Pemetrexed	5%		

LUME-COLUMBUS,2016	Docetaxel+Nintedanib	50%	Docetaxel	50%
LUME-LUNG 1, 2014	Docetaxel+Nintedanib	34%	Docetaxel	31%
LUME-LUNG 2, 2013	Pemetrexed+Nintedanib	30%	Pemetrexed	33%
Lux-Lung 8, 2015	Afatinib	39%	Erlotinib	38%
Maitland, 2014	Pemetrexed+Cetuximab	30%	Cetuximab	55%
MARQUEE, 2013	Erlotinib+Tivantinib	42%	Erlotinib	37%
MARVEL, 2013	Erlotinib	0%	Pemetrexed	0%
Moran, 2014	Erlotinib+Dalotuzumab	51%	Erlotinib	58%
NCCTG N0626, 2011	Pemetrexed+Sorafenib	28%	Pemetrexed	14%
NCIC CTG BR.26, 2014	Dacomitinib	40%	Placebo	36%
OAK,2016	Atezolizumab	32%	Docetaxel	31%
POPLAR, 2016	Atezolizumab	35%	Docetaxel	34%
PROSE, 2014	Chemotherapy	2%	Erlotinib	3%
Ramalingam, 2011	Erlotinib+R1507	25%	Erlotinib	14%
Ramalingam, 2012	Dacomitinib	37%	Erlotinib	32%
Ramlau, 2012	Docetaxel+Aflibercept	48%	Docetaxel	35%
REVEL, 2014	Docetaxel+Ramucirumab	45%	Docetaxel	45%
Scagliotti, 2012	Erlotinib+Sunitinib	44%	Erlotinib	38%
Scagliotti, 2014	Erlotinib+Figitumumab	71%	Erlotinib	53%
SELECT 1,2017	Docetaxel+Selumetinib	49%	Docetaxel	32%
Spigel, 2012	Erlotinib+Pazopanib	35%	Erlotinib	28%
Spigel, 2013	Erlotinib+Onartuzumab	42%	Erlotinib	33%
Sun, 2013	Docetaxel	8%	Pemetrexed	5%
TAILOR, 2013	Docetaxel	4%	Erlotinib	2%
TALISMAN, 2015	Docetaxel+Erlotinib	27%	Erlotinib	42%
TITAN, 2012	Chemotherapy	15%	Erlotinib	10%
V15-32, 2008	Gefitinib	17%	Docetaxel	14%
Witta, 2012	Entinostat+Erlotinib	49%	Erlotinib	46%
ZEAL, 2011	Pemetrexed+Vandetanib	32%	Pemetrexed	34%
ZEST, 2011	Vandetanib	22%	Erlotinib	18%
ZODIAC, 2010	Docetaxel+Vandetanib	38%	Docetaxel	34%

Appendix 10: Results of pairwise meta-analyses and patient characteristics across trials within each comparison

Overall survival

Treatment Comparisons	GEF vs DOC	PEM vs DOC	ERL vs PEM	GEF vs PEM	PEM+ ERL vs PEM	DAC vs ERL	DOC vs PAC	DOC+ VAN vs DOC	ERL vs DOC	ERL vs Chem	ERL+ ONAR vs ERL	ERL+ SUN vs ERL	ERL+ TIV vs ERL	DOC+ ERL vs ERL	NIV vs DOC	ATE vs DOC	DOC+ NIN vs DOC	DOC+ SEL vs DOC	TOP vs DOC	DOC+ RAM vs DOC
**Trials included in MA/total available trials***	6/8	3/5	3/4	3/4	3/3	2/2	2/2	2/2	2/2	2/2	2/2	2/2	3/3	2/2	2/2	2/2	1/2	2/2	2/2	2/2
**Patients included in MA***	2462	932	642	397	399	1066	920	1474	523	709	636	1092	1522	143	854	1512	1314	597	909	1445
**Pairwise MA HR [95%CI]**	1.02 [0.93-1.11]	1.05 [0.88-1.26]	0.93 [0.76-1.13]	1.07 [0.78-1.47]	**0.71 [0.56-0.90]**	0.97 [0.79-1.18]	0.74 [0.45-1.24]	0.91 [0.8-1.04]	1.06 [0.76-1.48]	1.03 [0.87-1.21]	1.05 [0.67-1.64]	0.95 [0.84-1.09]	0.95 [0.84-1.08]	0.78 [0.54-1.14]	**0.68 [0.55-0.83]**	**0.73 [0.63-0.84]**	NA	1.01 [0.83-1.23]	1.02 [0.72-1.46]	**0.86 [0.76-0.98]**
**I² (%)**	0	0	13.9	39.7	0	34	75.5	0	51.6	0	64.8	0	0	0	28.5	0	NA	0	59.0	0
**τ**	0	0	0.05	0.18	0	0.09	0.33	0	0.17	0	0.26	0	0	0	0.08	0	NA	0	0.21	0
**Mean age**	60	57	61	61	60	63	62	59	67	61,4	63	61	62	65	62	63	61	61	59	62
**Men[†]**	65,6	66,1	56,8	52,9	47,1	61,7	77,5	62,4	69,5	71,2	58	62	62.5	92,3	65,7	51	74	53	66,2	69
**Stage IV[†]**	66,2	71	77,2	87,8	85,7	91,4	90,3	77,1	86,6	79,7	NA	95,4	86.6	84,4	79,8	100	90	90	73,1	NA
**SCC[†]**	27,4	27,9	7	6	0	31,1	32,5	27,3	22,3	26,4	21,7	26,9	11	70,8	50	30	42	8	40,5	26,2
**PS2[†]**	16	10	8,5	16,5	9,9	10,9	24,6	0,2	5,6	12,8	3	0,4	0,3	15,7	0	0	0	0	11,7	0
**Smokers[†]**	72,1	53,1	53	44	53,2	81	NA	83,4	76,3	84,7	86,9	84,3	78	97,1	86,7	84	76	90	NA	80
**Patients receiving 2nd**	95,5	96,4	88,3	91,3	100	61	86,7	100	87,2	100	65,4	67,6	62	98,6	94,2	70	100	100	100	100
**Asian patients[†]**	77,8	76	63	100	27,6	22,7	1,1	14,7	50,5	12,8	8,3	6,5	51	0	2,6	NA	19	NA	8,4	56,4

* : number ; [†] : mean proportion ; MA : pairwise meta-analysis; SCC: squamous cell carcinoma

Progression-free survival

Treatment Comparisons	GEF vs DOC	PEM vs DOC	ERL vs PEM	GEF vs PEM	PEM+ ERL vs PEM	DAC vs ERL	DOC vs PAC	DOC+ VAN vs DOC	ERL vs DOC	ERL vs Chem	ERL+ ONAR vs ERL	ERL+ SUN vs ERL	ERL+ TIV vs ERL	DOC+ ERL vs ERL	NIV vs DOC	ATE vs DOC	DOC+ NIN vs DOC	DOC+ SEL vs DOC	TOP vs DOC	DOC+ RAM vs DOC
**Trials included in MA/total available trials***	5/8	3/5	2/4	4/4	3/3	2/2	0/2	2/2	2/2	2/2	2/2	2/2	3/3	2/2	2/2	2/2	2/2	2/2	0/2	2/2
**Patients included in MA***	2364	932	285	443	399	1066	0	1474	523	709	636	1092	1522	143	854	1512	1314	597	0	1445
**Pairwise MA HR [95%CI]**	0.98 [0.88-1.11]	1.00 [0.87-1.15]	0.97 [0.75-1.26]	0.96 [0.56-1.66]	**0.61 [0.49-0.77]**	0.81 [0.58-1.13]	NA	**0.78 [0.70-0.87]**	**1.29 [1.08-1.54]**	**1.22 [1.04-1.43]**	1.01 [0.85-1.2]	**0.81 [0.7-0.94]**	**0.74 [0.65-0.83]**	0.79 [0.54-1.16]	0.77 [0.52-1.13]	0.95 [0.83-1.08]	NA	0.78 [0.49-1.22]	NA	**0.77 [0.69-0.86]**
**I^2 (%)**	14.3	0	0	85.2	0	70.0	NA	0	0	0	0	0	0	14	82.6	0	NA	69	NA	0
**τ**	0.05	0	0	0.51	0	0.21	NA	0	0	0	0	0	0	0.1	0.25	0	NA	0.28	NA	0

Objective response

Treatment Comparisons	GEF vs DOC	PEM vs DOC	ERL vs PEM	GEF vs PEM	PEM+ ERL vs PEM	DAC vs ERL	DOC vs PACL	DOC+ VAN vs DOC	ERL vs DOC	ERL vs Chem	ERL+ ONAR vs ERL	ERL+ SUN vs ERL	ERL+ TIV vs ERL	DOC+ ERL vs ERL	NIV vs DOC	ATE vs DOC	DOC+ NIN vs DOC	DOC+ SEL vs DOC	TOP vs DOC	DOC+ RAM vs DOC
**Trials included in MA/total available trials***	8/8	5/5	3/4	4/4	3/3	2/2	2/2	2/2	2/2	2/2	2/2	2/2	3/3	2/2	2/2	2/2	2/2	2/2	2/2	2/2
**Patients included in MA***	2505	1224	617	443	399	1066	920	1474	523	709	636	1092	1522	141	854	1512	1314	597	909	1445
**Pairwise MA HR [95%CI]**	**1.62 [1.06-2.47]**	1.32 [0.87-1.98]	1.91. [0.69-5.26]	1.25 [0.68-2.31]	**3.34 [1.35-8.27]**	2.08 [0.94-4.60]	0.71 [0.09-5.53]	**1.88 [1.39-2.55]**	0.45 [0.09-2.38]	1.01 [0.57-1.77]	0.78 [0.42-1.44]	**1.61 [1.03-2.51]**	**1.55 [1.06-2.26]**	1.02 [0.11-9]	**1.90 [1.28-2.81]**	1.01 [0.73-1.41]	NA	6.49 [0.24-176]	1.01 [0.55-1.85]	**1.86 [1.42-2.45]**
**I^2 (%)**	45	0	74.5	28	58.0	54	71.9	0	82	0	0	0	0	43.8	1.9	0	NA	84	0	0
**τ**	0.34	0	0.77	0.33	0.61	0.44	1.30	0	1.09	0	0	0	0	1.04	0.04	0	NA	1.76	0	0

Serious adverse events

Treatment Comparisons	GEF vs DOC	PEM vs DOC	ERL vs PEM	GEF vs PEM	PEM+ ERL vs PEM	DAC vs ERL	DOC vs PAC	DOC+ VAN vs DOC	ERL vs DOC	ERL vs Chem	ERL+ ONAR vs ERL	ERL+ SUN vs ERL	ERL+ TIV vs ERL	DOC+ ERL vs ERL	NIV vs DOC	ATE vs DOC	DOC+ NIN vs DOC	DOC+ SEL vs DOC	TOP vs DOC	DOC+ RAM vs DOC
**Trials included in MA/total available [illegible]**	5/8	1/5	3/4	1/4	2/3	2/2	0/2	1/2	1/2	2/2	1/2	2/2	1/3	1/2	2/2	2/2	2/2	2/2	0/2	2/2
**Patients included in MA***	2128	208	303	135	310	1059	0	1379	211	672	136	1078	1037	73	815	1512	1314	597	0	1437
**Pairwise MA HR [95%CI]**	0.8 [0.52-1.21]	NA	0.78 [0.41-1.48]	NA	1.64 [0.95-2.83]	1.11 [0.87-1.42]	NA	NA	NA	0.73 [0.42-1.27]	NA	1.25 [0.98-1.59]	NA	NA	0.98 [0.59-1.64]	1.03 [0.83-1.28]	1.14 [0.91-1.43]	**2.1 [1.5-2.96]**	NA	0.99 [0.80-1.22]
**I^2 (%)**	43.5	NA	0	NA	24.6	0	NA	NA	NA	0	NA	0	NA	NA	66.6	0	0	0	NA	0
**τ**	0.29	NA	0	NA	0.19	0	NA	NA	NA	0	NA	0	NA	NA	0.30	0	0	0	NA	0

Appendix 11: Treatment categories analysis

OS (HR (95% CrI))

Targeted therapy	**1.17 (1.06,1.31)**	**0.94 (0.88,0.99)**	**0.66 (0.59,0.74)**	0.97 (0.91,1.04)	**0.88 (0.82,0.96)**
**0.85 (0.77,0.95)**	Placebo	**0.80 (0.71,0.91)**	**0.57 (0.48,0.66)**	**0.83 (0.73,0.94)**	**0.75 (0.66,0.86)**
**1.07 (1.01,1.13)**	**1.25 (1.10,1.41)**	Monochemotherapy	**0.71 (0.63,0.79)**	1.04 (0.95,1.13)	**0.94 (0.90,0.99)**
**1.51 (1.34,1.71)**	**1.77 (1.51,2.08)**	**1.41 (1.27,1.58)**	Immunotherapy	**1.47 (1.29,1.69)**	**1.34 (1.19,1.52)**
1.03 (0.96,1.10)	**1.20 (1.06,1.37)**	0.96 (0.88,1.05)	**0.68 (0.59,0.78)**	Dual targeted therapies	0.91 (0.82,1.00)
**1.13 (1.05,1.22)**	**1.32 (1.16,1.51)**	**1.06 (1.01,1.12)**	**0.75 (0.66,0.84)**	1.10 (1.00,1.22)	Chemotherapy+ Targeted therapy

PFS (HR (95% CrI))

Targeted therapy	**1.58 (1.25,1.98)**	0.92 (0.84,1.00)	**0.79 (0.67,0.94)**	**0.82 (0.74,0.90)**	**0.76 (0.68,0.84)**
**0.63 (0.50,0.80)**	Placebo	**0.58 (0.46,0.74)**	**0.50 (0.38,0.67)**	**0.52 (0.40,0.66)**	**0.48 (0.37,0.62)**
1.09 (1.00,1.19)	**1.72 (1.34,2.19)**	Monochemotherapy	0.87 (0.74,1.01)	0.89 (0.78,1.01)	**0.83 (0.76,0.89)**
**1.26 (1.06,1.50)**	**1.98 (1.49,2.64)**	1.15 (0.99,1.34)	Immunotherapy	1.03 (0.84,1.25)	0.95 (0.80,1.13)
**1.23 (1.11,1.35)**	**1.93 (1.50,2.48)**	1.12 (0.99,1.28)	0.97 (0.80,1.19)	Dual targeted therapies	0.93 (0.81,1.07)
**1.32 (1.19,1.47)**	**2.08 (1.62,2.68)**	**1.21 (1.12,1.31)**	1.05 (0.89,1.24)	1.08 (0.94,1.24)	Chemotherapy+ Targeted therapy

SAE (OR (95% CrI))

Targeted therapy	0.86 (0.46,1.58)	1.10 (0.87,1.38)	1.11 (0.76,1.58)	**1.42 (1.14,1.77)**	**1.40 (1.08,1.81)**
1.17 (0.63,2.16)	Placebo	1.29 (0.67,2.46)	1.29 (0.63,2.61)	1.66 (0.86,3.19)	1.64 (0.84,3.17)
0.91 (0.73,1.14)	0.78 (0.41,1.50)	Monochemotherapy	1.00 (0.75,1.33)	1.29 (0.96,1.75)	**1.27 (1.08,1.51)**
0.90 (0.63,1.32)	0.77 (0.38,1.60)	1.00 (0.75,1.33)	Immunotherapy	1.28 (0.85,1.97)	1.27 (0.91,1.78)
**0.70 (0.56,0.88)**	0.60 (0.31,1.16)	0.78 (0.57,1.04)	0.78 (0.51,1.18)	Dual targeted therapies	0.99 (0.71,1.36)
**0.71 (0.55,0.93)**	0.61 (0.32,1.19)	0.79 (0.66,0.93)	0.79 (0.56,1.09)	1.01 (0.73,1.41)	Chemotherapy+ Targeted therapy

HRs compared the column-defining versus the treatment row-defining treatment. Significant results are in bold font.

Cumulative ranking curves and SUCRA values for OS

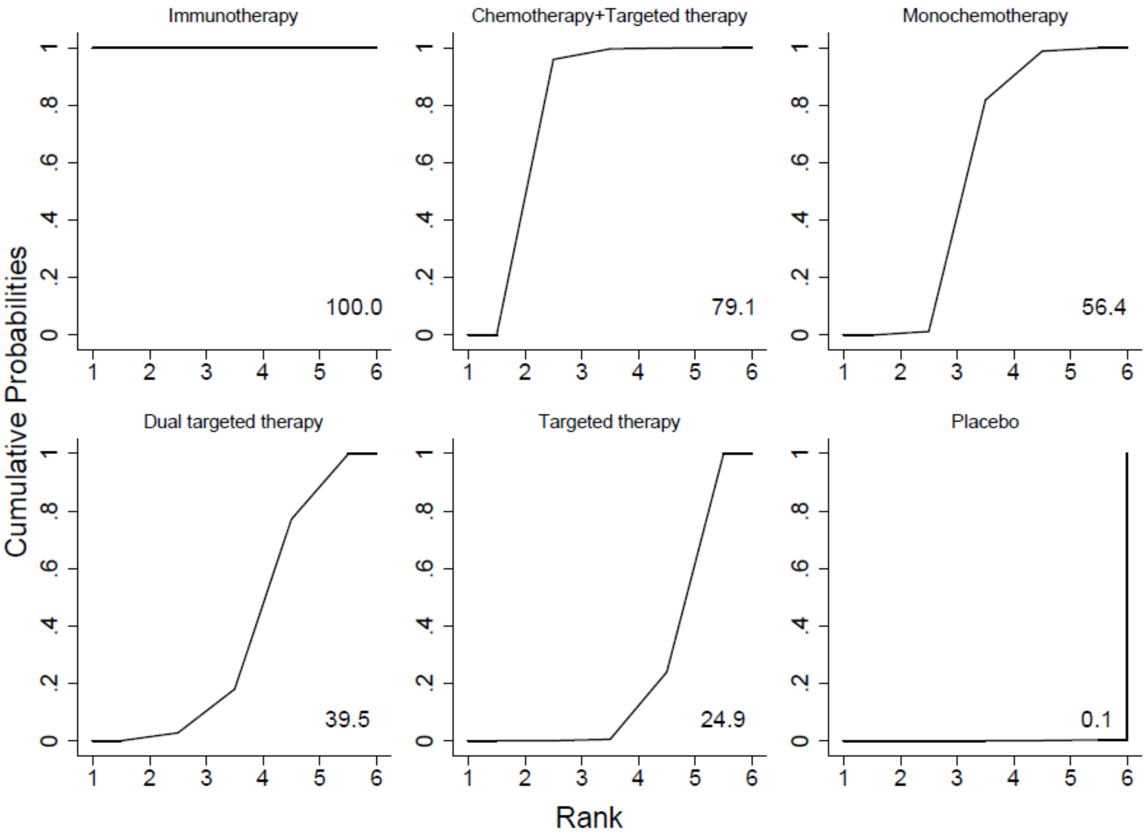

Rankograms for OS

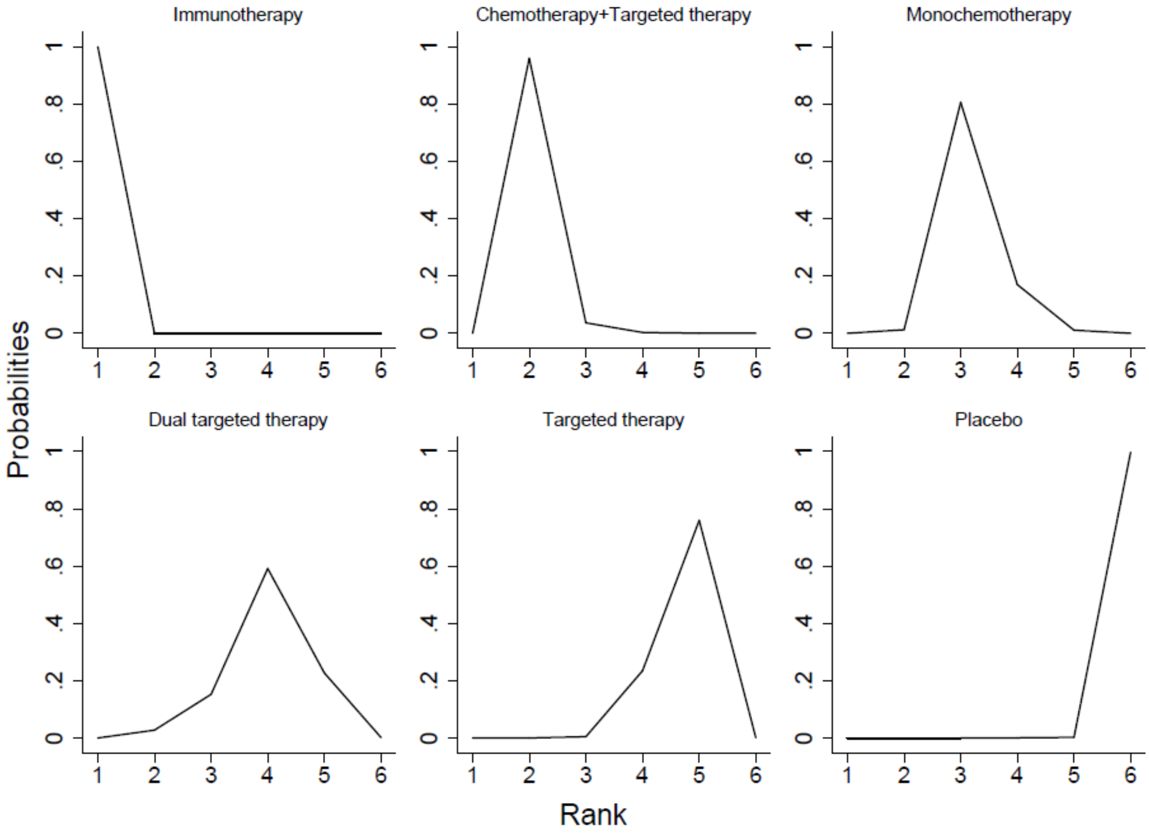

Cumulative ranking curves and SUCRA values for PFS

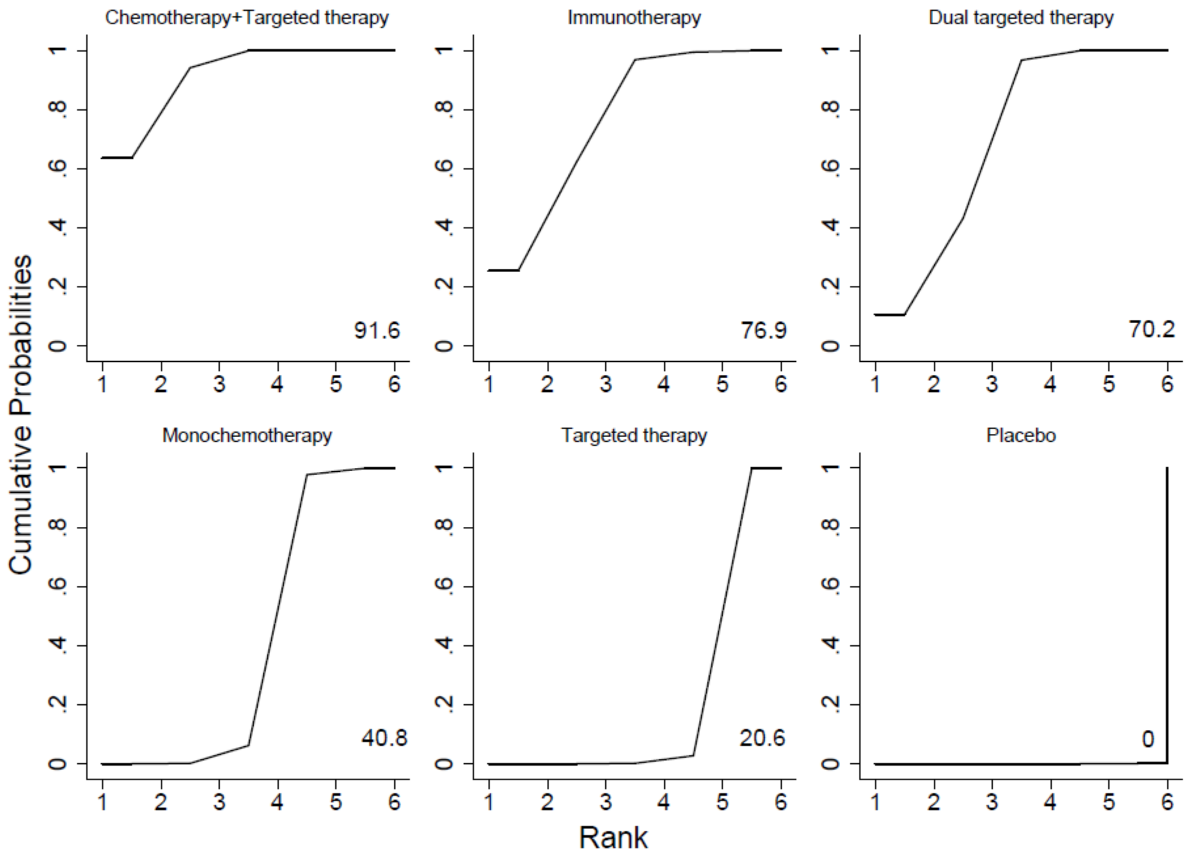

Rankograms for PFS

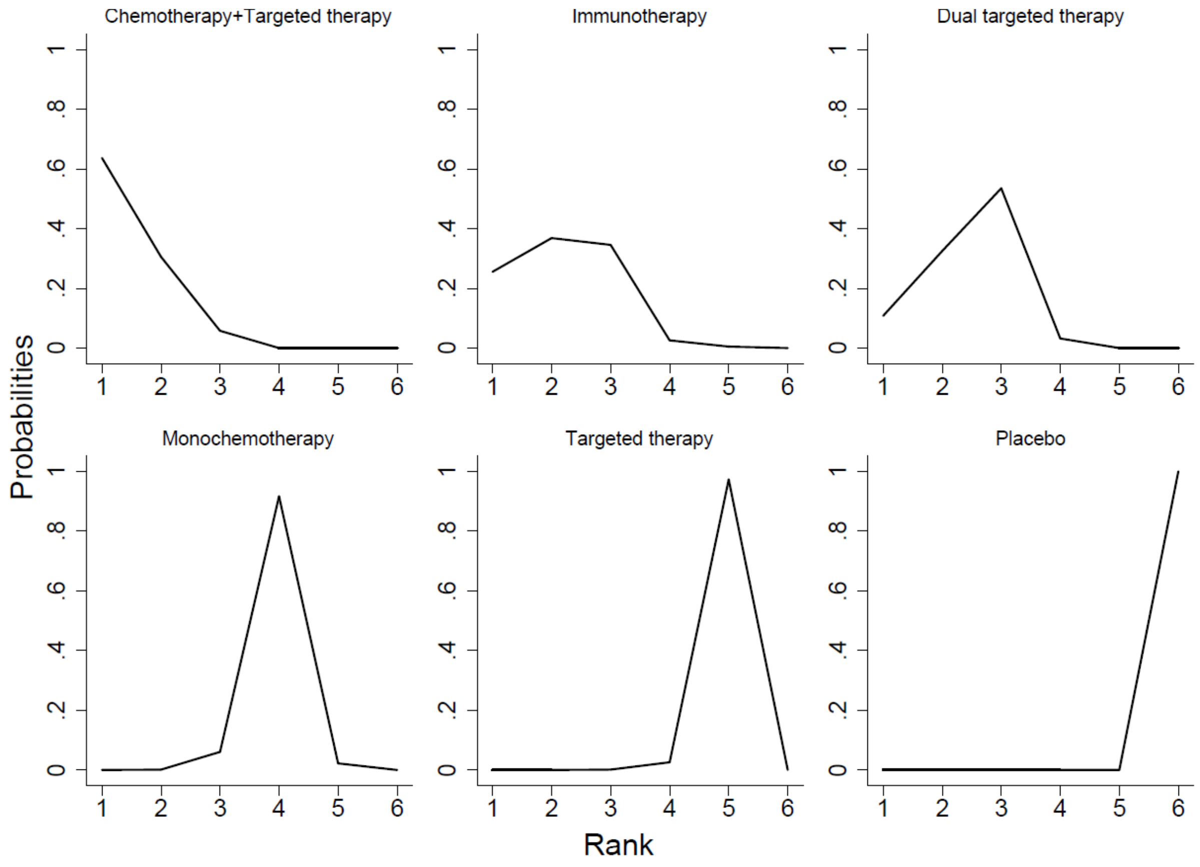

Cumulative ranking curves and SUCRA values for SAE

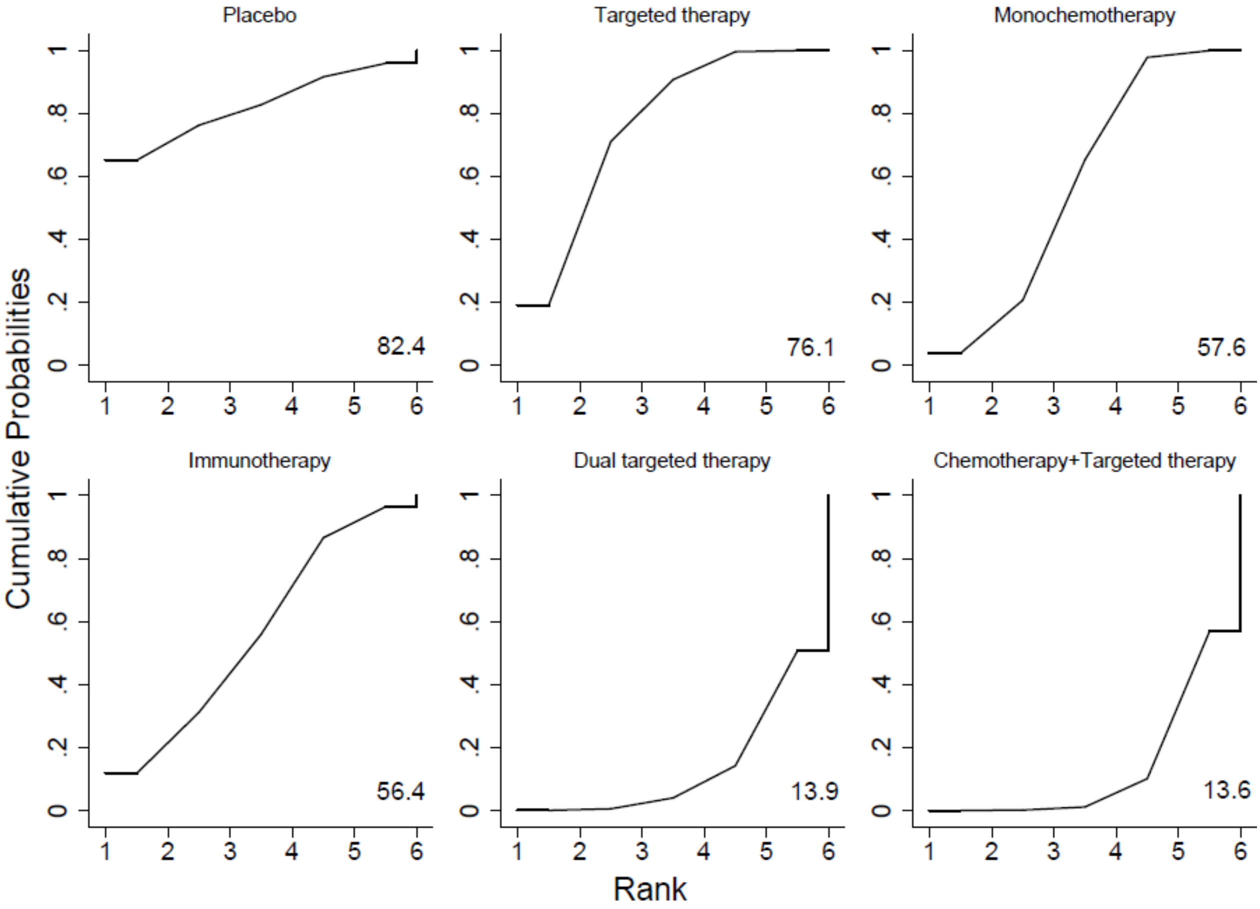

Rankograms for SAE

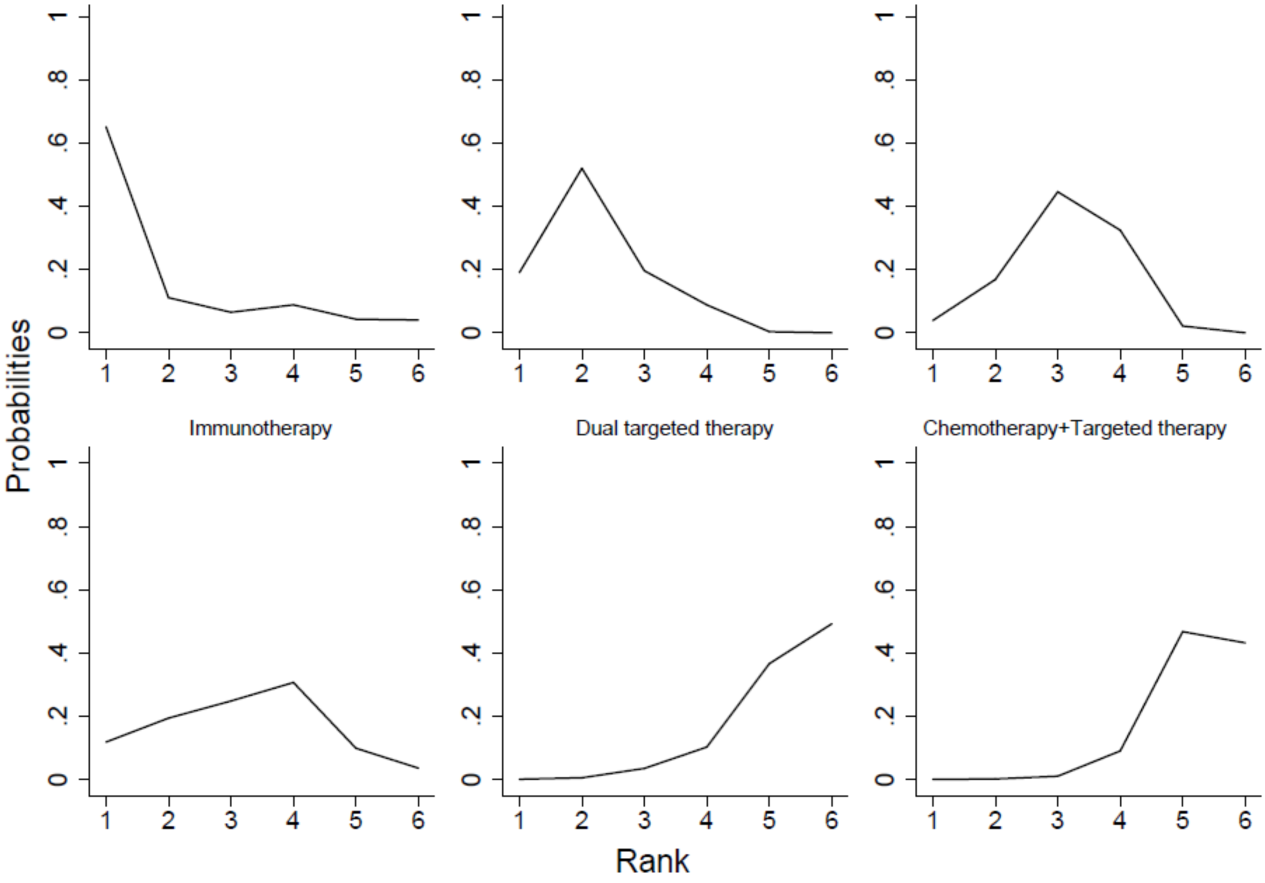

Appendix 12: WinBUGS codes

WinBUGS models used for all outcomes

## Network meta-analysis for study-level data not accounting for multi-arm trials##

```
model {
	for(i in 1:ns) {
		y[i] ~ dnorm(delta[i],prec[i])
		prec[i]<- 1/V[i]
		V[i]<- se[i]*se[i]
		delta[i] ~ dnorm(md[i],taud[i])
		md[i]<- d[t[i]]-d[b[i]]
		taud[i]<- tau
	}
	d[ref]<- 0

	for(k in 1:(ref-1)) {
		d[k] ~ dnorm(0,.0001)
	}
	for(k in (ref+1):nt) {
		d[k] ~ dnorm(0,.0001)
	}
	sd ~ dnorm(0,1)I(0,)
	tau<- 1/pow(sd,2)

	for (c in 1:(ref-1)) {
		ES.ref[c]<- exp(d[c] - d[ref])
		LES.ref[c]<- d[c] - d[ref]
		predLES.ref[c] ~ dnorm( LES.ref[c],tau)
		predES.ref[c]<- exp(predLES.ref[c])
	}
	for (c in (ref+1):nt) {
		ES.ref[c]<- exp(d[c] - d[ref])
		LES.ref[c]<- d[c] - d[ref]
		predLES.ref[c] ~ dnorm( LES.ref[c],tau)
		predES.ref[c]<- exp(predLES.ref[c])
	}
	for (c in 1:(nt-1)) {
		for (k in (c+1):nt) {
			ES[c,k]<- exp(d[c] - d[k])
			LES[c,k]<- d[c] - d[k]
			predLES[c,k] ~ dnorm(LES[c,k],tau)
			predES[c,k]<- exp(predLES[c,k])
		}
	}
	for (k in 1:nt) {
		order[k]<-nt+1-rank(d[],k)
# this is when the outcome is positive - omit  'nt+1-' when the outcome is negative
		most.effective[k]<- equals(order[k],1)
```

```
			for(j in 1:nt) {
				effectiveness[k,j]<- equals(order[k],j)
			}
		}
		for(k in 1:nt) {
			for(j in 1:nt) {
				cumeffectiveness[k,j]<- sum(effectiveness[k,1:j])
			}
		}
		for(k in 1:nt) {
			SUCRA[k]<- sum(cumeffectiveness[k,1:(nt-1)]) /(nt-1)
		}
}
```

## Network meta-analysis for arm-level binary data accounting for multi-arm trials##

```
model {
		for(i in 1:ns) {
		w[i,1]<- 0
		  delta[i,t[i,1]]<- 0
		  mu[i] ~ dnorm(0,.0001)

			for (k in 1:na[i]) {
			r[i,t[i,k]] ~ dbin(p[i,t[i,k]],n[i,t[i,k]])
			}
			logit(p[i,t[i,1]])<- mu[i]

			for (k in 2:na[i]) {
		logit(p[i,t[i,k]])<- mu[i] + delta[i,t[i,k]]
				delta[i,t[i,k]] ~ dnorm(md[i,t[i,k]],taud[i,t[i,k]])
				taud[i,t[i,k]]<- tau *2*(k-1)/k
	md[i,t[i,k]]<-  d[t[i,k]] - d[t[i,1]]  + sw[i,k]
	w[i,k]<- (delta[i,t[i,k]]  - d[t[i,k]] + d[t[i,1]])
	sw[i,k]<- sum(w[i,1:k-1])/(k-1)
			}
		}
		sd ~ dunif(0,5)
		tau<- 1/pow(sd,2)

		d[ref]<- 0

		for(k in 1:(ref-1)) {
			d[k] ~ dnorm(0,.0001)
		}
		for(k in (ref+1):nt) {
			d[k] ~ dnorm(0,.0001)
		}
		for (c in 1:(ref-1)) {
			OR.ref[c]<- exp(d[c] - d[ref])
			LOR.ref[c]<- d[c] - d[ref]
			predLOR.ref[c] ~ dnorm(LOR.ref[c],tau)
```

```
			predOR.ref[c]<- exp(predLOR.ref[c])
		}
		for (c in (ref+1):nt) {
			OR.ref[c]<- exp(d[c] - d[ref])
			LOR.ref[c]<- d[c] - d[ref]
		predLOR.ref[c] ~ dnorm(LOR.ref[c],tau)
			predOR.ref[c]<- exp(predLOR.ref[c])
		}
		for(i in 1:(nt-1)) {
			for (j in (i+1):nt) {
				OR[i,j]<- exp(d[i] - d[j])
				LOR[i,j]<- d[i] - d[j]
				predLOR[i,j] ~ dnorm(LOR[i,j],tau)
				predOR[i,j]<- exp(predLOR[i,j])
			}
		}
		for(k in 1:nt) {
			order[k]<- nt+1-rank(d[],k)
# this is when the outcome is positive - omit  'nt+1-' when the outcome is negative
			most.effective[k]<-equals(order[k],1)

			for(j in 1:nt) {
				effectiveness[k,j]<- equals(order[k],j)
			}
		}
		for(k in 1:nt) {
			for(j in 1:nt) {
				cumeffectiveness[k,j]<- sum(effectiveness[k,1:j])
			}
		}
		for(k in 1:nt) {
			SUCRA[k]<- sum(cumeffectiveness[k,1:(nt-1)]) /(nt-1)
		}
}
```

Appendix 13 : Difference of restricted mean survival times (RMST) at 18 months and the 1-year overall survival for trials comparing immunotherapy to docetaxel

Trial	Arm	HR (95% CI)	Median OS (95% CI), mo	1-year OS (95% CI),%	Difference of RMST at 18 mo (95% CI),mo
CheckMate017	Docetaxel	-	6.0 (5.1-7.3)	39 (34-45)	-
	Nivolumab	0.59 (0.44-0.79)	9.2 (7.3-13.3)	50 (45-56)	0.9 (0.8-1)
CheckMate057	Docetaxel	-	9.4 (8.1-10.7)	23 (17-31)	-
	Nivolumab	0.73 (0.60-0.89)	12.2 (9.7-15)	42 (34-54)	2.7 (1.2-4.3)
KEYNOTE-010	Docetaxel	-	8.5 (7.5-9.8)	34 (29-41)	-
	Pembrolizumab	0.71 (0.58-0.88)	12.7 (10-17.3)	43 (37-49)	1.3 (-0.1-2.8)
OAK	Docetaxel	-	9.6 (8.6-11.2)	42 (37-47)	-
	Atezolizumab	0.74 (0.63-0.87)	13.8 (11.8-15.7)	55 (49-61)	1.4 (0.4-2.4)
POPLAR	Docetaxel	-	9.7 (8.6-12)	41 (34-50)	-
	Atezolizumab	0.69 (0.52-0.92)	12.6 (12.6-16.4)	51 (44-60)	1.4 (0.5-2.4)
