# Supplementary material for: Comparative efficacy and safety of second-line treatments for advanced non-small cell lung cancer with wild-type or unknown status for epidermal growth factor receptor: a systematic review and network meta-analysis
Source: BMC Med. 2017 Oct 30;15:193. doi: 10.1186/s12916-017-0954-x (PMC5662096; doi:10.1186/s12916-017-0954-x)
Supplement: Supplementary file 2 — Forest plots for four outcomes. Figure S2. Cumulative ranking curves and SUCRA values for four outcomes. Figure S3. Rankograms for four outcomes. Figure S4. Evaluation of inconsistency. Figure S5. Subgroup analyses by histology and ethnicity: network graphs of trials for OS, forest plots, and SUCRA values for OS and PFS. Figure S6. Funnel plots of small-study effects. Figure S7. Overall assessment of bias. (PDF 1930 kb) [file 12916_2017_954_MOESM2_ESM.pdf]

Supplementary appendix figures for

"Comparative efficacy and safety of second-line treatments for advanced non–small-cell lung cancer with wild-type or unknown status for epidermal growth factor receptor: a systematic review and network meta-analyses"

Appendix Figure 1: Forest plots for 4 outcomes..p2

Appendix Figure 2: Cumulative ranking curves and SUCRA values for 4 outcomes..................p55

Appendix Figure 3: Rankograms for 4 outcomes...p60

Appendix Figure 4: Evaluation of inconsistency...p65

Appendix Figures 5: Subgroup analyses by histology and ethnicity: network graphs of trials for OS, forest plots and SUCRA values for OS and PFS...p70

Appendix Figure 6: Funnel plots of small-study effects...p120

Appendix Figure 7: Overall assessment of bias...p122

Appendix Figure 1: Forest plots for 4 outcomes

(a) Overall survival

Treatments vs. Docetaxel	HR[CrI 95%]
Erlotinib+Cabozantinib	0.53 [0.27 − 1.05]
Nivolumab	0.69 [0.56 − 0.83]
Cabozantinib	0.7 [0.35 − 1.25]
Pembrolizumab	0.71 [0.56 − 0.9]
Docetaxel+Tamoxifen	0.71 [0.46 − 1.09]
Erlotinib+R1507	0.73 [0.49 − 1.09]
Atezolizumab	0.73 [0.62 − 0.87]
Pemetrexed+Erlotinib	0.74 [0.56 − 0.96]
Docetaxel+Erlotinib	0.77 [0.52 − 1.21]
Afatinib	0.82 [0.64 − 1.06]
Nab−Paclitaxel	0.84 [0.54 − 1.23]
Pralatrexate	0.84 [0.58 − 1.19]
Tegafur/uracil+Gefitinib	0.85 [0.47 − 1.43]
Docetaxel+Ramucirumab	0.86 [0.73 − 1.01]
Entinostat+Erlotinib	0.87 [0.58 − 1.29]
Gefitinib+Nimotuzumab	0.89 [0.58 − 1.39]
Pemetrexed+Vandetanib	0.9 [0.67 − 1.2]
Erlotinib+Sorafenib	0.91 [0.59 − 1.43]
Docetaxel+Vandetanib	0.91 [0.77 − 1.08]
Docetaxel+Nintedanib	0.94 [0.8 − 1.11]
Erlotinib+Fulvestrant	0.94 [0.56 − 1.54]
Trametinib	0.96 [0.49 − 1.92]
Erlotinib+Tivantinib	0.96 [0.79 − 1.17]
Vinflunine	0.97 [0.78 − 1.2]
Erlotinib+Sunitinib	0.97 [0.79 − 1.21]
Erlotinib+Bevacizumab	0.98 [0.75 − 1.28]
Amrubicin	1.01 [0.71 − 1.45]
Docetaxel+Aflibercept	1.01 [0.83 − 1.22]
Docetaxel+Selumetinib	1.01 [0.81 − 1.25]
Erlotinib	1.01 [0.89 − 1.16]
Pemetrexed	1.03 [0.92 − 1.17]
Gefitinib	1.04 [0.94 − 1.14]
Vandetanib	1.05 [0.87 − 1.29]
Pemetrexed+Cetuximab	1.05 [0.83 − 1.32]
Pemetrexed+Nintedanib	1.06 [0.83 − 1.34]
Icotinib	1.06 [0.81 − 1.38]
Dacomitinib	1.07 [0.89 − 1.29]
Topotecan	1.1 [0.92 − 1.3]
Erlotinib+Figitumumab	1.1 [0.86 − 1.42]
AXL1717	1.1 [0.94 − 1.28]
Erlotinib+Pazopanib	1.11 [0.75 − 1.65]
Erlotinib+Onartuzumab	1.13 [0.86 − 1.49]
Docetaxel+Cetuximab	1.14 [0.88 − 1.45]
Paclitaxel	1.16 [0.98 − 1.41]
Placebo	1.22 [1.05 − 1.43]
Paclitaxel+Bevacizumab	1.23 [0.87 − 1.8]
Erlotinib+Everolimus	1.32 [0.7 − 2.38]
Sunitinib	1.46 [0.89 − 2.41]
Docetaxel+PX−866	1.5 [0.91 − 2.43]
Cetuximab	1.7 [0.83 − 3.43]
Pemetrexed+Matuzumab	1.73 [0.98 − 3.02]
Erlotinib+Dalotuzumab	1.74 [0.86 − 3.56]
Pemetrexed+Sunitinib	2.06 [1.21 − 3.52]

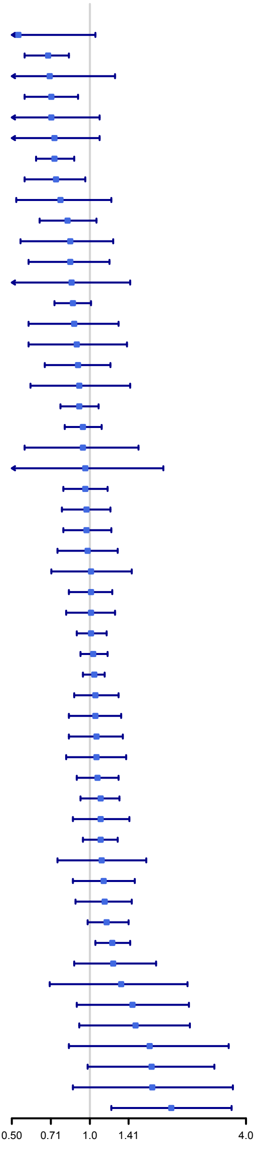

Treatments vs. Pemetrexed	HR[CrI 95%]
Erlotinib+Cabozantinib	0.51 [0.26 − 1.02]
Nivolumab	0.67 [0.52 − 0.83]
Cabozantinib	0.68 [0.34 − 1.19]
Pembrolizumab	0.69 [0.53 − 0.89]
Docetaxel+Tamoxifen	0.69 [0.44 − 1.07]
Erlotinib+R1507	0.7 [0.48 − 1.05]
Atezolizumab	0.71 [0.57 − 0.87]
Pemetrexed+Erlotinib	0.71 [0.56 − 0.91]
Docetaxel+Erlotinib	0.75 [0.5 − 1.16]
Afatinib	0.79 [0.62 − 1.01]
Nab−Paclitaxel	0.81 [0.54 − 1.16]
Pralatrexate	0.82 [0.56 − 1.15]
Tegafur/uracil+Gefitinib	0.82 [0.45 − 1.39]
Docetaxel+Ramucirumab	0.83 [0.67 − 1.02]
Entinostat+Erlotinib	0.84 [0.55 − 1.26]
Pemetrexed+Vandetanib	0.87 [0.66 − 1.12]
Gefitinib+Nimotuzumab	0.87 [0.55 − 1.35]
Erlotinib+Sorafenib	0.88 [0.57 − 1.37]
Docetaxel+Vandetanib	0.88 [0.71 − 1.08]
Docetaxel+Nintedanib	0.91 [0.73 − 1.12]
Erlotinib+Fulvestrant	0.91 [0.56 − 1.48]
Trametinib	0.92 [0.47 − 1.85]
Erlotinib+Tivantinib	0.93 [0.75 − 1.13]
Vinflunine	0.93 [0.73 − 1.19]
Erlotinib+Sunitinib	0.94 [0.76 − 1.17]
Erlotinib+Bevacizumab	0.95 [0.72 − 1.24]
Docetaxel	0.97 [0.85 − 1.09]
Amrubicin	0.97 [0.66 − 1.42]
Docetaxel+Aflibercept	0.98 [0.77 − 1.21]
Erlotinib	0.98 [0.84 − 1.13]
Docetaxel+Selumetinib	0.98 [0.75 − 1.25]
Gefitinib	1 [0.88 − 1.14]
Pemetrexed+Cetuximab	1.01 [0.82 − 1.23]
Vandetanib	1.01 [0.83 − 1.26]
Pemetrexed+Nintedanib	1.02 [0.82 − 1.25]
Icotinib	1.02 [0.78 − 1.34]
Dacomitinib	1.04 [0.85 − 1.25]
Topotecan	1.06 [0.85 − 1.3]
AXL1717	1.06 [0.87 − 1.29]
Erlotinib+Figitumumab	1.06 [0.82 − 1.38]
Erlotinib+Pazopanib	1.07 [0.72 − 1.58]
Erlotinib+Onartuzumab	1.09 [0.82 − 1.44]
Docetaxel+Cetuximab	1.1 [0.83 − 1.44]
Paclitaxel	1.12 [0.91 − 1.4]
Placebo	1.18 [1 − 1.4]
Paclitaxel+Bevacizumab	1.19 [0.83 − 1.77]
Erlotinib+Everolimus	1.28 [0.67 − 2.29]
Sunitinib	1.41 [0.85 − 2.26]
Docetaxel+PX−866	1.45 [0.86 − 2.38]
Cetuximab	1.63 [0.81 − 3.24]
Pemetrexed+Matuzumab	1.67 [0.96 − 2.86]
Erlotinib+Dalotuzumab	1.68 [0.83 − 3.47]
Pemetrexed+Sunitinib	1.99 [1.2 − 3.31]

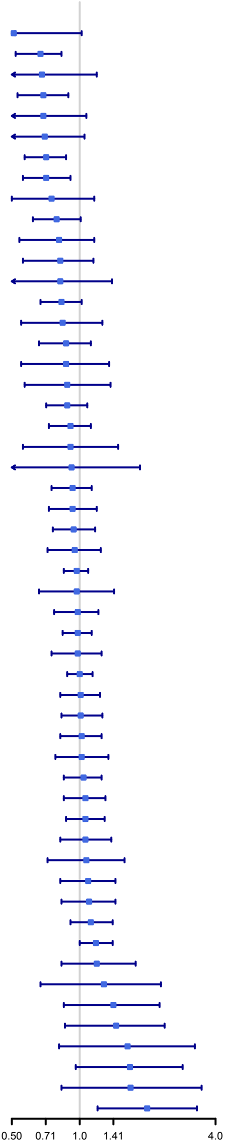

Treatments vs. Erlotinib	HR[CrI 95%]
Erlotinib+Cabozantinib	0.52 [0.27 − 1.03]
Nivolumab	0.68 [0.53 − 0.86]
Cabozantinib	0.7 [0.36 − 1.2]
Pembrolizumab	0.7 [0.54 − 0.92]
Docetaxel+Tamoxifen	0.7 [0.45 − 1.1]
Erlotinib+R1507	0.71 [0.5 − 1.04]
Atezolizumab	0.72 [0.58 − 0.88]
Pemetrexed+Erlotinib	0.73 [0.55 − 0.97]
Docetaxel+Erlotinib	0.77 [0.51 − 1.16]
Afatinib	0.81 [0.66 − 0.99]
Nab−Paclitaxel	0.83 [0.53 − 1.21]
Pralatrexate	0.83 [0.6 − 1.15]
Tegafur/uracil+Gefitinib	0.84 [0.48 − 1.42]
Docetaxel+Ramucirumab	0.85 [0.69 − 1.04]
Entinostat+Erlotinib	0.86 [0.59 − 1.24]
Gefitinib+Nimotuzumab	0.88 [0.56 − 1.38]
Pemetrexed+Vandetanib	0.89 [0.65 − 1.19]
Erlotinib+Sorafenib	0.9 [0.59 − 1.36]
Docetaxel+Vandetanib	0.9 [0.72 − 1.11]
Docetaxel+Nintedanib	0.93 [0.75 − 1.15]
Erlotinib+Fulvestrant	0.93 [0.57 − 1.48]
Trametinib	0.94 [0.48 − 1.9]
Erlotinib+Tivantinib	0.95 [0.81 − 1.09]
Vinflunine	0.95 [0.74 − 1.23]
Erlotinib+Sunitinib	0.96 [0.81 − 1.13]
Erlotinib+Bevacizumab	0.96 [0.77 − 1.22]
Docetaxel	0.99 [0.87 − 1.13]
Amrubicin	1 [0.67 − 1.47]
Docetaxel+Aflibercept	1 [0.79 − 1.25]
Docetaxel+Selumetinib	1 [0.76 − 1.31]
Gefitinib	1.02 [0.9 − 1.16]
Pemetrexed	1.02 [0.89 − 1.18]
Pemetrexed+Cetuximab	1.04 [0.81 − 1.32]
Vandetanib	1.04 [0.9 − 1.23]
Icotinib	1.04 [0.8 − 1.38]
Pemetrexed+Nintedanib	1.04 [0.8 − 1.33]
Dacomitinib	1.06 [0.92 − 1.22]
Topotecan	1.08 [0.87 − 1.33]
AXL1717	1.09 [0.89 − 1.32]
Erlotinib+Figitumumab	1.09 [0.88 − 1.35]
Erlotinib+Pazopanib	1.1 [0.76 − 1.59]
Erlotinib+Onartuzumab	1.11 [0.88 − 1.42]
Docetaxel+Cetuximab	1.12 [0.85 − 1.49]
Paclitaxel	1.14 [0.93 − 1.44]
Placebo	1.2 [1.05 − 1.39]
Paclitaxel+Bevacizumab	1.21 [0.85 − 1.81]
Erlotinib+Everolimus	1.3 [0.69 − 2.32]
Sunitinib	1.44 [0.86 − 2.36]
Docetaxel+PX−866	1.49 [0.88 − 2.46]
Cetuximab	1.69 [0.82 − 3.37]
Pemetrexed+Matuzumab	1.69 [0.98 − 2.97]
Erlotinib+Dalotuzumab	1.72 [0.84 − 3.53]
Pemetrexed+Sunitinib	2.04 [1.2 − 3.46]

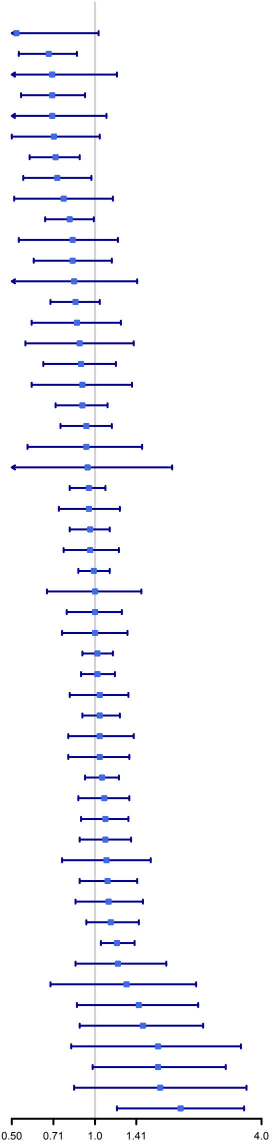

Treatments vs. Gefitinib	HR[CrI 95%]
Erlotinib+Cabozantinib	0.51 [0.26 − 1.03]
Nivolumab	0.66 [0.53 − 0.83]
Cabozantinib	0.68 [0.34 − 1.19]
Pembrolizumab	0.68 [0.53 − 0.88]
Docetaxel+Tamoxifen	0.69 [0.44 − 1.07]
Erlotinib+R1507	0.7 [0.48 − 1.04]
Atezolizumab	0.7 [0.58 − 0.85]
Pemetrexed+Erlotinib	0.72 [0.54 − 0.94]
Docetaxel+Erlotinib	0.75 [0.5 − 1.16]
Afatinib	0.79 [0.62 − 1.01]
Pralatrexate	0.81 [0.57 − 1.15]
Nab−Paclitaxel	0.82 [0.52 − 1.19]
Tegafur/uracil+Gefitinib	0.82 [0.46 − 1.38]
Docetaxel+Ramucirumab	0.83 [0.69 − 1]
Entinostat+Erlotinib	0.84 [0.56 − 1.26]
Gefitinib+Nimotuzumab	0.86 [0.57 − 1.34]
Pemetrexed+Vandetanib	0.87 [0.64 − 1.16]
Erlotinib+Sorafenib	0.87 [0.58 − 1.36]
Docetaxel+Vandetanib	0.88 [0.73 − 1.07]
Erlotinib+Fulvestrant	0.91 [0.55 − 1.49]
Docetaxel+Nintedanib	0.91 [0.74 − 1.1]
Trametinib	0.93 [0.48 − 1.85]
Erlotinib+Tivantinib	0.93 [0.76 − 1.12]
Vinflunine	0.93 [0.74 − 1.18]
Erlotinib+Sunitinib	0.94 [0.76 − 1.16]
Erlotinib+Bevacizumab	0.95 [0.73 − 1.23]
Docetaxel	0.97 [0.88 − 1.06]
Amrubicin	0.97 [0.67 − 1.41]
Docetaxel+Aflibercept	0.98 [0.78 − 1.2]
Docetaxel+Selumetinib	0.98 [0.76 − 1.25]
Erlotinib	0.98 [0.86 − 1.11]
Pemetrexed	1 [0.88 − 1.14]
Pemetrexed+Cetuximab	1.01 [0.8 − 1.28]
Vandetanib	1.01 [0.85 − 1.23]
Icotinib	1.02 [0.8 − 1.3]
Pemetrexed+Nintedanib	1.02 [0.79 − 1.3]
Dacomitinib	1.04 [0.87 − 1.22]
Topotecan	1.06 [0.86 − 1.28]
AXL1717	1.06 [0.88 − 1.27]
Erlotinib+Figitumumab	1.06 [0.83 − 1.37]
Erlotinib+Pazopanib	1.07 [0.72 − 1.59]
Erlotinib+Onartuzumab	1.09 [0.83 − 1.44]
Docetaxel+Cetuximab	1.1 [0.84 − 1.43]
Paclitaxel	1.12 [0.92 − 1.38]
Placebo	1.18 [1.03 − 1.36]
Paclitaxel+Bevacizumab	1.19 [0.84 − 1.76]
Erlotinib+Everolimus	1.28 [0.67 − 2.3]
Sunitinib	1.41 [0.85 − 2.3]
Docetaxel+PX−866	1.45 [0.86 − 2.38]
Cetuximab	1.65 [0.8 − 3.28]
Pemetrexed+Matuzumab	1.67 [0.96 − 2.89]
Erlotinib+Dalotuzumab	1.68 [0.83 − 3.49]
Pemetrexed+Sunitinib	1.99 [1.17 − 3.39]

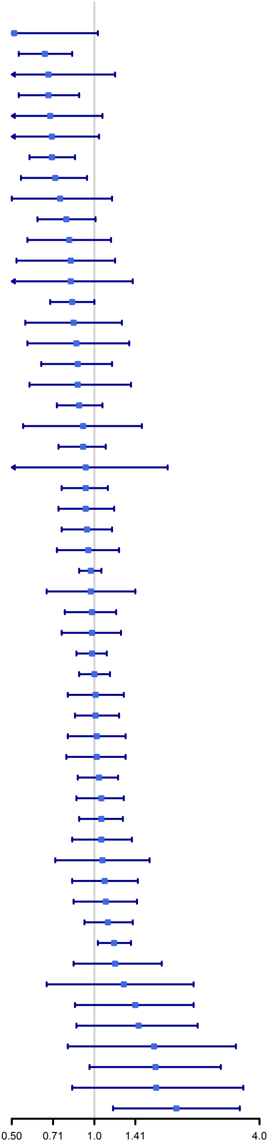

Treatments vs. Nivolumab	HR[Crl 95%]
Erlotinib+Cabozantinib	0.77 [0.38 − 1.56]
Pembrolizumab	1.03 [0.77 − 1.4]
Cabozantinib	1.03 [0.51 − 1.89]
Docetaxel+Tamoxifen	1.04 [0.64 − 1.65]
Erlotinib+R1507	1.05 [0.69 − 1.67]
Atezolizumab	1.06 [0.82 − 1.37]
Pemetrexed+Erlotinib	1.08 [0.77 − 1.5]
Docetaxel+Erlotinib	1.13 [0.71 − 1.85]
Afatinib	1.19 [0.89 − 1.64]
Nab−Paclitaxel	1.22 [0.77 − 1.88]
Pralatrexate	1.22 [0.83 − 1.84]
Tegafur/uracil+Gefitinib	1.23 [0.69 − 2.19]
Docetaxel+Ramucirumab	1.25 [0.99 − 1.61]
Entinostat+Erlotinib	1.26 [0.82 − 1.95]
Gefitinib+Nimotuzumab	1.29 [0.82 − 2.13]
Pemetrexed+Vandetanib	1.3 [0.94 − 1.86]
Docetaxel+Vandetanib	1.33 [1.02 − 1.72]
Erlotinib+Sorafenib	1.34 [0.8 − 2.14]
Docetaxel+Nintedanib	1.37 [1.08 − 1.77]
Erlotinib+Fulvestrant	1.38 [0.8 − 2.34]
Erlotinib+Tivantinib	1.39 [1.07 − 1.84]
Trametinib	1.4 [0.67 − 2.84]
Vinflunine	1.41 [1.07 − 1.89]
Erlotinib+Sunitinib	1.41 [1.07 − 1.9]
Erlotinib+Bevacizumab	1.43 [1.03 − 2]
Docetaxel	1.45 [1.21 − 1.77]
Docetaxel+Aflibercept	1.47 [1.13 − 1.94]
Amrubicin	1.47 [0.98 − 2.22]
Docetaxel+Selumetinib	1.47 [1.1 − 1.96]
Erlotinib	1.48 [1.17 − 1.87]
Pemetrexed	1.5 [1.2 − 1.91]
Gefitinib	1.51 [1.21 − 1.89]
Pemetrexed+Cetuximab	1.53 [1.15 − 2.08]
Vandetanib	1.53 [1.18 − 2.05]
Icotinib	1.54 [1.12 − 2.16]
Pemetrexed+Nintedanib	1.54 [1.14 − 2.09]
Dacomitinib	1.56 [1.21 − 2.04]
AXL1717	1.6 [1.27 − 2.05]
Topotecan	1.6 [1.24 − 2.07]
Erlotinib+Figitumumab	1.61 [1.18 − 2.23]
Erlotinib+Pazopanib	1.62 [1.07 − 2.52]
Erlotinib+Onartuzumab	1.64 [1.19 − 2.34]
Docetaxel+Cetuximab	1.66 [1.23 − 2.28]
Paclitaxel	1.69 [1.31 − 2.24]
Placebo	1.78 [1.39 − 2.29]
Paclitaxel+Bevacizumab	1.8 [1.21 − 2.73]
Erlotinib+Everolimus	1.93 [0.99 − 3.57]
Sunitinib	2.11 [1.25 − 3.62]
Docetaxel+PX−866	2.2 [1.29 − 3.65]
Cetuximab	2.45 [1.19 − 5.16]
Pemetrexed+Matuzumab	2.51 [1.34 − 4.56]
Erlotinib+Dalotuzumab	2.53 [1.23 − 5.39]
Pemetrexed+Sunitinib	3.01 [1.72 − 5.31]

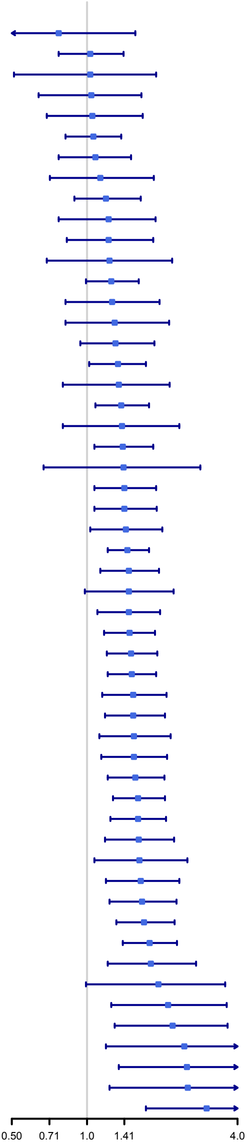

Treatments vs. Pembrolizumab	HR[CrI 95%]
Erlotinib+Cabozantinib	0.74 [0.37 − 1.51]
Nivolumab	0.97 [0.71 − 1.3]
Cabozantinib	0.99 [0.47 − 1.82]
Docetaxel+Tamoxifen	1 [0.62 − 1.64]
Erlotinib+R1507	1.02 [0.65 − 1.64]
Atezolizumab	1.03 [0.77 − 1.36]
Pemetrexed+Erlotinib	1.05 [0.73 − 1.48]
Docetaxel+Erlotinib	1.09 [0.69 − 1.8]
Afatinib	1.16 [0.83 − 1.62]
Nab−Paclitaxel	1.19 [0.7 − 1.85]
Tegafur/uracil+Gefitinib	1.19 [0.65 − 2.13]
Pralatrexate	1.19 [0.77 − 1.8]
Docetaxel+Ramucirumab	1.21 [0.91 − 1.61]
Entinostat+Erlotinib	1.23 [0.76 − 1.92]
Gefitinib+Nimotuzumab	1.25 [0.75 − 2.08]
Pemetrexed+Vandetanib	1.27 [0.87 − 1.83]
Erlotinib+Sorafenib	1.28 [0.78 − 2.15]
Docetaxel+Vandetanib	1.29 [0.96 − 1.71]
Erlotinib+Fulvestrant	1.32 [0.75 − 2.31]
Docetaxel+Nintedanib	1.33 [0.99 − 1.76]
Trametinib	1.34 [0.65 − 2.84]
Erlotinib+Tivantinib	1.35 [1 − 1.82]
Vinflunine	1.36 [0.99 − 1.88]
Erlotinib+Sunitinib	1.37 [1 − 1.89]
Erlotinib+Bevacizumab	1.39 [0.97 − 1.96]
Docetaxel	1.41 [1.11 − 1.78]
Amrubicin	1.42 [0.93 − 2.2]
Docetaxel+Aflibercept	1.42 [1.05 − 1.92]
Erlotinib	1.43 [1.09 − 1.87]
Docetaxel+Selumetinib	1.43 [1.02 − 1.96]
Pemetrexed	1.46 [1.12 − 1.9]
Gefitinib	1.46 [1.13 − 1.88]
Vandetanib	1.48 [1.1 − 2.02]
Pemetrexed+Cetuximab	1.48 [1.06 − 2.04]
Pemetrexed+Nintedanib	1.49 [1.07 − 2.07]
Icotinib	1.5 [1.05 − 2.12]
Dacomitinib	1.51 [1.12 − 2.02]
Topotecan	1.55 [1.14 − 2.06]
AXL1717	1.55 [1.17 − 2.05]
Erlotinib+Figitumumab	1.56 [1.1 − 2.19]
Erlotinib+Pazopanib	1.56 [1 − 2.49]
Erlotinib+Onartuzumab	1.58 [1.11 − 2.28]
Docetaxel+Cetuximab	1.61 [1.14 − 2.26]
Paclitaxel	1.64 [1.22 − 2.21]
Placebo	1.72 [1.3 − 2.27]
Paclitaxel+Bevacizumab	1.74 [1.16 − 2.73]
Erlotinib+Everolimus	1.85 [0.94 − 3.52]
Sunitinib	2.07 [1.17 − 3.51]
Docetaxel+PX−866	2.13 [1.21 − 3.57]
Cetuximab	2.36 [1.13 − 4.99]
Erlotinib+Dalotuzumab	2.44 [1.15 − 5.21]
Pemetrexed+Matuzumab	2.45 [1.29 − 4.47]
Pemetrexed+Sunitinib	2.91 [1.66 − 5.18]

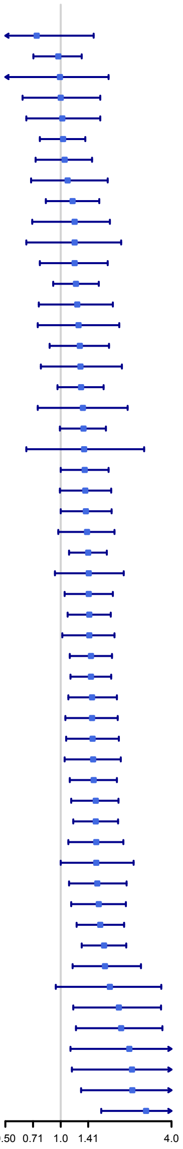

Treatments vs. Atezolizumab	HR[CrI 95%]
Erlotinib+Cabozantinib	0.73 [0.36 − 1.46]
Nivolumab	0.94 [0.73 − 1.22]
Cabozantinib	0.97 [0.48 − 1.76]
Pembrolizumab	0.97 [0.73 − 1.3]
Docetaxel+Tamoxifen	0.98 [0.59 − 1.55]
Erlotinib+R1507	0.99 [0.66 − 1.53]
Pemetrexed+Erlotinib	1.01 [0.74 − 1.39]
Docetaxel+Erlotinib	1.06 [0.63 − 1.72]
Afatinib	1.13 [0.84 − 1.54]
Nab−Paclitaxel	1.15 [0.73 − 1.75]
Pralatrexate	1.16 [0.78 − 1.72]
Tegafur/uracil+Gefitinib	1.17 [0.64 − 2.04]
Docetaxel+Ramucirumab	1.18 [0.92 − 1.5]
Entinostat+Erlotinib	1.19 [0.78 − 1.84]
Gefitinib+Nimotuzumab	1.22 [0.77 − 1.98]
Pemetrexed+Vandetanib	1.23 [0.89 − 1.73]
Erlotinib+Sorafenib	1.25 [0.79 − 2.02]
Docetaxel+Vandetanib	1.25 [0.98 − 1.59]
Docetaxel+Nintedanib	1.29 [1.02 − 1.64]
Erlotinib+Fulvestrant	1.3 [0.78 − 2.2]
Erlotinib+Tivantinib	1.31 [1.02 − 1.7]
Trametinib	1.32 [0.68 − 2.63]
Erlotinib+Sunitinib	1.33 [1.03 − 1.78]
Vinflunine	1.33 [1.01 − 1.75]
Erlotinib+Bevacizumab	1.34 [0.98 − 1.85]
Docetaxel	1.37 [1.15 − 1.62]
Amrubicin	1.38 [0.94 − 2.09]
Docetaxel+Aflibercept	1.39 [1.08 − 1.78]
Erlotinib	1.39 [1.13 − 1.74]
Docetaxel+Selumetinib	1.39 [1.06 − 1.83]
Pemetrexed	1.42 [1.15 − 1.77]
Gefitinib	1.42 [1.17 − 1.74]
Pemetrexed+Cetuximab	1.44 [1.09 − 1.93]
Vandetanib	1.44 [1.1 − 1.91]
Icotinib	1.45 [1.07 − 2]
Pemetrexed+Nintedanib	1.45 [1.08 − 1.95]
Dacomitinib	1.47 [1.16 − 1.89]
Topotecan	1.51 [1.14 − 1.91]
Erlotinib+Figitumumab	1.51 [1.13 − 2.06]
AXL1717	1.51 [1.21 − 1.89]
Erlotinib+Pazopanib	1.52 [0.99 − 2.38]
Erlotinib+Onartuzumab	1.55 [1.13 − 2.15]
Docetaxel+Cetuximab	1.56 [1.15 − 2.11]
Paclitaxel	1.59 [1.26 − 2.06]
Placebo	1.67 [1.35 − 2.12]
Paclitaxel+Bevacizumab	1.7 [1.16 − 2.55]
Erlotinib+Everolimus	1.8 [0.94 − 3.39]
Sunitinib	2.01 [1.19 − 3.43]
Docetaxel+PX−866	2.07 [1.2 − 3.44]
Cetuximab	2.34 [1.12 − 4.83]
Pemetrexed+Matuzumab	2.35 [1.31 − 4.28]
Erlotinib+Dalotuzumab	2.41 [1.17 − 5]
Pemetrexed+Sunitinib	2.8 [1.64 − 4.93]

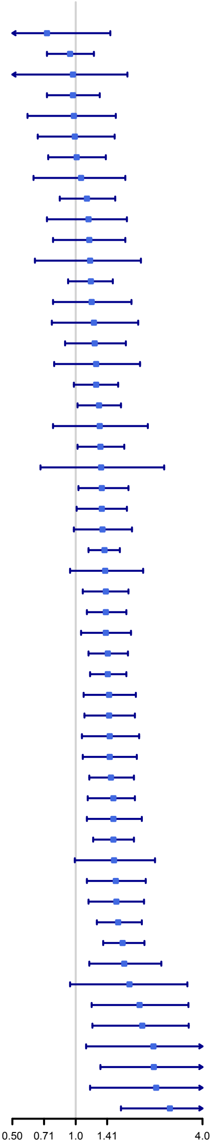

Treatments vs. Docetaxel+Nintedanib	HR[CrI 95%]
Erlotinib+Cabozantinib	0.57 [0.28 − 1.13]
Nivolumab	0.73 [0.56 − 0.93]
Cabozantinib	0.75 [0.37 − 1.36]
Pembrolizumab	0.75 [0.57 − 1.01]
Docetaxel+Tamoxifen	0.76 [0.48 − 1.2]
Erlotinib+R1507	0.77 [0.51 − 1.2]
Atezolizumab	0.78 [0.61 − 0.98]
Pemetrexed+Erlotinib	0.79 [0.57 − 1.08]
Docetaxel+Erlotinib	0.82 [0.53 − 1.33]
Afatinib	0.88 [0.65 − 1.19]
Pralatrexate	0.9 [0.6 − 1.35]
Nab−Paclitaxel	0.9 [0.57 − 1.36]
Tegafur/uracil+Gefitinib	0.9 [0.49 − 1.55]
Docetaxel+Ramucirumab	0.91 [0.73 − 1.16]
Entinostat+Erlotinib	0.92 [0.59 − 1.43]
Gefitinib+Nimotuzumab	0.95 [0.59 − 1.53]
Pemetrexed+Vandetanib	0.95 [0.68 − 1.35]
Erlotinib+Sorafenib	0.97 [0.61 − 1.56]
Docetaxel+Vandetanib	0.97 [0.76 − 1.23]
Erlotinib+Fulvestrant	1 [0.6 − 1.7]
Trametinib	1.02 [0.52 − 2.11]
Erlotinib+Tivantinib	1.02 [0.79 − 1.31]
Vinflunine	1.03 [0.78 − 1.35]
Erlotinib+Sunitinib	1.03 [0.79 − 1.38]
Erlotinib+Bevacizumab	1.04 [0.76 − 1.44]
Docetaxel	1.06 [0.9 − 1.25]
Amrubicin	1.07 [0.73 − 1.6]
Docetaxel+Aflibercept	1.08 [0.84 − 1.38]
Erlotinib	1.08 [0.87 − 1.34]
Docetaxel+Selumetinib	1.08 [0.81 − 1.4]
Gefitinib	1.1 [0.91 − 1.35]
Pemetrexed	1.1 [0.89 − 1.36]
Pemetrexed+Cetuximab	1.12 [0.84 − 1.49]
Vandetanib	1.12 [0.88 − 1.46]
Pemetrexed+Nintedanib	1.12 [0.84 − 1.51]
Icotinib	1.13 [0.82 − 1.53]
Dacomitinib	1.14 [0.89 − 1.46]
Topotecan	1.17 [0.91 − 1.47]
AXL1717	1.17 [0.93 − 1.46]
Erlotinib+Figitumumab	1.18 [0.87 − 1.59]
Erlotinib+Pazopanib	1.18 [0.77 − 1.85]
Erlotinib+Onartuzumab	1.2 [0.87 − 1.66]
Docetaxel+Cetuximab	1.22 [0.89 − 1.63]
Paclitaxel	1.23 [0.98 − 1.6]
Placebo	1.29 [1.04 − 1.63]
Paclitaxel+Bevacizumab	1.31 [0.89 − 1.99]
Erlotinib+Everolimus	1.4 [0.73 − 2.64]
Sunitinib	1.55 [0.91 − 2.64]
Docetaxel+PX−866	1.61 [0.94 − 2.63]
Cetuximab	1.81 [0.86 − 3.74]
Pemetrexed+Matuzumab	1.84 [1.02 − 3.31]
Erlotinib+Dalotuzumab	1.85 [0.9 − 3.86]
Pemetrexed+Sunitinib	2.19 [1.26 − 3.84]

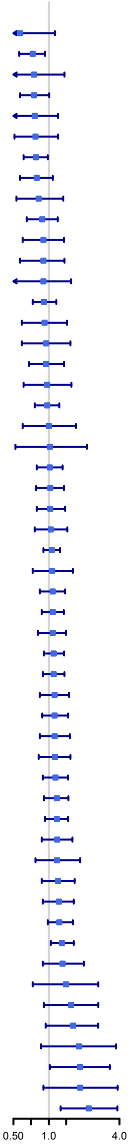

Treatments vs. Docetaxel+Ramucirumab	HR[CrI 95%]
Erlotinib+Cabozantinib	0.61 [0.31 − 1.24]
Nivolumab	0.8 [0.62 − 1.01]
Cabozantinib	0.82 [0.4 − 1.48]
Pembrolizumab	0.83 [0.62 − 1.1]
Docetaxel+Tamoxifen	0.83 [0.52 − 1.31]
Erlotinib+R1507	0.85 [0.56 − 1.31]
Atezolizumab	0.85 [0.66 − 1.09]
Pemetrexed+Erlotinib	0.86 [0.63 − 1.18]
Docetaxel+Erlotinib	0.9 [0.59 − 1.45]
Afatinib	0.96 [0.71 − 1.28]
Nab−Paclitaxel	0.98 [0.6 − 1.48]
Pralatrexate	0.99 [0.66 − 1.45]
Tegafur/uracil+Gefitinib	0.99 [0.54 − 1.7]
Entinostat+Erlotinib	1.01 [0.66 − 1.55]
Gefitinib+Nimotuzumab	1.04 [0.66 − 1.66]
Pemetrexed+Vandetanib	1.05 [0.74 − 1.46]
Erlotinib+Sorafenib	1.06 [0.66 − 1.72]
Docetaxel+Vandetanib	1.06 [0.84 − 1.34]
Docetaxel+Nintedanib	1.1 [0.86 − 1.38]
Erlotinib+Fulvestrant	1.1 [0.63 − 1.84]
Trametinib	1.11 [0.55 − 2.3]
Erlotinib+Tivantinib	1.11 [0.86 − 1.44]
Vinflunine	1.13 [0.86 − 1.48]
Erlotinib+Sunitinib	1.13 [0.86 − 1.48]
Erlotinib+Bevacizumab	1.14 [0.84 − 1.55]
Docetaxel	1.17 [0.99 − 1.37]
Docetaxel+Aflibercept	1.18 [0.91 − 1.5]
Amrubicin	1.18 [0.79 − 1.75]
Erlotinib	1.18 [0.96 − 1.46]
Docetaxel+Selumetinib	1.18 [0.89 − 1.53]
Gefitinib	1.21 [1 − 1.46]
Pemetrexed	1.21 [0.98 − 1.48]
Vandetanib	1.22 [0.96 − 1.6]
Pemetrexed+Cetuximab	1.22 [0.9 − 1.61]
Pemetrexed+Nintedanib	1.23 [0.92 − 1.64]
Icotinib	1.23 [0.92 − 1.68]
Dacomitinib	1.25 [0.98 − 1.62]
Topotecan	1.28 [1 − 1.61]
Erlotinib+Figitumumab	1.28 [0.95 − 1.76]
AXL1717	1.28 [1.02 − 1.6]
Erlotinib+Pazopanib	1.29 [0.85 − 1.99]
Erlotinib+Onartuzumab	1.31 [0.96 − 1.81]
Docetaxel+Cetuximab	1.33 [0.98 − 1.78]
Paclitaxel	1.35 [1.07 − 1.74]
Placebo	1.42 [1.15 − 1.78]
Paclitaxel+Bevacizumab	1.44 [0.99 − 2.17]
Erlotinib+Everolimus	1.52 [0.79 − 2.85]
Sunitinib	1.71 [1.01 − 2.83]
Docetaxel+PX−866	1.77 [1.02 − 2.92]
Cetuximab	1.97 [0.95 − 4.05]
Erlotinib+Dalotuzumab	2.02 [1.01 − 4.25]
Pemetrexed+Matuzumab	2.02 [1.11 − 3.61]
Pemetrexed+Sunitinib	2.4 [1.39 − 4.2]

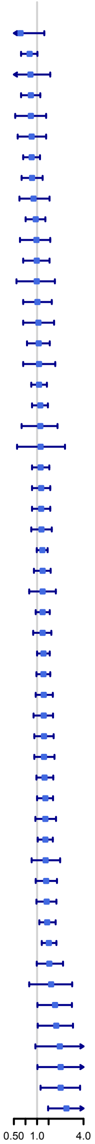

Treatments vs. Afatinib	HR[CrI 95%]
Erlotinib+Cabozantinib	0.64 [0.32 − 1.3]
Nivolumab	0.84 [0.61 − 1.13]
Cabozantinib	0.86 [0.42 − 1.52]
Pembrolizumab	0.86 [0.62 − 1.2]
Docetaxel+Tamoxifen	0.87 [0.52 − 1.41]
Erlotinib+R1507	0.88 [0.59 − 1.35]
Atezolizumab	0.88 [0.65 − 1.19]
Pemetrexed+Erlotinib	0.9 [0.64 − 1.26]
Docetaxel+Erlotinib	0.94 [0.6 − 1.5]
Nab−Paclitaxel	1.02 [0.64 − 1.57]
Tegafur/uracil+Gefitinib	1.03 [0.58 − 1.8]
Pralatrexate	1.03 [0.69 − 1.51]
Docetaxel+Ramucirumab	1.04 [0.78 − 1.4]
Entinostat+Erlotinib	1.06 [0.69 − 1.61]
Gefitinib+Nimotuzumab	1.08 [0.67 − 1.79]
Pemetrexed+Vandetanib	1.09 [0.76 − 1.56]
Erlotinib+Sorafenib	1.11 [0.69 − 1.77]
Docetaxel+Vandetanib	1.11 [0.83 − 1.49]
Docetaxel+Nintedanib	1.14 [0.84 − 1.54]
Erlotinib+Fulvestrant	1.14 [0.69 − 1.92]
Trametinib	1.16 [0.56 − 2.42]
Erlotinib+Tivantinib	1.17 [0.9 − 1.48]
Erlotinib+Sunitinib	1.18 [0.91 − 1.56]
Vinflunine	1.18 [0.85 − 1.62]
Erlotinib+Bevacizumab	1.19 [0.88 − 1.62]
Docetaxel	1.22 [0.95 − 1.55]
Amrubicin	1.22 [0.79 − 1.92]
Docetaxel+Aflibercept	1.23 [0.9 − 1.65]
Docetaxel+Selumetinib	1.23 [0.89 − 1.73]
Erlotinib	1.23 [1.01 − 1.52]
Gefitinib	1.26 [0.99 − 1.6]
Pemetrexed	1.26 [0.99 − 1.62]
Pemetrexed+Cetuximab	1.27 [0.94 − 1.76]
Vandetanib	1.27 [1 − 1.66]
Pemetrexed+Nintedanib	1.28 [0.93 − 1.78]
Icotinib	1.29 [0.92 − 1.81]
Dacomitinib	1.3 [1.02 − 1.68]
Topotecan	1.33 [0.99 − 1.78]
Erlotinib+Figitumumab	1.34 [1.01 − 1.81]
AXL1717	1.34 [0.99 − 1.79]
Erlotinib+Pazopanib	1.35 [0.89 − 2.04]
Erlotinib+Onartuzumab	1.37 [1.01 − 1.87]
Docetaxel+Cetuximab	1.39 [0.97 − 1.98]
Paclitaxel	1.41 [1.06 − 1.92]
Placebo	1.48 [1.17 − 1.9]
Paclitaxel+Bevacizumab	1.5 [1 − 2.33]
Erlotinib+Everolimus	1.6 [0.83 − 2.96]
Sunitinib	1.77 [1.02 − 2.99]
Docetaxel+PX−866	1.83 [1.04 − 3.15]
Cetuximab	2.06 [0.97 − 4.26]
Erlotinib+Dalotuzumab	2.1 [1 − 4.5]
Pemetrexed+Matuzumab	2.1 [1.15 − 3.78]
Pemetrexed+Sunitinib	2.49 [1.43 − 4.4]

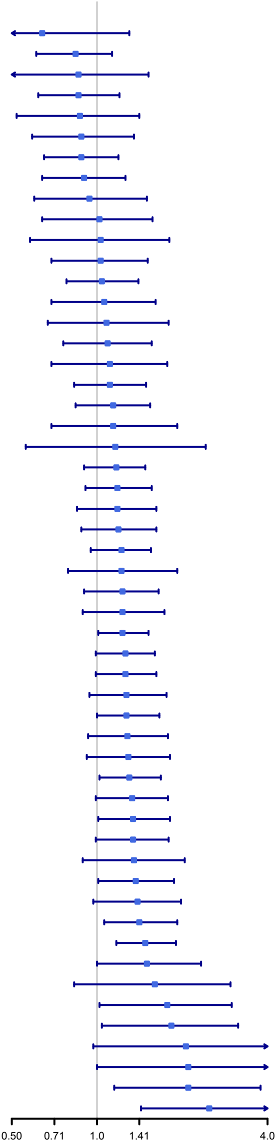

Treatments vs. Cabozantinib	HR[CrI 95%]
Erlotinib+Cabozantinib	0.76 [0.32 − 1.92]
Nivolumab	0.97 [0.53 − 1.98]
Pembrolizumab	1.01 [0.55 − 2.11]
Erlotinib+R1507	1.03 [0.52 − 2.24]
Docetaxel+Tamoxifen	1.03 [0.5 − 2.11]
Atezolizumab	1.03 [0.57 − 2.09]
Pemetrexed+Erlotinib	1.05 [0.57 − 2.12]
Docetaxel+Erlotinib	1.11 [0.57 − 2.31]
Afatinib	1.17 [0.66 − 2.38]
Nab−Paclitaxel	1.18 [0.6 − 2.47]
Pralatrexate	1.2 [0.64 − 2.5]
Tegafur/uracil+Gefitinib	1.21 [0.56 − 2.83]
Docetaxel+Ramucirumab	1.21 [0.68 − 2.48]
Entinostat+Erlotinib	1.22 [0.63 − 2.61]
Gefitinib+Nimotuzumab	1.27 [0.62 − 2.78]
Pemetrexed+Vandetanib	1.27 [0.69 − 2.52]
Docetaxel+Vandetanib	1.29 [0.72 − 2.61]
Erlotinib+Sorafenib	1.3 [0.64 − 2.84]
Docetaxel+Nintedanib	1.33 [0.74 − 2.67]
Erlotinib+Fulvestrant	1.35 [0.67 − 2.9]
Erlotinib+Tivantinib	1.35 [0.77 − 2.73]
Vinflunine	1.37 [0.75 − 2.8]
Erlotinib+Sunitinib	1.37 [0.77 − 2.75]
Erlotinib+Bevacizumab	1.39 [0.77 − 2.82]
Trametinib	1.39 [0.53 − 3.53]
Docetaxel	1.42 [0.8 − 2.82]
Docetaxel+Selumetinib	1.43 [0.77 − 2.92]
Erlotinib	1.43 [0.83 − 2.8]
Docetaxel+Aflibercept	1.43 [0.79 − 2.93]
Amrubicin	1.44 [0.73 − 3]
Gefitinib	1.47 [0.84 − 2.91]
Pemetrexed	1.47 [0.84 − 2.91]
Vandetanib	1.49 [0.85 − 3.02]
Pemetrexed+Cetuximab	1.49 [0.82 − 2.89]
Pemetrexed+Nintedanib	1.49 [0.83 − 3.03]
Icotinib	1.49 [0.82 − 3.14]
Dacomitinib	1.52 [0.87 − 2.99]
Topotecan	1.55 [0.86 − 3.19]
AXL1717	1.56 [0.87 − 3.14]
Erlotinib+Figitumumab	1.56 [0.88 − 3.16]
Erlotinib+Pazopanib	1.57 [0.85 − 3.15]
Erlotinib+Onartuzumab	1.6 [0.89 − 3.37]
Docetaxel+Cetuximab	1.61 [0.86 − 3.31]
Paclitaxel	1.64 [0.91 − 3.35]
Placebo	1.72 [0.99 − 3.43]
Paclitaxel+Bevacizumab	1.76 [0.9 − 3.86]
Erlotinib+Everolimus	1.84 [0.77 − 4.47]
Sunitinib	2.1 [0.93 − 4.61]
Docetaxel+PX−866	2.14 [0.99 − 4.78]
Cetuximab	2.42 [0.99 − 5.88]
Pemetrexed+Matuzumab	2.47 [1.11 − 5.5]
Erlotinib+Dalotuzumab	2.49 [1 − 6.46]
Pemetrexed+Sunitinib	2.91 [1.36 − 6.5]

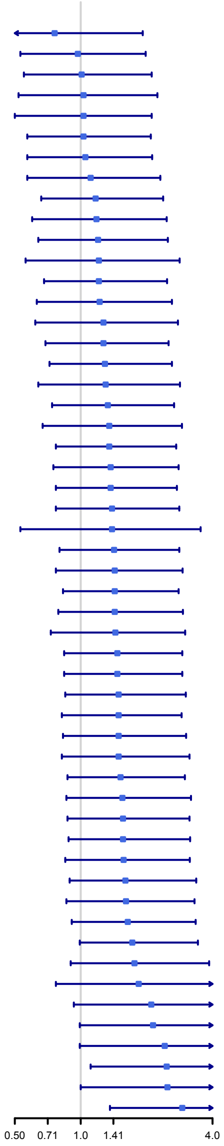

Treatments vs. Erlotinib+Cabozantinib	HR[CrI 95%]
Nivolumab	1.29 [0.64 − 2.62]
Cabozantinib	1.31 [0.52 − 3.08]
Pembrolizumab	1.35 [0.66 − 2.73]
Docetaxel+Tamoxifen	1.35 [0.55 − 3.04]
Atezolizumab	1.37 [0.69 − 2.75]
Pemetrexed+Erlotinib	1.38 [0.67 − 2.9]
Erlotinib+R1507	1.38 [0.65 − 2.89]
Docetaxel+Erlotinib	1.47 [0.59 − 3.25]
Afatinib	1.55 [0.77 − 3.1]
Nab−Paclitaxel	1.57 [0.71 − 3.51]
Tegafur/uracil+Gefitinib	1.58 [0.69 − 3.78]
Pralatrexate	1.58 [0.77 − 3.29]
Docetaxel+Ramucirumab	1.63 [0.81 − 3.24]
Entinostat+Erlotinib	1.63 [0.78 − 3.44]
Pemetrexed+Vandetanib	1.68 [0.82 − 3.54]
Gefitinib+Nimotuzumab	1.7 [0.71 − 3.83]
Erlotinib+Sorafenib	1.72 [0.75 − 3.78]
Docetaxel+Vandetanib	1.73 [0.83 − 3.47]
Docetaxel+Nintedanib	1.77 [0.88 − 3.56]
Erlotinib+Fulvestrant	1.8 [0.75 − 4.1]
Erlotinib+Tivantinib	1.81 [0.92 − 3.53]
Trametinib	1.82 [0.7 − 4.93]
Vinflunine	1.82 [0.89 − 3.73]
Erlotinib+Sunitinib	1.83 [0.93 − 3.63]
Erlotinib+Bevacizumab	1.85 [0.91 − 3.72]
Docetaxel	1.9 [0.95 − 3.74]
Amrubicin	1.91 [0.88 − 4.1]
Docetaxel+Selumetinib	1.91 [0.93 − 3.99]
Docetaxel+Aflibercept	1.92 [0.93 − 3.87]
Erlotinib	1.93 [0.97 − 3.69]
Pemetrexed	1.96 [0.98 − 3.9]
Gefitinib	1.96 [0.97 − 3.84]
Pemetrexed+Cetuximab	1.97 [0.98 − 4.02]
Vandetanib	1.99 [1 − 3.9]
Pemetrexed+Nintedanib	1.99 [0.97 − 4.07]
Icotinib	2.01 [0.93 − 4.04]
Dacomitinib	2.02 [1.02 − 3.97]
Erlotinib+Pazopanib	2.08 [0.97 − 4.48]
AXL1717	2.08 [1.03 − 4.17]
Erlotinib+Figitumumab	2.08 [1.02 − 4.17]
Topotecan	2.09 [1.02 − 4.2]
Erlotinib+Onartuzumab	2.14 [1.03 − 4.29]
Docetaxel+Cetuximab	2.17 [1.04 − 4.43]
Paclitaxel	2.22 [1.09 − 4.43]
Placebo	2.32 [1.15 − 4.51]
Paclitaxel+Bevacizumab	2.35 [1.06 − 5.14]
Erlotinib+Everolimus	2.43 [1.04 − 5.76]
Sunitinib	2.74 [1.24 − 6.19]
Docetaxel+PX−866	2.79 [1.24 − 6.85]
Cetuximab	3.17 [1.21 − 8]
Pemetrexed+Matuzumab	3.26 [1.32 − 8.4]
Erlotinib+Dalotuzumab	3.31 [1.17 − 8.78]
Pemetrexed+Sunitinib	3.82 [1.7 − 9.67]

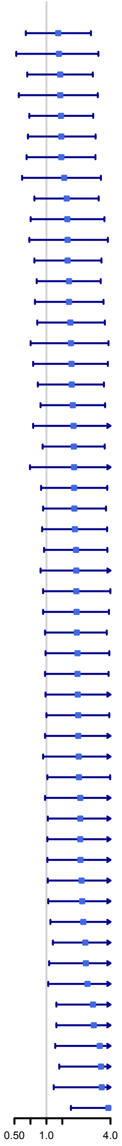

**Treatments vs. Placebo**	**HR[CrI 95%]**
Erlotinib+Cabozantinib	0.43 [0.22 − 0.87]
Nivolumab	0.56 [0.44 − 0.72]
Cabozantinib	0.58 [0.29 − 1.01]
Pembrolizumab	0.58 [0.44 − 0.77]
Docetaxel+Tamoxifen	0.58 [0.37 − 0.92]
Erlotinib+R1507	0.59 [0.4 − 0.89]
Atezolizumab	0.6 [0.47 − 0.74]
Pemetrexed+Erlotinib	0.61 [0.45 − 0.82]
Docetaxel+Erlotinib	0.63 [0.42 − 0.98]
Afatinib	0.67 [0.53 − 0.85]
Pralatrexate	0.69 [0.48 − 0.98]
Nab−Paclitaxel	0.69 [0.44 − 1.02]
Tegafur/uracil+Gefitinib	0.7 [0.39 − 1.18]
Docetaxel+Ramucirumab	0.7 [0.56 − 0.87]
Entinostat+Erlotinib	0.71 [0.47 − 1.06]
Gefitinib+Nimotuzumab	0.73 [0.47 − 1.16]
Pemetrexed+Vandetanib	0.74 [0.54 − 1]
Erlotinib+Sorafenib	0.74 [0.48 − 1.16]
Docetaxel+Vandetanib	0.75 [0.59 − 0.93]
Docetaxel+Nintedanib	0.77 [0.61 − 0.96]
Erlotinib+Fulvestrant	0.78 [0.47 − 1.26]
Trametinib	0.78 [0.4 − 1.59]
Erlotinib+Tivantinib	0.79 [0.64 − 0.95]
Vinflunine	0.79 [0.61 − 1.03]
Erlotinib+Sunitinib	0.8 [0.64 − 0.98]
Erlotinib+Bevacizumab	0.8 [0.61 − 1.05]
Docetaxel	0.82 [0.7 − 0.95]
Amrubicin	0.83 [0.56 − 1.22]
Docetaxel+Aflibercept	0.83 [0.64 − 1.04]
Docetaxel+Selumetinib	0.83 [0.63 − 1.09]
Erlotinib	0.83 [0.72 − 0.95]
Pemetrexed	0.85 [0.72 − 1]
Gefitinib	0.85 [0.74 − 0.97]
Pemetrexed+Cetuximab	0.86 [0.66 − 1.11]
Vandetanib	0.86 [0.71 − 1.06]
Icotinib	0.87 [0.66 − 1.14]
Pemetrexed+Nintedanib	0.87 [0.65 − 1.12]
Dacomitinib	0.88 [0.74 − 1.03]
Topotecan	0.9 [0.7 − 1.12]
Erlotinib+Figitumumab	0.9 [0.7 − 1.17]
AXL1717	0.91 [0.72 − 1.11]
Erlotinib+Pazopanib	0.91 [0.61 − 1.34]
Erlotinib+Onartuzumab	0.92 [0.7 − 1.22]
Docetaxel+Cetuximab	0.93 [0.69 − 1.24]
Paclitaxel	0.95 [0.76 − 1.2]
Paclitaxel+Bevacizumab	1.01 [0.7 − 1.52]
Erlotinib+Everolimus	1.09 [0.56 − 1.94]
Sunitinib	1.2 [0.7 − 1.97]
Docetaxel+PX−866	1.24 [0.72 − 2.04]
Cetuximab	1.4 [0.67 − 2.79]
Pemetrexed+Matuzumab	1.42 [0.8 − 2.48]
Erlotinib+Dalotuzumab	1.43 [0.69 − 2.99]
Pemetrexed+Sunitinib	1.69 [0.98 − 2.89]

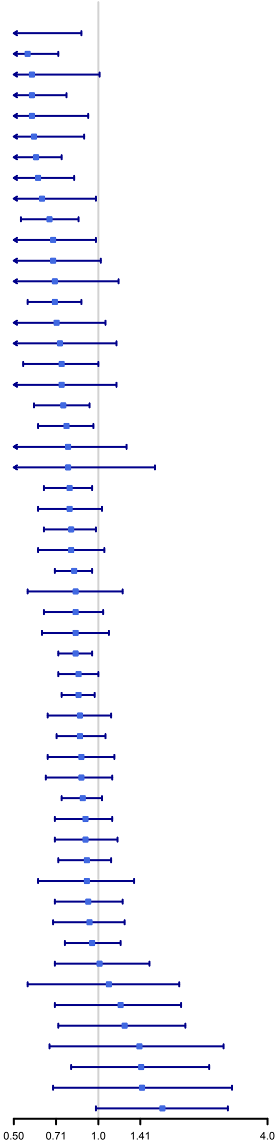

(b) Progression free survival

Treatments vs. Docetaxel	HR[CrI 95%]
Erlotinib+Cabozantinib	0.39 [0.18 − 0.84]
Cabozantinib	0.42 [0.2 − 0.87]
Paclitaxel+Bevacizumab	0.62 [0.39 − 0.98]
Erlotinib+Pazopanib	0.63 [0.38 − 1.03]
Pemetrexed+Erlotinib	0.63 [0.46 − 0.89]
Erlotinib+Bevacizumab	0.66 [0.44 − 1]
Docetaxel+Tamoxifen	0.67 [0.41 − 1.09]
Tegafur/uracil+Gefitinib	0.67 [0.39 − 1.14]
Docetaxel+Vandetanib	0.76 [0.56 − 1]
Erlotinib+Tivantinib	0.78 [0.58 − 1.05]
Nivolumab	0.78 [0.59 − 1.02]
Docetaxel+Ramucirumab	0.78 [0.6 − 1.03]
Erlotinib+Everolimus	0.82 [0.47 − 1.43]
Docetaxel+Aflibercept	0.82 [0.58 − 1.16]
Docetaxel+Selumetinib	0.82 [0.59 − 1.1]
Docetaxel+Erlotinib	0.84 [0.53 − 1.33]
Docetaxel+Nintedanib	0.85 [0.61 − 1.19]
Icotinib	0.87 [0.57 − 1.3]
Afatinib	0.87 [0.58 − 1.32]
Pemetrexed+Nintedanib	0.87 [0.58 − 1.3]
Erlotinib+Sunitinib	0.87 [0.61 − 1.27]
Pembrolizumab	0.88 [0.61 − 1.26]
Pemetrexed+Vandetanib	0.89 [0.6 − 1.34]
Erlotinib+Fulvestrant	0.9 [0.5 − 1.59]
Docetaxel+Cetuximab	0.91 [0.62 − 1.33]
Erlotinib+Sorafenib	0.92 [0.55 − 1.52]
Erlotinib+Dalotuzumab	0.92 [0.46 − 1.85]
Vandetanib	0.92 [0.66 − 1.24]
Nab−Paclitaxel	0.93 [0.57 − 1.5]
Dacomitinib	0.94 [0.68 − 1.27]
Atezolizumab	0.95 [0.73 − 1.23]
Amrubicin	0.96 [0.61 − 1.51]
Pralatrexate	0.97 [0.58 − 1.62]
Erlotinib+R1507	0.99 [0.59 − 1.67]
Vinflunine	1 [0.71 − 1.42]
Gefitinib	1.03 [0.88 − 1.2]
Pemetrexed	1.04 [0.87 − 1.24]
Entinostat+Erlotinib	1.05 [0.62 − 1.78]
Erlotinib	1.06 [0.87 − 1.29]
Gefitinib+Nimotuzumab	1.06 [0.65 − 1.72]
Pemetrexed+Cetuximab	1.07 [0.72 − 1.59]
Erlotinib+Onartuzumab	1.08 [0.76 − 1.55]
Docetaxel+PX−866	1.09 [0.65 − 1.82]
Selumetinib	1.11 [0.58 − 2.15]
Erlotinib+Figitumumab	1.15 [0.76 − 1.72]
Trametinib	1.23 [0.73 − 2.06]
AXL1717	1.3 [0.86 − 1.96]
Pemetrexed+Sunitinib	1.34 [0.78 − 2.34]
Sunitinib	1.45 [0.83 − 2.57]
Pemetrexed+Matuzumab	1.51 [0.83 − 2.76]
Placebo	1.57 [1.11 − 2.2]
Cetuximab	1.8 [0.81 − 3.97]

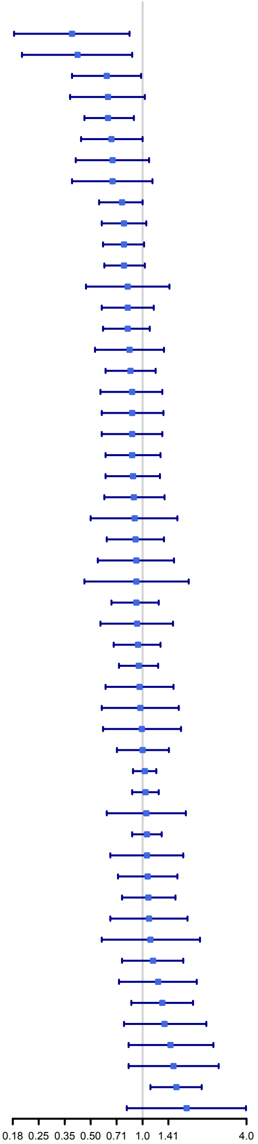

Treatments vs. Pemetrexed	HR[CrI 95%]
Erlotinib+Cabozantinib	0.38 [0.18 − 0.82]
Cabozantinib	0.4 [0.19 − 0.84]
Paclitaxel+Bevacizumab	0.6 [0.36 − 0.98]
Erlotinib+Pazopanib	0.6 [0.36 − 1]
Pemetrexed+Erlotinib	0.61 [0.46 − 0.82]
Erlotinib+Bevacizumab	0.63 [0.42 − 0.97]
Docetaxel+Tamoxifen	0.64 [0.39 − 1.08]
Tegafur/uracil+Gefitinib	0.65 [0.38 − 1.11]
Docetaxel+Vandetanib	0.73 [0.51 − 1.01]
Erlotinib+Tivantinib	0.75 [0.55 − 1.03]
Nivolumab	0.75 [0.54 − 1.03]
Docetaxel+Ramucirumab	0.75 [0.55 − 1.04]
Erlotinib+Everolimus	0.79 [0.45 − 1.38]
Docetaxel+Aflibercept	0.79 [0.54 − 1.16]
Docetaxel+Selumetinib	0.79 [0.55 − 1.1]
Docetaxel+Erlotinib	0.81 [0.51 − 1.29]
Docetaxel+Nintedanib	0.82 [0.56 − 1.2]
Icotinib	0.83 [0.54 − 1.27]
Afatinib	0.84 [0.55 − 1.28]
Pemetrexed+Nintedanib	0.84 [0.58 − 1.21]
Erlotinib+Sunitinib	0.84 [0.58 − 1.23]
Pembrolizumab	0.85 [0.57 − 1.26]
Pemetrexed+Vandetanib	0.86 [0.6 − 1.24]
Erlotinib+Fulvestrant	0.87 [0.48 − 1.55]
Docetaxel+Cetuximab	0.88 [0.58 − 1.33]
Erlotinib+Sorafenib	0.88 [0.52 − 1.48]
Erlotinib+Dalotuzumab	0.88 [0.44 − 1.79]
Vandetanib	0.89 [0.63 − 1.21]
Nab−Paclitaxel	0.89 [0.57 − 1.39]
Dacomitinib	0.9 [0.65 − 1.23]
Atezolizumab	0.91 [0.66 − 1.25]
Amrubicin	0.93 [0.57 − 1.5]
Pralatrexate	0.93 [0.55 − 1.57]
Erlotinib+R1507	0.95 [0.56 − 1.62]
Docetaxel	0.96 [0.81 − 1.14]
Vinflunine	0.96 [0.66 − 1.42]
Gefitinib	0.99 [0.83 − 1.18]
Entinostat+Erlotinib	1.01 [0.6 − 1.72]
Erlotinib	1.02 [0.83 − 1.27]
Gefitinib+Nimotuzumab	1.03 [0.62 − 1.67]
Pemetrexed+Cetuximab	1.03 [0.72 − 1.47]
Erlotinib+Onartuzumab	1.04 [0.73 − 1.51]
Docetaxel+PX−866	1.05 [0.61 − 1.8]
Selumetinib	1.08 [0.57 − 2.02]
Erlotinib+Figitumumab	1.11 [0.73 − 1.68]
Trametinib	1.18 [0.69 − 2.03]
AXL1717	1.25 [0.8 − 1.95]
Pemetrexed+Sunitinib	1.3 [0.77 − 2.19]
Sunitinib	1.4 [0.82 − 2.42]
Pemetrexed+Matuzumab	1.45 [0.82 − 2.6]
Placebo	1.51 [1.06 − 2.15]
Cetuximab	1.73 [0.8 − 3.75]

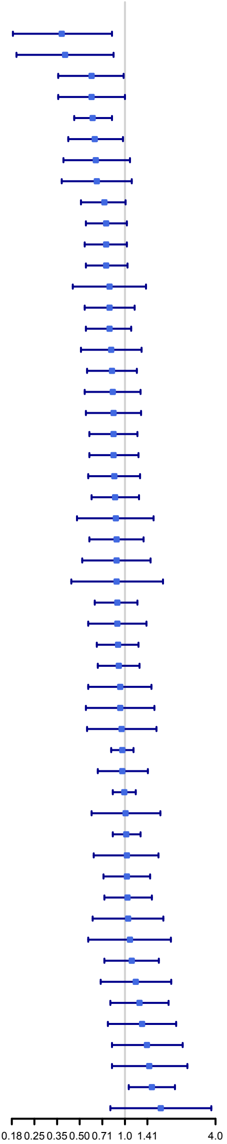

Treatments vs. Erlotinib	HR[CrI 95%]
Erlotinib+Cabozantinib	0.37 [0.18 − 0.78]
Cabozantinib	0.39 [0.19 − 0.8]
Paclitaxel+Bevacizumab	0.58 [0.35 − 0.96]
Erlotinib+Pazopanib	0.59 [0.38 − 0.93]
Pemetrexed+Erlotinib	0.6 [0.42 − 0.86]
Erlotinib+Bevacizumab	0.62 [0.43 − 0.89]
Docetaxel+Tamoxifen	0.63 [0.37 − 1.06]
Tegafur/uracil+Gefitinib	0.63 [0.36 − 1.09]
Docetaxel+Vandetanib	0.71 [0.5 − 0.99]
Erlotinib+Tivantinib	0.73 [0.58 − 0.93]
Nivolumab	0.74 [0.52 − 1.02]
Docetaxel+Ramucirumab	0.74 [0.53 − 1.03]
Erlotinib+Everolimus	0.77 [0.46 − 1.29]
Docetaxel+Aflibercept	0.77 [0.52 − 1.15]
Docetaxel+Selumetinib	0.77 [0.53 − 1.1]
Docetaxel+Erlotinib	0.79 [0.52 − 1.2]
Docetaxel+Nintedanib	0.8 [0.54 − 1.18]
Icotinib	0.81 [0.53 − 1.25]
Afatinib	0.82 [0.57 − 1.19]
Pemetrexed+Nintedanib	0.82 [0.54 − 1.24]
Erlotinib+Sunitinib	0.82 [0.61 − 1.13]
Pembrolizumab	0.83 [0.55 − 1.25]
Pemetrexed+Vandetanib	0.84 [0.55 − 1.29]
Erlotinib+Fulvestrant	0.85 [0.49 − 1.45]
Docetaxel+Cetuximab	0.86 [0.56 − 1.31]
Erlotinib+Sorafenib	0.86 [0.54 − 1.38]
Erlotinib+Dalotuzumab	0.86 [0.44 − 1.69]
Vandetanib	0.87 [0.64 − 1.13]
Nab−Paclitaxel	0.87 [0.53 − 1.43]
Dacomitinib	0.88 [0.68 − 1.11]
Atezolizumab	0.89 [0.64 − 1.23]
Amrubicin	0.91 [0.55 − 1.48]
Pralatrexate	0.91 [0.56 − 1.48]
Erlotinib+R1507	0.93 [0.57 − 1.51]
Docetaxel	0.94 [0.77 − 1.14]
Vinflunine	0.94 [0.63 − 1.4]
Gefitinib	0.97 [0.79 − 1.18]
Pemetrexed	0.98 [0.79 − 1.21]
Entinostat+Erlotinib	0.99 [0.61 − 1.61]
Gefitinib+Nimotuzumab	1 [0.6 − 1.65]
Pemetrexed+Cetuximab	1.01 [0.66 − 1.52]
Erlotinib+Onartuzumab	1.02 [0.76 − 1.38]
Docetaxel+PX−866	1.02 [0.59 − 1.77]
Selumetinib	1.05 [0.54 − 2.04]
Erlotinib+Figitumumab	1.08 [0.75 − 1.54]
Trametinib	1.15 [0.67 − 2]
AXL1717	1.22 [0.77 − 1.92]
Pemetrexed+Sunitinib	1.26 [0.72 − 2.23]
Sunitinib	1.37 [0.77 − 2.46]
Pemetrexed+Matuzumab	1.42 [0.77 − 2.63]
Placebo	1.48 [1.11 − 1.95]
Cetuximab	1.69 [0.76 − 3.76]

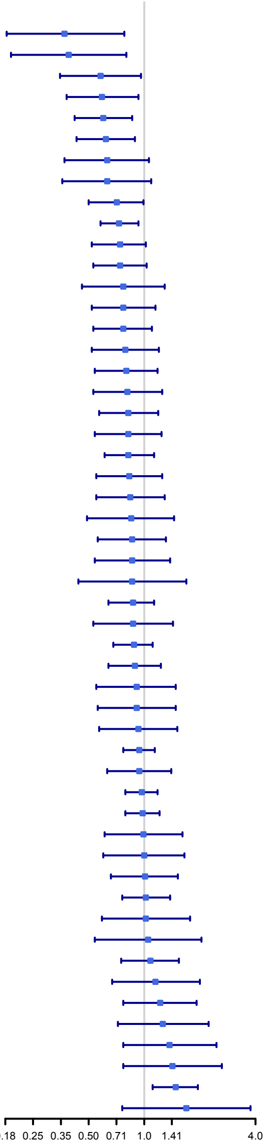

Treatments vs. Gefitinib	HR[CrI 95%]
Erlotinib+Cabozantinib	0.38 [0.18 − 0.82]
Cabozantinib	0.41 [0.19 − 0.85]
Paclitaxel+Bevacizumab	0.6 [0.37 − 0.98]
Erlotinib+Pazopanib	0.61 [0.37 − 1]
Pemetrexed+Erlotinib	0.61 [0.44 − 0.87]
Erlotinib+Bevacizumab	0.64 [0.42 − 0.97]
Docetaxel+Tamoxifen	0.65 [0.39 − 1.09]
Tegafur/uracil+Gefitinib	0.65 [0.39 − 1.09]
Docetaxel+Vandetanib	0.73 [0.52 − 1.01]
Erlotinib+Tivantinib	0.76 [0.56 − 1.03]
Nivolumab	0.76 [0.55 − 1.03]
Docetaxel+Ramucirumab	0.76 [0.56 − 1.04]
Docetaxel+Aflibercept	0.8 [0.55 − 1.17]
Erlotinib+Everolimus	0.8 [0.46 − 1.39]
Docetaxel+Selumetinib	0.8 [0.56 − 1.11]
Docetaxel+Erlotinib	0.81 [0.51 − 1.3]
Docetaxel+Nintedanib	0.82 [0.57 − 1.2]
Icotinib	0.84 [0.57 − 1.23]
Afatinib	0.84 [0.56 − 1.29]
Pemetrexed+Nintedanib	0.84 [0.57 − 1.27]
Erlotinib+Sunitinib	0.85 [0.59 − 1.24]
Pembrolizumab	0.85 [0.58 − 1.27]
Pemetrexed+Vandetanib	0.87 [0.58 − 1.31]
Erlotinib+Fulvestrant	0.88 [0.49 − 1.55]
Docetaxel+Cetuximab	0.88 [0.59 − 1.33]
Erlotinib+Dalotuzumab	0.89 [0.44 − 1.8]
Erlotinib+Sorafenib	0.89 [0.53 − 1.48]
Vandetanib	0.9 [0.66 − 1.19]
Nab−Paclitaxel	0.9 [0.56 − 1.46]
Dacomitinib	0.91 [0.66 − 1.23]
Atezolizumab	0.92 [0.68 − 1.25]
Amrubicin	0.93 [0.58 − 1.51]
Pralatrexate	0.94 [0.56 − 1.59]
Erlotinib+R1507	0.96 [0.57 − 1.63]
Docetaxel	0.97 [0.84 − 1.13]
Vinflunine	0.97 [0.67 − 1.43]
Pemetrexed	1.01 [0.85 − 1.21]
Entinostat+Erlotinib	1.02 [0.6 − 1.73]
Erlotinib	1.03 [0.85 − 1.26]
Gefitinib+Nimotuzumab	1.03 [0.65 − 1.64]
Pemetrexed+Cetuximab	1.04 [0.7 − 1.55]
Erlotinib+Onartuzumab	1.05 [0.74 − 1.52]
Docetaxel+PX−866	1.05 [0.61 − 1.8]
Selumetinib	1.08 [0.56 − 2.1]
Erlotinib+Figitumumab	1.12 [0.74 − 1.68]
Trametinib	1.19 [0.7 − 2.04]
AXL1717	1.26 [0.82 − 1.96]
Pemetrexed+Sunitinib	1.3 [0.76 − 2.28]
Sunitinib	1.41 [0.8 − 2.51]
Pemetrexed+Matuzumab	1.46 [0.8 − 2.69]
Placebo	1.52 [1.08 − 2.15]
Cetuximab	1.74 [0.79 − 3.88]

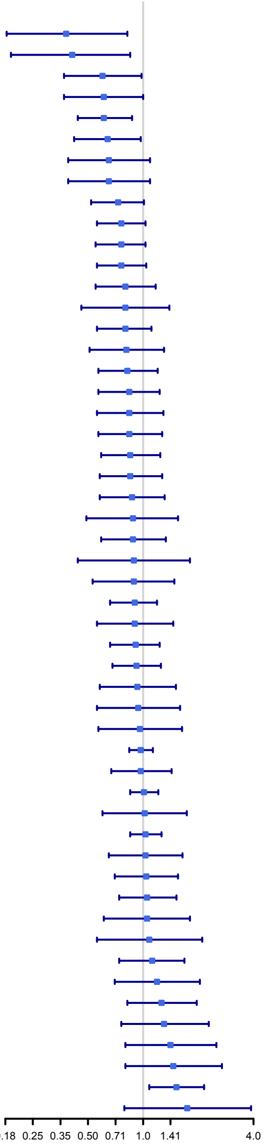

Treatments vs. Nivolumab	HR[CrI 95%]
Erlotinib+Cabozantinib	0.5 [0.22 − 1.13]
Cabozantinib	0.54 [0.24 − 1.17]
Paclitaxel+Bevacizumab	0.79 [0.47 − 1.36]
Erlotinib+Pazopanib	0.8 [0.46 − 1.42]
Pemetrexed+Erlotinib	0.81 [0.53 − 1.26]
Erlotinib+Bevacizumab	0.84 [0.52 − 1.4]
Docetaxel+Tamoxifen	0.85 [0.49 − 1.51]
Tegafur/uracil+Gefitinib	0.86 [0.47 − 1.57]
Docetaxel+Vandetanib	0.97 [0.65 − 1.43]
Erlotinib+Tivantinib	1 [0.67 − 1.52]
Docetaxel+Ramucirumab	1 [0.69 − 1.49]
Docetaxel+Aflibercept	1.05 [0.69 − 1.64]
Erlotinib+Everolimus	1.05 [0.57 − 1.96]
Docetaxel+Selumetinib	1.05 [0.69 − 1.57]
Docetaxel+Erlotinib	1.08 [0.63 − 1.85]
Docetaxel+Nintedanib	1.09 [0.71 − 1.7]
Icotinib	1.11 [0.68 − 1.83]
Afatinib	1.11 [0.68 − 1.85]
Pemetrexed+Nintedanib	1.11 [0.7 − 1.83]
Erlotinib+Sunitinib	1.12 [0.72 − 1.79]
Pembrolizumab	1.13 [0.72 − 1.79]
Pemetrexed+Vandetanib	1.14 [0.71 − 1.88]
Erlotinib+Fulvestrant	1.16 [0.61 − 2.19]
Docetaxel+Cetuximab	1.17 [0.74 − 1.87]
Erlotinib+Sorafenib	1.17 [0.66 − 2.1]
Erlotinib+Dalotuzumab	1.18 [0.56 − 2.51]
Vandetanib	1.18 [0.78 − 1.77]
Nab−Paclitaxel	1.18 [0.69 − 2.08]
Dacomitinib	1.2 [0.79 − 1.81]
Atezolizumab	1.21 [0.83 − 1.79]
Amrubicin	1.23 [0.72 − 2.1]
Pralatrexate	1.24 [0.7 − 2.24]
Erlotinib+R1507	1.27 [0.71 − 2.31]
Vinflunine	1.28 [0.83 − 2.02]
Docetaxel	1.28 [0.98 − 1.7]
Gefitinib	1.32 [0.97 − 1.81]
Pemetrexed	1.33 [0.97 − 1.85]
Entinostat+Erlotinib	1.35 [0.75 − 2.44]
Erlotinib	1.36 [0.98 − 1.92]
Gefitinib+Nimotuzumab	1.36 [0.78 − 2.38]
Pemetrexed+Cetuximab	1.37 [0.86 − 2.23]
Erlotinib+Onartuzumab	1.39 [0.9 − 2.2]
Docetaxel+PX−866	1.39 [0.78 − 2.51]
Selumetinib	1.43 [0.71 − 2.92]
Erlotinib+Figitumumab	1.47 [0.91 − 2.43]
Trametinib	1.57 [0.88 − 2.83]
AXL1717	1.66 [1.02 − 2.74]
Pemetrexed+Sunitinib	1.72 [0.94 − 3.21]
Sunitinib	1.86 [1 − 3.51]
Pemetrexed+Matuzumab	1.93 [1 − 3.77]
Placebo	2.01 [1.3 − 3.13]
Cetuximab	2.3 [1 − 5.34]

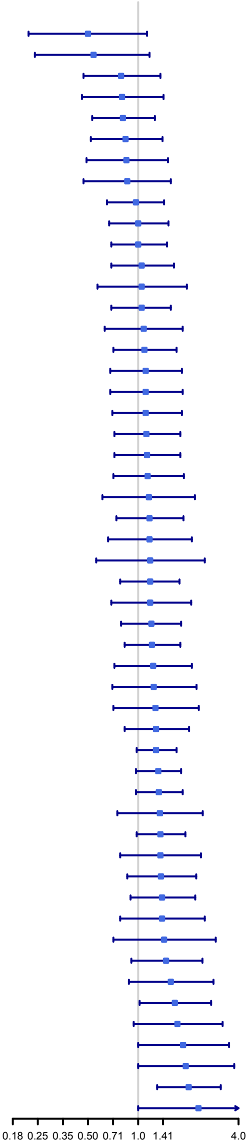

Treatments vs. Pembrolizumab	HR[CrI 95%]
Erlotinib+Cabozantinib	0.45 [0.19 − 1.03]
Cabozantinib	0.47 [0.21 − 1.07]
Paclitaxel+Bevacizumab	0.7 [0.39 − 1.25]
Erlotinib+Pazopanib	0.71 [0.38 − 1.31]
Pemetrexed+Erlotinib	0.72 [0.44 − 1.18]
Erlotinib+Bevacizumab	0.75 [0.43 − 1.3]
Docetaxel+Tamoxifen	0.76 [0.42 − 1.4]
Tegafur/uracil+Gefitinib	0.76 [0.4 − 1.44]
Docetaxel+Vandetanib	0.86 [0.53 − 1.35]
Erlotinib+Tivantinib	0.89 [0.55 − 1.42]
Docetaxel+Ramucirumab	0.89 [0.57 − 1.4]
Nivolumab	0.89 [0.56 − 1.38]
Erlotinib+Everolimus	0.93 [0.48 − 1.8]
Docetaxel+Aflibercept	0.93 [0.57 − 1.53]
Docetaxel+Selumetinib	0.94 [0.57 − 1.47]
Docetaxel+Erlotinib	0.95 [0.53 − 1.71]
Docetaxel+Nintedanib	0.97 [0.59 − 1.59]
Icotinib	0.98 [0.56 − 1.7]
Afatinib	0.99 [0.57 − 1.72]
Pemetrexed+Nintedanib	0.99 [0.58 − 1.7]
Erlotinib+Sunitinib	0.99 [0.6 − 1.67]
Pemetrexed+Vandetanib	1.01 [0.59 − 1.75]
Erlotinib+Fulvestrant	1.02 [0.52 − 2.01]
Docetaxel+Cetuximab	1.03 [0.61 − 1.74]
Erlotinib+Dalotuzumab	1.04 [0.47 − 2.29]
Erlotinib+Sorafenib	1.04 [0.55 − 1.94]
Vandetanib	1.05 [0.64 − 1.65]
Nab−Paclitaxel	1.05 [0.58 − 1.92]
Dacomitinib	1.06 [0.65 − 1.69]
Atezolizumab	1.07 [0.69 − 1.68]
Amrubicin	1.09 [0.61 − 1.95]
Pralatrexate	1.1 [0.59 − 2.08]
Erlotinib+R1507	1.12 [0.6 − 2.12]
Vinflunine	1.14 [0.69 − 1.87]
Docetaxel	1.14 [0.79 − 1.63]
Gefitinib	1.17 [0.79 − 1.72]
Pemetrexed	1.18 [0.79 − 1.75]
Entinostat+Erlotinib	1.2 [0.63 − 2.26]
Erlotinib	1.21 [0.8 − 1.82]
Gefitinib+Nimotuzumab	1.21 [0.66 − 2.2]
Pemetrexed+Cetuximab	1.21 [0.71 − 2.08]
Erlotinib+Onartuzumab	1.23 [0.75 − 2.05]
Docetaxel+PX−866	1.23 [0.66 − 2.31]
Selumetinib	1.26 [0.6 − 2.67]
Erlotinib+Figitumumab	1.31 [0.76 − 2.25]
Trametinib	1.39 [0.74 − 2.62]
AXL1717	1.48 [0.85 − 2.55]
Pemetrexed+Sunitinib	1.52 [0.8 − 2.95]
Sunitinib	1.65 [0.84 − 3.23]
Pemetrexed+Matuzumab	1.71 [0.85 − 3.45]
Placebo	1.78 [1.08 − 2.92]
Cetuximab	2.04 [0.85 − 4.86]

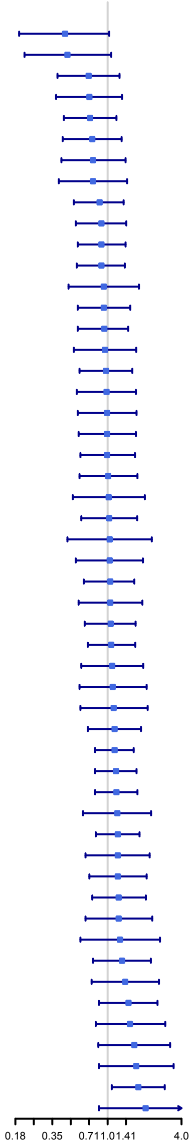

Treatments vs. Atezolizumab	HR[CrI 95%]
Erlotinib+Cabozantinib	0.42 [0.19 − 0.93]
Cabozantinib	0.44 [0.2 − 0.96]
Paclitaxel+Bevacizumab	0.66 [0.39 − 1.11]
Erlotinib+Pazopanib	0.66 [0.38 − 1.17]
Pemetrexed+Erlotinib	0.67 [0.44 − 1.03]
Erlotinib+Bevacizumab	0.7 [0.43 − 1.14]
Docetaxel+Tamoxifen	0.71 [0.41 − 1.23]
Tegafur/uracil+Gefitinib	0.71 [0.39 − 1.29]
Docetaxel+Vandetanib	0.8 [0.53 − 1.17]
Erlotinib+Tivantinib	0.82 [0.55 − 1.23]
Nivolumab	0.83 [0.56 − 1.2]
Docetaxel+Ramucirumab	0.83 [0.57 − 1.21]
Erlotinib+Everolimus	0.87 [0.47 − 1.6]
Docetaxel+Aflibercept	0.87 [0.56 − 1.34]
Docetaxel+Selumetinib	0.87 [0.57 − 1.28]
Docetaxel+Erlotinib	0.89 [0.52 − 1.51]
Docetaxel+Nintedanib	0.9 [0.59 − 1.38]
Icotinib	0.92 [0.56 − 1.49]
Afatinib	0.92 [0.56 − 1.5]
Pemetrexed+Nintedanib	0.92 [0.57 − 1.49]
Erlotinib+Sunitinib	0.92 [0.59 − 1.46]
Pembrolizumab	0.93 [0.6 − 1.46]
Pemetrexed+Vandetanib	0.94 [0.58 − 1.53]
Erlotinib+Fulvestrant	0.95 [0.5 − 1.79]
Docetaxel+Cetuximab	0.96 [0.6 − 1.52]
Erlotinib+Sorafenib	0.97 [0.54 − 1.73]
Erlotinib+Dalotuzumab	0.97 [0.46 − 2.04]
Vandetanib	0.98 [0.64 − 1.44]
Nab−Paclitaxel	0.98 [0.57 − 1.7]
Dacomitinib	0.99 [0.65 − 1.47]
Amrubicin	1.02 [0.6 − 1.72]
Pralatrexate	1.02 [0.57 − 1.84]
Erlotinib+R1507	1.05 [0.59 − 1.88]
Vinflunine	1.06 [0.69 − 1.64]
Docetaxel	1.06 [0.81 − 1.38]
Gefitinib	1.09 [0.8 − 1.47]
Pemetrexed	1.1 [0.8 − 1.51]
Entinostat+Erlotinib	1.11 [0.62 − 2]
Erlotinib	1.12 [0.81 − 1.56]
Gefitinib+Nimotuzumab	1.12 [0.65 − 1.95]
Pemetrexed+Cetuximab	1.13 [0.7 − 1.83]
Erlotinib+Onartuzumab	1.15 [0.74 − 1.79]
Docetaxel+PX−866	1.15 [0.64 − 2.04]
Selumetinib	1.18 [0.58 − 2.39]
Erlotinib+Figitumumab	1.22 [0.75 − 1.97]
Trametinib	1.3 [0.73 − 2.32]
AXL1717	1.38 [0.85 − 2.23]
Pemetrexed+Sunitinib	1.42 [0.77 − 2.63]
Sunitinib	1.53 [0.83 − 2.87]
Pemetrexed+Matuzumab	1.59 [0.84 − 3.08]
Placebo	1.66 [1.07 − 2.55]
Cetuximab	1.9 [0.82 − 4.38]

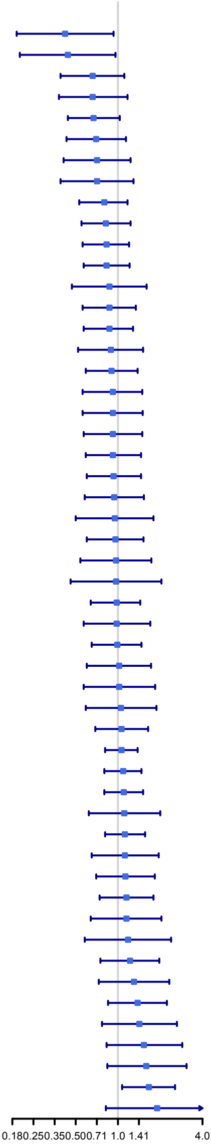

Treatments vs. Docetaxel+Nintedanib	HR[CrI 95%]
Erlotinib+Cabozantinib	0.46 [0.2 − 1.06]
Cabozantinib	0.49 [0.22 − 1.1]
Paclitaxel+Bevacizumab	0.73 [0.41 − 1.29]
Erlotinib+Pazopanib	0.74 [0.41 − 1.34]
Pemetrexed+Erlotinib	0.75 [0.47 − 1.2]
Erlotinib+Bevacizumab	0.77 [0.46 − 1.32]
Docetaxel+Tamoxifen	0.78 [0.44 − 1.42]
Tegafur/uracil+Gefitinib	0.79 [0.42 − 1.48]
Docetaxel+Vandetanib	0.89 [0.56 − 1.36]
Erlotinib+Tivantinib	0.92 [0.58 − 1.44]
Nivolumab	0.92 [0.59 − 1.4]
Docetaxel+Ramucirumab	0.92 [0.6 − 1.42]
Erlotinib+Everolimus	0.96 [0.51 − 1.84]
Docetaxel+Aflibercept	0.96 [0.6 − 1.57]
Docetaxel+Selumetinib	0.97 [0.6 − 1.49]
Docetaxel+Erlotinib	0.99 [0.56 − 1.74]
Icotinib	1.02 [0.59 − 1.73]
Afatinib	1.02 [0.6 − 1.75]
Pemetrexed+Nintedanib	1.02 [0.61 − 1.73]
Erlotinib+Sunitinib	1.03 [0.63 − 1.7]
Pembrolizumab	1.03 [0.63 − 1.7]
Pemetrexed+Vandetanib	1.05 [0.62 − 1.8]
Erlotinib+Fulvestrant	1.06 [0.54 − 2.05]
Docetaxel+Cetuximab	1.07 [0.64 − 1.78]
Erlotinib+Dalotuzumab	1.08 [0.5 − 2.34]
Erlotinib+Sorafenib	1.08 [0.58 − 1.98]
Nab−Paclitaxel	1.09 [0.61 − 1.96]
Vandetanib	1.09 [0.67 − 1.69]
Dacomitinib	1.1 [0.69 − 1.72]
Atezolizumab	1.11 [0.72 − 1.71]
Amrubicin	1.13 [0.64 − 1.99]
Pralatrexate	1.14 [0.62 − 2.11]
Erlotinib+R1507	1.17 [0.63 − 2.16]
Vinflunine	1.18 [0.73 − 1.9]
Docetaxel	1.18 [0.84 − 1.64]
Gefitinib	1.21 [0.83 − 1.75]
Pemetrexed	1.22 [0.84 − 1.79]
Entinostat+Erlotinib	1.24 [0.67 − 2.31]
Erlotinib	1.25 [0.85 − 1.85]
Gefitinib+Nimotuzumab	1.25 [0.69 − 2.25]
Pemetrexed+Cetuximab	1.26 [0.75 − 2.12]
Erlotinib+Onartuzumab	1.27 [0.78 − 2.09]
Docetaxel+PX−866	1.27 [0.69 − 2.35]
Selumetinib	1.31 [0.63 − 2.74]
Erlotinib+Figitumumab	1.35 [0.79 − 2.3]
Trametinib	1.44 [0.78 − 2.67]
AXL1717	1.53 [0.9 − 2.58]
Pemetrexed+Sunitinib	1.58 [0.83 − 3.03]
Sunitinib	1.71 [0.89 − 3.31]
Pemetrexed+Matuzumab	1.77 [0.89 − 3.54]
Placebo	1.85 [1.14 − 2.97]
Cetuximab	2.11 [0.89 − 5]

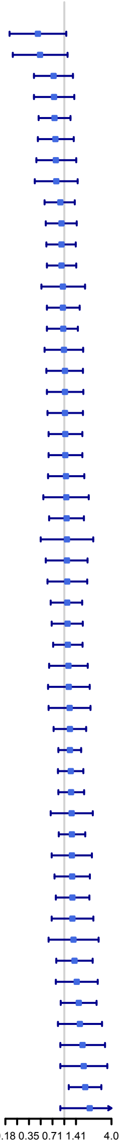

Treatments vs. Docetaxel+Ramucirumab	HR[CrI 95%]
Erlotinib+Cabozantinib	0.5 [0.23 − 1.13]
Cabozantinib	0.53 [0.24 − 1.16]
Paclitaxel+Bevacizumab	0.79 [0.46 − 1.34]
Erlotinib+Pazopanib	0.8 [0.46 − 1.4]
Pemetrexed+Erlotinib	0.81 [0.53 − 1.25]
Erlotinib+Bevacizumab	0.84 [0.51 − 1.37]
Docetaxel+Tamoxifen	0.85 [0.49 − 1.49]
Tegafur/uracil+Gefitinib	0.86 [0.47 − 1.55]
Docetaxel+Vandetanib	0.97 [0.64 − 1.41]
Erlotinib+Tivantinib	1 [0.66 − 1.49]
Nivolumab	1 [0.67 − 1.45]
Erlotinib+Everolimus	1.05 [0.57 − 1.93]
Docetaxel+Aflibercept	1.05 [0.68 − 1.62]
Docetaxel+Selumetinib	1.05 [0.69 − 1.55]
Docetaxel+Erlotinib	1.07 [0.63 − 1.83]
Docetaxel+Nintedanib	1.09 [0.7 − 1.67]
Icotinib	1.11 [0.67 − 1.8]
Afatinib	1.11 [0.68 − 1.82]
Pemetrexed+Nintedanib	1.11 [0.69 − 1.8]
Erlotinib+Sunitinib	1.12 [0.71 − 1.77]
Pembrolizumab	1.13 [0.72 − 1.76]
Pemetrexed+Vandetanib	1.14 [0.7 − 1.86]
Erlotinib+Fulvestrant	1.15 [0.61 − 2.17]
Docetaxel+Cetuximab	1.16 [0.73 − 1.84]
Erlotinib+Dalotuzumab	1.17 [0.55 − 2.48]
Erlotinib+Sorafenib	1.17 [0.65 − 2.07]
Vandetanib	1.18 [0.76 − 1.75]
Nab−Paclitaxel	1.18 [0.68 − 2.04]
Dacomitinib	1.2 [0.78 − 1.79]
Atezolizumab	1.21 [0.82 − 1.76]
Amrubicin	1.23 [0.72 − 2.07]
Pralatrexate	1.24 [0.69 − 2.21]
Erlotinib+R1507	1.27 [0.7 − 2.27]
Docetaxel	1.28 [0.97 − 1.67]
Vinflunine	1.28 [0.82 − 1.98]
Gefitinib	1.32 [0.96 − 1.78]
Pemetrexed	1.33 [0.96 − 1.83]
Entinostat+Erlotinib	1.35 [0.75 − 2.42]
Erlotinib	1.36 [0.97 − 1.89]
Gefitinib+Nimotuzumab	1.36 [0.78 − 2.36]
Pemetrexed+Cetuximab	1.37 [0.85 − 2.2]
Erlotinib+Onartuzumab	1.39 [0.89 − 2.17]
Docetaxel+PX−866	1.39 [0.77 − 2.47]
Selumetinib	1.42 [0.7 − 2.9]
Erlotinib+Figitumumab	1.47 [0.89 − 2.39]
Trametinib	1.57 [0.87 − 2.8]
AXL1717	1.66 [1.02 − 2.69]
Pemetrexed+Sunitinib	1.72 [0.93 − 3.16]
Sunitinib	1.86 [1 − 3.47]
Pemetrexed+Matuzumab	1.93 [1 − 3.73]
Placebo	2.01 [1.29 − 3.08]
Cetuximab	2.29 [0.99 − 5.29]

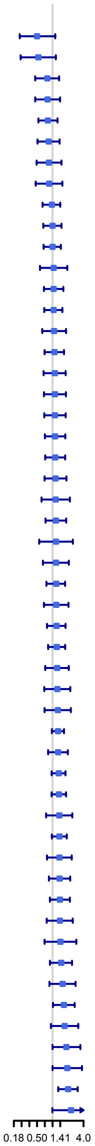

Treatments vs. Afatinib	HR[CrI 95%]
Erlotinib+Cabozantinib	0.45 [0.2 − 1.03]
Cabozantinib	0.48 [0.22 − 1.08]
Paclitaxel+Bevacizumab	0.71 [0.38 − 1.32]
Erlotinib+Pazopanib	0.72 [0.4 − 1.29]
Pemetrexed+Erlotinib	0.73 [0.44 − 1.22]
Erlotinib+Bevacizumab	0.76 [0.45 − 1.27]
Docetaxel+Tamoxifen	0.77 [0.4 − 1.46]
Tegafur/uracil+Gefitinib	0.77 [0.39 − 1.48]
Docetaxel+Vandetanib	0.87 [0.52 − 1.42]
Erlotinib+Tivantinib	0.9 [0.58 − 1.38]
Nivolumab	0.9 [0.54 − 1.46]
Docetaxel+Ramucirumab	0.9 [0.55 − 1.47]
Erlotinib+Everolimus	0.94 [0.5 − 1.78]
Docetaxel+Aflibercept	0.94 [0.55 − 1.62]
Docetaxel+Selumetinib	0.95 [0.55 − 1.56]
Docetaxel+Erlotinib	0.97 [0.55 − 1.67]
Docetaxel+Nintedanib	0.98 [0.57 − 1.67]
Icotinib	1 [0.56 − 1.74]
Pemetrexed+Nintedanib	1 [0.57 − 1.75]
Erlotinib+Sunitinib	1.01 [0.62 − 1.63]
Pembrolizumab	1.01 [0.58 − 1.76]
Pemetrexed+Vandetanib	1.03 [0.59 − 1.79]
Erlotinib+Fulvestrant	1.04 [0.54 − 1.98]
Docetaxel+Cetuximab	1.05 [0.59 − 1.83]
Erlotinib+Sorafenib	1.05 [0.58 − 1.91]
Erlotinib+Dalotuzumab	1.06 [0.49 − 2.27]
Vandetanib	1.06 [0.65 − 1.65]
Nab−Paclitaxel	1.06 [0.58 − 1.97]
Dacomitinib	1.08 [0.68 − 1.65]
Atezolizumab	1.09 [0.66 − 1.78]
Amrubicin	1.11 [0.59 − 2.03]
Pralatrexate	1.11 [0.61 − 2.04]
Erlotinib+R1507	1.14 [0.62 − 2.08]
Docetaxel	1.15 [0.76 − 1.74]
Vinflunine	1.15 [0.67 − 1.98]
Gefitinib	1.19 [0.77 − 1.79]
Pemetrexed	1.19 [0.78 − 1.82]
Entinostat+Erlotinib	1.21 [0.66 − 2.22]
Erlotinib	1.22 [0.84 − 1.76]
Gefitinib+Nimotuzumab	1.22 [0.65 − 2.27]
Pemetrexed+Cetuximab	1.23 [0.7 − 2.13]
Erlotinib+Onartuzumab	1.25 [0.78 − 2]
Docetaxel+PX−866	1.25 [0.64 − 2.4]
Selumetinib	1.28 [0.6 − 2.73]
Erlotinib+Figitumumab	1.32 [0.78 − 2.2]
Trametinib	1.41 [0.73 − 2.73]
AXL1717	1.5 [0.83 − 2.67]
Pemetrexed+Sunitinib	1.54 [0.79 − 3.03]
Sunitinib	1.67 [0.85 − 3.33]
Pemetrexed+Matuzumab	1.74 [0.85 − 3.55]
Placebo	1.81 [1.13 − 2.84]
Cetuximab	2.06 [0.86 − 4.96]

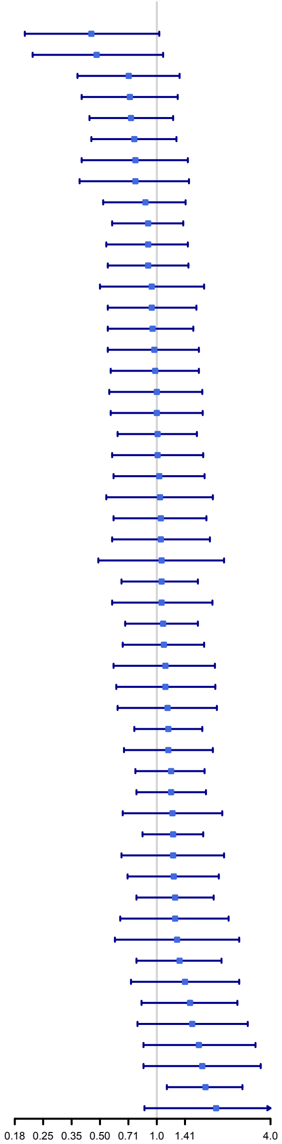

Treatments vs. Cabozantinib	HR[CrI 95%]
Erlotinib+Cabozantinib	0.94 [0.34 − 2.63]
Paclitaxel+Bevacizumab	1.49 [0.63 − 3.59]
Erlotinib+Pazopanib	1.5 [0.65 − 3.5]
Pemetrexed+Erlotinib	1.52 [0.69 − 3.39]
Erlotinib+Bevacizumab	1.58 [0.71 − 3.51]
Docetaxel+Tamoxifen	1.6 [0.66 − 3.88]
Tegafur/uracil+Gefitinib	1.6 [0.66 − 3.94]
Docetaxel+Vandetanib	1.81 [0.82 − 4]
Nivolumab	1.87 [0.85 − 4.13]
Erlotinib+Tivantinib	1.87 [0.89 − 3.95]
Docetaxel+Ramucirumab	1.87 [0.86 − 4.15]
Erlotinib+Everolimus	1.96 [0.82 − 4.76]
Docetaxel+Selumetinib	1.96 [0.89 − 4.38]
Docetaxel+Aflibercept	1.96 [0.89 − 4.46]
Docetaxel+Erlotinib	2.01 [0.87 − 4.6]
Docetaxel+Nintedanib	2.04 [0.91 − 4.61]
Icotinib	2.08 [0.9 − 4.74]
Afatinib	2.09 [0.93 − 4.64]
Pemetrexed+Nintedanib	2.09 [0.92 − 4.75]
Erlotinib+Sunitinib	2.11 [0.97 − 4.54]
Pembrolizumab	2.11 [0.93 − 4.8]
Pemetrexed+Vandetanib	2.14 [0.95 − 4.89]
Erlotinib+Fulvestrant	2.16 [0.88 − 5.25]
Docetaxel+Cetuximab	2.18 [0.95 − 5.05]
Erlotinib+Sorafenib	2.19 [0.93 − 5.17]
Erlotinib+Dalotuzumab	2.2 [0.84 − 5.78]
Vandetanib	2.2 [1.03 − 4.72]
Nab−Paclitaxel	2.21 [0.94 − 5.3]
Dacomitinib	2.24 [1.05 − 4.74]
Atezolizumab	2.27 [1.04 − 4.98]
Amrubicin	2.3 [0.97 − 5.48]
Pralatrexate	2.32 [0.98 − 5.52]
Erlotinib+R1507	2.37 [1.01 − 5.64]
Docetaxel	2.4 [1.15 − 5.06]
Vinflunine	2.4 [1.07 − 5.4]
Gefitinib	2.47 [1.18 − 5.2]
Pemetrexed	2.49 [1.19 − 5.24]
Entinostat+Erlotinib	2.52 [1.06 − 6]
Gefitinib+Nimotuzumab	2.54 [1.07 − 6.12]
Erlotinib	2.55 [1.25 − 5.21]
Pemetrexed+Cetuximab	2.57 [1.12 − 5.81]
Docetaxel+PX−866	2.59 [1.07 − 6.53]
Erlotinib+Onartuzumab	2.6 [1.21 − 5.65]
Selumetinib	2.67 [1 − 7.11]
Erlotinib+Figitumumab	2.75 [1.24 − 6.12]
Trametinib	2.94 [1.2 − 7.23]
AXL1717	3.11 [1.33 − 7.34]
Pemetrexed+Sunitinib	3.23 [1.31 − 7.98]
Sunitinib	3.47 [1.4 − 8.78]
Pemetrexed+Matuzumab	3.61 [1.42 − 9.2]
Placebo	3.77 [1.75 − 8.08]
Cetuximab	4.3 [1.45 − 12.57]

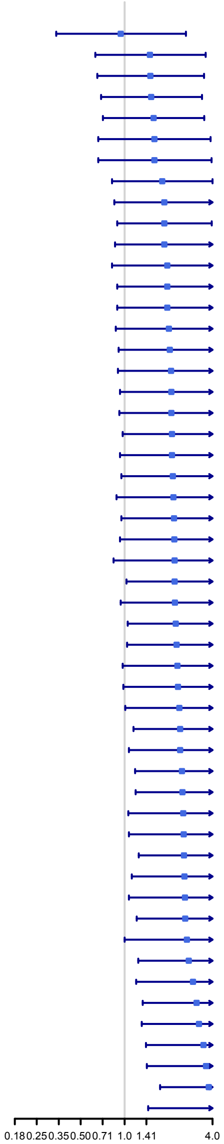

Treatments vs. Erlotinib+Cabozantinib	HR[CrI 95%]
Cabozantinib	1.06 [0.38 − 2.94]
Paclitaxel+Bevacizumab	1.58 [0.64 − 3.85]
Erlotinib+Pazopanib	1.59 [0.66 − 3.77]
Pemetrexed+Erlotinib	1.62 [0.71 − 3.64]
Erlotinib+Bevacizumab	1.67 [0.73 − 3.82]
Docetaxel+Tamoxifen	1.7 [0.69 − 4.2]
Tegafur/uracil+Gefitinib	1.71 [0.68 − 4.24]
Docetaxel+Vandetanib	1.93 [0.84 − 4.3]
Nivolumab	1.99 [0.88 − 4.45]
Erlotinib+Tivantinib	1.99 [0.92 − 4.27]
Docetaxel+Ramucirumab	1.99 [0.89 − 4.43]
Docetaxel+Aflibercept	2.09 [0.91 − 4.81]
Erlotinib+Everolimus	2.09 [0.84 − 5.17]
Docetaxel+Selumetinib	2.1 [0.91 − 4.72]
Docetaxel+Erlotinib	2.14 [0.91 − 4.97]
Docetaxel+Nintedanib	2.17 [0.94 − 4.94]
Icotinib	2.2 [0.94 − 5.16]
Afatinib	2.21 [0.97 − 5.03]
Pemetrexed+Nintedanib	2.22 [0.95 − 5.13]
Erlotinib+Sunitinib	2.22 [1 − 4.92]
Pembrolizumab	2.24 [0.97 − 5.2]
Pemetrexed+Vandetanib	2.28 [0.98 − 5.28]
Erlotinib+Fulvestrant	2.29 [0.92 − 5.74]
Docetaxel+Cetuximab	2.32 [0.99 − 5.37]
Erlotinib+Dalotuzumab	2.33 [0.86 − 6.37]
Erlotinib+Sorafenib	2.34 [0.96 − 5.57]
Vandetanib	2.35 [1.05 − 5.06]
Nab−Paclitaxel	2.35 [0.97 − 5.7]
Dacomitinib	2.38 [1.09 − 5.11]
Atezolizumab	2.41 [1.08 − 5.34]
Amrubicin	2.45 [1 − 5.92]
Pralatrexate	2.46 [1.03 − 5.95]
Erlotinib+R1507	2.52 [1.05 − 6.12]
Vinflunine	2.55 [1.1 − 5.85]
Docetaxel	2.55 [1.19 − 5.41]
Gefitinib	2.62 [1.22 − 5.59]
Pemetrexed	2.65 [1.23 − 5.65]
Entinostat+Erlotinib	2.68 [1.1 − 6.49]
Gefitinib+Nimotuzumab	2.7 [1.11 − 6.61]
Erlotinib	2.71 [1.29 − 5.64]
Pemetrexed+Cetuximab	2.72 [1.17 − 6.23]
Docetaxel+PX−866	2.76 [1.09 − 6.9]
Erlotinib+Onartuzumab	2.76 [1.25 − 6.15]
Selumetinib	2.84 [1.05 − 7.64]
Erlotinib+Figitumumab	2.92 [1.29 − 6.62]
Trametinib	3.12 [1.26 − 7.83]
AXL1717	3.31 [1.4 − 7.86]
Pemetrexed+Sunitinib	3.41 [1.35 − 8.73]
Sunitinib	3.7 [1.46 − 9.4]
Pemetrexed+Matuzumab	3.83 [1.49 − 10.04]
Placebo	4 [1.8 − 8.71]
Cetuximab	4.56 [1.54 − 13.37]

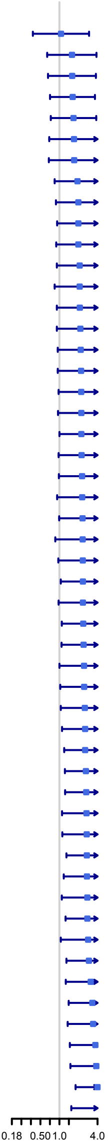

Treatments vs. Placebo	HR[CrI 95%]
Erlotinib+Cabozantinib	0.25 [0.11 − 0.56]
Cabozantinib	0.27 [0.12 − 0.57]
Paclitaxel+Bevacizumab	0.39 [0.22 − 0.7]
Erlotinib+Pazopanib	0.4 [0.23 − 0.69]
Pemetrexed+Erlotinib	0.4 [0.26 − 0.64]
Erlotinib+Bevacizumab	0.42 [0.27 − 0.67]
Docetaxel+Tamoxifen	0.42 [0.24 − 0.77]
Tegafur/uracil+Gefitinib	0.43 [0.23 − 0.79]
Docetaxel+Vandetanib	0.48 [0.31 − 0.75]
Erlotinib+Tivantinib	0.5 [0.35 − 0.72]
Nivolumab	0.5 [0.32 − 0.77]
Docetaxel+Ramucirumab	0.5 [0.32 − 0.78]
Erlotinib+Everolimus	0.52 [0.29 − 0.95]
Docetaxel+Aflibercept	0.52 [0.32 − 0.85]
Docetaxel+Selumetinib	0.52 [0.33 − 0.82]
Docetaxel+Erlotinib	0.53 [0.32 − 0.88]
Docetaxel+Nintedanib	0.54 [0.34 − 0.88]
Icotinib	0.55 [0.33 − 0.93]
Afatinib	0.55 [0.35 − 0.89]
Pemetrexed+Nintedanib	0.55 [0.34 − 0.92]
Erlotinib+Sunitinib	0.56 [0.37 − 0.86]
Pembrolizumab	0.56 [0.34 − 0.93]
Pemetrexed+Vandetanib	0.57 [0.34 − 0.95]
Erlotinib+Fulvestrant	0.57 [0.31 − 1.05]
Docetaxel+Cetuximab	0.58 [0.35 − 0.97]
Erlotinib+Sorafenib	0.58 [0.34 − 1.01]
Erlotinib+Dalotuzumab	0.59 [0.28 − 1.21]
Vandetanib	0.59 [0.39 − 0.86]
Nab−Paclitaxel	0.59 [0.33 − 1.04]
Dacomitinib	0.6 [0.44 − 0.79]
Atezolizumab	0.6 [0.39 − 0.93]
Amrubicin	0.61 [0.35 − 1.08]
Pralatrexate	0.62 [0.35 − 1.08]
Erlotinib+R1507	0.63 [0.36 − 1.11]
Docetaxel	0.64 [0.45 − 0.9]
Vinflunine	0.64 [0.39 − 1.04]
Gefitinib	0.66 [0.46 − 0.93]
Pemetrexed	0.66 [0.47 − 0.95]
Entinostat+Erlotinib	0.67 [0.38 − 1.18]
Erlotinib	0.68 [0.51 − 0.9]
Gefitinib+Nimotuzumab	0.68 [0.38 − 1.21]
Pemetrexed+Cetuximab	0.68 [0.42 − 1.13]
Docetaxel+PX−866	0.69 [0.37 − 1.28]
Erlotinib+Onartuzumab	0.69 [0.46 − 1.05]
Selumetinib	0.71 [0.35 − 1.46]
Erlotinib+Figitumumab	0.73 [0.46 − 1.16]
Trametinib	0.78 [0.42 − 1.45]
AXL1717	0.83 [0.49 − 1.42]
Pemetrexed+Sunitinib	0.86 [0.46 − 1.61]
Sunitinib	0.92 [0.49 − 1.77]
Pemetrexed+Matuzumab	0.96 [0.49 − 1.89]
Cetuximab	1.14 [0.49 − 2.69]

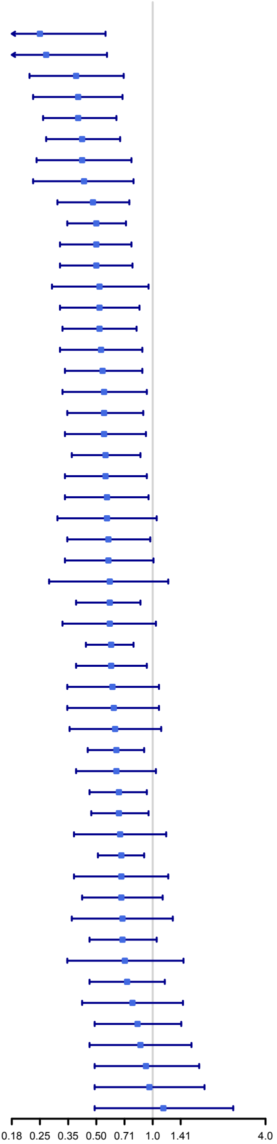

(c) Objective response

Treatments vs. Docetaxel	OR[CrI 95%]
Cabozantinib	8 [0.73 − 289.17]
Docetaxel+PX−866	7.43 [0.33 − 159.02]
Paclitaxel+Bevacizumab	5.7 [1.35 − 30.81]
Docetaxel+Tamoxifen	3.38 [0.99 − 11.8]
Erlotinib+Cabozantinib	3.15 [0.2 − 113.18]
Docetaxel+Aflibercept	3.07 [1.18 − 8.1]
Pemetrexed+Vandetanib	3.05 [1.04 − 9.51]
Pemetrexed+Erlotinib	3.03 [1.4 − 6.88]
Erlotinib+Pazopanib	2.86 [0.59 − 17.62]
Erlotinib+Bevacizumab	2.69 [0.85 − 8.74]
Erlotinib+Fulvestrant	2.48 [0.61 − 10.99]
Dacomitinib	2.26 [0.98 − 5.49]
Pembrolizumab	2.15 [0.8 − 5.67]
Docetaxel+Selumetinib	2.14 [0.91 − 6.16]
Afatinib	2.04 [0.58 − 7.7]
Nivolumab	2.02 [0.98 − 4.27]
Docetaxel+Vandetanib	2.01 [0.95 − 4.5]
Erlotinib+Sunitinib	2.01 [0.75 − 5.6]
Pemetrexed+Sunitinib	1.88 [0.43 − 8.95]
Erlotinib+Tivantinib	1.88 [0.81 − 4.38]
Erlotinib+Figitumumab	1.84 [0.52 − 6.5]
Docetaxel+Ramucirumab	1.83 [0.89 − 3.71]
Vandetanib	1.79 [0.78 − 5.24]
Pemetrexed+Cetuximab	1.77 [0.54 − 6.09]
Tegafur/uracil+Gefitinib	1.62 [0.47 − 5.46]
Nab−Paclitaxel	1.61 [0.36 − 7.4]
Icotinib	1.52 [0.53 − 4.34]
Pemetrexed+Sorafenib	1.48 [0.29 − 7.61]
Gefitinib	1.45 [0.97 − 2.15]
Erlotinib+Everolimus	1.43 [0.33 − 6.62]
Sunitinib	1.37 [0.31 − 6.48]
Docetaxel+Nintedanib	1.35 [0.48 − 3.78]
Docetaxel+Erlotinib	1.26 [0.23 − 7.4]
Erlotinib	1.24 [0.76 − 2.08]
Selumetinib	1.22 [0.1 − 14.61]
Docetaxe+Cetuximab	1.21 [0.36 − 4.06]
Pemetrexed+Nintedanib	1.21 [0.41 − 3.68]
Pemetrexed	1.1 [0.72 − 1.73]
Trametinib	1.04 [0.26 − 4.75]
Gefitinib+Nimotuzumab	1.03 [0.29 − 3.56]
Erlotinib+Onartuzumab	1.02 [0.36 − 3.04]
Topotecan	1.02 [0.4 − 2.62]
Atezolizumab	1.01 [0.49 − 2.07]
Erlotinib+R1507	0.97 [0.16 − 5.38]
Erlotinib+Sorafenib	0.91 [0.21 − 4.2]
Paclitaxel	0.88 [0.4 − 2.37]
Vinflunine	0.79 [0.24 − 2.54]
Amrubicin	0.68 [0.21 − 2.14]
Pemetrexed+Matuzumab	0.45 [0.01 − 7.48]
Vinorelbine	0.4 [0.01 − 10.68]
Entinostat+Erlotinib	0.33 [0.03 − 2.11]
Erlotinib+Dalotuzumab	0.31 [0.01 − 4.08]
Pralatrexate	0.3 [0.03 − 1.88]
Cetuximab	0.25 [0.01 − 6.83]
Placebo	0.2 [0.08 − 0.46]
AXL1717	0.06 [0 − 1.17]

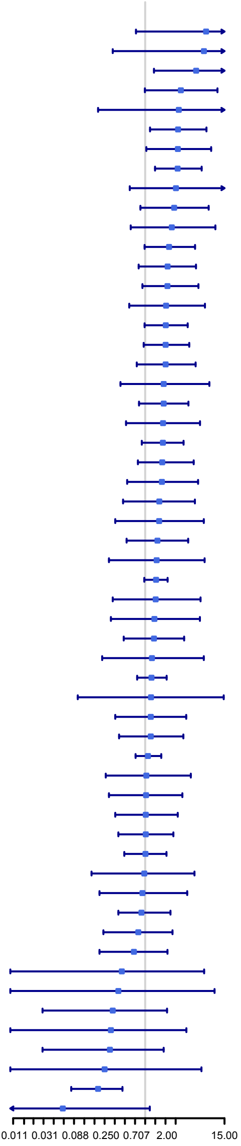

Treatments vs. Pemetrexed	OR[CrI 95%]
Cabozantinib	7.19 [0.66 − 263.75]
Docetaxel+PX−866	6.71 [0.29 − 150.96]
Paclitaxel+Bevacizumab	5.21 [1.15 − 28.36]
Docetaxel+Tamoxifen	3.07 [0.83 − 11.47]
Erlotinib+Cabozantinib	2.85 [0.18 − 103.34]
Docetaxel+Aflibercept	2.81 [0.95 − 7.89]
Pemetrexed+Vandetanib	2.77 [1.01 − 7.78]
Pemetrexed+Erlotinib	2.76 [1.39 − 5.59]
Erlotinib+Pazopanib	2.59 [0.53 − 15.8]
Erlotinib+Bevacizumab	2.44 [0.76 − 7.74]
Erlotinib+Fulvestrant	2.26 [0.55 − 9.78]
Dacomitinib	2.05 [0.88 − 4.86]
Pembrolizumab	1.96 [0.65 − 5.54]
Docetaxel+Selumetinib	1.95 [0.74 − 6]
Afatinib	1.86 [0.53 − 6.9]
Nivolumab	1.84 [0.78 − 4.29]
Docetaxel+Vandetanib	1.84 [0.76 − 4.45]
Erlotinib+Sunitinib	1.83 [0.67 − 5.01]
Pemetrexed+Sunitinib	1.72 [0.41 − 7.52]
Erlotinib+Tivantinib	1.7 [0.73 − 3.87]
Docetaxel+Ramucirumab	1.67 [0.7 − 3.75]
Erlotinib+Figitumumab	1.66 [0.47 − 5.86]
Vandetanib	1.62 [0.7 − 4.66]
Pemetrexed+Cetuximab	1.61 [0.52 − 5.01]
Tegafur/uracil+Gefitinib	1.48 [0.41 − 4.95]
Nab−Paclitaxel	1.47 [0.34 − 6.22]
Icotinib	1.39 [0.46 − 3.93]
Pemetrexed+Sorafenib	1.35 [0.28 − 6.51]
Gefitinib	1.32 [0.82 − 2.03]
Erlotinib+Everolimus	1.29 [0.3 − 6]
Sunitinib	1.24 [0.29 − 5.48]
Docetaxel+Nintedanib	1.23 [0.39 − 3.65]
Docetaxel+Erlotinib	1.14 [0.21 − 6.64]
Erlotinib	1.13 [0.69 − 1.83]
Selumetinib	1.11 [0.09 − 12.79]
Pemetrexed+Nintedanib	1.1 [0.4 − 3.05]
Docetaxel+Cetuximab	1.09 [0.3 − 3.94]
Trametinib	0.94 [0.22 − 4.48]
Gefitinib+Nimotuzumab	0.93 [0.26 − 3.25]
Erlotinib+Onartuzumab	0.93 [0.33 − 2.69]
Topotecan	0.93 [0.33 − 2.56]
Atezolizumab	0.91 [0.39 − 2.07]
Docetaxel	0.91 [0.58 − 1.38]
Erlotinib+R1507	0.88 [0.15 − 4.87]
Erlotinib+Sorafenib	0.83 [0.19 − 3.73]
Paclitaxel	0.8 [0.32 − 2.31]
Vinflunine	0.71 [0.2 − 2.47]
Amrubicin	0.62 [0.18 − 2.08]
Pemetrexed+Matuzumab	0.41 [0.01 − 6.48]
Vinorelbine	0.36 [0.01 − 9.92]
Entinostat+Erlotinib	0.3 [0.03 − 1.89]
Erlotinib+Dalotuzumab	0.28 [0.01 − 3.65]
Pralatrexate	0.28 [0.03 − 1.66]
Cetuximab	0.23 [0.01 − 5.94]
Placebo	0.18 [0.07 − 0.42]
AXL1717	0.05 [0 − 1.07]

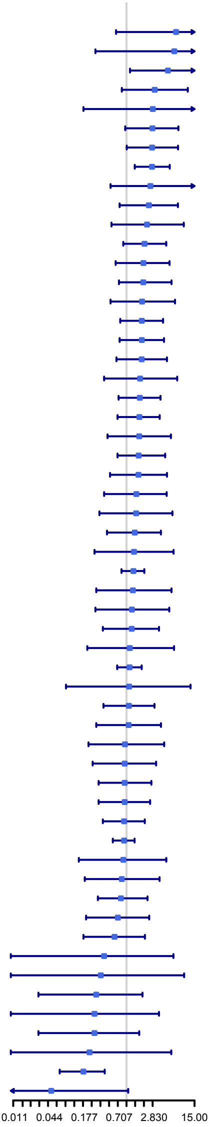

Treatments vs. Erlotinib	OR[CrI 95%]
Cabozantinib	6.34 [0.61 − 227.69]
Docetaxel+PX−866	5.93 [0.26 − 132.95]
Paclitaxel+Bevacizumab	4.6 [1 − 25.97]
Docetaxel+Tamoxifen	2.71 [0.71 − 10.35]
Erlotinib+Cabozantinib	2.53 [0.16 − 89.75]
Docetaxel+Aflibercept	2.48 [0.82 − 7.24]
Pemetrexed+Vandetanib	2.46 [0.8 − 7.71]
Pemetrexed+Erlotinib	2.44 [1.15 − 5.33]
Erlotinib+Pazopanib	2.29 [0.51 − 13.11]
Erlotinib+Bevacizumab	2.17 [0.76 − 6.21]
Erlotinib+Fulvestrant	2 [0.53 − 8.01]
Dacomitinib	1.81 [0.89 − 3.82]
Pembrolizumab	1.74 [0.57 − 5.08]
Docetaxel+Selumetinib	1.73 [0.65 − 5.53]
Afatinib	1.65 [0.51 − 5.55]
Nivolumab	1.63 [0.67 − 3.96]
Docetaxel+Vandetanib	1.62 [0.65 − 4.13]
Erlotinib+Sunitinib	1.62 [0.68 − 3.89]
Pemetrexed+Sunitinib	1.52 [0.34 − 7.24]
Erlotinib+Tivantinib	1.51 [0.76 − 2.94]
Docetaxel+Ramucirumab	1.48 [0.6 − 3.47]
Erlotinib+Figitumumab	1.48 [0.46 − 4.72]
Vandetanib	1.44 [0.68 − 3.77]
Pemetrexed+Cetuximab	1.42 [0.42 − 5]
Tegafur/uracil+Gefitinib	1.31 [0.36 − 4.48]
Nab−Paclitaxel	1.29 [0.28 − 5.98]
Icotinib	1.23 [0.4 − 3.6]
Pemetrexed+Sorafenib	1.19 [0.23 − 6.19]
Gefitinib	1.17 [0.7 − 1.88]
Erlotinib+Everolimus	1.15 [0.29 − 4.82]
Sunitinib	1.1 [0.24 − 5.22]
Docetaxel+Nintedanib	1.08 [0.34 − 3.35]
Docetaxel+Erlotinib	1.02 [0.19 − 5.39]
Selumetinib	0.99 [0.08 − 11.97]
Pemetrexed+Nintedanib	0.97 [0.32 − 3.01]
Docetaxel+Cetuximab	0.97 [0.26 − 3.6]
Pemetrexed	0.89 [0.55 − 1.45]
Trametinib	0.83 [0.19 − 4.03]
Gefitinib+Nimotuzumab	0.83 [0.22 − 2.93]
Erlotinib+Onartuzumab	0.83 [0.33 − 2.12]
Topotecan	0.82 [0.28 − 2.35]
Atezolizumab	0.81 [0.34 − 1.92]
Docetaxel	0.8 [0.48 − 1.32]
Erlotinib+R1507	0.78 [0.14 − 4.03]
Erlotinib+Sorafenib	0.73 [0.18 − 3.05]
Paclitaxel	0.71 [0.28 − 2.16]
Vinflunine	0.64 [0.17 − 2.26]
Amrubicin	0.55 [0.15 − 1.91]
Pemetrexed+Matuzumab	0.36 [0.01 − 6]
Vinorelbine	0.32 [0.01 − 8.88]
Entinostat+Erlotinib	0.26 [0.03 − 1.57]
Erlotinib+Dalotuzumab	0.25 [0.01 − 3.08]
Pralatrexate	0.25 [0.03 − 1.41]
Cetuximab	0.2 [0.01 − 5.56]
Placebo	0.16 [0.06 − 0.35]
AXL1717	0.04 [0 − 0.94]

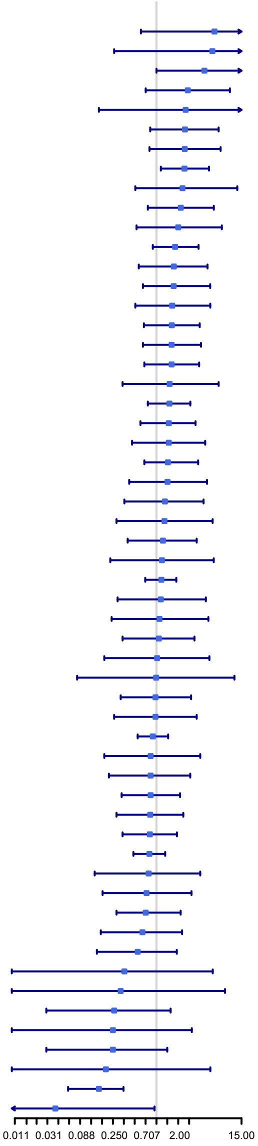

Treatments vs. Gefitinib	OR[CrI 95%]
Cabozantinib	5.5 [0.51 − 198.54]
Docetaxel+PX−866	5.11 [0.23 − 115.01]
Paclitaxel+Bevacizumab	3.95 [0.88 − 22]
Docetaxel+Tamoxifen	2.31 [0.64 − 8.71]
Erlotinib+Cabozantinib	2.19 [0.14 − 79.6]
Docetaxel+Aflibercept	2.11 [0.75 − 6.06]
Pemetrexed+Vandetanib	2.11 [0.71 − 6.58]
Pemetrexed+Erlotinib	2.09 [0.97 − 4.77]
Erlotinib+Pazopanib	1.97 [0.41 − 12.1]
Erlotinib+Bevacizumab	1.85 [0.59 − 6.03]
Erlotinib+Fulvestrant	1.72 [0.42 − 7.56]
Dacomitinib	1.55 [0.69 − 3.79]
Pembrolizumab	1.48 [0.51 − 4.28]
Docetaxel+Selumetinib	1.48 [0.58 − 4.68]
Afatinib	1.41 [0.4 − 5.34]
Nivolumab	1.39 [0.61 − 3.29]
Docetaxel+Vandetanib	1.38 [0.6 − 3.42]
Erlotinib+Sunitinib	1.38 [0.52 − 3.89]
Pemetrexed+Sunitinib	1.31 [0.29 − 6.19]
Erlotinib+Tivantinib	1.29 [0.57 − 3.03]
Erlotinib+Figitumumab	1.27 [0.36 − 4.53]
Docetaxel+Ramucirumab	1.26 [0.56 − 2.85]
Vandetanib	1.23 [0.55 − 3.51]
Pemetrexed+Cetuximab	1.22 [0.37 − 4.26]
Tegafur/uracil+Gefitinib	1.12 [0.35 − 3.53]
Nab−Paclitaxel	1.1 [0.25 − 5.13]
Icotinib	1.05 [0.39 − 2.76]
Pemetrexed+Sorafenib	1.02 [0.2 − 5.31]
Erlotinib+Everolimus	0.98 [0.23 − 4.54]
Sunitinib	0.94 [0.21 − 4.46]
Docetaxel+Nintedanib	0.92 [0.31 − 2.81]
Docetaxel+Erlotinib	0.87 [0.16 − 5]
Erlotinib	0.85 [0.53 − 1.43]
Selumetinib	0.85 [0.07 − 10.23]
Pemetrexed+Nintedanib	0.83 [0.28 − 2.56]
Docetaxel+Cetuximab	0.83 [0.24 − 3.02]
Pemetrexed	0.76 [0.49 − 1.22]
Trametinib	0.72 [0.17 − 3.45]
Gefitinib+Nimotuzumab	0.71 [0.22 − 2.32]
Erlotinib+Onartuzumab	0.71 [0.25 − 2.1]
Topotecan	0.7 [0.25 − 1.96]
Atezolizumab	0.69 [0.31 − 1.59]
Docetaxel	0.69 [0.46 − 1.03]
Erlotinib+R1507	0.67 [0.11 − 3.73]
Erlotinib+Sorafenib	0.63 [0.15 − 2.9]
Paclitaxel	0.61 [0.25 − 1.78]
Vinflunine	0.54 [0.16 − 1.88]
Amrubicin	0.47 [0.14 − 1.6]
Pemetrexed+Matuzumab	0.31 [0.01 − 5.19]
Vinorelbine	0.27 [0.01 − 7.49]
Entinostat+Erlotinib	0.23 [0.02 − 1.48]
Erlotinib+Dalotuzumab	0.21 [0.01 − 2.8]
Pralatrexate	0.21 [0.02 − 1.29]
Cetuximab	0.17 [0.01 − 4.69]
Placebo	0.14 [0.06 − 0.31]
AXL1717	0.04 [0 − 0.83]

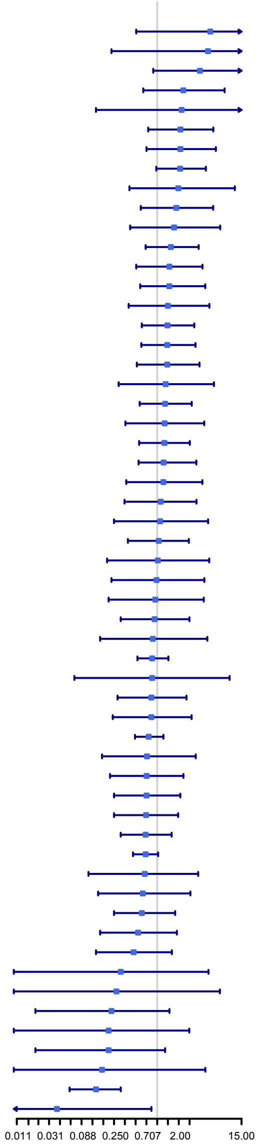

Treatments vs. Nivolumab	OR[CrI 95%]
Cabozantinib	3.95 [0.32 − 152.02]
Docetaxel+PX−866	3.67 [0.15 − 86.83]
Paclitaxel+Bevacizumab	2.82 [0.56 − 17.48]
Docetaxel+Tamoxifen	1.67 [0.39 − 7.06]
Erlotinib+Cabozantinib	1.56 [0.09 − 59.5]
Docetaxel+Aflibercept	1.52 [0.44 − 5.07]
Pemetrexed+Vandetanib	1.51 [0.41 − 5.77]
Pemetrexed+Erlotinib	1.5 [0.51 − 4.46]
Erlotinib+Pazopanib	1.42 [0.24 − 10.06]
Erlotinib+Bevacizumab	1.33 [0.33 − 5.32]
Erlotinib+Fulvestrant	1.23 [0.25 − 6.34]
Dacomitinib	1.12 [0.37 − 3.54]
Pembrolizumab	1.06 [0.31 − 3.53]
Docetaxel+Selumetinib	1.05 [0.35 − 3.88]
Afatinib	1.01 [0.24 − 4.59]
Docetaxel+Vandetanib	1 [0.34 − 2.93]
Erlotinib+Sunitinib	0.99 [0.28 − 3.48]
Pemetrexed+Sunitinib	0.93 [0.18 − 5.17]
Erlotinib+Tivantinib	0.93 [0.3 − 2.8]
Erlotinib+Figitumumab	0.91 [0.21 − 3.91]
Docetaxel+Ramucirumab	0.9 [0.32 − 2.48]
Vandetanib	0.88 [0.3 − 3.34]
Pemetrexed+Cetuximab	0.88 [0.22 − 3.66]
Tegafur/uracil+Gefitinib	0.8 [0.18 − 3.26]
Nab−Paclitaxel	0.8 [0.15 − 4.28]
Icotinib	0.75 [0.2 − 2.7]
Pemetrexed+Sorafenib	0.73 [0.12 − 4.42]
Gefitinib	0.72 [0.3 − 1.63]
Erlotinib+Everolimus	0.7 [0.14 − 3.9]
Sunitinib	0.67 [0.13 − 3.77]
Docetaxel+Nintedanib	0.67 [0.19 − 2.33]
Docetaxel+Erlotinib	0.62 [0.1 − 4.18]
Erlotinib	0.61 [0.25 − 1.5]
Selumetinib	0.6 [0.04 − 7.96]
Pemetrexed+Nintedanib	0.59 [0.16 − 2.26]
Docetaxel+Cetuximab	0.59 [0.14 − 2.43]
Pemetrexed	0.54 [0.23 − 1.28]
Trametinib	0.52 [0.1 − 2.7]
Gefitinib+Nimotuzumab	0.51 [0.12 − 2.14]
Erlotinib+Onartuzumab	0.51 [0.14 − 1.88]
Topotecan	0.5 [0.15 − 1.66]
Atezolizumab	0.5 [0.18 − 1.36]
Docetaxel	0.5 [0.23 − 1.02]
Erlotinib+R1507	0.48 [0.07 − 3.1]
Erlotinib+Sorafenib	0.45 [0.09 − 2.41]
Paclitaxel	0.43 [0.15 − 1.51]
Vinflunine	0.39 [0.1 − 1.55]
Amrubicin	0.34 [0.09 − 1.31]
Pemetrexed+Matuzumab	0.22 [0.01 − 4.03]
Vinorelbine	0.19 [0.01 − 5.67]
Entinostat+Erlotinib	0.16 [0.02 − 1.18]
Erlotinib+Dalotuzumab	0.15 [0 − 2.23]
Pralatrexate	0.15 [0.01 − 1.07]
Cetuximab	0.12 [0 − 3.62]
Placebo	0.1 [0.03 − 0.29]
AXL1717	0.03 [0 − 0.61]

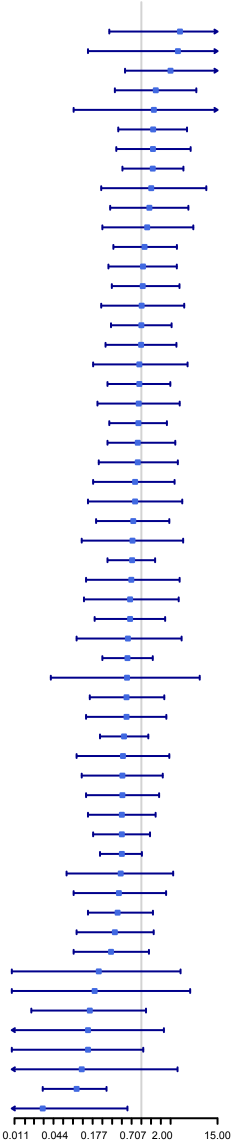

Treatments vs. Pembrolizumab	OR[CrI 95%]
Cabozantinib	3.78 [0.28 − 146.2]
Docetaxel+PX−866	3.46 [0.14 − 86.66]
Paclitaxel+Bevacizumab	2.67 [0.47 − 18.45]
Docetaxel+Tamoxifen	1.56 [0.33 − 7.8]
Erlotinib+Cabozantinib	1.49 [0.08 − 58.67]
Docetaxel+Aflibercept	1.42 [0.37 − 5.73]
Pemetrexed+Vandetanib	1.42 [0.33 − 6.41]
Pemetrexed+Erlotinib	1.41 [0.42 − 5.16]
Erlotinib+Pazopanib	1.35 [0.21 − 10.53]
Erlotinib+Bevacizumab	1.24 [0.27 − 5.88]
Erlotinib+Fulvestrant	1.16 [0.21 − 6.94]
Dacomitinib	1.05 [0.29 − 3.99]
Docetaxel+Selumetinib	0.99 [0.28 − 4.37]
Afatinib	0.95 [0.2 − 4.98]
Nivolumab	0.94 [0.28 − 3.22]
Docetaxel+Vandetanib	0.93 [0.28 − 3.33]
Erlotinib+Sunitinib	0.93 [0.23 − 3.93]
Pemetrexed+Sunitinib	0.88 [0.15 − 5.49]
Erlotinib+Tivantinib	0.87 [0.24 − 3.21]
Erlotinib+Figitumumab	0.85 [0.18 − 4.3]
Docetaxel+Ramucirumab	0.85 [0.25 − 2.85]
Vandetanib	0.83 [0.24 − 3.72]
Pemetrexed+Cetuximab	0.82 [0.18 − 3.99]
Tegafur/uracil+Gefitinib	0.75 [0.15 − 3.54]
Nab−Paclitaxel	0.75 [0.13 − 4.64]
Icotinib	0.71 [0.17 − 2.97]
Pemetrexed+Sorafenib	0.69 [0.11 − 4.7]
Gefitinib	0.67 [0.23 − 1.96]
Erlotinib+Everolimus	0.67 [0.12 − 4.12]
Sunitinib	0.63 [0.11 − 4.03]
Docetaxel+Nintedanib	0.62 [0.15 − 2.6]
Docetaxel+Erlotinib	0.59 [0.08 − 4.43]
Erlotinib	0.57 [0.2 − 1.77]
Selumetinib	0.57 [0.04 − 8.25]
Docetaxel+Cetuximab	0.56 [0.12 − 2.68]
Pemetrexed+Nintedanib	0.56 [0.13 − 2.54]
Pemetrexed	0.51 [0.18 − 1.54]
Trametinib	0.48 [0.09 − 2.95]
Topotecan	0.48 [0.12 − 1.86]
Gefitinib+Nimotuzumab	0.48 [0.1 − 2.36]
Erlotinib+Onartuzumab	0.47 [0.12 − 2.09]
Atezolizumab	0.47 [0.14 − 1.57]
Docetaxel	0.47 [0.18 − 1.25]
Erlotinib+R1507	0.45 [0.06 − 3.26]
Erlotinib+Sorafenib	0.42 [0.07 − 2.58]
Paclitaxel	0.41 [0.12 − 1.71]
Vinflunine	0.37 [0.08 − 1.69]
Amrubicin	0.32 [0.07 − 1.45]
Pemetrexed+Matuzumab	0.21 [0.01 − 4.11]
Vinorelbine	0.18 [0.01 − 5.74]
Entinostat+Erlotinib	0.15 [0.01 − 1.26]
Erlotinib+Dalotuzumab	0.14 [0 − 2.27]
Pralatrexate	0.14 [0.01 − 1.13]
Cetuximab	0.11 [0 − 3.67]
Placebo	0.09 [0.02 − 0.34]
AXL1717	0.03 [0 − 0.61]

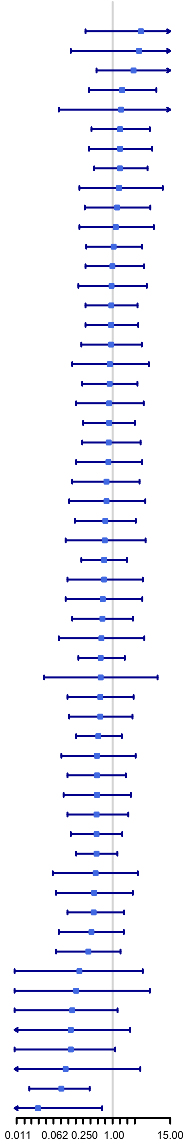

Treatments vs. Atezolizumab	OR[CrI 95%]
Cabozantinib	7.99 [0.65 − 300.37]
Docetaxel+PX−866	7.35 [0.31 − 174.69]
Paclitaxel+Bevacizumab	5.7 [1.12 − 35.34]
Docetaxel+Tamoxifen	3.36 [0.81 − 14.11]
Erlotinib+Cabozantinib	3.14 [0.18 − 117.68]
Docetaxel+Aflibercept	3.06 [0.91 − 10.1]
Pemetrexed+Vandetanib	3.04 [0.83 − 11.63]
Pemetrexed+Erlotinib	3.03 [1.07 − 8.97]
Erlotinib+Pazopanib	2.86 [0.5 − 20.05]
Erlotinib+Bevacizumab	2.67 [0.68 − 10.38]
Erlotinib+Fulvestrant	2.49 [0.51 − 12.73]
Dacomitinib	2.25 [0.75 − 7.01]
Pembrolizumab	2.14 [0.64 − 7.13]
Docetaxel+Selumetinib	2.13 [0.72 − 7.77]
Afatinib	2.04 [0.48 − 9.27]
Nivolumab	2.02 [0.73 − 5.66]
Docetaxel+Vandetanib	2.01 [0.71 − 5.89]
Erlotinib+Sunitinib	2.01 [0.58 − 6.91]
Pemetrexed+Sunitinib	1.89 [0.37 − 10.27]
Erlotinib+Tivantinib	1.87 [0.62 − 5.65]
Erlotinib+Figitumumab	1.83 [0.43 − 7.75]
Docetaxel+Ramucirumab	1.82 [0.65 − 4.95]
Vandetanib	1.77 [0.61 − 6.61]
Pemetrexed+Cetuximab	1.75 [0.44 − 7.32]
Tegafur/uracil+Gefitinib	1.61 [0.38 − 6.49]
Nab−Paclitaxel	1.6 [0.31 − 8.67]
Icotinib	1.52 [0.42 − 5.38]
Pemetrexed+Sorafenib	1.49 [0.25 − 8.88]
Gefitinib	1.45 [0.63 − 3.26]
Erlotinib+Everolimus	1.42 [0.28 − 7.75]
Sunitinib	1.35 [0.26 − 7.52]
Docetaxel+Nintedanib	1.34 [0.38 − 4.68]
Docetaxel+Erlotinib	1.26 [0.2 − 8.26]
Erlotinib	1.23 [0.52 − 2.98]
Selumetinib	1.21 [0.09 − 16.15]
Docetaxel+Cetuximab	1.2 [0.3 − 4.9]
Pemetrexed+Nintedanib	1.2 [0.33 − 4.5]
Pemetrexed	1.09 [0.48 − 2.57]
Trametinib	1.04 [0.22 − 5.52]
Topotecan	1.02 [0.31 − 3.25]
Erlotinib+Onartuzumab	1.02 [0.29 − 3.74]
Gefitinib+Nimotuzumab	1.02 [0.24 − 4.23]
Docetaxel	0.99 [0.48 − 2.03]
Erlotinib+R1507	0.97 [0.14 − 6.16]
Erlotinib+Sorafenib	0.91 [0.18 − 4.89]
Paclitaxel	0.87 [0.31 − 3.01]
Vinflunine	0.78 [0.2 − 3.06]
Amrubicin	0.67 [0.18 − 2.63]
Pemetrexed+Matuzumab	0.44 [0.01 − 8.07]
Vinorelbine	0.39 [0.01 − 11.31]
Entinostat+Erlotinib	0.32 [0.03 − 2.42]
Erlotinib+Dalotuzumab	0.31 [0.01 − 4.45]
Pralatrexate	0.3 [0.03 − 2.17]
Cetuximab	0.25 [0.01 − 7.24]
Placebo	0.2 [0.06 − 0.59]
AXL1717	0.06 [0 − 1.25]

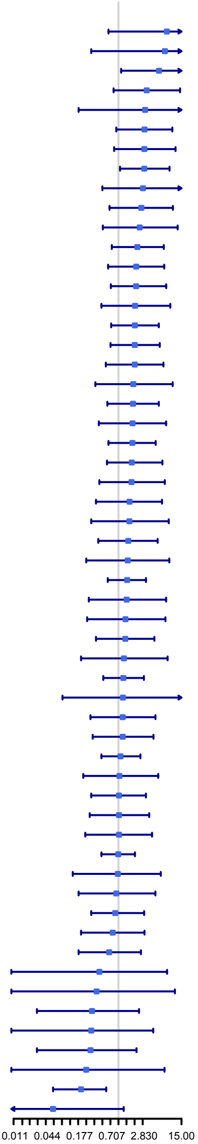

Treatments vs. Docetaxel+Nintedanib	OR[CrI 95%]
Cabozantinib	5.99 [0.41 − 244.45]
Docetaxel+PX−866	5.51 [0.21 − 142.02]
Paclitaxel+Bevacizumab	4.3 [0.73 − 30.2]
Docetaxel+Tamoxifen	2.51 [0.5 − 12.48]
Erlotinib+Cabozantinib	2.38 [0.12 − 97.13]
Docetaxel+Aflibercept	2.28 [0.56 − 9.32]
Pemetrexed+Vandetanib	2.26 [0.52 − 10.36]
Pemetrexed+Erlotinib	2.24 [0.63 − 8.42]
Erlotinib+Pazopanib	2.14 [0.33 − 17]
Erlotinib+Bevacizumab	1.99 [0.43 − 9.43]
Erlotinib+Fulvestrant	1.85 [0.32 − 11.18]
Dacomitinib	1.68 [0.45 − 6.65]
Pembrolizumab	1.6 [0.38 − 6.51]
Docetaxel+Selumetinib	1.58 [0.44 − 7.21]
Afatinib	1.52 [0.3 − 8.33]
Nivolumab	1.5 [0.43 − 5.4]
Docetaxel+Vandetanib	1.49 [0.42 − 5.57]
Erlotinib+Sunitinib	1.49 [0.36 − 6.46]
Pemetrexed+Sunitinib	1.41 [0.23 − 8.95]
Erlotinib+Tivantinib	1.4 [0.37 − 5.3]
Erlotinib+Figitumumab	1.37 [0.27 − 7.02]
Docetaxel+Ramucirumab	1.36 [0.39 − 4.69]
Vandetanib	1.33 [0.37 − 6.17]
Pemetrexed+Cetuximab	1.32 [0.27 − 6.55]
Tegafur/uracil+Gefitinib	1.2 [0.24 − 5.86]
Nab−Paclitaxel	1.19 [0.19 − 7.56]
Icotinib	1.13 [0.26 − 4.85]
Pemetrexed+Sorafenib	1.1 [0.16 − 7.58]
Gefitinib	1.08 [0.36 − 3.22]
Erlotinib+Everolimus	1.07 [0.18 − 6.75]
Sunitinib	1.02 [0.16 − 6.55]
Docetaxel+Erlotinib	0.93 [0.13 − 7.09]
Erlotinib	0.92 [0.3 − 2.96]
Selumetinib	0.91 [0.06 − 13.57]
Pemetrexed+Nintedanib	0.9 [0.2 − 4.18]
Docetaxel+Cetuximab	0.9 [0.18 − 4.37]
Pemetrexed	0.81 [0.27 − 2.53]
Trametinib	0.78 [0.14 − 4.75]
Erlotinib+Onartuzumab	0.76 [0.18 − 3.44]
Gefitinib+Nimotuzumab	0.76 [0.15 − 3.81]
Topotecan	0.76 [0.19 − 3.04]
Atezolizumab	0.75 [0.21 − 2.61]
Docetaxel	0.74 [0.26 − 2.07]
Erlotinib+R1507	0.72 [0.09 − 5.24]
Erlotinib+Sorafenib	0.68 [0.11 − 4.27]
Paclitaxel	0.65 [0.19 − 2.79]
Vinflunine	0.59 [0.12 − 2.79]
Amrubicin	0.5 [0.11 − 2.36]
Pemetrexed+Matuzumab	0.33 [0.01 − 6.64]
Vinorelbine	0.29 [0.01 − 9.29]
Entinostat+Erlotinib	0.24 [0.02 − 2.1]
Erlotinib+Dalotuzumab	0.23 [0.01 − 3.7]
Pralatrexate	0.22 [0.02 − 1.86]
Cetuximab	0.19 [0.01 − 5.94]
Placebo	0.15 [0.04 − 0.55]
AXL1717	0.04 [0 − 1.02]

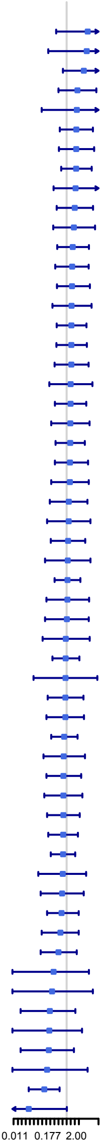

Treatments vs. Docetaxel+Ramucirumab	OR[CrI 95%]
Cabozantinib	4.41 [0.37 − 166.67]
Docetaxel+PX−866	4.09 [0.17 − 95.77]
Paclitaxel+Bevacizumab	3.15 [0.63 − 19.28]
Docetaxel+Tamoxifen	1.84 [0.45 − 7.81]
Erlotinib+Cabozantinib	1.74 [0.1 − 66.35]
Pemetrexed+Vandetanib	1.67 [0.46 − 6.47]
Docetaxel+Aflibercept	1.67 [0.51 − 5.61]
Pemetrexed+Erlotinib	1.65 [0.59 − 4.98]
Erlotinib+Pazopanib	1.57 [0.28 − 10.88]
Erlotinib+Bevacizumab	1.47 [0.38 − 5.97]
Erlotinib+Fulvestrant	1.36 [0.28 − 7.07]
Dacomitinib	1.23 [0.42 − 3.91]
Pembrolizumab	1.18 [0.35 − 3.94]
Docetaxel+Selumetinib	1.17 [0.4 − 4.34]
Afatinib	1.12 [0.26 − 5.1]
Nivolumab	1.11 [0.4 − 3.16]
Erlotinib+Sunitinib	1.1 [0.33 − 3.9]
Docetaxel+Vandetanib	1.1 [0.39 − 3.27]
Pemetrexed+Sunitinib	1.04 [0.2 − 5.8]
Erlotinib+Tivantinib	1.03 [0.34 − 3.15]
Erlotinib+Figitumumab	1.01 [0.24 − 4.35]
Vandetanib	0.97 [0.34 − 3.75]
Pemetrexed+Cetuximab	0.96 [0.25 − 4.1]
Tegafur/uracil+Gefitinib	0.89 [0.21 − 3.59]
Nab−Paclitaxel	0.88 [0.17 − 4.79]
Icotinib	0.83 [0.23 − 3]
Pemetrexed+Sorafenib	0.81 [0.14 − 4.91]
Gefitinib	0.79 [0.35 − 1.8]
Erlotinib+Everolimus	0.78 [0.16 − 4.25]
Sunitinib	0.75 [0.14 − 4.15]
Docetaxel+Nintedanib	0.74 [0.21 − 2.59]
Docetaxel+Erlotinib	0.69 [0.11 − 4.6]
Erlotinib	0.68 [0.29 − 1.67]
Selumetinib	0.67 [0.05 − 9.1]
Docetaxel+Cetuximab	0.66 [0.16 − 2.71]
Pemetrexed+Nintedanib	0.66 [0.18 − 2.52]
Pemetrexed	0.6 [0.27 − 1.43]
Trametinib	0.57 [0.12 − 3.07]
Topotecan	0.56 [0.17 − 1.83]
Erlotinib+Onartuzumab	0.56 [0.16 − 2.1]
Gefitinib+Nimotuzumab	0.56 [0.13 − 2.38]
Atezolizumab	0.55 [0.2 − 1.53]
Docetaxel	0.55 [0.27 − 1.13]
Erlotinib+R1507	0.53 [0.08 − 3.43]
Erlotinib+Sorafenib	0.5 [0.1 − 2.71]
Paclitaxel	0.48 [0.17 − 1.7]
Vinflunine	0.43 [0.11 − 1.69]
Amrubicin	0.37 [0.1 − 1.45]
Pemetrexed+Matuzumab	0.25 [0.01 − 4.43]
Vinorelbine	0.22 [0.01 − 6.25]
Entinostat+Erlotinib	0.18 [0.02 − 1.34]
Erlotinib+Dalotuzumab	0.17 [0 − 2.44]
Pralatrexate	0.17 [0.02 − 1.2]
Cetuximab	0.14 [0 − 4.08]
Placebo	0.11 [0.03 − 0.33]
AXL1717	0.03 [0 − 0.69]

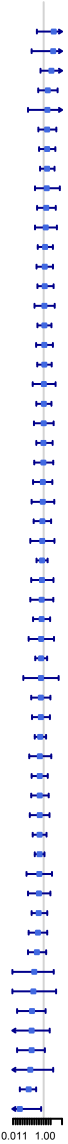

Treatments vs. Afatinib	OR[CrI 95%]
Cabozantinib	3.9 [0.27 − 152.93]
Docetaxel+PX−866	3.63 [0.13 − 100.58]
Paclitaxel+Bevacizumab	2.82 [0.4 − 22.31]
Docetaxel+Tamoxifen	1.64 [0.27 − 9.84]
Erlotinib+Cabozantinib	1.55 [0.08 − 64.65]
Docetaxel+Aflibercept	1.5 [0.29 − 7.37]
Pemetrexed+Vandetanib	1.49 [0.29 − 7.6]
Pemetrexed+Erlotinib	1.48 [0.36 − 6.05]
Erlotinib+Pazopanib	1.41 [0.2 − 11.12]
Erlotinib+Bevacizumab	1.3 [0.26 − 6.21]
Erlotinib+Fulvestrant	1.21 [0.2 − 7.35]
Dacomitinib	1.1 [0.27 − 4.45]
Docetaxel+Selumetinib	1.05 [0.22 − 5.56]
Pembrolizumab	1.05 [0.2 − 5.11]
Nivolumab	0.99 [0.22 − 4.23]
Docetaxel+Vandetanib	0.98 [0.21 − 4.4]
Erlotinib+Sunitinib	0.98 [0.22 − 4.26]
Pemetrexed+Sunitinib	0.93 [0.13 − 6.44]
Erlotinib+Tivantinib	0.91 [0.23 − 3.49]
Erlotinib+Figitumumab	0.9 [0.17 − 4.53]
Docetaxel+Ramucirumab	0.89 [0.2 − 3.8]
Vandetanib	0.88 [0.22 − 4.11]
Pemetrexed+Cetuximab	0.87 [0.16 − 4.76]
Tegafur/uracil+Gefitinib	0.78 [0.13 − 4.31]
Nab−Paclitaxel	0.78 [0.11 − 5.42]
Icotinib	0.74 [0.14 − 3.59]
Pemetrexed+Sorafenib	0.72 [0.1 − 5.36]
Gefitinib	0.71 [0.19 − 2.49]
Erlotinib+Everolimus	0.7 [0.11 − 4.45]
Sunitinib	0.66 [0.1 − 4.66]
Docetaxel+Nintedanib	0.66 [0.12 − 3.35]
Docetaxel+Erlotinib	0.62 [0.08 − 4.68]
Erlotinib	0.61 [0.18 − 1.96]
Selumetinib	0.59 [0.04 − 9.31]
Pemetrexed+Nintedanib	0.59 [0.11 − 2.96]
Docetaxel+Cetuximab	0.59 [0.1 − 3.42]
Pemetrexed	0.54 [0.14 − 1.9]
Trametinib	0.5 [0.07 − 3.65]
Topotecan	0.5 [0.1 − 2.37]
Gefitinib+Nimotuzumab	0.5 [0.08 − 2.77]
Erlotinib+Onartuzumab	0.5 [0.11 − 2.29]
Atezolizumab	0.49 [0.11 − 2.09]
Docetaxel	0.49 [0.13 − 1.74]
Erlotinib+R1507	0.47 [0.06 − 3.59]
Erlotinib+Sorafenib	0.44 [0.07 − 2.8]
Paclitaxel	0.43 [0.1 − 2.18]
Vinflunine	0.38 [0.07 − 2.13]
Amrubicin	0.33 [0.06 − 1.83]
Pemetrexed+Matuzumab	0.22 [0.01 − 4.69]
Vinorelbine	0.19 [0.01 − 6.45]
Entinostat+Erlotinib	0.16 [0.01 − 1.36]
Erlotinib+Dalotuzumab	0.15 [0 − 2.46]
Pralatrexate	0.14 [0.01 − 1.26]
Cetuximab	0.12 [0 − 4.03]
Placebo	0.1 [0.02 − 0.4]
AXL1717	0.03 [0 − 0.71]

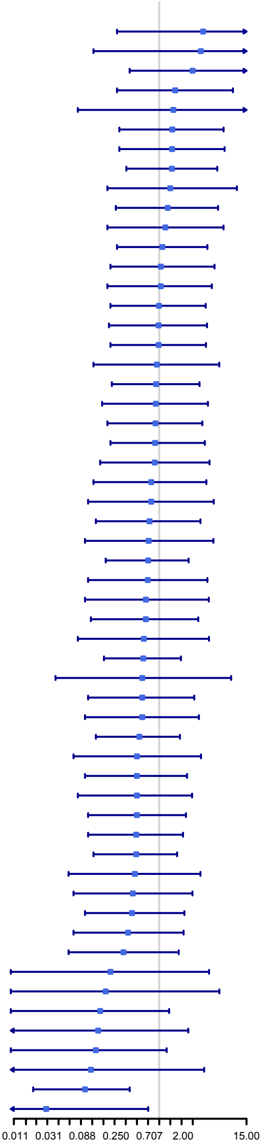

Treatments vs. Cabozantinib	OR[CrI 95%]
Docetaxel+PX−866	0.87 [0.01 − 48.86]
Paclitaxel+Bevacizumab	0.72 [0.01 − 14.06]
Docetaxel+Tamoxifen	0.41 [0.01 − 6.76]
Erlotinib+Cabozantinib	0.41 [0.04 − 2.76]
Pemetrexed+Vandetanib	0.38 [0.01 − 5.46]
Pemetrexed+Erlotinib	0.38 [0.01 − 4.53]
Docetaxel+Aflibercept	0.38 [0.01 − 5]
Erlotinib+Pazopanib	0.36 [0.01 − 6.69]
Erlotinib+Bevacizumab	0.34 [0.01 − 4.48]
Erlotinib+Fulvestrant	0.31 [0.01 − 4.97]
Dacomitinib	0.29 [0.01 − 3.28]
Docetaxel+Selumetinib	0.27 [0.01 − 3.71]
Pembrolizumab	0.26 [0.01 − 3.55]
Afatinib	0.26 [0.01 − 3.67]
Erlotinib+Sunitinib	0.25 [0.01 − 3.07]
Nivolumab	0.25 [0.01 − 3.14]
Docetaxel+Vandetanib	0.25 [0.01 − 3.15]
Pemetrexed+Sunitinib	0.24 [0.01 − 3.92]
Erlotinib+Tivantinib	0.24 [0.01 − 2.71]
Vandetanib	0.23 [0.01 − 2.86]
Erlotinib+Figitumumab	0.23 [0.01 − 3.2]
Docetaxel+Ramucirumab	0.23 [0.01 − 2.72]
Pemetrexed+Cetuximab	0.22 [0.01 − 3.17]
Nab−Paclitaxel	0.2 [0 − 3.26]
Tegafur/uracil+Gefitinib	0.2 [0 − 2.86]
Icotinib	0.19 [0 − 2.49]
Gefitinib	0.18 [0.01 − 1.98]
Pemetrexed+Sorafenib	0.18 [0 − 3.23]
Erlotinib+Everolimus	0.18 [0 − 2.85]
Sunitinib	0.17 [0 − 2.91]
Docetaxel+Nintedanib	0.17 [0 − 2.43]
Docetaxel+Erlotinib	0.16 [0 − 2.85]
Erlotinib	0.16 [0 − 1.64]
Pemetrexed+Nintedanib	0.15 [0 − 2.06]
Docetaxel+Cetuximab	0.15 [0 − 2.37]
Selumetinib	0.14 [0 − 4.82]
Pemetrexed	0.14 [0 − 1.51]
Trametinib	0.13 [0 − 2.22]
Erlotinib+Onartuzumab	0.13 [0 − 1.62]
Gefitinib+Nimotuzumab	0.13 [0 − 1.84]
Topotecan	0.13 [0 − 1.68]
Atezolizumab	0.13 [0 − 1.54]
Docetaxel	0.13 [0 − 1.38]
Erlotinib+R1507	0.12 [0 − 2.38]
Erlotinib+Sorafenib	0.11 [0 − 1.79]
Paclitaxel	0.11 [0 − 1.48]
Vinflunine	0.1 [0 − 1.45]
Amrubicin	0.08 [0 − 1.21]
Pemetrexed+Matuzumab	0.05 [0 − 2.18]
Vinorelbine	0.05 [0 − 3.1]
Entinostat+Erlotinib	0.04 [0 − 0.81]
Pralatrexate	0.04 [0 − 0.73]
Erlotinib+Dalotuzumab	0.03 [0 − 1.31]
Cetuximab	0.03 [0 − 2.05]
Placebo	0.02 [0 − 0.29]
AXL1717	0.01 [0 − 0.34]

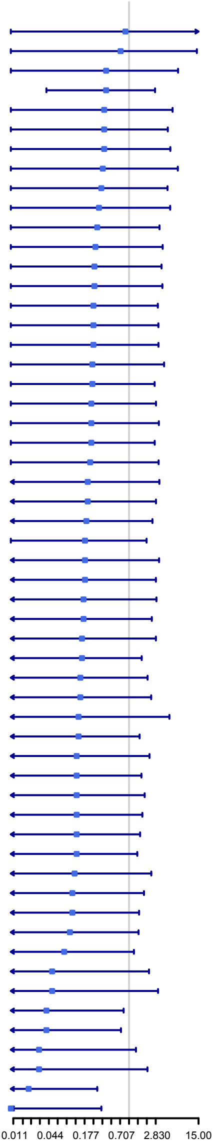

Treatments vs. Erlotinib+Cabozantinib	OR[CrI 95%]
Cabozantinib	2.46 [0.36 − 23.22]
Docetaxel+PX−866	2.31 [0.02 − 160.45]
Paclitaxel+Bevacizumab	1.83 [0.04 − 47.75]
Docetaxel+Tamoxifen	1.05 [0.03 − 22.29]
Pemetrexed+Vandetanib	0.96 [0.02 − 19.47]
Docetaxel+Aflibercept	0.96 [0.02 − 17.89]
Pemetrexed+Erlotinib	0.95 [0.03 − 17.06]
Erlotinib+Pazopanib	0.91 [0.02 − 22]
Erlotinib+Bevacizumab	0.85 [0.02 − 16.18]
Erlotinib+Fulvestrant	0.77 [0.02 − 17.08]
Dacomitinib	0.71 [0.02 − 12.18]
Docetaxel+Selumetinib	0.69 [0.02 − 12.91]
Pembrolizumab	0.67 [0.02 − 12.43]
Afatinib	0.64 [0.02 − 12.69]
Nivolumab	0.64 [0.02 − 11.3]
Erlotinib+Sunitinib	0.64 [0.02 − 10.9]
Docetaxel+Vandetanib	0.64 [0.02 − 11.54]
Pemetrexed+Sunitinib	0.6 [0.01 − 13.42]
Erlotinib+Tivantinib	0.59 [0.02 − 9.87]
Vandetanib	0.58 [0.02 − 10.52]
Docetaxel+Ramucirumab	0.57 [0.02 − 10.16]
Erlotinib+Figitumumab	0.57 [0.01 − 11.19]
Pemetrexed+Cetuximab	0.56 [0.01 − 11.27]
Tegafur/uracil+Gefitinib	0.51 [0.01 − 9.95]
Nab−Paclitaxel	0.5 [0.01 − 12.23]
Icotinib	0.48 [0.01 − 8.77]
Pemetrexed+Sorafenib	0.46 [0.01 − 11.63]
Gefitinib	0.46 [0.01 − 7.36]
Erlotinib+Everolimus	0.45 [0.01 − 9.94]
Sunitinib	0.43 [0.01 − 9.91]
Docetaxel+Nintedanib	0.42 [0.01 − 8.26]
Docetaxel+Erlotinib	0.4 [0.01 − 9.36]
Erlotinib	0.39 [0.01 − 6.09]
Pemetrexed+Nintedanib	0.38 [0.01 − 7.56]
Docetaxel+Cetuximab	0.37 [0.01 − 8]
Selumetinib	0.37 [0.01 − 15.52]
Pemetrexed	0.35 [0.01 − 5.65]
Trametinib	0.33 [0.01 − 7.43]
Erlotinib+Onartuzumab	0.32 [0.01 − 5.99]
Atezolizumab	0.32 [0.01 − 5.5]
Topotecan	0.32 [0.01 − 6.1]
Docetaxel	0.32 [0.01 − 5.08]
Gefitinib+Nimotuzumab	0.32 [0.01 − 6.61]
Erlotinib+R1507	0.3 [0.01 − 7.55]
Erlotinib+Sorafenib	0.28 [0.01 − 6.24]
Paclitaxel	0.28 [0.01 − 5.37]
Vinflunine	0.25 [0.01 − 4.97]
Amrubicin	0.22 [0 − 4.35]
Pemetrexed+Matuzumab	0.14 [0 − 6.37]
Vinorelbine	0.12 [0 − 10.58]
Entinostat+Erlotinib	0.1 [0 − 2.8]
Pralatrexate	0.09 [0 − 2.47]
Erlotinib+Dalotuzumab	0.09 [0 − 4.01]
Cetuximab	0.07 [0 − 6.28]
Placebo	0.06 [0 − 1.07]
AXL1717	0.02 [0 − 1.13]

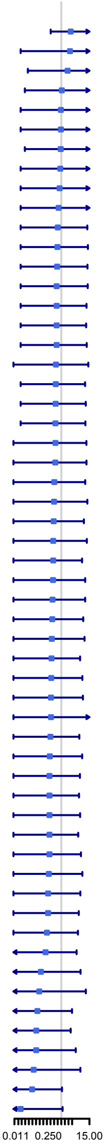

Treatments vs. Placebo	OR[CrI 95%]
Cabozantinib	41.1 [3.43 − 1559.31]
Docetaxel+PX−866	38.24 [1.56 − 928.9]
Paclitaxel+Bevacizumab	29.49 [5.51 − 192.48]
Docetaxel+Tamoxifen	17.17 [3.86 − 81.45]
Erlotinib+Cabozantinib	16.36 [0.93 − 638.42]
Pemetrexed+Vandetanib	15.66 [4.17 − 63.12]
Docetaxel+Aflibercept	15.63 [4.36 − 58.73]
Pemetrexed+Erlotinib	15.5 [5.36 − 47.8]
Erlotinib+Pazopanib	14.57 [2.66 − 102.41]
Erlotinib+Bevacizumab	13.61 [3.69 − 54.43]
Erlotinib+Fulvestrant	12.63 [2.69 − 66.09]
Dacomitinib	11.48 [4.46 − 33.25]
Docetaxel+Selumetinib	10.99 [3.35 − 44.08]
Pembrolizumab	10.94 [2.98 − 41.1]
Afatinib	10.42 [2.52 − 47.09]
Nivolumab	10.3 [3.41 − 33.65]
Docetaxel+Vandetanib	10.27 [3.34 − 34.61]
Erlotinib+Sunitinib	10.26 [3.16 − 35.59]
Pemetrexed+Sunitinib	9.73 [1.82 − 56.43]
Erlotinib+Tivantinib	9.57 [3.35 − 28.85]
Erlotinib+Figitumumab	9.33 [2.28 − 40.21]
Docetaxel+Ramucirumab	9.29 [3.04 − 29.31]
Vandetanib	9.16 [3.2 − 33.52]
Pemetrexed+Cetuximab	9.03 [2.2 − 40.21]
Tegafur/uracil+Gefitinib	8.27 [1.99 − 34.33]
Nab−Paclitaxel	8.23 [1.52 − 46.39]
Icotinib	7.74 [2.15 − 28.33]
Pemetrexed+Sorafenib	7.54 [1.28 − 47.99]
Gefitinib	7.36 [3.28 − 17.71]
Erlotinib+Everolimus	7.32 [1.48 − 39.88]
Sunitinib	7 [1.29 − 40.29]
Docetaxel+Nintedanib	6.86 [1.82 − 26.87]
Docetaxel+Erlotinib	6.51 [1.03 − 42.44]
Erlotinib	6.31 [2.85 − 15.52]
Selumetinib	6.29 [0.46 − 86.14]
Pemetrexed+Nintedanib	6.17 [1.65 − 24.56]
Docetaxel+Cetuximab	6.17 [1.39 − 27.85]
Pemetrexed	5.61 [2.37 − 14.41]
Trametinib	5.29 [1.02 − 31.16]
Gefitinib+Nimotuzumab	5.2 [1.22 − 22.71]
Topotecan	5.19 [1.47 − 19.49]
Erlotinib+Onartuzumab	5.18 [1.54 − 19.14]
Atezolizumab	5.1 [1.7 − 16.3]
Docetaxel	5.07 [2.18 − 12.72]
Erlotinib+R1507	4.97 [0.73 − 31.85]
Erlotinib+Sorafenib	4.65 [0.94 − 25.13]
Paclitaxel	4.53 [1.42 − 17.44]
Vinflunine	4.03 [0.94 − 17.69]
Amrubicin	3.48 [0.82 − 14.85]
Pemetrexed+Matuzumab	2.3 [0.07 − 43.03]
Vinorelbine	2.01 [0.07 − 63.12]
Entinostat+Erlotinib	1.67 [0.16 − 12.45]
Erlotinib+Dalotuzumab	1.6 [0.04 − 22.76]
Pralatrexate	1.53 [0.15 − 10.9]
Cetuximab	1.24 [0.04 − 39.41]
AXL1717	0.28 [0.01 − 6.68]

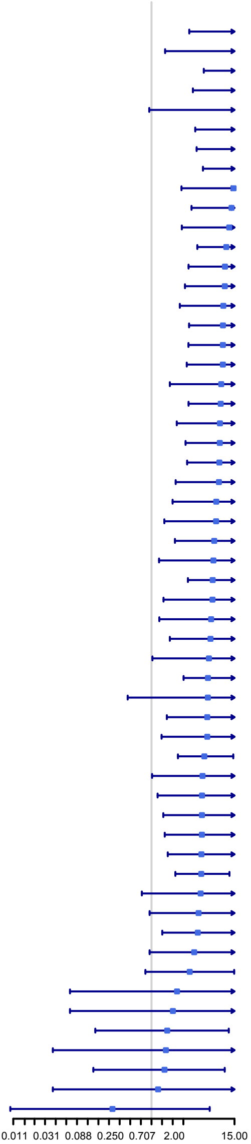

(d) Serious adverse events

Treatments vs. Docetaxel	OR[CrI 95%]
Selumetinib	0.13 [0 − 1.42]
Docetaxel+Erlotinib	0.24 [0.06 − 0.93]
Erlotinib+Dalotuzumab	0.35 [0.08 − 1.32]
Placebo	0.44 [0.15 − 1.33]
Pralatrexate	0.45 [0.15 − 1.57]
Erlotinib	0.45 [0.19 − 1.16]
Pemetrexed+Nintedanib	0.47 [0.2 − 1.13]
Afatinib	0.48 [0.18 − 1.38]
Pemetrexed+Vandetanib	0.48 [0.2 − 1.2]
Dacomitinib	0.51 [0.2 − 1.39]
Entinostat+Erlotinib	0.52 [0.15 − 1.88]
Pemetrexed	0.53 [0.26 − 1.09]
Erlotinib+Sunitinib	0.55 [0.21 − 1.53]
Erlotinib+Bevacizumab	0.56 [0.21 − 1.63]
Erlotinib+Tivantinib	0.57 [0.21 − 1.59]
Vandetanib	0.61 [0.23 − 1.73]
Erlotinib+Pazopanib	0.64 [0.19 − 2.16]
Icotinib	0.64 [0.25 − 1.64]
Erlotinib+Onartuzumab	0.66 [0.19 − 2.37]
Amrubicin	0.68 [0.29 − 1.52]
Gefitinib	0.76 [0.56 − 1.06]
Sunitinib	0.8 [0.27 − 2.77]
Pemetrexed+Cetuximab	0.87 [0.37 − 2.06]
Pemetrexed+Erlotinib	0.91 [0.38 − 2.17]
Erlotinib+R1507	0.93 [0.21 − 3.86]
Pembrolizumab	0.97 [0.59 − 1.59]
Erlotinib+Figitumumab	0.97 [0.35 − 2.82]
Docetaxel+Ramucirumab	0.99 [0.69 − 1.43]
Nivolumab	1.02 [0.68 − 1.47]
Atezolizumab	1.03 [0.72 − 1.48]
Docetaxel+Nintedanib	1.13 [0.73 − 1.74]
Docetaxel+Vandetanib	1.22 [0.78 − 1.89]
Pemetrexed+Sunitinib	1.27 [0.4 − 4.42]
Pemetrexed+Sorafenib	1.33 [0.37 − 4.91]
Erlotinib+Everolimus	1.47 [0.42 − 5.37]
Docetaxel+Cetuximab	1.65 [0.94 − 2.96]
Docetaxel+Aflibercept	1.74 [1.08 − 2.72]
Docetaxel+Selumetinib	2.15 [1.38 − 3.43]
Trametinib	2.17 [0.88 − 5.66]
Cabozantinib	2.2 [0.61 − 9.4]
Cetuximab	2.41 [0.51 − 13.68]
Erlotinib+Cabozantinib	2.42 [0.64 − 10]

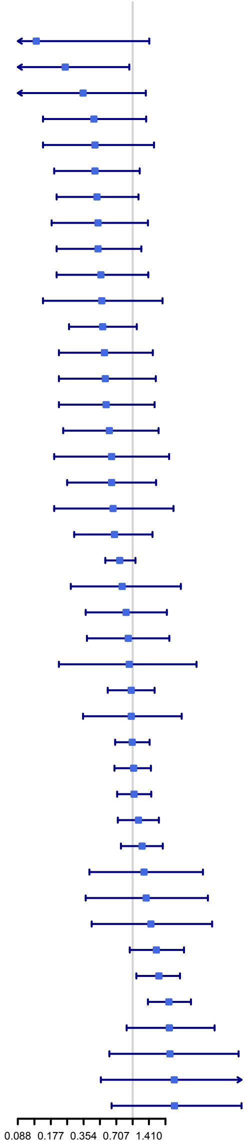

Treatments vs. Pemetrexed	OR[CrI 95%]
Selumetinib	0.26 [0.01 − 2.32]
Docetaxel+Erlotinib	0.44 [0.12 − 1.47]
Erlotinib+Dalotuzumab	0.65 [0.17 − 2.1]
Placebo	0.81 [0.34 − 2.03]
Erlotinib	0.85 [0.44 − 1.71]
Pralatrexate	0.85 [0.3 − 2.39]
Pemetrexed+Nintedanib	0.88 [0.53 − 1.43]
Afatinib	0.89 [0.39 − 2.06]
Pemetrexed+Vandetanib	0.9 [0.54 − 1.5]
Dacomitinib	0.95 [0.45 − 2.06]
Entinostat+Erlotinib	0.97 [0.33 − 2.94]
Erlotinib+Sunitinib	1.04 [0.48 − 2.26]
Erlotinib+Bevacizumab	1.05 [0.46 − 2.49]
Erlotinib+Tivantinib	1.06 [0.48 − 2.38]
Vandetanib	1.14 [0.49 − 2.56]
Erlotinib+Pazopanib	1.19 [0.43 − 3.33]
Icotinib	1.19 [0.39 − 3.73]
Erlotinib+Onartuzumab	1.25 [0.44 − 3.64]
Amrubicin	1.27 [0.42 − 3.8]
Gefitinib	1.43 [0.69 − 2.88]
Sunitinib	1.53 [0.59 − 3.91]
Pemetrexed+Cetuximab	1.62 [0.99 − 2.68]
Erlotinib+R1507	1.71 [0.48 − 6.49]
Pemetrexed+Erlotinib	1.72 [1 − 2.86]
Pembrolizumab	1.82 [0.75 − 4.36]
Erlotinib+Figitumumab	1.84 [0.79 − 4.22]
Docetaxel+Ramucirumab	1.86 [0.83 − 4.17]
Docetaxel	1.87 [0.92 − 3.83]
Nivolumab	1.9 [0.8 − 4.3]
Atezolizumab	1.94 [0.87 − 4.33]
Docetaxel+Nintedanib	2.11 [0.91 − 4.81]
Docetaxel+Vandetanib	2.28 [0.97 − 5.22]
Pemetrexed+Sunitinib	2.39 [0.89 − 6.29]
Pemetrexed+Sorafenib	2.45 [0.85 − 7.24]
Erlotinib+Everolimus	2.72 [0.88 − 8.34]
Docetaxel+Cetuximab	3.12 [1.23 − 7.82]
Docetaxel+Aflibercept	3.23 [1.37 − 7.54]
Docetaxel+Selumetinib	4.04 [1.65 − 9.48]
Trametinib	4.08 [1.18 − 13.14]
Cabozantinib	4.16 [1.26 − 15.77]
Erlotinib+Cabozantinib	4.44 [1.38 − 17.01]
Cetuximab	4.54 [1.14 − 19.43]

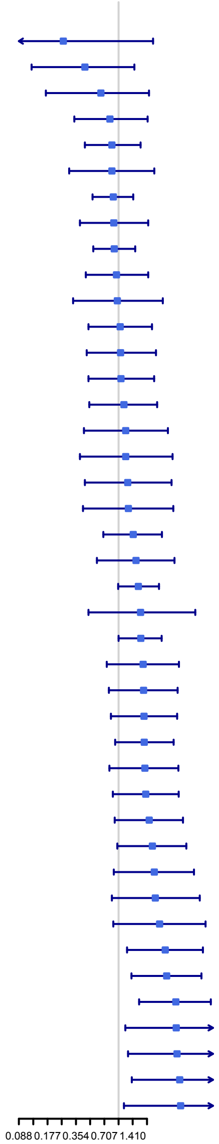

**Treatments vs. Erlotinib**	**OR[CrI 95%]**
Selumetinib	0.31 [0.01 − 3.03]
Docetaxel+Erlotinib	0.52 [0.18 − 1.45]
Erlotinib+Dalotuzumab	0.76 [0.27 − 2.06]
Placebo	0.96 [0.52 − 1.78]
Pralatrexate	1 [0.49 − 2.03]
Pemetrexed+Nintedanib	1.03 [0.45 − 2.37]
Afatinib	1.05 [0.65 − 1.72]
Pemetrexed+Vandetanib	1.06 [0.45 − 2.51]
Dacomitinib	1.12 [0.77 − 1.64]
Entinostat+Erlotinib	1.14 [0.51 − 2.57]
Pemetrexed	1.18 [0.59 − 2.28]
Erlotinib+Sunitinib	1.23 [0.82 − 1.79]
Erlotinib+Tivantinib	1.24 [0.8 − 1.95]
Erlotinib+Bevacizumab	1.24 [0.76 − 2.02]
Vandetanib	1.34 [0.84 − 2.15]
Erlotinib+Pazopanib	1.38 [0.67 − 2.94]
Icotinib	1.4 [0.39 − 4.98]
Erlotinib+Onartuzumab	1.45 [0.67 − 3.28]
Amrubicin	1.47 [0.43 − 5.11]
Gefitinib	1.67 [0.65 − 4.1]
Sunitinib	1.78 [0.59 − 5.57]
Pemetrexed+Cetuximab	1.91 [0.83 − 4.34]
Pemetrexed+Erlotinib	2.02 [0.98 − 4.01]
Erlotinib+R1507	2.02 [0.7 − 5.81]
Pembrolizumab	2.14 [0.74 − 5.91]
Erlotinib+Figitumumab	2.14 [1.31 − 3.55]
Docetaxel+Ramucirumab	2.19 [0.8 − 5.67]
Docetaxel	2.2 [0.86 − 5.21]
Nivolumab	2.23 [0.81 − 5.83]
Atezolizumab	2.26 [0.83 − 5.85]
Docetaxel+Nintedanib	2.49 [0.89 − 6.71]
Docetaxel+Vandetanib	2.67 [0.94 − 7.22]
Pemetrexed+Sunitinib	2.77 [0.89 − 9.04]
Pemetrexed+Sorafenib	2.94 [0.75 − 10.17]
Erlotinib+Everolimus	3.23 [1.36 − 7.65]
Docetaxel+Cetuximab	3.61 [1.25 − 10.78]
Docetaxel+Aflibercept	3.78 [1.35 − 10.26]
Docetaxel+Selumetinib	4.78 [1.7 − 13]
Trametinib	4.86 [1.26 − 17.5]
Cabozantinib	4.91 [1.79 − 14.82]
Erlotinib+Cabozantinib	5.24 [1.9 − 16.33]
Cetuximab	5.34 [1.11 − 25.15]

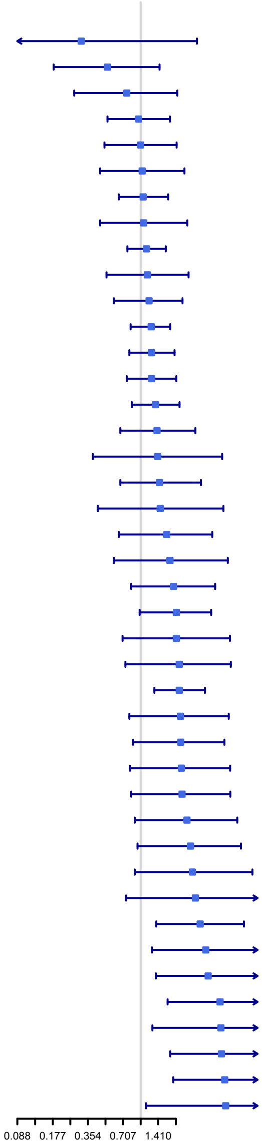

Treatments vs. Gefitinib	OR[CrI 95%]
Selumetinib	0.18 [0.01 − 1.89]
Docetaxel+Erlotinib	0.31 [0.08 − 1.23]
Erlotinib+Dalotuzumab	0.46 [0.1 − 1.72]
Placebo	0.57 [0.19 − 1.74]
Pralatrexate	0.59 [0.19 − 2.04]
Erlotinib	0.6 [0.24 − 1.53]
Pemetrexed+Nintedanib	0.62 [0.26 − 1.52]
Afatinib	0.63 [0.23 − 1.81]
Pemetrexed+Vandetanib	0.63 [0.27 − 1.6]
Dacomitinib	0.67 [0.25 − 1.82]
Entinostat+Erlotinib	0.68 [0.2 − 2.47]
Pemetrexed	0.7 [0.35 − 1.45]
Erlotinib+Sunitinib	0.74 [0.27 − 1.99]
Erlotinib+Bevacizumab	0.75 [0.26 − 2.13]
Erlotinib+Tivantinib	0.75 [0.27 − 2.1]
Vandetanib	0.81 [0.29 − 2.29]
Erlotinib+Pazopanib	0.83 [0.25 − 2.79]
Icotinib	0.84 [0.35 − 2.02]
Erlotinib+Onartuzumab	0.87 [0.25 − 3.1]
Amrubicin	0.89 [0.36 − 2.11]
Sunitinib	1.06 [0.34 − 3.54]
Pemetrexed+Cetuximab	1.14 [0.48 − 2.73]
Pemetrexed+Erlotinib	1.2 [0.5 − 2.9]
Erlotinib+R1507	1.22 [0.28 − 5.09]
Pembrolizumab	1.28 [0.7 − 2.27]
Erlotinib+Figitumumab	1.3 [0.45 − 3.67]
Docetaxel+Ramucirumab	1.31 [0.79 − 2.1]
Docetaxel	1.32 [0.94 − 1.78]
Nivolumab	1.34 [0.79 − 2.14]
Atezolizumab	1.37 [0.83 − 2.14]
Docetaxel+Nintedanib	1.5 [0.85 − 2.5]
Docetaxel+Vandetanib	1.61 [0.92 − 2.71]
Pemetrexed+Sunitinib	1.69 [0.52 − 5.92]
Pemetrexed+Sorafenib	1.73 [0.48 − 6.4]
Erlotinib+Everolimus	1.94 [0.54 − 6.96]
Docetaxel+Cetuximab	2.19 [1.11 − 4.15]
Docetaxel+Aflibercept	2.28 [1.27 − 3.88]
Docetaxel+Selumetinib	2.81 [1.63 − 4.91]
Trametinib	2.85 [1.1 − 7.74]
Cabozantinib	2.9 [0.79 − 12.39]
Erlotinib+Cabozantinib	3.18 [0.83 − 13.12]
Cetuximab	3.18 [0.66 − 17.67]

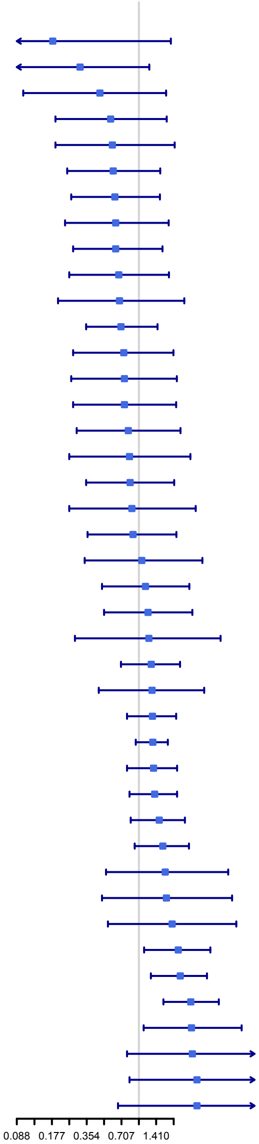

Treatments vs. Nivolumab	OR[CrI 95%]
Selumetinib	0.13 [0 − 1.47]
Docetaxel+Erlotinib	0.23 [0.05 − 0.96]
Erlotinib+Dalotuzumab	0.34 [0.08 − 1.39]
Placebo	0.43 [0.14 − 1.4]
Erlotinib	0.45 [0.17 − 1.23]
Pralatrexate	0.45 [0.13 − 1.63]
Pemetrexed+Nintedanib	0.46 [0.18 − 1.27]
Pemetrexed+Vandetanib	0.47 [0.18 − 1.3]
Afatinib	0.47 [0.16 − 1.46]
Dacomitinib	0.51 [0.18 − 1.48]
Entinostat+Erlotinib	0.51 [0.14 − 1.96]
Pemetrexed	0.52 [0.23 − 1.25]
Erlotinib+Sunitinib	0.55 [0.19 − 1.62]
Erlotinib+Bevacizumab	0.56 [0.19 − 1.73]
Erlotinib+Tivantinib	0.56 [0.19 − 1.66]
Vandetanib	0.6 [0.2 − 1.85]
Erlotinib+Pazopanib	0.62 [0.18 − 2.2]
Icotinib	0.63 [0.24 − 1.75]
Erlotinib+Onartuzumab	0.66 [0.18 − 2.46]
Amrubicin	0.67 [0.27 − 1.62]
Gefitinib	0.75 [0.47 − 1.27]
Sunitinib	0.79 [0.25 − 2.9]
Pemetrexed+Cetuximab	0.86 [0.33 − 2.25]
Pemetrexed+Erlotinib	0.89 [0.35 − 2.39]
Erlotinib+R1507	0.92 [0.2 − 4.01]
Pembrolizumab	0.95 [0.52 − 1.81]
Erlotinib+Figitumumab	0.96 [0.32 − 3]
Docetaxel+Ramucirumab	0.97 [0.59 − 1.68]
Docetaxel	0.98 [0.68 − 1.46]
Atezolizumab	1.01 [0.61 − 1.75]
Docetaxel+Nintedanib	1.11 [0.63 − 2.02]
Docetaxel+Vandetanib	1.19 [0.68 − 2.19]
Pemetrexed+Sunitinib	1.24 [0.38 − 4.69]
Pemetrexed+Sorafenib	1.3 [0.34 − 5.11]
Erlotinib+Everolimus	1.43 [0.39 − 5.67]
Docetaxel+Cetuximab	1.63 [0.82 − 3.3]
Docetaxel+Aflibercept	1.69 [0.94 − 3.15]
Docetaxel+Selumetinib	2.09 [1.19 − 3.95]
Trametinib	2.11 [0.84 − 6.08]
Cabozantinib	2.16 [0.56 − 9.76]
Cetuximab	2.36 [0.48 − 14.32]
Erlotinib+Cabozantinib	2.37 [0.6 − 10.3]

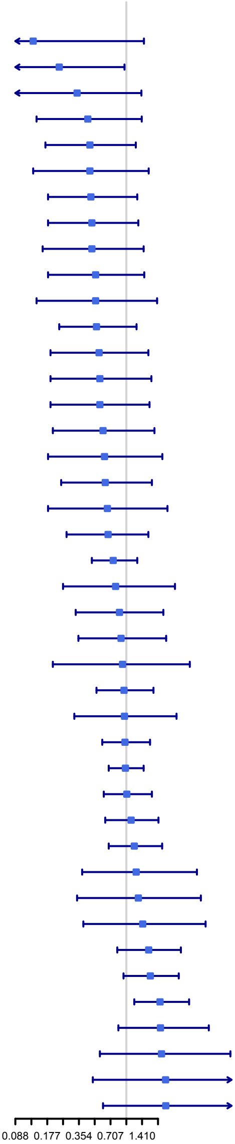

Treatments vs. Pembrolizumab	OR[CrI 95%]
Selumetinib	0.14 [0 − 1.55]
Docetaxel+Erlotinib	0.24 [0.05 − 1.06]
Erlotinib+Dalotuzumab	0.36 [0.08 − 1.52]
Placebo	0.45 [0.14 − 1.54]
Erlotinib	0.47 [0.17 − 1.35]
Pralatrexate	0.47 [0.13 − 1.77]
Pemetrexed+Nintedanib	0.48 [0.18 − 1.33]
Afatinib	0.5 [0.16 − 1.6]
Pemetrexed+Vandetanib	0.5 [0.18 − 1.41]
Dacomitinib	0.53 [0.18 − 1.65]
Entinostat+Erlotinib	0.53 [0.14 − 2.14]
Pemetrexed	0.55 [0.23 − 1.33]
Erlotinib+Sunitinib	0.57 [0.19 − 1.76]
Erlotinib+Bevacizumab	0.58 [0.19 − 1.86]
Erlotinib+Tivantinib	0.59 [0.19 − 1.84]
Vandetanib	0.63 [0.2 − 2]
Erlotinib+Pazopanib	0.66 [0.18 − 2.44]
Icotinib	0.66 [0.23 − 1.9]
Erlotinib+Onartuzumab	0.68 [0.18 − 2.7]
Amrubicin	0.7 [0.26 − 1.77]
Gefitinib	0.78 [0.44 − 1.43]
Sunitinib	0.83 [0.25 − 3.1]
Pemetrexed+Cetuximab	0.89 [0.33 − 2.47]
Pemetrexed+Erlotinib	0.94 [0.35 − 2.59]
Erlotinib+R1507	0.95 [0.21 − 4.36]
Erlotinib+Figitumumab	1 [0.32 − 3.27]
Docetaxel+Ramucirumab	1.03 [0.55 − 1.9]
Docetaxel	1.03 [0.63 − 1.69]
Nivolumab	1.05 [0.55 − 1.93]
Atezolizumab	1.06 [0.58 − 1.96]
Docetaxel+Nintedanib	1.16 [0.61 − 2.26]
Docetaxel+Vandetanib	1.26 [0.65 − 2.43]
Pemetrexed+Sunitinib	1.31 [0.37 − 5.06]
Pemetrexed+Sorafenib	1.37 [0.34 − 5.54]
Erlotinib+Everolimus	1.53 [0.39 − 5.95]
Docetaxel+Cetuximab	1.71 [0.8 − 3.68]
Docetaxel+Aflibercept	1.79 [0.89 − 3.49]
Docetaxel+Selumetinib	2.21 [1.14 − 4.41]
Cabozantinib	2.25 [0.57 − 10.95]
Trametinib	2.26 [0.81 − 6.6]
Cetuximab	2.47 [0.5 − 15.36]
Erlotinib+Cabozantinib	2.49 [0.6 − 11.25]

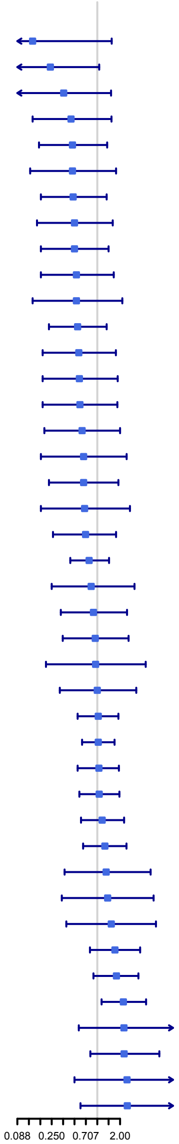

Treatments vs. Atezolizumab	OR[CrI 95%]
Selumetinib	0.13 [0 − 1.41]
Docetaxel+Erlotinib	0.23 [0.05 − 0.95]
Erlotinib+Dalotuzumab	0.34 [0.07 − 1.36]
Placebo	0.43 [0.14 − 1.36]
Pralatrexate	0.44 [0.14 − 1.59]
Erlotinib	0.44 [0.17 − 1.2]
Pemetrexed+Nintedanib	0.45 [0.18 − 1.17]
Pemetrexed+Vandetanib	0.47 [0.18 − 1.24]
Afatinib	0.47 [0.16 − 1.42]
Dacomitinib	0.5 [0.18 − 1.44]
Entinostat+Erlotinib	0.5 [0.14 − 1.91]
Pemetrexed	0.52 [0.23 − 1.15]
Erlotinib+Sunitinib	0.54 [0.19 − 1.56]
Erlotinib+Bevacizumab	0.55 [0.19 − 1.67]
Erlotinib+Tivantinib	0.56 [0.19 − 1.64]
Vandetanib	0.59 [0.2 − 1.79]
Icotinib	0.61 [0.24 − 1.7]
Erlotinib+Pazopanib	0.62 [0.18 − 2.19]
Erlotinib+Onartuzumab	0.64 [0.18 − 2.41]
Amrubicin	0.66 [0.26 − 1.57]
Gefitinib	0.73 [0.47 − 1.21]
Sunitinib	0.79 [0.24 − 2.83]
Pemetrexed+Cetuximab	0.84 [0.33 − 2.15]
Pemetrexed+Erlotinib	0.88 [0.34 − 2.26]
Erlotinib+R1507	0.9 [0.2 − 3.9]
Erlotinib+Figitumumab	0.94 [0.32 − 2.92]
Pembrolizumab	0.94 [0.51 − 1.72]
Docetaxel+Ramucirumab	0.96 [0.58 − 1.61]
Docetaxel	0.97 [0.68 − 1.38]
Nivolumab	0.99 [0.57 − 1.65]
Docetaxel+Nintedanib	1.1 [0.62 − 1.92]
Docetaxel+Vandetanib	1.18 [0.67 − 2.06]
Pemetrexed+Sunitinib	1.23 [0.37 − 4.5]
Pemetrexed+Sorafenib	1.29 [0.34 − 5.02]
Erlotinib+Everolimus	1.43 [0.38 − 5.48]
Docetaxel+Cetuximab	1.6 [0.83 − 3.17]
Docetaxel+Aflibercept	1.68 [0.94 − 3]
Docetaxel+Selumetinib	2.08 [1.19 − 3.8]
Trametinib	2.11 [0.82 − 5.81]
Cabozantinib	2.12 [0.56 − 9.5]
Erlotinib+Cabozantinib	2.32 [0.59 − 10.1]
Cetuximab	2.33 [0.48 − 13.74]

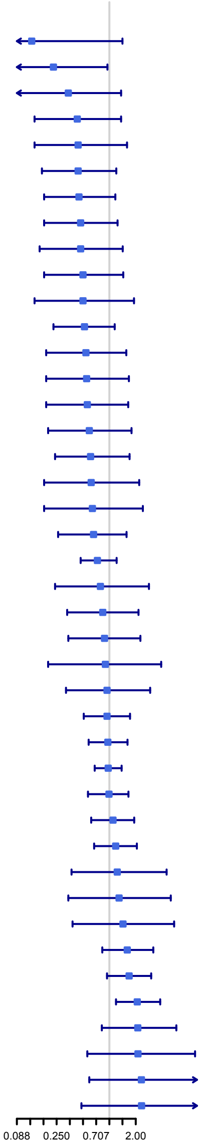

Treatments vs. Docetaxel+Nintedanib	OR[CrI 95%]
Selumetinib	0.12 [0 − 1.32]
Docetaxel+Erlotinib	0.21 [0.05 − 0.88]
Erlotinib+Dalotuzumab	0.31 [0.07 − 1.24]
Placebo	0.38 [0.12 − 1.28]
Pralatrexate	0.4 [0.12 − 1.45]
Erlotinib	0.4 [0.15 − 1.13]
Pemetrexed+Nintedanib	0.41 [0.16 − 1.11]
Pemetrexed+Vandetanib	0.42 [0.16 − 1.17]
Afatinib	0.42 [0.14 − 1.33]
Dacomitinib	0.45 [0.16 − 1.35]
Entinostat+Erlotinib	0.45 [0.12 − 1.78]
Pemetrexed	0.47 [0.21 − 1.1]
Erlotinib+Sunitinib	0.49 [0.17 − 1.46]
Erlotinib+Bevacizumab	0.5 [0.16 − 1.56]
Erlotinib+Tivantinib	0.51 [0.17 − 1.52]
Vandetanib	0.54 [0.18 − 1.69]
Erlotinib+Pazopanib	0.56 [0.16 − 2.03]
Icotinib	0.57 [0.21 − 1.59]
Erlotinib+Onartuzumab	0.59 [0.16 − 2.23]
Amrubicin	0.6 [0.24 − 1.47]
Gefitinib	0.67 [0.4 − 1.17]
Sunitinib	0.71 [0.22 − 2.63]
Pemetrexed+Cetuximab	0.77 [0.3 − 2.03]
Pemetrexed+Erlotinib	0.81 [0.31 − 2.12]
Erlotinib+R1507	0.82 [0.18 − 3.64]
Pembrolizumab	0.86 [0.44 − 1.65]
Erlotinib+Figitumumab	0.86 [0.29 − 2.7]
Docetaxel+Ramucirumab	0.88 [0.49 − 1.56]
Docetaxel	0.88 [0.57 − 1.37]
Nivolumab	0.9 [0.5 − 1.59]
Atezolizumab	0.91 [0.52 − 1.61]
Docetaxel+Vandetanib	1.07 [0.58 − 1.99]
Pemetrexed+Sunitinib	1.12 [0.33 − 4.19]
Pemetrexed+Sorafenib	1.17 [0.3 − 4.63]
Erlotinib+Everolimus	1.31 [0.34 − 5.03]
Docetaxel+Cetuximab	1.47 [0.73 − 2.99]
Docetaxel+Aflibercept	1.53 [0.81 − 2.88]
Docetaxel+Selumetinib	1.89 [1.04 − 3.6]
Trametinib	1.92 [0.71 − 5.5]
Cabozantinib	1.94 [0.5 − 8.87]
Erlotinib+Cabozantinib	2.13 [0.53 − 9.37]
Cetuximab	2.14 [0.42 − 12.88]

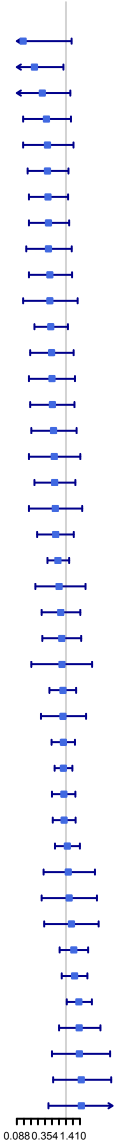

Treatments vs. Docetaxel+Ramucirumab	OR[CrI 95%]
Selumetinib	0.14 [0 − 1.49]
Docetaxel+Erlotinib	0.24 [0.05 − 0.99]
Erlotinib+Dalotuzumab	0.35 [0.08 − 1.41]
Placebo	0.44 [0.14 − 1.43]
Erlotinib	0.46 [0.18 − 1.25]
Pralatrexate	0.46 [0.14 − 1.64]
Pemetrexed+Nintedanib	0.47 [0.19 − 1.21]
Afatinib	0.48 [0.16 − 1.47]
Pemetrexed+Vandetanib	0.48 [0.19 − 1.3]
Dacomitinib	0.52 [0.19 − 1.51]
Entinostat+Erlotinib	0.52 [0.14 − 1.98]
Pemetrexed	0.54 [0.24 − 1.2]
Erlotinib+Sunitinib	0.56 [0.2 − 1.63]
Erlotinib+Bevacizumab	0.57 [0.19 − 1.73]
Erlotinib+Tivantinib	0.58 [0.2 − 1.71]
Vandetanib	0.62 [0.21 − 1.87]
Erlotinib+Pazopanib	0.64 [0.19 − 2.28]
Icotinib	0.64 [0.24 − 1.79]
Erlotinib+Onartuzumab	0.68 [0.19 − 2.51]
Amrubicin	0.69 [0.27 − 1.64]
Gefitinib	0.76 [0.48 − 1.27]
Sunitinib	0.81 [0.25 − 2.84]
Pemetrexed+Cetuximab	0.88 [0.34 − 2.25]
Pemetrexed+Erlotinib	0.92 [0.36 − 2.35]
Erlotinib+R1507	0.93 [0.21 − 4.06]
Pembrolizumab	0.97 [0.53 − 1.81]
Erlotinib+Figitumumab	0.98 [0.33 − 3.05]
Docetaxel	1.01 [0.7 − 1.46]
Nivolumab	1.03 [0.6 − 1.71]
Atezolizumab	1.04 [0.62 − 1.74]
Docetaxel+Nintedanib	1.14 [0.64 − 2.02]
Docetaxel+Vandetanib	1.23 [0.69 − 2.18]
Pemetrexed+Sunitinib	1.28 [0.38 − 4.7]
Pemetrexed+Sorafenib	1.33 [0.34 − 5.21]
Erlotinib+Everolimus	1.49 [0.4 − 5.5]
Docetaxel+Cetuximab	1.67 [0.85 − 3.3]
Docetaxel+Aflibercept	1.74 [0.96 − 3.13]
Docetaxel+Selumetinib	2.16 [1.21 − 3.95]
Trametinib	2.19 [0.84 − 6.18]
Cabozantinib	2.22 [0.57 − 10.02]
Erlotinib+Cabozantinib	2.43 [0.61 − 10.56]
Cetuximab	2.44 [0.5 − 14.59]

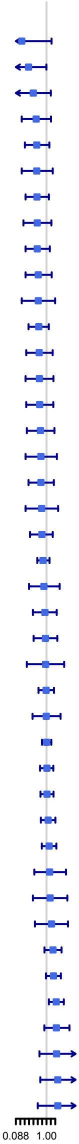

Treatments vs. Afatinib	OR[CrI 95%]
Selumetinib	0.29 [0.01 − 3.01]
Docetaxel+Erlotinib	0.49 [0.16 − 1.53]
Erlotinib+Dalotuzumab	0.72 [0.23 − 2.19]
Placebo	0.91 [0.42 − 2]
Erlotinib	0.95 [0.58 − 1.55]
Pralatrexate	0.95 [0.4 − 2.23]
Pemetrexed+Nintedanib	0.97 [0.38 − 2.56]
Pemetrexed+Vandetanib	1 [0.38 − 2.65]
Dacomitinib	1.06 [0.58 − 1.98]
Entinostat+Erlotinib	1.08 [0.43 − 2.81]
Pemetrexed	1.12 [0.49 − 2.54]
Erlotinib+Sunitinib	1.17 [0.62 − 2.14]
Erlotinib+Bevacizumab	1.18 [0.59 − 2.35]
Erlotinib+Tivantinib	1.18 [0.61 − 2.29]
Vandetanib	1.27 [0.65 − 2.51]
Erlotinib+Pazopanib	1.31 [0.54 − 3.17]
Icotinib	1.33 [0.34 − 5.2]
Erlotinib+Onartuzumab	1.38 [0.56 − 3.55]
Amrubicin	1.4 [0.37 − 5.22]
Gefitinib	1.58 [0.55 − 4.44]
Sunitinib	1.68 [0.5 − 5.83]
Pemetrexed+Cetuximab	1.82 [0.7 − 4.72]
Pemetrexed+Erlotinib	1.91 [0.82 − 4.45]
Erlotinib+R1507	1.92 [0.6 − 6.12]
Pembrolizumab	2.02 [0.63 − 6.36]
Erlotinib+Figitumumab	2.04 [1.02 − 4.1]
Docetaxel+Ramucirumab	2.07 [0.68 − 6.07]
Docetaxel	2.09 [0.72 − 5.7]
Nivolumab	2.11 [0.68 − 6.23]
Atezolizumab	2.14 [0.7 − 6.37]
Docetaxel+Nintedanib	2.35 [0.75 − 7.08]
Docetaxel+Vandetanib	2.53 [0.8 − 7.71]
Pemetrexed+Sunitinib	2.65 [0.77 − 9.47]
Pemetrexed+Sorafenib	2.77 [0.66 − 10.58]
Erlotinib+Everolimus	3.1 [1.13 − 8.24]
Docetaxel+Cetuximab	3.43 [1.06 − 11.34]
Docetaxel+Aflibercept	3.59 [1.13 − 11]
Docetaxel+Selumetinib	4.52 [1.43 − 13.89]
Trametinib	4.62 [1.1 − 18.27]
Cabozantinib	4.65 [1.52 − 15.69]
Erlotinib+Cabozantinib	5.01 [1.64 − 17.25]
Cetuximab	5.06 [0.99 − 25.58]

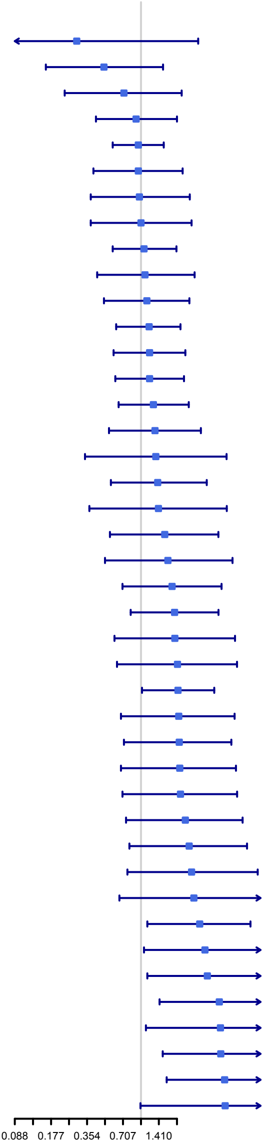

Treatments vs. Cabozantinib	OR[CrI 95%]
Selumetinib	0.06 [0 − 0.71]
Docetaxel+Erlotinib	0.11 [0.02 − 0.43]
Erlotinib+Dalotuzumab	0.15 [0.03 − 0.66]
Placebo	0.2 [0.06 − 0.63]
Pralatrexate	0.2 [0.05 − 0.72]
Erlotinib	0.2 [0.07 − 0.56]
Pemetrexed+Nintedanib	0.21 [0.05 − 0.77]
Afatinib	0.22 [0.06 − 0.66]
Pemetrexed+Vandetanib	0.22 [0.05 − 0.8]
Dacomitinib	0.23 [0.07 − 0.67]
Entinostat+Erlotinib	0.24 [0.06 − 0.85]
Pemetrexed	0.24 [0.06 − 0.79]
Erlotinib+Sunitinib	0.25 [0.08 − 0.72]
Erlotinib+Bevacizumab	0.25 [0.08 − 0.79]
Erlotinib+Tivantinib	0.25 [0.08 − 0.76]
Vandetanib	0.27 [0.08 − 0.83]
Erlotinib+Pazopanib	0.28 [0.07 − 1.03]
Icotinib	0.29 [0.05 − 1.44]
Erlotinib+Onartuzumab	0.3 [0.08 − 1.1]
Amrubicin	0.31 [0.06 − 1.42]
Gefitinib	0.34 [0.08 − 1.27]
Sunitinib	0.37 [0.07 − 1.6]
Pemetrexed+Cetuximab	0.39 [0.09 − 1.41]
Erlotinib+R1507	0.41 [0.09 − 1.85]
Pemetrexed+Erlotinib	0.41 [0.11 − 1.34]
Erlotinib+Figitumumab	0.44 [0.13 − 1.35]
Pembrolizumab	0.45 [0.09 − 1.76]
Docetaxel+Ramucirumab	0.45 [0.1 − 1.75]
Docetaxel	0.45 [0.11 − 1.65]
Nivolumab	0.46 [0.1 − 1.78]
Atezolizumab	0.47 [0.11 − 1.8]
Docetaxel+Nintedanib	0.52 [0.11 − 2.01]
Docetaxel+Vandetanib	0.55 [0.12 − 2.16]
Pemetrexed+Sorafenib	0.59 [0.11 − 2.98]
Pemetrexed+Sunitinib	0.59 [0.11 − 2.57]
Erlotinib+Everolimus	0.66 [0.16 − 2.58]
Docetaxel+Cetuximab	0.75 [0.15 − 3.13]
Docetaxel+Aflibercept	0.79 [0.17 − 3.11]
Docetaxel+Selumetinib	0.97 [0.22 − 3.86]
Trametinib	0.99 [0.18 − 4.86]
Cetuximab	1.06 [0.17 − 6.55]
Erlotinib+Cabozantinib	1.08 [0.37 − 3.16]

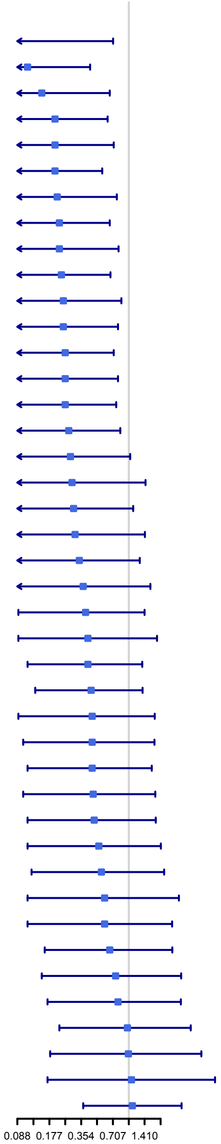

Treatments vs. Erlotinib+Cabozantinib	OR[CrI 95%]
Selumetinib	0.06 [0 − 0.69]
Docetaxel+Erlotinib	0.1 [0.02 − 0.4]
Erlotinib+Dalotuzumab	0.15 [0.03 − 0.58]
Placebo	0.18 [0.05 − 0.58]
Pralatrexate	0.19 [0.05 − 0.66]
Erlotinib	0.19 [0.06 − 0.53]
Pemetrexed+Nintedanib	0.2 [0.05 − 0.7]
Afatinib	0.2 [0.06 − 0.61]
Pemetrexed+Vandetanib	0.2 [0.05 − 0.72]
Dacomitinib	0.21 [0.07 − 0.62]
Entinostat+Erlotinib	0.22 [0.06 − 0.79]
Pemetrexed	0.23 [0.06 − 0.72]
Erlotinib+Sunitinib	0.23 [0.07 − 0.68]
Erlotinib+Bevacizumab	0.24 [0.07 − 0.72]
Erlotinib+Tivantinib	0.24 [0.07 − 0.72]
Vandetanib	0.25 [0.07 − 0.76]
Icotinib	0.26 [0.05 − 1.27]
Erlotinib+Pazopanib	0.26 [0.07 − 0.93]
Amrubicin	0.28 [0.05 − 1.35]
Erlotinib+Onartuzumab	0.28 [0.07 − 0.99]
Gefitinib	0.31 [0.08 − 1.2]
Sunitinib	0.34 [0.07 − 1.53]
Pemetrexed+Cetuximab	0.37 [0.09 − 1.28]
Pemetrexed+Erlotinib	0.38 [0.1 − 1.23]
Erlotinib+R1507	0.39 [0.09 − 1.63]
Pembrolizumab	0.4 [0.09 − 1.66]
Erlotinib+Figitumumab	0.41 [0.12 − 1.25]
Docetaxel+Ramucirumab	0.41 [0.09 − 1.63]
Docetaxel	0.41 [0.1 − 1.57]
Nivolumab	0.42 [0.1 − 1.66]
Atezolizumab	0.43 [0.1 − 1.69]
Docetaxel+Nintedanib	0.47 [0.11 − 1.88]
Docetaxel+Vandetanib	0.5 [0.11 − 2.02]
Pemetrexed+Sunitinib	0.53 [0.11 − 2.36]
Pemetrexed+Sorafenib	0.56 [0.09 − 2.56]
Erlotinib+Everolimus	0.62 [0.14 − 2.32]
Docetaxel+Cetuximab	0.69 [0.15 − 2.91]
Docetaxel+Aflibercept	0.71 [0.16 − 2.84]
Docetaxel+Selumetinib	0.88 [0.2 − 3.56]
Trametinib	0.91 [0.17 − 4.55]
Cabozantinib	0.93 [0.32 − 2.72]
Cetuximab	0.99 [0.16 − 6.15]

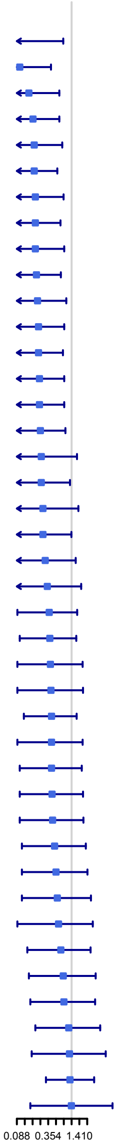

Treatments vs. Placebo	OR[CrI 95%]
Selumetinib	0.32 [0.01 − 3.38]
Docetaxel+Erlotinib	0.54 [0.16 − 1.78]
Erlotinib+Dalotuzumab	0.78 [0.24 − 2.49]
Pralatrexate	1.03 [0.41 − 2.64]
Erlotinib	1.05 [0.56 − 1.91]
Pemetrexed+Nintedanib	1.07 [0.39 − 2.98]
Afatinib	1.1 [0.5 − 2.38]
Pemetrexed+Vandetanib	1.11 [0.39 − 3.12]
Dacomitinib	1.17 [0.72 − 1.9]
Entinostat+Erlotinib	1.2 [0.43 − 3.22]
Pemetrexed	1.23 [0.49 − 2.98]
Erlotinib+Sunitinib	1.28 [0.61 − 2.59]
Erlotinib+Bevacizumab	1.29 [0.59 − 2.81]
Erlotinib+Tivantinib	1.3 [0.61 − 2.74]
Vandetanib	1.39 [0.64 − 3.02]
Erlotinib+Pazopanib	1.43 [0.55 − 3.82]
Icotinib	1.45 [0.36 − 5.77]
Erlotinib+Onartuzumab	1.51 [0.58 − 4.22]
Amrubicin	1.53 [0.39 − 6.16]
Gefitinib	1.74 [0.58 − 5.16]
Sunitinib	1.87 [0.52 − 6.49]
Pemetrexed+Cetuximab	2.01 [0.71 − 5.49]
Pemetrexed+Erlotinib	2.1 [0.81 − 5.13]
Erlotinib+R1507	2.13 [0.63 − 7.3]
Pembrolizumab	2.21 [0.65 − 7.26]
Erlotinib+Figitumumab	2.24 [1 − 4.88]
Docetaxel+Ramucirumab	2.25 [0.7 − 6.95]
Docetaxel	2.28 [0.75 − 6.66]
Nivolumab	2.3 [0.72 − 7.2]
Atezolizumab	2.34 [0.73 − 7.31]
Docetaxel+Nintedanib	2.6 [0.78 − 8.22]
Docetaxel+Vandetanib	2.76 [0.85 − 8.96]
Pemetrexed+Sunitinib	2.92 [0.8 − 10.41]
Pemetrexed+Sorafenib	3.03 [0.68 − 12.19]
Erlotinib+Everolimus	3.39 [1.17 − 9.6]
Docetaxel+Cetuximab	3.74 [1.1 − 12.97]
Docetaxel+Aflibercept	3.91 [1.19 − 12.59]
Docetaxel+Selumetinib	4.89 [1.53 − 16.04]
Trametinib	5 [1.2 − 20.43]
Cabozantinib	5.05 [1.6 − 17.87]
Erlotinib+Cabozantinib	5.47 [1.73 − 19.87]
Cetuximab	5.53 [1.12 − 29.52]

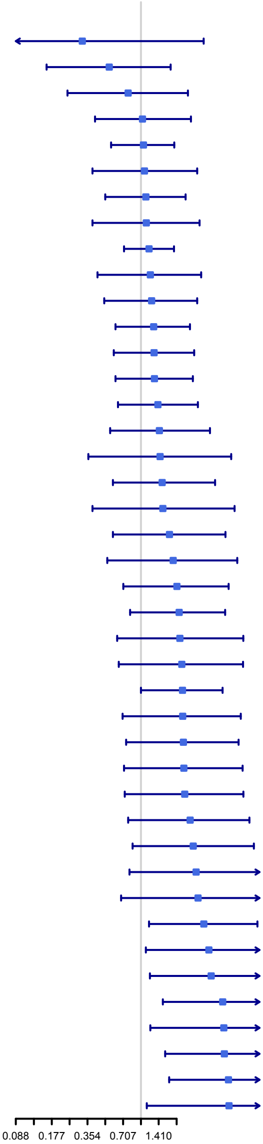

Appendix Figure 2: Cumulative ranking curves and SUCRA values for 4 outcomes

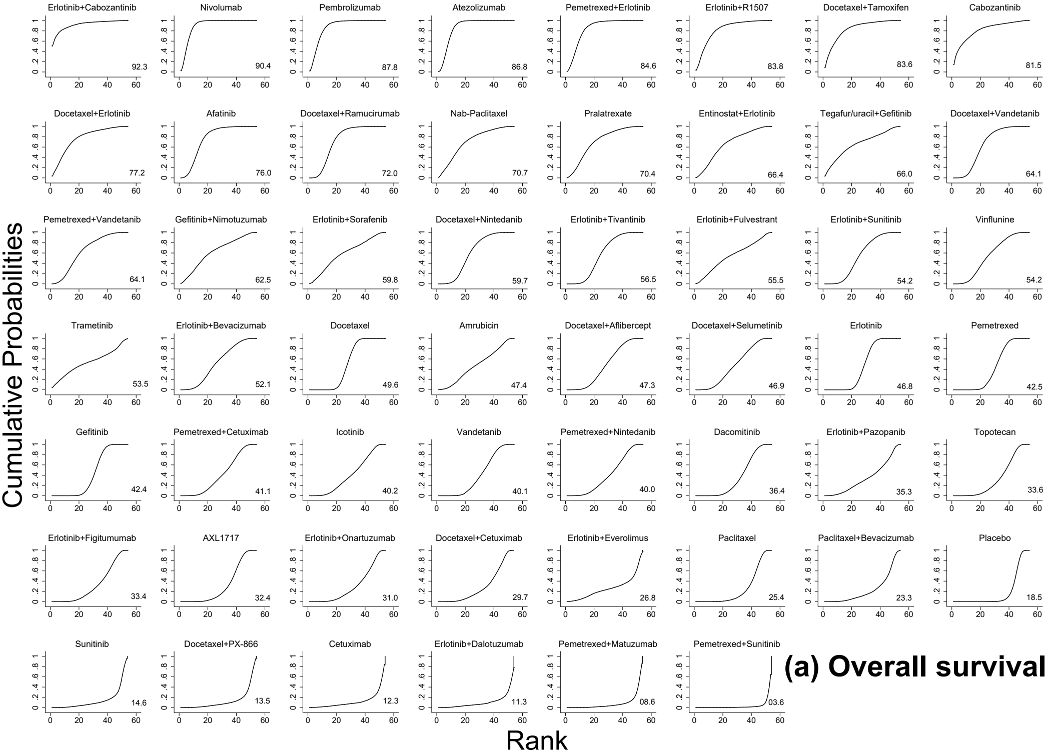
(a) Overall survival
Cumulative Probabilities
Rank
Erlotinib+Cabozantinib 92.3
Nivolumab 90.4
Pembrolizumab 87.8
Atezolizumab 86.8
Pemetrexed+Erlotinib 84.6
Erlotinib+R1507 83.8
Docetaxel+Tamoxifen 83.6
Cabozantinib 81.5
Docetaxel+Erlotinib 77.2
Afatinib 76.0
Docetaxel+Ramucirumab 72.0
Nab-Paclitaxel 70.7
Pralatrexate 70.4
Entinostat+Erlotinib 66.4
Tegafur/uracil+Gefitinib 66.0
Docetaxel+Vandetanib 64.1
Pemetrexed+Vandetanib 64.1
Gefitinib+Nimotuzumab 62.5
Erlotinib+Sorafenib 59.8
Docetaxel+Nintedanib 59.7
Erlotinib+Tivantinib 56.5
Erlotinib+Fulvestrant 55.5
Erlotinib+Sunitinib 54.2
Vinflunine 54.2
Trametinib 53.5
Erlotinib+Bevacizumab 52.1
Docetaxel 49.6
Amrubicin 47.4
Docetaxel+Aflibercept 47.3
Docetaxel+Selumetinib 46.9
Erlotinib 46.8
Pemetrexed 42.5
Gefitinib 42.4
Pemetrexed+Cetuximab 41.1
Icotinib 40.2
Vandetanib 40.1
Pemetrexed+Nintedanib 40.0
Dacomitinib 36.4
Erlotinib+Pazopanib 35.3
Topotecan 33.6
Erlotinib+Figitumumab 33.4
AXL1717 32.4
Erlotinib+Onartuzumab 31.0
Docetaxel+Cetuximab 29.7
Erlotinib+Everolimus 26.8
Paclitaxel 25.4
Paclitaxel+Bevacizumab 23.3
Placebo 18.5
Sunitinib 14.6
Docetaxel+PX-866 13.5
Cetuximab 12.3
Erlotinib+Dalotuzumab 11.3
Pemetrexed+Matuzumab 08.6
Pemetrexed+Sunitinib 03.6

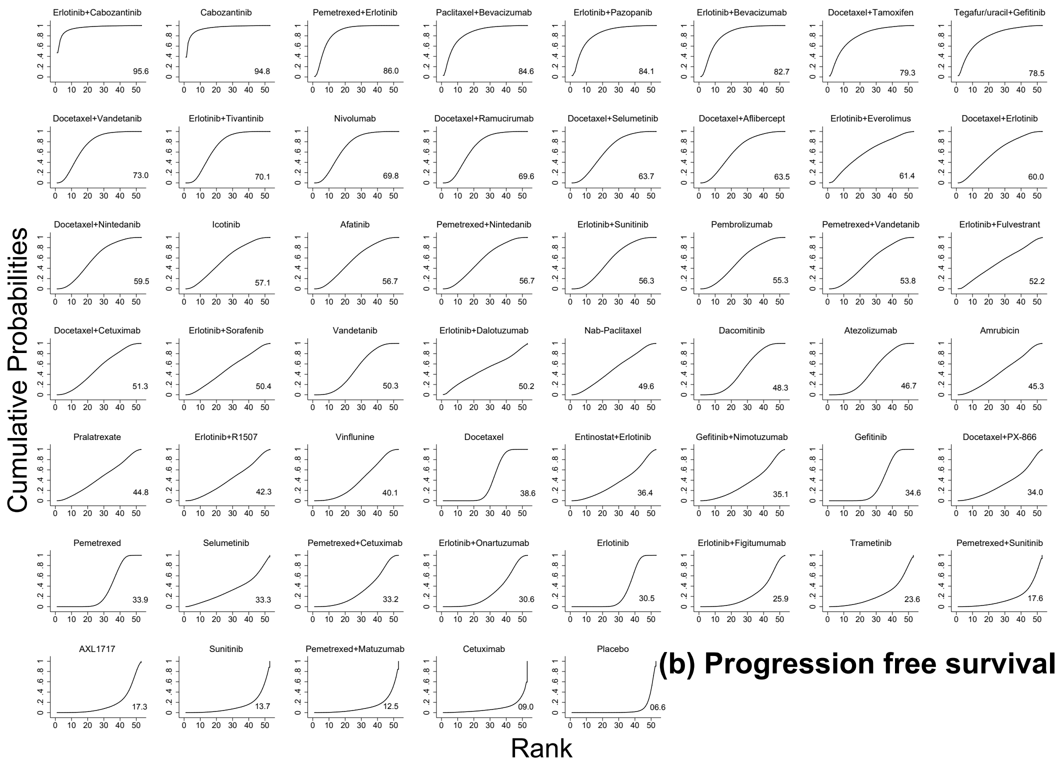
Cumulative Probabilities
Rank
(b) Progression free survival
Erlotinib+Cabozantinib 95.6
Cabozantinib 94.8
Pemetrexed+Erlotinib 86.0
Paclitaxel+Bevacizumab 84.6
Erlotinib+Pazopanib 84.1
Erlotinib+Bevacizumab 82.7
Docetaxel+Tamoxifen 79.3
Tegafur/uracil+Gefitinib 78.5
Docetaxel+Vandetanib 73.0
Erlotinib+Tivantinib 70.1
Nivolumab 69.8
Docetaxel+Ramucirumab 69.6
Docetaxel+Selumetinib 63.7
Docetaxel+Aflibercept 63.5
Erlotinib+Everolimus 61.4
Docetaxel+Erlotinib 60.0
Docetaxel+Nintedanib 59.5
Icotinib 57.1
Afatinib 56.7
Pemetrexed+Nintedanib 56.7
Erlotinib+Sunitinib 56.3
Pembrolizumab 55.3
Pemetrexed+Vandetanib 53.8
Erlotinib+Fulvestrant 52.2
Docetaxel+Cetuximab 51.3
Erlotinib+Sorafenib 50.4
Vandetanib 50.3
Erlotinib+Dalotuzumab 50.2
Nab-Paclitaxel 49.6
Dacomitinib 48.3
Atezolizumab 46.7
Amrubicin 45.3
Pralatrexate 44.8
Erlotinib+R1507 42.3
Vinflunine 40.1
Docetaxel 38.6
Entinostat+Erlotinib 36.4
Gefitinib+Nimotuzumab 35.1
Gefitinib 34.6
Docetaxel+PX-866 34.0
Pemetrexed 33.9
Selumetinib 33.3
Pemetrexed+Cetuximab 33.2
Erlotinib+Onartuzumab 30.6
Erlotinib 30.5
Erlotinib+Figitumumab 25.9
Trametinib 23.6
Pemetrexed+Sunitinib 17.6
AXL1717 17.3
Sunitinib 13.7
Pemetrexed+Matuzumab 12.5
Cetuximab 09.0
Placebo 06.6

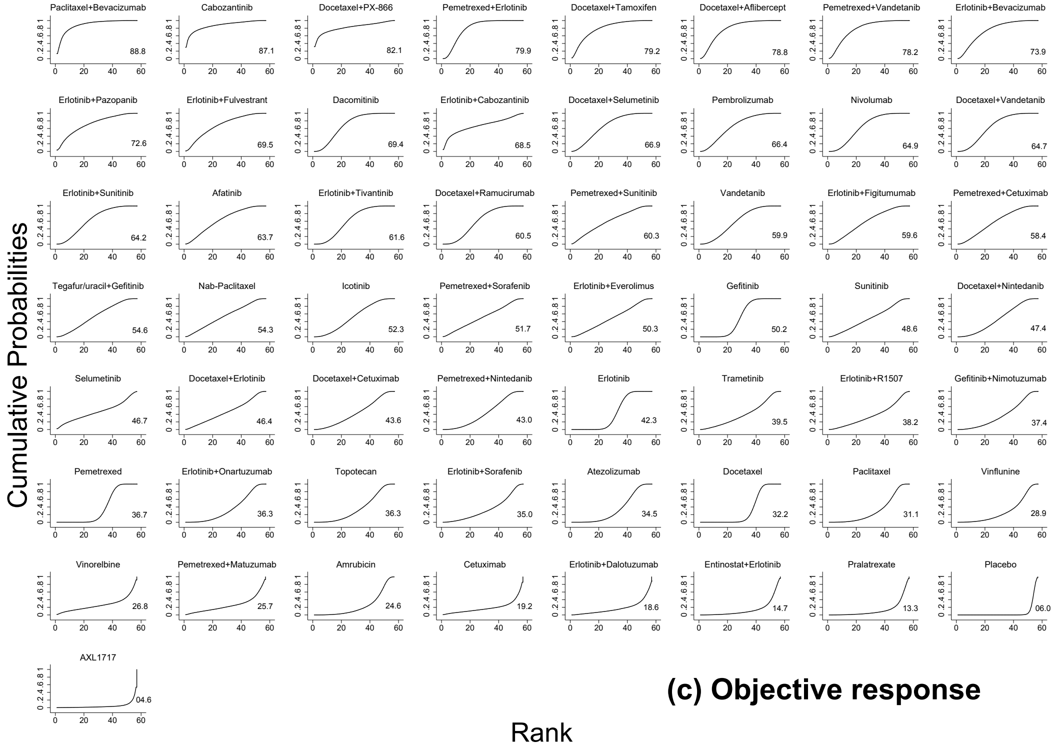

(c) Objective response

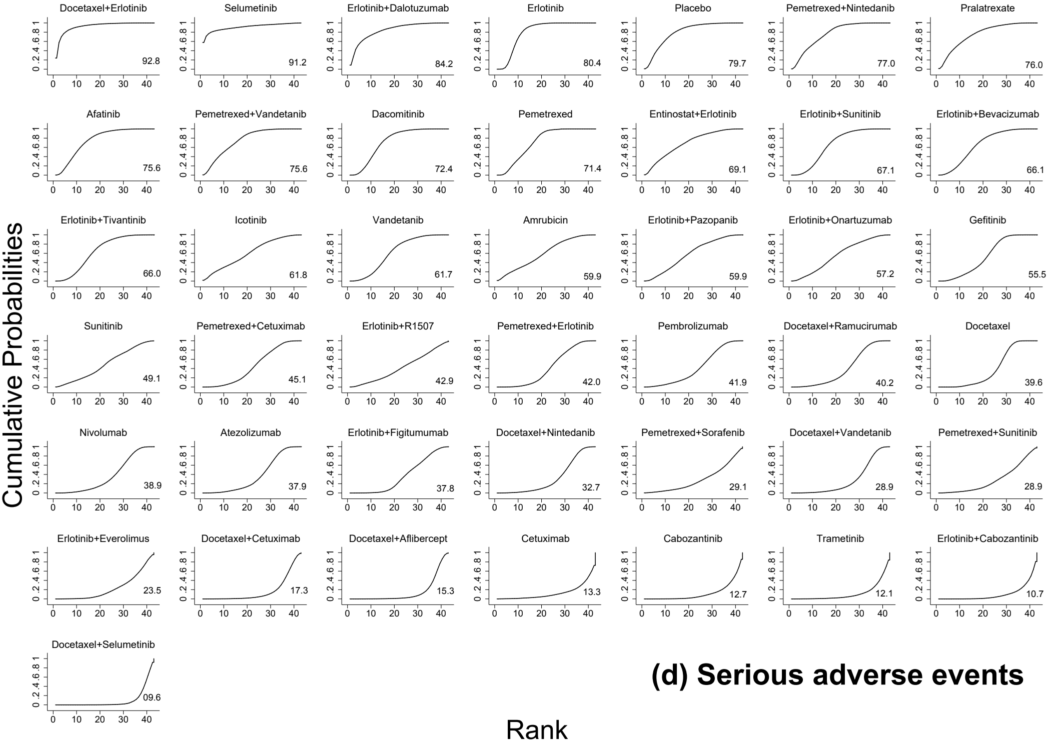

(d) Serious adverse events

Appendix Figure 3: Rankograms for 4 outcomes

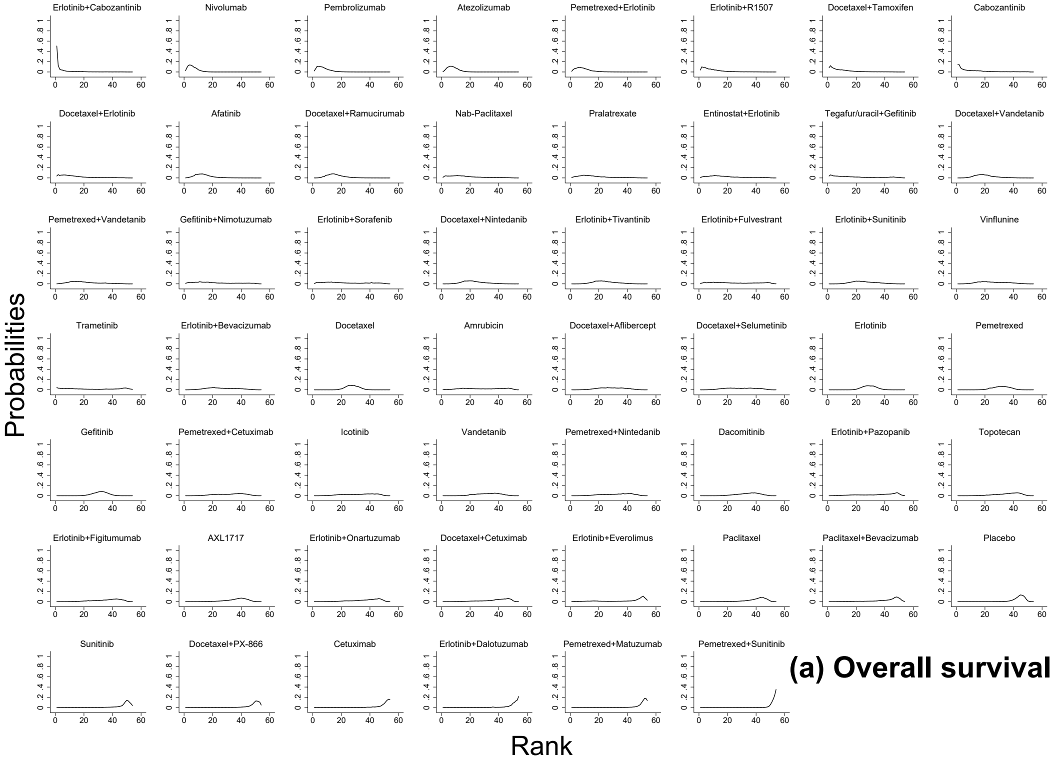
Erlotinib+Cabozantinib
Nivolumab
Pembrolizumab
Atezolizumab
Pemetrexed+Erlotinib
Erlotinib+R1507
Docetaxel+Tamoxifen
Cabozantinib
Docetaxel+Erlotinib
Afatinib
Docetaxel+Ramucirumab
Nab-Paclitaxel
Pralatrexate
Entinostat+Erlotinib
Tegafur/uracil+Gefitinib
Docetaxel+Vandetanib
Pemetrexed+Vandetanib
Gefitinib+Nimotuzumab
Erlotinib+Sorafenib
Docetaxel+Nintedanib
Erlotinib+Tivantinib
Erlotinib+Fulvestrant
Erlotinib+Sunitinib
Vinflunine
Trametinib
Erlotinib+Bevacizumab
Docetaxel
Amrubicin
Docetaxel+Aflibercept
Docetaxel+Selumetinib
Erlotinib
Pemetrexed
Gefitinib
Pemetrexed+Cetuximab
Icotinib
Vandetanib
Pemetrexed+Nintedanib
Dacomitinib
Erlotinib+Pazopanib
Topotecan
Erlotinib+Figitumumab
AXL1717
Erlotinib+Onartuzumab
Docetaxel+Cetuximab
Erlotinib+Everolimus
Paclitaxel
Paclitaxel+Bevacizumab
Placebo
Sunitinib
Docetaxel+PX-866
Cetuximab
Erlotinib+Dalotuzumab
Pemetrexed+Matuzumab
Pemetrexed+Sunitinib
Probabilities
Rank
(a) Overall survival

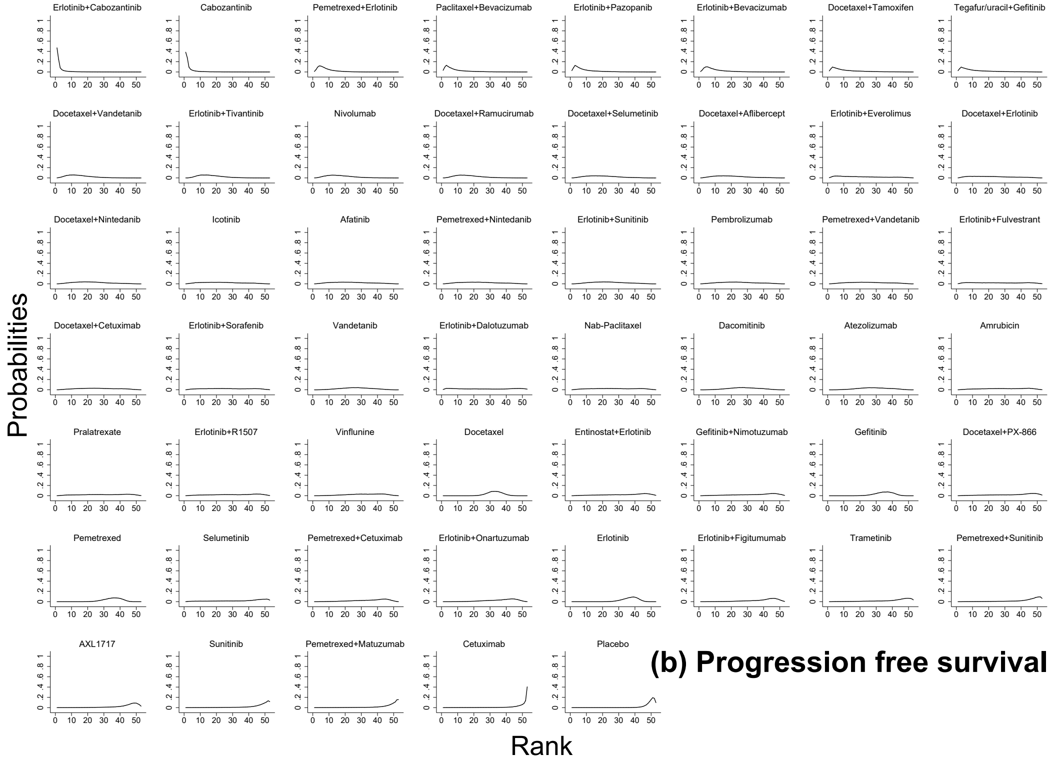

(b) Progression free survival

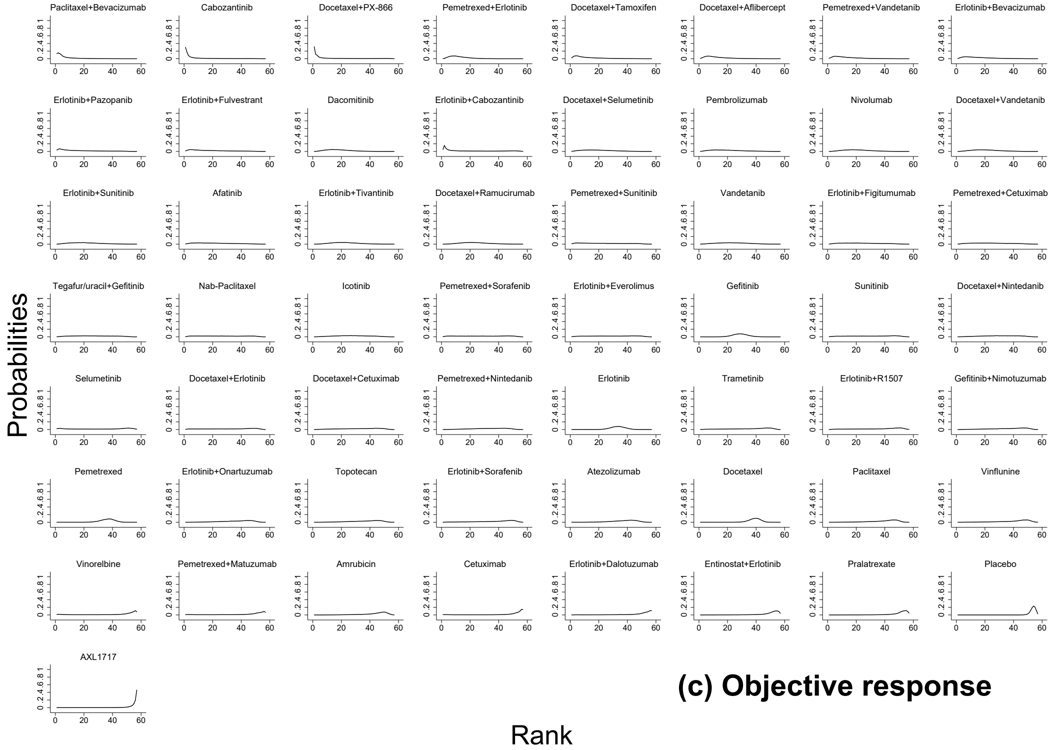

(c) Objective response

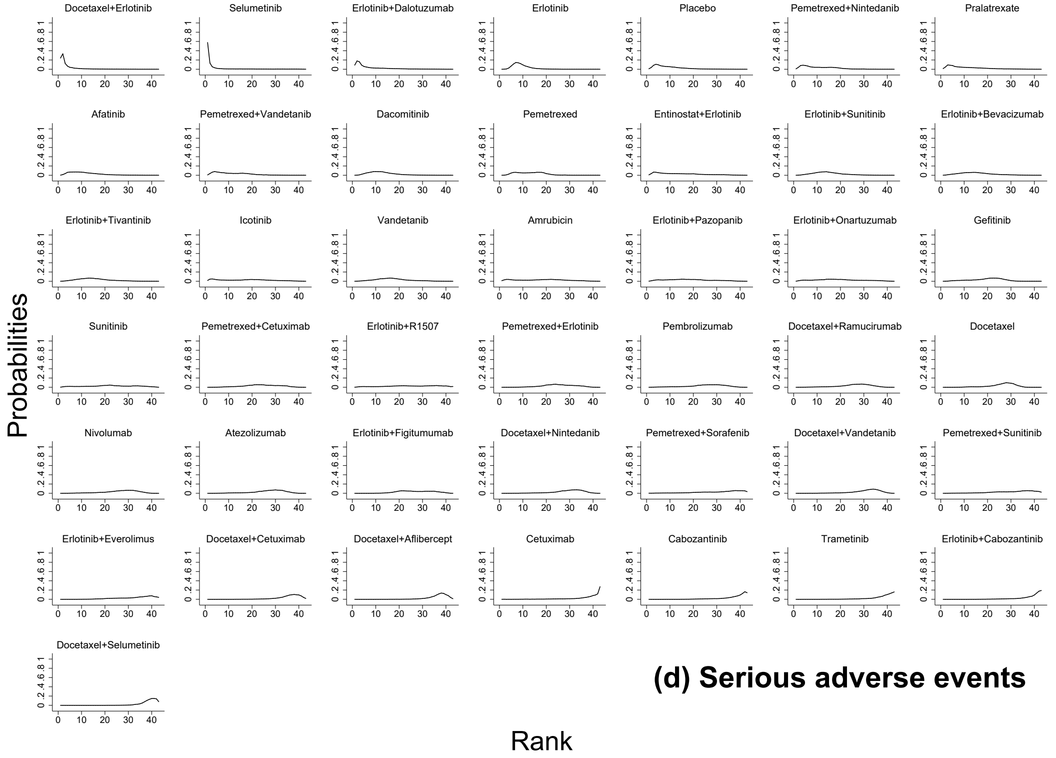

(d) Serious adverse events

Appendix Figure 4: Evaluation of inconsistency

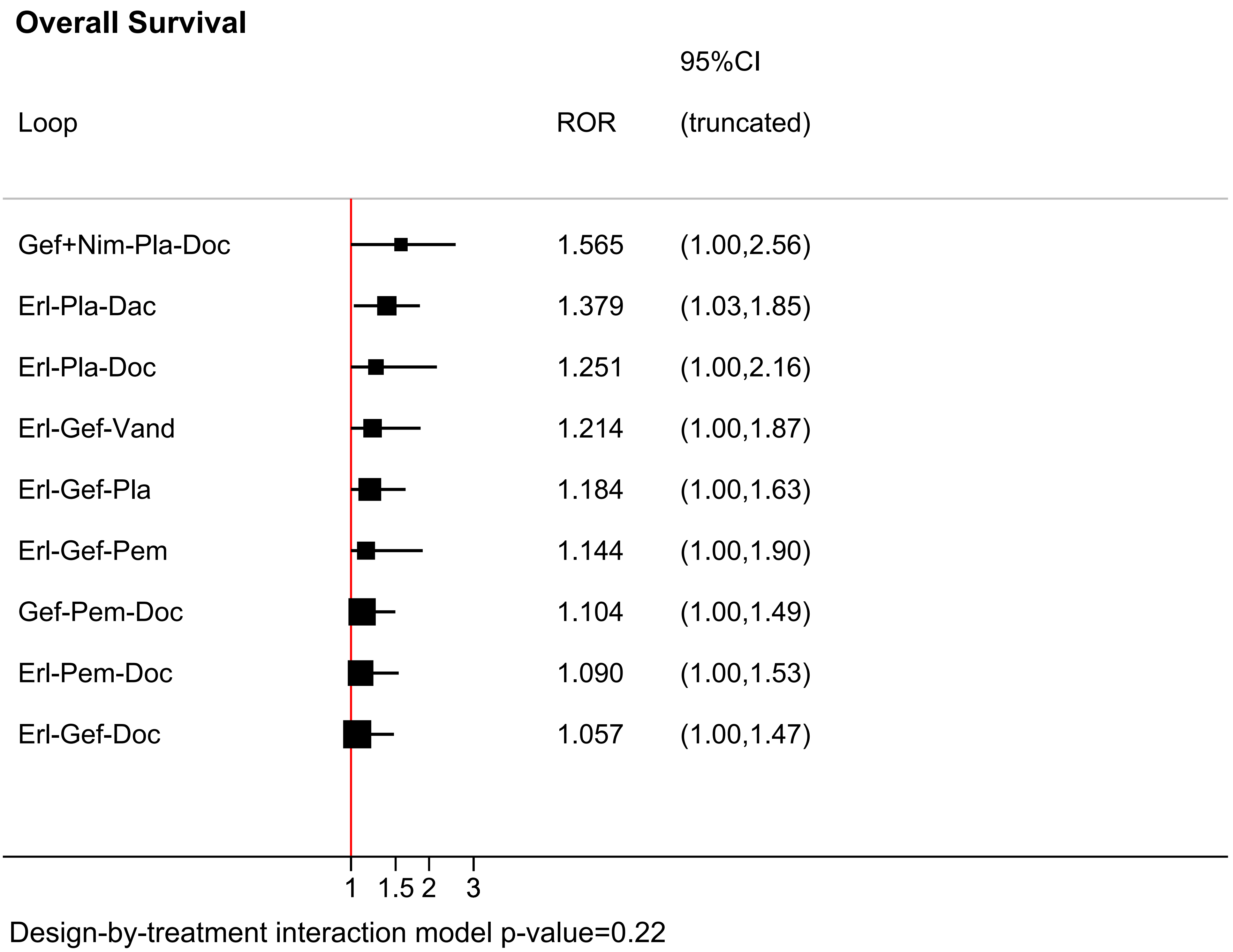
Overall Survival
Loop
ROR
95%CI
(truncated)
Gef+Nim-Pla-Doc
1.565
(1.00,2.56)
Erl-Pla-Dac
1.379
(1.03,1.85)
Erl-Pla-Doc
1.251
(1.00,2.16)
Erl-Gef-Vand
1.214
(1.00,1.87)
Erl-Gef-Pla
1.184
(1.00,1.63)
Erl-Gef-Pem
1.144
(1.00,1.90)
Gef-Pem-Doc
1.104
(1.00,1.49)
Erl-Pem-Doc
1.090
(1.00,1.53)
Erl-Gef-Doc
1.057
(1.00,1.47)
1
1.5
2
3
Design-by-treatment interaction model p-value=0.22

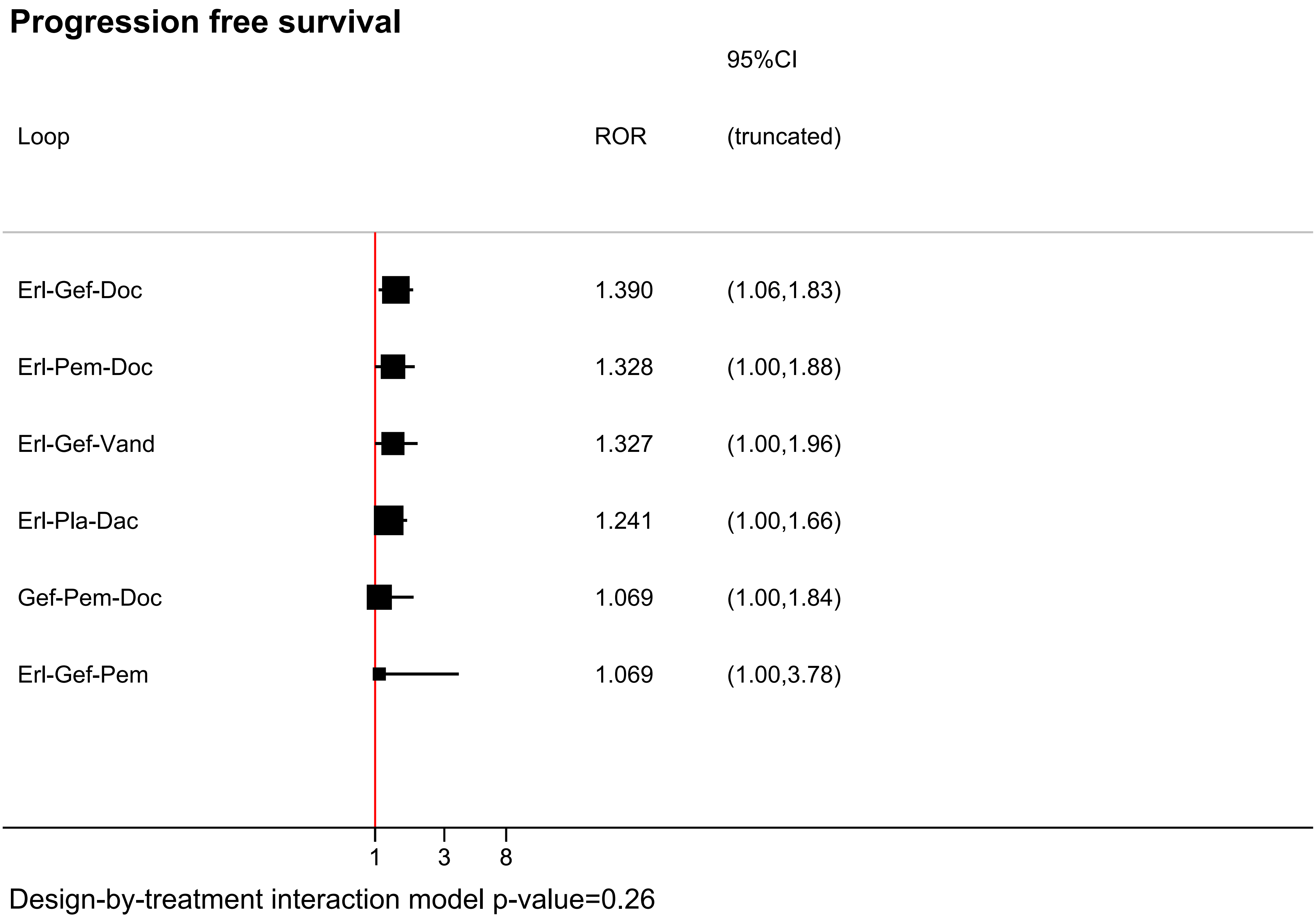
Progression free survival
Loop
ROR
95%CI (truncated)
Erl-Gef-Doc 1.390 (1.06,1.83)
Erl-Pem-Doc 1.328 (1.00,1.88)
Erl-Gef-Vand 1.327 (1.00,1.96)
Erl-Pla-Dac 1.241 (1.00,1.66)
Gef-Pem-Doc 1.069 (1.00,1.84)
Erl-Gef-Pem 1.069 (1.00,3.78)
1
3
8
Design-by-treatment interaction model p-value=0.26

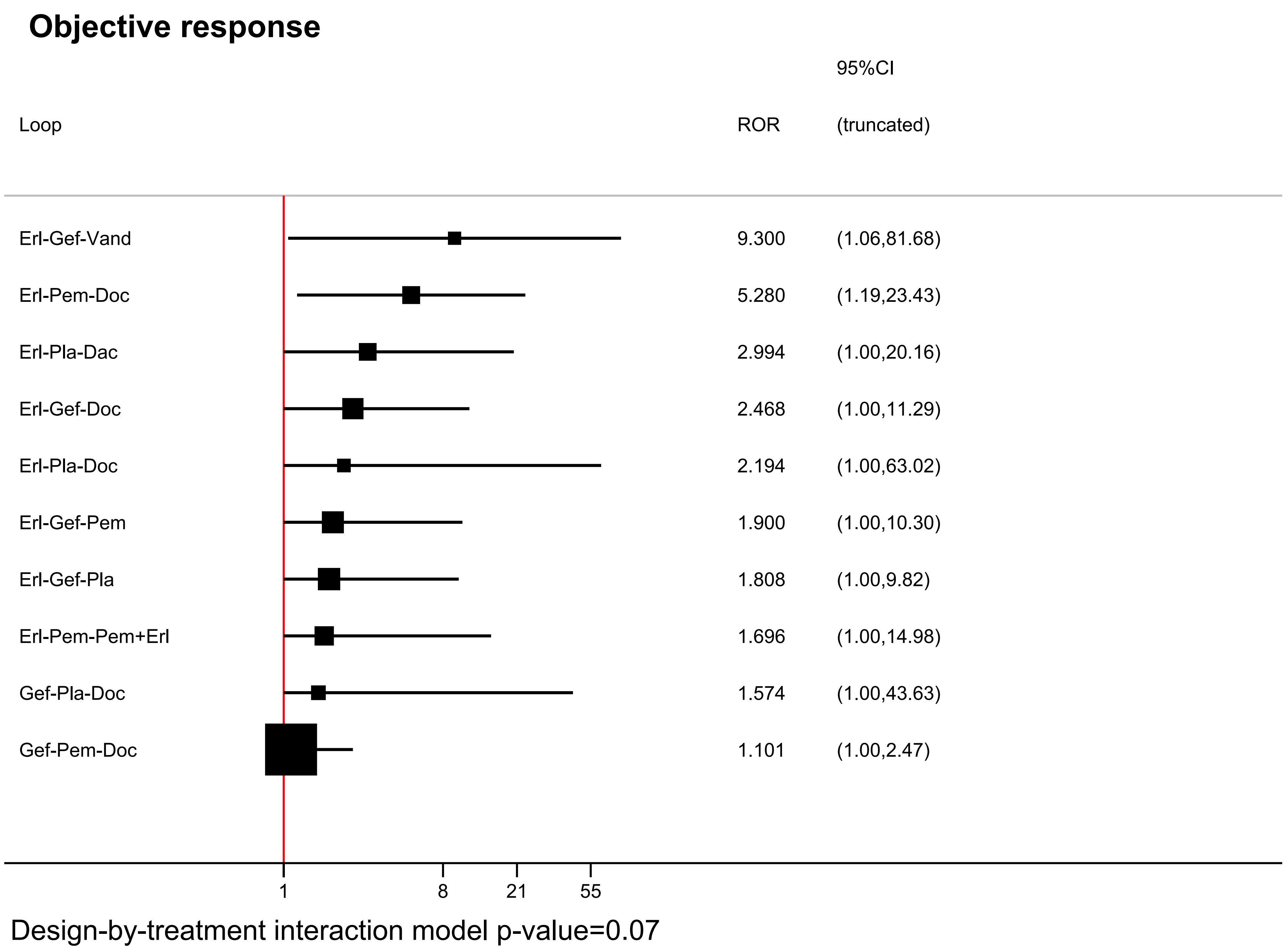
Objective response
Loop
ROR
95%CI
(truncated)
Erl-Gef-Vand 9.300 (1.06,81.68)
Erl-Pem-Doc 5.280 (1.19,23.43)
Erl-Pla-Dac 2.994 (1.00,20.16)
Erl-Gef-Doc 2.468 (1.00,11.29)
Erl-Pla-Doc 2.194 (1.00,63.02)
Erl-Gef-Pem 1.900 (1.00,10.30)
Erl-Gef-Pla 1.808 (1.00,9.82)
Erl-Pem-Pem+Erl 1.696 (1.00,14.98)
Gef-Pla-Doc 1.574 (1.00,43.63)
Gef-Pem-Doc 1.101 (1.00,2.47)
1
8
21
55
Design-by-treatment interaction model p-value=0.07

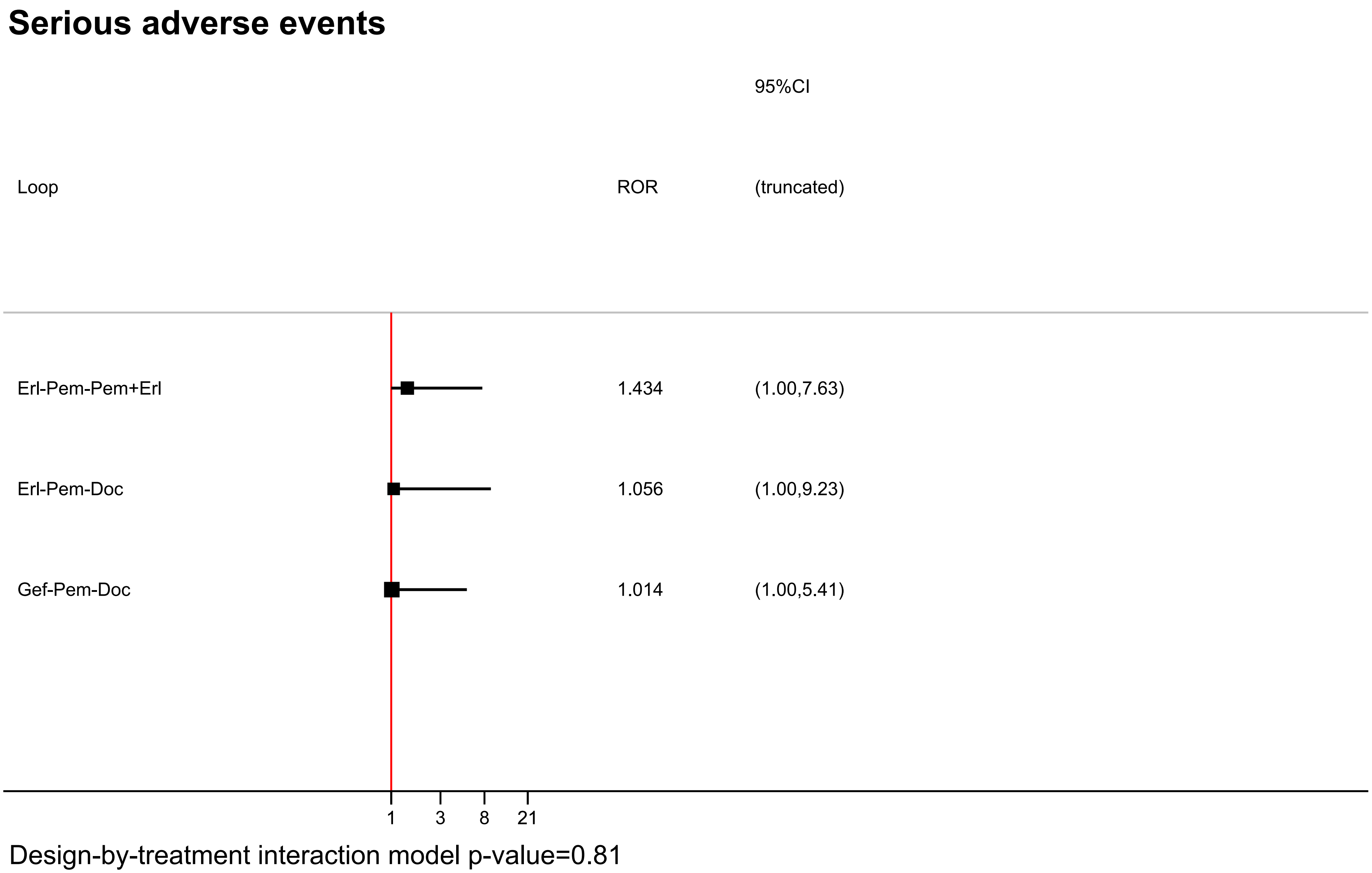
Serious adverse events
95%CI
Loop
ROR
(truncated)
Erl-Pem-Pem+Erl
1.434
(1.00,7.63)
Erl-Pem-Doc
1.056
(1.00,9.23)
Gef-Pem-Doc
1.014
(1.00,5.41)
1
3
8
21
Design-by-treatment interaction model p-value=0.81

Appendix Figures 5: Subgroup analyses by histology and ethnicity: network graphs of trials for OS, forest plots and SUCRA values for OS and PFS

For each network, we have included both:

- trials performed in a specific subtype of population (i.e., including only asian population, only squamous cell carcinoma, or only nonsquamous)
- trials providing results from subgroup analyses either for asian, squamous or nonsquamous population.

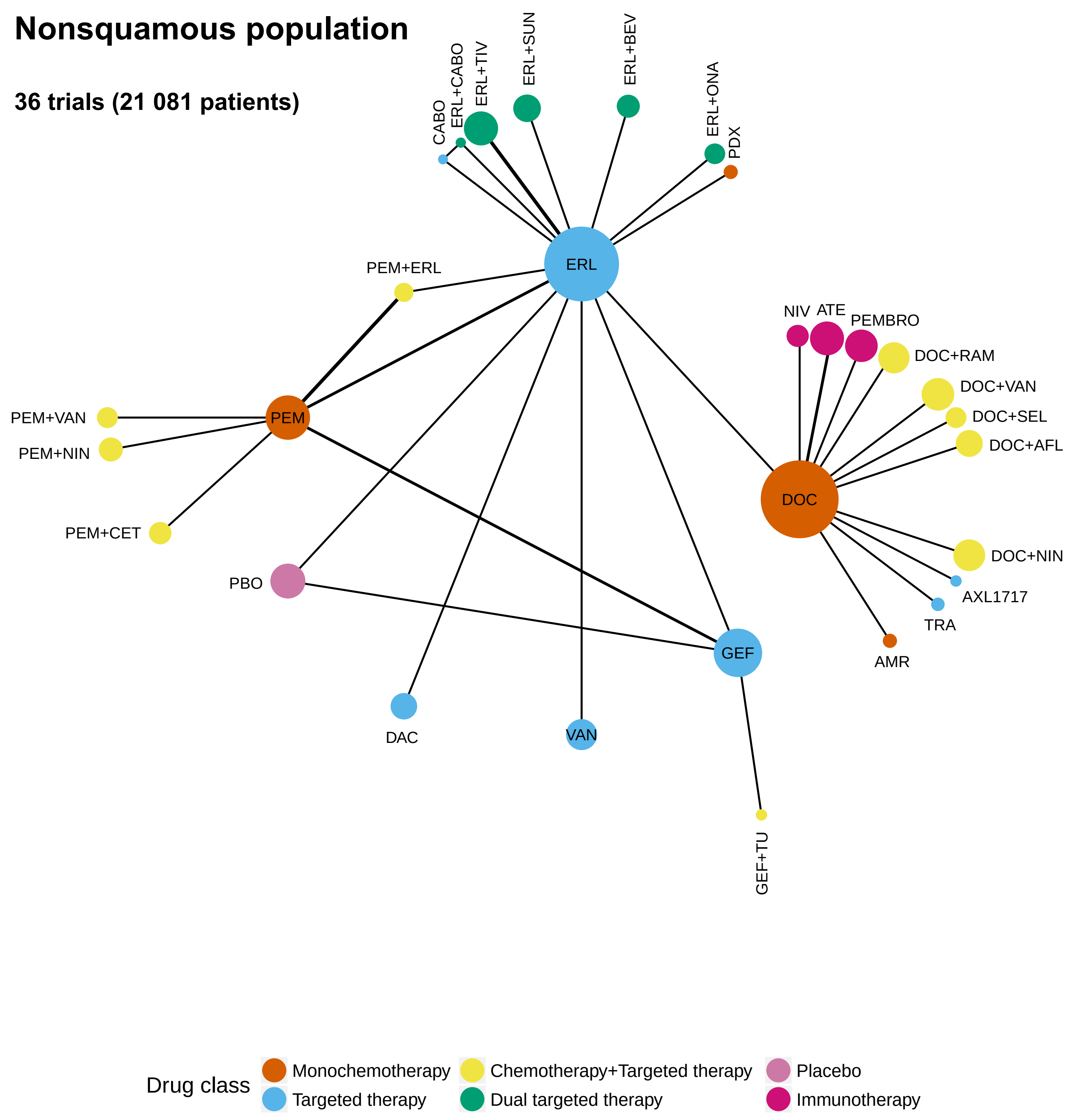

Nonsquamous population
36 trials (21 081 patients)
CABO
ERL+CABO
ERL+TIV
ERL+SUN
ERL+BEV
ERL+ONA
PDX
ERL
PEM+ERL
NIV
ATE
PEMBRO
DOC+RAM
DOC+VAN
DOC+SEL
DOC+AFL
PEM+VAN
PEM
PEM+NIN
DOC
PEM+CET
DOC+NIN
PBO
AXL1717
TRA
AMR
GEF
DAC
VAN
GEF+TU
Drug class
Monochemotherapy
Targeted therapy
Chemotherapy+Targeted therapy
Dual targeted therapy
Placebo
Immunotherapy

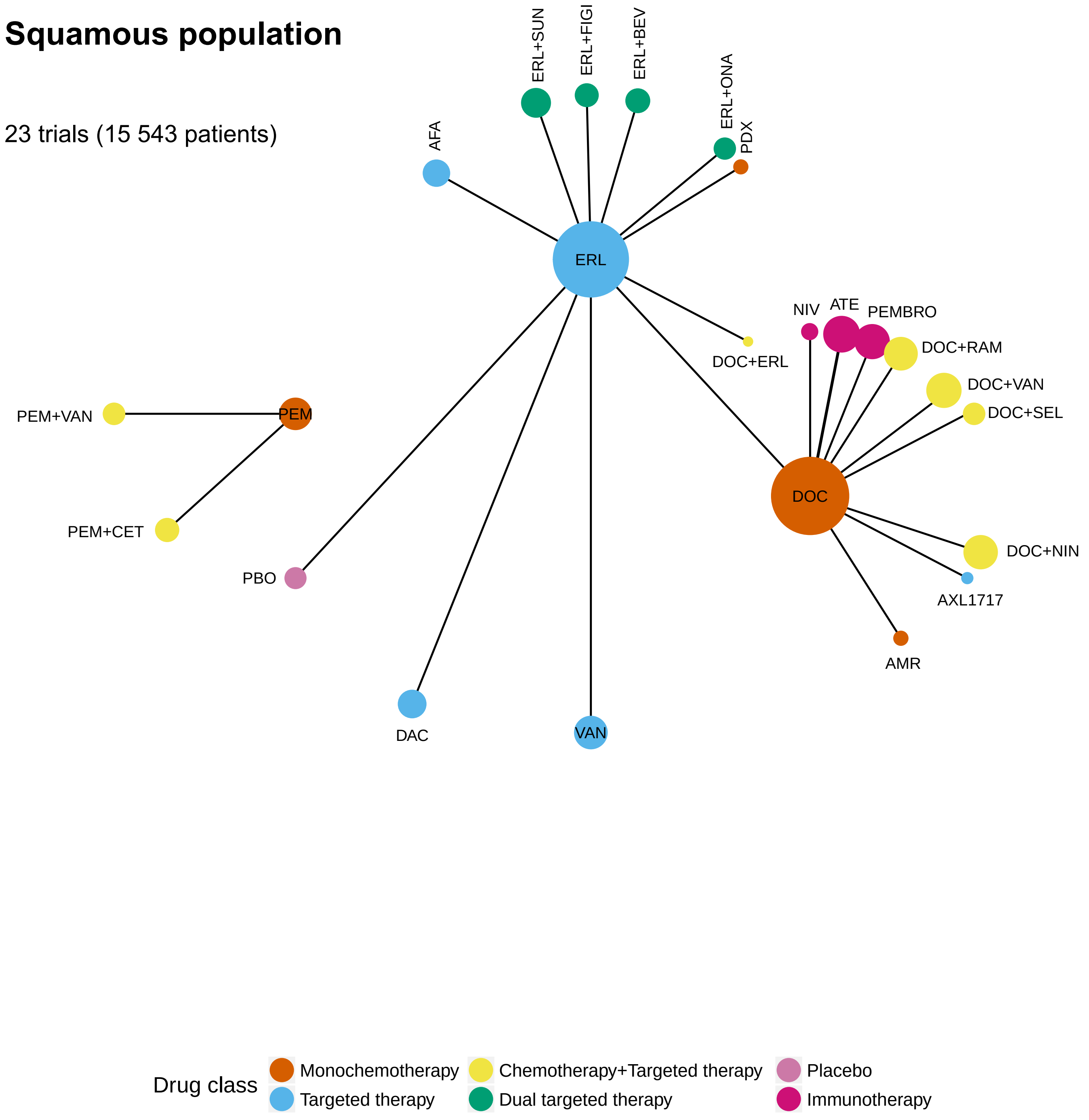
Squamous population
23 trials (15 543 patients)
ERL+SUN
ERL+FIGI
ERL+BEV
ERL+ONA
PDX
AFA
ERL
NIV
ATE
PEMBRO
DOC+RAM
DOC+ERL
DOC+VAN
DOC+SEL
PEM+VAN
PEM
PEM+CET
DOC
DOC+NIN
PBO
AXL1717
AMR
DAC
VAN
Drug class
Monochemotherapy
Targeted therapy
Chemotherapy+Targeted therapy
Dual targeted therapy
Placebo
Immunotherapy

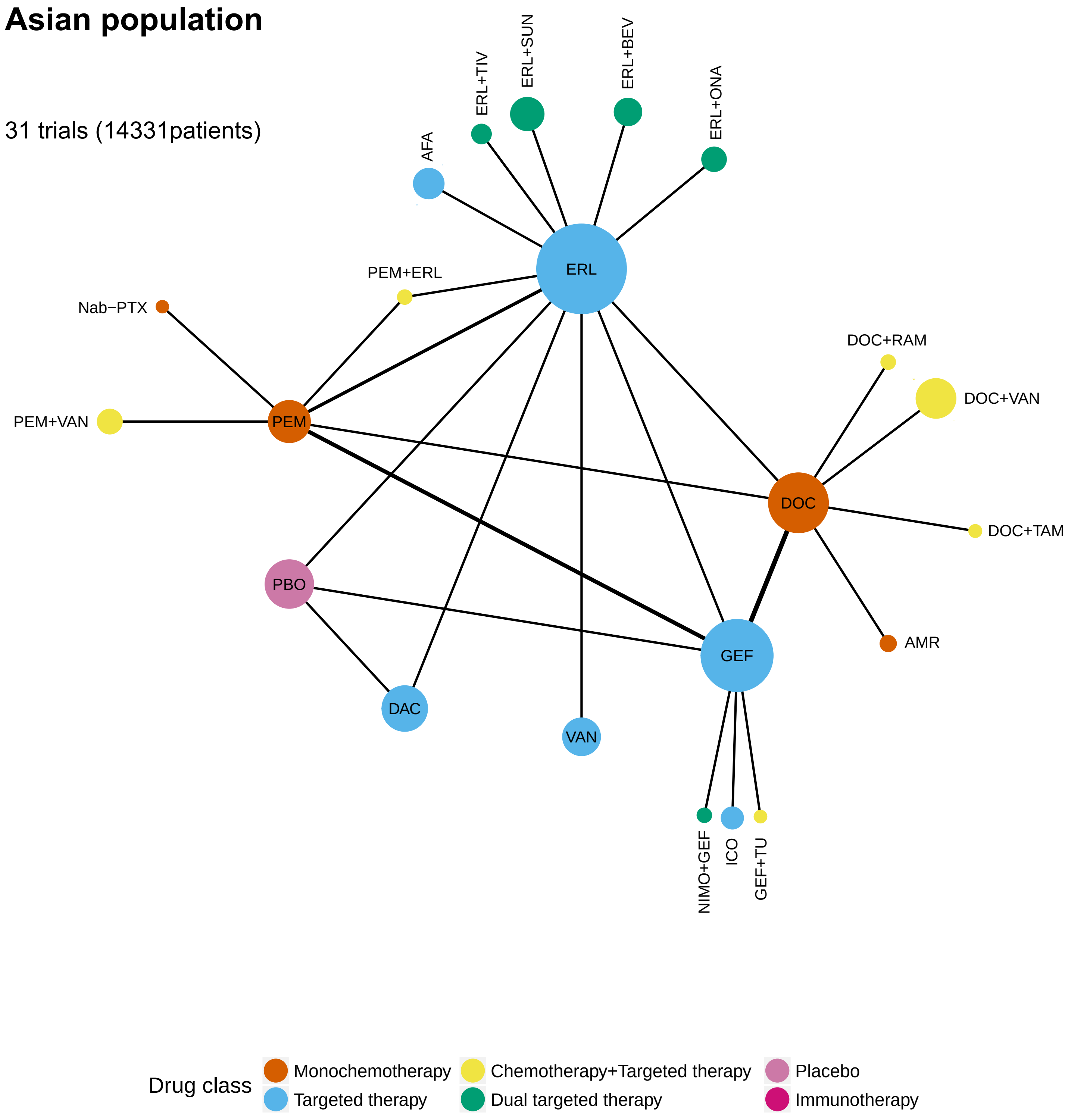
Asian population
31 trials (14331patients)
ERL
ERL+TIV
ERL+SUN
ERL+BEV
ERL+ONA
AFA
PEM+ERL
Nab−PTX
PEM+VAN
PEM
DOC+RAM
DOC+VAN
DOC
DOC+TAM
PBO
AMR
GEF
DAC
VAN
NIMO+GEF
ICO
GEF+TU
Drug class
Monochemotherapy
Targeted therapy
Chemotherapy+Targeted therapy
Dual targeted therapy
Placebo
Immunotherapy

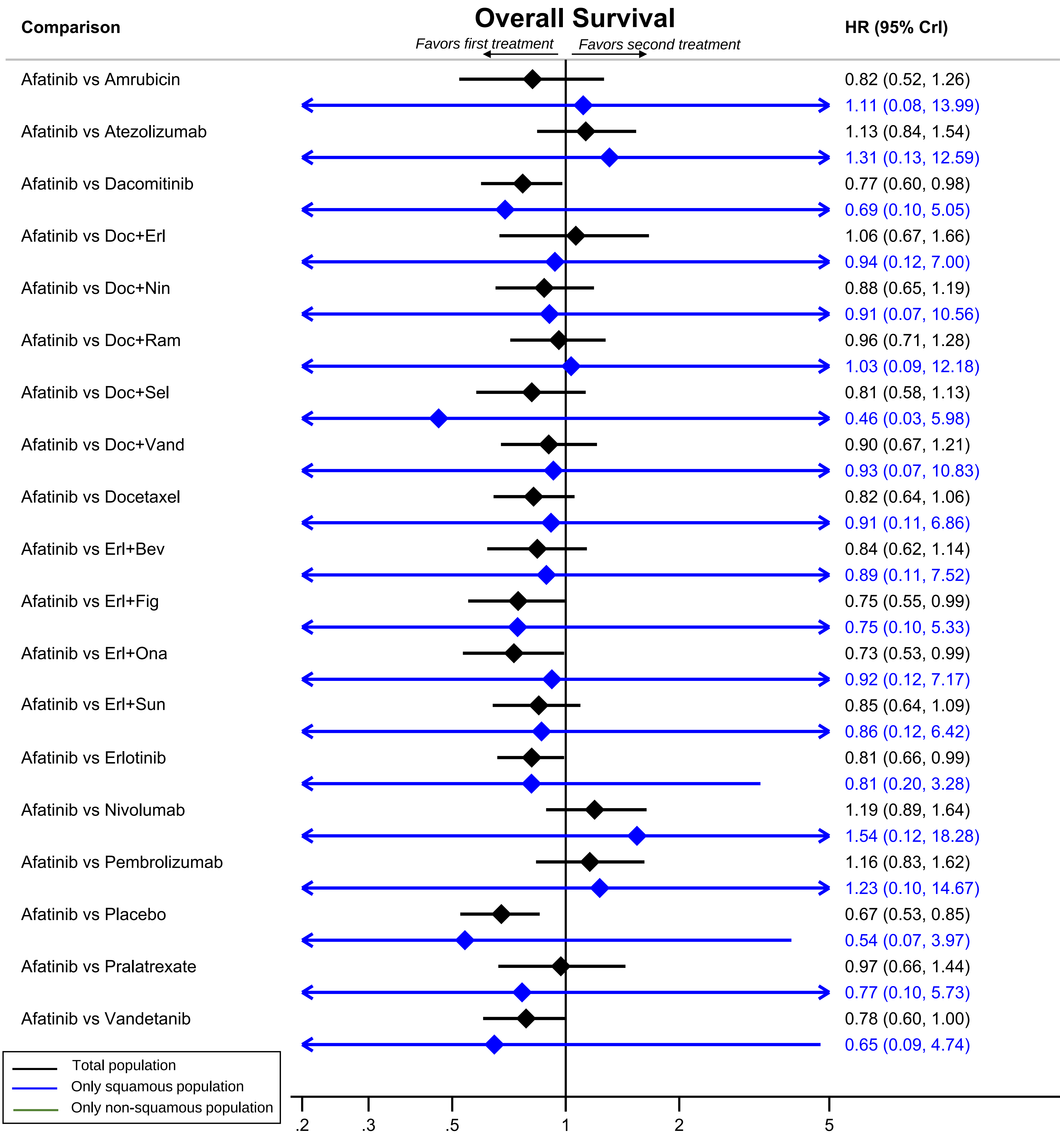
Overall Survival
Comparison
HR (95% CrI)
Favors first treatment
Favors second treatment
Afatinib vs Amrubicin
0.82 (0.52, 1.26)
1.11 (0.08, 13.99)
Afatinib vs Atezolizumab
1.13 (0.84, 1.54)
1.31 (0.13, 12.59)
Afatinib vs Dacomitinib
0.77 (0.60, 0.98)
0.69 (0.10, 5.05)
Afatinib vs Doc+Erl
1.06 (0.67, 1.66)
0.94 (0.12, 7.00)
Afatinib vs Doc+Nin
0.88 (0.65, 1.19)
0.91 (0.07, 10.56)
Afatinib vs Doc+Ram
0.96 (0.71, 1.28)
1.03 (0.09, 12.18)
Afatinib vs Doc+Sel
0.81 (0.58, 1.13)
0.46 (0.03, 5.98)
Afatinib vs Doc+Vand
0.90 (0.67, 1.21)
0.93 (0.07, 10.83)
Afatinib vs Docetaxel
0.82 (0.64, 1.06)
0.91 (0.11, 6.86)
Afatinib vs Erl+Bev
0.84 (0.62, 1.14)
0.89 (0.11, 7.52)
Afatinib vs Erl+Fig
0.75 (0.55, 0.99)
0.75 (0.10, 5.33)
Afatinib vs Erl+Ona
0.73 (0.53, 0.99)
0.92 (0.12, 7.17)
Afatinib vs Erl+Sun
0.85 (0.64, 1.09)
0.86 (0.12, 6.42)
Afatinib vs Erlotinib
0.81 (0.66, 0.99)
0.81 (0.20, 3.28)
Afatinib vs Nivolumab
1.19 (0.89, 1.64)
1.54 (0.12, 18.28)
Afatinib vs Pembrolizumab
1.16 (0.83, 1.62)
1.23 (0.10, 14.67)
Afatinib vs Placebo
0.67 (0.53, 0.85)
0.54 (0.07, 3.97)
Afatinib vs Pralatrexate
0.97 (0.66, 1.44)
0.77 (0.10, 5.73)
Afatinib vs Vandetanib
0.78 (0.60, 1.00)
0.65 (0.09, 4.74)
Total population
Only squamous population
Only non-squamous population
.2
.3
.5
1
2
5

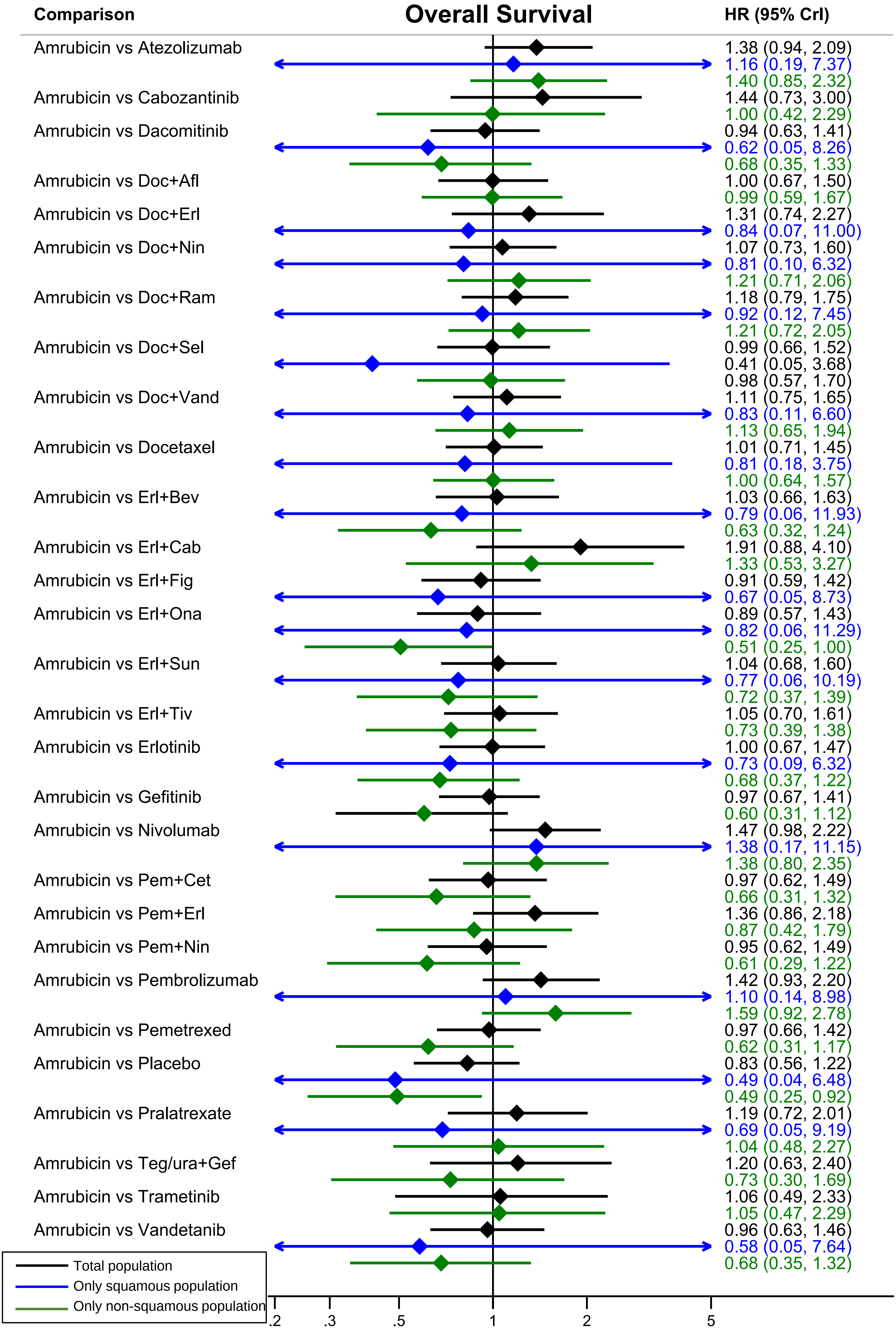
Comparison
Overall Survival
HR (95% CrI)
Amrubicin vs Atezolizumab
1.38 (0.94, 2.09)
1.16 (0.19, 7.37)
1.40 (0.85, 2.32)
Amrubicin vs Cabozantinib
1.44 (0.73, 3.00)
1.00 (0.42, 2.29)
Amrubicin vs Dacomitinib
0.94 (0.63, 1.41)
0.62 (0.05, 8.26)
0.68 (0.35, 1.33)
Amrubicin vs Doc+Afl
1.00 (0.67, 1.50)
0.99 (0.59, 1.67)
Amrubicin vs Doc+Erl
1.31 (0.74, 2.27)
0.84 (0.07, 11.00)
Amrubicin vs Doc+Nin
1.07 (0.73, 1.60)
0.81 (0.10, 6.32)
1.21 (0.71, 2.06)
Amrubicin vs Doc+Ram
1.18 (0.79, 1.75)
0.92 (0.12, 7.45)
1.21 (0.72, 2.05)
Amrubicin vs Doc+Sel
0.99 (0.66, 1.52)
0.41 (0.05, 3.68)
0.98 (0.57, 1.70)
Amrubicin vs Doc+Vand
1.11 (0.75, 1.65)
0.83 (0.11, 6.60)
1.13 (0.65, 1.94)
Amrubicin vs Docetaxel
1.01 (0.71, 1.45)
0.81 (0.18, 3.75)
1.00 (0.64, 1.57)
Amrubicin vs Erl+Bev
1.03 (0.66, 1.63)
0.79 (0.06, 11.93)
0.63 (0.32, 1.24)
Amrubicin vs Erl+Cab
1.91 (0.88, 4.10)
1.33 (0.53, 3.27)
Amrubicin vs Erl+Fig
0.91 (0.59, 1.42)
0.67 (0.05, 8.73)
Amrubicin vs Erl+Ona
0.89 (0.57, 1.43)
0.82 (0.06, 11.29)
0.51 (0.25, 1.00)
Amrubicin vs Erl+Sun
1.04 (0.68, 1.60)
0.77 (0.06, 10.19)
0.72 (0.37, 1.39)
Amrubicin vs Erl+Tiv
1.05 (0.70, 1.61)
0.73 (0.39, 1.38)
Amrubicin vs Erlotinib
1.00 (0.67, 1.47)
0.73 (0.09, 6.32)
0.68 (0.37, 1.22)
Amrubicin vs Gefitinib
0.97 (0.67, 1.41)
0.60 (0.31, 1.12)
Amrubicin vs Nivolumab
1.47 (0.98, 2.22)
1.38 (0.17, 11.15)
1.38 (0.80, 2.35)
Amrubicin vs Pem+Cet
0.97 (0.62, 1.49)
0.66 (0.31, 1.32)
Amrubicin vs Pem+Erl
1.36 (0.86, 2.18)
0.87 (0.42, 1.79)
Amrubicin vs Pem+Nin
0.95 (0.62, 1.49)
0.61 (0.29, 1.22)
Amrubicin vs Pembrolizumab
1.42 (0.93, 2.20)
1.10 (0.14, 8.98)
1.59 (0.92, 2.78)
Amrubicin vs Pemetrexed
0.97 (0.66, 1.42)
0.62 (0.31, 1.17)
Amrubicin vs Placebo
0.83 (0.56, 1.22)
0.49 (0.04, 6.48)
0.49 (0.25, 0.92)
Amrubicin vs Pralatrexate
1.19 (0.72, 2.01)
0.69 (0.05, 9.19)
1.04 (0.48, 2.27)
Amrubicin vs Teg/ura+Gef
1.20 (0.63, 2.40)
0.73 (0.30, 1.69)
Amrubicin vs Trametinib
1.06 (0.49, 2.33)
1.05 (0.47, 2.29)
Amrubicin vs Vandetanib
0.96 (0.63, 1.46)
0.58 (0.05, 7.64)
0.68 (0.35, 1.32)
Total population
Only squamous population
Only non-squamous population
.2
.3
.5
1
2
5

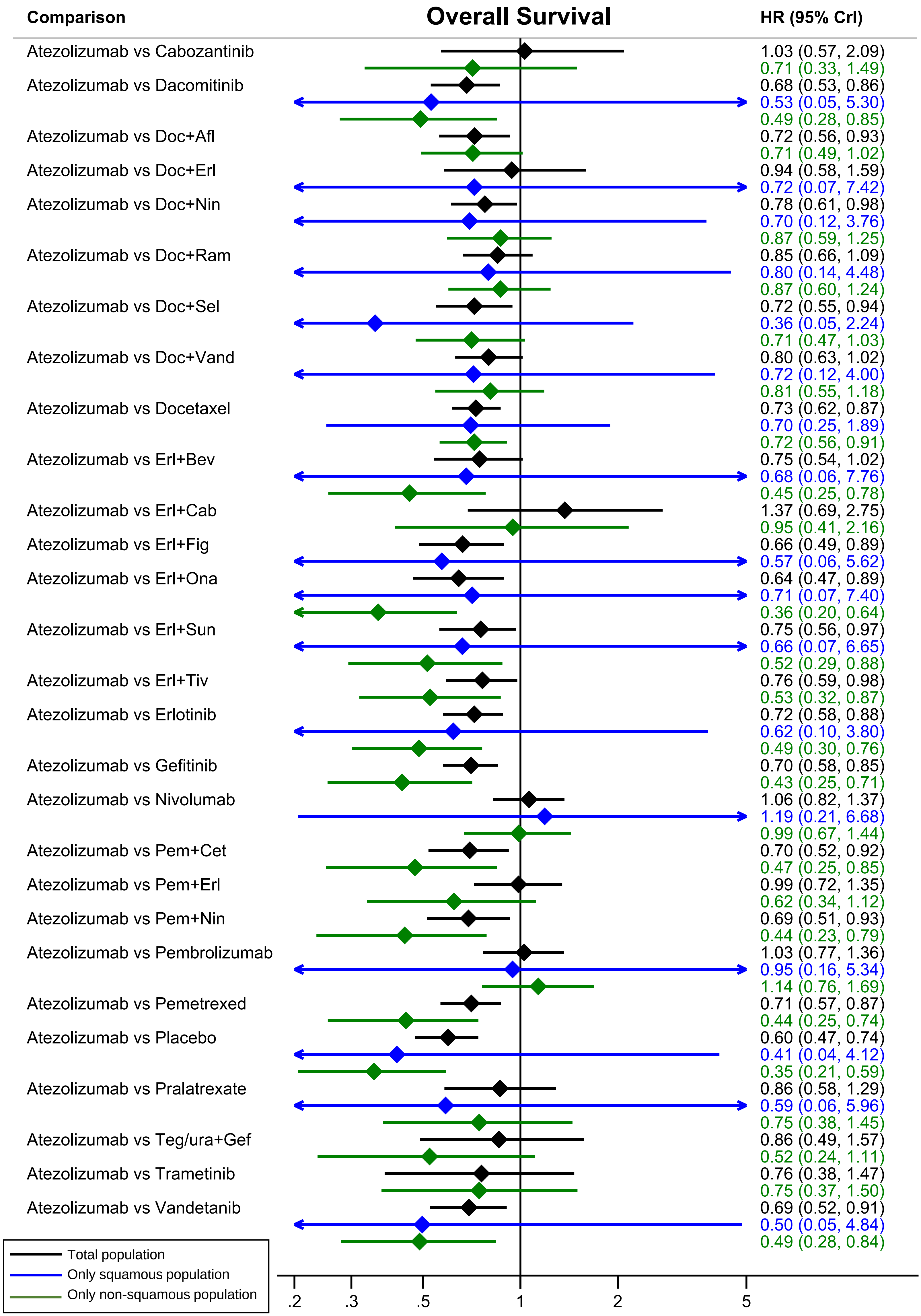
Comparison
Overall Survival
HR (95% CrI)
Atezolizumab vs Cabozantinib
1.03 (0.57, 2.09)
0.71 (0.33, 1.49)
Atezolizumab vs Dacomitinib
0.68 (0.53, 0.86)
0.53 (0.05, 5.30)
0.49 (0.28, 0.85)
Atezolizumab vs Doc+Afl
0.72 (0.56, 0.93)
0.71 (0.49, 1.02)
Atezolizumab vs Doc+Erl
0.94 (0.58, 1.59)
0.72 (0.07, 7.42)
Atezolizumab vs Doc+Nin
0.78 (0.61, 0.98)
0.70 (0.12, 3.76)
0.87 (0.59, 1.25)
Atezolizumab vs Doc+Ram
0.85 (0.66, 1.09)
0.80 (0.14, 4.48)
0.87 (0.60, 1.24)
Atezolizumab vs Doc+Sel
0.72 (0.55, 0.94)
0.36 (0.05, 2.24)
0.71 (0.47, 1.03)
Atezolizumab vs Doc+Vand
0.80 (0.63, 1.02)
0.72 (0.12, 4.00)
0.81 (0.55, 1.18)
Atezolizumab vs Docetaxel
0.73 (0.62, 0.87)
0.70 (0.25, 1.89)
0.72 (0.56, 0.91)
Atezolizumab vs Erl+Bev
0.75 (0.54, 1.02)
0.68 (0.06, 7.76)
0.45 (0.25, 0.78)
Atezolizumab vs Erl+Cab
1.37 (0.69, 2.75)
0.95 (0.41, 2.16)
Atezolizumab vs Erl+Fig
0.66 (0.49, 0.89)
0.57 (0.06, 5.62)
Atezolizumab vs Erl+Ona
0.64 (0.47, 0.89)
0.71 (0.07, 7.40)
0.36 (0.20, 0.64)
Atezolizumab vs Erl+Sun
0.75 (0.56, 0.97)
0.66 (0.07, 6.65)
0.52 (0.29, 0.88)
Atezolizumab vs Erl+Tiv
0.76 (0.59, 0.98)
0.53 (0.32, 0.87)
Atezolizumab vs Erlotinib
0.72 (0.58, 0.88)
0.62 (0.10, 3.80)
0.49 (0.30, 0.76)
Atezolizumab vs Gefitinib
0.70 (0.58, 0.85)
0.43 (0.25, 0.71)
Atezolizumab vs Nivolumab
1.06 (0.82, 1.37)
1.19 (0.21, 6.68)
0.99 (0.67, 1.44)
Atezolizumab vs Pem+Cet
0.70 (0.52, 0.92)
0.47 (0.25, 0.85)
Atezolizumab vs Pem+Erl
0.99 (0.72, 1.35)
0.62 (0.34, 1.12)
Atezolizumab vs Pem+Nin
0.69 (0.51, 0.93)
0.44 (0.23, 0.79)
Atezolizumab vs Pembrolizumab
1.03 (0.77, 1.36)
0.95 (0.16, 5.34)
1.14 (0.76, 1.69)
Atezolizumab vs Pemetrexed
0.71 (0.57, 0.87)
0.44 (0.25, 0.74)
Atezolizumab vs Placebo
0.60 (0.47, 0.74)
0.41 (0.04, 4.12)
0.35 (0.21, 0.59)
Atezolizumab vs Pralatrexate
0.86 (0.58, 1.29)
0.59 (0.06, 5.96)
0.75 (0.38, 1.45)
Atezolizumab vs Teg/ura+Gef
0.86 (0.49, 1.57)
0.52 (0.24, 1.11)
Atezolizumab vs Trametinib
0.76 (0.38, 1.47)
0.75 (0.37, 1.50)
Atezolizumab vs Vandetanib
0.69 (0.52, 0.91)
0.50 (0.05, 4.84)
0.49 (0.28, 0.84)
Total population
Only squamous population
Only non-squamous population
.2
.3
.5
1
2
5

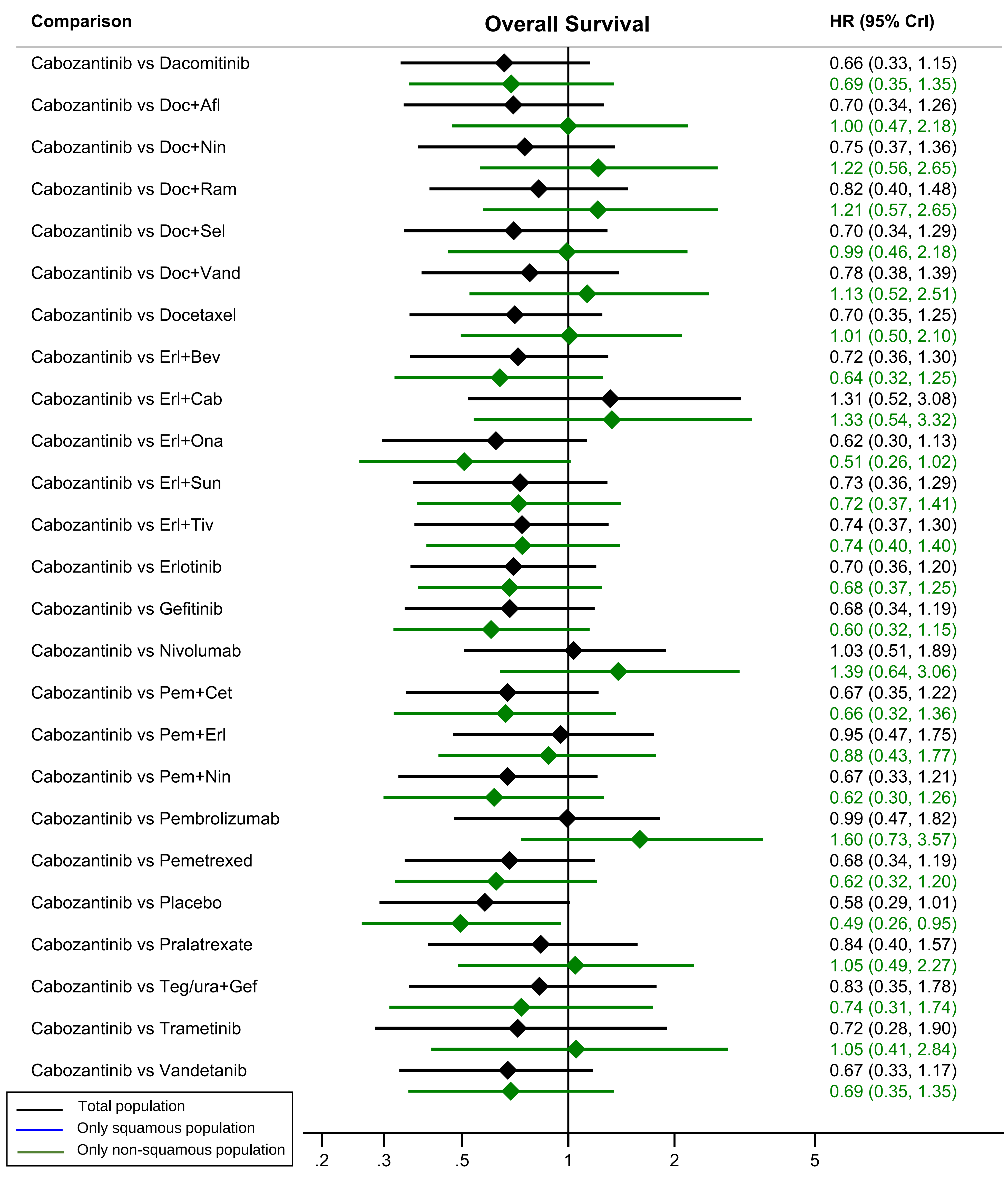
Comparison
Overall Survival
HR (95% Crl)
Cabozantinib vs Dacomitinib
0.66 (0.33, 1.15)
0.69 (0.35, 1.35)
Cabozantinib vs Doc+Afl
0.70 (0.34, 1.26)
1.00 (0.47, 2.18)
Cabozantinib vs Doc+Nin
0.75 (0.37, 1.36)
1.22 (0.56, 2.65)
Cabozantinib vs Doc+Ram
0.82 (0.40, 1.48)
1.21 (0.57, 2.65)
Cabozantinib vs Doc+Sel
0.70 (0.34, 1.29)
0.99 (0.46, 2.18)
Cabozantinib vs Doc+Vand
0.78 (0.38, 1.39)
1.13 (0.52, 2.51)
Cabozantinib vs Docetaxel
0.70 (0.35, 1.25)
1.01 (0.50, 2.10)
Cabozantinib vs Erl+Bev
0.72 (0.36, 1.30)
0.64 (0.32, 1.25)
Cabozantinib vs Erl+Cab
1.31 (0.52, 3.08)
1.33 (0.54, 3.32)
Cabozantinib vs Erl+Ona
0.62 (0.30, 1.13)
0.51 (0.26, 1.02)
Cabozantinib vs Erl+Sun
0.73 (0.36, 1.29)
0.72 (0.37, 1.41)
Cabozantinib vs Erl+Tiv
0.74 (0.37, 1.30)
0.74 (0.40, 1.40)
Cabozantinib vs Erlotinib
0.70 (0.36, 1.20)
0.68 (0.37, 1.25)
Cabozantinib vs Gefitinib
0.68 (0.34, 1.19)
0.60 (0.32, 1.15)
Cabozantinib vs Nivolumab
1.03 (0.51, 1.89)
1.39 (0.64, 3.06)
Cabozantinib vs Pem+Cet
0.67 (0.35, 1.22)
0.66 (0.32, 1.36)
Cabozantinib vs Pem+Erl
0.95 (0.47, 1.75)
0.88 (0.43, 1.77)
Cabozantinib vs Pem+Nin
0.67 (0.33, 1.21)
0.62 (0.30, 1.26)
Cabozantinib vs Pembrolizumab
0.99 (0.47, 1.82)
1.60 (0.73, 3.57)
Cabozantinib vs Pemetrexed
0.68 (0.34, 1.19)
0.62 (0.32, 1.20)
Cabozantinib vs Placebo
0.58 (0.29, 1.01)
0.49 (0.26, 0.95)
Cabozantinib vs Pralatrexate
0.84 (0.40, 1.57)
1.05 (0.49, 2.27)
Cabozantinib vs Teg/ura+Gef
0.83 (0.35, 1.78)
0.74 (0.31, 1.74)
Cabozantinib vs Trametinib
0.72 (0.28, 1.90)
1.05 (0.41, 2.84)
Cabozantinib vs Vandetanib
0.67 (0.33, 1.17)
0.69 (0.35, 1.35)
Total population
Only squamous population
Only non-squamous population
.2
.3
.5
1
2
5

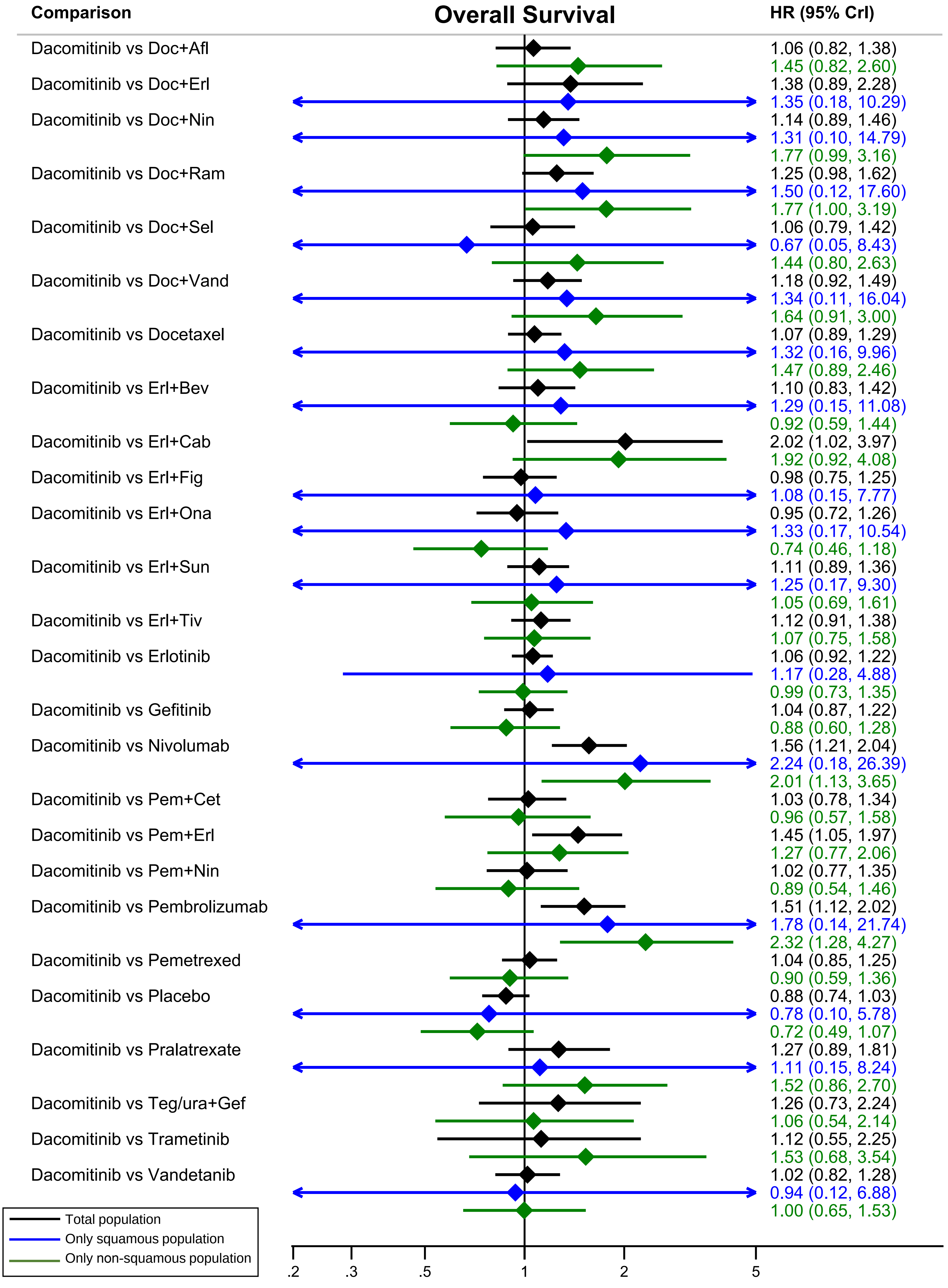
Comparison
Overall Survival
HR (95% CrI)
Dacomitinib vs Doc+Afl
1.06 (0.82, 1.38)
1.45 (0.82, 2.60)
Dacomitinib vs Doc+Erl
1.38 (0.89, 2.28)
1.35 (0.18, 10.29)
Dacomitinib vs Doc+Nin
1.14 (0.89, 1.46)
1.31 (0.10, 14.79)
1.77 (0.99, 3.16)
Dacomitinib vs Doc+Ram
1.25 (0.98, 1.62)
1.50 (0.12, 17.60)
1.77 (1.00, 3.19)
Dacomitinib vs Doc+Sel
1.06 (0.79, 1.42)
0.67 (0.05, 8.43)
1.44 (0.80, 2.63)
Dacomitinib vs Doc+Vand
1.18 (0.92, 1.49)
1.34 (0.11, 16.04)
1.64 (0.91, 3.00)
Dacomitinib vs Docetaxel
1.07 (0.89, 1.29)
1.32 (0.16, 9.96)
1.47 (0.89, 2.46)
Dacomitinib vs Erl+Bev
1.10 (0.83, 1.42)
1.29 (0.15, 11.08)
0.92 (0.59, 1.44)
Dacomitinib vs Erl+Cab
2.02 (1.02, 3.97)
1.92 (0.92, 4.08)
Dacomitinib vs Erl+Fig
0.98 (0.75, 1.25)
1.08 (0.15, 7.77)
Dacomitinib vs Erl+Ona
0.95 (0.72, 1.26)
1.33 (0.17, 10.54)
0.74 (0.46, 1.18)
Dacomitinib vs Erl+Sun
1.11 (0.89, 1.36)
1.25 (0.17, 9.30)
1.05 (0.69, 1.61)
Dacomitinib vs Erl+Tiv
1.12 (0.91, 1.38)
1.07 (0.75, 1.58)
Dacomitinib vs Erlotinib
1.06 (0.92, 1.22)
1.17 (0.28, 4.88)
0.99 (0.73, 1.35)
Dacomitinib vs Gefitinib
1.04 (0.87, 1.22)
0.88 (0.60, 1.28)
Dacomitinib vs Nivolumab
1.56 (1.21, 2.04)
2.24 (0.18, 26.39)
2.01 (1.13, 3.65)
Dacomitinib vs Pem+Cet
1.03 (0.78, 1.34)
0.96 (0.57, 1.58)
Dacomitinib vs Pem+Erl
1.45 (1.05, 1.97)
1.27 (0.77, 2.06)
Dacomitinib vs Pem+Nin
1.02 (0.77, 1.35)
0.89 (0.54, 1.46)
Dacomitinib vs Pembrolizumab
1.51 (1.12, 2.02)
1.78 (0.14, 21.74)
2.32 (1.28, 4.27)
Dacomitinib vs Pemetrexed
1.04 (0.85, 1.25)
0.90 (0.59, 1.36)
Dacomitinib vs Placebo
0.88 (0.74, 1.03)
0.78 (0.10, 5.78)
0.72 (0.49, 1.07)
Dacomitinib vs Pralatrexate
1.27 (0.89, 1.81)
1.11 (0.15, 8.24)
1.52 (0.86, 2.70)
Dacomitinib vs Teg/ura+Gef
1.26 (0.73, 2.24)
1.06 (0.54, 2.14)
Dacomitinib vs Trametinib
1.12 (0.55, 2.25)
1.53 (0.68, 3.54)
Dacomitinib vs Vandetanib
1.02 (0.82, 1.28)
0.94 (0.12, 6.88)
1.00 (0.65, 1.53)
Total population
Only squamous population
Only non-squamous population
.2
.3
.5
1
2
5

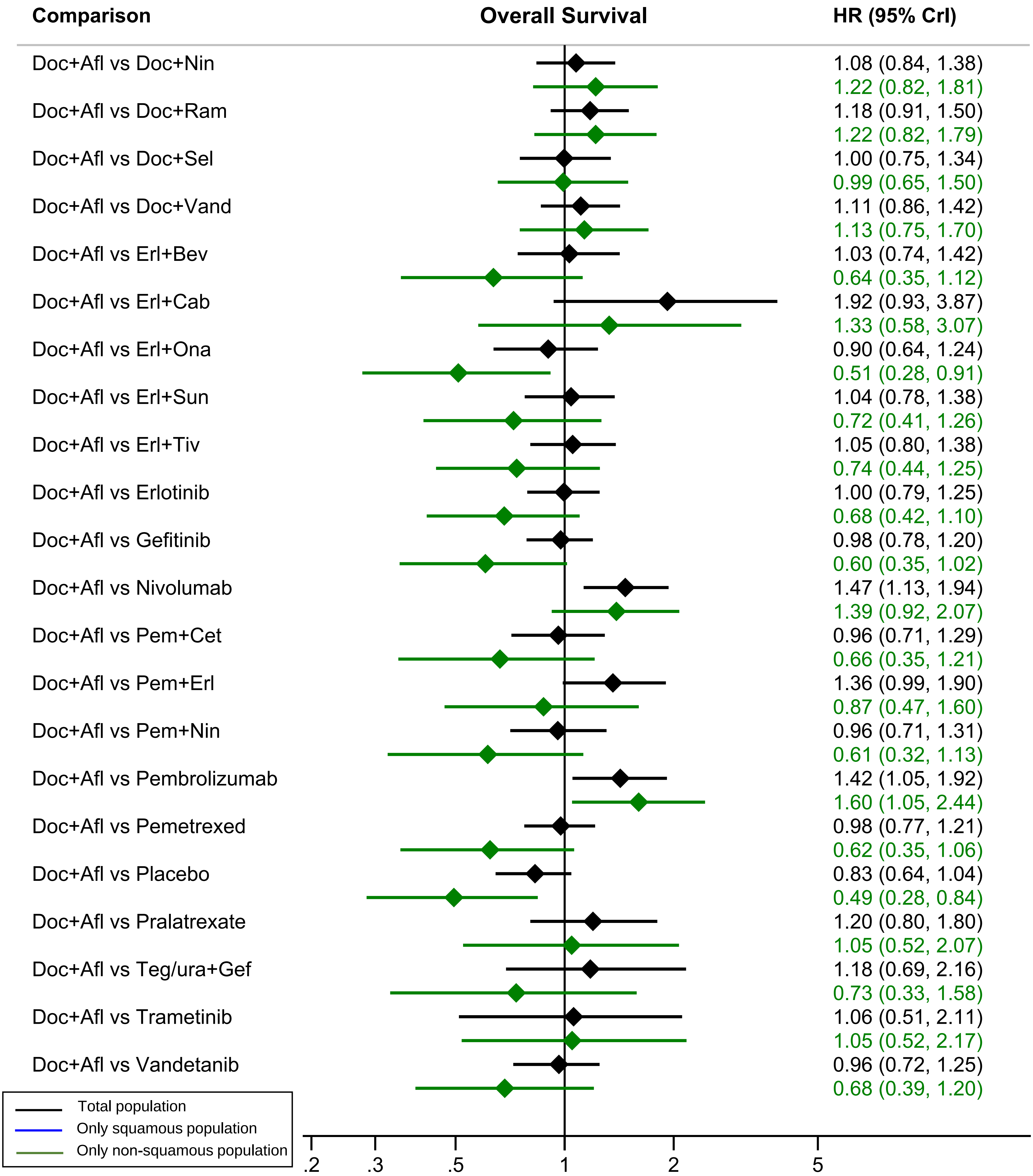
Comparison
Overall Survival
HR (95% CrI)
Doc+Afl vs Doc+Nin
1.08 (0.84, 1.38)
1.22 (0.82, 1.81)
Doc+Afl vs Doc+Ram
1.18 (0.91, 1.50)
1.22 (0.82, 1.79)
Doc+Afl vs Doc+Sel
1.00 (0.75, 1.34)
0.99 (0.65, 1.50)
Doc+Afl vs Doc+Vand
1.11 (0.86, 1.42)
1.13 (0.75, 1.70)
Doc+Afl vs Erl+Bev
1.03 (0.74, 1.42)
0.64 (0.35, 1.12)
Doc+Afl vs Erl+Cab
1.92 (0.93, 3.87)
1.33 (0.58, 3.07)
Doc+Afl vs Erl+Ona
0.90 (0.64, 1.24)
0.51 (0.28, 0.91)
Doc+Afl vs Erl+Sun
1.04 (0.78, 1.38)
0.72 (0.41, 1.26)
Doc+Afl vs Erl+Tiv
1.05 (0.80, 1.38)
0.74 (0.44, 1.25)
Doc+Afl vs Erlotinib
1.00 (0.79, 1.25)
0.68 (0.42, 1.10)
Doc+Afl vs Gefitinib
0.98 (0.78, 1.20)
0.60 (0.35, 1.02)
Doc+Afl vs Nivolumab
1.47 (1.13, 1.94)
1.39 (0.92, 2.07)
Doc+Afl vs Pem+Cet
0.96 (0.71, 1.29)
0.66 (0.35, 1.21)
Doc+Afl vs Pem+Erl
1.36 (0.99, 1.90)
0.87 (0.47, 1.60)
Doc+Afl vs Pem+Nin
0.96 (0.71, 1.31)
0.61 (0.32, 1.13)
Doc+Afl vs Pembrolizumab
1.42 (1.05, 1.92)
1.60 (1.05, 2.44)
Doc+Afl vs Pemetrexed
0.98 (0.77, 1.21)
0.62 (0.35, 1.06)
Doc+Afl vs Placebo
0.83 (0.64, 1.04)
0.49 (0.28, 0.84)
Doc+Afl vs Pralatrexate
1.20 (0.80, 1.80)
1.05 (0.52, 2.07)
Doc+Afl vs Teg/ura+Gef
1.18 (0.69, 2.16)
0.73 (0.33, 1.58)
Doc+Afl vs Trametinib
1.06 (0.51, 2.11)
1.05 (0.52, 2.17)
Doc+Afl vs Vandetanib
0.96 (0.72, 1.25)
0.68 (0.39, 1.20)
Total population
Only squamous population
Only non-squamous population
.2
.3
.5
1
2
5

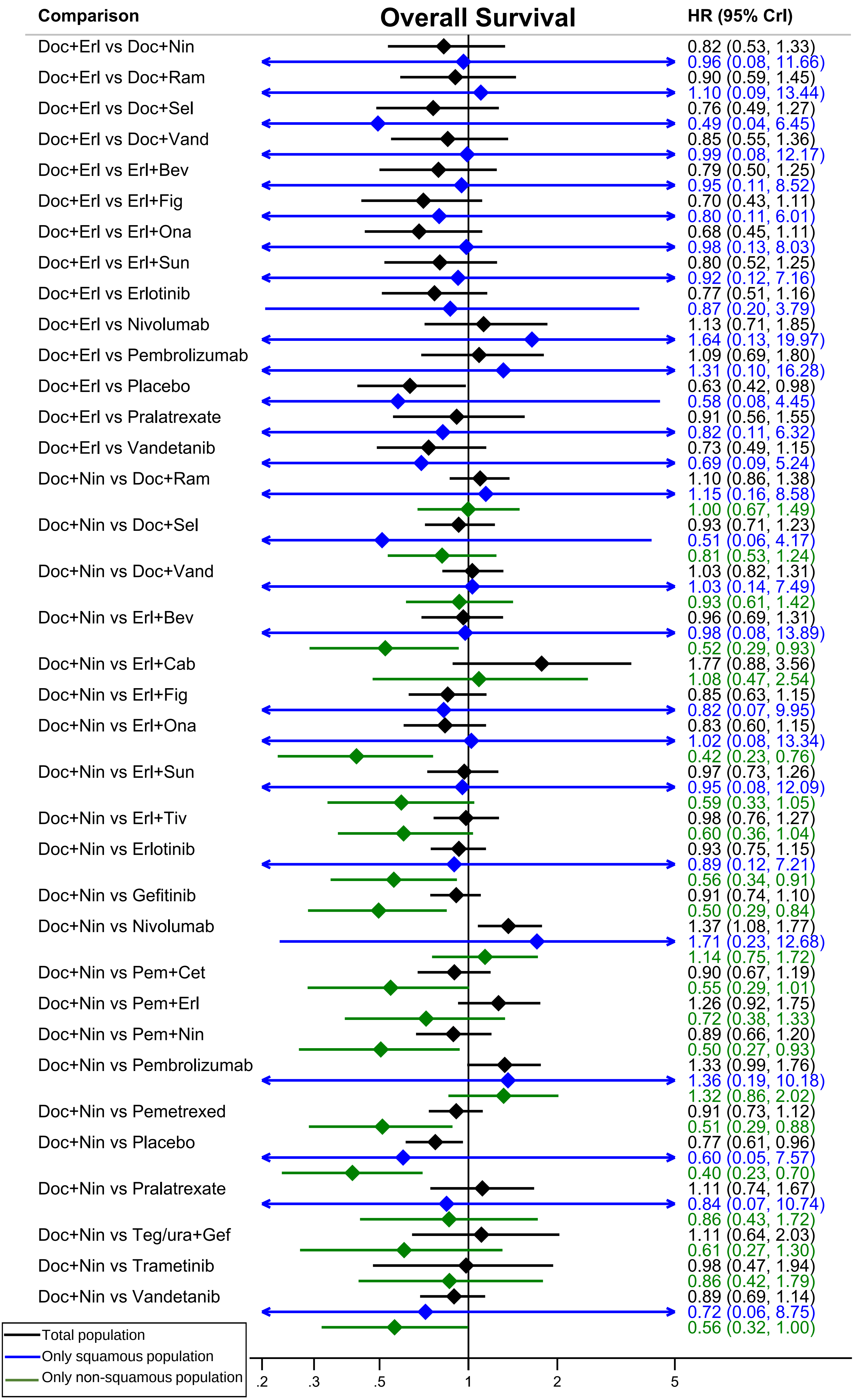
Comparison
Overall Survival
HR (95% CrI)
Doc+Erl vs Doc+Nin
0.82 (0.53, 1.33)
0.96 (0.08, 11.66)
Doc+Erl vs Doc+Ram
0.90 (0.59, 1.45)
1.10 (0.09, 13.44)
Doc+Erl vs Doc+Sel
0.76 (0.49, 1.27)
0.49 (0.04, 6.45)
Doc+Erl vs Doc+Vand
0.85 (0.55, 1.36)
0.99 (0.08, 12.17)
Doc+Erl vs Erl+Bev
0.79 (0.50, 1.25)
0.95 (0.11, 8.52)
Doc+Erl vs Erl+Fig
0.70 (0.43, 1.11)
0.80 (0.11, 6.01)
Doc+Erl vs Erl+Ona
0.68 (0.45, 1.11)
0.98 (0.13, 8.03)
Doc+Erl vs Erl+Sun
0.80 (0.52, 1.25)
0.92 (0.12, 7.16)
Doc+Erl vs Erlotinib
0.77 (0.51, 1.16)
0.87 (0.20, 3.79)
Doc+Erl vs Nivolumab
1.13 (0.71, 1.85)
1.64 (0.13, 19.97)
Doc+Erl vs Pembrolizumab
1.09 (0.69, 1.80)
1.31 (0.10, 16.28)
Doc+Erl vs Placebo
0.63 (0.42, 0.98)
0.58 (0.08, 4.45)
Doc+Erl vs Pralatrexate
0.91 (0.56, 1.55)
0.82 (0.11, 6.32)
Doc+Erl vs Vandetanib
0.73 (0.49, 1.15)
0.69 (0.09, 5.24)
Doc+Nin vs Doc+Ram
1.10 (0.86, 1.38)
1.15 (0.16, 8.58)
1.00 (0.67, 1.49)
Doc+Nin vs Doc+Sel
0.93 (0.71, 1.23)
0.51 (0.06, 4.17)
0.81 (0.53, 1.24)
Doc+Nin vs Doc+Vand
1.03 (0.82, 1.31)
1.03 (0.14, 7.49)
0.93 (0.61, 1.42)
Doc+Nin vs Erl+Bev
0.96 (0.69, 1.31)
0.98 (0.08, 13.89)
0.52 (0.29, 0.93)
Doc+Nin vs Erl+Cab
1.77 (0.88, 3.56)
1.08 (0.47, 2.54)
Doc+Nin vs Erl+Fig
0.85 (0.63, 1.15)
0.82 (0.07, 9.95)
Doc+Nin vs Erl+Ona
0.83 (0.60, 1.15)
1.02 (0.08, 13.34)
0.42 (0.23, 0.76)
Doc+Nin vs Erl+Sun
0.97 (0.73, 1.26)
0.95 (0.08, 12.09)
0.59 (0.33, 1.05)
Doc+Nin vs Erl+Tiv
0.98 (0.76, 1.27)
0.60 (0.36, 1.04)
Doc+Nin vs Erlotinib
0.93 (0.75, 1.15)
0.89 (0.12, 7.21)
0.56 (0.34, 0.91)
Doc+Nin vs Gefitinib
0.91 (0.74, 1.10)
0.50 (0.29, 0.84)
Doc+Nin vs Nivolumab
1.37 (1.08, 1.77)
1.71 (0.23, 12.68)
1.14 (0.75, 1.72)
Doc+Nin vs Pem+Cet
0.90 (0.67, 1.19)
0.55 (0.29, 1.01)
Doc+Nin vs Pem+Erl
1.26 (0.92, 1.75)
0.72 (0.38, 1.33)
Doc+Nin vs Pem+Nin
0.89 (0.66, 1.20)
0.50 (0.27, 0.93)
Doc+Nin vs Pembrolizumab
1.33 (0.99, 1.76)
1.36 (0.19, 10.18)
1.32 (0.86, 2.02)
Doc+Nin vs Pemetrexed
0.91 (0.73, 1.12)
0.51 (0.29, 0.88)
Doc+Nin vs Placebo
0.77 (0.61, 0.96)
0.60 (0.05, 7.57)
0.40 (0.23, 0.70)
Doc+Nin vs Pralatrexate
1.11 (0.74, 1.67)
0.84 (0.07, 10.74)
0.86 (0.43, 1.72)
Doc+Nin vs Teg/ura+Gef
1.11 (0.64, 2.03)
0.61 (0.27, 1.30)
Doc+Nin vs Trametinib
0.98 (0.47, 1.94)
0.86 (0.42, 1.79)
Doc+Nin vs Vandetanib
0.89 (0.69, 1.14)
0.72 (0.06, 8.75)
0.56 (0.32, 1.00)
Total population
Only squamous population
Only non-squamous population
.2
.3
.5
1
2
5

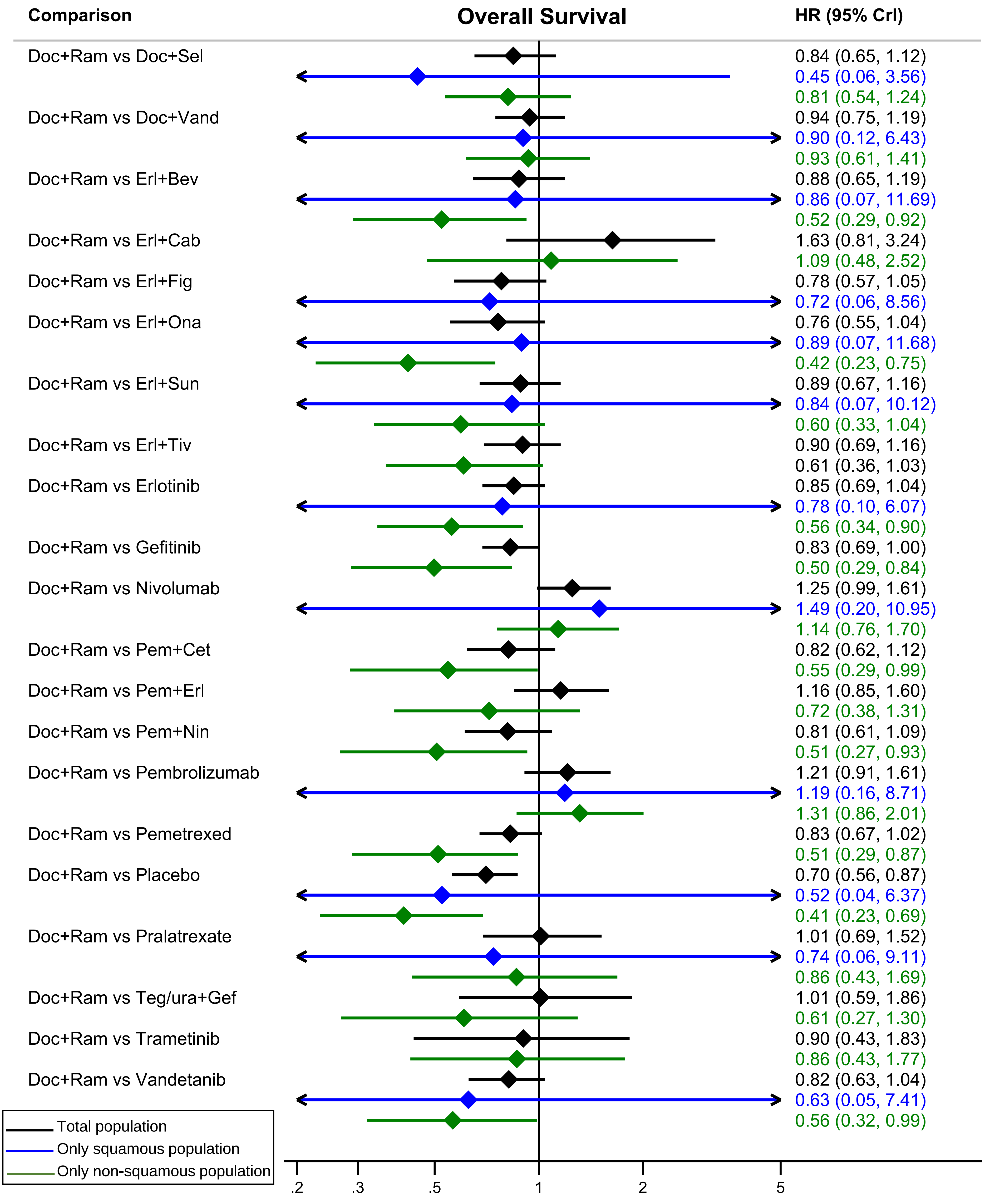
Comparison
Overall Survival
HR (95% CrI)
Doc+Ram vs Doc+Sel
0.84 (0.65, 1.12)
0.45 (0.06, 3.56)
0.81 (0.54, 1.24)
Doc+Ram vs Doc+Vand
0.94 (0.75, 1.19)
0.90 (0.12, 6.43)
0.93 (0.61, 1.41)
Doc+Ram vs Erl+Bev
0.88 (0.65, 1.19)
0.86 (0.07, 11.69)
0.52 (0.29, 0.92)
Doc+Ram vs Erl+Cab
1.63 (0.81, 3.24)
1.09 (0.48, 2.52)
Doc+Ram vs Erl+Fig
0.78 (0.57, 1.05)
0.72 (0.06, 8.56)
Doc+Ram vs Erl+Ona
0.76 (0.55, 1.04)
0.89 (0.07, 11.68)
0.42 (0.23, 0.75)
Doc+Ram vs Erl+Sun
0.89 (0.67, 1.16)
0.84 (0.07, 10.12)
0.60 (0.33, 1.04)
Doc+Ram vs Erl+Tiv
0.90 (0.69, 1.16)
0.61 (0.36, 1.03)
Doc+Ram vs Erlotinib
0.85 (0.69, 1.04)
0.78 (0.10, 6.07)
0.56 (0.34, 0.90)
Doc+Ram vs Gefitinib
0.83 (0.69, 1.00)
0.50 (0.29, 0.84)
Doc+Ram vs Nivolumab
1.25 (0.99, 1.61)
1.49 (0.20, 10.95)
1.14 (0.76, 1.70)
Doc+Ram vs Pem+Cet
0.82 (0.62, 1.12)
0.55 (0.29, 0.99)
Doc+Ram vs Pem+Erl
1.16 (0.85, 1.60)
0.72 (0.38, 1.31)
Doc+Ram vs Pem+Nin
0.81 (0.61, 1.09)
0.51 (0.27, 0.93)
Doc+Ram vs Pembrolizumab
1.21 (0.91, 1.61)
1.19 (0.16, 8.71)
1.31 (0.86, 2.01)
Doc+Ram vs Pemetrexed
0.83 (0.67, 1.02)
0.51 (0.29, 0.87)
Doc+Ram vs Placebo
0.70 (0.56, 0.87)
0.52 (0.04, 6.37)
0.41 (0.23, 0.69)
Doc+Ram vs Pralatrexate
1.01 (0.69, 1.52)
0.74 (0.06, 9.11)
0.86 (0.43, 1.69)
Doc+Ram vs Teg/ura+Gef
1.01 (0.59, 1.86)
0.61 (0.27, 1.30)
Doc+Ram vs Trametinib
0.90 (0.43, 1.83)
0.86 (0.43, 1.77)
Doc+Ram vs Vandetanib
0.82 (0.63, 1.04)
0.63 (0.05, 7.41)
0.56 (0.32, 0.99)
Total population
Only squamous population
Only non-squamous population
.2
.3
.5
1
2
5

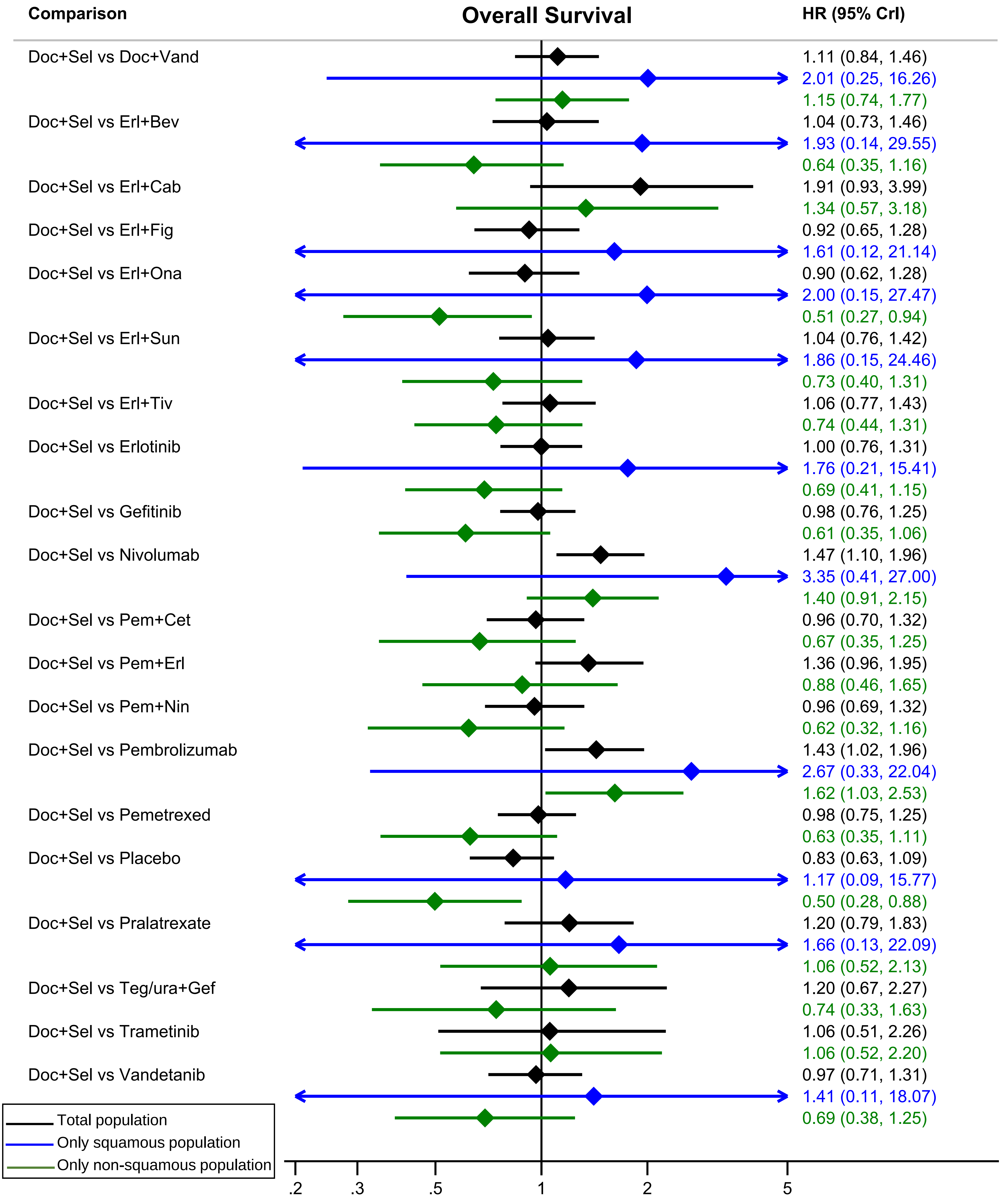
Comparison
Overall Survival
HR (95% Crl)
Doc+Sel vs Doc+Vand
1.11 (0.84, 1.46)
2.01 (0.25, 16.26)
1.15 (0.74, 1.77)
Doc+Sel vs Erl+Bev
1.04 (0.73, 1.46)
1.93 (0.14, 29.55)
0.64 (0.35, 1.16)
Doc+Sel vs Erl+Cab
1.91 (0.93, 3.99)
1.34 (0.57, 3.18)
Doc+Sel vs Erl+Fig
0.92 (0.65, 1.28)
1.61 (0.12, 21.14)
Doc+Sel vs Erl+Ona
0.90 (0.62, 1.28)
2.00 (0.15, 27.47)
0.51 (0.27, 0.94)
Doc+Sel vs Erl+Sun
1.04 (0.76, 1.42)
1.86 (0.15, 24.46)
0.73 (0.40, 1.31)
Doc+Sel vs Erl+Tiv
1.06 (0.77, 1.43)
0.74 (0.44, 1.31)
Doc+Sel vs Erlotinib
1.00 (0.76, 1.31)
1.76 (0.21, 15.41)
0.69 (0.41, 1.15)
Doc+Sel vs Gefitinib
0.98 (0.76, 1.25)
0.61 (0.35, 1.06)
Doc+Sel vs Nivolumab
1.47 (1.10, 1.96)
3.35 (0.41, 27.00)
1.40 (0.91, 2.15)
Doc+Sel vs Pem+Cet
0.96 (0.70, 1.32)
0.67 (0.35, 1.25)
Doc+Sel vs Pem+Erl
1.36 (0.96, 1.95)
0.88 (0.46, 1.65)
Doc+Sel vs Pem+Nin
0.96 (0.69, 1.32)
0.62 (0.32, 1.16)
Doc+Sel vs Pembrolizumab
1.43 (1.02, 1.96)
2.67 (0.33, 22.04)
1.62 (1.03, 2.53)
Doc+Sel vs Pemetrexed
0.98 (0.75, 1.25)
0.63 (0.35, 1.11)
Doc+Sel vs Placebo
0.83 (0.63, 1.09)
1.17 (0.09, 15.77)
0.50 (0.28, 0.88)
Doc+Sel vs Pralatrexate
1.20 (0.79, 1.83)
1.66 (0.13, 22.09)
1.06 (0.52, 2.13)
Doc+Sel vs Teg/ura+Gef
1.20 (0.67, 2.27)
0.74 (0.33, 1.63)
Doc+Sel vs Trametinib
1.06 (0.51, 2.26)
1.06 (0.52, 2.20)
Doc+Sel vs Vandetanib
0.97 (0.71, 1.31)
1.41 (0.11, 18.07)
0.69 (0.38, 1.25)
Total population
Only squamous population
Only non-squamous population
.2
.3
.5
1
2
5

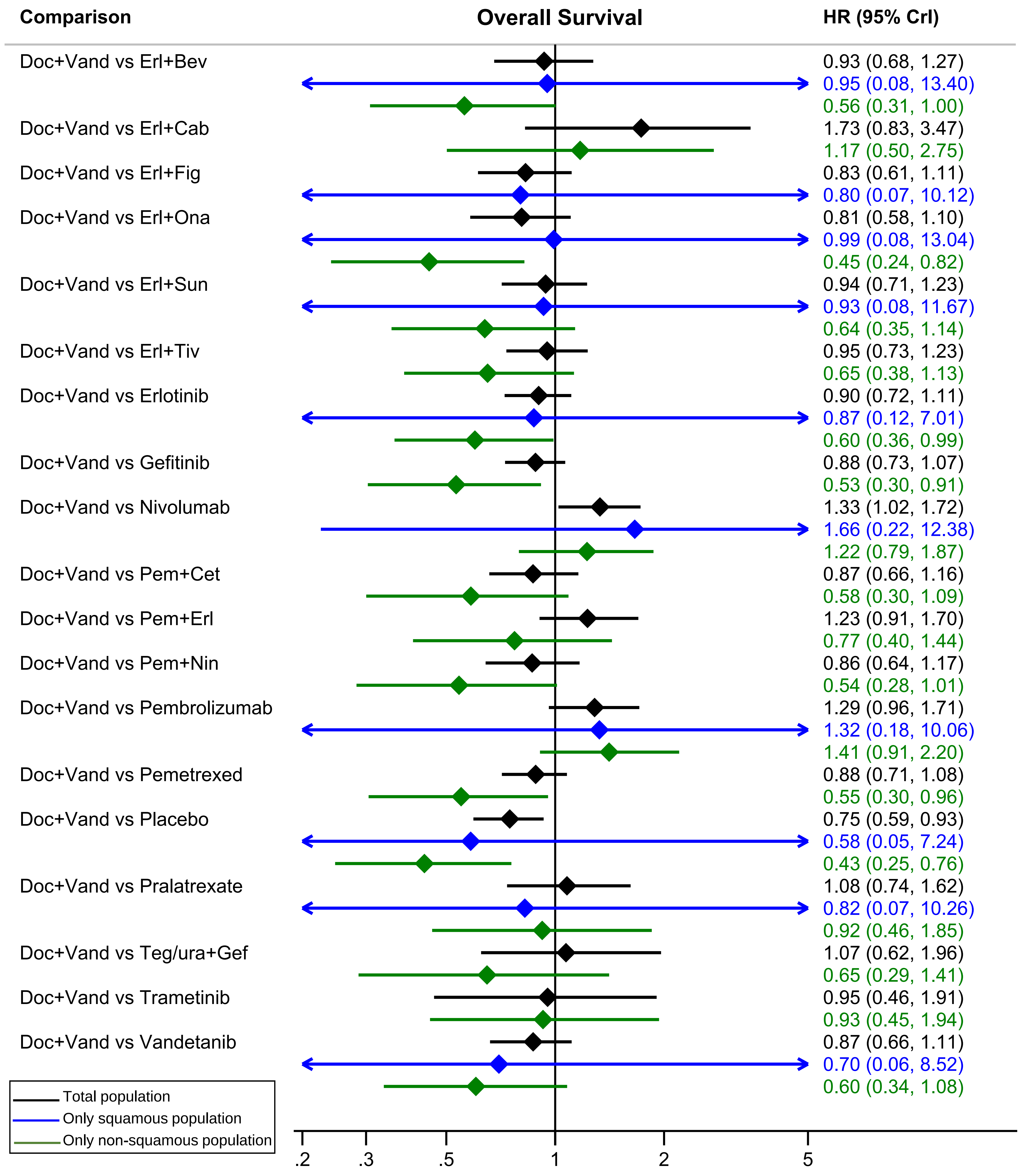
Comparison
Overall Survival
HR (95% CrI)
Doc+Vand vs Erl+Bev
0.93 (0.68, 1.27)
0.95 (0.08, 13.40)
0.56 (0.31, 1.00)
Doc+Vand vs Erl+Cab
1.73 (0.83, 3.47)
1.17 (0.50, 2.75)
Doc+Vand vs Erl+Fig
0.83 (0.61, 1.11)
0.80 (0.07, 10.12)
Doc+Vand vs Erl+Ona
0.81 (0.58, 1.10)
0.99 (0.08, 13.04)
0.45 (0.24, 0.82)
Doc+Vand vs Erl+Sun
0.94 (0.71, 1.23)
0.93 (0.08, 11.67)
0.64 (0.35, 1.14)
Doc+Vand vs Erl+Tiv
0.95 (0.73, 1.23)
0.65 (0.38, 1.13)
Doc+Vand vs Erlotinib
0.90 (0.72, 1.11)
0.87 (0.12, 7.01)
0.60 (0.36, 0.99)
Doc+Vand vs Gefitinib
0.88 (0.73, 1.07)
0.53 (0.30, 0.91)
Doc+Vand vs Nivolumab
1.33 (1.02, 1.72)
1.66 (0.22, 12.38)
1.22 (0.79, 1.87)
Doc+Vand vs Pem+Cet
0.87 (0.66, 1.16)
0.58 (0.30, 1.09)
Doc+Vand vs Pem+Erl
1.23 (0.91, 1.70)
0.77 (0.40, 1.44)
Doc+Vand vs Pem+Nin
0.86 (0.64, 1.17)
0.54 (0.28, 1.01)
Doc+Vand vs Pembrolizumab
1.29 (0.96, 1.71)
1.32 (0.18, 10.06)
1.41 (0.91, 2.20)
Doc+Vand vs Pemetrexed
0.88 (0.71, 1.08)
0.55 (0.30, 0.96)
Doc+Vand vs Placebo
0.75 (0.59, 0.93)
0.58 (0.05, 7.24)
0.43 (0.25, 0.76)
Doc+Vand vs Pralatrexate
1.08 (0.74, 1.62)
0.82 (0.07, 10.26)
0.92 (0.46, 1.85)
Doc+Vand vs Teg/ura+Gef
1.07 (0.62, 1.96)
0.65 (0.29, 1.41)
Doc+Vand vs Trametinib
0.95 (0.46, 1.91)
0.93 (0.45, 1.94)
Doc+Vand vs Vandetanib
0.87 (0.66, 1.11)
0.70 (0.06, 8.52)
0.60 (0.34, 1.08)
Total population
Only squamous population
Only non-squamous population
.2
.3
.5
1
2
5

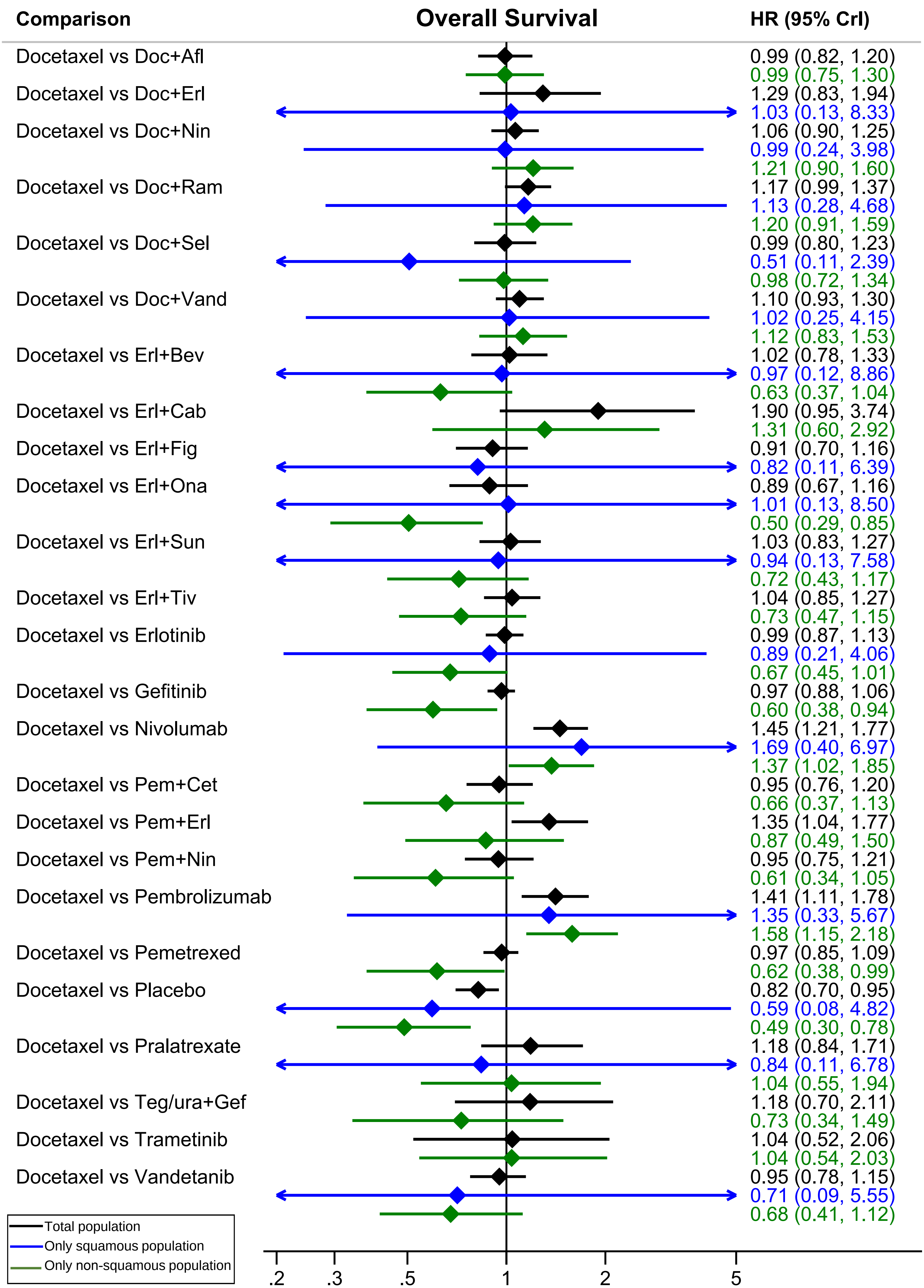
Comparison
Overall Survival
HR (95% CrI)
Docetaxel vs Doc+Afl
0.99 (0.82, 1.20)
0.99 (0.75, 1.30)
Docetaxel vs Doc+Erl
1.29 (0.83, 1.94)
1.03 (0.13, 8.33)
Docetaxel vs Doc+Nin
1.06 (0.90, 1.25)
0.99 (0.24, 3.98)
1.21 (0.90, 1.60)
Docetaxel vs Doc+Ram
1.17 (0.99, 1.37)
1.13 (0.28, 4.68)
1.20 (0.91, 1.59)
Docetaxel vs Doc+Sel
0.99 (0.80, 1.23)
0.51 (0.11, 2.39)
0.98 (0.72, 1.34)
Docetaxel vs Doc+Vand
1.10 (0.93, 1.30)
1.02 (0.25, 4.15)
1.12 (0.83, 1.53)
Docetaxel vs Erl+Bev
1.02 (0.78, 1.33)
0.97 (0.12, 8.86)
0.63 (0.37, 1.04)
Docetaxel vs Erl+Cab
1.90 (0.95, 3.74)
1.31 (0.60, 2.92)
Docetaxel vs Erl+Fig
0.91 (0.70, 1.16)
0.82 (0.11, 6.39)
Docetaxel vs Erl+Ona
0.89 (0.67, 1.16)
1.01 (0.13, 8.50)
0.50 (0.29, 0.85)
Docetaxel vs Erl+Sun
1.03 (0.83, 1.27)
0.94 (0.13, 7.58)
0.72 (0.43, 1.17)
Docetaxel vs Erl+Tiv
1.04 (0.85, 1.27)
0.73 (0.47, 1.15)
Docetaxel vs Erlotinib
0.99 (0.87, 1.13)
0.89 (0.21, 4.06)
0.67 (0.45, 1.01)
Docetaxel vs Gefitinib
0.97 (0.88, 1.06)
0.60 (0.38, 0.94)
Docetaxel vs Nivolumab
1.45 (1.21, 1.77)
1.69 (0.40, 6.97)
1.37 (1.02, 1.85)
Docetaxel vs Pem+Cet
0.95 (0.76, 1.20)
0.66 (0.37, 1.13)
Docetaxel vs Pem+Erl
1.35 (1.04, 1.77)
0.87 (0.49, 1.50)
Docetaxel vs Pem+Nin
0.95 (0.75, 1.21)
0.61 (0.34, 1.05)
Docetaxel vs Pembrolizumab
1.41 (1.11, 1.78)
1.35 (0.33, 5.67)
1.58 (1.15, 2.18)
Docetaxel vs Pemetrexed
0.97 (0.85, 1.09)
0.62 (0.38, 0.99)
Docetaxel vs Placebo
0.82 (0.70, 0.95)
0.59 (0.08, 4.82)
0.49 (0.30, 0.78)
Docetaxel vs Pralatrexate
1.18 (0.84, 1.71)
0.84 (0.11, 6.78)
1.04 (0.55, 1.94)
Docetaxel vs Teg/ura+Gef
1.18 (0.70, 2.11)
0.73 (0.34, 1.49)
Docetaxel vs Trametinib
1.04 (0.52, 2.06)
1.04 (0.54, 2.03)
Docetaxel vs Vandetanib
0.95 (0.78, 1.15)
0.71 (0.09, 5.55)
0.68 (0.41, 1.12)
Total population
Only squamous population
Only non-squamous population
.2
.3
.5
1
2
5

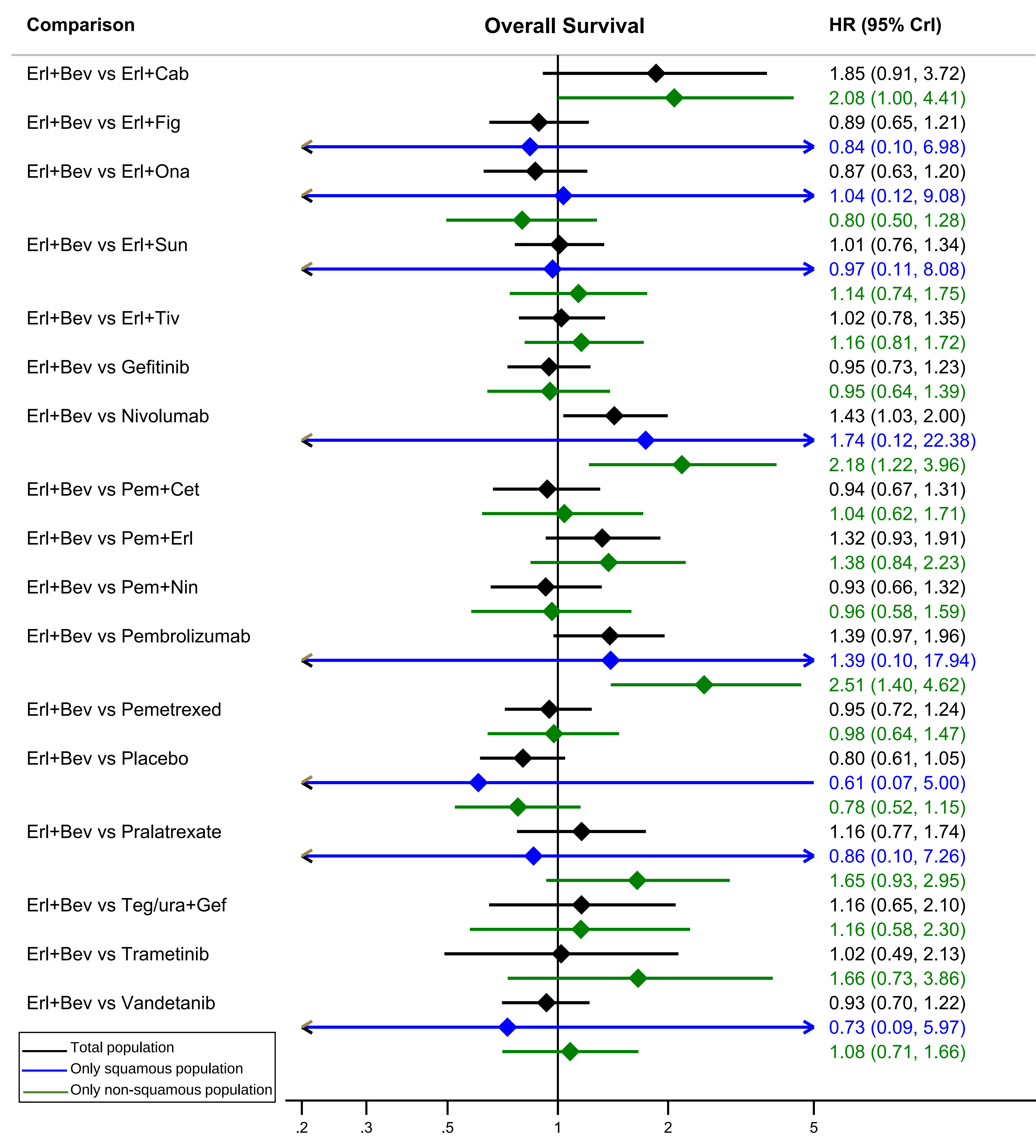
Comparison
Overall Survival
HR (95% CrI)
Erl+Bev vs Erl+Cab
1.85 (0.91, 3.72)
2.08 (1.00, 4.41)
Erl+Bev vs Erl+Fig
0.89 (0.65, 1.21)
0.84 (0.10, 6.98)
Erl+Bev vs Erl+Ona
0.87 (0.63, 1.20)
1.04 (0.12, 9.08)
0.80 (0.50, 1.28)
Erl+Bev vs Erl+Sun
1.01 (0.76, 1.34)
0.97 (0.11, 8.08)
1.14 (0.74, 1.75)
Erl+Bev vs Erl+Tiv
1.02 (0.78, 1.35)
1.16 (0.81, 1.72)
Erl+Bev vs Gefitinib
0.95 (0.73, 1.23)
0.95 (0.64, 1.39)
Erl+Bev vs Nivolumab
1.43 (1.03, 2.00)
1.74 (0.12, 22.38)
2.18 (1.22, 3.96)
Erl+Bev vs Pem+Cet
0.94 (0.67, 1.31)
1.04 (0.62, 1.71)
Erl+Bev vs Pem+Erl
1.32 (0.93, 1.91)
1.38 (0.84, 2.23)
Erl+Bev vs Pem+Nin
0.93 (0.66, 1.32)
0.96 (0.58, 1.59)
Erl+Bev vs Pembrolizumab
1.39 (0.97, 1.96)
1.39 (0.10, 17.94)
2.51 (1.40, 4.62)
Erl+Bev vs Pemetrexed
0.95 (0.72, 1.24)
0.98 (0.64, 1.47)
Erl+Bev vs Placebo
0.80 (0.61, 1.05)
0.61 (0.07, 5.00)
0.78 (0.52, 1.15)
Erl+Bev vs Pralatrexate
1.16 (0.77, 1.74)
0.86 (0.10, 7.26)
1.65 (0.93, 2.95)
Erl+Bev vs Teg/ura+Gef
1.16 (0.65, 2.10)
1.16 (0.58, 2.30)
Erl+Bev vs Trametinib
1.02 (0.49, 2.13)
1.66 (0.73, 3.86)
Erl+Bev vs Vandetanib
0.93 (0.70, 1.22)
0.73 (0.09, 5.97)
1.08 (0.71, 1.66)
Total population
Only squamous population
Only non-squamous population
.2
.3
.5
1
2
5

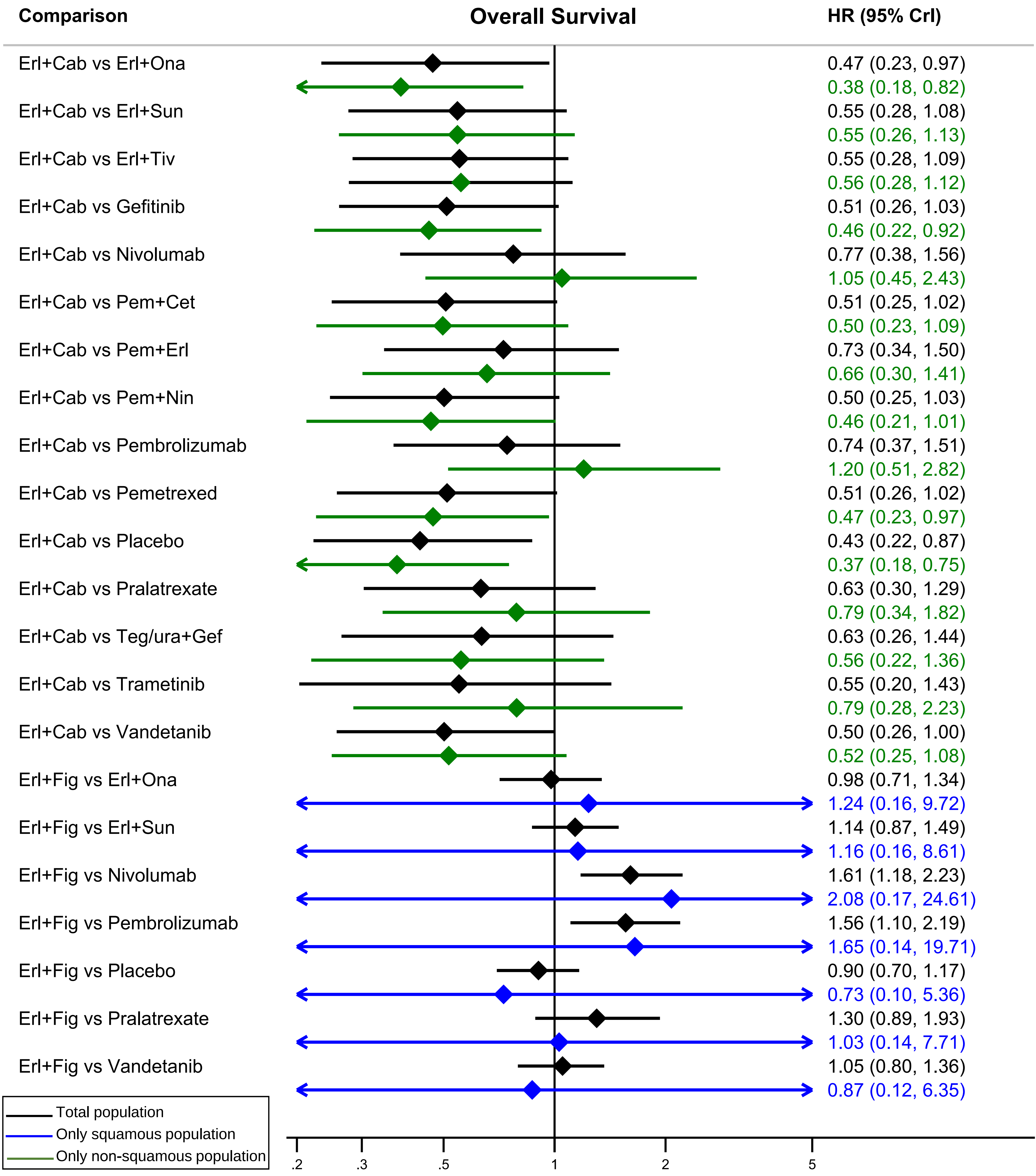

Comparison
Overall Survival
HR (95% CrI)
Erl+Cab vs Erl+Ona
0.47 (0.23, 0.97)
0.38 (0.18, 0.82)
Erl+Cab vs Erl+Sun
0.55 (0.28, 1.08)
0.55 (0.26, 1.13)
Erl+Cab vs Erl+Tiv
0.55 (0.28, 1.09)
0.56 (0.28, 1.12)
Erl+Cab vs Gefitinib
0.51 (0.26, 1.03)
0.46 (0.22, 0.92)
Erl+Cab vs Nivolumab
0.77 (0.38, 1.56)
1.05 (0.45, 2.43)
Erl+Cab vs Pem+Cet
0.51 (0.25, 1.02)
0.50 (0.23, 1.09)
Erl+Cab vs Pem+Erl
0.73 (0.34, 1.50)
0.66 (0.30, 1.41)
Erl+Cab vs Pem+Nin
0.50 (0.25, 1.03)
0.46 (0.21, 1.01)
Erl+Cab vs Pembrolizumab
0.74 (0.37, 1.51)
1.20 (0.51, 2.82)
Erl+Cab vs Pemetrexed
0.51 (0.26, 1.02)
0.47 (0.23, 0.97)
Erl+Cab vs Placebo
0.43 (0.22, 0.87)
0.37 (0.18, 0.75)
Erl+Cab vs Pralatrexate
0.63 (0.30, 1.29)
0.79 (0.34, 1.82)
Erl+Cab vs Teg/ura+Gef
0.63 (0.26, 1.44)
0.56 (0.22, 1.36)
Erl+Cab vs Trametinib
0.55 (0.20, 1.43)
0.79 (0.28, 2.23)
Erl+Cab vs Vandetanib
0.50 (0.26, 1.00)
0.52 (0.25, 1.08)
Erl+Fig vs Erl+Ona
0.98 (0.71, 1.34)
1.24 (0.16, 9.72)
Erl+Fig vs Erl+Sun
1.14 (0.87, 1.49)
1.16 (0.16, 8.61)
Erl+Fig vs Nivolumab
1.61 (1.18, 2.23)
2.08 (0.17, 24.61)
Erl+Fig vs Pembrolizumab
1.56 (1.10, 2.19)
1.65 (0.14, 19.71)
Erl+Fig vs Placebo
0.90 (0.70, 1.17)
0.73 (0.10, 5.36)
Erl+Fig vs Pralatrexate
1.30 (0.89, 1.93)
1.03 (0.14, 7.71)
Erl+Fig vs Vandetanib
1.05 (0.80, 1.36)
0.87 (0.12, 6.35)
Total population
Only squamous population
Only non-squamous population
.2
.3
.5
1
2
5

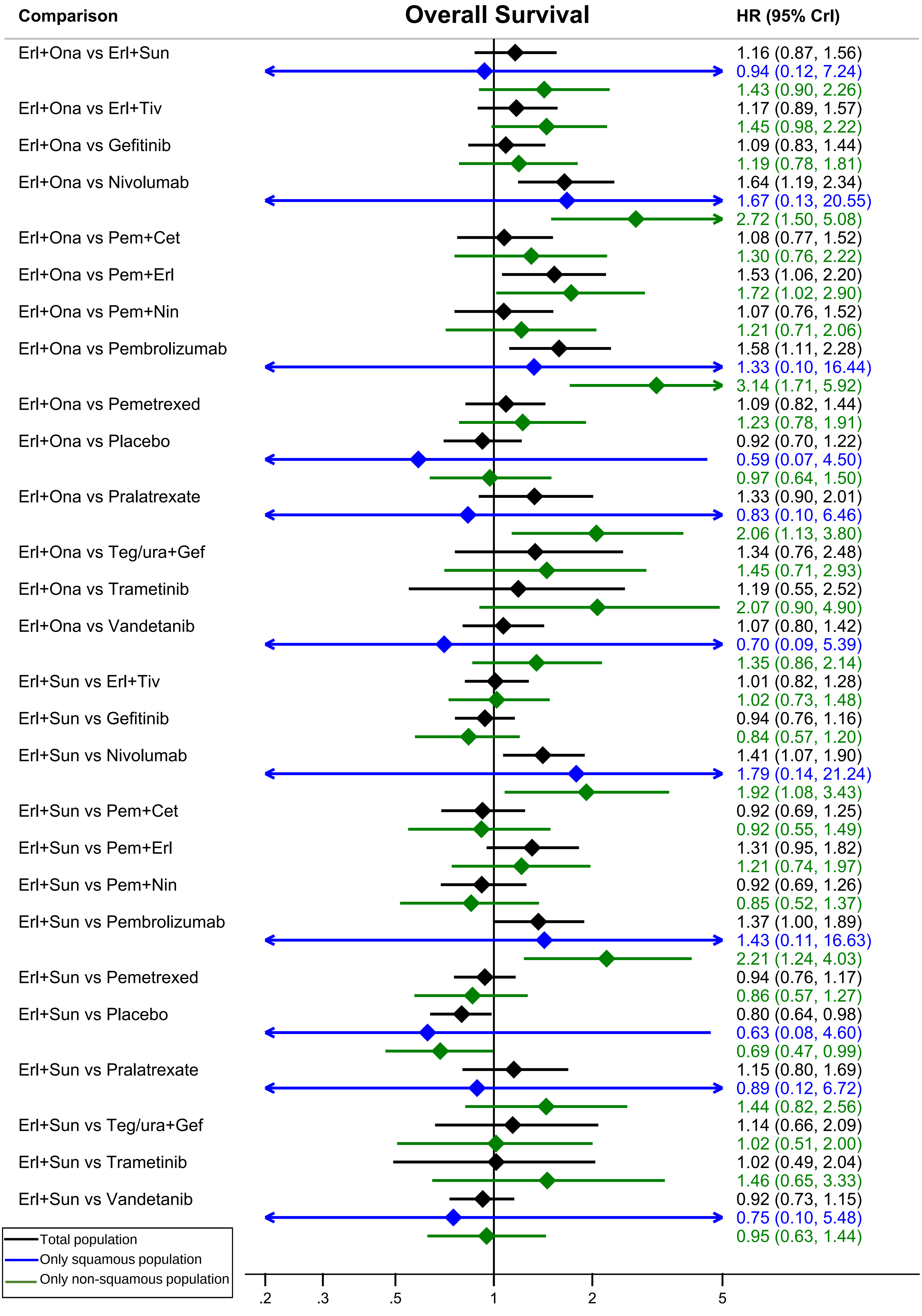
Comparison
Overall Survival
HR (95% CrI)
Erl+Ona vs Erl+Sun
1.16 (0.87, 1.56)
0.94 (0.12, 7.24)
1.43 (0.90, 2.26)
Erl+Ona vs Erl+Tiv
1.17 (0.89, 1.57)
1.45 (0.98, 2.22)
Erl+Ona vs Gefitinib
1.09 (0.83, 1.44)
1.19 (0.78, 1.81)
Erl+Ona vs Nivolumab
1.64 (1.19, 2.34)
1.67 (0.13, 20.55)
2.72 (1.50, 5.08)
Erl+Ona vs Pem+Cet
1.08 (0.77, 1.52)
1.30 (0.76, 2.22)
Erl+Ona vs Pem+Erl
1.53 (1.06, 2.20)
1.72 (1.02, 2.90)
Erl+Ona vs Pem+Nin
1.07 (0.76, 1.52)
1.21 (0.71, 2.06)
Erl+Ona vs Pembrolizumab
1.58 (1.11, 2.28)
1.33 (0.10, 16.44)
3.14 (1.71, 5.92)
Erl+Ona vs Pemetrexed
1.09 (0.82, 1.44)
1.23 (0.78, 1.91)
Erl+Ona vs Placebo
0.92 (0.70, 1.22)
0.59 (0.07, 4.50)
0.97 (0.64, 1.50)
Erl+Ona vs Pralatrexate
1.33 (0.90, 2.01)
0.83 (0.10, 6.46)
2.06 (1.13, 3.80)
Erl+Ona vs Teg/ura+Gef
1.34 (0.76, 2.48)
1.45 (0.71, 2.93)
Erl+Ona vs Trametinib
1.19 (0.55, 2.52)
2.07 (0.90, 4.90)
Erl+Ona vs Vandetanib
1.07 (0.80, 1.42)
0.70 (0.09, 5.39)
1.35 (0.86, 2.14)
Erl+Sun vs Erl+Tiv
1.01 (0.82, 1.28)
1.02 (0.73, 1.48)
Erl+Sun vs Gefitinib
0.94 (0.76, 1.16)
0.84 (0.57, 1.20)
Erl+Sun vs Nivolumab
1.41 (1.07, 1.90)
1.79 (0.14, 21.24)
1.92 (1.08, 3.43)
Erl+Sun vs Pem+Cet
0.92 (0.69, 1.25)
0.92 (0.55, 1.49)
Erl+Sun vs Pem+Erl
1.31 (0.95, 1.82)
1.21 (0.74, 1.97)
Erl+Sun vs Pem+Nin
0.92 (0.69, 1.26)
0.85 (0.52, 1.37)
Erl+Sun vs Pembrolizumab
1.37 (1.00, 1.89)
1.43 (0.11, 16.63)
2.21 (1.24, 4.03)
Erl+Sun vs Pemetrexed
0.94 (0.76, 1.17)
0.86 (0.57, 1.27)
Erl+Sun vs Placebo
0.80 (0.64, 0.98)
0.63 (0.08, 4.60)
0.69 (0.47, 0.99)
Erl+Sun vs Pralatrexate
1.15 (0.80, 1.69)
0.89 (0.12, 6.72)
1.44 (0.82, 2.56)
Erl+Sun vs Teg/ura+Gef
1.14 (0.66, 2.09)
1.02 (0.51, 2.00)
Erl+Sun vs Trametinib
1.02 (0.49, 2.04)
1.46 (0.65, 3.33)
Erl+Sun vs Vandetanib
0.92 (0.73, 1.15)
0.75 (0.10, 5.48)
0.95 (0.63, 1.44)
Total population
Only squamous population
Only non-squamous population
.2
.3
.5
1
2
5

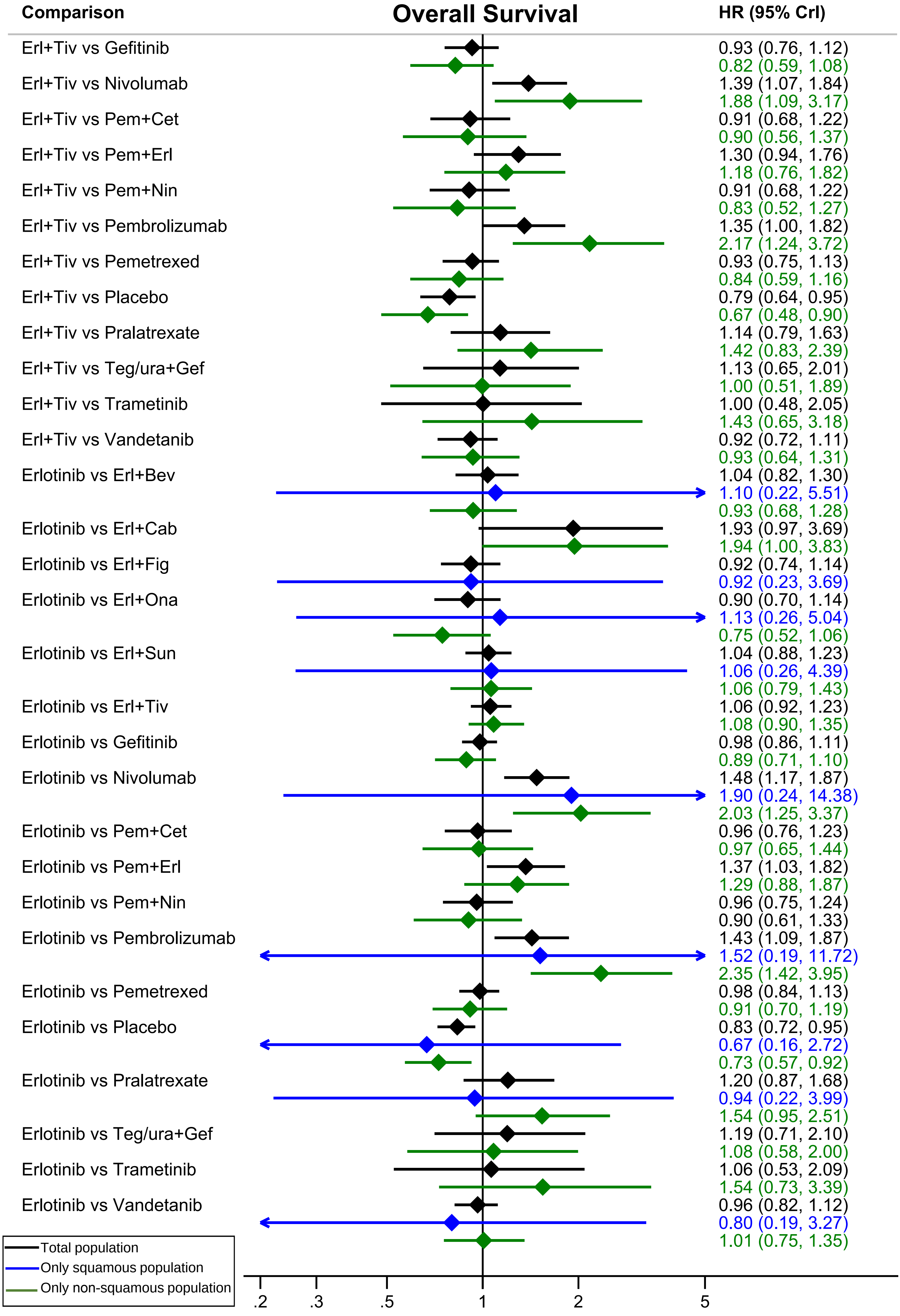
Comparison
Overall Survival
HR (95% CrI)
Erl+Tiv vs Gefitinib
0.93 (0.76, 1.12)
0.82 (0.59, 1.08)
Erl+Tiv vs Nivolumab
1.39 (1.07, 1.84)
1.88 (1.09, 3.17)
Erl+Tiv vs Pem+Cet
0.91 (0.68, 1.22)
0.90 (0.56, 1.37)
Erl+Tiv vs Pem+Erl
1.30 (0.94, 1.76)
1.18 (0.76, 1.82)
Erl+Tiv vs Pem+Nin
0.91 (0.68, 1.22)
0.83 (0.52, 1.27)
Erl+Tiv vs Pembrolizumab
1.35 (1.00, 1.82)
2.17 (1.24, 3.72)
Erl+Tiv vs Pemetrexed
0.93 (0.75, 1.13)
0.84 (0.59, 1.16)
Erl+Tiv vs Placebo
0.79 (0.64, 0.95)
0.67 (0.48, 0.90)
Erl+Tiv vs Pralatrexate
1.14 (0.79, 1.63)
1.42 (0.83, 2.39)
Erl+Tiv vs Teg/ura+Gef
1.13 (0.65, 2.01)
1.00 (0.51, 1.89)
Erl+Tiv vs Trametinib
1.00 (0.48, 2.05)
1.43 (0.65, 3.18)
Erl+Tiv vs Vandetanib
0.92 (0.72, 1.11)
0.93 (0.64, 1.31)
Erlotinib vs Erl+Bev
1.04 (0.82, 1.30)
1.10 (0.22, 5.51)
0.93 (0.68, 1.28)
Erlotinib vs Erl+Cab
1.93 (0.97, 3.69)
1.94 (1.00, 3.83)
Erlotinib vs Erl+Fig
0.92 (0.74, 1.14)
0.92 (0.23, 3.69)
Erlotinib vs Erl+Ona
0.90 (0.70, 1.14)
1.13 (0.26, 5.04)
0.75 (0.52, 1.06)
Erlotinib vs Erl+Sun
1.04 (0.88, 1.23)
1.06 (0.26, 4.39)
1.06 (0.79, 1.43)
Erlotinib vs Erl+Tiv
1.06 (0.92, 1.23)
1.08 (0.90, 1.35)
Erlotinib vs Gefitinib
0.98 (0.86, 1.11)
0.89 (0.71, 1.10)
Erlotinib vs Nivolumab
1.48 (1.17, 1.87)
1.90 (0.24, 14.38)
2.03 (1.25, 3.37)
Erlotinib vs Pem+Cet
0.96 (0.76, 1.23)
0.97 (0.65, 1.44)
Erlotinib vs Pem+Erl
1.37 (1.03, 1.82)
1.29 (0.88, 1.87)
Erlotinib vs Pem+Nin
0.96 (0.75, 1.24)
0.90 (0.61, 1.33)
Erlotinib vs Pembrolizumab
1.43 (1.09, 1.87)
1.52 (0.19, 11.72)
2.35 (1.42, 3.95)
Erlotinib vs Pemetrexed
0.98 (0.84, 1.13)
0.91 (0.70, 1.19)
Erlotinib vs Placebo
0.83 (0.72, 0.95)
0.67 (0.16, 2.72)
0.73 (0.57, 0.92)
Erlotinib vs Pralatrexate
1.20 (0.87, 1.68)
0.94 (0.22, 3.99)
1.54 (0.95, 2.51)
Erlotinib vs Teg/ura+Gef
1.19 (0.71, 2.10)
1.08 (0.58, 2.00)
Erlotinib vs Trametinib
1.06 (0.53, 2.09)
1.54 (0.73, 3.39)
Erlotinib vs Vandetanib
0.96 (0.82, 1.12)
0.80 (0.19, 3.27)
1.01 (0.75, 1.35)
Total population
Only squamous population
Only non-squamous population
.2
.3
.5
1
2
5

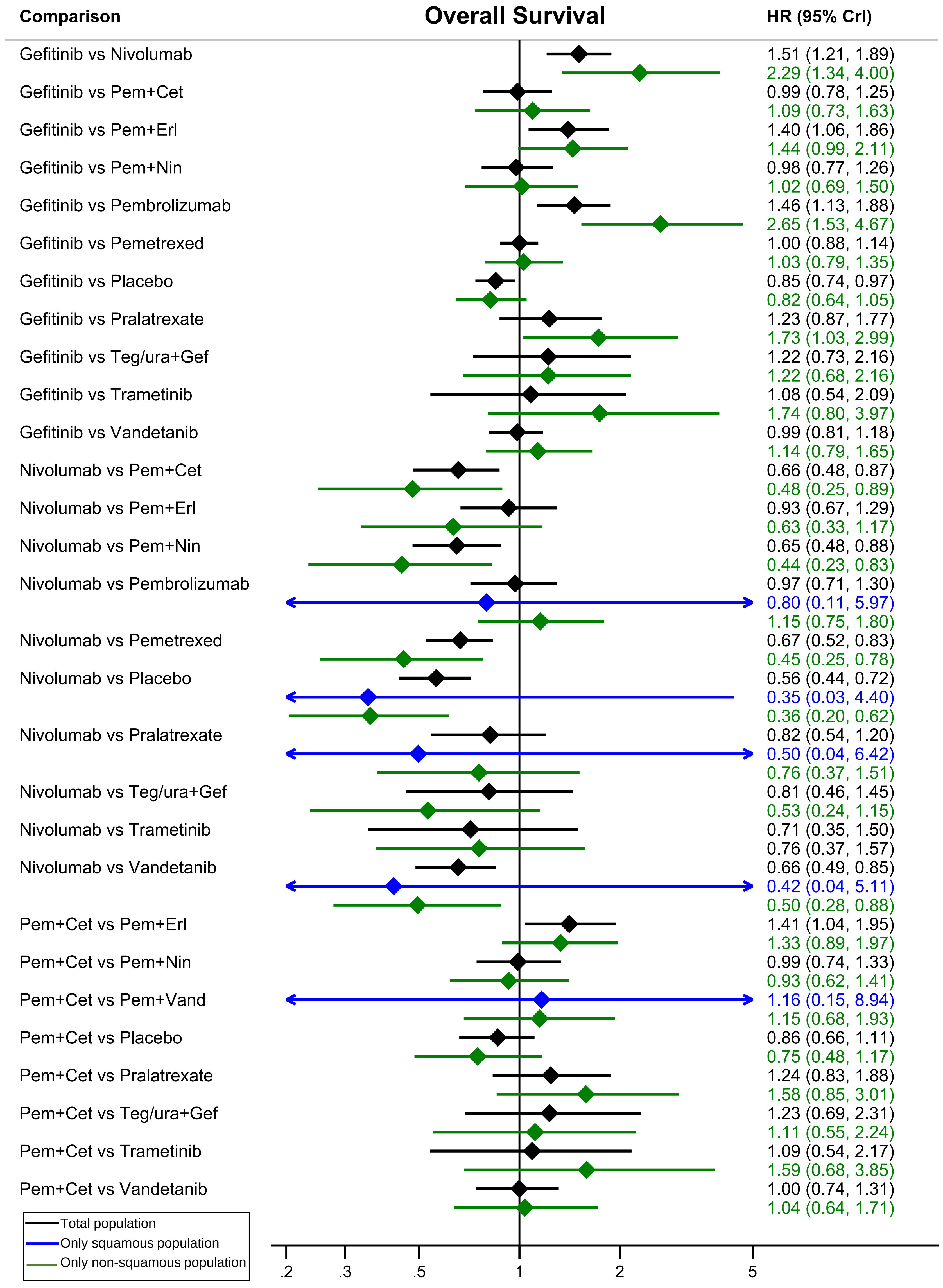
Comparison
Overall Survival
HR (95% CrI)
Gefitinib vs Nivolumab
1.51 (1.21, 1.89)
2.29 (1.34, 4.00)
Gefitinib vs Pem+Cet
0.99 (0.78, 1.25)
1.09 (0.73, 1.63)
Gefitinib vs Pem+Erl
1.40 (1.06, 1.86)
1.44 (0.99, 2.11)
Gefitinib vs Pem+Nin
0.98 (0.77, 1.26)
1.02 (0.69, 1.50)
Gefitinib vs Pembrolizumab
1.46 (1.13, 1.88)
2.65 (1.53, 4.67)
Gefitinib vs Pemetrexed
1.00 (0.88, 1.14)
1.03 (0.79, 1.35)
Gefitinib vs Placebo
0.85 (0.74, 0.97)
0.82 (0.64, 1.05)
Gefitinib vs Pralatrexate
1.23 (0.87, 1.77)
1.73 (1.03, 2.99)
Gefitinib vs Teg/ura+Gef
1.22 (0.73, 2.16)
1.22 (0.68, 2.16)
Gefitinib vs Trametinib
1.08 (0.54, 2.09)
1.74 (0.80, 3.97)
Gefitinib vs Vandetanib
0.99 (0.81, 1.18)
1.14 (0.79, 1.65)
Nivolumab vs Pem+Cet
0.66 (0.48, 0.87)
0.48 (0.25, 0.89)
Nivolumab vs Pem+Erl
0.93 (0.67, 1.29)
0.63 (0.33, 1.17)
Nivolumab vs Pem+Nin
0.65 (0.48, 0.88)
0.44 (0.23, 0.83)
Nivolumab vs Pembrolizumab
0.97 (0.71, 1.30)
0.80 (0.11, 5.97)
1.15 (0.75, 1.80)
Nivolumab vs Pemetrexed
0.67 (0.52, 0.83)
0.45 (0.25, 0.78)
Nivolumab vs Placebo
0.56 (0.44, 0.72)
0.35 (0.03, 4.40)
0.36 (0.20, 0.62)
Nivolumab vs Pralatrexate
0.82 (0.54, 1.20)
0.50 (0.04, 6.42)
0.76 (0.37, 1.51)
Nivolumab vs Teg/ura+Gef
0.81 (0.46, 1.45)
0.53 (0.24, 1.15)
Nivolumab vs Trametinib
0.71 (0.35, 1.50)
0.76 (0.37, 1.57)
Nivolumab vs Vandetanib
0.66 (0.49, 0.85)
0.42 (0.04, 5.11)
0.50 (0.28, 0.88)
Pem+Cet vs Pem+Erl
1.41 (1.04, 1.95)
1.33 (0.89, 1.97)
Pem+Cet vs Pem+Nin
0.99 (0.74, 1.33)
0.93 (0.62, 1.41)
Pem+Cet vs Pem+Vand
1.16 (0.15, 8.94)
1.15 (0.68, 1.93)
Pem+Cet vs Placebo
0.86 (0.66, 1.11)
0.75 (0.48, 1.17)
Pem+Cet vs Pralatrexate
1.24 (0.83, 1.88)
1.58 (0.85, 3.01)
Pem+Cet vs Teg/ura+Gef
1.23 (0.69, 2.31)
1.11 (0.55, 2.24)
Pem+Cet vs Trametinib
1.09 (0.54, 2.17)
1.59 (0.68, 3.85)
Pem+Cet vs Vandetanib
1.00 (0.74, 1.31)
1.04 (0.64, 1.71)
Total population
Only squamous population
Only non-squamous population
.2
.3
.5
1
2
5

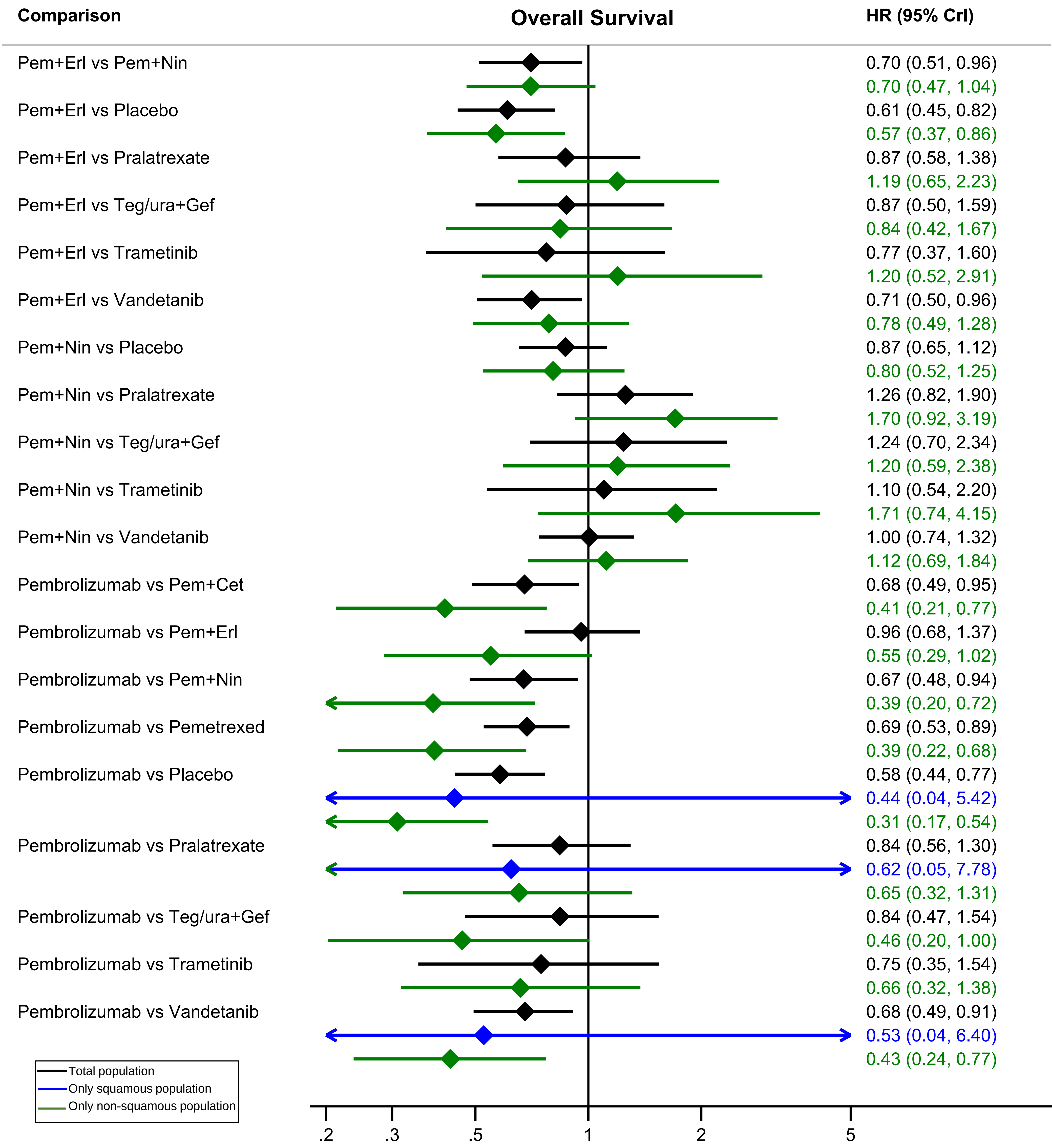

Comparison
Overall Survival
HR (95% CrI)
Pem+Erl vs Pem+Nin
0.70 (0.51, 0.96)
0.70 (0.47, 1.04)
Pem+Erl vs Placebo
0.61 (0.45, 0.82)
0.57 (0.37, 0.86)
Pem+Erl vs Pralatrexate
0.87 (0.58, 1.38)
1.19 (0.65, 2.23)
Pem+Erl vs Teg/ura+Gef
0.87 (0.50, 1.59)
0.84 (0.42, 1.67)
Pem+Erl vs Trametinib
0.77 (0.37, 1.60)
1.20 (0.52, 2.91)
Pem+Erl vs Vandetanib
0.71 (0.50, 0.96)
0.78 (0.49, 1.28)
Pem+Nin vs Placebo
0.87 (0.65, 1.12)
0.80 (0.52, 1.25)
Pem+Nin vs Pralatrexate
1.26 (0.82, 1.90)
1.70 (0.92, 3.19)
Pem+Nin vs Teg/ura+Gef
1.24 (0.70, 2.34)
1.20 (0.59, 2.38)
Pem+Nin vs Trametinib
1.10 (0.54, 2.20)
1.71 (0.74, 4.15)
Pem+Nin vs Vandetanib
1.00 (0.74, 1.32)
1.12 (0.69, 1.84)
Pembrolizumab vs Pem+Cet
0.68 (0.49, 0.95)
0.41 (0.21, 0.77)
Pembrolizumab vs Pem+Erl
0.96 (0.68, 1.37)
0.55 (0.29, 1.02)
Pembrolizumab vs Pem+Nin
0.67 (0.48, 0.94)
0.39 (0.20, 0.72)
Pembrolizumab vs Pemetrexed
0.69 (0.53, 0.89)
0.39 (0.22, 0.68)
Pembrolizumab vs Placebo
0.58 (0.44, 0.77)
0.44 (0.04, 5.42)
0.31 (0.17, 0.54)
Pembrolizumab vs Pralatrexate
0.84 (0.56, 1.30)
0.62 (0.05, 7.78)
0.65 (0.32, 1.31)
Pembrolizumab vs Teg/ura+Gef
0.84 (0.47, 1.54)
0.46 (0.20, 1.00)
Pembrolizumab vs Trametinib
0.75 (0.35, 1.54)
0.66 (0.32, 1.38)
Pembrolizumab vs Vandetanib
0.68 (0.49, 0.91)
0.53 (0.04, 6.40)
0.43 (0.24, 0.77)
Total population
Only squamous population
Only non-squamous population
.2
.3
.5
1
2
5

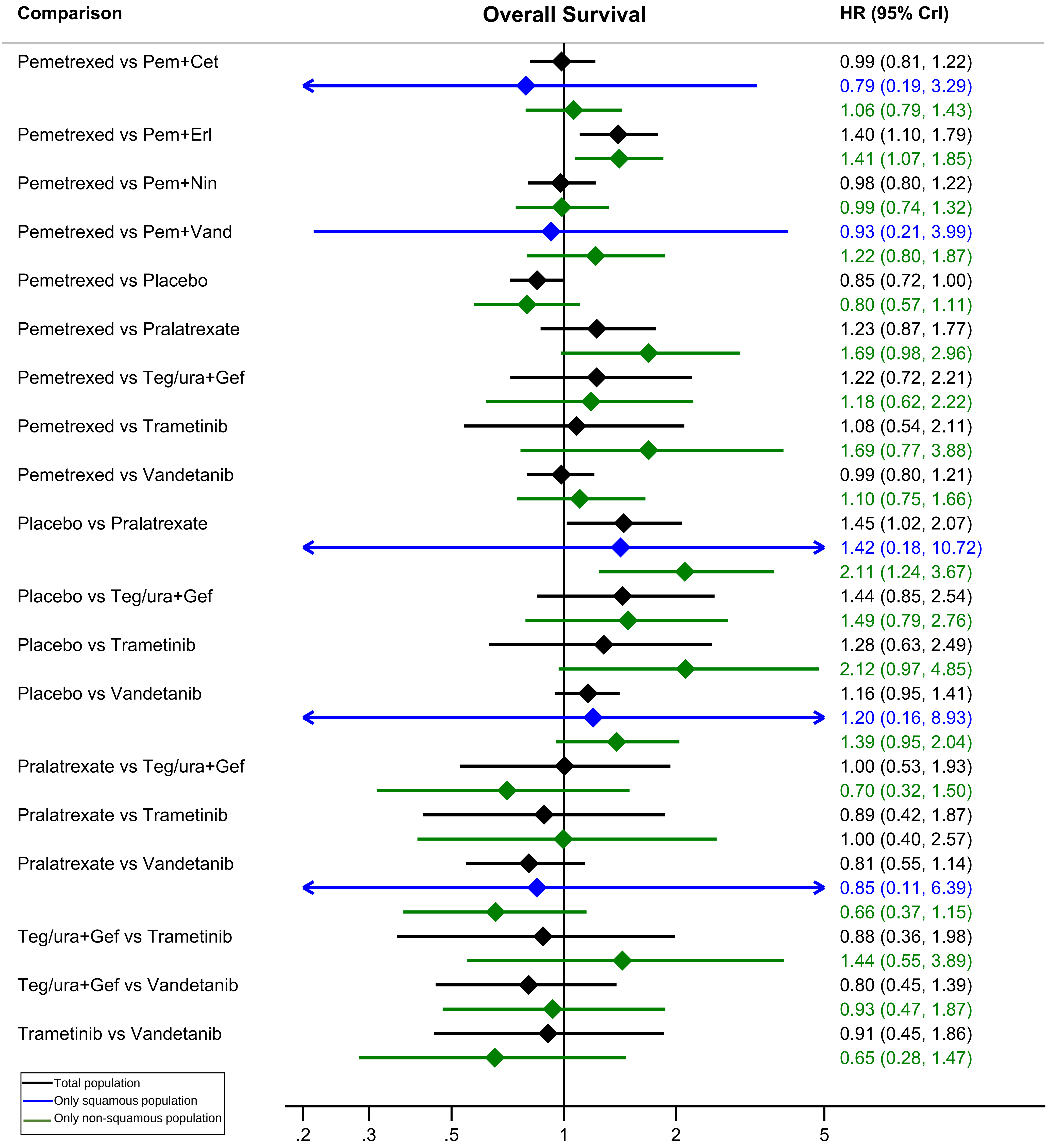
Comparison
Overall Survival
HR (95% CrI)
Pemetrexed vs Pem+Cet
0.99 (0.81, 1.22)
0.79 (0.19, 3.29)
1.06 (0.79, 1.43)
Pemetrexed vs Pem+Erl
1.40 (1.10, 1.79)
1.41 (1.07, 1.85)
Pemetrexed vs Pem+Nin
0.98 (0.80, 1.22)
0.99 (0.74, 1.32)
Pemetrexed vs Pem+Vand
0.93 (0.21, 3.99)
1.22 (0.80, 1.87)
Pemetrexed vs Placebo
0.85 (0.72, 1.00)
0.80 (0.57, 1.11)
Pemetrexed vs Pralatrexate
1.23 (0.87, 1.77)
1.69 (0.98, 2.96)
Pemetrexed vs Teg/ura+Gef
1.22 (0.72, 2.21)
1.18 (0.62, 2.22)
Pemetrexed vs Trametinib
1.08 (0.54, 2.11)
1.69 (0.77, 3.88)
Pemetrexed vs Vandetanib
0.99 (0.80, 1.21)
1.10 (0.75, 1.66)
Placebo vs Pralatrexate
1.45 (1.02, 2.07)
1.42 (0.18, 10.72)
2.11 (1.24, 3.67)
Placebo vs Teg/ura+Gef
1.44 (0.85, 2.54)
1.49 (0.79, 2.76)
Placebo vs Trametinib
1.28 (0.63, 2.49)
2.12 (0.97, 4.85)
Placebo vs Vandetanib
1.16 (0.95, 1.41)
1.20 (0.16, 8.93)
1.39 (0.95, 2.04)
Pralatrexate vs Teg/ura+Gef
1.00 (0.53, 1.93)
0.70 (0.32, 1.50)
Pralatrexate vs Trametinib
0.89 (0.42, 1.87)
1.00 (0.40, 2.57)
Pralatrexate vs Vandetanib
0.81 (0.55, 1.14)
0.85 (0.11, 6.39)
0.66 (0.37, 1.15)
Teg/ura+Gef vs Trametinib
0.88 (0.36, 1.98)
1.44 (0.55, 3.89)
Teg/ura+Gef vs Vandetanib
0.80 (0.45, 1.39)
0.93 (0.47, 1.87)
Trametinib vs Vandetanib
0.91 (0.45, 1.86)
0.65 (0.28, 1.47)
Total population
Only squamous population
Only non-squamous population
.2
.3
.5
1
2
5

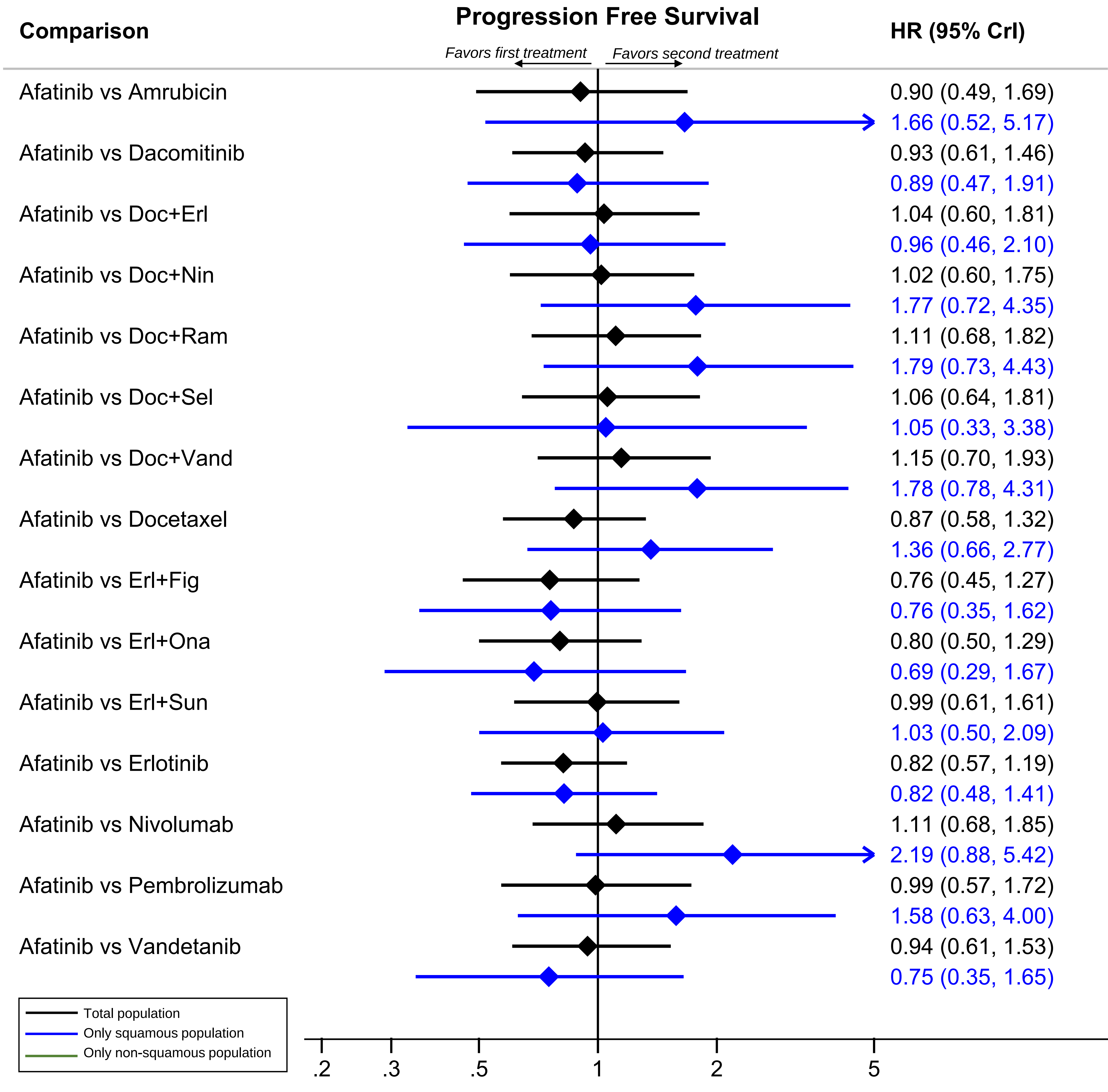

Progression Free Survival
Comparison
HR (95% CrI)
Favors first treatment
Favors second treatment
Afatinib vs Amrubicin
0.90 (0.49, 1.69)
1.66 (0.52, 5.17)
Afatinib vs Dacomitinib
0.93 (0.61, 1.46)
0.89 (0.47, 1.91)
Afatinib vs Doc+Erl
1.04 (0.60, 1.81)
0.96 (0.46, 2.10)
Afatinib vs Doc+Nin
1.02 (0.60, 1.75)
1.77 (0.72, 4.35)
Afatinib vs Doc+Ram
1.11 (0.68, 1.82)
1.79 (0.73, 4.43)
Afatinib vs Doc+Sel
1.06 (0.64, 1.81)
1.05 (0.33, 3.38)
Afatinib vs Doc+Vand
1.15 (0.70, 1.93)
1.78 (0.78, 4.31)
Afatinib vs Docetaxel
0.87 (0.58, 1.32)
1.36 (0.66, 2.77)
Afatinib vs Erl+Fig
0.76 (0.45, 1.27)
0.76 (0.35, 1.62)
Afatinib vs Erl+Ona
0.80 (0.50, 1.29)
0.69 (0.29, 1.67)
Afatinib vs Erl+Sun
0.99 (0.61, 1.61)
1.03 (0.50, 2.09)
Afatinib vs Erlotinib
0.82 (0.57, 1.19)
0.82 (0.48, 1.41)
Afatinib vs Nivolumab
1.11 (0.68, 1.85)
2.19 (0.88, 5.42)
Afatinib vs Pembrolizumab
0.99 (0.57, 1.72)
1.58 (0.63, 4.00)
Afatinib vs Vandetanib
0.94 (0.61, 1.53)
0.75 (0.35, 1.65)
Total population
Only squamous population
Only non-squamous population
.2
.3
.5
1
2
5

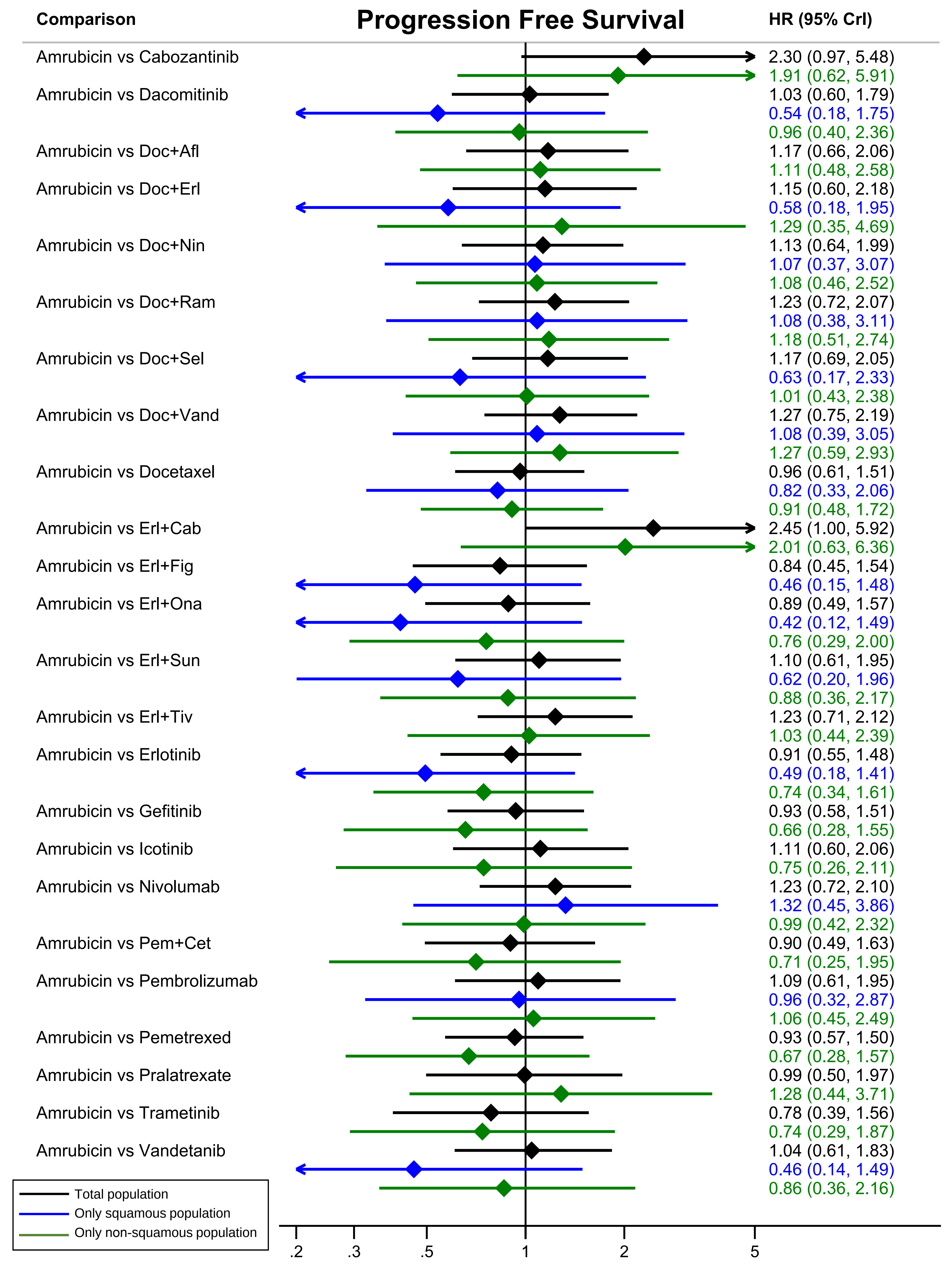
Comparison
Progression Free Survival
HR (95% CrI)
Amrubicin vs Cabozantinib
2.30 (0.97, 5.48)
1.91 (0.62, 5.91)
Amrubicin vs Dacomitinib
1.03 (0.60, 1.79)
0.54 (0.18, 1.75)
0.96 (0.40, 2.36)
Amrubicin vs Doc+Afl
1.17 (0.66, 2.06)
1.11 (0.48, 2.58)
Amrubicin vs Doc+Erl
1.15 (0.60, 2.18)
0.58 (0.18, 1.95)
1.29 (0.35, 4.69)
Amrubicin vs Doc+Nin
1.13 (0.64, 1.99)
1.07 (0.37, 3.07)
1.08 (0.46, 2.52)
Amrubicin vs Doc+Ram
1.23 (0.72, 2.07)
1.08 (0.38, 3.11)
1.18 (0.51, 2.74)
Amrubicin vs Doc+Sel
1.17 (0.69, 2.05)
0.63 (0.17, 2.33)
1.01 (0.43, 2.38)
Amrubicin vs Doc+Vand
1.27 (0.75, 2.19)
1.08 (0.39, 3.05)
1.27 (0.59, 2.93)
Amrubicin vs Docetaxel
0.96 (0.61, 1.51)
0.82 (0.33, 2.06)
0.91 (0.48, 1.72)
Amrubicin vs Erl+Cab
2.45 (1.00, 5.92)
2.01 (0.63, 6.36)
Amrubicin vs Erl+Fig
0.84 (0.45, 1.54)
0.46 (0.15, 1.48)
Amrubicin vs Erl+Ona
0.89 (0.49, 1.57)
0.42 (0.12, 1.49)
0.76 (0.29, 2.00)
Amrubicin vs Erl+Sun
1.10 (0.61, 1.95)
0.62 (0.20, 1.96)
0.88 (0.36, 2.17)
Amrubicin vs Erl+Tiv
1.23 (0.71, 2.12)
1.03 (0.44, 2.39)
Amrubicin vs Erlotinib
0.91 (0.55, 1.48)
0.49 (0.18, 1.41)
0.74 (0.34, 1.61)
Amrubicin vs Gefitinib
0.93 (0.58, 1.51)
0.66 (0.28, 1.55)
Amrubicin vs Icotinib
1.11 (0.60, 2.06)
0.75 (0.26, 2.11)
Amrubicin vs Nivolumab
1.23 (0.72, 2.10)
1.32 (0.45, 3.86)
0.99 (0.42, 2.32)
Amrubicin vs Pem+Cet
0.90 (0.49, 1.63)
0.71 (0.25, 1.95)
Amrubicin vs Pembrolizumab
1.09 (0.61, 1.95)
0.96 (0.32, 2.87)
1.06 (0.45, 2.49)
Amrubicin vs Pemetrexed
0.93 (0.57, 1.50)
0.67 (0.28, 1.57)
Amrubicin vs Pralatrexate
0.99 (0.50, 1.97)
1.28 (0.44, 3.71)
Amrubicin vs Trametinib
0.78 (0.39, 1.56)
0.74 (0.29, 1.87)
Amrubicin vs Vandetanib
1.04 (0.61, 1.83)
0.46 (0.14, 1.49)
0.86 (0.36, 2.16)
Total population
Only squamous population
Only non-squamous population
.2
.3
.5
1
2
5

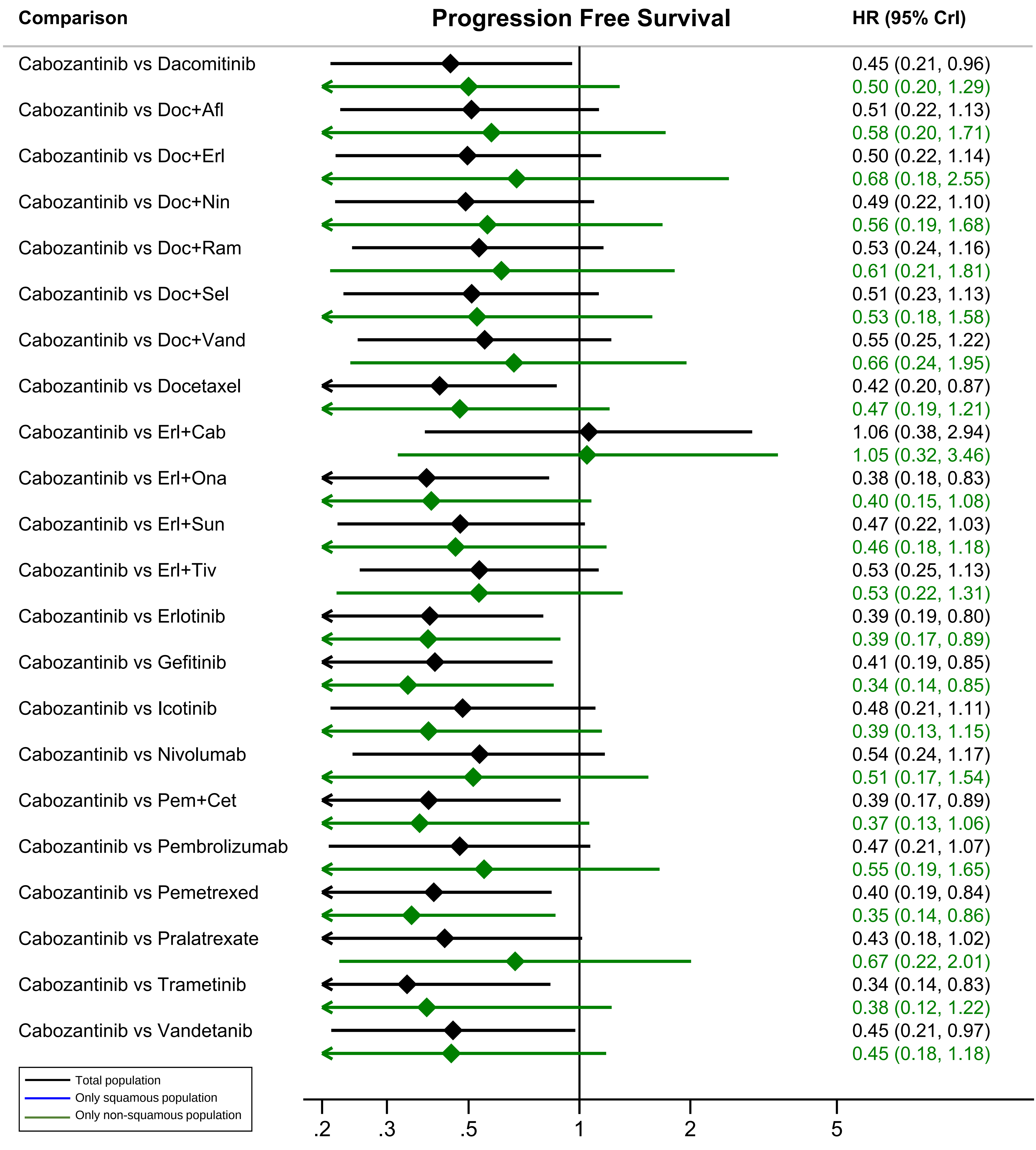
Comparison
Progression Free Survival
HR (95% CrI)
Cabozantinib vs Dacomitinib
0.45 (0.21, 0.96)
0.50 (0.20, 1.29)
Cabozantinib vs Doc+Afl
0.51 (0.22, 1.13)
0.58 (0.20, 1.71)
Cabozantinib vs Doc+Erl
0.50 (0.22, 1.14)
0.68 (0.18, 2.55)
Cabozantinib vs Doc+Nin
0.49 (0.22, 1.10)
0.56 (0.19, 1.68)
Cabozantinib vs Doc+Ram
0.53 (0.24, 1.16)
0.61 (0.21, 1.81)
Cabozantinib vs Doc+Sel
0.51 (0.23, 1.13)
0.53 (0.18, 1.58)
Cabozantinib vs Doc+Vand
0.55 (0.25, 1.22)
0.66 (0.24, 1.95)
Cabozantinib vs Docetaxel
0.42 (0.20, 0.87)
0.47 (0.19, 1.21)
Cabozantinib vs Erl+Cab
1.06 (0.38, 2.94)
1.05 (0.32, 3.46)
Cabozantinib vs Erl+Ona
0.38 (0.18, 0.83)
0.40 (0.15, 1.08)
Cabozantinib vs Erl+Sun
0.47 (0.22, 1.03)
0.46 (0.18, 1.18)
Cabozantinib vs Erl+Tiv
0.53 (0.25, 1.13)
0.53 (0.22, 1.31)
Cabozantinib vs Erlotinib
0.39 (0.19, 0.80)
0.39 (0.17, 0.89)
Cabozantinib vs Gefitinib
0.41 (0.19, 0.85)
0.34 (0.14, 0.85)
Cabozantinib vs Icotinib
0.48 (0.21, 1.11)
0.39 (0.13, 1.15)
Cabozantinib vs Nivolumab
0.54 (0.24, 1.17)
0.51 (0.17, 1.54)
Cabozantinib vs Pem+Cet
0.39 (0.17, 0.89)
0.37 (0.13, 1.06)
Cabozantinib vs Pembrolizumab
0.47 (0.21, 1.07)
0.55 (0.19, 1.65)
Cabozantinib vs Pemetrexed
0.40 (0.19, 0.84)
0.35 (0.14, 0.86)
Cabozantinib vs Pralatrexate
0.43 (0.18, 1.02)
0.67 (0.22, 2.01)
Cabozantinib vs Trametinib
0.34 (0.14, 0.83)
0.38 (0.12, 1.22)
Cabozantinib vs Vandetanib
0.45 (0.21, 0.97)
0.45 (0.18, 1.18)
Total population
Only squamous population
Only non-squamous population
.2
.3
.5
1
2
5

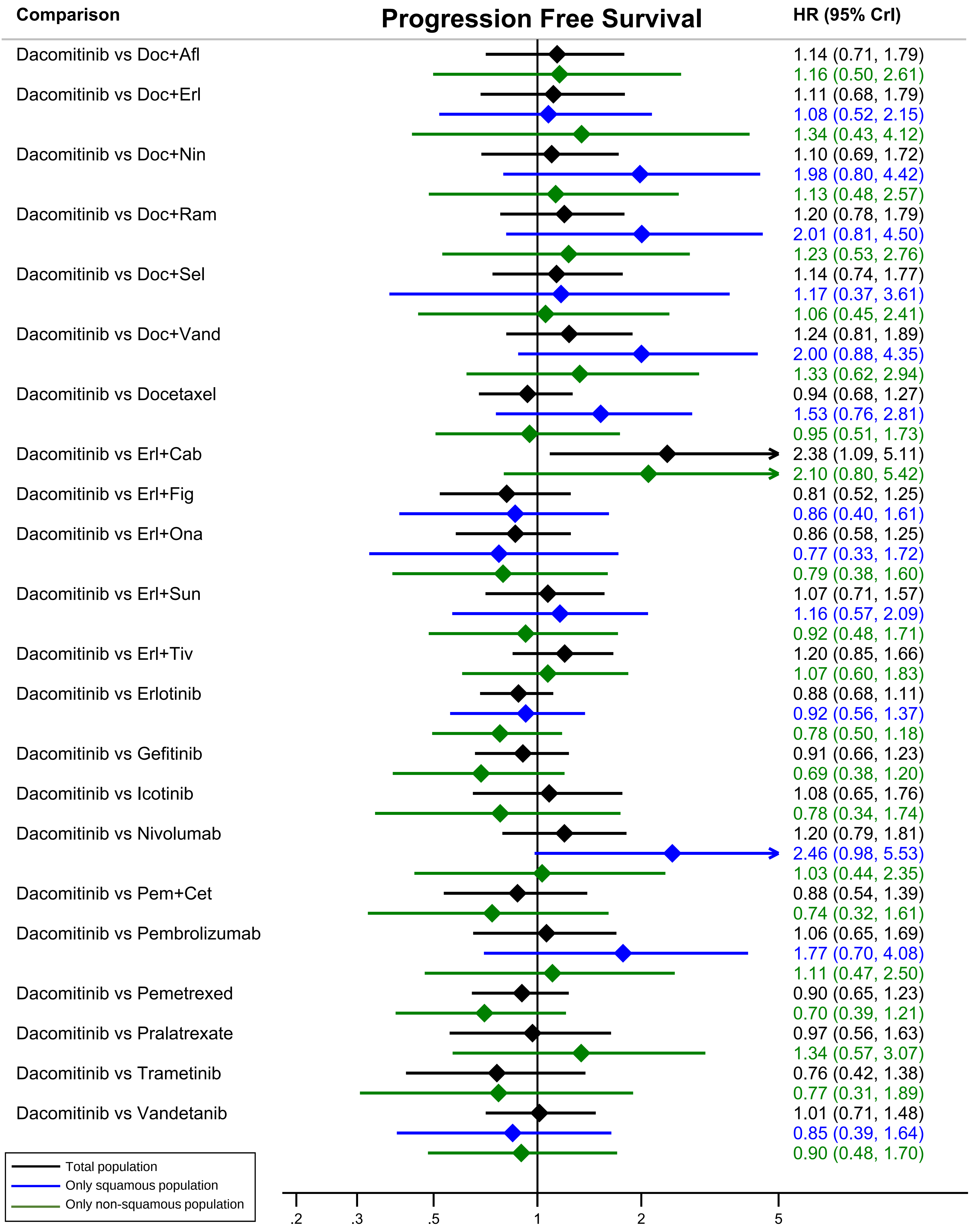
Comparison
Progression Free Survival
HR (95% CrI)
Dacomitinib vs Doc+Afl
1.14 (0.71, 1.79)
1.16 (0.50, 2.61)
Dacomitinib vs Doc+Erl
1.11 (0.68, 1.79)
1.08 (0.52, 2.15)
1.34 (0.43, 4.12)
Dacomitinib vs Doc+Nin
1.10 (0.69, 1.72)
1.98 (0.80, 4.42)
1.13 (0.48, 2.57)
Dacomitinib vs Doc+Ram
1.20 (0.78, 1.79)
2.01 (0.81, 4.50)
1.23 (0.53, 2.76)
Dacomitinib vs Doc+Sel
1.14 (0.74, 1.77)
1.17 (0.37, 3.61)
1.06 (0.45, 2.41)
Dacomitinib vs Doc+Vand
1.24 (0.81, 1.89)
2.00 (0.88, 4.35)
1.33 (0.62, 2.94)
Dacomitinib vs Docetaxel
0.94 (0.68, 1.27)
1.53 (0.76, 2.81)
0.95 (0.51, 1.73)
Dacomitinib vs Erl+Cab
2.38 (1.09, 5.11)
2.10 (0.80, 5.42)
Dacomitinib vs Erl+Fig
0.81 (0.52, 1.25)
0.86 (0.40, 1.61)
Dacomitinib vs Erl+Ona
0.86 (0.58, 1.25)
0.77 (0.33, 1.72)
0.79 (0.38, 1.60)
Dacomitinib vs Erl+Sun
1.07 (0.71, 1.57)
1.16 (0.57, 2.09)
0.92 (0.48, 1.71)
Dacomitinib vs Erl+Tiv
1.20 (0.85, 1.66)
1.07 (0.60, 1.83)
Dacomitinib vs Erlotinib
0.88 (0.68, 1.11)
0.92 (0.56, 1.37)
0.78 (0.50, 1.18)
Dacomitinib vs Gefitinib
0.91 (0.66, 1.23)
0.69 (0.38, 1.20)
Dacomitinib vs Icotinib
1.08 (0.65, 1.76)
0.78 (0.34, 1.74)
Dacomitinib vs Nivolumab
1.20 (0.79, 1.81)
2.46 (0.98, 5.53)
1.03 (0.44, 2.35)
Dacomitinib vs Pem+Cet
0.88 (0.54, 1.39)
0.74 (0.32, 1.61)
Dacomitinib vs Pembrolizumab
1.06 (0.65, 1.69)
1.77 (0.70, 4.08)
1.11 (0.47, 2.50)
Dacomitinib vs Pemetrexed
0.90 (0.65, 1.23)
0.70 (0.39, 1.21)
Dacomitinib vs Pralatrexate
0.97 (0.56, 1.63)
1.34 (0.57, 3.07)
Dacomitinib vs Trametinib
0.76 (0.42, 1.38)
0.77 (0.31, 1.89)
Dacomitinib vs Vandetanib
1.01 (0.71, 1.48)
0.85 (0.39, 1.64)
0.90 (0.48, 1.70)
Total population
Only squamous population
Only non-squamous population
.2
.3
.5
1
2
5

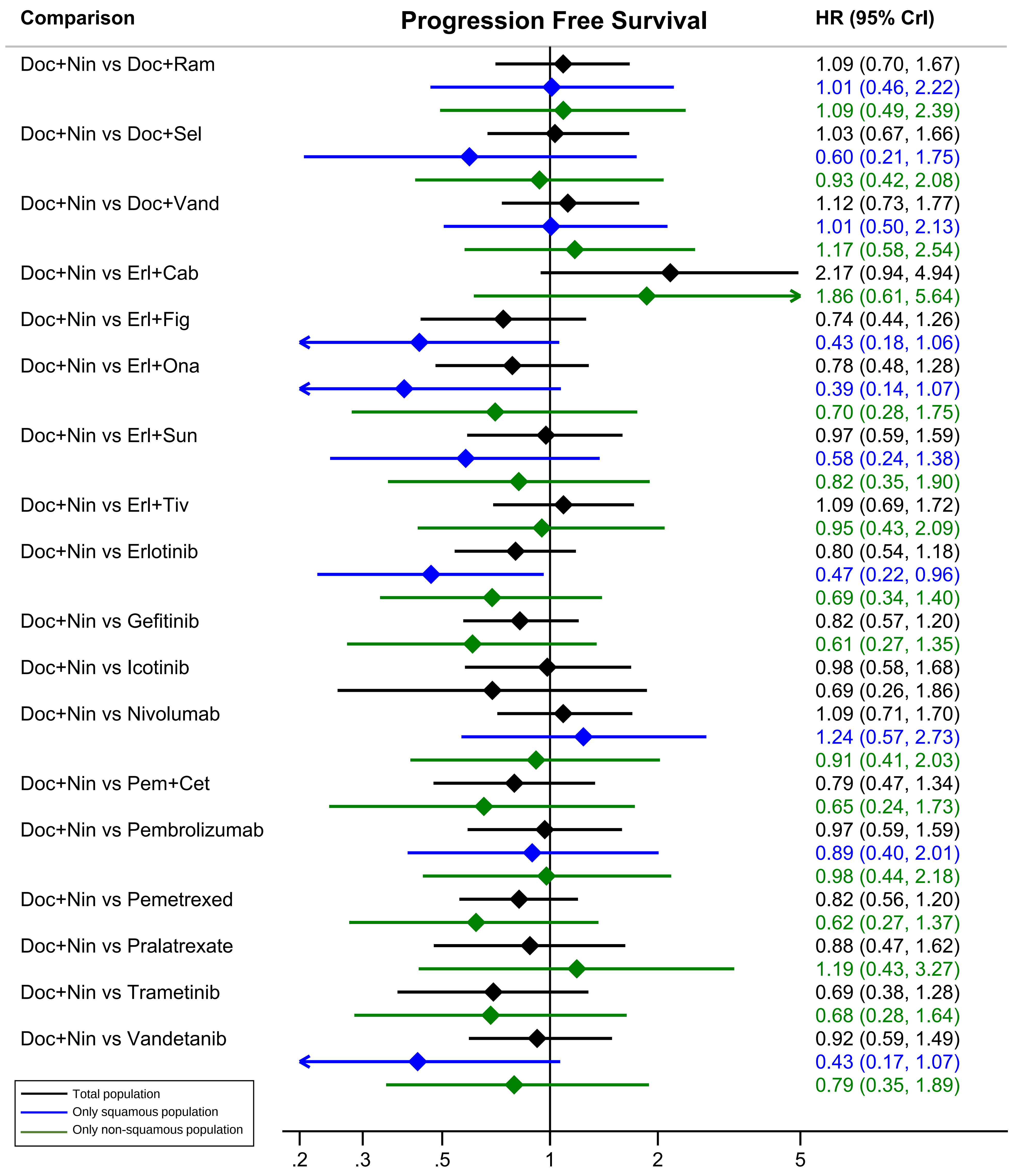
Comparison
Progression Free Survival
HR (95% CrI)
Doc+Nin vs Doc+Ram
1.09 (0.70, 1.67)
1.01 (0.46, 2.22)
1.09 (0.49, 2.39)
Doc+Nin vs Doc+Sel
1.03 (0.67, 1.66)
0.60 (0.21, 1.75)
0.93 (0.42, 2.08)
Doc+Nin vs Doc+Vand
1.12 (0.73, 1.77)
1.01 (0.50, 2.13)
1.17 (0.58, 2.54)
Doc+Nin vs Erl+Cab
2.17 (0.94, 4.94)
1.86 (0.61, 5.64)
Doc+Nin vs Erl+Fig
0.74 (0.44, 1.26)
0.43 (0.18, 1.06)
Doc+Nin vs Erl+Ona
0.78 (0.48, 1.28)
0.39 (0.14, 1.07)
0.70 (0.28, 1.75)
Doc+Nin vs Erl+Sun
0.97 (0.59, 1.59)
0.58 (0.24, 1.38)
0.82 (0.35, 1.90)
Doc+Nin vs Erl+Tiv
1.09 (0.69, 1.72)
0.95 (0.43, 2.09)
Doc+Nin vs Erlotinib
0.80 (0.54, 1.18)
0.47 (0.22, 0.96)
0.69 (0.34, 1.40)
Doc+Nin vs Gefitinib
0.82 (0.57, 1.20)
0.61 (0.27, 1.35)
Doc+Nin vs Icotinib
0.98 (0.58, 1.68)
0.69 (0.26, 1.86)
Doc+Nin vs Nivolumab
1.09 (0.71, 1.70)
1.24 (0.57, 2.73)
0.91 (0.41, 2.03)
Doc+Nin vs Pem+Cet
0.79 (0.47, 1.34)
0.65 (0.24, 1.73)
Doc+Nin vs Pembrolizumab
0.97 (0.59, 1.59)
0.89 (0.40, 2.01)
0.98 (0.44, 2.18)
Doc+Nin vs Pemetrexed
0.82 (0.56, 1.20)
0.62 (0.27, 1.37)
Doc+Nin vs Pralatrexate
0.88 (0.47, 1.62)
1.19 (0.43, 3.27)
Doc+Nin vs Trametinib
0.69 (0.38, 1.28)
0.68 (0.28, 1.64)
Doc+Nin vs Vandetanib
0.92 (0.59, 1.49)
0.43 (0.17, 1.07)
0.79 (0.35, 1.89)
Total population
Only squamous population
Only non-squamous population
.2
.3
.5
1
2
5

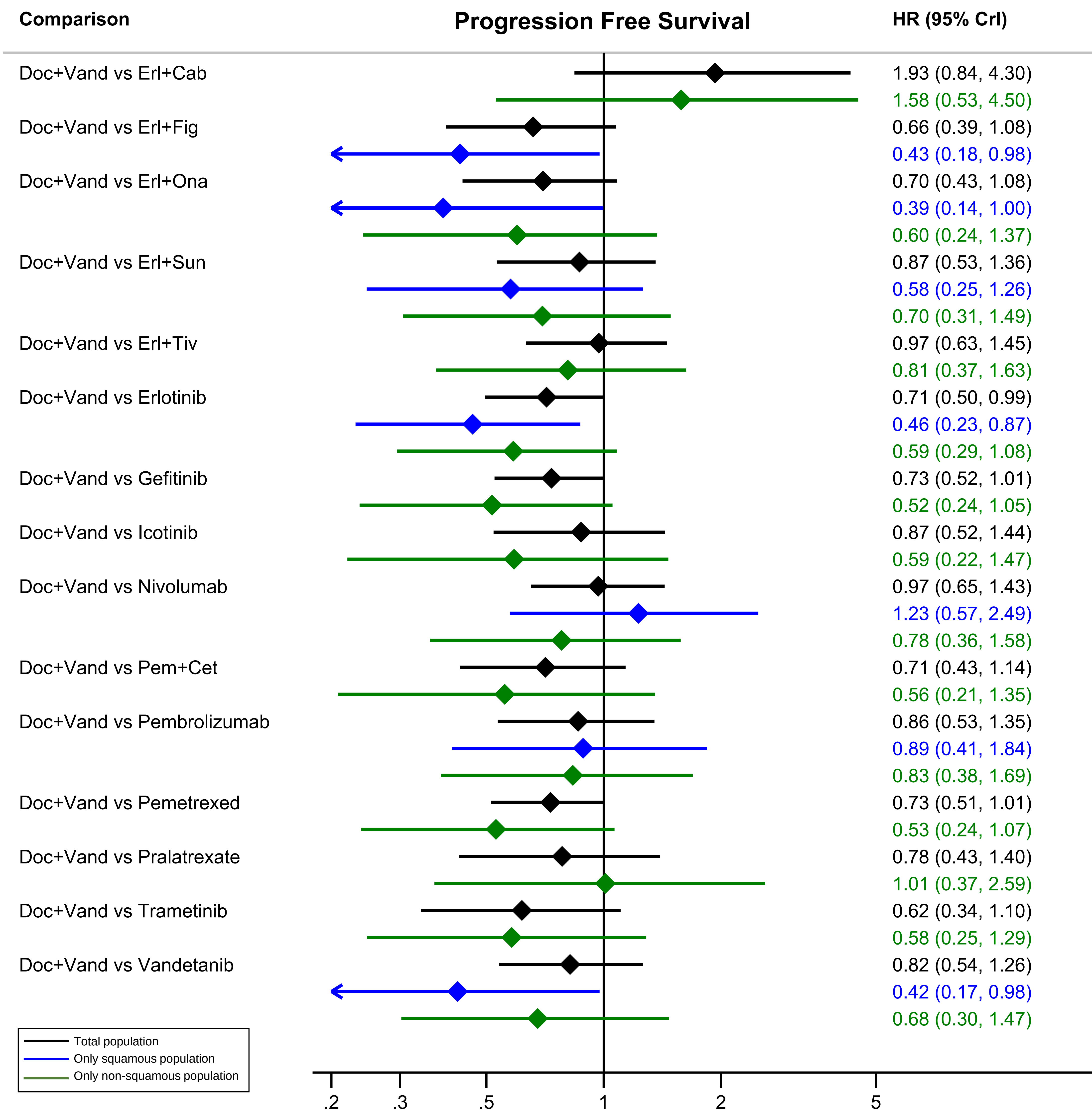
Comparison
Progression Free Survival
HR (95% CrI)
Doc+Vand vs Erl+Cab
1.93 (0.84, 4.30)
1.58 (0.53, 4.50)
Doc+Vand vs Erl+Fig
0.66 (0.39, 1.08)
0.43 (0.18, 0.98)
Doc+Vand vs Erl+Ona
0.70 (0.43, 1.08)
0.39 (0.14, 1.00)
0.60 (0.24, 1.37)
Doc+Vand vs Erl+Sun
0.87 (0.53, 1.36)
0.58 (0.25, 1.26)
0.70 (0.31, 1.49)
Doc+Vand vs Erl+Tiv
0.97 (0.63, 1.45)
0.81 (0.37, 1.63)
Doc+Vand vs Erlotinib
0.71 (0.50, 0.99)
0.46 (0.23, 0.87)
0.59 (0.29, 1.08)
Doc+Vand vs Gefitinib
0.73 (0.52, 1.01)
0.52 (0.24, 1.05)
Doc+Vand vs Icotinib
0.87 (0.52, 1.44)
0.59 (0.22, 1.47)
Doc+Vand vs Nivolumab
0.97 (0.65, 1.43)
1.23 (0.57, 2.49)
0.78 (0.36, 1.58)
Doc+Vand vs Pem+Cet
0.71 (0.43, 1.14)
0.56 (0.21, 1.35)
Doc+Vand vs Pembrolizumab
0.86 (0.53, 1.35)
0.89 (0.41, 1.84)
0.83 (0.38, 1.69)
Doc+Vand vs Pemetrexed
0.73 (0.51, 1.01)
0.53 (0.24, 1.07)
Doc+Vand vs Pralatrexate
0.78 (0.43, 1.40)
1.01 (0.37, 2.59)
Doc+Vand vs Trametinib
0.62 (0.34, 1.10)
0.58 (0.25, 1.29)
Doc+Vand vs Vandetanib
0.82 (0.54, 1.26)
0.42 (0.17, 0.98)
0.68 (0.30, 1.47)
Total population
Only squamous population
Only non-squamous population
.2
.3
.5
1
2
5

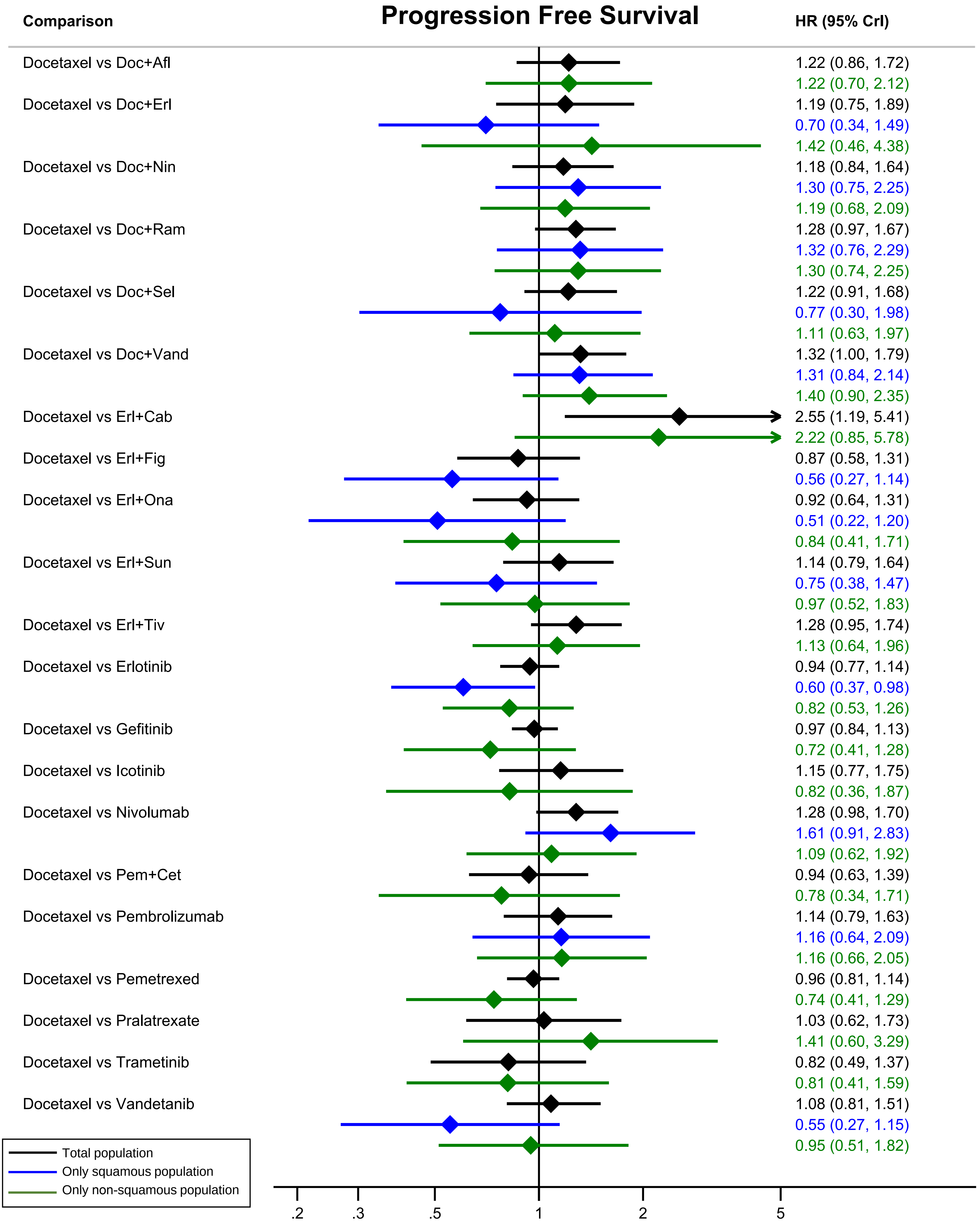
Progression Free Survival
Comparison
HR (95% CrI)
Docetaxel vs Doc+Afl
1.22 (0.86, 1.72)
1.22 (0.70, 2.12)
Docetaxel vs Doc+Erl
1.19 (0.75, 1.89)
0.70 (0.34, 1.49)
1.42 (0.46, 4.38)
Docetaxel vs Doc+Nin
1.18 (0.84, 1.64)
1.30 (0.75, 2.25)
1.19 (0.68, 2.09)
Docetaxel vs Doc+Ram
1.28 (0.97, 1.67)
1.32 (0.76, 2.29)
1.30 (0.74, 2.25)
Docetaxel vs Doc+Sel
1.22 (0.91, 1.68)
0.77 (0.30, 1.98)
1.11 (0.63, 1.97)
Docetaxel vs Doc+Vand
1.32 (1.00, 1.79)
1.31 (0.84, 2.14)
1.40 (0.90, 2.35)
Docetaxel vs Erl+Cab
2.55 (1.19, 5.41)
2.22 (0.85, 5.78)
Docetaxel vs Erl+Fig
0.87 (0.58, 1.31)
0.56 (0.27, 1.14)
Docetaxel vs Erl+Ona
0.92 (0.64, 1.31)
0.51 (0.22, 1.20)
0.84 (0.41, 1.71)
Docetaxel vs Erl+Sun
1.14 (0.79, 1.64)
0.75 (0.38, 1.47)
0.97 (0.52, 1.83)
Docetaxel vs Erl+Tiv
1.28 (0.95, 1.74)
1.13 (0.64, 1.96)
Docetaxel vs Erlotinib
0.94 (0.77, 1.14)
0.60 (0.37, 0.98)
0.82 (0.53, 1.26)
Docetaxel vs Gefitinib
0.97 (0.84, 1.13)
0.72 (0.41, 1.28)
Docetaxel vs Icotinib
1.15 (0.77, 1.75)
0.82 (0.36, 1.87)
Docetaxel vs Nivolumab
1.28 (0.98, 1.70)
1.61 (0.91, 2.83)
1.09 (0.62, 1.92)
Docetaxel vs Pem+Cet
0.94 (0.63, 1.39)
0.78 (0.34, 1.71)
Docetaxel vs Pembrolizumab
1.14 (0.79, 1.63)
1.16 (0.64, 2.09)
1.16 (0.66, 2.05)
Docetaxel vs Pemetrexed
0.96 (0.81, 1.14)
0.74 (0.41, 1.29)
Docetaxel vs Pralatrexate
1.03 (0.62, 1.73)
1.41 (0.60, 3.29)
Docetaxel vs Trametinib
0.82 (0.49, 1.37)
0.81 (0.41, 1.59)
Docetaxel vs Vandetanib
1.08 (0.81, 1.51)
0.55 (0.27, 1.15)
0.95 (0.51, 1.82)
Total population
Only squamous population
Only non-squamous population
.2
.3
.5
1
2
5

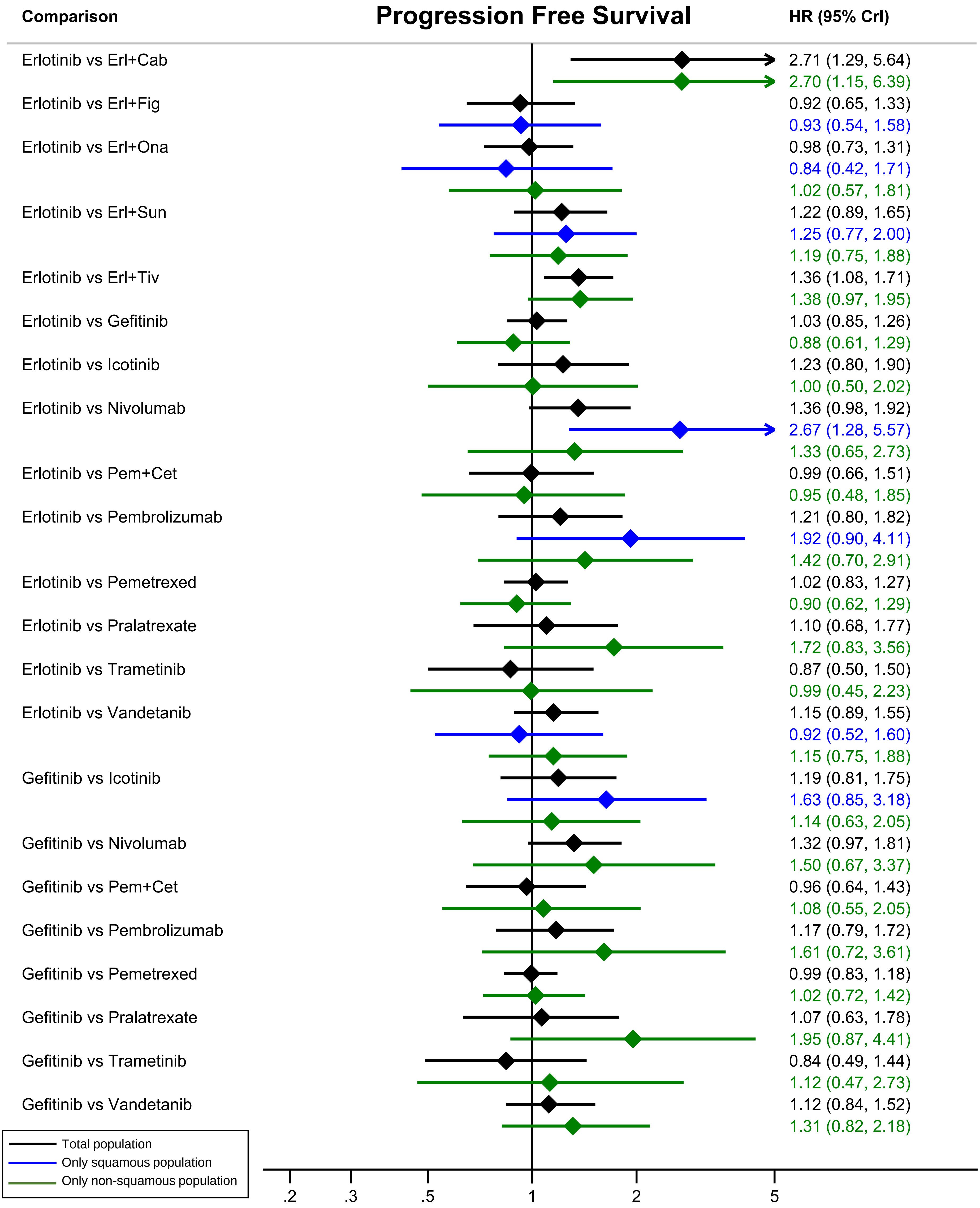
Comparison
Progression Free Survival
HR (95% CrI)
Erlotinib vs Erl+Cab
2.71 (1.29, 5.64)
2.70 (1.15, 6.39)
Erlotinib vs Erl+Fig
0.92 (0.65, 1.33)
0.93 (0.54, 1.58)
Erlotinib vs Erl+Ona
0.98 (0.73, 1.31)
0.84 (0.42, 1.71)
1.02 (0.57, 1.81)
Erlotinib vs Erl+Sun
1.22 (0.89, 1.65)
1.25 (0.77, 2.00)
1.19 (0.75, 1.88)
Erlotinib vs Erl+Tiv
1.36 (1.08, 1.71)
1.38 (0.97, 1.95)
Erlotinib vs Gefitinib
1.03 (0.85, 1.26)
0.88 (0.61, 1.29)
Erlotinib vs Icotinib
1.23 (0.80, 1.90)
1.00 (0.50, 2.02)
Erlotinib vs Nivolumab
1.36 (0.98, 1.92)
2.67 (1.28, 5.57)
1.33 (0.65, 2.73)
Erlotinib vs Pem+Cet
0.99 (0.66, 1.51)
0.95 (0.48, 1.85)
Erlotinib vs Pembrolizumab
1.21 (0.80, 1.82)
1.92 (0.90, 4.11)
1.42 (0.70, 2.91)
Erlotinib vs Pemetrexed
1.02 (0.83, 1.27)
0.90 (0.62, 1.29)
Erlotinib vs Pralatrexate
1.10 (0.68, 1.77)
1.72 (0.83, 3.56)
Erlotinib vs Trametinib
0.87 (0.50, 1.50)
0.99 (0.45, 2.23)
Erlotinib vs Vandetanib
1.15 (0.89, 1.55)
0.92 (0.52, 1.60)
1.15 (0.75, 1.88)
Gefitinib vs Icotinib
1.19 (0.81, 1.75)
1.63 (0.85, 3.18)
1.14 (0.63, 2.05)
Gefitinib vs Nivolumab
1.32 (0.97, 1.81)
1.50 (0.67, 3.37)
Gefitinib vs Pem+Cet
0.96 (0.64, 1.43)
1.08 (0.55, 2.05)
Gefitinib vs Pembrolizumab
1.17 (0.79, 1.72)
1.61 (0.72, 3.61)
Gefitinib vs Pemetrexed
0.99 (0.83, 1.18)
1.02 (0.72, 1.42)
Gefitinib vs Pralatrexate
1.07 (0.63, 1.78)
1.95 (0.87, 4.41)
Gefitinib vs Trametinib
0.84 (0.49, 1.44)
1.12 (0.47, 2.73)
Gefitinib vs Vandetanib
1.12 (0.84, 1.52)
1.31 (0.82, 2.18)
Total population
Only squamous population
Only non-squamous population
.2
.3
.5
1
2
5

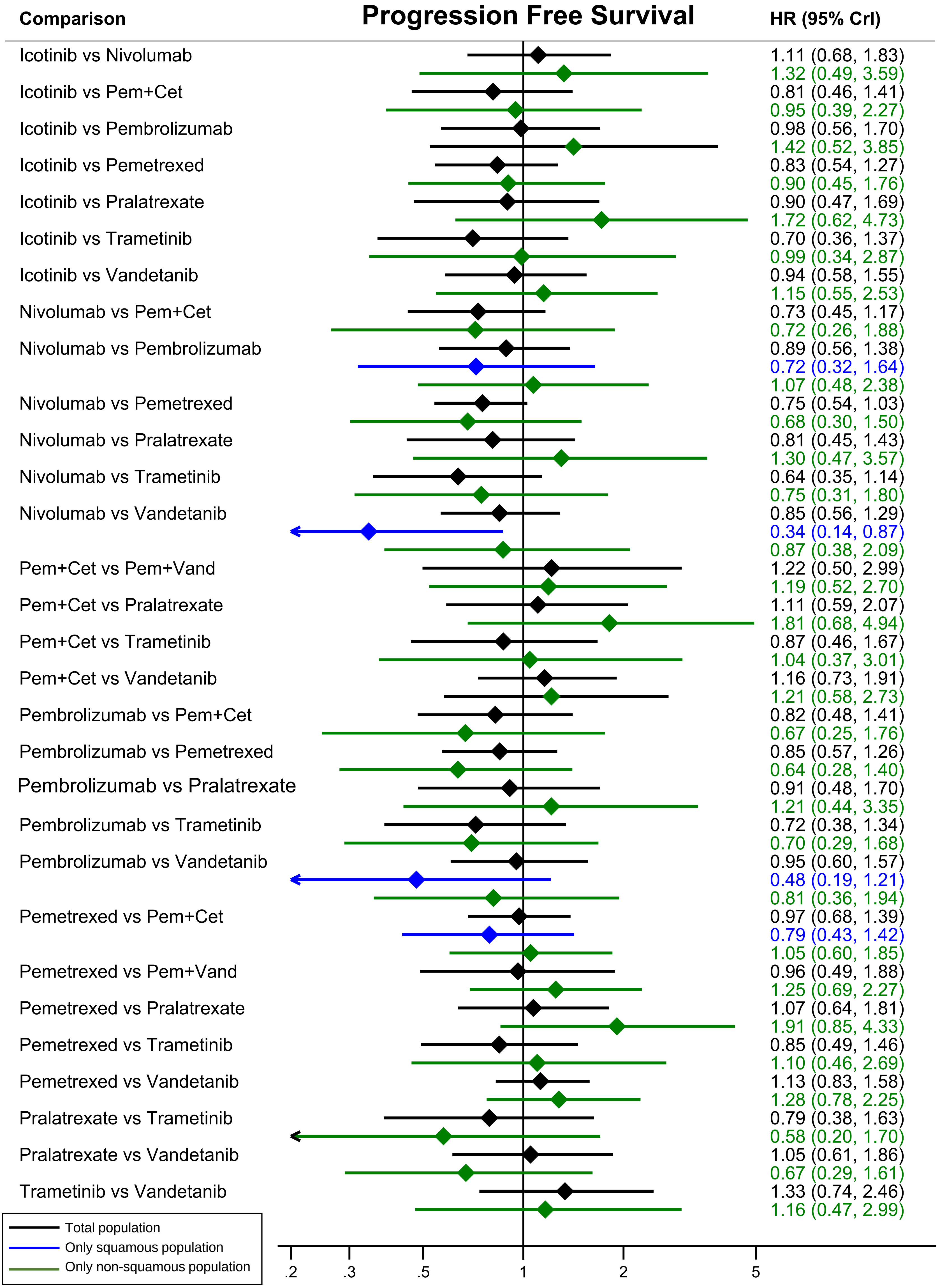
Comparison
Progression Free Survival
HR (95% CrI)
Icotinib vs Nivolumab
1.11 (0.68, 1.83)
1.32 (0.49, 3.59)
Icotinib vs Pem+Cet
0.81 (0.46, 1.41)
0.95 (0.39, 2.27)
Icotinib vs Pembrolizumab
0.98 (0.56, 1.70)
1.42 (0.52, 3.85)
Icotinib vs Pemetrexed
0.83 (0.54, 1.27)
0.90 (0.45, 1.76)
Icotinib vs Pralatrexate
0.90 (0.47, 1.69)
1.72 (0.62, 4.73)
Icotinib vs Trametinib
0.70 (0.36, 1.37)
0.99 (0.34, 2.87)
Icotinib vs Vandetanib
0.94 (0.58, 1.55)
1.15 (0.55, 2.53)
Nivolumab vs Pem+Cet
0.73 (0.45, 1.17)
0.72 (0.26, 1.88)
Nivolumab vs Pembrolizumab
0.89 (0.56, 1.38)
0.72 (0.32, 1.64)
1.07 (0.48, 2.38)
Nivolumab vs Pemetrexed
0.75 (0.54, 1.03)
0.68 (0.30, 1.50)
Nivolumab vs Pralatrexate
0.81 (0.45, 1.43)
1.30 (0.47, 3.57)
Nivolumab vs Trametinib
0.64 (0.35, 1.14)
0.75 (0.31, 1.80)
Nivolumab vs Vandetanib
0.85 (0.56, 1.29)
0.34 (0.14, 0.87)
0.87 (0.38, 2.09)
Pem+Cet vs Pem+Vand
1.22 (0.50, 2.99)
1.19 (0.52, 2.70)
Pem+Cet vs Pralatrexate
1.11 (0.59, 2.07)
1.81 (0.68, 4.94)
Pem+Cet vs Trametinib
0.87 (0.46, 1.67)
1.04 (0.37, 3.01)
Pem+Cet vs Vandetanib
1.16 (0.73, 1.91)
1.21 (0.58, 2.73)
Pembrolizumab vs Pem+Cet
0.82 (0.48, 1.41)
0.67 (0.25, 1.76)
Pembrolizumab vs Pemetrexed
0.85 (0.57, 1.26)
0.64 (0.28, 1.40)
Pembrolizumab vs Pralatrexate
0.91 (0.48, 1.70)
1.21 (0.44, 3.35)
Pembrolizumab vs Trametinib
0.72 (0.38, 1.34)
0.70 (0.29, 1.68)
Pembrolizumab vs Vandetanib
0.95 (0.60, 1.57)
0.48 (0.19, 1.21)
0.81 (0.36, 1.94)
Pemetrexed vs Pem+Cet
0.97 (0.68, 1.39)
0.79 (0.43, 1.42)
1.05 (0.60, 1.85)
Pemetrexed vs Pem+Vand
0.96 (0.49, 1.88)
1.25 (0.69, 2.27)
Pemetrexed vs Pralatrexate
1.07 (0.64, 1.81)
1.91 (0.85, 4.33)
Pemetrexed vs Trametinib
0.85 (0.49, 1.46)
1.10 (0.46, 2.69)
Pemetrexed vs Vandetanib
1.13 (0.83, 1.58)
1.28 (0.78, 2.25)
Pralatrexate vs Trametinib
0.79 (0.38, 1.63)
0.58 (0.20, 1.70)
Pralatrexate vs Vandetanib
1.05 (0.61, 1.86)
0.67 (0.29, 1.61)
Trametinib vs Vandetanib
1.33 (0.74, 2.46)
1.16 (0.47, 2.99)
Total population
Only squamous population
Only non-squamous population
.2
.3
.5
1
2
5

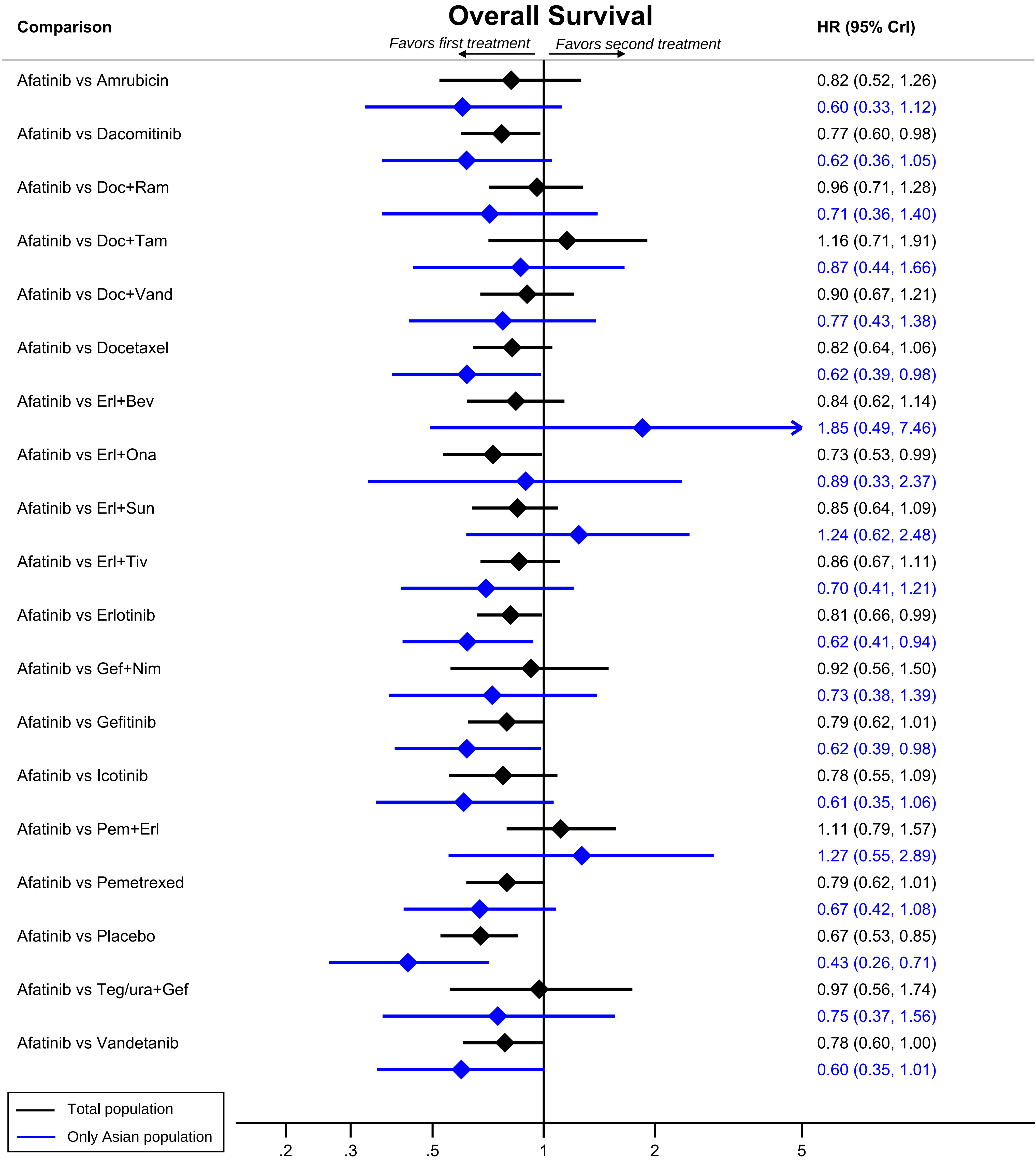

Overall Survival
Comparison
HR (95% CrI)
Favors first treatment
Favors second treatment
Afatinib vs Amrubicin
0.82 (0.52, 1.26)
0.60 (0.33, 1.12)
Afatinib vs Dacomitinib
0.77 (0.60, 0.98)
0.62 (0.36, 1.05)
Afatinib vs Doc+Ram
0.96 (0.71, 1.28)
0.71 (0.36, 1.40)
Afatinib vs Doc+Tam
1.16 (0.71, 1.91)
0.87 (0.44, 1.66)
Afatinib vs Doc+Vand
0.90 (0.67, 1.21)
0.77 (0.43, 1.38)
Afatinib vs Docetaxel
0.82 (0.64, 1.06)
0.62 (0.39, 0.98)
Afatinib vs Erl+Bev
0.84 (0.62, 1.14)
1.85 (0.49, 7.46)
Afatinib vs Erl+Ona
0.73 (0.53, 0.99)
0.89 (0.33, 2.37)
Afatinib vs Erl+Sun
0.85 (0.64, 1.09)
1.24 (0.62, 2.48)
Afatinib vs Erl+Tiv
0.86 (0.67, 1.11)
0.70 (0.41, 1.21)
Afatinib vs Erlotinib
0.81 (0.66, 0.99)
0.62 (0.41, 0.94)
Afatinib vs Gef+Nim
0.92 (0.56, 1.50)
0.73 (0.38, 1.39)
Afatinib vs Gefitinib
0.79 (0.62, 1.01)
0.62 (0.39, 0.98)
Afatinib vs Icotinib
0.78 (0.55, 1.09)
0.61 (0.35, 1.06)
Afatinib vs Pem+Erl
1.11 (0.79, 1.57)
1.27 (0.55, 2.89)
Afatinib vs Pemetrexed
0.79 (0.62, 1.01)
0.67 (0.42, 1.08)
Afatinib vs Placebo
0.67 (0.53, 0.85)
0.43 (0.26, 0.71)
Afatinib vs Teg/ura+Gef
0.97 (0.56, 1.74)
0.75 (0.37, 1.56)
Afatinib vs Vandetanib
0.78 (0.60, 1.00)
0.60 (0.35, 1.01)
Total population
Only Asian population
.2
.3
.5
1
2
5

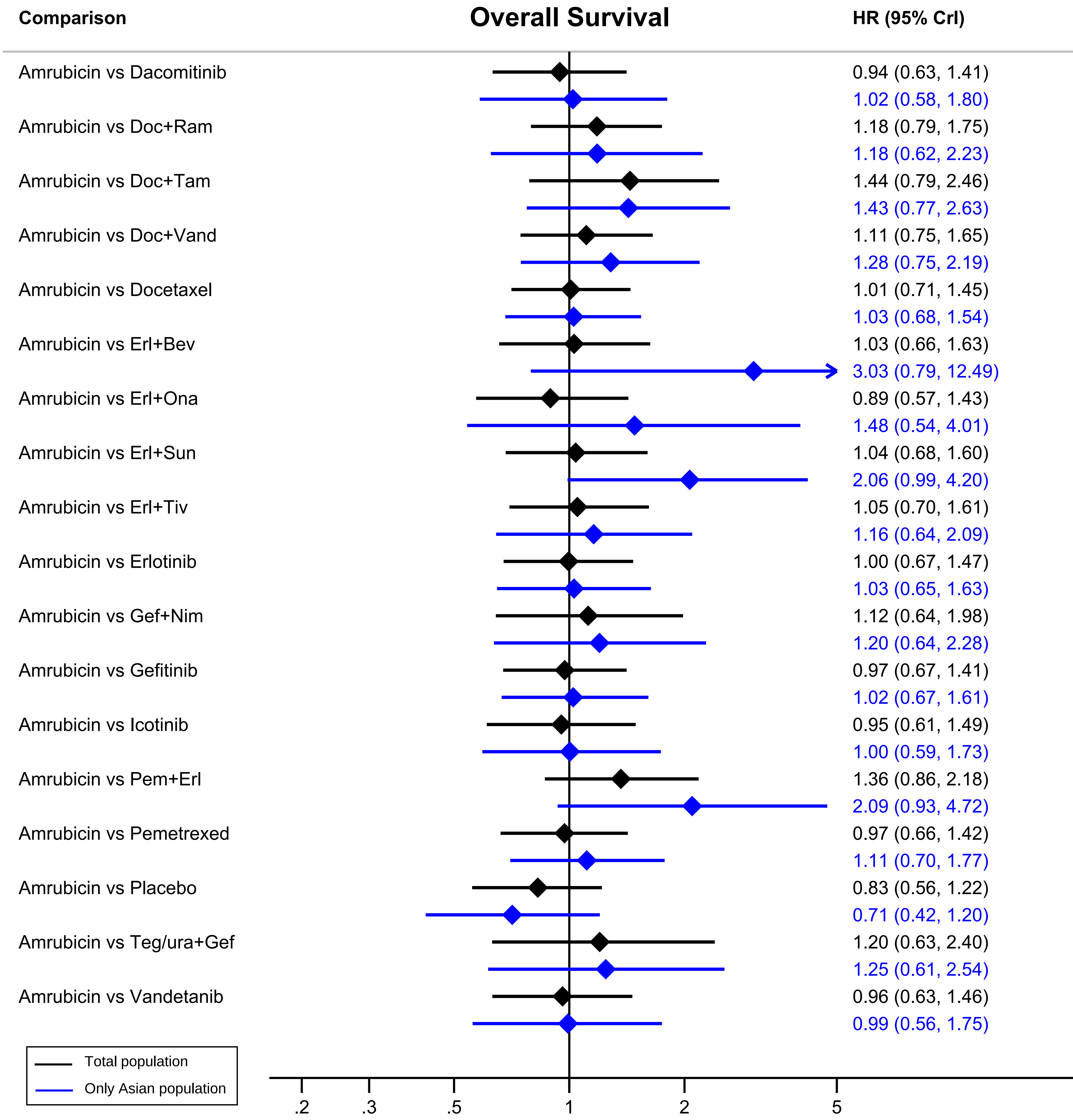
Comparison
Overall Survival
HR (95% CrI)
Amrubicin vs Dacomitinib
0.94 (0.63, 1.41)
1.02 (0.58, 1.80)
Amrubicin vs Doc+Ram
1.18 (0.79, 1.75)
1.18 (0.62, 2.23)
Amrubicin vs Doc+Tam
1.44 (0.79, 2.46)
1.43 (0.77, 2.63)
Amrubicin vs Doc+Vand
1.11 (0.75, 1.65)
1.28 (0.75, 2.19)
Amrubicin vs Docetaxel
1.01 (0.71, 1.45)
1.03 (0.68, 1.54)
Amrubicin vs Erl+Bev
1.03 (0.66, 1.63)
3.03 (0.79, 12.49)
Amrubicin vs Erl+Ona
0.89 (0.57, 1.43)
1.48 (0.54, 4.01)
Amrubicin vs Erl+Sun
1.04 (0.68, 1.60)
2.06 (0.99, 4.20)
Amrubicin vs Erl+Tiv
1.05 (0.70, 1.61)
1.16 (0.64, 2.09)
Amrubicin vs Erlotinib
1.00 (0.67, 1.47)
1.03 (0.65, 1.63)
Amrubicin vs Gef+Nim
1.12 (0.64, 1.98)
1.20 (0.64, 2.28)
Amrubicin vs Gefitinib
0.97 (0.67, 1.41)
1.02 (0.67, 1.61)
Amrubicin vs Icotinib
0.95 (0.61, 1.49)
1.00 (0.59, 1.73)
Amrubicin vs Pem+Erl
1.36 (0.86, 2.18)
2.09 (0.93, 4.72)
Amrubicin vs Pemetrexed
0.97 (0.66, 1.42)
1.11 (0.70, 1.77)
Amrubicin vs Placebo
0.83 (0.56, 1.22)
0.71 (0.42, 1.20)
Amrubicin vs Teg/ura+Gef
1.20 (0.63, 2.40)
1.25 (0.61, 2.54)
Amrubicin vs Vandetanib
0.96 (0.63, 1.46)
0.99 (0.56, 1.75)
Total population
Only Asian population
.2
.3
.5
1
2
5

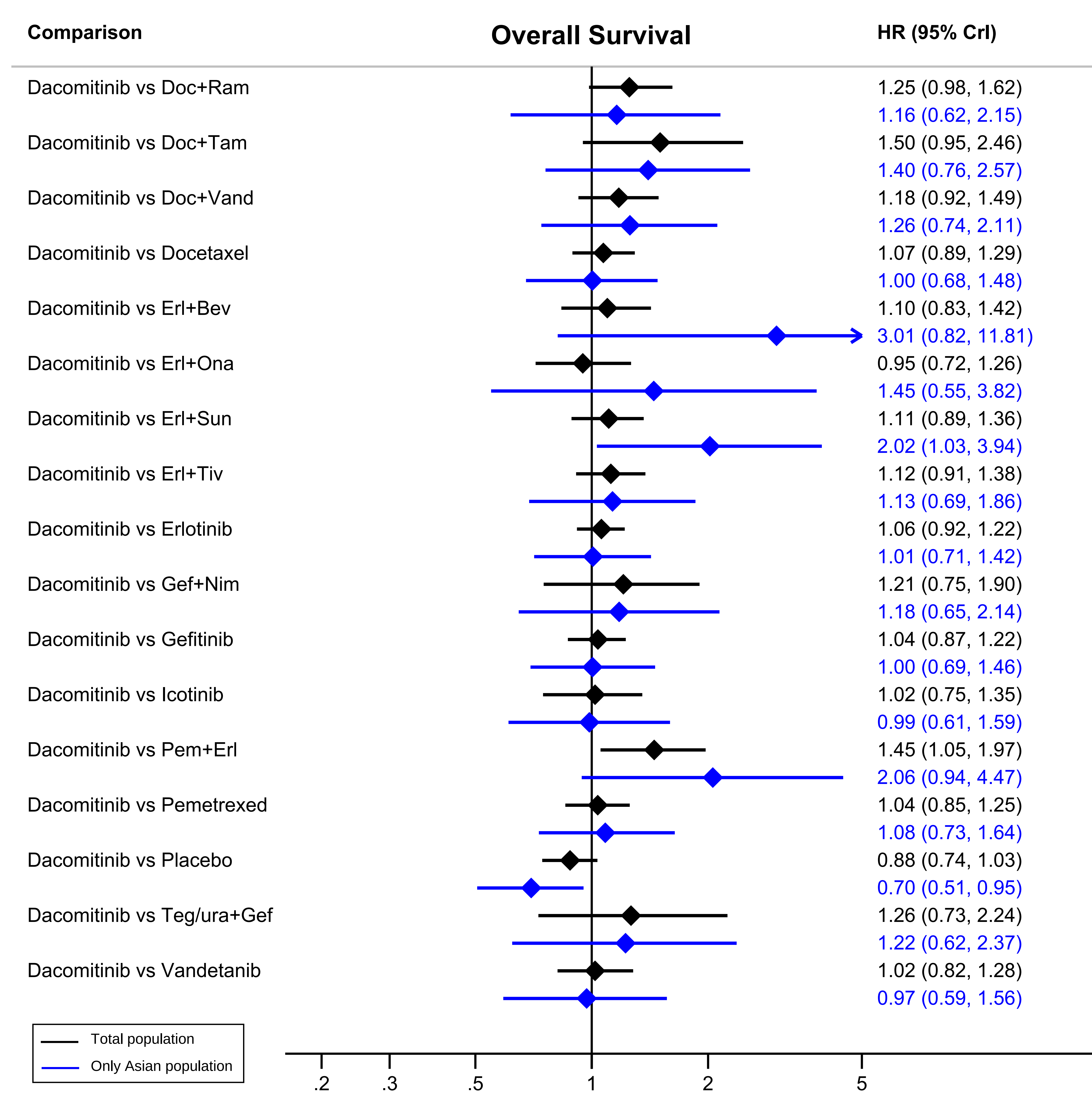
Comparison
Overall Survival
HR (95% CrI)
Dacomitinib vs Doc+Ram
1.25 (0.98, 1.62)
1.16 (0.62, 2.15)
Dacomitinib vs Doc+Tam
1.50 (0.95, 2.46)
1.40 (0.76, 2.57)
Dacomitinib vs Doc+Vand
1.18 (0.92, 1.49)
1.26 (0.74, 2.11)
Dacomitinib vs Docetaxel
1.07 (0.89, 1.29)
1.00 (0.68, 1.48)
Dacomitinib vs Erl+Bev
1.10 (0.83, 1.42)
3.01 (0.82, 11.81)
Dacomitinib vs Erl+Ona
0.95 (0.72, 1.26)
1.45 (0.55, 3.82)
Dacomitinib vs Erl+Sun
1.11 (0.89, 1.36)
2.02 (1.03, 3.94)
Dacomitinib vs Erl+Tiv
1.12 (0.91, 1.38)
1.13 (0.69, 1.86)
Dacomitinib vs Erlotinib
1.06 (0.92, 1.22)
1.01 (0.71, 1.42)
Dacomitinib vs Gef+Nim
1.21 (0.75, 1.90)
1.18 (0.65, 2.14)
Dacomitinib vs Gefitinib
1.04 (0.87, 1.22)
1.00 (0.69, 1.46)
Dacomitinib vs Icotinib
1.02 (0.75, 1.35)
0.99 (0.61, 1.59)
Dacomitinib vs Pem+Erl
1.45 (1.05, 1.97)
2.06 (0.94, 4.47)
Dacomitinib vs Pemetrexed
1.04 (0.85, 1.25)
1.08 (0.73, 1.64)
Dacomitinib vs Placebo
0.88 (0.74, 1.03)
0.70 (0.51, 0.95)
Dacomitinib vs Teg/ura+Gef
1.26 (0.73, 2.24)
1.22 (0.62, 2.37)
Dacomitinib vs Vandetanib
1.02 (0.82, 1.28)
0.97 (0.59, 1.56)
Total population
Only Asian population
.2
.3
.5
1
2
5

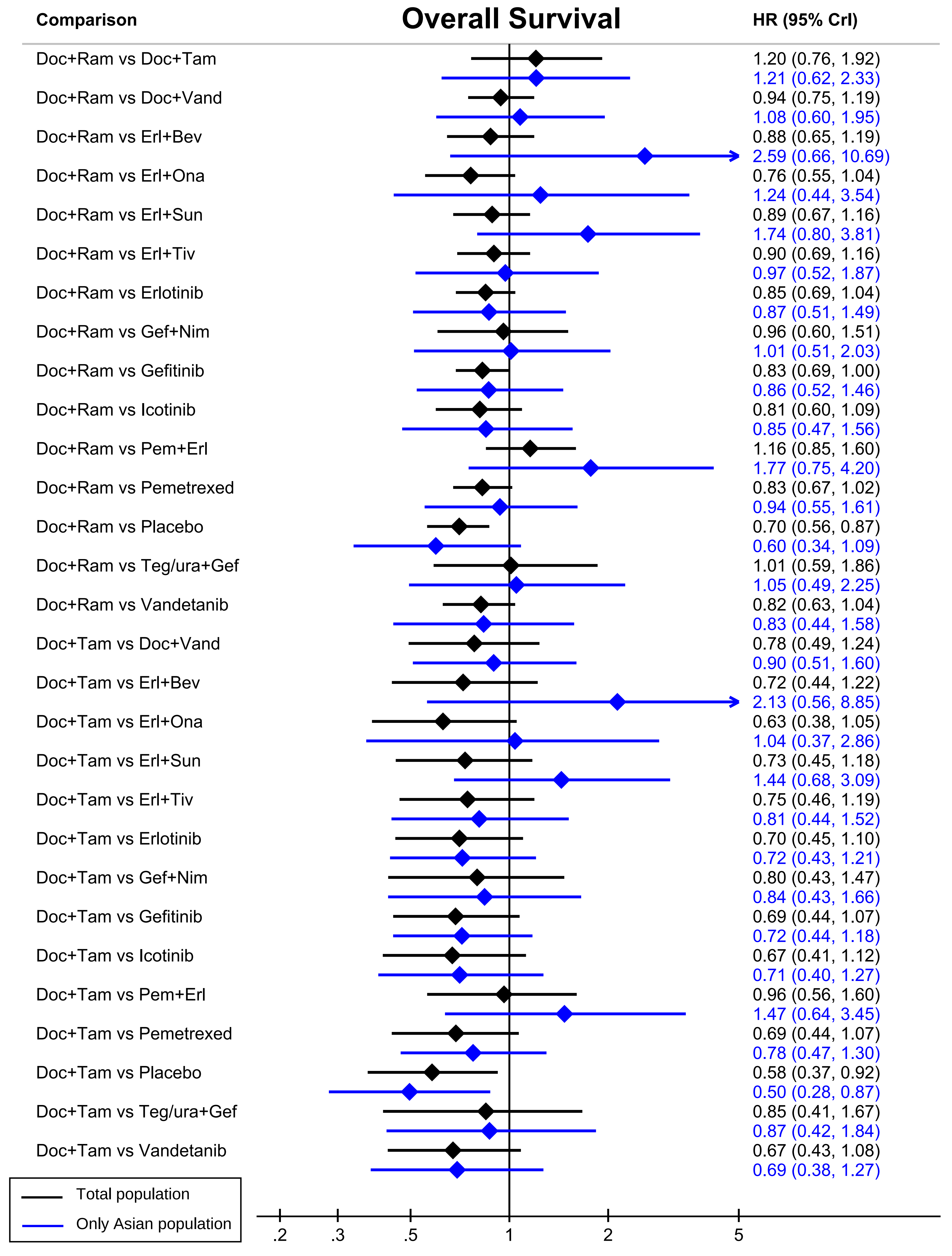
Comparison
Overall Survival
HR (95% Crl)
Doc+Ram vs Doc+Tam
1.20 (0.76, 1.92)
1.21 (0.62, 2.33)
Doc+Ram vs Doc+Vand
0.94 (0.75, 1.19)
1.08 (0.60, 1.95)
Doc+Ram vs Erl+Bev
0.88 (0.65, 1.19)
2.59 (0.66, 10.69)
Doc+Ram vs Erl+Ona
0.76 (0.55, 1.04)
1.24 (0.44, 3.54)
Doc+Ram vs Erl+Sun
0.89 (0.67, 1.16)
1.74 (0.80, 3.81)
Doc+Ram vs Erl+Tiv
0.90 (0.69, 1.16)
0.97 (0.52, 1.87)
Doc+Ram vs Erlotinib
0.85 (0.69, 1.04)
0.87 (0.51, 1.49)
Doc+Ram vs Gef+Nim
0.96 (0.60, 1.51)
1.01 (0.51, 2.03)
Doc+Ram vs Gefitinib
0.83 (0.69, 1.00)
0.86 (0.52, 1.46)
Doc+Ram vs Icotinib
0.81 (0.60, 1.09)
0.85 (0.47, 1.56)
Doc+Ram vs Pem+Erl
1.16 (0.85, 1.60)
1.77 (0.75, 4.20)
Doc+Ram vs Pemetrexed
0.83 (0.67, 1.02)
0.94 (0.55, 1.61)
Doc+Ram vs Placebo
0.70 (0.56, 0.87)
0.60 (0.34, 1.09)
Doc+Ram vs Teg/ura+Gef
1.01 (0.59, 1.86)
1.05 (0.49, 2.25)
Doc+Ram vs Vandetanib
0.82 (0.63, 1.04)
0.83 (0.44, 1.58)
Doc+Tam vs Doc+Vand
0.78 (0.49, 1.24)
0.90 (0.51, 1.60)
Doc+Tam vs Erl+Bev
0.72 (0.44, 1.22)
2.13 (0.56, 8.85)
Doc+Tam vs Erl+Ona
0.63 (0.38, 1.05)
1.04 (0.37, 2.86)
Doc+Tam vs Erl+Sun
0.73 (0.45, 1.18)
1.44 (0.68, 3.09)
Doc+Tam vs Erl+Tiv
0.75 (0.46, 1.19)
0.81 (0.44, 1.52)
Doc+Tam vs Erlotinib
0.70 (0.45, 1.10)
0.72 (0.43, 1.21)
Doc+Tam vs Gef+Nim
0.80 (0.43, 1.47)
0.84 (0.43, 1.66)
Doc+Tam vs Gefitinib
0.69 (0.44, 1.07)
0.72 (0.44, 1.18)
Doc+Tam vs Icotinib
0.67 (0.41, 1.12)
0.71 (0.40, 1.27)
Doc+Tam vs Pem+Erl
0.96 (0.56, 1.60)
1.47 (0.64, 3.45)
Doc+Tam vs Pemetrexed
0.69 (0.44, 1.07)
0.78 (0.47, 1.30)
Doc+Tam vs Placebo
0.58 (0.37, 0.92)
0.50 (0.28, 0.87)
Doc+Tam vs Teg/ura+Gef
0.85 (0.41, 1.67)
0.87 (0.42, 1.84)
Doc+Tam vs Vandetanib
0.67 (0.43, 1.08)
0.69 (0.38, 1.27)
Total population
Only Asian population
.2
.3
.5
1
2
5

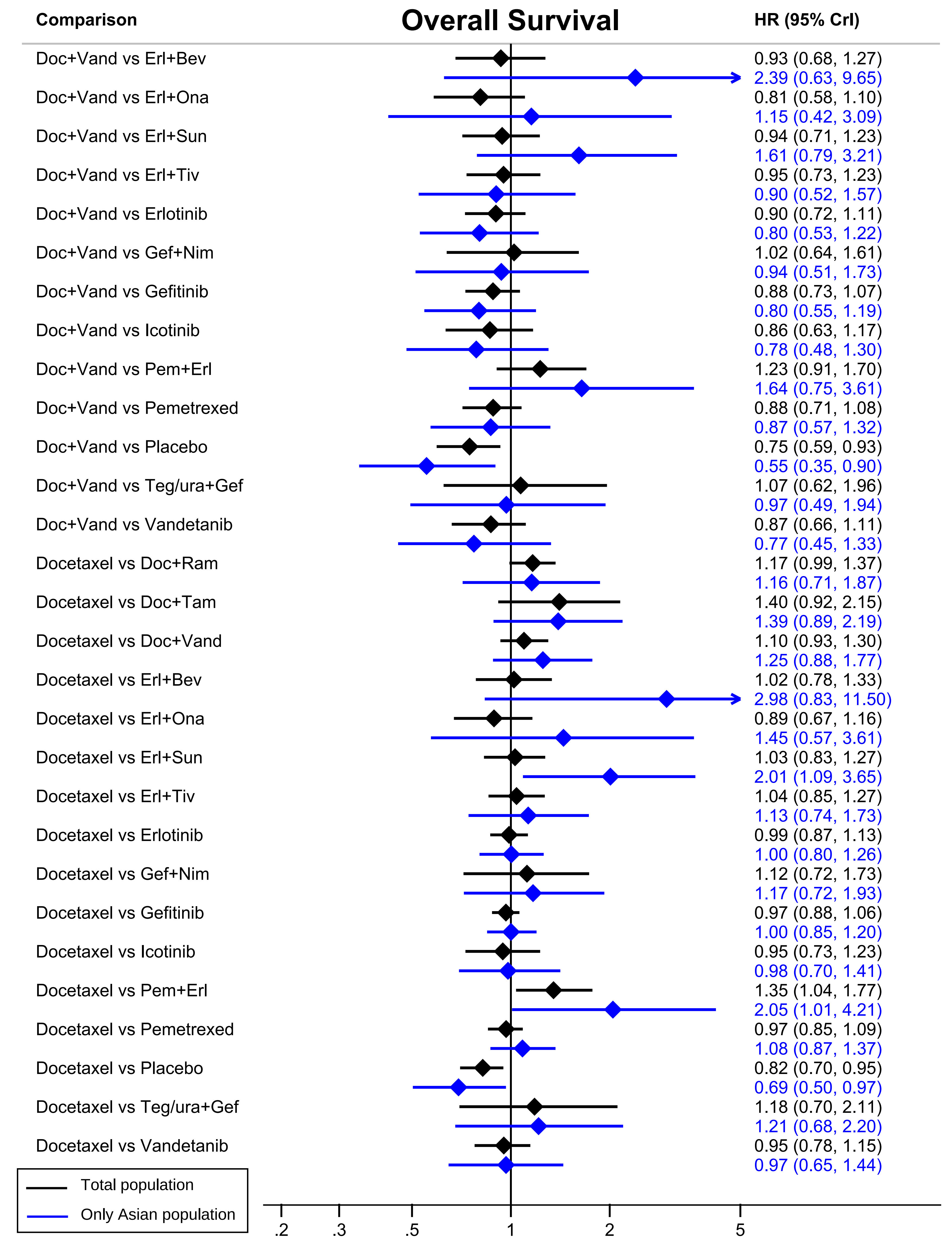
Comparison
Overall Survival
HR (95% CrI)
Doc+Vand vs Erl+Bev
0.93 (0.68, 1.27)
2.39 (0.63, 9.65)
Doc+Vand vs Erl+Ona
0.81 (0.58, 1.10)
1.15 (0.42, 3.09)
Doc+Vand vs Erl+Sun
0.94 (0.71, 1.23)
1.61 (0.79, 3.21)
Doc+Vand vs Erl+Tiv
0.95 (0.73, 1.23)
0.90 (0.52, 1.57)
Doc+Vand vs Erlotinib
0.90 (0.72, 1.11)
0.80 (0.53, 1.22)
Doc+Vand vs Gef+Nim
1.02 (0.64, 1.61)
0.94 (0.51, 1.73)
Doc+Vand vs Gefitinib
0.88 (0.73, 1.07)
0.80 (0.55, 1.19)
Doc+Vand vs Icotinib
0.86 (0.63, 1.17)
0.78 (0.48, 1.30)
Doc+Vand vs Pem+Erl
1.23 (0.91, 1.70)
1.64 (0.75, 3.61)
Doc+Vand vs Pemetrexed
0.88 (0.71, 1.08)
0.87 (0.57, 1.32)
Doc+Vand vs Placebo
0.75 (0.59, 0.93)
0.55 (0.35, 0.90)
Doc+Vand vs Teg/ura+Gef
1.07 (0.62, 1.96)
0.97 (0.49, 1.94)
Doc+Vand vs Vandetanib
0.87 (0.66, 1.11)
0.77 (0.45, 1.33)
Docetaxel vs Doc+Ram
1.17 (0.99, 1.37)
1.16 (0.71, 1.87)
Docetaxel vs Doc+Tam
1.40 (0.92, 2.15)
1.39 (0.89, 2.19)
Docetaxel vs Doc+Vand
1.10 (0.93, 1.30)
1.25 (0.88, 1.77)
Docetaxel vs Erl+Bev
1.02 (0.78, 1.33)
2.98 (0.83, 11.50)
Docetaxel vs Erl+Ona
0.89 (0.67, 1.16)
1.45 (0.57, 3.61)
Docetaxel vs Erl+Sun
1.03 (0.83, 1.27)
2.01 (1.09, 3.65)
Docetaxel vs Erl+Tiv
1.04 (0.85, 1.27)
1.13 (0.74, 1.73)
Docetaxel vs Erlotinib
0.99 (0.87, 1.13)
1.00 (0.80, 1.26)
Docetaxel vs Gef+Nim
1.12 (0.72, 1.73)
1.17 (0.72, 1.93)
Docetaxel vs Gefitinib
0.97 (0.88, 1.06)
1.00 (0.85, 1.20)
Docetaxel vs Icotinib
0.95 (0.73, 1.23)
0.98 (0.70, 1.41)
Docetaxel vs Pem+Erl
1.35 (1.04, 1.77)
2.05 (1.01, 4.21)
Docetaxel vs Pemetrexed
0.97 (0.85, 1.09)
1.08 (0.87, 1.37)
Docetaxel vs Placebo
0.82 (0.70, 0.95)
0.69 (0.50, 0.97)
Docetaxel vs Teg/ura+Gef
1.18 (0.70, 2.11)
1.21 (0.68, 2.20)
Docetaxel vs Vandetanib
0.95 (0.78, 1.15)
0.97 (0.65, 1.44)
Total population
Only Asian population
.2
.3
.5
1
2
5

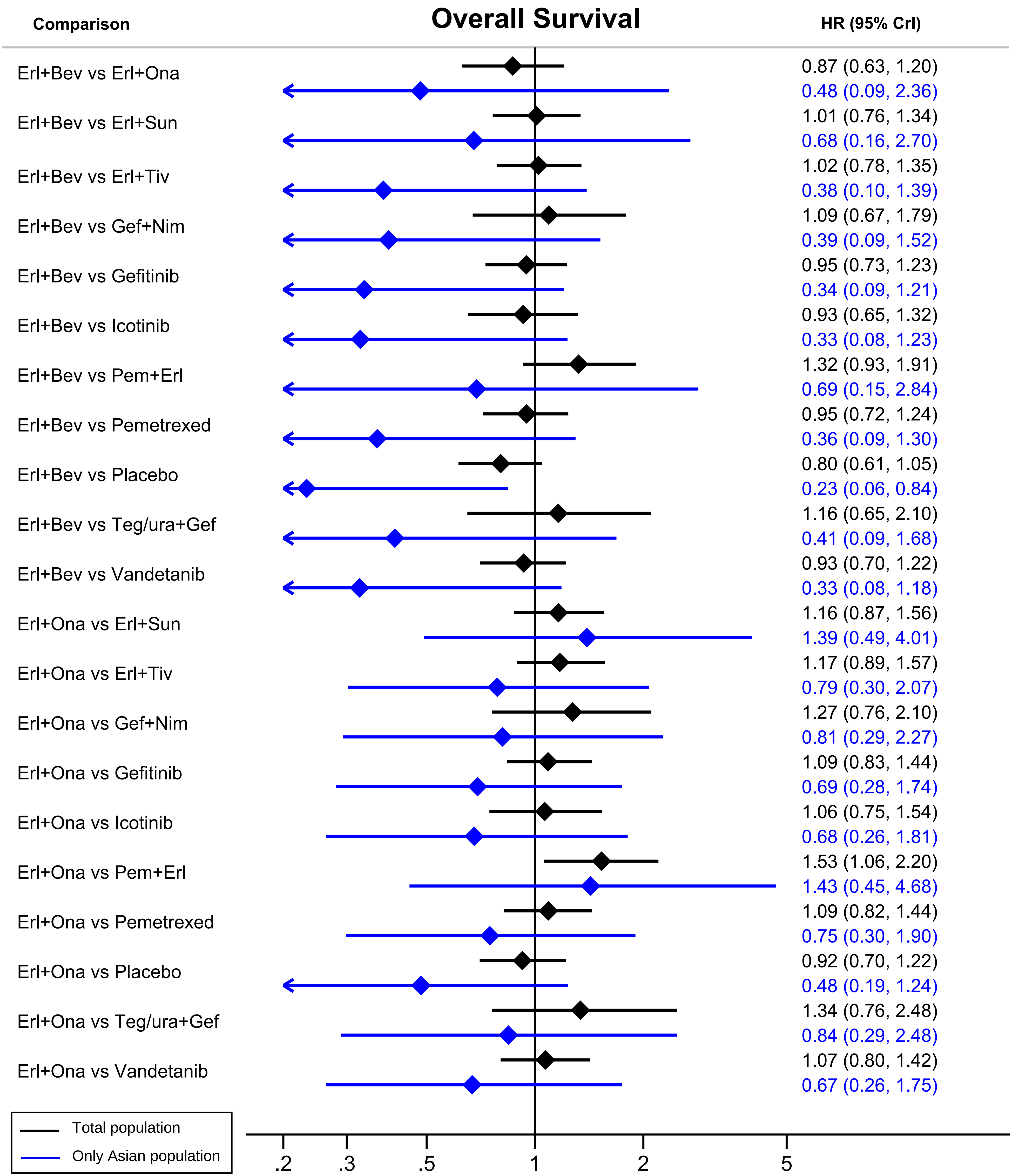
Overall Survival
Comparison
HR (95% CrI)
Erl+Bev vs Erl+Ona
0.87 (0.63, 1.20)
0.48 (0.09, 2.36)
Erl+Bev vs Erl+Sun
1.01 (0.76, 1.34)
0.68 (0.16, 2.70)
Erl+Bev vs Erl+Tiv
1.02 (0.78, 1.35)
0.38 (0.10, 1.39)
Erl+Bev vs Gef+Nim
1.09 (0.67, 1.79)
0.39 (0.09, 1.52)
Erl+Bev vs Gefitinib
0.95 (0.73, 1.23)
0.34 (0.09, 1.21)
Erl+Bev vs Icotinib
0.93 (0.65, 1.32)
0.33 (0.08, 1.23)
Erl+Bev vs Pem+Erl
1.32 (0.93, 1.91)
0.69 (0.15, 2.84)
Erl+Bev vs Pemetrexed
0.95 (0.72, 1.24)
0.36 (0.09, 1.30)
Erl+Bev vs Placebo
0.80 (0.61, 1.05)
0.23 (0.06, 0.84)
Erl+Bev vs Teg/ura+Gef
1.16 (0.65, 2.10)
0.41 (0.09, 1.68)
Erl+Bev vs Vandetanib
0.93 (0.70, 1.22)
0.33 (0.08, 1.18)
Erl+Ona vs Erl+Sun
1.16 (0.87, 1.56)
1.39 (0.49, 4.01)
Erl+Ona vs Erl+Tiv
1.17 (0.89, 1.57)
0.79 (0.30, 2.07)
Erl+Ona vs Gef+Nim
1.27 (0.76, 2.10)
0.81 (0.29, 2.27)
Erl+Ona vs Gefitinib
1.09 (0.83, 1.44)
0.69 (0.28, 1.74)
Erl+Ona vs Icotinib
1.06 (0.75, 1.54)
0.68 (0.26, 1.81)
Erl+Ona vs Pem+Erl
1.53 (1.06, 2.20)
1.43 (0.45, 4.68)
Erl+Ona vs Pemetrexed
1.09 (0.82, 1.44)
0.75 (0.30, 1.90)
Erl+Ona vs Placebo
0.92 (0.70, 1.22)
0.48 (0.19, 1.24)
Erl+Ona vs Teg/ura+Gef
1.34 (0.76, 2.48)
0.84 (0.29, 2.48)
Erl+Ona vs Vandetanib
1.07 (0.80, 1.42)
0.67 (0.26, 1.75)
Total population
Only Asian population
.2
.3
.5
1
2
5

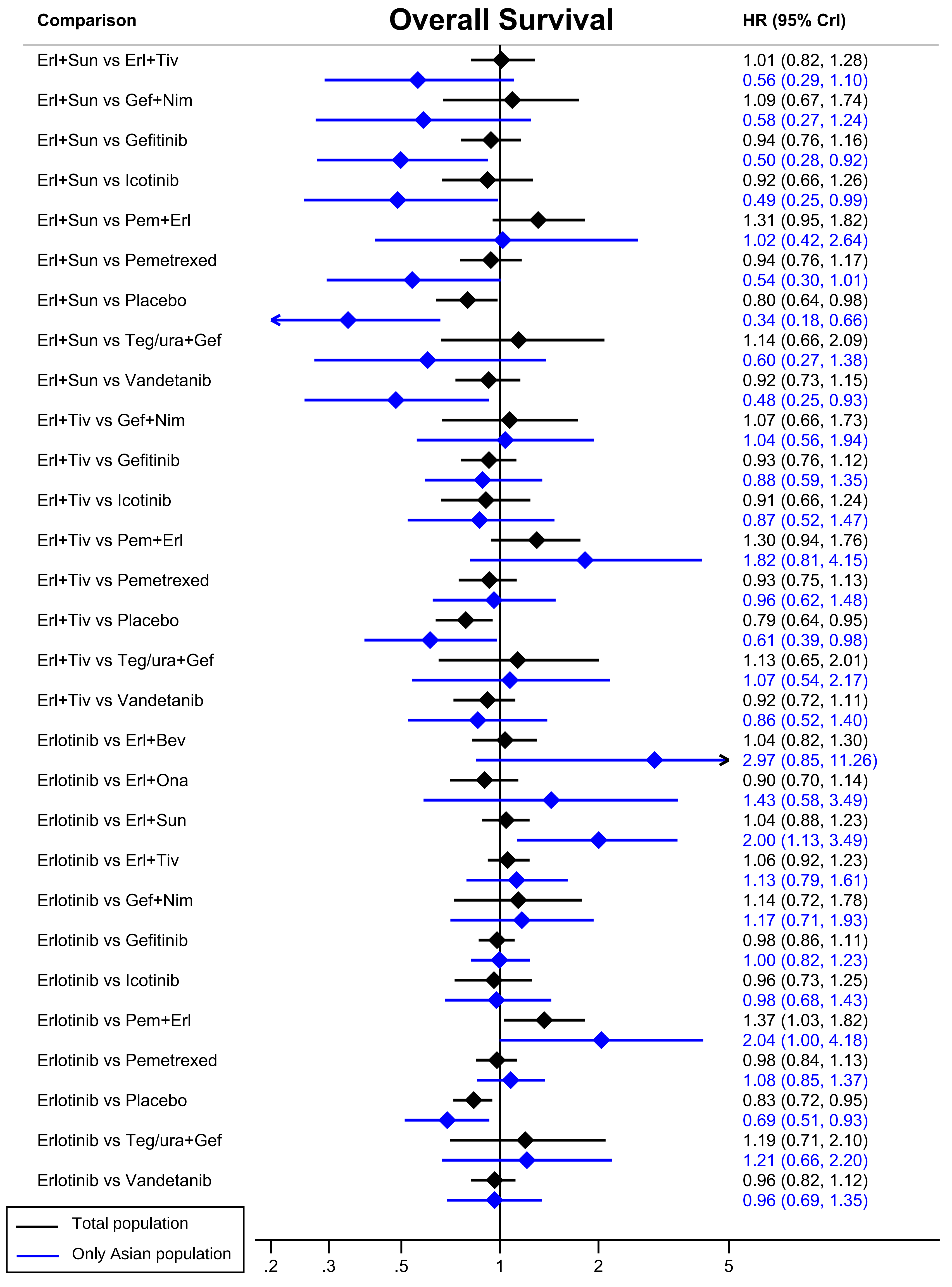
Comparison
Overall Survival
HR (95% CrI)
Erl+Sun vs Erl+Tiv
1.01 (0.82, 1.28)
0.56 (0.29, 1.10)
Erl+Sun vs Gef+Nim
1.09 (0.67, 1.74)
0.58 (0.27, 1.24)
Erl+Sun vs Gefitinib
0.94 (0.76, 1.16)
0.50 (0.28, 0.92)
Erl+Sun vs Icotinib
0.92 (0.66, 1.26)
0.49 (0.25, 0.99)
Erl+Sun vs Pem+Erl
1.31 (0.95, 1.82)
1.02 (0.42, 2.64)
Erl+Sun vs Pemetrexed
0.94 (0.76, 1.17)
0.54 (0.30, 1.01)
Erl+Sun vs Placebo
0.80 (0.64, 0.98)
0.34 (0.18, 0.66)
Erl+Sun vs Teg/ura+Gef
1.14 (0.66, 2.09)
0.60 (0.27, 1.38)
Erl+Sun vs Vandetanib
0.92 (0.73, 1.15)
0.48 (0.25, 0.93)
Erl+Tiv vs Gef+Nim
1.07 (0.66, 1.73)
1.04 (0.56, 1.94)
Erl+Tiv vs Gefitinib
0.93 (0.76, 1.12)
0.88 (0.59, 1.35)
Erl+Tiv vs Icotinib
0.91 (0.66, 1.24)
0.87 (0.52, 1.47)
Erl+Tiv vs Pem+Erl
1.30 (0.94, 1.76)
1.82 (0.81, 4.15)
Erl+Tiv vs Pemetrexed
0.93 (0.75, 1.13)
0.96 (0.62, 1.48)
Erl+Tiv vs Placebo
0.79 (0.64, 0.95)
0.61 (0.39, 0.98)
Erl+Tiv vs Teg/ura+Gef
1.13 (0.65, 2.01)
1.07 (0.54, 2.17)
Erl+Tiv vs Vandetanib
0.92 (0.72, 1.11)
0.86 (0.52, 1.40)
Erlotinib vs Erl+Bev
1.04 (0.82, 1.30)
2.97 (0.85, 11.26)
Erlotinib vs Erl+Ona
0.90 (0.70, 1.14)
1.43 (0.58, 3.49)
Erlotinib vs Erl+Sun
1.04 (0.88, 1.23)
2.00 (1.13, 3.49)
Erlotinib vs Erl+Tiv
1.06 (0.92, 1.23)
1.13 (0.79, 1.61)
Erlotinib vs Gef+Nim
1.14 (0.72, 1.78)
1.17 (0.71, 1.93)
Erlotinib vs Gefitinib
0.98 (0.86, 1.11)
1.00 (0.82, 1.23)
Erlotinib vs Icotinib
0.96 (0.73, 1.25)
0.98 (0.68, 1.43)
Erlotinib vs Pem+Erl
1.37 (1.03, 1.82)
2.04 (1.00, 4.18)
Erlotinib vs Pemetrexed
0.98 (0.84, 1.13)
1.08 (0.85, 1.37)
Erlotinib vs Placebo
0.83 (0.72, 0.95)
0.69 (0.51, 0.93)
Erlotinib vs Teg/ura+Gef
1.19 (0.71, 2.10)
1.21 (0.66, 2.20)
Erlotinib vs Vandetanib
0.96 (0.82, 1.12)
0.96 (0.69, 1.35)
Total population
Only Asian population
.2
.3
.5
1
2
5

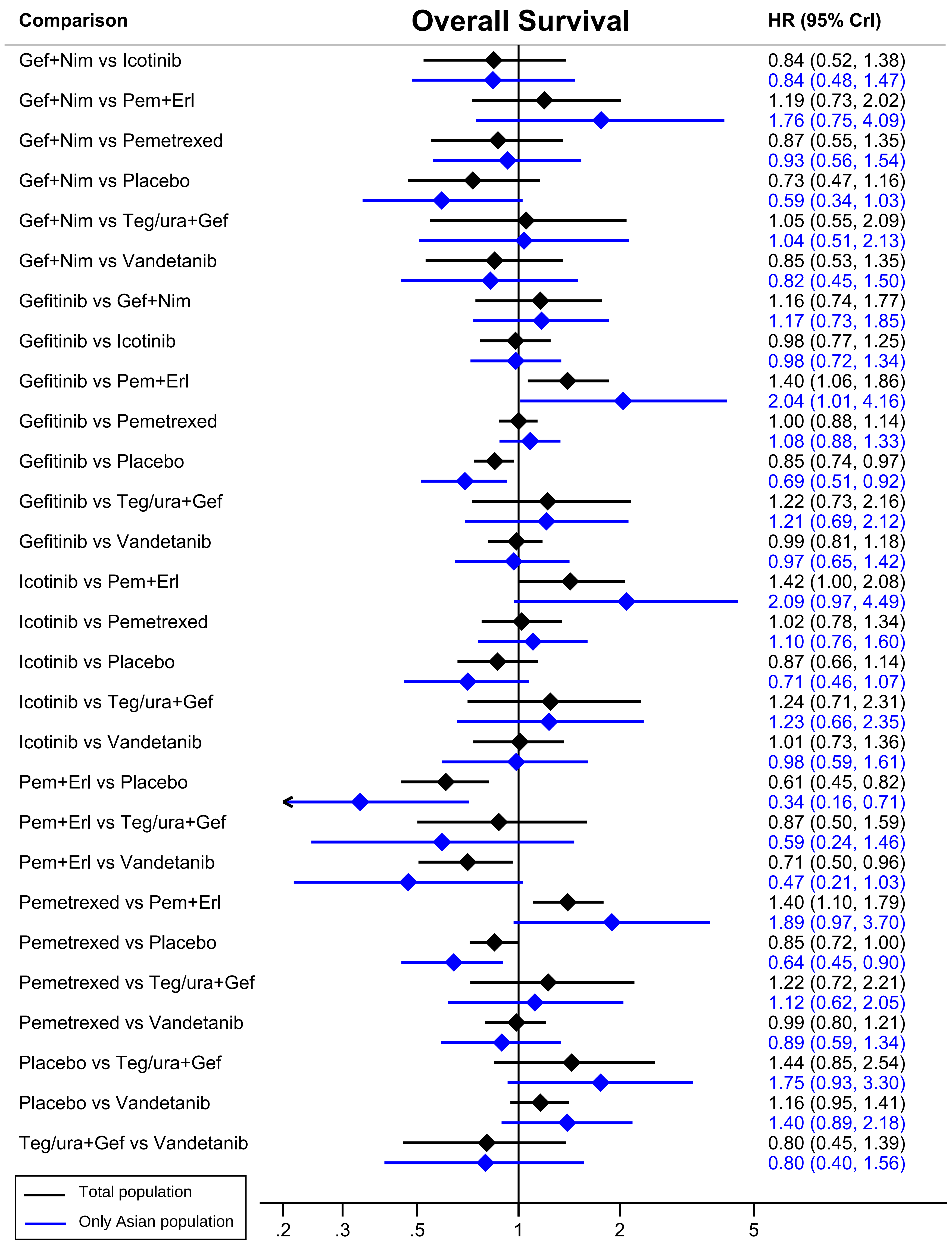
Comparison
Overall Survival
HR (95% CrI)
Gef+Nim vs Icotinib
0.84 (0.52, 1.38)
0.84 (0.48, 1.47)
Gef+Nim vs Pem+Erl
1.19 (0.73, 2.02)
1.76 (0.75, 4.09)
Gef+Nim vs Pemetrexed
0.87 (0.55, 1.35)
0.93 (0.56, 1.54)
Gef+Nim vs Placebo
0.73 (0.47, 1.16)
0.59 (0.34, 1.03)
Gef+Nim vs Teg/ura+Gef
1.05 (0.55, 2.09)
1.04 (0.51, 2.13)
Gef+Nim vs Vandetanib
0.85 (0.53, 1.35)
0.82 (0.45, 1.50)
Gefitinib vs Gef+Nim
1.16 (0.74, 1.77)
1.17 (0.73, 1.85)
Gefitinib vs Icotinib
0.98 (0.77, 1.25)
0.98 (0.72, 1.34)
Gefitinib vs Pem+Erl
1.40 (1.06, 1.86)
2.04 (1.01, 4.16)
Gefitinib vs Pemetrexed
1.00 (0.88, 1.14)
1.08 (0.88, 1.33)
Gefitinib vs Placebo
0.85 (0.74, 0.97)
0.69 (0.51, 0.92)
Gefitinib vs Teg/ura+Gef
1.22 (0.73, 2.16)
1.21 (0.69, 2.12)
Gefitinib vs Vandetanib
0.99 (0.81, 1.18)
0.97 (0.65, 1.42)
Icotinib vs Pem+Erl
1.42 (1.00, 2.08)
2.09 (0.97, 4.49)
Icotinib vs Pemetrexed
1.02 (0.78, 1.34)
1.10 (0.76, 1.60)
Icotinib vs Placebo
0.87 (0.66, 1.14)
0.71 (0.46, 1.07)
Icotinib vs Teg/ura+Gef
1.24 (0.71, 2.31)
1.23 (0.66, 2.35)
Icotinib vs Vandetanib
1.01 (0.73, 1.36)
0.98 (0.59, 1.61)
Pem+Erl vs Placebo
0.61 (0.45, 0.82)
0.34 (0.16, 0.71)
Pem+Erl vs Teg/ura+Gef
0.87 (0.50, 1.59)
0.59 (0.24, 1.46)
Pem+Erl vs Vandetanib
0.71 (0.50, 0.96)
0.47 (0.21, 1.03)
Pemetrexed vs Pem+Erl
1.40 (1.10, 1.79)
1.89 (0.97, 3.70)
Pemetrexed vs Placebo
0.85 (0.72, 1.00)
0.64 (0.45, 0.90)
Pemetrexed vs Teg/ura+Gef
1.22 (0.72, 2.21)
1.12 (0.62, 2.05)
Pemetrexed vs Vandetanib
0.99 (0.80, 1.21)
0.89 (0.59, 1.34)
Placebo vs Teg/ura+Gef
1.44 (0.85, 2.54)
1.75 (0.93, 3.30)
Placebo vs Vandetanib
1.16 (0.95, 1.41)
1.40 (0.89, 2.18)
Teg/ura+Gef vs Vandetanib
0.80 (0.45, 1.39)
0.80 (0.40, 1.56)
Total population
Only Asian population
.2
.3
.5
1
2
5

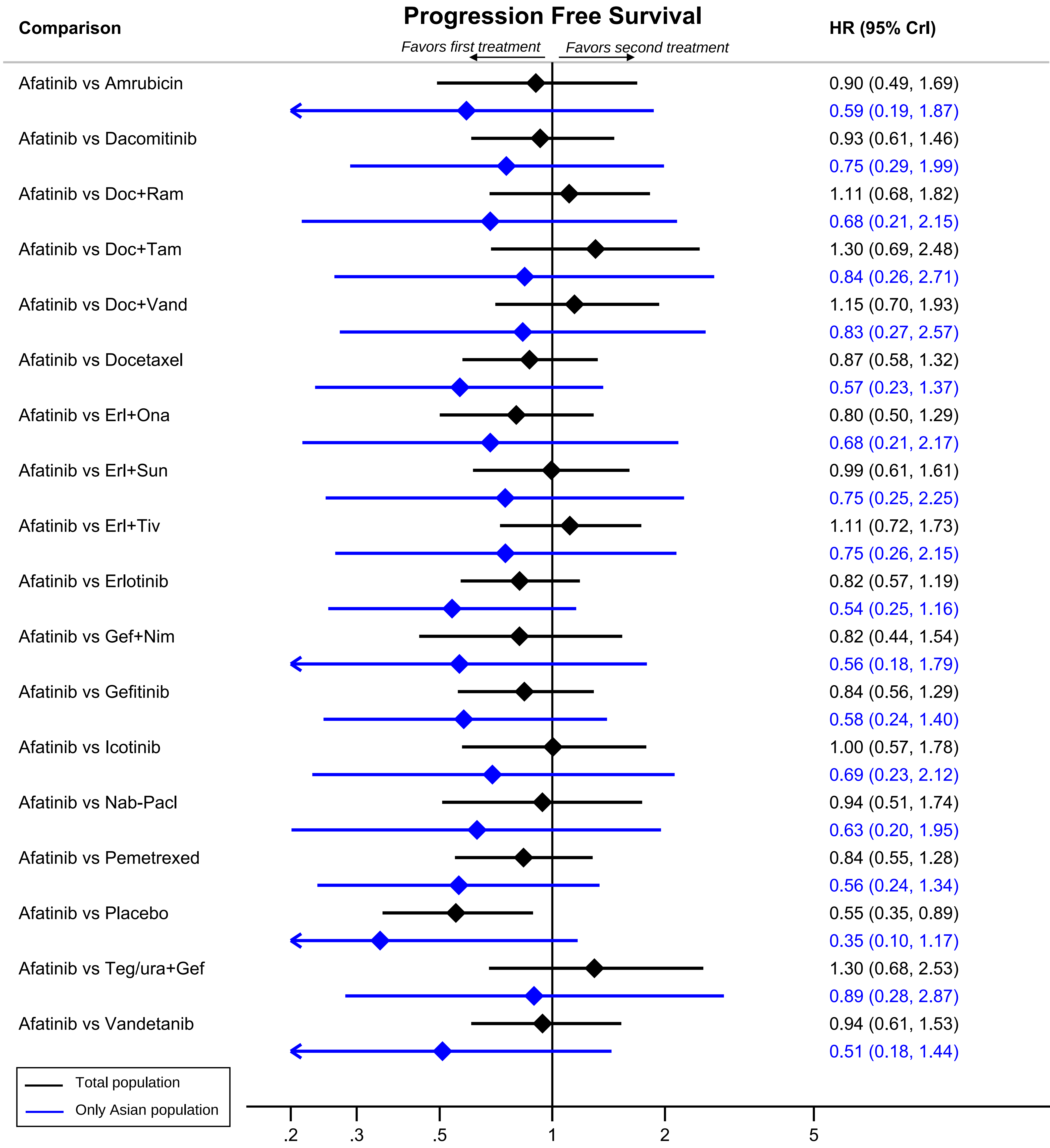
Progression Free Survival
Comparison
HR (95% CrI)
Favors first treatment
Favors second treatment
Afatinib vs Amrubicin
0.90 (0.49, 1.69)
0.59 (0.19, 1.87)
Afatinib vs Dacomitinib
0.93 (0.61, 1.46)
0.75 (0.29, 1.99)
Afatinib vs Doc+Ram
1.11 (0.68, 1.82)
0.68 (0.21, 2.15)
Afatinib vs Doc+Tam
1.30 (0.69, 2.48)
0.84 (0.26, 2.71)
Afatinib vs Doc+Vand
1.15 (0.70, 1.93)
0.83 (0.27, 2.57)
Afatinib vs Docetaxel
0.87 (0.58, 1.32)
0.57 (0.23, 1.37)
Afatinib vs Erl+Ona
0.80 (0.50, 1.29)
0.68 (0.21, 2.17)
Afatinib vs Erl+Sun
0.99 (0.61, 1.61)
0.75 (0.25, 2.25)
Afatinib vs Erl+Tiv
1.11 (0.72, 1.73)
0.75 (0.26, 2.15)
Afatinib vs Erlotinib
0.82 (0.57, 1.19)
0.54 (0.25, 1.16)
Afatinib vs Gef+Nim
0.82 (0.44, 1.54)
0.56 (0.18, 1.79)
Afatinib vs Gefitinib
0.84 (0.56, 1.29)
0.58 (0.24, 1.40)
Afatinib vs Icotinib
1.00 (0.57, 1.78)
0.69 (0.23, 2.12)
Afatinib vs Nab-Pacl
0.94 (0.51, 1.74)
0.63 (0.20, 1.95)
Afatinib vs Pemetrexed
0.84 (0.55, 1.28)
0.56 (0.24, 1.34)
Afatinib vs Placebo
0.55 (0.35, 0.89)
0.35 (0.10, 1.17)
Afatinib vs Teg/ura+Gef
1.30 (0.68, 2.53)
0.89 (0.28, 2.87)
Afatinib vs Vandetanib
0.94 (0.61, 1.53)
0.51 (0.18, 1.44)
Total population
Only Asian population
.2
.3
.5
1
2
5

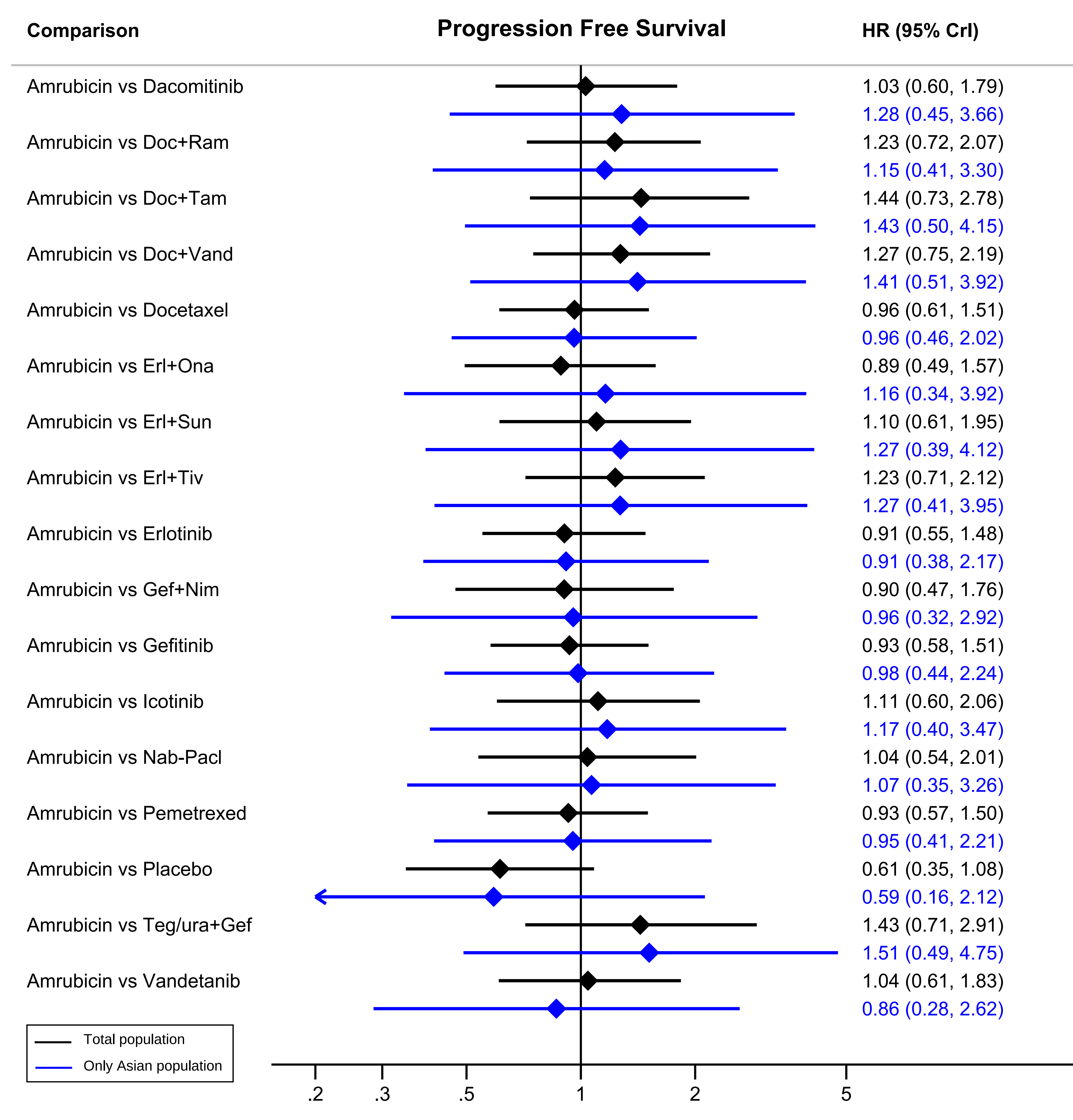
Comparison
Progression Free Survival
HR (95% CrI)
Amrubicin vs Dacomitinib
1.03 (0.60, 1.79)
1.28 (0.45, 3.66)
Amrubicin vs Doc+Ram
1.23 (0.72, 2.07)
1.15 (0.41, 3.30)
Amrubicin vs Doc+Tam
1.44 (0.73, 2.78)
1.43 (0.50, 4.15)
Amrubicin vs Doc+Vand
1.27 (0.75, 2.19)
1.41 (0.51, 3.92)
Amrubicin vs Docetaxel
0.96 (0.61, 1.51)
0.96 (0.46, 2.02)
Amrubicin vs Erl+Ona
0.89 (0.49, 1.57)
1.16 (0.34, 3.92)
Amrubicin vs Erl+Sun
1.10 (0.61, 1.95)
1.27 (0.39, 4.12)
Amrubicin vs Erl+Tiv
1.23 (0.71, 2.12)
1.27 (0.41, 3.95)
Amrubicin vs Erlotinib
0.91 (0.55, 1.48)
0.91 (0.38, 2.17)
Amrubicin vs Gef+Nim
0.90 (0.47, 1.76)
0.96 (0.32, 2.92)
Amrubicin vs Gefitinib
0.93 (0.58, 1.51)
0.98 (0.44, 2.24)
Amrubicin vs Icotinib
1.11 (0.60, 2.06)
1.17 (0.40, 3.47)
Amrubicin vs Nab-Pacl
1.04 (0.54, 2.01)
1.07 (0.35, 3.26)
Amrubicin vs Pemetrexed
0.93 (0.57, 1.50)
0.95 (0.41, 2.21)
Amrubicin vs Placebo
0.61 (0.35, 1.08)
0.59 (0.16, 2.12)
Amrubicin vs Teg/ura+Gef
1.43 (0.71, 2.91)
1.51 (0.49, 4.75)
Amrubicin vs Vandetanib
1.04 (0.61, 1.83)
0.86 (0.28, 2.62)
Total population
Only Asian population
.2
.3
.5
1
2
5

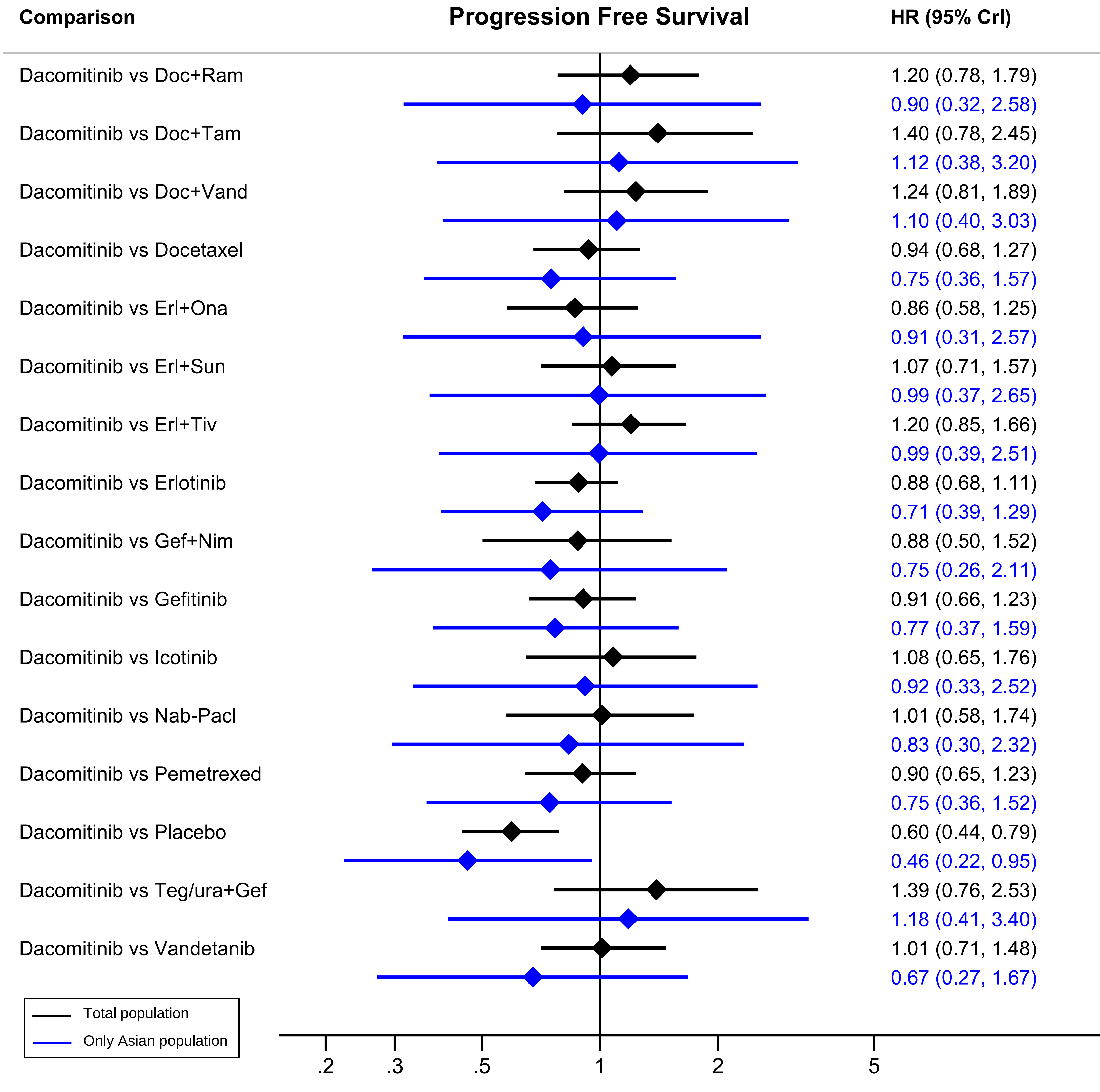
Comparison
Progression Free Survival
HR (95% CrI)
Dacomitinib vs Doc+Ram
1.20 (0.78, 1.79)
0.90 (0.32, 2.58)
Dacomitinib vs Doc+Tam
1.40 (0.78, 2.45)
1.12 (0.38, 3.20)
Dacomitinib vs Doc+Vand
1.24 (0.81, 1.89)
1.10 (0.40, 3.03)
Dacomitinib vs Docetaxel
0.94 (0.68, 1.27)
0.75 (0.36, 1.57)
Dacomitinib vs Erl+Ona
0.86 (0.58, 1.25)
0.91 (0.31, 2.57)
Dacomitinib vs Erl+Sun
1.07 (0.71, 1.57)
0.99 (0.37, 2.65)
Dacomitinib vs Erl+Tiv
1.20 (0.85, 1.66)
0.99 (0.39, 2.51)
Dacomitinib vs Erlotinib
0.88 (0.68, 1.11)
0.71 (0.39, 1.29)
Dacomitinib vs Gef+Nim
0.88 (0.50, 1.52)
0.75 (0.26, 2.11)
Dacomitinib vs Gefitinib
0.91 (0.66, 1.23)
0.77 (0.37, 1.59)
Dacomitinib vs Icotinib
1.08 (0.65, 1.76)
0.92 (0.33, 2.52)
Dacomitinib vs Nab-Pacl
1.01 (0.58, 1.74)
0.83 (0.30, 2.32)
Dacomitinib vs Pemetrexed
0.90 (0.65, 1.23)
0.75 (0.36, 1.52)
Dacomitinib vs Placebo
0.60 (0.44, 0.79)
0.46 (0.22, 0.95)
Dacomitinib vs Teg/ura+Gef
1.39 (0.76, 2.53)
1.18 (0.41, 3.40)
Dacomitinib vs Vandetanib
1.01 (0.71, 1.48)
0.67 (0.27, 1.67)
Total population
Only Asian population
.2
.3
.5
1
2
5

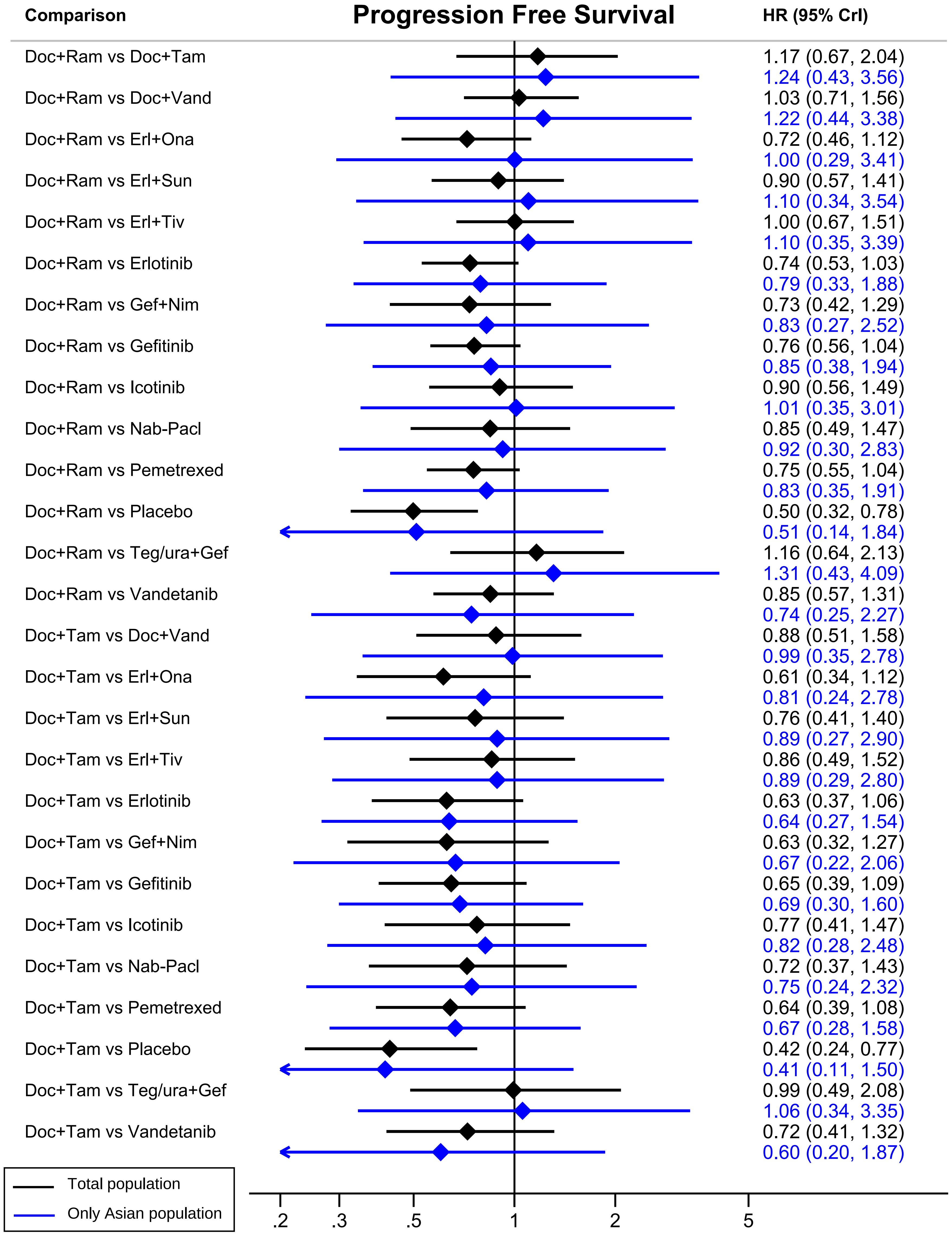
Comparison
Progression Free Survival
HR (95% CrI)
Doc+Ram vs Doc+Tam
1.17 (0.67, 2.04)
1.24 (0.43, 3.56)
Doc+Ram vs Doc+Vand
1.03 (0.71, 1.56)
1.22 (0.44, 3.38)
Doc+Ram vs Erl+Ona
0.72 (0.46, 1.12)
1.00 (0.29, 3.41)
Doc+Ram vs Erl+Sun
0.90 (0.57, 1.41)
1.10 (0.34, 3.54)
Doc+Ram vs Erl+Tiv
1.00 (0.67, 1.51)
1.10 (0.35, 3.39)
Doc+Ram vs Erlotinib
0.74 (0.53, 1.03)
0.79 (0.33, 1.88)
Doc+Ram vs Gef+Nim
0.73 (0.42, 1.29)
0.83 (0.27, 2.52)
Doc+Ram vs Gefitinib
0.76 (0.56, 1.04)
0.85 (0.38, 1.94)
Doc+Ram vs Icotinib
0.90 (0.56, 1.49)
1.01 (0.35, 3.01)
Doc+Ram vs Nab-Pacl
0.85 (0.49, 1.47)
0.92 (0.30, 2.83)
Doc+Ram vs Pemetrexed
0.75 (0.55, 1.04)
0.83 (0.35, 1.91)
Doc+Ram vs Placebo
0.50 (0.32, 0.78)
0.51 (0.14, 1.84)
Doc+Ram vs Teg/ura+Gef
1.16 (0.64, 2.13)
1.31 (0.43, 4.09)
Doc+Ram vs Vandetanib
0.85 (0.57, 1.31)
0.74 (0.25, 2.27)
Doc+Tam vs Doc+Vand
0.88 (0.51, 1.58)
0.99 (0.35, 2.78)
Doc+Tam vs Erl+Ona
0.61 (0.34, 1.12)
0.81 (0.24, 2.78)
Doc+Tam vs Erl+Sun
0.76 (0.41, 1.40)
0.89 (0.27, 2.90)
Doc+Tam vs Erl+Tiv
0.86 (0.49, 1.52)
0.89 (0.29, 2.80)
Doc+Tam vs Erlotinib
0.63 (0.37, 1.06)
0.64 (0.27, 1.54)
Doc+Tam vs Gef+Nim
0.63 (0.32, 1.27)
0.67 (0.22, 2.06)
Doc+Tam vs Gefitinib
0.65 (0.39, 1.09)
0.69 (0.30, 1.60)
Doc+Tam vs Icotinib
0.77 (0.41, 1.47)
0.82 (0.28, 2.48)
Doc+Tam vs Nab-Pacl
0.72 (0.37, 1.43)
0.75 (0.24, 2.32)
Doc+Tam vs Pemetrexed
0.64 (0.39, 1.08)
0.67 (0.28, 1.58)
Doc+Tam vs Placebo
0.42 (0.24, 0.77)
0.41 (0.11, 1.50)
Doc+Tam vs Teg/ura+Gef
0.99 (0.49, 2.08)
1.06 (0.34, 3.35)
Doc+Tam vs Vandetanib
0.72 (0.41, 1.32)
0.60 (0.20, 1.87)
Total population
Only Asian population
.2
.3
.5
1
2
5

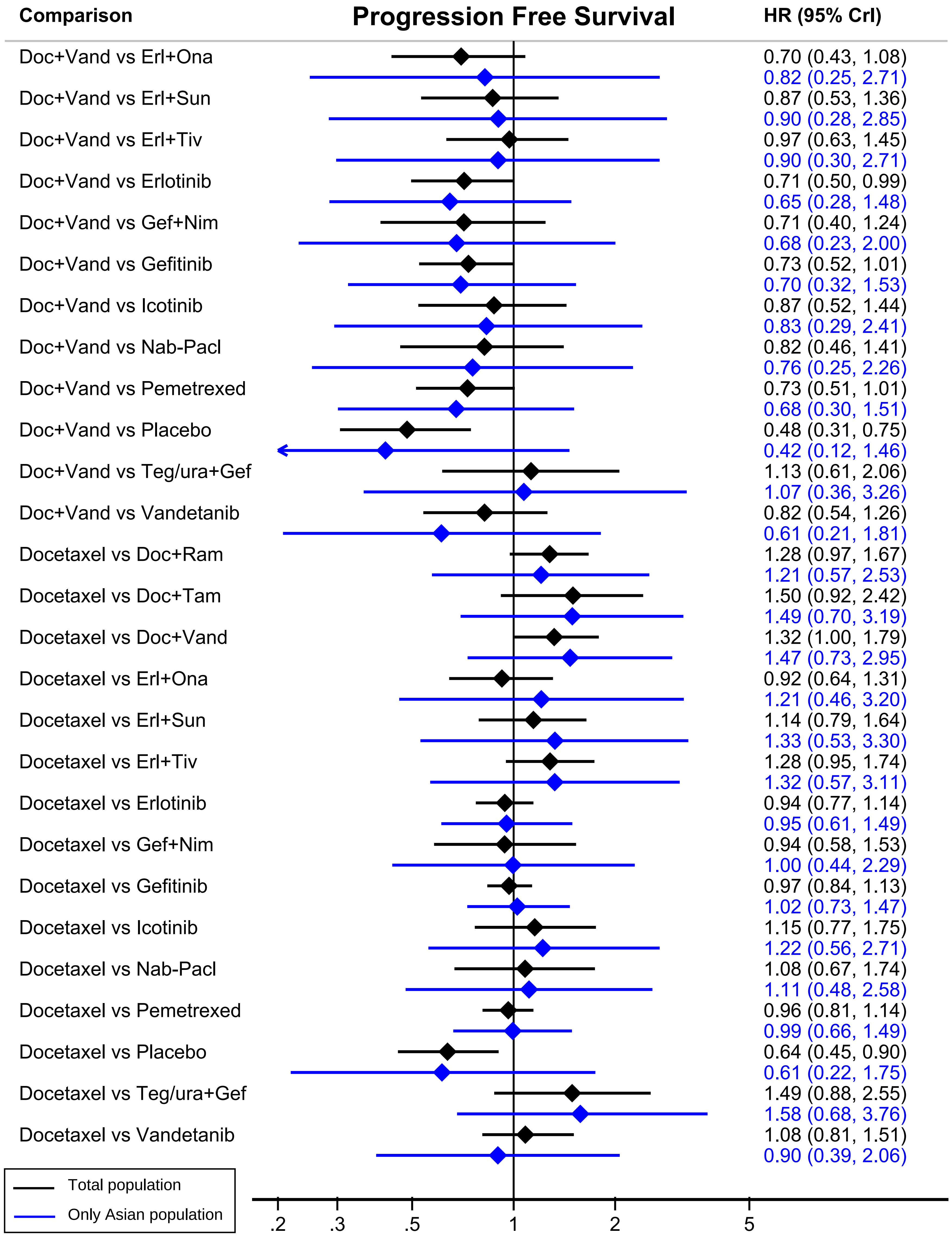

Comparison
Progression Free Survival
HR (95% CrI)
Doc+Vand vs Erl+Ona
0.70 (0.43, 1.08)
0.82 (0.25, 2.71)
Doc+Vand vs Erl+Sun
0.87 (0.53, 1.36)
0.90 (0.28, 2.85)
Doc+Vand vs Erl+Tiv
0.97 (0.63, 1.45)
0.90 (0.30, 2.71)
Doc+Vand vs Erlotinib
0.71 (0.50, 0.99)
0.65 (0.28, 1.48)
Doc+Vand vs Gef+Nim
0.71 (0.40, 1.24)
0.68 (0.23, 2.00)
Doc+Vand vs Gefitinib
0.73 (0.52, 1.01)
0.70 (0.32, 1.53)
Doc+Vand vs Icotinib
0.87 (0.52, 1.44)
0.83 (0.29, 2.41)
Doc+Vand vs Nab-Pacl
0.82 (0.46, 1.41)
0.76 (0.25, 2.26)
Doc+Vand vs Pemetrexed
0.73 (0.51, 1.01)
0.68 (0.30, 1.51)
Doc+Vand vs Placebo
0.48 (0.31, 0.75)
0.42 (0.12, 1.46)
Doc+Vand vs Teg/ura+Gef
1.13 (0.61, 2.06)
1.07 (0.36, 3.26)
Doc+Vand vs Vandetanib
0.82 (0.54, 1.26)
0.61 (0.21, 1.81)
Docetaxel vs Doc+Ram
1.28 (0.97, 1.67)
1.21 (0.57, 2.53)
Docetaxel vs Doc+Tam
1.50 (0.92, 2.42)
1.49 (0.70, 3.19)
Docetaxel vs Doc+Vand
1.32 (1.00, 1.79)
1.47 (0.73, 2.95)
Docetaxel vs Erl+Ona
0.92 (0.64, 1.31)
1.21 (0.46, 3.20)
Docetaxel vs Erl+Sun
1.14 (0.79, 1.64)
1.33 (0.53, 3.30)
Docetaxel vs Erl+Tiv
1.28 (0.95, 1.74)
1.32 (0.57, 3.11)
Docetaxel vs Erlotinib
0.94 (0.77, 1.14)
0.95 (0.61, 1.49)
Docetaxel vs Gef+Nim
0.94 (0.58, 1.53)
1.00 (0.44, 2.29)
Docetaxel vs Gefitinib
0.97 (0.84, 1.13)
1.02 (0.73, 1.47)
Docetaxel vs Icotinib
1.15 (0.77, 1.75)
1.22 (0.56, 2.71)
Docetaxel vs Nab-Pacl
1.08 (0.67, 1.74)
1.11 (0.48, 2.58)
Docetaxel vs Pemetrexed
0.96 (0.81, 1.14)
0.99 (0.66, 1.49)
Docetaxel vs Placebo
0.64 (0.45, 0.90)
0.61 (0.22, 1.75)
Docetaxel vs Teg/ura+Gef
1.49 (0.88, 2.55)
1.58 (0.68, 3.76)
Docetaxel vs Vandetanib
1.08 (0.81, 1.51)
0.90 (0.39, 2.06)
Total population
Only Asian population
.2
.3
.5
1
2
5

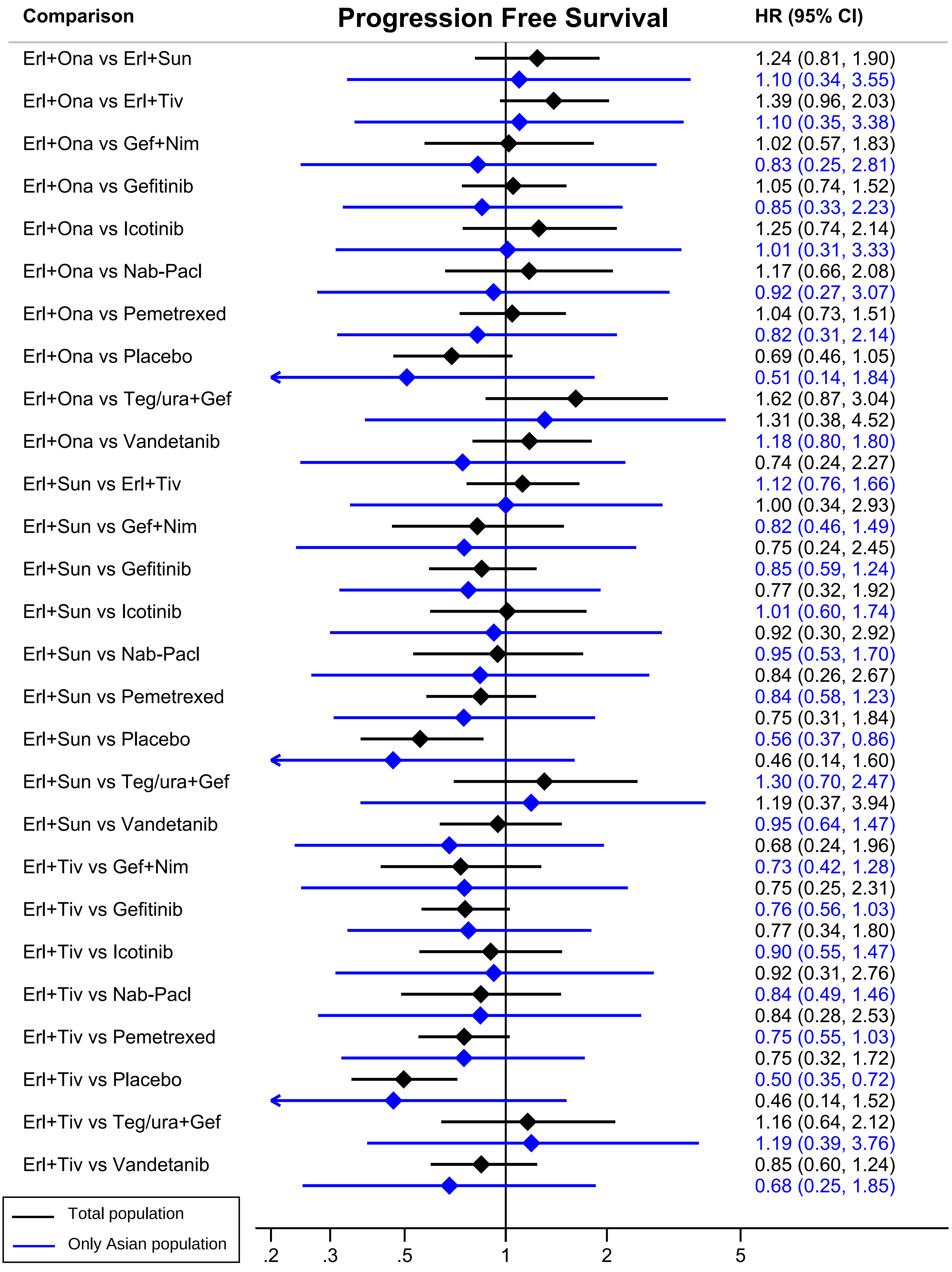
Comparison
Progression Free Survival
HR (95% CI)
Erl+Ona vs Erl+Sun
Erl+Ona vs Erl+Tiv
Erl+Ona vs Gef+Nim
Erl+Ona vs Gefitinib
Erl+Ona vs Icotinib
Erl+Ona vs Nab-Pacl
Erl+Ona vs Pemetrexed
Erl+Ona vs Placebo
Erl+Ona vs Teg/ura+Gef
Erl+Ona vs Vandetanib
Erl+Sun vs Erl+Tiv
Erl+Sun vs Gef+Nim
Erl+Sun vs Gefitinib
Erl+Sun vs Icotinib
Erl+Sun vs Nab-Pacl
Erl+Sun vs Pemetrexed
Erl+Sun vs Placebo
Erl+Sun vs Teg/ura+Gef
Erl+Sun vs Vandetanib
Erl+Tiv vs Gef+Nim
Erl+Tiv vs Gefitinib
Erl+Tiv vs Icotinib
Erl+Tiv vs Nab-Pacl
Erl+Tiv vs Pemetrexed
Erl+Tiv vs Placebo
Erl+Tiv vs Teg/ura+Gef
Erl+Tiv vs Vandetanib
1.24 (0.81, 1.90)
1.10 (0.34, 3.55)
1.39 (0.96, 2.03)
1.10 (0.35, 3.38)
1.02 (0.57, 1.83)
0.83 (0.25, 2.81)
1.05 (0.74, 1.52)
0.85 (0.33, 2.23)
1.25 (0.74, 2.14)
1.01 (0.31, 3.33)
1.17 (0.66, 2.08)
0.92 (0.27, 3.07)
1.04 (0.73, 1.51)
0.82 (0.31, 2.14)
0.69 (0.46, 1.05)
0.51 (0.14, 1.84)
1.62 (0.87, 3.04)
1.31 (0.38, 4.52)
1.18 (0.80, 1.80)
0.74 (0.24, 2.27)
1.12 (0.76, 1.66)
1.00 (0.34, 2.93)
0.82 (0.46, 1.49)
0.75 (0.24, 2.45)
0.85 (0.59, 1.24)
0.77 (0.32, 1.92)
1.01 (0.60, 1.74)
0.92 (0.30, 2.92)
0.95 (0.53, 1.70)
0.84 (0.26, 2.67)
0.84 (0.58, 1.23)
0.75 (0.31, 1.84)
0.56 (0.37, 0.86)
0.46 (0.14, 1.60)
1.30 (0.70, 2.47)
1.19 (0.37, 3.94)
0.95 (0.64, 1.47)
0.68 (0.24, 1.96)
0.73 (0.42, 1.28)
0.75 (0.25, 2.31)
0.76 (0.56, 1.03)
0.77 (0.34, 1.80)
0.90 (0.55, 1.47)
0.92 (0.31, 2.76)
0.84 (0.49, 1.46)
0.84 (0.28, 2.53)
0.75 (0.55, 1.03)
0.75 (0.32, 1.72)
0.50 (0.35, 0.72)
0.46 (0.14, 1.52)
1.16 (0.64, 2.12)
1.19 (0.39, 3.76)
0.85 (0.60, 1.24)
0.68 (0.25, 1.85)
Total population
Only Asian population
.2
.3
.5
1
2
5

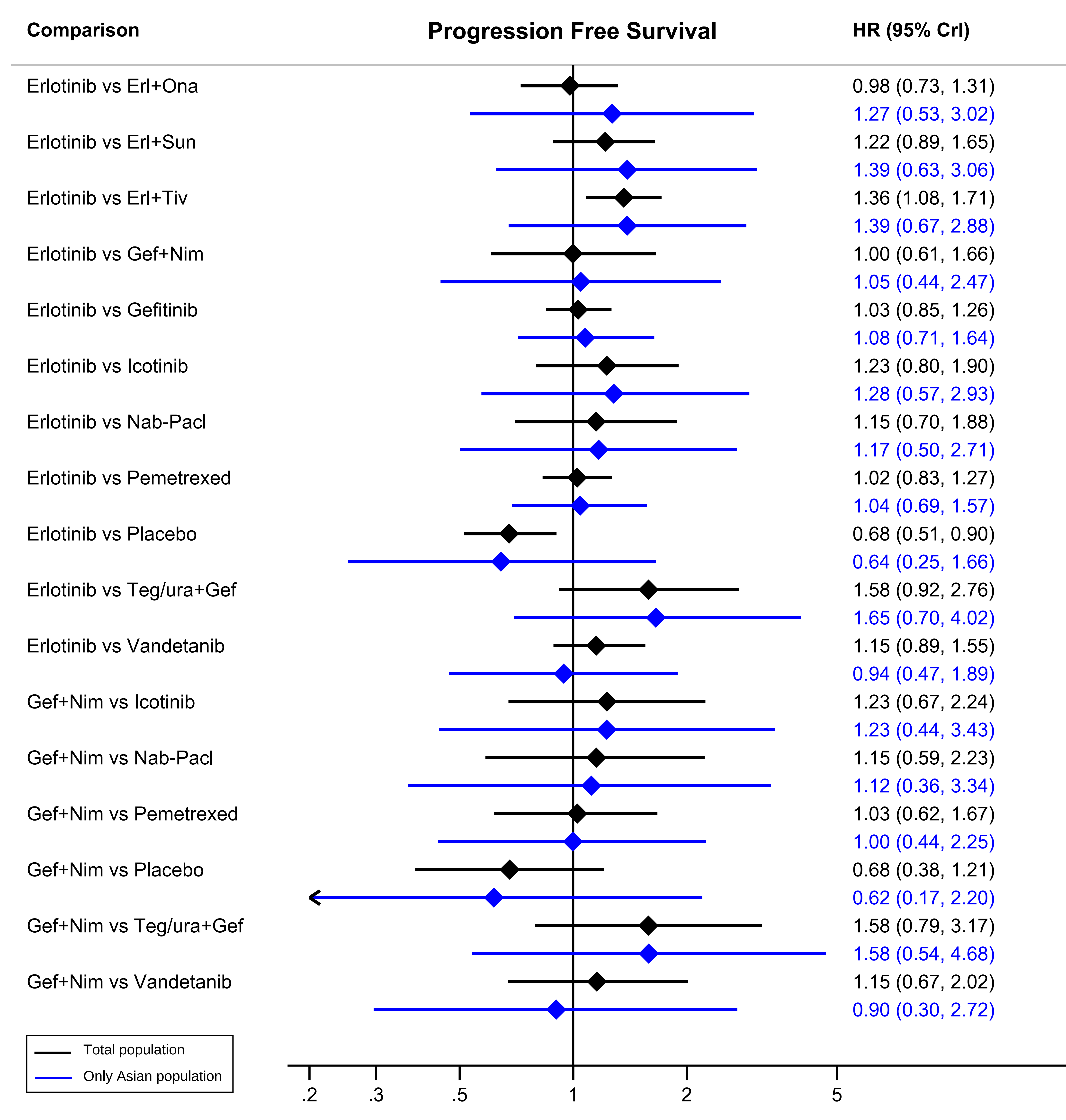
Comparison
Progression Free Survival
HR (95% CrI)
Erlotinib vs Erl+Ona
0.98 (0.73, 1.31)
1.27 (0.53, 3.02)
Erlotinib vs Erl+Sun
1.22 (0.89, 1.65)
1.39 (0.63, 3.06)
Erlotinib vs Erl+Tiv
1.36 (1.08, 1.71)
1.39 (0.67, 2.88)
Erlotinib vs Gef+Nim
1.00 (0.61, 1.66)
1.05 (0.44, 2.47)
Erlotinib vs Gefitinib
1.03 (0.85, 1.26)
1.08 (0.71, 1.64)
Erlotinib vs Icotinib
1.23 (0.80, 1.90)
1.28 (0.57, 2.93)
Erlotinib vs Nab-Pacl
1.15 (0.70, 1.88)
1.17 (0.50, 2.71)
Erlotinib vs Pemetrexed
1.02 (0.83, 1.27)
1.04 (0.69, 1.57)
Erlotinib vs Placebo
0.68 (0.51, 0.90)
0.64 (0.25, 1.66)
Erlotinib vs Teg/ura+Gef
1.58 (0.92, 2.76)
1.65 (0.70, 4.02)
Erlotinib vs Vandetanib
1.15 (0.89, 1.55)
0.94 (0.47, 1.89)
Gef+Nim vs Icotinib
1.23 (0.67, 2.24)
1.23 (0.44, 3.43)
Gef+Nim vs Nab-Pacl
1.15 (0.59, 2.23)
1.12 (0.36, 3.34)
Gef+Nim vs Pemetrexed
1.03 (0.62, 1.67)
1.00 (0.44, 2.25)
Gef+Nim vs Placebo
0.68 (0.38, 1.21)
0.62 (0.17, 2.20)
Gef+Nim vs Teg/ura+Gef
1.58 (0.79, 3.17)
1.58 (0.54, 4.68)
Gef+Nim vs Vandetanib
1.15 (0.67, 2.02)
0.90 (0.30, 2.72)
Total population
Only Asian population
.2
.3
.5
1
2
5

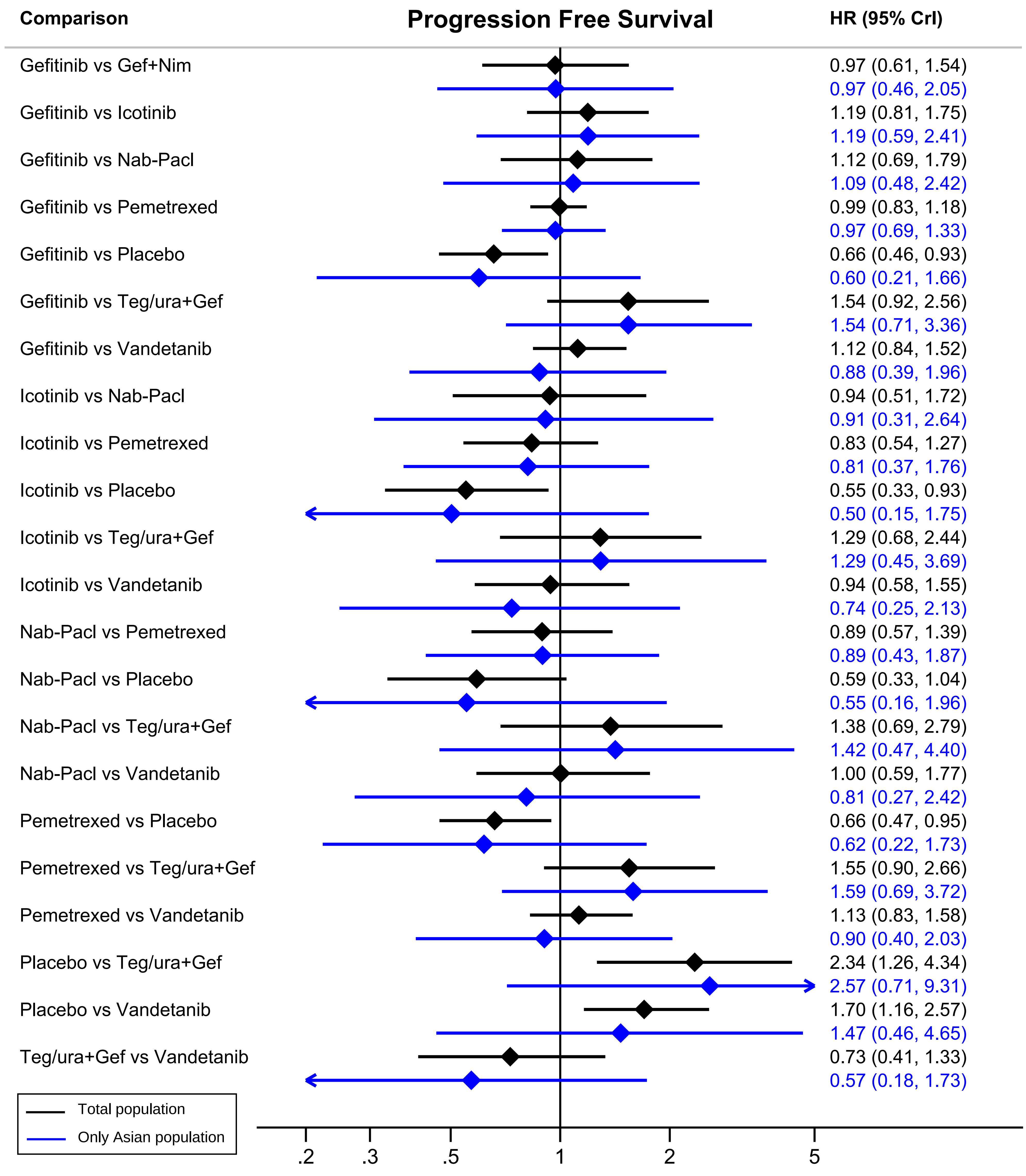
Comparison
Progression Free Survival
HR (95% CrI)
Gefitinib vs Gef+Nim
0.97 (0.61, 1.54)
0.97 (0.46, 2.05)
Gefitinib vs Icotinib
1.19 (0.81, 1.75)
1.19 (0.59, 2.41)
Gefitinib vs Nab-Pacl
1.12 (0.69, 1.79)
1.09 (0.48, 2.42)
Gefitinib vs Pemetrexed
0.99 (0.83, 1.18)
0.97 (0.69, 1.33)
Gefitinib vs Placebo
0.66 (0.46, 0.93)
0.60 (0.21, 1.66)
Gefitinib vs Teg/ura+Gef
1.54 (0.92, 2.56)
1.54 (0.71, 3.36)
Gefitinib vs Vandetanib
1.12 (0.84, 1.52)
0.88 (0.39, 1.96)
Icotinib vs Nab-Pacl
0.94 (0.51, 1.72)
0.91 (0.31, 2.64)
Icotinib vs Pemetrexed
0.83 (0.54, 1.27)
0.81 (0.37, 1.76)
Icotinib vs Placebo
0.55 (0.33, 0.93)
0.50 (0.15, 1.75)
Icotinib vs Teg/ura+Gef
1.29 (0.68, 2.44)
1.29 (0.45, 3.69)
Icotinib vs Vandetanib
0.94 (0.58, 1.55)
0.74 (0.25, 2.13)
Nab-Pacl vs Pemetrexed
0.89 (0.57, 1.39)
0.89 (0.43, 1.87)
Nab-Pacl vs Placebo
0.59 (0.33, 1.04)
0.55 (0.16, 1.96)
Nab-Pacl vs Teg/ura+Gef
1.38 (0.69, 2.79)
1.42 (0.47, 4.40)
Nab-Pacl vs Vandetanib
1.00 (0.59, 1.77)
0.81 (0.27, 2.42)
Pemetrexed vs Placebo
0.66 (0.47, 0.95)
0.62 (0.22, 1.73)
Pemetrexed vs Teg/ura+Gef
1.55 (0.90, 2.66)
1.59 (0.69, 3.72)
Pemetrexed vs Vandetanib
1.13 (0.83, 1.58)
0.90 (0.40, 2.03)
Placebo vs Teg/ura+Gef
2.34 (1.26, 4.34)
2.57 (0.71, 9.31)
Placebo vs Vandetanib
1.70 (1.16, 2.57)
1.47 (0.46, 4.65)
Teg/ura+Gef vs Vandetanib
0.73 (0.41, 1.33)
0.57 (0.18, 1.73)
Total population
Only Asian population
.2
.3
.5
1
2
5

SUCRA values for OS for second-line treatments assessed in squamous and nonsquamous NSCLC EGFR wild-type or unknown

Nonsquamous cell carcinoma

Treatment	SUCRA
Pembrolizumab	94.2
Atezolizumab	88.9
Nivolumab	87.6
Erlotinib+Cabozantinib	80.2
Docetaxel+Nintedanib	78.7
Docetaxel+Ramucirumab	78.7
Docetaxel+Vandetanib	72.8
Pralatrexate	67.5
Trametinib	64.5
Cabozantinib	62.9
Docetaxel	62.5
Amrubicin	61.9
Docetaxel+Aflibercept	61.5
Docetaxel+Selumetinib	60.7
Pemetrexed+Erlotinib	54.9
Pemetrexed+Vandetanib	41.1
Erlotinib+Tivantinib	39.6
Gefitinib+Tegafur-uracil	38.6
Erlotinib+Sunitinib	36.9
Dacomitinib	31.6
Vandetanib	31.3
Erlotinib	29.9
Pemetrexed+Cetuximab	28.2
Erlotinib+Bevacizumab	23.8
Pemetrexed+Nintedanib	20.8
Pemetrexed	20.6
Gefitinib	17.6
Erlotinib+Onartuzumab	07.8
Placebo	04.6

Squamous cell carcinoma

Treatment	SUCRA
Nivolumab	75.5
Atezolizumab	69.8
Pembrolizumab	65.8
Amrubicin	59.7
Afatinib	58.2
Docetaxel+Ramucirumab	57.5
Docetaxel+Erlotinib	53.5
Erlotinib+Onartuzumab	52.5
Docetaxel+Vandetanib	51.4
Erlotinib+Bevacizumab	50.5
Docetaxel	49.8
Docetaxel+Nintedanib	49.6
Erlotinib+Sunitinib	49.5
Erlotinib	45.5
Pralatrexate	43.0
Erlotinib+Figitumumab	40.6
Dacomitinib	36.7
Vandetanib	33.5
Placebo	25.7
Docetaxel+Selumetinib	24.9

SUCRA values for PFS for second-line treatments assessed in squamous and nonsquamous NSCLC EGFR wild-type or unknown

Nonsquamous cell carcinoma

Treatment	SUCRA
Erlotinib+Cabozantinib	89.8
Cabozantinib	88.7
Docetaxel+Vandetanib	74.2
Pralatrexate	71.3
Docetaxel+Ramucirumab	67.6
Docetaxel+Erlotinib	67.5
Pemetrexed+Erlotinib	63.4
Docetaxel+Aflibercept	62.6
Docetaxel+Nintedanib	60.4
Pembrolizumab	58.4
Erlotinib+Tivantinib	57.8
Gefitinib+Tegafur-uracil	54.7
Docetaxel+Selumetinib	54.3
Amrubicin	53.1
Nivolumab	52.3
Dacomitinib	51.0
Docetaxel	43.8
Erlotinib+Sunitinib	43.2
Vandetanib	40.9
Pemetrexed+Vandetanib	39.4
Pemetrexed+Nintedanib	35.2
Erlotinib+Onartuzumab	30.0
Icotinib	29.9
Trametinib	29.0
Pemetrexed+Cetuximab	25.1
Erlotinib	24.6
Pemetrexed	16.9
Gefitinib	15.3

Squamous cell carcinoma

Treatment	SUCRA
Nivolumab	79.5
Docetaxel+Vandetanib	71.4
Docetaxel+Ramucirumab	71.1
Docetaxel+Nintedanib	70.5
Amrubicin	64.6
Pembrolizumab	64.4
Docetaxel	56.3
Erlotinib+Sunitinib	44.2
Docetaxel+Selumetinib	43.6
Afatinib	42.7
Docetaxel+Erlotinib	39.9
Dacomitinib	35.5
Erlotinib	28.9
Erlotinib+Figitumumab	25.5
Vandetanib	25.4
Erlotinib+Onartuzumab	23.6

SUCRA values for OS and PFS for second-line treatments assessed in Asian population of NSCLC EGFR wild-type or unknown

OS

Treatment	SUCRA
Erlotinib+Onartuzumab	89.2
Erlotinib+Tivantinib	86.7
Pemetrexed+Nintedanib	86.2
Afatinib	78.4
Docetaxel+Tamoxifen	67.1
Pemetrexed	63.6
Erlotinib+Sunitinib	62.9
Docetaxel+Vandetanib	58.8
Tegafur/uracil+Gefitinib	53.3
Icotinib	50.6
Docetaxel+Ramucirumab	49.3
Gefitinib	47.4
Pemetrexed+Erlotinib	43.5
Pemetrexed+Vandetanib	43.2
Dacomitinib	32.7
Erlotinib	31.8
Gefitinib+Nimotuzumab	31.1
Docetaxel	30.9
Amrubicin	30.8
Nab-Paclitaxel	30.3
Vandetanib	28.9
Placebo	03.5

PFS

Treatment	SUCRA
Pemetrexed+Erlotinib	89.8
Afatinib	77.4
Tegafur/uracil+Gefitinib	70.7
Docetaxel+Tamoxifen	67.3
Docetaxel+Vandetanib	67.2
Dacomitinib	60.8
Erlotinib+Tivantinib	59.4
Erlotinib+Sunitinib	58.7
Icotinib	53.4
Docetaxel+Ramucirumab	52.0
Erlotinib+Onartuzumab	51.8
Pemetrexed+Vandetanib	47.0
Nab-Paclitaxel	45.9
Pemetrexed+Nintedanib	41.9
Amrubicin	40.8
Gefitinib	37.6
Gefitinib+Nimotuzumab	37.4
Docetaxel	34.8
Pemetrexed	34.2
Erlotinib	29.9
Vandetanib	29.3
Placebo	12.6

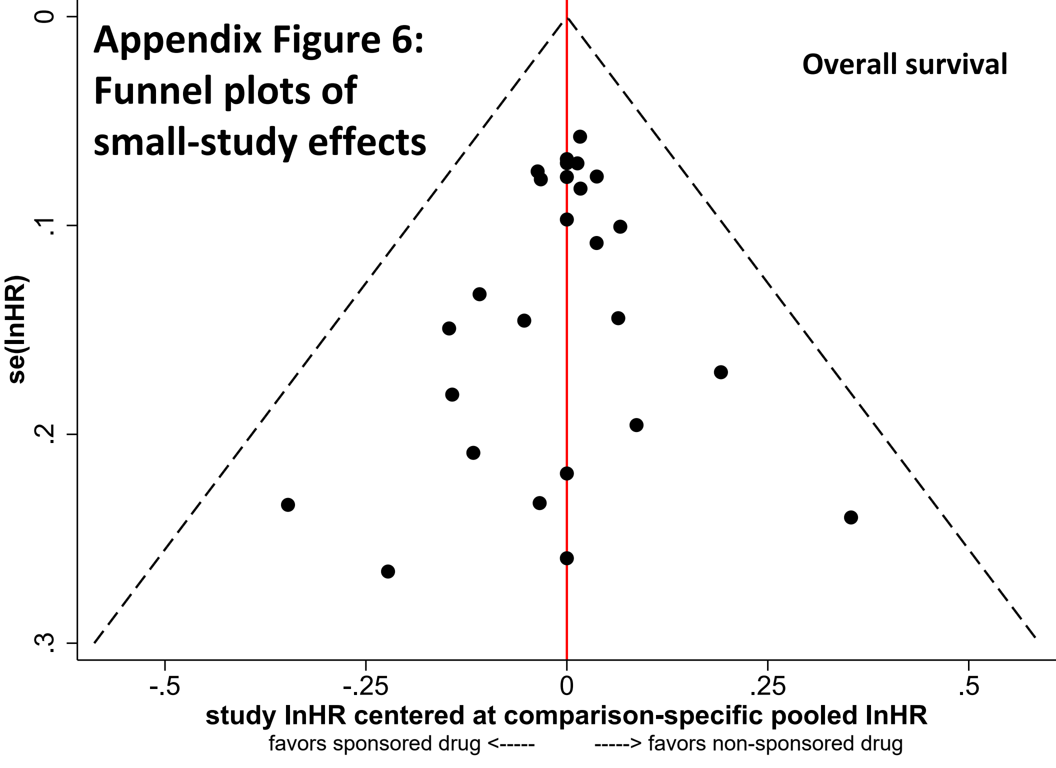
Appendix Figure 6:
Funnel plots of
small-study effects
Overall survival
se(lnHR)
0
.1
.2
.3
-.5
-.25
0
.25
.5
study lnHR centered at comparison-specific pooled lnHR
favors sponsored drug <-----
-----> favors non-sponsored drug

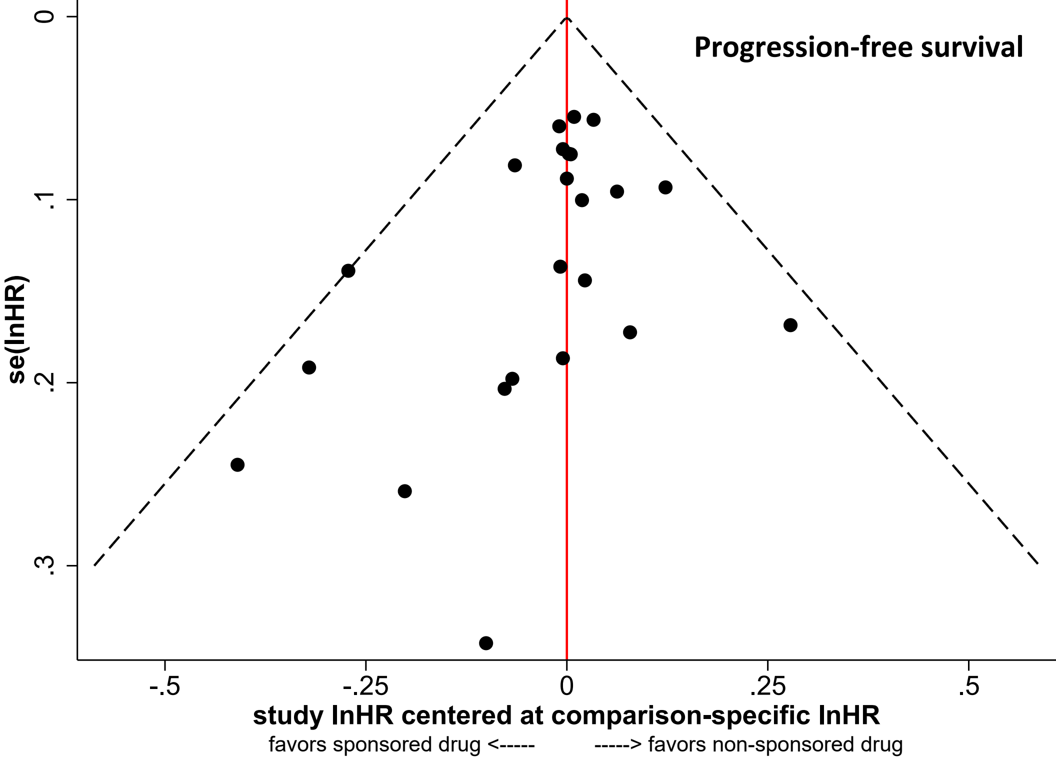
Progression-free survival
se(lnHR)
0
.1
.2
.3
-.5
-.25
0
.25
.5
study lnHR centered at comparison-specific lnHR
favors sponsored drug <-----
-----> favors non-sponsored drug

Appendix Figure 7: Overall assessment of bias

We evaluated the credibility of the evidence from network meta-analysis based on the approach suggested by *Salanti G et al. Evaluating the quality of evidence from a network meta-analysis. PloS One. 2014;9(7): e99682* that extends the GRADE system into network meta-analysis.

To evaluate study limitations we used the online tool CiNeMA available from http://ec2-52-28-232-32.eu-central-1.compute.amazonaws.com:8004/ocpu/library/contribution/www/ that combines the contribution of each direct comparison to the estimation in the network (see Chaimani et al. Graphical tools for network meta-analysis in Stata. Plos One 2013 and Krahn et al. A graphical tool for locating inconsistency in network meta-analyses. BMC Med Res Methodol 2013) with the risk of bias assessments for each direct comparison and produces a bar-graph. The bar-graph shows for the network estimate of every pairwise comparison how much information comes from low (green), moderate (yellow) or high (red) risk of bias trials.

Below we present our GRADE evaluation for the two primary outcomes and all comparisons presented in our league tables.

GRADE evaluation for Overall survival

Study limitations

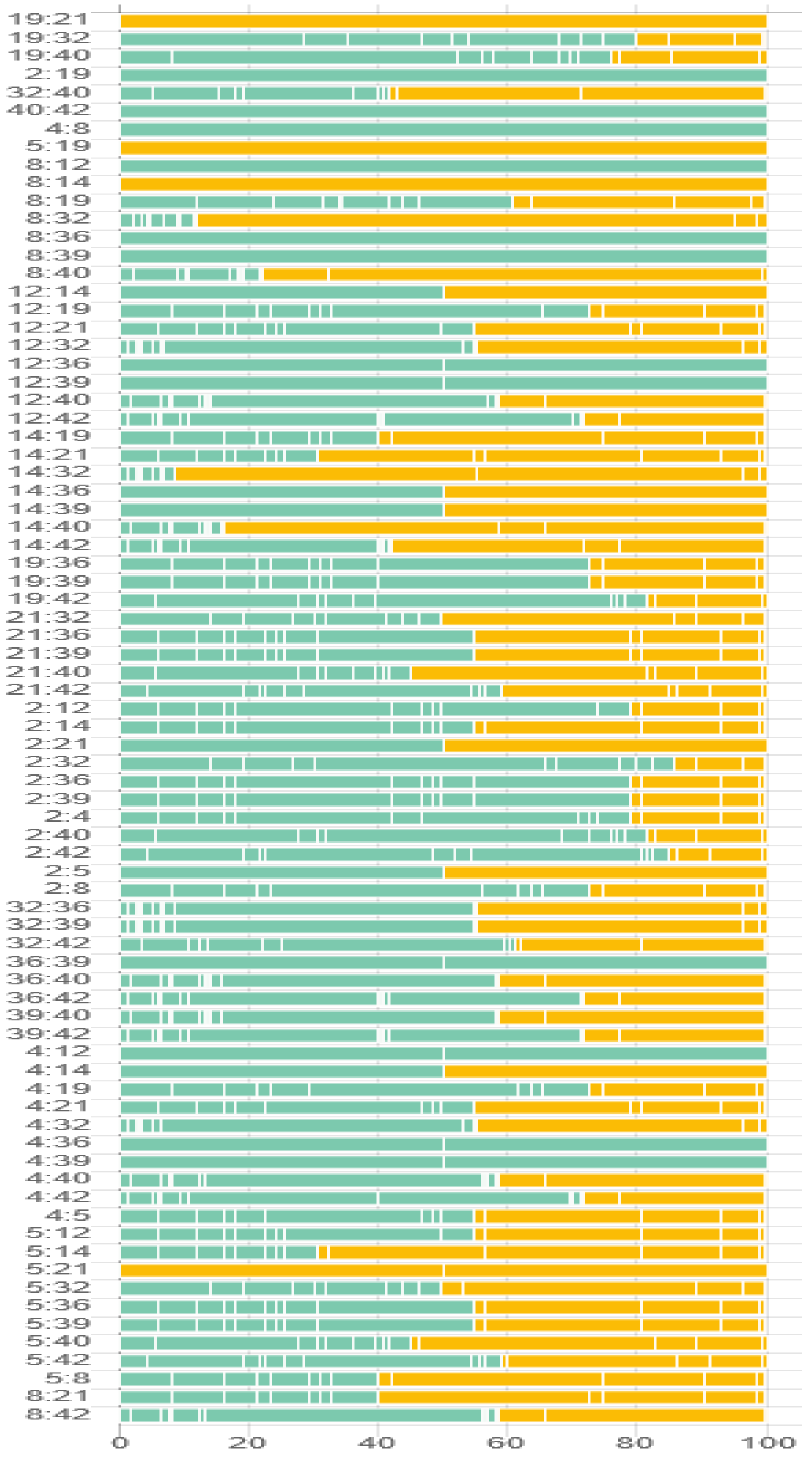

Treatment codes:

8=Docetaxel, 40=Pemetrexed, 19=Erlotinib, 32=Gefitinib, 36=Nivolumab, 39=Pembrolizumab, 4=Atezolizumab, 12=Docetaxel+Nintedanib, 14=Docetaxel+Ramucirumab, 2=Afatinib, 5=Cabozantinib, 21=Erlotinib+Cabozantinib, 42=Pemetrexed+Erlotinib

We have serious concerns for all comparisons that at least 50% of the evidence is at moderate risk of bias

Indirectness

Given the small number of trials for most comparisons the transitivity assumption could not be evaluated properly. Therefore, we have serious concerns about intransitivity for all pairwise comparisons.

Imprecision

We downgraded all non-significant HRs for imprecision.

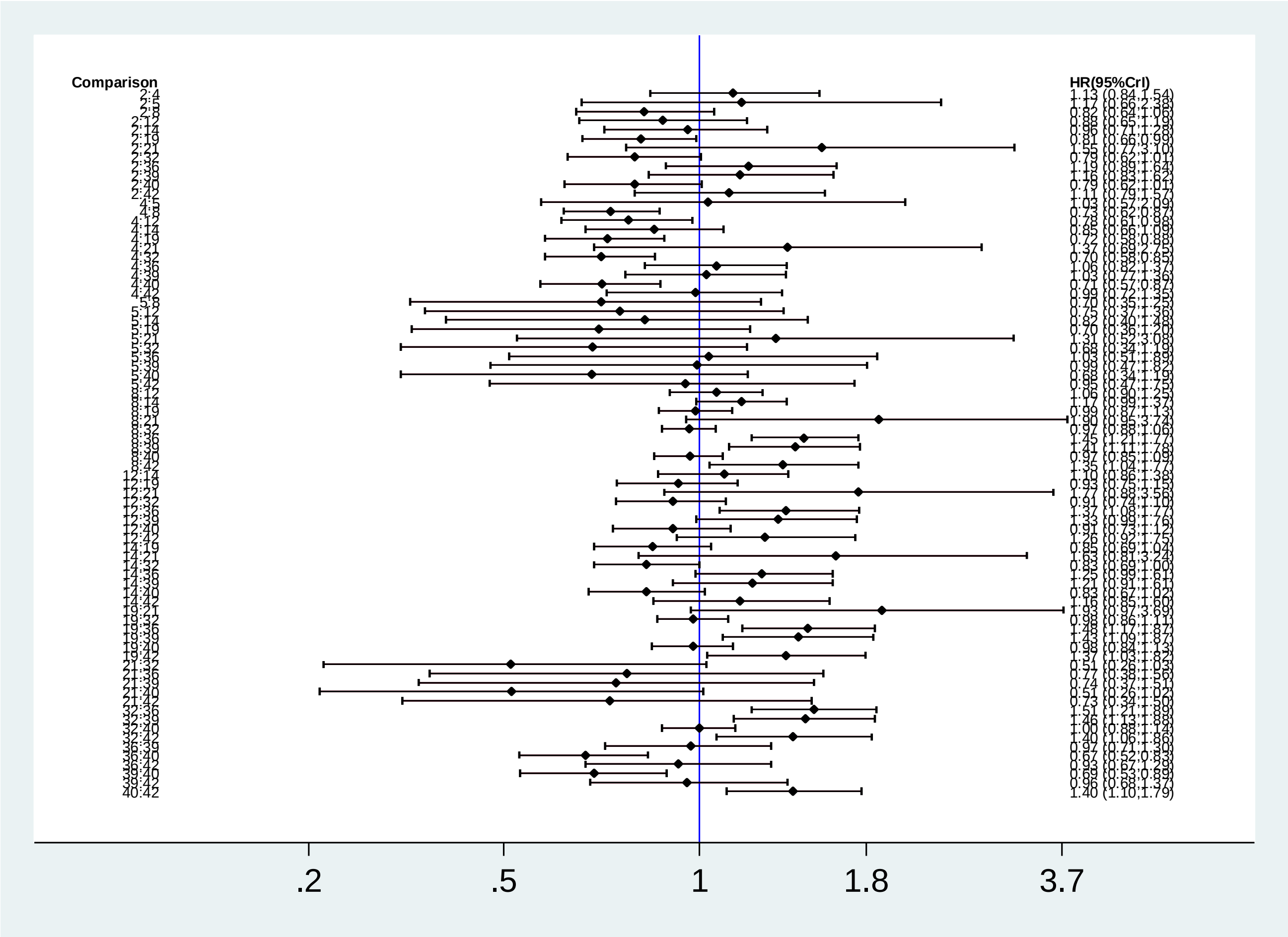

Heterogeneity

No empirical distributions for heterogeneity have been suggested for survival outcomes and therefore we rely on the prediction intervals. Given the very small value of the estimated heterogeneity (tau=0.04) the prediction intervals were almost identical with the credible intervals and therefore are not presented in the above graph.

Inconsistency

Appendix Figure 4
We have serious concerns for comparisons included in inconsistent loops.

Publication bias

Based on the comprehensive search that identified also unpublished trials we do not have serious concerns about the presence of publication bias. Appendix Figure 6 also does not imply the presence of small-study effects.

Final assessments

Comparison	Study Limitations	Indirectness	Inconsistency	Imprecision	Publications bias	GRADE	Downgraded for
2:4	No serious concerns	Serious concerns	No serious concerns	Serious concerns	No serious concerns	Moderate	Indirectness, Imprecision
2:5	No serious concerns	Serious concerns	No serious concerns	Serious concerns	No serious concerns	Moderate	Indirectness, Imprecision
2:8	No serious concerns	Serious concerns	No serious concerns	Serious concerns	No serious concerns	Moderate	Indirectness, Imprecision
2:12	No serious concerns	Serious concerns	No serious concerns	Serious concerns	No serious concerns	Moderate	Indirectness, Imprecision
2:14	No serious concerns	Serious concerns	No serious concerns	Serious concerns	No serious concerns	Moderate	Indirectness, Imprecision
2:19	No serious concerns	Serious concerns	No serious concerns	No serious concerns	No serious concerns	Moderate	Indirectness
2:21	No serious concerns	Serious concerns	No serious concerns	Serious concerns	No serious concerns	Moderate	Indirectness, Imprecision
2:32	No serious concerns	Serious concerns	No serious concerns	Serious concerns	No serious concerns	Moderate	Indirectness, Imprecision
2:36	No serious concerns	Serious concerns	No serious concerns	Serious concerns	No serious concerns	Moderate	Indirectness, Imprecision
2:39	No serious concerns	Serious concerns	No serious concerns	Serious concerns	No serious concerns	Moderate	Indirectness, Imprecision
2:40	No serious concerns	Serious concerns	No serious concerns	Serious concerns	No serious concerns	Moderate	Indirectness, Imprecision
2:42	No serious concerns	Serious concerns	No serious concerns	Serious concerns	No serious concerns	Moderate	Indirectness, Imprecision
4:5	No serious concerns	Serious concerns	No serious concerns	Serious concerns	No serious concerns	Moderate	Indirectness, Imprecision
4:8	No serious concerns	Serious concerns	No serious concerns	No serious concerns	No serious concerns	Moderate	Indirectness
4:12	No serious concerns	Serious concerns	No serious concerns	Serious concerns	No serious concerns	Moderate	Indirectness, Imprecision
4:14	No serious concerns	Serious concerns	No serious concerns	Serious concerns	No serious concerns	Moderate	Indirectness, Imprecision
4:19	No serious concerns	Serious concerns	No serious concerns	No serious concerns	No serious concerns	Moderate	Indirectness
4:21	No serious concerns	Serious concerns	No serious concerns	Serious concerns	No serious concerns	Moderate	Indirectness, Imprecision
4:32	No serious concerns	Serious concerns	No serious concerns	No serious concerns	No serious concerns	Moderate	Indirectness
4:36	No serious concerns	Serious concerns	No serious concerns	Serious concerns	No serious concerns	Moderate	Indirectness, Imprecision
4:39	No serious concerns	Serious concerns	No serious concerns	Serious concerns	No serious concerns	Moderate	Indirectness, Imprecision
4:40	No serious concerns	Serious concerns	No serious concerns	No serious concerns	No serious concerns	Moderate	Indirectness
4:42	No serious concerns	Serious concerns	No serious concerns	Serious concerns	No serious concerns	Moderate	Indirectness, Imprecision
5:8	Serious concerns	Serious concerns	No serious concerns	Serious concerns	No serious concerns	Low	Study limitations, Indirectness, Imprecision
5:12	No serious concerns	Serious concerns	No serious concerns	Serious concerns	No serious concerns	Moderate	Indirectness, Imprecision

5:14	Serious concerns	Serious concerns	No serious concerns	Serious concerns	No serious concerns	Low	Study limitations, Indirectness, Imprecision
5:19	Serious concerns	Serious concerns	No serious concerns	Serious concerns	No serious concerns	Low	Study limitations, Indirectness, Imprecision
5:21	Serious concerns	Serious concerns	No serious concerns	Serious concerns	No serious concerns	Low	Study limitations, Indirectness, Imprecision
5:32	No serious concerns	Serious concerns	No serious concerns	Serious concerns	No serious concerns	Moderate	Indirectness, Imprecision
5:36	No serious concerns	Serious concerns	No serious concerns	Serious concerns	No serious concerns	Moderate	Indirectness, Imprecision
5:39	No serious concerns	Serious concerns	No serious concerns	Serious concerns	No serious concerns	Moderate	Indirectness, Imprecision
5:40	Serious concerns	Serious concerns	No serious concerns	Serious concerns	No serious concerns	Low	Study limitations, Indirectness, Imprecision
5:42	No serious concerns	Serious concerns	No serious concerns	Serious concerns	No serious concerns	Moderate	Indirectness, Imprecision
8:12	No serious concerns	Serious concerns	No serious concerns	Serious concerns	No serious concerns	Moderate	Indirectness, Imprecision
8:14	Serious concerns	Serious concerns	No serious concerns	Serious concerns	No serious concerns	Low	Study limitations, Indirectness, Imprecision
8:19	No serious concerns	Serious concerns	No serious concerns	Serious concerns	No serious concerns	Moderate	Indirectness, Imprecision
8:21	Serious concerns	Serious concerns	No serious concerns	Serious concerns	No serious concerns	Low	Study limitations, Indirectness, Imprecision
8:32	Serious concerns	Serious concerns	No serious concerns	Serious concerns	No serious concerns	Low	Study limitations, Indirectness, Imprecision
8:36	No serious concerns	Serious concerns	No serious concerns	No serious concerns	No serious concerns	Moderate	Indirectness
8:39	No serious concerns	Serious concerns	No serious concerns	No serious concerns	No serious concerns	Moderate	Indirectness
8:40	Serious concerns	Serious concerns	No serious concerns	Serious concerns	No serious concerns	Low	Study limitations, Indirectness, Imprecision
8:42	No serious concerns	Serious concerns	No serious concerns	No serious concerns	No serious concerns	Moderate	Indirectness
12:14	No serious concerns	Serious concerns	No serious concerns	Serious concerns	No serious concerns	Moderate	Indirectness, Imprecision
12:19	No serious concerns	Serious concerns	No serious concerns	Serious concerns	No serious concerns	Moderate	Indirectness, Imprecision
12:21	No serious concerns	Serious concerns	No serious concerns	Serious concerns	No serious concerns	Moderate	Indirectness, Imprecision
12:32	No serious concerns	Serious concerns	No serious concerns	Serious concerns	No serious concerns	Moderate	Indirectness, Imprecision
12:36	No serious concerns	Serious concerns	No serious concerns	Serious concerns	No serious concerns	Moderate	Indirectness, Imprecision
12:39	No serious concerns	Serious concerns	No serious concerns	Serious concerns	No serious concerns	Moderate	Indirectness, Imprecision

12:40	No serious concerns	Serious concerns	No serious concerns	Serious concerns	No serious concerns	Moderate	Indirectness, Imprecision
12:42	No serious concerns	Serious concerns	No serious concerns	Serious concerns	No serious concerns	Moderate	Indirectness, Imprecision
14:19	Serious concerns	Serious concerns	No serious concerns	Serious concerns	No serious concerns	Low	Study limitations, Indirectness, Imprecision
14:21	Serious concerns	Serious concerns	No serious concerns	Serious concerns	No serious concerns	Low	Study limitations, Indirectness, Imprecision
14:32	Serious concerns	Serious concerns	No serious concerns	Serious concerns	No serious concerns	Low	Study limitations, Indirectness, Imprecision
14:36	No serious concerns	Serious concerns	No serious concerns	Serious concerns	No serious concerns	Moderate	Indirectness, Imprecision
14:39	No serious concerns	Serious concerns	No serious concerns	Serious concerns	No serious concerns	Moderate	Indirectness, Imprecision
14:40	Serious concerns	Serious concerns	No serious concerns	Serious concerns	No serious concerns	Low	Study limitations, Indirectness, Imprecision
14:42	Serious concerns	Serious concerns	No serious concerns	Serious concerns	No serious concerns	Low	Study limitations, Indirectness, Imprecision
19:21	Serious concerns	Serious concerns	No serious concerns	Serious concerns	No serious concerns	Low	Study limitations, Indirectness, Imprecision
19:32	No serious concerns	Serious concerns	No serious concerns	Serious concerns	No serious concerns	Moderate	Indirectness, Imprecision
19:36	No serious concerns	Serious concerns	No serious concerns	No serious concerns	No serious concerns	Moderate	Indirectness
19:39	No serious concerns	Serious concerns	No serious concerns	No serious concerns	No serious concerns	Moderate	Indirectness
19:40	No serious concerns	Serious concerns	No serious concerns	Serious concerns	No serious concerns	Moderate	Indirectness, Imprecision
19:42	No serious concerns	Serious concerns	No serious concerns	No serious concerns	No serious concerns	Moderate	Indirectness
21:32	No serious concerns	Serious concerns	No serious concerns	Serious concerns	No serious concerns	Moderate	Indirectness, Imprecision
21:36	No serious concerns	Serious concerns	No serious concerns	Serious concerns	No serious concerns	Moderate	Indirectness, Imprecision
21:39	No serious concerns	Serious concerns	No serious concerns	Serious concerns	No serious concerns	Moderate	Indirectness, Imprecision
21:40	Serious concerns	Serious concerns	No serious concerns	Serious concerns	No serious concerns	Low	Study limitations, Indirectness, Imprecision
21:42	No serious concerns	Serious concerns	No serious concerns	Serious concerns	No serious concerns	Moderate	Indirectness, Imprecision
32:36	No serious concerns	Serious concerns	No serious concerns	No serious concerns	No serious concerns	Moderate	Indirectness
32:39	No serious concerns	Serious concerns	No serious concerns	No serious concerns	No serious concerns	Moderate	Indirectness
32:40	Serious concerns	Serious concerns	No serious concerns	Serious concerns	No serious concerns	Low	Study limitations, Indirectness, Imprecision

32:42	No serious concerns	Serious concerns	No serious concerns	No serious concerns	No serious concerns	Moderate	Indirectness
36:39	No serious concerns	Serious concerns	No serious concerns	Serious concerns	No serious concerns	Moderate	Indirectness, Imprecision
36:40	No serious concerns	Serious concerns	No serious concerns	No serious concerns	No serious concerns	Moderate	Indirectness
36:42	No serious concerns	Serious concerns	No serious concerns	Serious concerns	No serious concerns	Moderate	Indirectness, Imprecision
39:40	No serious concerns	Serious concerns	No serious concerns	No serious concerns	No serious concerns	Moderate	Indirectness
39:42	No serious concerns	Serious concerns	No serious concerns	Serious concerns	No serious concerns	Moderate	Indirectness, Imprecision
40:42	No serious concerns	Serious concerns	No serious concerns	No serious concerns	No serious concerns	Moderate	Indirectness

GRADE evaluation for Progression-free survival

Study limitations

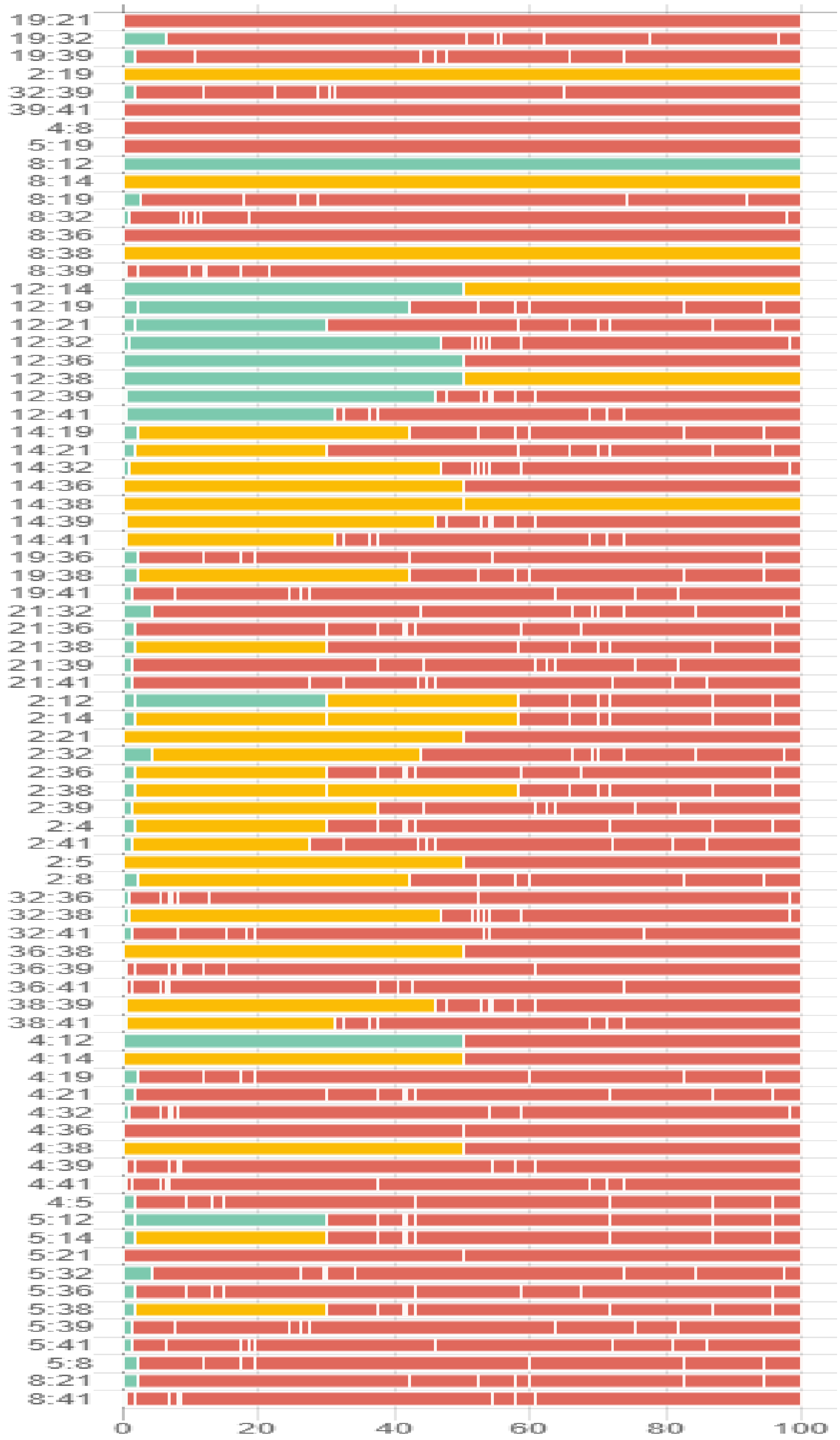

Treatment codes:

8=Docetaxel, 39=Pemetrexed, 19=Erlotinib, 32=Gefitinib, 36=Nivolumab, 38=Pembrolizumab, 4=Atezolizumab, 12=Docetaxel+Nintedanib, 14=Docetaxel+Ramucirumab, 2=Afatinib, 5=Cabozantinib, 21=Erlotinib+Cabozantinib, 41=Pemetrexed+Erlotinib

We have serious concerns for all comparisons that at least 50% of the evidence is at moderate risk of bias and very serious concerns when at least 50% of the evidence if at high risk of bias.

Indirectness

Given the small number of trials for most comparisons the transitivity assumption could not be evaluated properly. Therefore, we have serious concerns about intransitivity for all pairwise comparisons.

Imprecision

We downgraded all non-significant HRs for imprecision.

Heterogeneity

No empirical distributions for heterogeneity have been suggested for survival outcomes and therefore we rely on the prediction intervals. We downgraded comparisons that were statistically significant but the prediction interval crossed the line of no effect.

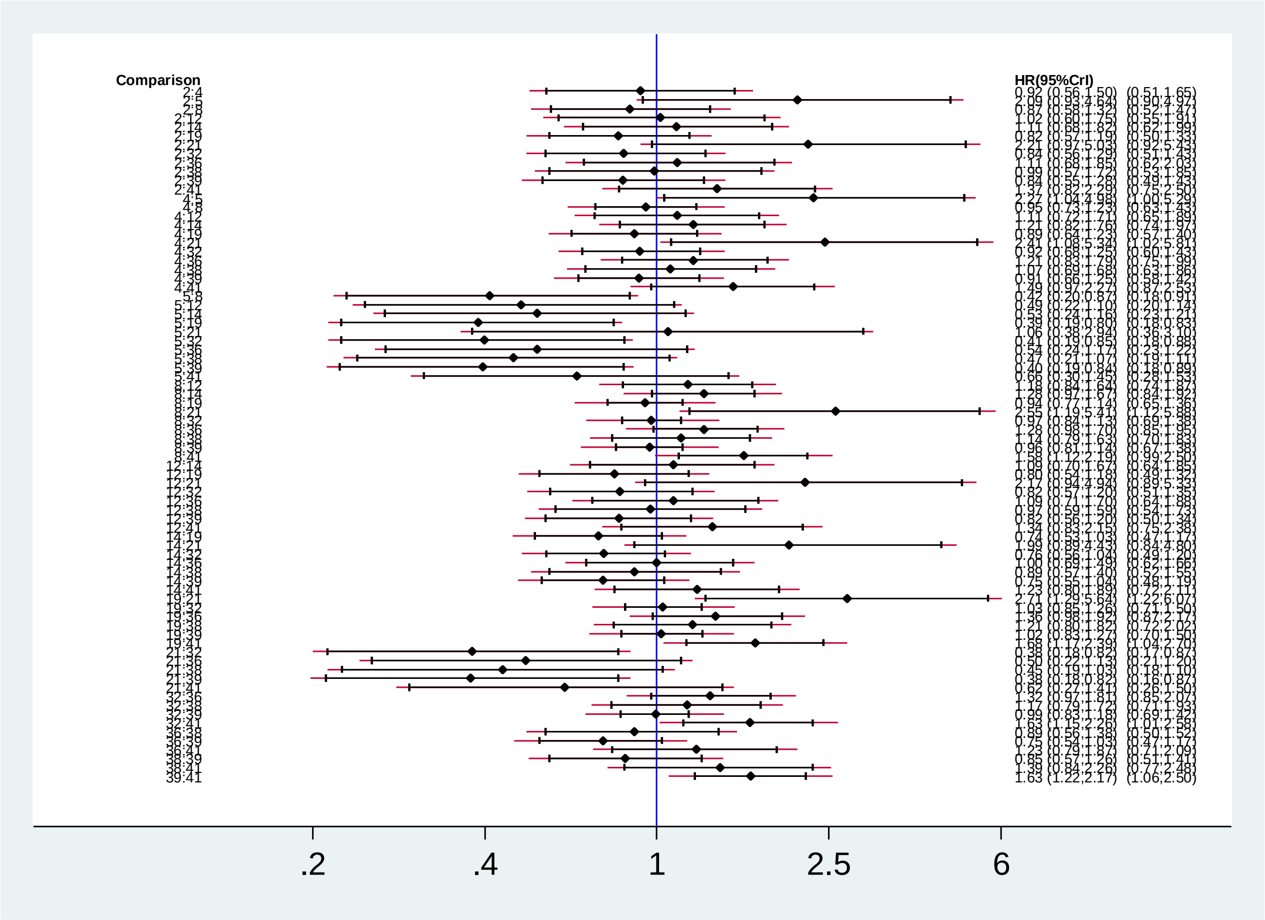

Inconsistency

Appendix Figure 4

We have serious concerns for comparisons included in inconsistent loops.

Publication bias

Appendix Figure 6 implies the presence of small-study effects which might be due to selective reporting bias and therefore we have serious concerns for all comparisons.

Final assessments

Comparison	Study Limitations	Indirectness	Inconsistency	Imprecision	Publication bias	GRADE	Downgraded for
2:4	Very serious concerns	Serious concerns	No serious concerns	Serious concerns	Serious concerns	Very low	Study limitations (2 levels), Indirectness, Imprecision, Publication bias
2:5	Serious concerns	Serious concerns	No serious concerns	Serious concerns	Serious concerns	Low	Study limitations, Indirectness, Imprecision, Publication bias
2:8	Very serious concerns	Serious concerns	No serious concerns	Serious concerns	Serious concerns	Very low	Study limitations (2 levels), Indirectness, Imprecision, Publication bias
2:12	Serious concerns	Serious concerns	No serious concerns	Serious concerns	Serious concerns	Low	Study limitations, Indirectness, Imprecision, Publication bias
2:14	Serious concerns	Serious concerns	No serious concerns	Serious concerns	Serious concerns	Low	Study limitations, Indirectness, Imprecision, Publication bias
2:19	Serious concerns	Serious concerns	No serious concerns	No serious concerns	Serious concerns	Low	Study limitations, Indirectness, Publication bias
2:21	Serious concerns	Serious concerns	No serious concerns	Serious concerns	Serious concerns	Low	Study limitations, Indirectness, Imprecision, Publication bias
2:32	Serious concerns	Serious concerns	No serious concerns	Serious concerns	Serious concerns	Low	Study limitations, Indirectness, Imprecision, Publication bias
2:36	Very serious concerns	Serious concerns	No serious concerns	Serious concerns	Serious concerns	Very low	Study limitations (2 levels), Indirectness, Imprecision, Publication bias
2:38	Serious concerns	Serious concerns	No serious concerns	Serious concerns	Serious concerns	Low	Study limitations, Indirectness, Imprecision, Publication bias
2:39	Serious concerns	Serious concerns	No serious concerns	Serious concerns	Serious concerns	Low	Study limitations, Indirectness, Imprecision, Publication bias
2:41	Very serious concerns	Serious concerns	No serious concerns	Serious concerns	Serious concerns	Very low	Study limitations (2 levels), Indirectness, Imprecision, Publication bias
4:5	Very serious concerns	Serious concerns	No serious concerns	Serious concerns	Serious concerns	Very low	Study limitations (2 levels), Indirectness, Imprecision, Publication bias
4:8	Very serious concerns	Serious concerns	No serious concerns	No serious concerns	Serious concerns	Very low	Study limitations (2 levels), Indirectness, Publication bias

4:12	Serious concerns	Serious concerns	No serious concerns	No serious concerns	Serious concerns	Low	Study limitations, Indirectness, Publication bias
4:14	Serious concerns	Serious concerns	No serious concerns	Serious concerns	Serious concerns	Low	Study limitations, Indirectness, Imprecision, Publication bias
4:19	Very serious concerns	Serious concerns	No serious concerns	No serious concerns	Serious concerns	Very low	Study limitations (2 levels), Indirectness, Publication bias
4:21	Very serious concerns	Serious concerns	No serious concerns	Serious concerns	Serious concerns	Very low	Study limitations (2 levels), Indirectness, Imprecision, Publication bias
4:32	Very serious concerns	Serious concerns	No serious concerns	No serious concerns	Serious concerns	Very low	Study limitations (2 levels), Indirectness, Publication bias
4:36	Very serious concerns	Serious concerns	No serious concerns	Serious concerns	Serious concerns	Very low	Study limitations (2 levels), Indirectness, Imprecision, Publication bias
4:38	Serious concerns	Serious concerns	No serious concerns	Serious concerns	Serious concerns	Low	Study limitations, Indirectness, Imprecision, Publication bias
4:39	Very serious concerns	Serious concerns	No serious concerns	No serious concerns	Serious concerns	Very low	Study limitations (2 levels), Indirectness, Publication bias
4:41	Very serious concerns	Serious concerns	No serious concerns	Serious concerns	Serious concerns	Very low	Study limitations (2 levels), Indirectness, Imprecision, Publication bias
5:8	Very serious concerns	Serious concerns	No serious concerns	Serious concerns	Serious concerns	Very low	Study limitations (2 levels), Indirectness, Imprecision, Publication bias
5:12	Very serious concerns	Serious concerns	No serious concerns	Serious concerns	Serious concerns	Very low	Study limitations (2 levels), Indirectness, Imprecision, Publication bias
5:14	Very serious concerns	Serious concerns	No serious concerns	Serious concerns	Serious concerns	Very low	Study limitations (2 levels), Indirectness, Imprecision, Publication bias
5:19	Very serious concerns	Serious concerns	No serious concerns	Serious concerns	Serious concerns	Very low	Study limitations (2 levels), Indirectness, Imprecision, Publication bias
5:21	Very serious concerns	Serious concerns	No serious concerns	Serious concerns	Serious concerns	Very low	Study limitations (2 levels), Indirectness, Imprecision, Publication bias
5:32	Very serious concerns	Serious concerns	No serious concerns	Serious concerns	Serious concerns	Very low	Study limitations (2 levels), Indirectness, Imprecision, Publication bias
5:36	Very serious concerns	Serious concerns	No serious concerns	Serious concerns	Serious concerns	Very low	Study limitations (2 levels), Indirectness, Imprecision, Publication bias

5:38	Very serious concerns	Serious concerns	No serious concerns	Serious concerns	Serious concerns	Very low	Study limitations (2 levels), Indirectness, Imprecision, Publication bias
5:39	Very serious concerns	Serious concerns	No serious concerns	Serious concerns	Serious concerns	Very low	Study limitations (2 levels), Indirectness, Imprecision, Publication bias
5:41	Very serious concerns	Serious concerns	No serious concerns	Serious concerns	Serious concerns	Very low	Study limitations (2 levels), Indirectness, Imprecision, Publication bias
8:12	No serious concerns	Serious concerns	No serious concerns	Serious concerns	Serious concerns	Low	Indirectness, Imprecision, Publication bias
8:14	Serious concerns	Serious concerns	No serious concerns	Serious concerns	Serious concerns	Low	Study limitations, Indirectness, Imprecision, Publication bias
8:19	Very serious concerns	Serious concerns	Serious concerns (incoherence)	Serious concerns	Serious concerns	Very low	Study limitations (2 levels), Indirectness, Inconsistency, Publication bias
8:21	Very serious concerns	Serious concerns	No serious concerns	Serious concerns	Serious concerns	Very low	Study limitations (2 levels), Indirectness, Imprecision, Publication bias
8:32	Very serious concerns	Serious concerns	Serious concerns (heterogeneity)	Serious concerns	Serious concerns	Very low	Study limitations (2 levels), Indirectness, Inconsistency, Publication bias
8:36	Very serious concerns	Serious concerns	No serious concerns	No serious concerns	Serious concerns	Very low	Study limitations (2 levels), Indirectness, Publication bias
8:38	Serious concerns	Serious concerns	No serious concerns	No serious concerns	Serious concerns	Low	Study limitations, Indirectness, Publication bias
8:39	Very serious concerns	Serious concerns	Serious concerns (incoherence)	Serious concerns	Serious concerns	Very low	Study limitations (2 levels), Indirectness, Inconsistency, Publication bias
8:41	Very serious concerns	Serious concerns	No serious concerns	No serious concerns	Serious concerns	Very low	Study limitations (2 levels), Indirectness, Publication bias
12:14	No serious concerns	Serious concerns	No serious concerns	Serious concerns	Serious concerns	Low	Indirectness, Imprecision, Publication bias
12:19	Very serious concerns	Serious concerns	No serious concerns	Serious concerns	Serious concerns	Very low	Study limitations (2 levels), Indirectness, Imprecision, Publication bias
12:21	Very serious concerns	Serious concerns	No serious concerns	Serious concerns	Serious concerns	Very low	Study limitations (2 levels), Indirectness, Imprecision, Publication bias
12:32	Serious concerns	Serious concerns	No serious concerns	Serious concerns	Serious concerns	Low	Study limitations, Indirectness, Imprecision, Publication bias
12:36	Serious concerns	Serious concerns	No serious concerns	Serious concerns	Serious concerns	Low	Study limitations, Indirectness,

							Imprecision, Publication bias
12:38	No serious concerns	Serious concerns	No serious concerns	Serious concerns	Serious concerns	Low	Indirectness, Imprecision, Publication bias
12:39	Serious concerns	Serious concerns	No serious concerns	Serious concerns	Serious concerns	Low	Study limitations, Indirectness, Imprecision, Publication bias
12:41	Very serious concerns	Serious concerns	No serious concerns	Serious concerns	Serious concerns	Very low	Study limitations (2 levels), Indirectness, Imprecision, Publication bias
14:19	Serious concerns	Serious concerns	No serious concerns	Serious concerns	Serious concerns	Low	Study limitations, Indirectness, Imprecision, Publication bias
14:21	Very serious concerns	Serious concerns	No serious concerns	Serious concerns	Serious concerns	Very low	Study limitations (2 levels), Indirectness, Imprecision, Publication bias
14:32	Serious concerns	Serious concerns	No serious concerns	Serious concerns	Serious concerns	Low	Study limitations, Indirectness, Imprecision, Publication bias
14:36	Serious concerns	Serious concerns	No serious concerns	Serious concerns	Serious concerns	Low	Study limitations, Indirectness, Imprecision, Publication bias
14:38	Serious concerns	Serious concerns	No serious concerns	Serious concerns	Serious concerns	Low	Study limitations, Indirectness, Imprecision, Publication bias
14:39	Serious concerns	Serious concerns	No serious concerns	Serious concerns	Serious concerns	Low	Study limitations, Indirectness, Imprecision, Publication bias
14:41	Very serious concerns	Serious concerns	No serious concerns	Serious concerns	Serious concerns	Very low	Study limitations (2 levels), Indirectness, Imprecision, Publication bias
19:21	Very serious concerns	Serious concerns	No serious concerns	Serious concerns	Serious concerns	Very low	Study limitations (2 levels), Indirectness, Imprecision, Publication bias
19:32	Very serious concerns	Serious concerns	Serious concerns (incoherence)	Serious concerns	Serious concerns	Very low	Study limitations (2 levels), Indirectness, Inconsistency, Imprecision, Publication bias
19:36	Very serious concerns	Serious concerns	No serious concerns	No serious concerns	Serious concerns	Very low	Study limitations (2 levels), Indirectness, Publication bias
19:38	Serious concerns	Serious concerns	No serious concerns	No serious concerns	Serious concerns	Low	Study limitations, Indirectness, Publication bias
19:39	Very serious concerns	Serious concerns	No serious concerns	Serious concerns	Serious concerns	Very low	Study limitations (2 levels), Indirectness, Imprecision, Publication bias

19:41	Very serious concerns	Serious concerns	No serious concerns	No serious concerns	Serious concerns	Very low	Study limitations (2 levels), Indirectness, Publication bias
21:32	Very serious concerns	Serious concerns	No serious concerns	Serious concerns	Serious concerns	Very low	Study limitations (2 levels), Indirectness, Imprecision, Publication bias
21:36	Very serious concerns	Serious concerns	No serious concerns	Serious concerns	Serious concerns	Very low	Study limitations (2 levels), Indirectness, Imprecision, Publication bias
21:38	Very serious concerns	Serious concerns	No serious concerns	Serious concerns	Serious concerns	Very low	Study limitations (2 levels), Indirectness, Imprecision, Publication bias
21:39	Very serious concerns	Serious concerns	No serious concerns	Serious concerns	Serious concerns	Very low	Study limitations (2 levels), Indirectness, Imprecision, Publication bias
21:41	Very serious concerns	Serious concerns	No serious concerns	Serious concerns	Serious concerns	Very low	Study limitations (2 levels), Indirectness, Imprecision, Publication bias
32:36	Very serious concerns	Serious concerns	No serious concerns	No serious concerns	Serious concerns	Very low	Study limitations (2 levels), Indirectness, Publication bias
32:38	Serious concerns	Serious concerns	No serious concerns	No serious concerns	Serious concerns	Low	Study limitations, Indirectness, Publication bias
32:39	Very serious concerns	Serious concerns	No serious concerns	Serious concerns	Serious concerns	Very low	Study limitations (2 levels), Indirectness, Imprecision, Publication bias
32:41	Very serious concerns	Serious concerns	No serious concerns	No serious concerns	Serious concerns	Very low	Study limitations (2 levels), Indirectness, Publication bias
36:38	Serious concerns	Serious concerns	No serious concerns	Serious concerns	Serious concerns	Low	Study limitations, Indirectness, Imprecision, Publication bias
36:39	Very serious concerns	Serious concerns	No serious concerns	No serious concerns	Serious concerns	Very low	Study limitations (2 levels), Indirectness, Publication bias
36:41	Very serious concerns	Serious concerns	No serious concerns	Serious concerns	Serious concerns	Very low	Study limitations (2 levels), Indirectness, Imprecision, Publication bias
38:39	Serious concerns	Serious concerns	No serious concerns	No serious concerns	Serious concerns	Low	Study limitations, Indirectness, Publication bias
38:41	Very serious concerns	Serious concerns	No serious concerns	Serious concerns	Serious concerns	Very low	Study limitations (2 levels), Indirectness, Imprecision, Publication bias
39:41	Very serious concerns	Serious concerns	No serious concerns	No serious concerns	Serious concerns	Very low	Study limitations (2 levels), Indirectness, Publication bias
